# Catalytic Enantioselective 6π Photocyclization of Acrylanilides

**DOI:** 10.1021/jacs.2c09267

**Published:** 2022-12-26

**Authors:** Benjamin A. Jones, Pearse Solon, Mihai V. Popescu, Ji-Yuan Du, Robert Paton, Martin D. Smith

**Affiliations:** † Chemistry Research Laboratory, University of Oxford, 12 Mansfield Road, Oxford, OX1 3TA, U.K.; ‡ Department of Chemistry, Colorado State University, 1301 Center Avenue, Ft. Collins, Colorado 80523-1872, United States

## Abstract

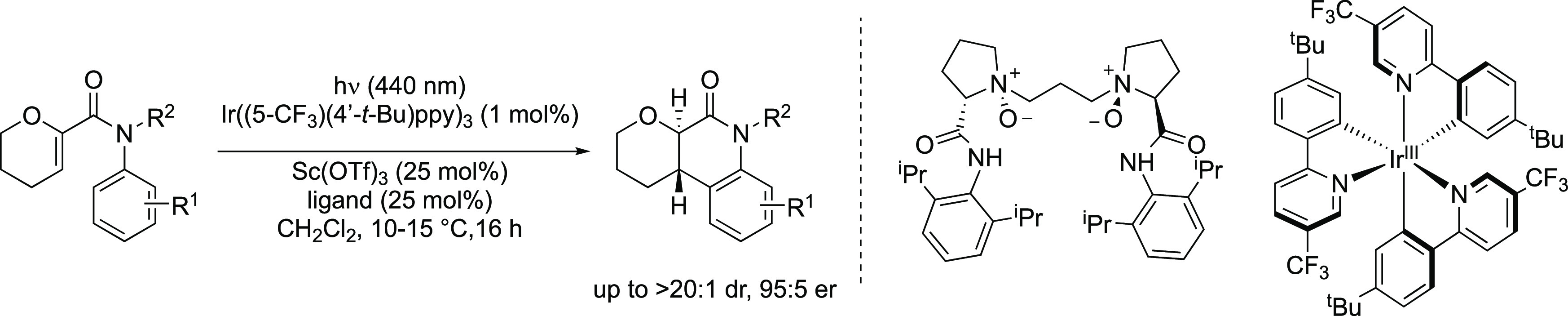

Controlling absolute stereochemistry in catalytic photochemical reactions is generally challenging owing to high rates of background reactivity. Successful strategies broadly rely on selective excitation of the reaction substrate when associated with a chiral catalyst. Recent studies have demonstrated that chiral Lewis acid complexes can enable selective energy transfer from a photosensitizer to facilitate enantioselective triplet state reactions. Here, we apply this approach to the enantioselective catalysis of a 6π photocyclization through the design of an iridium photosensitizer optimized to undergo energy transfer to a reaction substrate only in the presence of a chiral Lewis acid complex. Among a group of iridium(III) sensitizers, enantioselectivity and yield closely correlate with photocatalyst triplet energy within a narrow window enabled by a modest reduction in substrate triplet energy upon binding a scandium/ligand complex. These results demonstrate that photocatalyst tuning offers a means to suppress background reactivity and improve enantioselectivity in photochemical reactions.

## Introduction

**Figure 1:**
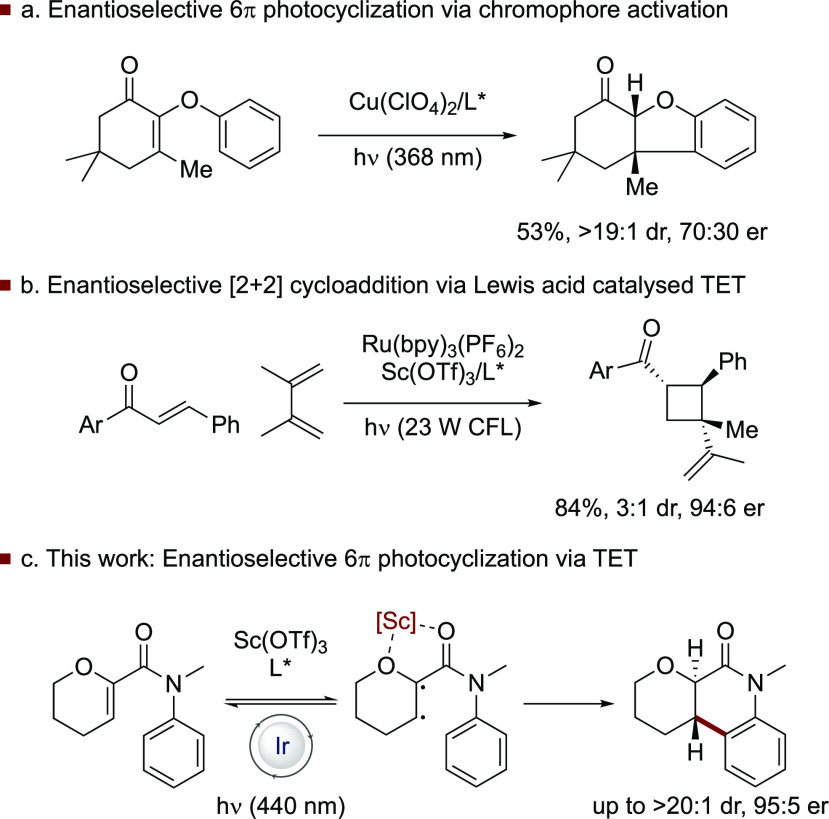
Enantioselective photocyclizations. (a) Enantioselective 6π photocyclization via a bathochromic shift in the absorption of the substrate upon coordination to a chiral Lewis acid enabling selective excitation. (b) Enantioselective [2+2] photocycloaddition via Lewis acid-lowering of triplet energy enabling selective energy transfer. (c) This work: enantioselective 6π photocyclization via TET (TET = triplet energy transfer; L* = chiral ligand; CFL = compact fluorescent lamp).

Photocyclization reactions constitute an important class of transformations with demonstrated application in complex molecule synthesis.^1^ Pioneering work by Schultz^2^ and Chapman^3^ developed the 6π photocyclization manifold from its origins in the excited state electrocyclization of stilbene^4^ into a useful class of reactions for the synthesis of aliphatic heterocycles.^5^ In spite of the synthetic potential of 6π photocyclization reactions, the necessity for high-energy UV irradiation to achieve excitation of typical chromophores has meant their broader application in synthesis remains limited. Recent studies by our group and others have demonstrated the possibility of performing triplet state 6π photocyclizations with more benign and readily available visible light sources by employing catalytic photosensitizers.^6^ While this approach renders such reactions more attractive, the challenge of promoting these transformations in an enantioselective fashion remains an enduring goal. Chiral auxiliary-based approaches,^7^ the application of chiral templating host compounds^8^ and reactions exploiting axial to point chirality transfer,^9^ have been disclosed, and studies investigating enantioselective catalysis of 6π photocyclizations are limited.^10^ Chromophore activation using chiral Lewis acid complexes has emerged as a viable strategy to induce enantioselectivity in photochemical reactions. In this approach, coordination of a Lewis acid to the reaction substrate results in a bathochromic shift in the UV/vis absorption, thereby facilitating selective excitation within the chiral environment and minimizing racemic background reactivity arising from direct excitation of the reaction substrate.^11,12^ In the 6π arena, the Bach group applied a chromophore activation strategy^10a^ to the enantioselective catalysis of Schultz’s α-aryloxyenone photocyclization (Figure 1a).^2^ Yoon has demonstrated that highly enantioselective [2+2] cycloadditions are possible via a related strategy involving dual Lewis acid and photocatalysis to lower the triplet energy of a coordinated substrate (Figure 1b).^13^ Further work from Yoon has identified a kinetic contribution to the rate of energy transfer arising from improved orbital overlap between the Lewis acid complexed substrate and the photosensitizer.^14^

Here we demonstrate that Lewis acid-catalyzed triplet energy transfer offers a viable approach to enantioselective 6π photocyclization. The synthesis and application of a modified photocatalyst designed to engage in triplet energy transfer to the reaction substrate only in the presence of a chiral Lewis acid complex was key to the success of this strategy. The reaction proceeds in high yield and diastereo- and enantioselectivity to yield a range of substituted dihydroquinolones (Figure 1c).

## Results and Discussion

Our investigation began with a modification of the acrylanilide photocyclization originally reported by Chapman.^3a−3c^ We envisaged that a Lewis acid catalyzed energy transfer mechanism^15^ analogous to that developed by Yoon (for enantioselective [2+2] photocycloadditions)^13^ could offer a viable route to an enantioselective photocyclization. However, we also recognized that an electron transfer process^15^ (via a radical anion cyclization) could also be operative.

In early experiments, we found that irradiation with a 440 nm LED light source in the presence of Ir(dFppy)_3_ led to high conversion of substrate **1** to product **2** (Table 1, entry 1). Ir(ppy)_3_, which possesses markedly lower triplet energy, also led to the formation of product **2**, albeit in significantly lower conversion (6%, entry 2). This could be augmented to 68% yield when the reaction was performed in the presence of scandium(III) triflate (entry 3), consistent with Lewis acid acceleration of the cyclization vs the background reaction. We consequently explored a range of different Lewis acid and ligand combinations, and we were able to identify the Feng ligand **3**(16,17) as a promising ligand for an enantioselective transformation in combination with Ir(ppy)_3_ (75:25 er, entry 4; see SI p S6 for extended optimization across a pair of substrates). This combination of ligand and Lewis acid did not lead to any significant enantioselectivity with photocatalyst Ir(dFppy)_3_, likely due to a high rate of competing background reactivity (entry 5). We also examined the efficacy of a series of cationic iridium-based photocatalysts spanning a range of triplet energies from 56.4 to 63.3 kcal·mol^–1^. As expected, the reaction yield increased with increasing triplet energy; however, low enantioselectivity was observed in every case. It appears that cationic iridium sensitizers in combination with scandium(III) complexes are not viable for this enantioselective process (see SI p S6 for details). We reasoned that the increased enantioselectivity observed with Ir(ppy)_3_ (*E*_T_ = 59.4 kcal mol^–1^) vs Ir(dFppy)_3_ (*E*_T_ = 63.8 kcal mol^–1^) as photocatalyst is due primarily to the lower triplet energy of Ir(ppy)_3_. A consequence of this is that triplet energy transfer to the substrate in the absence of the Lewis acid complex is lower with this photocatalyst than it is with Ir(dFppy)_3_. Consequently, we proposed that the enantioselectivity of the reaction could potentially be improved using related photocatalysts with a lower triplet energy.

**Table 1:**
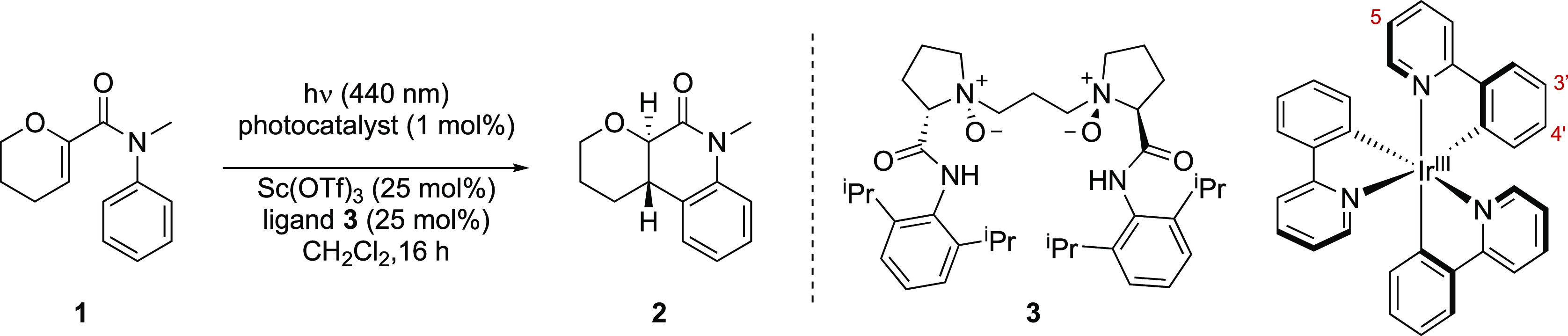
Reaction Optimization^a^

entry	photocatalyst	*E*_T_ (λ_10%_) (kcal·mol^**–**1^)	*E*_1/2_ Ir^IV/*III^ (V)	Lewis acid/ligand	yield (%)	er	dr
**1**	Ir(dFppy)_3_	63.8	–1.83	none	85		>20:1
**2**	Ir(ppy)_3_	59.4	–1.81	none	6		>20:1
**3**	Ir(ppy)_3_	59.4	–1.81	Sc(OTf)_3_/none	68		4:1
**4**	Ir(ppy)_3_	59.4	–1.81	Sc(OTf)_3_/**3**	67	75:25	>20:1
**5**	Ir(dFppy)_3_	63.8	–1.83	Sc(OTf)_3_/**3**	95	53:47	>20:1
**6**	Ir((5-F)ppy)_3_	59.6	–1.74	Sc(OTf)_3_/**3**	84	86:14	>20:1
**7**	Ir((5-F)(4′-*t*-Bu)ppy)_3_	58.8	–1.80	Sc(OTf)_3_/**3**	84	84:16	>20:1
**8**	Ir((5-CF_3_)ppy)_3_	57.0	–1.50	Sc(OTf)_3_/**3**	92	91:9	>20:1
**9**	Ir((5-CF_3_)(4′-*t*-Bu)ppy)_3_	55.7	–1.55	Sc(OTf)_3_/**3**	95^b^	95:5	>20:1
**10**	Ir((5-CF_3_)(4′-*t*-Bu)ppy)_3_	55.7	–1.55	none			

a Conditions: **1** (0.1 mmol), photocatalyst (1 mol %), Sc(OTf)_3_ (25 mol %), ligand **3** (25 mol %), CH_2_Cl_2_ ([acrylanilide] = 0.02 M); 440 nm LED, reaction time 16 h, 10–15 °C. *E*_T_ = triplet energy calculated from λ_10%_. Diastereomeric ratios were determined by ^1^H NMR analysis of the crude reaction mixture. Enantiomeric ratios were determined by chiral stationary phase HPLC. Yields were determined by quantitative ^1^H NMR analysis using 1,3,5-trimethoxybenzene as an internal standard.

b Refers to isolated yield of purified product.

With this goal in mind, we set about designing iridium photocatalysts with lower triplet energy that would participate preferentially in energy transfer to the substrate–Lewis acid complex while leaving the free substrate unaffected. For 2-phenylpyridine-derived ligands in general, electron-withdrawing groups on the pyridine lower the energy of the LUMO of these complexes (resulting in a smaller HOMO–LUMO gap) and a lower triplet energy. Conversely, electron-donating groups on the phenyl ring raise the energy of the HOMO, thereby also lowering the triplet energy.^18^

As expected, Ir((5-F)ppy)_3_ has a lower triplet energy than Ir(ppy)_3_. Gratifyingly, this catalyst delivered the product with improved enantioselectivity (86:14 er, 84% yield, entry 6). Incorporating *tert*-butyl groups at the 4′-position lowered the triplet energy further; this gave similar yield and enantioselectivity (84:16 er, 84% yield, entry 7). Using CF_3_ substituents as stronger electron-withdrawing groups at the 5-position, as in Ir((5-CF_3_)ppy)_3_, lowered the triplet energy to 57.0 kcal·mol^–1^ to give the product in improved yield and selectivity (91:9 er, 92% yield, entry 8). Incorporating a *tert*-butyl group at the 4′-position gave the optimal catalyst, Ir((5-CF_3_)(4′-*t-*Bu)ppy)_3_, **4**, which has a lower triplet energy of 55.7 kcal·mol^–1^. Under the optimal reaction conditions, product **2** was isolated as a single diastereomer in 95% yield in 95:5 er (entry 9). We confirmed that photocatalyst **4** did not mediate the cyclization reaction in the absence of the Lewis acid and ligand combination, leading only to recovered starting material (entry 10). Having identified suitable conditions for the enantioselective photocyclization, we next explored the scope of the reaction (Table 2). The anilide nitrogen can be substituted with a range of alkyl groups such as benzyl **5** (97% yield, >20:1 dr, 94:6 er), isobutyl **6** (92%, >20:1 dr, 93:7 er), and propyl **7** (85% yield, >20:1 dr, 95:5 er) without compromising the efficiency of the reaction.

**Table 2:**
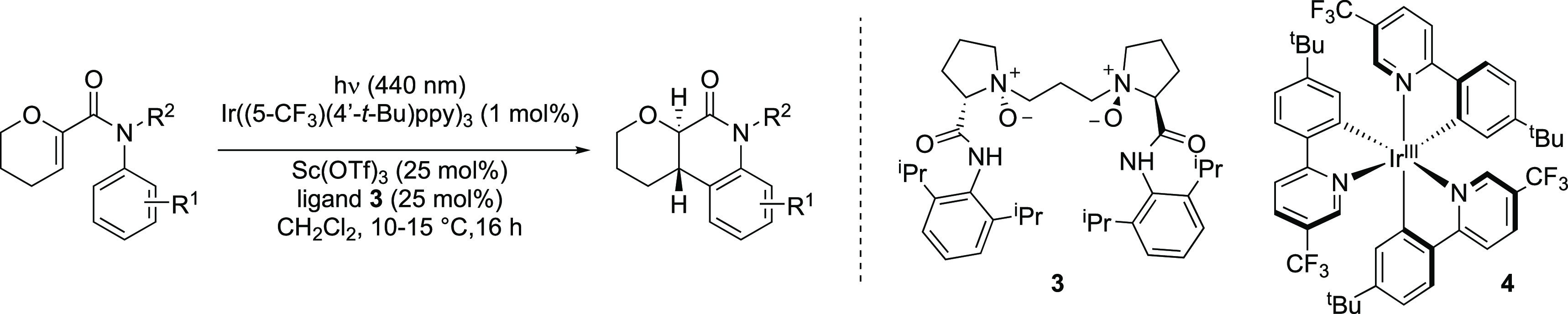

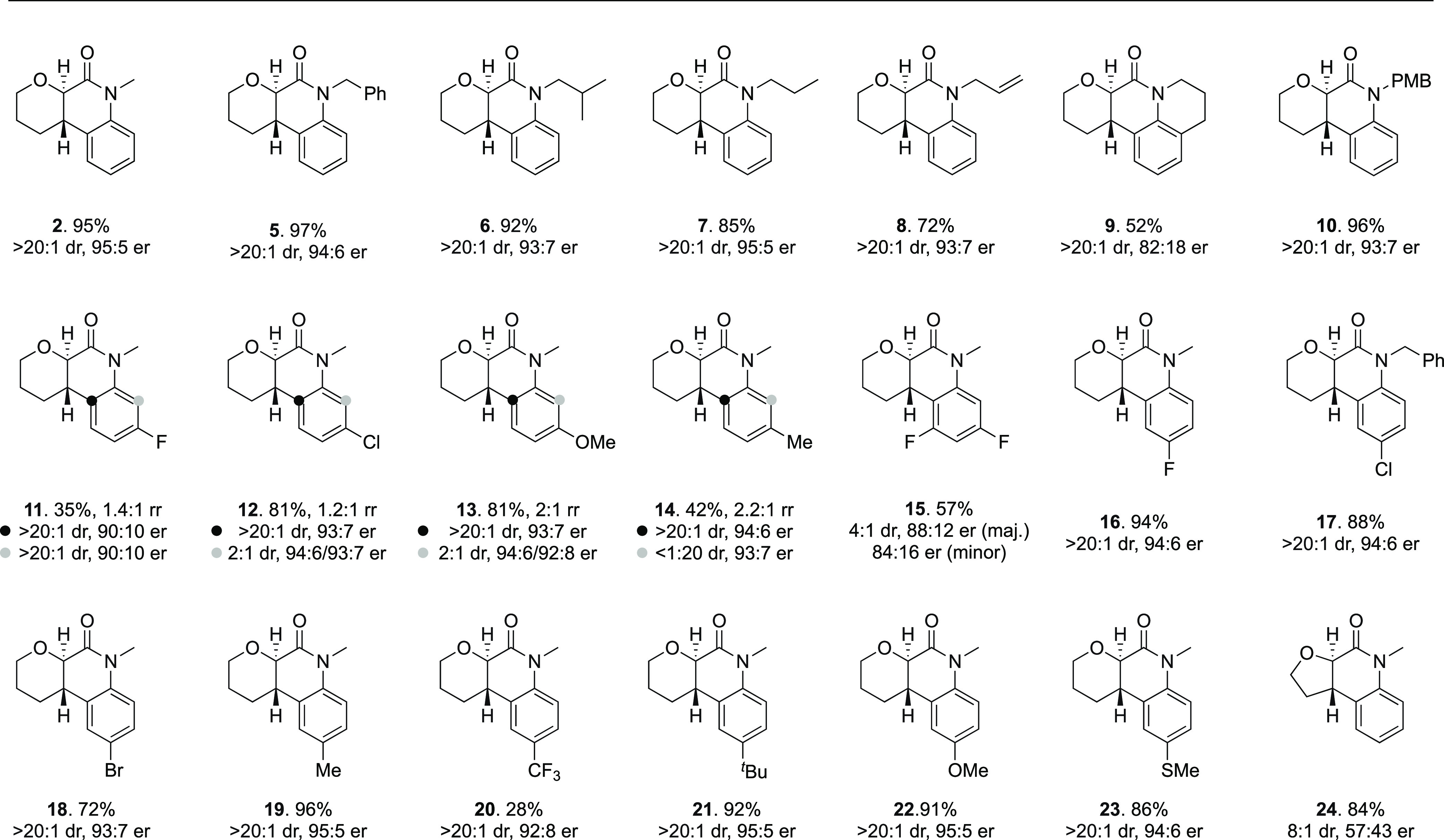
Scope of Enantioselective [6π] Photocyclization^a^

a Conditions: acrylanilide (0.1 mmol), Ir((5-CF_3_)(4′-*t-*Bu)ppy)_3_**4** (1 mol %), Sc(OTf)_3_ (25 mol %), ligand **3** (25 mol %), CH_2_Cl_2_ ([acrylanilide] = 0.02 M); 440 nm LED, reaction time 16 h, 10–15 °C. Diastereomeric (dr) and regioisomeric ratios (rr) were determined by ^1^H NMR analysis of the crude reaction mixture. Enantiomeric ratios (er) were determined by chiral stationary phase HPLC. Yields are for isolated and purified material.

Unsaturation in the *N*-substituent is also tolerated without prejudice to yield **8** (72% yield, 93:7 er). Substituting the anilide nitrogen with bulky groups or introducing substitution at the 2-position of the aryl group leads to 1,3-allylic strain in the cyclization transition state, resulting in poor conversion of starting material. This can be partially remedied using a tethering approach, as in the case of the tetrahydroquinoline derivative **9**, which cyclized in moderate yield and enantioselectivity (52% yield, >20:1 dr, 82:18 er). More electron-rich *N*-substituents such as *para*-methoxybenzyl **10** also cyclize without incident (96% yield, 93:7 er). In the case of 3-substituted aryl groups, cyclization onto either *ortho*-position is viable, resulting in the formation of two regioisomers. This is exemplified in the formation of **11** in 35% yield and a 1.4:1 mixture of regioisomers. Both regioisomers are generated with high levels of enantio- and diastereoselectivity (>20:1 dr, 90:10 er; and >20:1 dr, 90:10 er). When substituents in this position are larger, such as in chloride **12**, the major regioisomer in which the chlorine substituent is distal to the tetrahydropyran ring (THP) is produced with high levels of selectivity (>20:1 dr, 93:7 er). The minor regioisomer, where the chlorine is proximal to the THP, is produced in significantly lower diastereoselectivity but high enantioselectivity (2:1 dr, 94:6 er/93:7 er). This results from strain between the aryl chloride and the dihydropyran ring. This pattern is broadly similar for the cyclization of 3-methoxy-substituted anilide **13** (2:1 rr, >20:1 dr, 93:7 er; and 2:1 dr, 94:6 er/92:8 er), which cyclized to give a regioisomeric mixture (wherein the minor regioisomer was formed as a mixture of diastereomers). In contrast, the cyclization of the 3-methyl substrate led to **14** (2.2:1 rr, >20:1 dr, 94:6 er; and <1:20 dr, 93:7 er) with high levels of enantioselectivity but a complete reversal of diastereoselectivity in the minor regioisomer, so that the *cis-*isomer now dominates. This is most likely due to intermolecular protonation of an enolate in preference to the suprafacial 1,5-shift (see SI p S69 for details of deuteration experiments to support this).^8,19^ A 3,5-substitution pattern addresses the issue of regioselectivity (see 3,5-difluorinated substrate **15**). However, strain leads to diminished yield, enantioselectivity, and diastereoselectivity (57% yield, 4:1 dr, 88:12 er for the major *trans*-diastereoisomer). The 4-position of the phenyl group can be substituted with a variety of functional groups; fluoro **16** (>20:1 dr, 94:6 er), chloro **17** (>20:1 dr, 94:6 er), bromo **18** (>20:1 dr, 93:7 er), methyl **19** (>20:1 dr, 95:5 er), trifluoromethyl **20** (>20:1 dr, 92:8 er), *tert*-butyl **21** (>20:1 dr, 95:5 er), methoxy **22** (>20:1 dr, 95:5 er), and methylthio groups **23** (>20:1 dr, 94:6 er) were all well tolerated. We were able to confirm the absolute configuration of these products by X-ray crystallographic analysis of **17** (CCDC 2192820; see SI p S74). Finally, we examined the effect of ring size on the reaction. Tetrahydrofuran product **24** was delivered in high yield and dr but substantially reduced enantioselectivity (8:1 dr, 58:42 er), consistent with a fast background energy transfer mediated cyclization; this is consistent with our observation that conversion to product **24** occurs in the absence of scandium triflate and ligand. The ring-expanded oxepan analogue failed to cyclize in appreciable yield (<5% yield) but with high selectivity (>20:1 dr, 91:9 er; see SI p S56 for more details). Other substrates that did not cyclize successfully are detailed in the Supporting Information (p 58).

**Figure 2:**
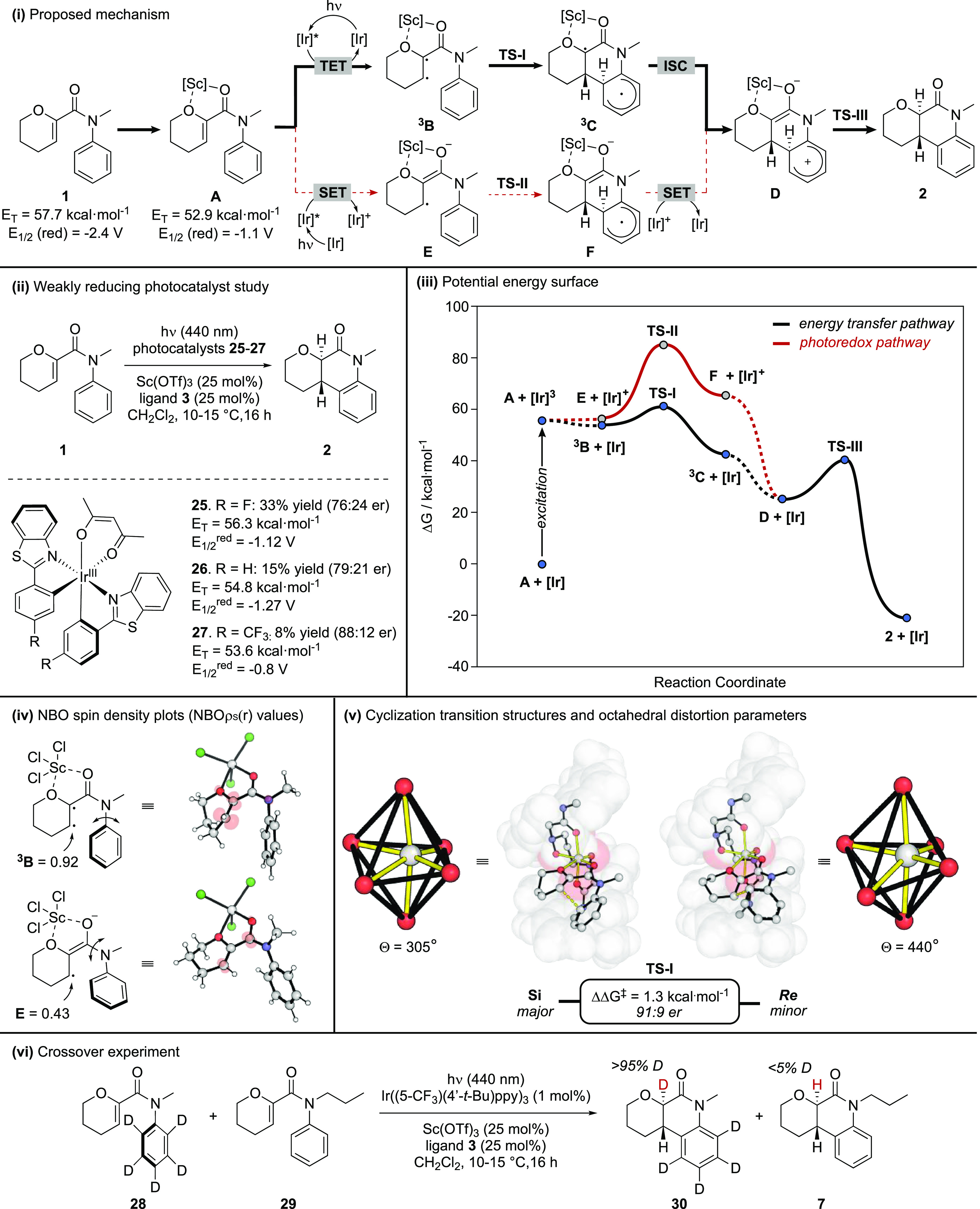
Mechanistic studies. (i) Proposed reaction mechanism. (ii) Study of weakly reducing photocatalyst efficacy. (iii) The potential energy surface was computed for substrate **1**-ScCl_3_ Lewis acid complex. Computational studies performed using the M06-2X-D3/Def2-TZVPP(SMD = CH_2_Cl_2_)//M06-2X-D3/6-31+G(d,p);def2TZVP[Sc](CPCM = CH_2_Cl_2_) level of theory. (iv) Natural bonding orbital spin density plots for intermediates **B** and **E** visualized using an isovalue of 0.03. (v) Cyclization transition state structures (**TS-I**) under the triplet pathway and octahedral distortion parameters. (vi) Deuterium labeling crossover experiment.

To gain further insight into the reaction, we probed the mechanism and the determinants of selectivity and reactivity by experimental and computational means. M06-2X-D3 DFT calculations were used throughout these studies.^20^ Computational investigations suggest that scandium–Feng complex coordination to **1** reduces the adiabatic triplet energy from 57.7 kcal·mol^–1^ to 52.9 kcal·mol^–1^, therefore facilitating a modest change of 4.8 kcal·mol^–1^ (Figure 2(i)). Square-wave voltammetry (SWV) measurements performed on **1** revealed a substantial change in the reduction potential from −2.39 V to −1.08 V (vs SCE, in MeCN) upon addition of Sc(OTf)_3_ to the measurement cell. This observation raised the possibility of an alternative photoredox mechanism initiated by single-electron reduction of the substrate by the excited state photocatalyst. Comparing triplet energies and reduction potentials of Lewis acid complex **A** with the best-performing catalyst Ir((5-CF_3_)(4′-*t*-Bu)ppy)_3_, it is not possible to rule out energy transfer or electron transfer pathways. The best-performing photocatalysts (see Table 1) have triplet energies between that of the free and scandium-complexed substrate **1**, at around 55 kcal·mol^–1^. However, catalysts with triplet energies higher than 57.7 kcal·mol^–1^ display poor enantiocontrol. We were unable to identify any photocatalysts with a triplet energy lower than that of the substrate–Lewis acid complex that were capable of delivering the product in appreciable yield. These results align with the hypothesis that the best performing catalysts function by minimizing the undesired background energy transfer process to the unbound substrate. As part of our screening regime, we were able to identify a group of relatively weakly reducing benzothiazole-containing photocatalysts **25**–**27**,^21^ for which energy transfer is exergonic and electron transfer is endergonic (Figure 2(ii)). These catalysts all afford the product **2** in moderate to good enantioselectivity, with a correlation between higher triplet energy and higher conversion. Within this catalyst class, this is indicative that we are most likely observing an energy transfer regime.

We characterized the reactive potential energy surface for both possibilities using **1**:ScCl_3_ as a model system for the larger Feng ligand–scandium complex (Figure 2(iii)). We observed significant kinetic differences for ring closure in the two manifolds. For electron transfer, the radical anion has an activation barrier to cyclization of 29.0 kcal·mol^–1^ (**TS-II**), and this step is endergonic by 9.0 kcal·mol^–1^.^22^ This activation barrier is inconsistent with reactivity at 15 °C. In contrast, following energy transfer, ring-closure from intermediate ^**3**^**B** has a much lower barrier of 6.6 kcal·mol^–1^ (via **TS-I**) and is exergonic by 12.0 kcal·mol^–1^. This analysis of the two pathways indicates that energy transfer is the more likely mechanistic candidate responsible for catalysis. Electron transfer may contribute as an off-cycle process where the resulting radical anion is recycled to its neutral form by back electron transfer to the oxidized photocatalyst. This hypothesis could explain why highly reducing catalysts such as Ir((3′-OMe)ppy)_3_ (see SI p S8), although enantioselective, lead to poor conversion. Measurement of the quantum yield for the reaction of **1** under optimized conditions gave a value consistent with a non-chain process (ϕ = 0.003).

The marked difference in ring closure barriers between electron transfer and energy transfer pathways can be understood from the open-shell electronic structures of intermediates ^**3**^**B** and **E** (Figure 2(iv)). One of the unpaired electrons in ^**3**^**B** is highly localized at the β-carbon (natural spin density of 0.92), leading to an efficient radical addition into the aromatic ring. On the other hand, the unpaired electron in **F** is highly delocalized across the enolate motif. The spin density at the reactive center (0.43) is much lower in this pathway. Further inspection of intermediate ^**3**^**B** indicates deconjugation of the amide nitrogen from the phenyl ring. The radical cyclization step can be interpreted as an ambiphilic secondary radical attack into an electron-neutral aromatic system, thus constituting a good polarity match. However, in the case of the radical anion intermediate **E**, the amide nitrogen remains conjugated with the aromatic ring but is deconjugated from the carbonyl group, leading to a relatively electron-rich aniline system. It can be argued that the attack of the nucleophilic radical anion motif into the nucleophilic aniline rings system is therefore disfavored based on a polarity mismatch.

Under the assumption of a fast subsequent intersystem crossing process (ISC) the ring-closing (**TS-I**) step is enantiodetermining (see Supporting Information S79). The stabilities of competing diastereomeric cyclization TSs with the Sc–Feng Lewis acid complex (ΔΔ*G*^⧧^ = 1.3 kcal·mol^–1^) correspond to a predicted er of 91:9 at 15 °C (Figure 2(v)). This compares favorably to the absolute sense and magnitude observed experimentally (94:6 er). The origins of enantioselectivity result from geometric changes at the octahedrally coordinated Sc center. The metal adopts a distorted octahedral geometry to accommodate cyclization on the *Re*-face of **1** in the chiral cavity generated by the Feng ligand. By using the trigonal distortion parameter Θ (a measure of deviation from ideal octahedral geometry to that of trigonal prismatic)^23^ as a quantitative measurement, it was found that cyclization on the *Re*-face of the tetrahydropyran group requires a distortion of Θ = 440°, which is substantially more than that of the *Si*-face ring closure (Θ = 305°).

Following intersystem crossing from intermediate ^**3**^**C**, the resulting zwitterion **D** can undergo a 1,5-H-shift (**TS-III**) with a thermally accessible activation barrier of 15.1 kcal·mol^–1^. Dissociation of the Lewis acid complex leads to the experimentally observed cyclization product **2**. To support the proposed 1,5-H-shift, we conducted the reaction on pentadeuterated substrate **28** (Figure 2(vi)). Under the optimized reaction conditions, complete transfer of deuterium from the *ortho*-position of the benzene ring to the carbonyl α-position was observed in **30**, consistent with the proposed 1,5-shift. When a nondeuterated substrate **29** was included in a crossover experiment, we did not observe any evidence of deuterium transfer between reactants, thus ruling out intermolecular proton transfer as a significant alternative mechanism for hydrogen migration (see SI p 65 for details).

## Conclusion

In conclusion, we have developed a 6π photocyclization that operates under Lewis acid-mediated triplet energy transfer to enable a catalytic and highly enantioselective example of this reaction class. The Lewis acid mediates a modest reduction in substrate triplet energy that offers a narrow window in which enantioselective catalysis can outcompete a background reaction. Thus, the yield and enantioselectivity of the process are heavily dependent on the photocatalyst employed, with the optimal sensitizer being developed through structural modifications that minimize background energy transfer to the unbound substrate. We believe this study will facilitate new 6π photocyclizations and inform the development of subsequent Lewis acid-mediated enantioselective triplet sensitized reactions.

## Supporting Information Available

Synthetic procedures, spectroscopic characterization data, HPLC data, photocatalyst characterization, computational methods, and associated data (PDF)
