# Supplementary material for: Catalytic Enantioselective 6π Photocyclization of Acrylanilides
Source: J Am Chem Soc. 2022 Dec 26;145(1):171–8. doi: 10.1021/jacs.2c09267 (PMC9837842; doi:10.1021/jacs.2c09267)
Supplement: Supplementary file 1 — ja2c09267_si_001.pdf [file ja2c09267_si_001.pdf]

Supporting Information for

Catalytic enantioselective 6π photocyclization of acrylanilides

Benjamin A. Jones[1], Pearse Solon[1], Mihai V. Popescu[1,2], Ji-Yuan Du[1], Robert Paton[2]* & Martin D. Smith[1]*

[1] Chemistry Research Laboratory, University of Oxford, 12 Mansfield Road, Oxford, OX1 3TA, UK.
[2] Department of Chemistry, Colorado State University, 1301 Center Ave, Ft. Collins, CO 80523-1872, USA.

*To whom correspondence should be addressed: martin.smith@chem.ox.ac.uk robert.paton@colostate.edu

Contents

1. General Information 4
2. Reaction Optimization 6
 2.1. Full Optimization Table 6
 2.2. Summary of Enantioselective Reaction Optimization 8
 2.3. Chiral Ligands Employed in this Study 9
 2.4. Photocatalysts Employed in this Study 10
3. Experimental Procedures 11
 3.1. General Procedures 11
 3.2. Photocatalysts and Phenylpyridines 13
 3.3. Precursors 20
 3.4. Substrates 25
 3.5. Photocyclization Products 38
 3.6. Unsuccessful Substrates 58
4. Characterisation of Novel Photocatalysts 59
 4.1. Absorbance and Emission Spectra of Novel Photocatalysts 59
 4.2. Square-Wave Voltammograms of Novel Photocatalysts 63
 4.3. Estimation of Excited State Redox Potentials for Novel Photocatalysts 67
 4.4. Summary of Photocatalyst Properties 67
5. Mechanistic Experiments and Photophysics 68
 5.1. Labelling and Crossover Experiments 68
 5.2. Chemical Actinometry and Quantum Yield Measurement 74
 5.3. Weakly Reducing Photocatalyst Study 75
 5.4. Electrochemical Measurements 76
 5.5. Stern-Volmer Quenching 77
 5.6. UV-Vis Absorbance Spectra 81
6. X-ray Crystallography 82
7. Computational Details 85
 7.1. Methods 85
 7.2. Model $ScCl_3$ Energy Transfer Potential Energy Surface 86
 7.3. Model $ScCl_3$ Redox Neutral Photocyclization Potential Energy Surface 88
 7.4. Potential Energy Surface Including Thermochemistry of the Photocatalyst 89
 7.5. 3D Structures of $ScCl_3$ Model Stationary Points 90
 7.6. Computational Studies of the Chiral Ligand-Containing System 91

7.7. Population Analysis 92
7.8. Thermochemical Data 93
7.9. XYZ Coordinates for Computed Structures 101
8. HPLC Traces for Chiral Compounds 133
8.1. HPLC Traces for Cyclisation of S3 164
9. NMR Spectra for Compounds 166
9.1. Photocatalysts and Phenylpyridines 166
9.2. Precursors 178
9.3. Substrates 187
9.4. Photocyclization Products 214
10. References 247

1. General Information

Naming and Numbering

Systematic compound names in accordance with regulations set forth by the International Union for Pure and Applied Chemistry (IUPAC) have been generated from the appropriate structures using the PerkinElmer Informatics software ChemDraw® (version 21).

Reaction Conditions

Reactions requiring moisture-sensitive reagents performed in flame-dried glassware, under an atmosphere of argon (balloon pressure). Room temperature refers to 20–25 °C. Temperatures of 0 °C were obtained using an ice/water bath. Temperatures of −78 °C were obtained using a dry ice/acetone bath. Temperatures of 0 °C to −40 °C were obtained using a polar bear plus. 10-15 °C refers to reactions carried out in a tempering beaker filled with water. Reflux and heating conditions were obtained using a Drysyn® heating block or an oil bath equipped with a thermometer where the reported temperature refers to the temperature of the Drysyn® block or oil bath. Syringe filters, where used, were 0.2 µm PTFE membrane filters obtained from Fisher Scientific (cat no. 15141499).

Solvents and Reagents

Anhydrous dichloromethane and tetrahydrofuran were obtained using an MBraun SPS-5. All other solvents were used without further purification. Degassed solvents were prepared by sparging with argon for 10 minutes. Reagents were used directly as supplied by major chemical suppliers.

Chromatography

Thin layer chromatography was carried out on Merck Kieselgel 60, F_{254} 0.25 mm precoated aluminium plates and visualisation was achieved by UV light (λ_{max} = 254 nm) and/or by staining with potassium permanganate solution or phosphomolybdic acid solution. Column chromatography was performed using VWR silica gel 60 (40-63 m particle size) or Aldrich silca gel 60 (40-63 nm particle size) using pressure by means of a nitrogen line.

NMR Spectroscopy

NMR spectra were recorded on a Bruker Avance spectrometers at room temperature (unless otherwise stated) in the deuterated solvent stated and are referenced to the residual non-deuterated solvent peak. Chemical shifts are quoted in ppm with signal splittings recorded as singlet (s), doublet (d), triplet (t), quartet (q), hept. (heptet), and multiplet (m). The abbreviation br. denotes broad. Coupling constants, *J*, are measured to the nearest 0.1 Hz and are presented as observed. All ^{19}F NMR spectra are proton decoupled.

Infrared

Infrared spectra were recorded on a Bruker Tensor 27 FTIR spectrometer equipped with a diamond ATR module. Absorption maxima (ν_{max}) are reported in wavenumbers (cm^{-1}).

Mass Spectrometry

Accurate masses (HRMS) were recorded on Bruker MicroTOF and Micromass GCT spectrometers under conditions of electrospray ionisation (ESI).

Polarimetry

Optical Rotations were determined using a Schmidt + Haensch Unipol 2020 polarimeter with a path length of 1 dm (using the sodium D line, 589 nm). Concentrations are reported in g/100 mL. Temperatures are reported in °C.

Chiral HPLC

Chiral HPLC was performed on a Dionex Ultimate 3000 system comprising of a Dionex LPG-3400SD pump, WPS-3000SL autosampler, TCC-3000SD column compartment fitted with the appropriate Daicel Chiralpak column (dimensions: 0.46 cm ø × 25 cm) and corresponding guard column (0.4 cm ø × 1 cm), and a DAD-3000 diode array detector. Wavelengths (λ) are reported in nm, retention times (τ_R) are reported in minutes and solvent flow rates are reported in mL min^{-1}.

LC/MS

LC/MS was carried out on an Agilent 1260 Infinity II system comprising of a 1260 Infinity II Quaternary Pump VL (G7111A), a 1260 Infinity II Vialsampler (G7129A) with integrated column compartment (G7130A), a Agilent 1260 Infinity II Diode Array Detector WR (G7115A), and a 6100 Series Single Quadrupole LC/MS (G7115A) using API-ES positive ionisation. Separations were carried out by gradient elution (1 minute dwell at 5% MeCN/H_2O, followed by a gradient to 95% MeCN/H_2O over 7 minutes) using a Poroshell 120 EC-C18 USP L1 Solvent Saver 3.0 x 100mm column at a flow rate of 0.4 mL min^{-1}. 0.1% formic acid was added to all solvents.

Melting Points

Melting points were determined using a Reichert melting point apparatus and are uncorrected.

Synthesis of Catalysts and Ligands

The chiral ligands and photocatalysts that were not commercially available were prepared following reported literature methods.[1–3]

Voltammetry Studies

Voltammetry measurements were made using an EmStat3 with a glassy carbon electrode as the working electrode, a platinum wire as counter electrode and a leak free Ag/AgCl (sat. KCl) reference electrode. Voltammograms were referenced to the Fc/Fc^+ couple as an internal reference. Square wave voltammograms were acquired with a 5 mV step potential, 50 mV modulation amplitude and 5 Hz frequency. The supporting electrolyte, (tetra-n-butylammonium hexafluorophosphate, Bu_4NPF_6, $TBAPF_6$) was prepared as a 0.1 M solution in either MeCN or CH_2Cl_2. Samples were measured at 0.01 M concentration.

Fluorescence spectroscopy

Emission spectra were recorded on an Edinburgh Instruments FS5 spectrometer. Emission spectra were recorded as 5 µM solutions in degassed MeCN by irradiating at 390 nm and recording between 400 and 800 nm.

2. Reaction Optimization

2.1. Full Optimization Table

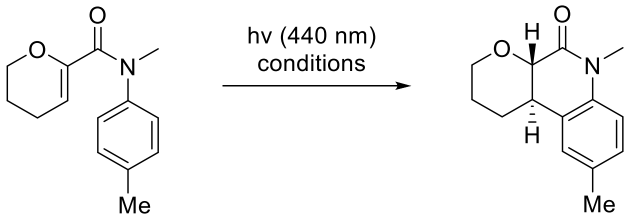

Entry	Photocatalyst	Lewis acid (mol %)	Ligand (mol %)	Solvent/conc. (M)	Temp. (°C)	Conversion (%)[a]	Yield (%)[a]	er[c]	dr[d]
**1**	$Ir(ppy)_3$	$Sc(OTf)_3$ (25)	**L1** (30)	CH_2Cl_2 (0.02)	10-15[b]	11	10	-	1:1
**2**	$Ir(ppy)_3$	$Sc(OTf)_3$ (25)	**L2** (30)	CH_2Cl_2 (0.02)	10-15[b]	9	10	50:50	-
**3**	$Ir(ppy)_3$	$Sc(OTf)_3$ (25)	**L3** (30)	CH_2Cl_2 (0.02)	10-15[b]	<10	5	70:30	-
**4**	$Ir(ppy)_3$	$Sc(OTf)_3$ (25)	**3** (30)	CH_2Cl_2 (0.02)	10-15[b]	100	96	75:25	>20:1
**5**	$Ir(ppy)_3$	$Sc(OTf)_3$ (25)	**3** (30)	DCE (0.02)	10-15[b]	59	40	67:33	>20:1
**6**	$Ir(ppy)_3$	$Sc(OTf)_3$ (25)	**3** (30)	PhMe (0.02)	10-15[b]	37	17	64:35	>20:1
**7**	$Ir(ppy)_3$	$Sc(OTf)_3$ (25)	**3** (30)	CPME (0.02)	10-15[b]	55	64	-	3:1
**8**	$Ir(ppy)_3$	$Sc(OTf)_3$ (25)	**3** (30)	EtOAc (0.02)	10-15[b]	79	<10	-	-
**9**	$Ir(ppy)_3$	$Sc(OTf)_3$ (25)	**L4** (30)	CH_2Cl_2 (0.02)	10-15[b]	n.d.	n.d.	51:49	n.d.
**10**	$Ir(ppy)_3$	$Sc(OTf)_3$ (25)	**L5** (30)	CH_2Cl_2 (0.02)	10-15[b]	n.d.	n.d.	51:49	n.d.
**11**	$Ir(ppy)_3$	$Sc(OTf)_3$ (25)	**L6** (30)	CH_2Cl_2 (0.02)	10-15[b]	n.d.	n.d.	70:30	n.d.
**12**	$Ir(ppy)_2(dtbbpy)PF_6$	$Sc(OTf)_3$ (25)	**L7** (30)	CH_2Cl_2 (0.02)	10-15[b]	<5	<5	55:45	-
**13**	$Ir(Fppy)_2(dtbbpy)PF_6$	$Sc(OTf)_3$ (25)	**L6** (30)	CH_2Cl_2 (0.02)	10-15[b]	17	12	56:44	>20:1
**14**	$Ir(dFppy)_2(dtbbpy)PF_6$	$Sc(OTf)_3$ (25)	**L6** (30)	CH_2Cl_2 (0.02)	10-15[b]	100	80	51:49	3:1
**15**	$Ir(ppy)_3$	$Cu(OTf)_2$ (25)	**3** (30)	CH_2Cl_2 (0.02)	10-15[b]	65	<5	63:37	-
**16**	$Ir(ppy)_3$	$Mg(OTf)_2$ (25)	**3** (30)	CH_2Cl_2 (0.02)	10-15[b]	84	40	50:50	-
**17**	$Ir(ppy)_3$	$La(OTf)_3$ (25)	**3** (30)	CH_2Cl_2 (0.02)	10-15[b]	84	50	50:50	4:1
**18**	$Ir(ppy)_3$	$Sc(OTf)_3$ (10)	**3** (12)	CH_2Cl_2 (0.02)	10-15[b]	100	80	50:50	7:1
**19**	$Ir(ppy)_3$	$Sc(OTf)_3$ (25)	**3** (30)	CH_2Cl_2 (0.02)	10-15[b]	79	73	75:25	10:1
**20**	$Ir(ppy)_3$	$Sc(OTf)_3$ (50)	**3** (60)	CH_2Cl_2 (0.02)	10-15[b]	-	-	64:36	-
**21**	$Ir(ppy)_3$	$Sc(OTf)_3$ (25)	**3** (30)	CH_2Cl_2 (0.02)	-30	82	48	42:58	3:1
**22**	$Ir(ppy)_3$	$Sc(OTf)_3$ (25)	**3** (30)	CH_2Cl_2 (0.01)	10-15[b]	79	71	75:25	>20:1
**23**	$Ir(ppy)_3$	$Sc(OTf)_3$ (25)	**3** (30)	CH_2Cl_2 (0.05)	10-15[b]	90	62	54:46	5:1
**24**	$Ir(ppy)_3$	none	none	CH_2Cl_2 (0.02)	10-15[b]	83	6	-	>20:1
**25**	none	$Sc(OTf)_3$ (25)	none	CH_2Cl_2 (0.02)	10-15[b]	100	<5	-	-
**26**	$Ir(dFppy)_3$	$Sc(OTf)_3$ (25)	**3** (30)	CH_2Cl_2 (0.02)	10-15[b]	100	95	53:47	>20:1
**27**	$Ir(ppy)_3$	$Sc(OTf)_3$ (25)	**L7** (30)	CH_2Cl_2 (0.02)	10-15[b]	79	70	54:46	5:1
**28**	$Ir(ppy)_3$	$Sc(OTf)_3$ (25)	**L8** (30)	CH_2Cl_2 (0.02)	10-15[b]	89	70	50:50	4:1

**29**	Ir((3'-OMe)ppy))$_3$	Sc(OTf)$_3$ (25)	**3** (30)	CH_2Cl_2 (0.02)	10-15[b]	48	13	86:14	>20:1
**30**	Ir(dF(CF$_3$)ppy)$_2$(bpy)(PF$_6$)	Sc(OTf)$_3$ (25)	**3** (30)	CH_2Cl_2 (0.02)	10-15[b]	100	61	51:49	5:1
**31**	Ir(Fppy)$_3$	Sc(OTf)$_3$ (25)	**3** (30)	CH_2Cl_2 (0.02)	10-15[b]	100	67	54:46	1:.1
**32**	Ir((3'-OMe)ppy))$_3$	Sc(OTf)$_3$ (25)	**3** (30)	MeCN (0.02)	10-15[b]	30	<5	70:30	-
**33**	Ir((3'-OMe)ppy))$_3$	Sc(OTf)$_3$ (25)	**L9** (30)	CH_2Cl_2 (0.02)	10-15[b]	47	32	84:16	5:1
**34**	Ir((3'-OMe)ppy))$_3$	Gd(OTf)$_3$ (25)	**L9** (30)	CH_2Cl_2 (0.02)	10-15[b]	<5	<5	-	-
**35**	Ir((3'-OMe)ppy))$_3$	Y(OTf)$_3$ (25)	**L9** (30)	CH_2Cl_2 (0.02)	10-15[b]	<5	<5	-	-
**36**	Ir((3'-OMe)ppy))$_3$	Yb(OTf)$_3$ (25)	**L9** (30)	CH_2Cl_2 (0.02)	10-15[b]	<5	<5	-	-
**37**	Ir((3'-OMe)ppy))$_3$	Eu(OTf)$_3$	**L9** (30)	CH_2Cl_2 (0.02)	10-15[b]	<5	<5	-	-
**38**	Ir((3'-OMe)ppy))$_3$	Zn(OTf)$_3$	**L9** (30)	CH_2Cl_2 (0.02)	10-15[b]	<5	<5	-	-
**39**	Ir((5-F)ppy)$_3$ (**C1**)	Sc(OTf)$_3$ (25)	**L9** (30)	CH_2Cl_2 (0.02)	10-15[b]	100	98	84:16	>20:1
**40**	Ir((5-F)ppy)$_3$ (**C1**)	Sc(OTf)$_3$ (25)	**L9** (30)	CH_2Cl_2 (0.02)	0	100	91	86:14	>20:1
**41**	Ir((5-F)ppy)$_3$ (**C1**)	Sc(OTf)$_3$ (25)	**L9** (30)	CH_2Cl_2 (0.02)	−30	100	92	88:12	>20:1
**42**	Ir((5-F)ppy)$_3$ (**C1**)	Sc(OTf)$_3$ (25)	**L9** (30)	CH_2Cl_2 (0.02)	−50	<5	<5	-	-
**43**	Ir((5-F)ppy)$_3$ (**C1**)	Sc(OTf)$_3$ (25)	**L9** (30)	CH_2Cl_2 (0.02)	−30	100	82	88:12	>20:1
**44**	Ir((5-F)ppy)$_3$ (**C1**)	Sc(OTf)$_3$ (25)	**3** (30)	CH_2Cl_2 (0.02)	−30	15	12	72:28	-
**45**	Ir((5-F)ppy)$_3$ (**C1**)	Sc(OTf)$_3$ (25)	**L10** (30)	CH_2Cl_2 (0.02)	−30	29	<5	80:20	-
**46**	Ir((5-F)ppy)$_3$ (**C1**)	Sc(OTf)$_3$ (25)	**L10** (30)	CH_2Cl_2 (0.02)	−20	88	80	88:12	>20:1
**47**	Ir((5-F)ppy)$_3$ (**C1**)	Sc(OTf)$_3$ (25)	**3** (30)	CH_2Cl_2 (0.02)	−20	72	56	88:12	>20:1
**48**	Ir((5-F)ppy)$_3$ (**C1**)	Sc(OTf)$_3$ (25)	**L10** (30)	CH_2Cl_2 (0.02)	−20	<5	<5	87:13	-
**49**	Ir((5-F, 4'-^{t}Bu)ppy)$_3$ (**C2**)	Sc(OTf)$_3$ (25)	**L10** (30)	CH_2Cl_2 (0.02)	−30	<5	<5	80:20	-
**50**	Ir((5-F, 4'-^{t}Bu)ppy)$_3$ (**C2**)	Sc(OTf)$_3$ (25)	**L10** (30)	CH_2Cl_2 (0.02)	10-15[b]	100	65	89:11	>20:1
**51**	Ir((5-F)ppy)$_3$ (**C1**)	Sc(OTf)$_3$ (25)	**L10** (27.5)	CH_2Cl_2 (0.02)	−30	100	90	89:11	>20:1
**52**	Ir((5-F)ppy)$_3$ (**C1**)	Sc(OTf)$_3$ (20)	**L10** (22)	CH_2Cl_2 (0.02)	−30	85	70	89:11	>20:1
**53**	Ir((5-F)ppy)$_3$ (**C1**)	Sc(OTf)$_3$ (15)	**L10**(16.5)	CH_2Cl_2 (0.02)	−30	74	57	89:11	>20:1
**54**	Ir((5-F)ppy)$_3$ (**C1**)	Sc(OTf)$_3$ (10)	**L10** (12)	CH_2Cl_2 (0.02)	−30	69	46	86:14	>20:1
**55**	Ir((5-F)ppy)$_3$ (**C1**)	Sc(OTf)$_3$ (25)	**3** (30)	CH_2Cl_2 (0.02)	10-15[b]	88	59	86:14	>20:1
**56**	Ir((5-F)ppy)$_3$ (**C1**)	Sc(OTf)$_3$ (25)	**L10** (30)	CH_2Cl_2 (0.02)	10-15[b]	90	90	84:16	>20:1
**57**	Ir((5-F)ppy)$_3$ (**C1**)	Sc(OTf)$_3$ (10)	**L10** (12)	CH_2Cl_2 (0.05)	10-15[b]	88	77	84:16	>20:1
**58**	Ir((5-F, 4'-^{t}Bu)ppy)$_3$ (**C2**)	Sc(OTf)$_3$ (15)	**3** (16)	CH_2Cl_2 (0.02)	10-15[b]	85	65	88:12	>20:1
**59**	Ir((5-F, 4'-^{t}Bu)ppy)$_3$ (**C2**)	Sc(OTf)$_3$ (15)	**3** (18)	CH_2Cl_2 (0.02)	−10	77	61	90:10	>20:1
**60**	Ir((5-F, 4'-^{t}Bu)ppy)$_3$ (**C2**)	Sc(OTf)$_3$ (25)	**3** (27)	CH_2Cl_2 (0.02)	−10	88	74	89:11	>20:1
**61**	Ir((5-F, 4'-^{t}Bu)ppy)$_3$ (**C2**)	Sc(OTf)$_3$ (25)	**L10** (27)	CH_2Cl_2 (0.02)	−10	87	84	89:11	>20:1
**62**	Ir((5-CF$_3$)ppy)$_3$ (**C3**)	Sc(OTf)$_3$ (25)	**L10** (27)	CH_2Cl_2 (0.02)	10-15[b]	55	45	89:11	>20:1
**63**	Ir((5-CF$_3$, 4'-^{t}Bu)ppy)$_3$ (**4**)	Sc(OTf)$_3$ (25)	**3** (27)	CH_2Cl_2 (0.02)	10-15[b]	100	95	94:6	>20:1
**64**	Ir((5-CF$_3$, 4'-^{t}Bu)ppy)$_3$ (**4**)	Sc(OTf)$_3$ (25)	**3** (25)	CH_2Cl_2 (0.02)	10-15[b]	100	95	95:5	>20:1
**65**	Ru(deeb)$_3$(PF6)$_2$	Sc(OTf)$_3$ (25)	**3** (25)	CH_2Cl_2 (0.02)	10-15[b]	0	0	-	-
**66**	Ir(phbt)$_2$acac	Sc(OTf)$_3$ (25)	**3** (25)	CH_2Cl_2 (0.02)	10-15[b]	31	15	79:21	>20:1
**67**	Ir((4-F)phbt)$_2$acac	Sc(OTf)$_3$ (25)	**3** (25)	CH_2Cl_2 (0.02)	10-15[b]	54	33	76:24	>20:1

**68**	Ir((4-CF3)phbt)$_2$acac	Sc(OTf)$_3$ (25)	**3** (25)	CH_2Cl_2 (0.02)	10-15[b]	22	8	88:12	>20:1
**69**	BODIPY 1	Sc(OTf)$_3$ (25)	**3** (25)	CH_2Cl_2 (0.02)	10-15[b]	0	0	-	-
**70**	BODIPY 2	Sc(OTf)$_3$ (25)	**3** (25)	CH_2Cl_2 (0.02)	10-15[b]	0	0	-	-
**71**	BODIPY 3	Sc(OTf)$_3$ (25)	**3** (25)	CH_2Cl_2 (0.02)	10-15[b]	0	0	-	-

Table S1: Reaction optimization. a) Determined by quantitative ^{1}H NMR spectroscopy using CH_2Br_2 as an internal standard. b) 10-15 °C refers to the internal temperature of a water-cooled water filled jacketed beaker in which the reaction vial was immersed. c) Determined by ^{1}H NMR analysis of the crude reaction mixture. d) Determined by chiral stationary phase HPLC.

2.2. Summary of Enantioselective Reaction Optimization

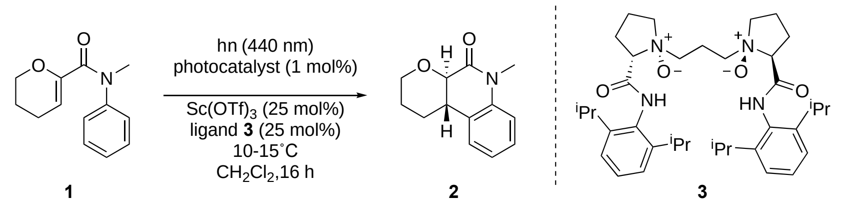

Entry	Photocatalyst	E_T (kcal mol^{-1})	$E_{1/2}^{red}$ Ir$^{IV/*III}$ (V)	Lewis acid /ligand	Yield (%)[a]	er[b]	dr[c]
**1**	Ir(dFppy)$_3$	63.8	-1.83	none	85	50:50	>20:1
**2**	Ir(dFppy)$_3$	63.8	-1.83	Sc(OTf)$_3$/**3**	95	53:47	>20:1
**3**	Ir(ppy)$_2$(dtbbpy)(PF$_6$)	56.4	-1.19	Sc(OTf)$_3$/**3**	<5	68:32	-
**4**	Ir(Fppy)$_2$(dtbbpy)(PF$_6$)	61.0	-1.19	Sc(OTf)$_3$/**3**	20	62:38	>20:1
**5**	Ir(dFppy)$_2$(dtbbpy)(PF$_6$)	63.3	-1.15	Sc(OTf)$_3$/**3**	70	58:42	14:1
**6**	Ir(dF(CF$_3$)ppy)$_2$(dtbbpy)(PF$_6$)	63.3	-1.01	Sc(OTf)$_3$/**3**	97	52:48	18:1
**7**	Ir(ppy)$_3$	59.4	-1.81	Sc(OTf)$_3$/**3**	67	75:25	>20:1
**8**	Ir((3'-OMe)ppy)$_3$	55.3	-1.89	Sc(OTf)$_3$/**3**	26	79:21	>20:1
**9**	Ir((5-F)ppy)$_3$	59.2	-1.74	Sc(OTf)$_3$/**3**	84	86:14	>20:1
**10**	Ir((5-F)(4'-*t*-Bu)ppy)$_3$	59.0	-1.80	Sc(OTf)$_3$/**3**	84	84:16	>20:1
**11**	Ir((5-CF$_3$)ppy)$_3$	56.0	-1.50	Sc(OTf)$_3$/**3**	92	91:9	>20:1
**12**	Ir((5-CF$_3$)(4'-*t*-Bu)ppy)$_3$	55.7	-1.55	Sc(OTf)$_3$/**3**	95	95:5	>20:1
**13**	Ir((5-CF$_3$)(4'-*t*-Bu)ppy)$_3$	55.7	-1.55	none	-	-	-

Table S2: Summary of reaction optimization. a) Determined by quantitative ^{1}H NMR spectroscopy using 1,3,5-trimethoxybenzene as an internal standard. b) Determined by chiral stationary phase HPLC. c) Determined by ^{1}H NMR analysis of the crude reaction mixture. 10-15 °C refers to the internal temperature of a water-cooled water filled jacketed beaker in which the reaction vial was immersed

2.3. Chiral Ligands Employed in this Study

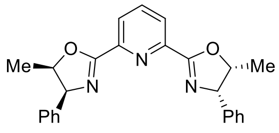

L1

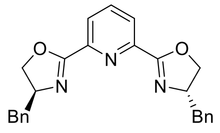

L2

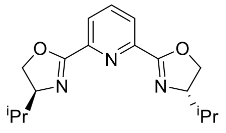

L3

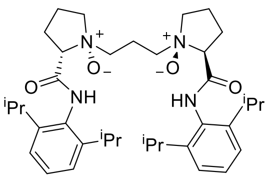

3

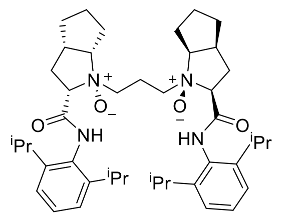

L4

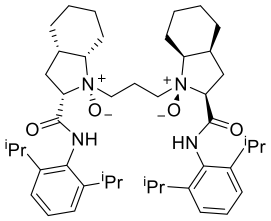

L5

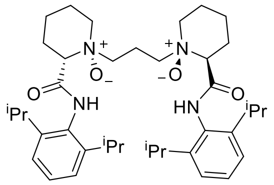

L6

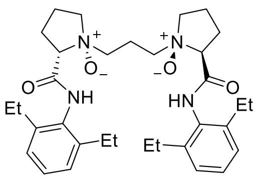

L7

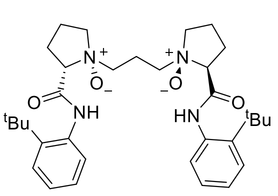

L8

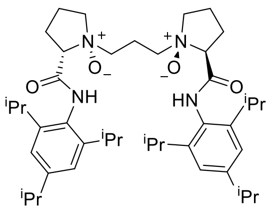

L9

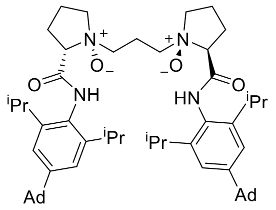

L10

Figure S1: Chiral ligands employed in this study.

2.4. Photocatalysts Employed in this Study

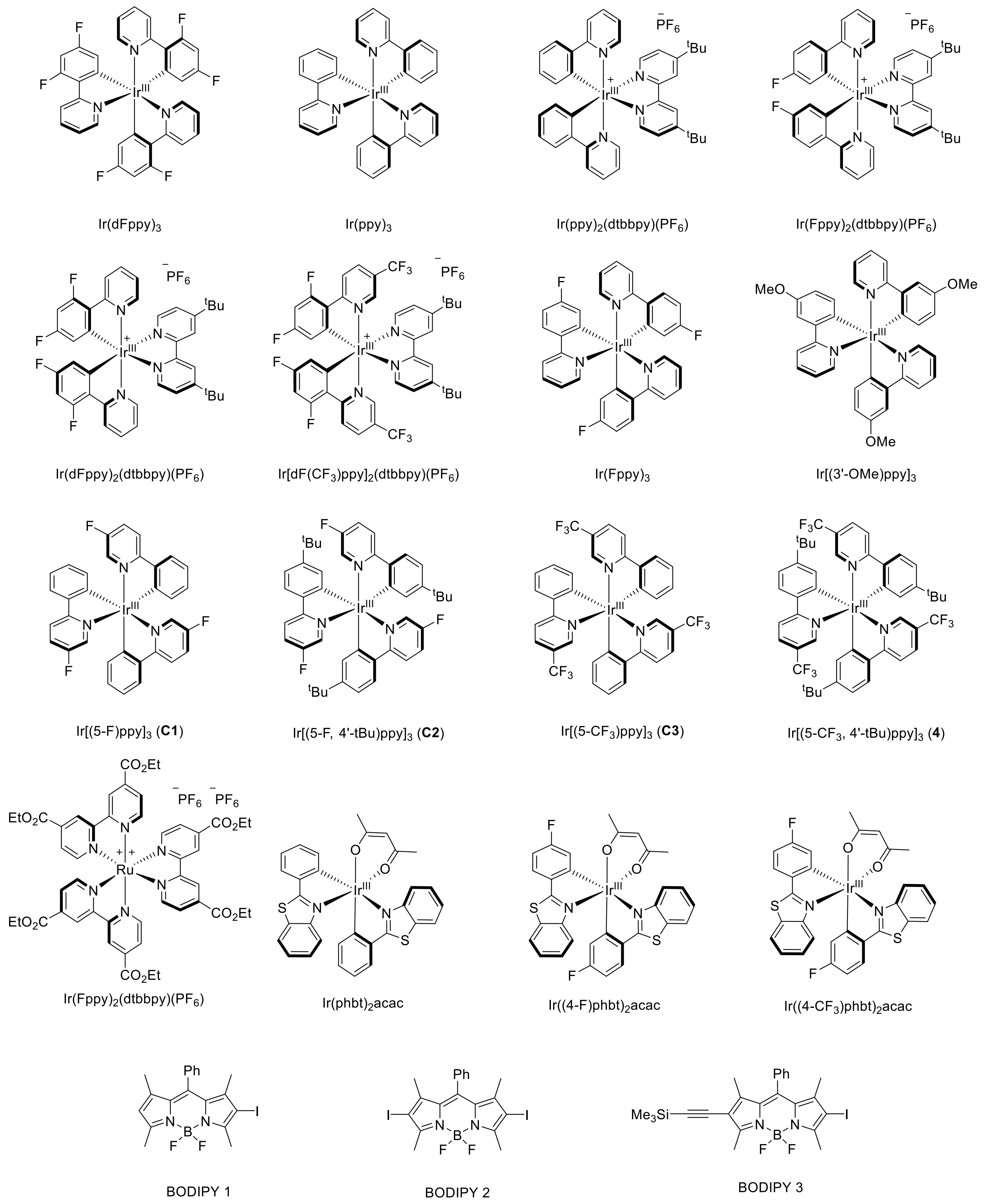

Figure S2: Photocatalysts employed in this study.

3. Experimental Procedures

3.1. General Procedures

General Procedure A: Preparation of alkylated anilines

To *the appropriate aniline* in anhydrous THF (0.2 M) at 0 °C was added ^{n}BuLi (2.6 M, 1.1 equiv.) dropwise. After a further 30 min, the appropriate alkyl halide (1.0 equiv.) was added and the reaction left to stir at room temperature for 16 hours. Upon completion, the reaction mixture was quenched by slow addition of H_2O, diluted with CH_2Cl_2, washed with sat.aq. $NaHCO_3$ (3 x), dried over $MgSO_4$, filtered, and concentrated *in vacuo*. The crude product was purified by flash column chromatography afford the corresponding *N*-alkylanilines.

General Procedure B: Preparation of photocyclization substrates by amide coupling

The appropriate carboxylic acid (1.00 equiv.) was dissolved in CH_2Cl_2 (0.5 M) and cooled to 0 °C and Et_3N (1.10 equiv.) followed by isobutyl chloroformate (1.00 equiv.) were added. After 30 minutes at 0 °C, *the appropriate* N-*alkyl aniline* (1.10 equiv.) was added and the reaction was warmed to room temperature and stirring was continued for 16 hours. Water was added and the phases were separated, the aqueous phase was extracted twice with CH_2Cl_2 and the combined organic layers were dried over anhydrous Na_2SO_4, filtered and concentrated to give crude product, which was purified by flash column chromatography to afford the corresponding acrylanilides.

General Procedure C: Enantioselective photocyclization

$Sc(OTf)_3$ (51.7 mg, 0.105 mmol) and **3** (65.1 mg, 0.105 mmol) were added to a 25 mL vial equipped with a stirrer bar. The vial was capped then evacuated and backfilled with argon three times. CH_2Cl_2 (21 mL) which had been sparged with argon for 15 minutes was added by syringe and the vial was heated to 60 °C with sonication for 30 minutes then cooled to room temperature to give a stock solution of the Lewis acid complex.

$Ir((5\text{-}CF_3, 4'\text{-}t\text{-}Bu)ppy)_3$ (**4**) (1.0 mg, 1 µmol, 1 mol%) and *the appropriate starting material* (0.100 mmol) were added to a 10 mL vial equipped with a stirrer bar. The vial was capped with a 20 mm aluminium crimp cap with silicone/PTFE septum then evacuated and backfilled with argon three times. The Lewis acid complex solution was drawn into a 24 mL syringe and, under a protective layer of argon, the syringe was equipped with a syringe filter. 5 mL of the Lewis acid complex solution (0.025 equiv.) was dispensed into each reaction vial. The reaction vials were placed in a water jacketed beaker and stirred for 30 min in the dark before being irradiated with a Kessil lamp centred at 440 nm for 16 h. The reaction was transferred to a separating funnel using an additional 5 mL CH_2Cl_2 and washed with 1 M aqueous sodium hydroxide solution (10 mL). The aqueous phase was extracted twice with CH_2Cl_2 (10 mL) and the combined organic layers were dried over anhydrous Na_2SO_4, filtered and concentrated to give the crude product. The diastereomeric ratio was determined by quantitative ^{1}H NMR analysis of the crude reaction mixture. The crude product was purified by column chromatography (EtOAc-pentane) to give the pure cyclization product.

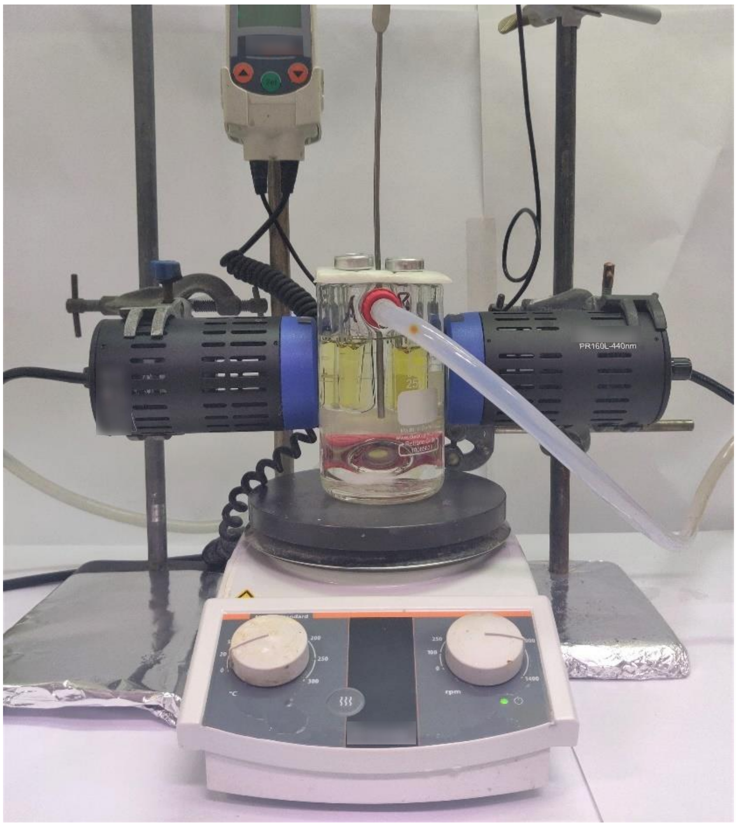

Experimental setup for General Procedure C showing 250 mL water jacketed beaker with reaction vials suspended in stirred water. Temperature is continuously monitored by an external probe (10 – 15°C) . Kessil PR160L-440 nm lamps are positioned touching the edge of the jacketed vessel.

General Procedure D1: Racemic photocyclization

Ir(dFppy)$_3$ (0.8 mg, 1 µmol, 1 mol%) and *the appropriate starting material* (0.100 mmol) were added to a 10 mL microwave vial. The vial was capped then evacuated and backfilled with argon three times. THF (2 mL) which had been sparged with argon for 15 minutes was added by syringe and the reaction was placed in a water jacketed beaker filled with water and irradiated with a kessil lamp centred at 440 nm for 16 h. After this time, the reaction mixture was concentrated to dryness to give the crude product. ^{1}H NMR analysis of the crude reaction mixture facilitated identification of the minor diastereomer peaks for determination of the dr for the enantioselective reactions. A small sample of the crude product was purified by preparative TLC to give the racemic reference material for chiral HPLC analysis.

General Procedure D2: Racemic photocyclization for *meta*-substituted substrates

Ir((5-CF$_3$, 4'-*t*-Bu)ppy)$_3$ (**4**) (1.0 mg, 1 µmol, 1 mol%), Sc(OTf)$_3$ (12.3 mg, 0.025 mmol, 0.25 equiv.), and *the appropriate starting material* were added to a 10 mL vial equipped with a stirrer bar. The vial was sealed and evacuated and backfilled with argon three times before CH_2Cl_2 (5.0 mL) sparged with argon for 15 min was added. The reaction was then irradiated with a Kessil lamp centred at 440 nm in a water jacketed beaker filled with water for 16 h. After this time, the reaction mixture was concentrated to dryness to afford the crude product. ^{1}H NMR analysis of the crude reaction mixture facilitated identification of the minor diastereomer and regioisomer peaks for determination of the diastereoisomeric ration (d.r.) and regioisomeric ratio (r.r.) for the enantioselective reactions. A small sample of the crude product was purified by preparative TLC to give the racemic reference material for chiral HPLC analysis.

3.2. Photocatalysts and Phenylpyridines

5-Fluoro-2-phenylpyridine (PP1)

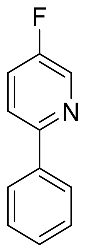

This compound was synthesized by a modification of a literature procedure.[1]

2-Bromo-5-fluoropyridine (440 mg, 2.50 mmol, 1.00 equiv.), phenylboronic acid (266 mg, 3.00 mmol, 1.20 equiv.), Pd(OAc)$_2$ (14.0 mg, 0.0625 mmol, 0.0250 equiv.), PPh$_3$ (65.5 mg, 0.0250 mmol, 0.100 equiv.) and K_2CO_3 (931 mg, 6.75 mmol, 2.70 equiv.) were dissolved in dimethoxyethane (2.5 ml) and water (3.3 mL). The resulting mixture was sparged with argon for 15 minutes then heated under reflux for 16 hours. Upon cooling to room temperature, the reaction mixture was transferred to a separating funnel, diluted with water and extracted three times with EtOAc. The combined organic layers were washed with brine, dried over anhydrous sodium sulfate, filtered and concentrated to give the crude product which was purified by flash column chromatography (1:19, CH_2Cl_2: pentane) to give 5-fluoro-2-phenylpyridine (**PP1**) (303 mg, 70%) as a white solid.

m.p. = 38-40 °C (CH_2Cl_2/pentane).

IR (film) ν_{max}/cm^{-1}: 2980, 1465, 1445, 1376, 1221, 1185, 1123, 1010, 833, 773, 727, 686.

^{1}H NMR (400 MHz, $CDCl_3$) δ = 8.55 (d, *J* = 2.9 Hz, 1H), 7.98–7.88 (m, 2H), 7.72 (ddd, *J* = 8.8, 4.3, 0.6 Hz, 1H), 7.55–7.39 (m, 4H).

^{13}C NMR (101 MHz, $CDCl_3$) δ = 159.0 (d, *J* = 256.1 Hz), 153.9 (d, *J* = 3.7 Hz), 138.6, 137.9 (d, *J* = 23.6 Hz), 129.0, 128.9, 126.9, 123.6 (d, *J* = 18.4 Hz), 121.4 (d, *J* = 4.3 Hz).

^{19}F NMR (377 MHz, $CDCl_3$) δ = −129.9.

HRMS (ESI$^+$) $C_{11}H_8FN$ [M+H]$^+$ requires 174.0714; found 174.0714, Δ 0.2 ppm.

2-(4-(*Tert*-butyl)phenyl)-5-fluoropyridine (PP2)

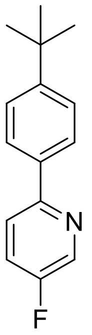

This compound was synthesized by a modification of a literature procedure.[1]

2-Bromo-5-fluoropyridine (880 mg, 5.00 mmol, 1.00 equiv.), 4-tert-butylphenylboronic acid (1.07 g, 6.00 mmol, 1.20 equiv.), $Pd(OAc)_2$ (28.0 mg, 0.125 mmol, 0.0250 equiv.), PPh_3 (131 mg, 0.0500 mmol, 0.100 equiv.) and K_2CO_3 (1.86 g, 13.5 mmol, 2.70 equiv.) were dissolved in dimethoxyethane (5 ml) and water (6.6 mL). The resulting mixture was sparged with argon for 15 minutes then heated under reflux for 16 hours. Upon cooling to room temperature, the reaction mixture was transferred to a separating funnel, diluted with water and extracted three times with EtOAc. The combined organic layers were washed with brine, dried over anhydrous sodium sulfate, filtered and concentrated to give the crude product which was purified by flash column chromatography (3:97, EtOAc: pentane) to give 2-(4-(*tert*-butyl)phenyl)-5-fluoropyridine (**PP2**) (949 mg, 83%) as a white solid.

m.p. = 64-66 °C (EtOAc-pentane).

IR (film) ν_{max}/cm^{-1}: 2963, 1470, 1260, 1224, 823, 729.

^{1}H NMR (400 MHz, $CDCl_3$) δ = 8.53 (d, J = 2.9 Hz, 1H), 7.90–7.84 (m, 2H), 7.70 (ddd, J = 8.8, 4.3, 0.6 Hz, 1H), 7.52–7.48 (m, 2H), 7.45 (ddd, J = 8.7, 8.1, 2.9 Hz, 1H), 1.36 (s, 9H).

^{13}C NMR (101 MHz, $CDCl_3$) δ = 158.8 (d, J = 255.6 Hz), 153.9, 153.9, 137.8 (d, J = 23.6 Hz), 135.7, 126.6, 125.9, 123.6 (d, J = 18.4 Hz), 121.2 (d, J = 4.1 Hz), 34.8, 31.4.

^{19}F NMR (377 MHz, $CDCl_3$) δ = 130.3 (dd, J = 8.1, 4.4 Hz).

HRMS (ESI$^+$) $C_{16}H_{16}F_3N$ $[M+H]^+$ requires 230.1340; found 230.1340, Δ 0.3 ppm.

2-Phenyl-5-(trifluoromethyl)pyridine (PP3)

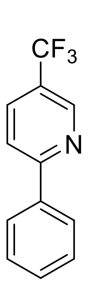

This compound was synthesized by a modification of a literature procedure.[1]

2-Bromo-5-trifluoromethylpyridine (2.26 g, 10.0 mmol, 1.00 equiv.), phenylboronic acid (1.46 g, 12.0 mmol, 1.20 equiv.), $Pd(OAc)_2$ (56.0 mg, 0.250 mmol, 0.0250 equiv.), PPh_3 (262 mg, 0.100 mmol, 0.100 equiv.) and K_2CO_3 (3.73 g, 13.5 mmol, 2.70 equiv.) were dissolved in dimethoxyethane (10 ml) and water (13.5 mL). The resulting mixture was sparged with argon for 15 minutes then heated under reflux for 16 hours. Upon cooling to room temperature, the reaction mixture was transferred to a separating funnel, diluted with water and extracted three times with EtOAc. The combined organic

layers were washed with brine, dried over anhydrous sodium sulfate, filtered and concentrated to give the crude product which was purified by flash column chromatography (1:19, EtOAc: pentane) then recrystallization from hexane to give 2-phenyl-5-(trifluoromethyl)pyridine (**PP3**) (949 mg, 36%) as a white needles.

m.p. = 60-62 °C (hexane).

IR (film) ν_{max}/cm^{-1}: 2980, 1598, 1388, 1330, 1293, 1241, 1085, 974, 955, 940, 739, 692, 652, 641.

^{1}H NMR (400 MHz, $CDCl_3$) δ = 8.99–8.89 (m, 1H), 8.07–8.01 (m, 2H), 7.98 (dd, *J* = 8.3, 0.7 Hz, 1H), 7.84 (d, *J* = 8.4 Hz, 1H), 7.55–7.45 (m, 3H).

^{13}C NMR (101 MHz, $CDCl_3$) δ = 160.7, 146.6 (q, *J* = 4.1 Hz), 138.0, 133.9 (q, *J* = 3.5 Hz), 130.1, 129.0, 127.3, 124.8 (q, *J* = 33.1 Hz), 123.8 (q, *J* = 272.2 Hz), 120.0.

^{19}F NMR (377 MHz, $CDCl_3$) δ = −62.2.

HRMS (ESI$^+$) $C_{12}H_8F_3N$ [M+H]$^+$ requires 224.0682; found 224.0682, Δ 0.1 ppm.

2-(4-(*tert*-Butyl)phenyl)-5-(trifluoromethyl)pyridine (PP4)

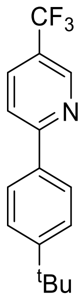

This compound was synthesized by a modification of a literature procedure.[1]

2-Chloro-5-trifluoromethylpyridine (908 mg, 5.0 mmol, 1.00 equiv.), (4-(*tert*-butyl)phenyl)boronic acid (1.07 g, 6.0 mmol, 1.20 equiv.), Pd(OAc)2 (28.0 mg, 0.125 mmol, 0.0250 equiv.), PPh_3 (131 mg, 0.050 mmol, 0.100 equiv.) and K_2CO_3 (1.87 g, 6.75 mmol, 2.70 equiv.) were dissolved in dimethoxyethane (5 ml) and water (6.75 mL). The resulting mixture was sparged with argon for 15 minutes then heated under reflux for 16 hours. Upon cooling to room temperature, the reaction mixture was transferred to a separating funnel, diluted with water and extracted three times with EtOAc. The combined organic layers were washed with brine, dried over anhydrous sodium sulfate, filtered and concentrated to give the crude product which was purified by flash column chromatography (1:19, EtOAc: pentane) to give 2-(4-(*tert*-butyl)phenyl)-5-(trifluoromethyl)pyridine (**PP4**) (1.23 g, 88%) as a white crystalline solid.

m.p. = 64-66°C (EtOAc-pentane).

IR (film) ν_{max}/cm^{-1}: 2917, 2849, 1601, 1326, 1163, 1122, 1084, 1012, 831.

^{1}H NMR (400 MHz, $CDCl_3$) δ = 8.97 – 8.91 (m, 1H), 8.00 – 7.92 (m, 3H), 7.83 (dt, J = 8.4, 0.8 Hz, 1H), 7.58 – 7.50 (m, 2H), 1.46 – 1.29 (m, 9H).

^{13}C NMR (101 MHz, $CDCl_3$) δ = 160.7, 153.5, 146.5 (q, J = 4.1 Hz), 135.2, 133.8 (q, J = 3.5 Hz), 127.0, 126.0, 124.5 (q, J = 32.9 Hz), 123.9 (q, J = 272.1 Hz), 119.6, 34.8, 31.2.

^{19}F NMR (377 MHz, $CDCl_3$) δ = -62.2.

HRMS (ESI$^+$) $C_{16}H_{16}F_3N$ [M+H]$^+$ requires 280.1308; found 280.1309, Δ 0.6 ppm.

Ir((5-F)ppy)$_3$ (C1)

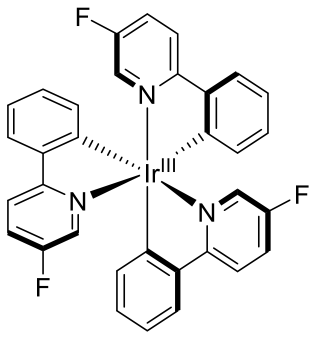

$IrCl_3$ (450 mg, 1.50 mmol, 1.00 equiv.) and 5-fluoro-2-phenylpyridine (**PP1**) (585 mg, 3.39 mmol, 2.26 equiv.) in methoxyethanol-water (20/10 mL) were heated under reflux for 16 hours then cooled to room temperature. The resulting precipitate was collected by vacuum filtration and washed with water to give the chloride dimer (670 mg, 61%) as a yellow solid.

The chloride dimer (22.8 mg, 0.0200 mmol, 1.00 equiv.), AgOTf (15.4 mg, 0.0600 mmol, 3.00 equiv.) and 5-fluoro-2-phenylpyridine (173 mg, 1.00 mmol, 50.0 equiv.) were added to a 10 mL reaction vial. The vial was sealed and evacuated and backfilled with argon three times. The reaction was heated to 160 °C for 16 h then cooled to room temperature. The reaction mixture was diluted with CH_2Cl_2 and purified directly by flash column chromatography (7:3, CH_2Cl_2: pentane) to give Ir(5-Fppy)$_3$ (**C1**) (25.7 mg, 91%) as a yellow solid.

m.p. > 250 °C (CH_2Cl_2/pentane).

IR (film) ν_{max}/cm^{-1}: 3041, 1477, 1442, 1431, 1260, 1230, 831, 774, 736, 722.

^{1}H NMR (500 MHz, $CDCl_3$) δ = 7.89 (dd, J = 8.9, 4.7 Hz, 3H), 7.59 (dd, J = 7.7, 1.4 Hz, 3H), 7.45 – 7.37 (m, 6H), 6.93 (ddd, J = 7.7, 7.5, 1.4 Hz, 3H), 6.85 (ddd, J = 7.3, 7.3, 1.4 Hz, 3H), 6.77 (dd, J = 7.6, 1.3 Hz, 3H).

^{13}C NMR (126 MHz, $CDCl_3$) δ = 163.3 (d, J = 3.3 Hz), 158.6 (d, J = 251.2 Hz), 158.6, 142.5, 137.0, 135.1 (d, J = 28.8 Hz), 129.9, 124.1 (obs. d), 124.0, 120.4, 119.8 (d, J = 5.7 Hz).

^{19}F NMR (471 MHz, $CDCl_3$) δ = −128.5.

HRMS (ESI$^+$) $C_{33}H_{21}F_3IrN_3$ [M+H]$^+$ requires 710.1391; found 710.1384, Δ 0.7 ppm.

Ir((5-F, 4'-*t*-Bu)ppy)$_3$ (C2)

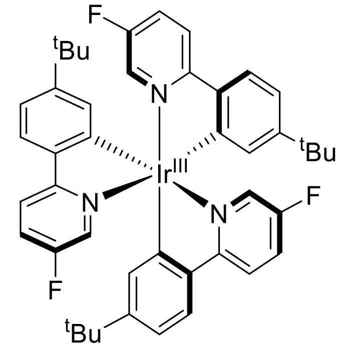

$IrCl_3$ (450 mg, 1.50 mmol, 1.00 equiv.) and 2-(4-(*tert*-butyl)phenyl)-5-fluoropyridine (**PP2**) (585 mg, 3.39 mmol, 2.26 equiv.) in methoxyethanol-water (20/10 mL) were heated under reflux for 16 hours then cooled to room temperature. The resulting precipitate was collected by vacuum filtration and washed with water to give the chloride dimer (125 mg, 73%) as a yellow solid.

The chloride dimer (68.4 mg, 0.0500 mmol, 1.00 equiv.), AgOTf (38.6 mg, 0.0600 mmol, 3.00 equiv.) and 2-(4-(tert-butyl)phenyl)-5-fluoropyridine (572 mg, 1.00 mmol, 50.0 equiv.) were added to a 10 mL reaction vial. The vial was sealed and evacuated and backfilled with argon three times. The reaction was heated to 160 °C for 16 h then cooled to room temperature. The reaction mixture was diluted with CH_2Cl_2 and purified directly by flash column chromatography (7:3, CH_2Cl_2: pentane) to give Ir((5-F)(4'-*t*-Bu)ppy)$_3$ (**C2**) (74.5 mg, 85%) as a yellow solid.

IR (film) ν_{max}/cm^{-1}: 2961, 1584, 1473, 1259, 1234, 843, 806, 629.

^{1}H NMR (500 MHz, $CDCl_3$) δ = 7.80 (dd, J = 9.2, 5.0 Hz, 3H), 7.48 (d, J = 8.2 Hz, 3H), 7.44 (app. t, J = 2.5 Hz, 3H), 7.35 (ddd, J = 9.0, 7.6, 2.8 Hz, 3H), 6.95 (dd, J = 8.2, 2.1 Hz, 3H), 6.82 (d, J = 2.1 Hz, 3H), 1.08 (s, 27H).

^{13}C NMR (126 MHz, $CDCl_3$) δ = 163.6 (d, J = 3.4 Hz), 158.5 (d, J = 250.2 Hz), 158.5, 152.2, 140.0, 135.0 (d, J = 28.6 Hz), 134.3, 123.8 (d, J = 19.1 Hz), 123.2, 119.2 (d, J = 5.6 Hz), 117.4, 34.5, 31.4.

^{19}F NMR (471 MHz, $CDCl_3$) δ = −129.6.

HRMS (ESI$^+$) $C_{45}H_{44}N_3F_3Ir$ [M+H]$^+$ requires 877.3192; found 877.3190, Δ 0.1 ppm.

Ir((5-CF$_3$)ppy)$_3$ (C3)

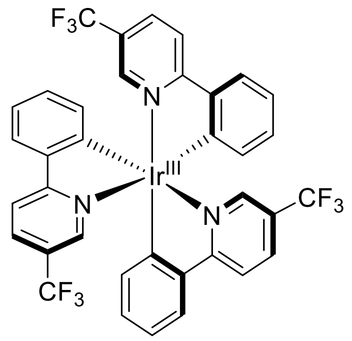

IrCl$_3$ (75 mg, 0.250 mmol, 1.00 equiv.) and 2-phenyl-5-(trifluoromethyl)pyridine (**PP3**) (125 mg, 0.562 mmol, 2.26 equiv.) in methoxyethanol-water (3/2 mL) were heated under reflux for 16 hours then cooled to room temperature. The resulting precipitate was collected by vacuum filtration and washed with water to give the chloride dimer (125 mg, 68%) as a yellow solid.

The chloride dimer dimer (67.2 mg, 0.0500 mmol, 1.00 equiv.), AgOTf (38.6 mg, 0.0600 mmol, 3.00 equiv.) and 2-phenyl-5-(trifluoromethyl)pyridine (557 mg, 1.00 mmol, 50.0 equiv.) were added to a 10 mL reaction vial. The vial was sealed and evacuated and backfilled with argon three times. The reaction was heated to 160 °C for 16 h then cooled to room temperature. The reaction mixture was diluted with CH_2Cl_2 and purified directly by flash column chromatography (7:3, CH_2Cl_2: pentane) to give Ir((5-CF$_3$)ppy)$_3$ (**C3**) (74.5 mg, 84%) as an orange solid.

IR (film) ν_{max}/cm^{-1}: 2359, 1612, 1582, 1328, 1309, 1265, 1232, 1175, 1135, 1084, 1054, 1034.

^{1}H NMR (500 MHz, $CDCl_3$) δ = 8.01 (d, J = 8.6 Hz, 3H), 7.84 (dd, J = 8.6, 2.1 Hz, 3H), 7.73 (dd, J = 7.8, 1.5 Hz, 3H), 7.67 (s, 3H), 6.99 (ddd, J = 7.8, 7.5, 1.4 Hz, 3H), 6.92 (ddd, J = 7.4, 7.2, 1.4 Hz, 3H), 6.82 (dd, J = 7.6, 1.3 Hz, 3H).

^{13}C NMR (126 MHz, $CDCl_3$) δ = 170.2, 144.0 (q, J = 4.7 Hz), 142.0, 137.3, 133.9 (d, J = 3.2 Hz), 131.6, 125.8, 125.1 (d, J = 33.8 Hz), 122.9 (d, J = 272.0 Hz), 121.0, 119.0.

^{19}F NMR (471 MHz, $CDCl_3$) δ = −62.7.

HRMS (ESI$^+$) $C_{36}H_{21}F_9IrN_3$ [M+H]$^+$ requires 860.1295; found 860.1288, Δ 0.3 ppm.

Ir((5-CF$_3$, 4'-*t*-Bu)ppy)$_3$ (4)

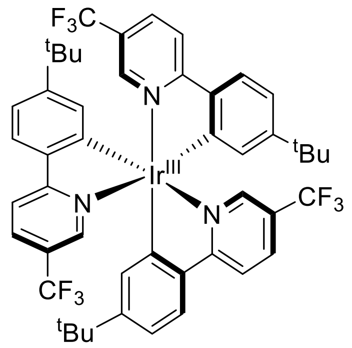

$IrCl_3$ (45 mg, 0.150 mmol, 1.00 equiv.) and 2-(4-(*tert*-butyl)phenyl)-5-(trifluoromethyl)pyridine (**PP4**) (95 mg, 0.340 mmol, 2.26 equiv.) in methoxyethanol-water (2/1 mL) were heated under reflux for 16 hours then cooled to room temperature. The resulting precipitate was collected by vacuum filtration and washed with water to give the chloride dimer (129 mg, 55%) as a yellow solid.

The chloride dimer (78.0 mg, 0.050 mmol, 1.00 equiv.), AgOTf (39.0 mg, 0.150 mmol, 3.00 equiv.) and 2-(4-(*tert*-butyl)phenyl)-5-(trifluoromethyl)pyridine (390 mg, 2.50 mmol, 50.0 equiv.) were added to a 10 mL reaction vial. The vial was sealed and evacuated and backfilled with argon three times. The reaction was heated to 160 °C for 16 h then cooled to room temperature. The reaction mixture was diluted with CH_2Cl_2 and purified directly by flash column chromatography (1:1, CH_2Cl_2: pentane, followed by 1:19, EtOAc: pentane) to give Ir((5-CF$_3$)(4'-*t*-Bu)ppy)$_3$ (**4**) (50.4 mg, 98%) as an orange solid.

IR (film) ν_{max}/cm^{-1}: 2963, 1693, 1611, 1586, 1328, 1310, 1128, 1087, 812, 773, 762, 736.

^{1}H NMR (500 MHz, $CDCl_3$) δ = 7.91 (d, *J* = 8.6 Hz, 3H), 7.75 (dd, *J* = 8.6, 2.1 Hz, 3H), 7.72 (s, 3H), 7.63 (d, *J* = 8.3 Hz, 3H), 7.02 (dd, *J* = 8.3, 2.1 Hz, 3H), 6.90 (d, *J* = 2.1 Hz, 3H), 1.11 (s, 9H).

^{13}C NMR (126 MHz, $CDCl_3$) δ = 170.4, 161.3, 154.2, 143.9 (d, *J* = 4.7 Hz), 139.5, 134.6, 133.5 (q, *J* = 3.3 Hz), 125.1, 124.7 (q, *J* = 33.9 Hz), 123.1 (q, *J* = 271.9 Hz), 118.6, 118.2, 34.7, 31.3.

^{19}F NMR (471 MHz, $CDCl_3$) δ = −62.8.

HRMS (ESI$^+$) $C_{48}H_{44}N_3F_9Ir$ $[M+H]^+$ requires 1027.3097; found 1027.3099, Δ 0.3 ppm.

3.3. Precursors

3,4-Dihydro-2H-pyran-6-carboxylic acid (P1)

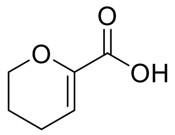

This compound was synthesized by a modification of a literature procedure.[4]

n-BuLi (2.5 M in hexane, 20 mL, 50.0 mmol, 1.00 equiv.) was cooled to 0 °C and dihydropyran (1.00 equiv.) was added. TMEDA (7.48 mL, 5.80 g, 50.0 mmol, 1.00 equiv.) was added and the reaction was warmed to room temperature and stirred for 2 hours. THF (50 mL) was added and the reaction was cooled to −78 °C. Powdered dry ice (~10 g) was added and the reaction was allowed to warm to room temperature and stirred for 3 hours. Et_2O was added and the resulting solution was extracted three times with water. The combined aqueous layers were acidified with 37% hydrochloric acid and extracted three times with EtOAc. The combined EtOAc extracts were washed with water, and concentrated to give 3,4-dihydro-2*H*-pyran-6-carboxylic acid (**P1**) (4.14 g, 65%) as a waxy solid.

m.p. = 40-43 °C (EtOAc).

IR (film) ν_{max}/cm^{-1}: broad signal 3467-3039, 2937, 2882, 1703, 1644, 1263, 1218, 1188, 1120, 1080, 1068, 1046, 919, 760, 702.

^{1}H NMR (400 MHz, $CDCl_3$) δ = 10.36 (br. s, 1H), 6.22 (t, J = 4.2 Hz, 1H), 4.19–4.02 (m, 2H), 2.31–2.15 (m, 2H), 1.93–1.79 (m, 2H).

^{13}C NMR (101 MHz, $CDCl_3$) δ = 167.6, 143.5, 113.9, 66.9, 21.4, 20.8.

HRMS (ESI$^+$) $C_6H_8O_3$ $[M+Na]^+$ requires 151.0366; found 151.0366, Δ 0.4 ppm.

4,5-Dihydrofuran-2-carboxylic acid (P2)

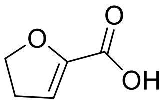

^{n}BuLi (2.5 M in hexane, 20 mL, 50.0 mmol, 1.00 equiv.) was cooled to 0 °C and dihydrofuran (3.78 mL, 3.50 g, 50 mmol, 1.00 equiv.) was added. TMEDA (7.48 mL, 5.80 g, 50.0 mmol, 1.00 equiv.) was added and the reaction was warmed to room temperature and stirred for 2 hours. THF (100 mL) was added and the reaction was cooled to −78 °C. Powdered dry ice (~10 g) was added and the reaction was allowed to warm to room temperature and stirred for 3 hours. Et_2O was added and the resulting solution was extracted three times with water. The combined aqueous layers were acidified with 37% hydrochloric acid and extracted three times with EtOAc. The combined EtOAc extracts were washed with water, and concentrated to give 4,5-dihydrofuran-2-carboxylic acid (**P2**) (2.74 g, 48%) as a waxy solid.

m.p. = 88-90 °C (EtOAc).

IR (film) ν_{max}/cm^{-1}: 3121-2566 (broad), 1689, 1632, 1622, 1443, 1310, 1262, 1221, 1179, 1132, 1086, 1072, 999, 932, 901, 739.

^{1}H NMR (400 MHz, $CDCl_3$) δ = 11.48 (br. s, 1H), 6.13 (t, J = 3.1 Hz, 1H), 4.51 (t, J = 9.8 Hz, 3H), 2.84 (td, J = 9.9, 3.1 Hz, 3H).

^{13}C NMR (101 MHz, $CDCl_3$) δ = 165.2, 148.0, 114.3, 71.0, 30.8.

HRMS (ESI$^+$) $C_5H_6O_3$ [M+Na]$^+$ requires 137.0209; found 137.0210, Δ 0.4 ppm.

***N*-Isobutylaniline (P3)**

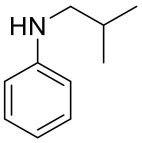

Prepared following **General Procedure A** using aniline (466 mg, 5.00 mmol, 1.00 equiv.), ^{n}BuLi (2.5 M in hexane, 2.20 mL, 5.50 mmol, 1.10 equiv.) and isobutyl iodide (631 µL, 1.01 g, 5.5 mmol, 1.10 equiv.). Flash column chromatography (3:97 Et_2O: pentane) afforded *N*-isobutylaniline (**P3**) (447 mg, 60%) as a colourless oil.

IR (film) ν_{max}/cm^{-1}: 3418, 2955, 1602, 1505, 745, 690.

^{1}H NMR (400 MHz, $CDCl_3$) δ = 7.23–7.14 (m, 2H), 6.71 (tt, *J* = 7.3, 1.1 Hz, 1H), 6.71-6.68 (m, 2H), 2.96 (d, *J* = 6.8 Hz, 2H), 1.92 (dh, *J* = 13.4, 6.7 Hz, 1H), 1.01 (d, *J* = 6.7 Hz, 6H).

^{13}C NMR (101 MHz, $CDCl_3$) δ = 148.7, 129.3, 117.1, 112.8, 52.0, 28.1, 20.6.

HRMS (ESI$^+$) $C_{10}H_{15}N$ [M+H]$^+$ requires 150.1277; found 150.1277, Δ 0.1 ppm.

***N*-Benzyl-4-chloroaniline (P4)**

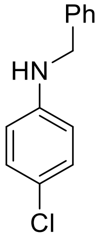

Prepared following **General Procedure A** using 4-chloroaniline (638 mg, 5.00 mmol, 1.00 equiv.), *n*-BuLi (2.5 M in hexane, 2.00 mL, 5.00 mmol, 1.00 equiv.) and benzyl bromide (654 µL, 5.0 mmol, 1.00 equiv.). Flash column chromatography (1:49, Et_2O: pentane) afforded *N*-benzyl-4-chloroaniline (**P4**) (842 mg, 77%) as a white solid.

m.p. = 40-42 °C (EtOAc).

IR (film) ν_{max}/cm^{-1}: 3422, 1602, 1509, 1494, 1482, 1449, 1325, 1295, 1274, 1172, 1124, 1089, 1070, 807, 731, 692.

^{1}H NMR (400 MHz, $CDCl_3$) δ = 7.27 (d, *J* = 4.4 Hz, 4H), 7.25 – 7.15 (m, 1H), 7.08 – 6.99 (m, 2H), 6.52 – 6.44 (m, 2H), 4.23 (s, 2H).

^{13}C NMR (101 MHz, $CDCl_3$) δ = 146.5, 138.8, 129.1, 128.7, 127.5, 127.4, 122.3, 114.1, 48.5.

HRMS (ESI$^+$) $C_{13}H_{13}NCl$ [M+H]$^+$ requires 218.0731; found 218.0732, Δ 0.4 ppm.

4-(*tert*-butyl)-*N*-methylaniline (P5)

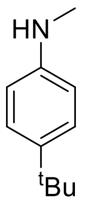

Prepared following **General Procedure A** using 4-(*tert*-butyl)-aniline (636 µL, 596 mg, 4.00 mmol), *n*-BuLi (2.5 M in hexane, 1.60 mL, 4.00 mmol, 1.00 equiv.) and iodomethane (249 µL, 568 mg, 4.00 mmol, 1.00 equiv.). Flash column chromatography (1:9, Et_2O: pentane) afforded 4-(*tert*-butyl)-*N*-methylaniline (**P5**) (290 mg, 44%) as a yellow oil.

IR (film) ν_{max}/cm^{-1}: 3412, 2959, 2900, 2868, 1617, 1521, 1481, 1461, 1392, 1320, 1263, 1156, 1059, 820.

^{1}H NMR (400 MHz, $CDCl_3$) δ = 7.30–7.24 (m, 2H), 6.67–6.60 (m, 2H), 2.87 (s, 3H), 1.33 (s, 9H).

^{13}C NMR (101 MHz, $CDCl_3$) δ = 146.8, 140.3, 126.0, 112.4, 33.9, 31.6, 31.1.

HRMS (ESI$^+$) $C_{11}H_{17}N$ [M+H]$^+$ requires 164.1434; found 164.1435, Δ 0.7ppm.

***N*-methyl-4-(methylthio)aniline (P6)**

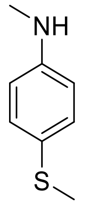

Prepared following **General Procedure A** using 4-(methylthio)aniline (695 mg, 5.00 mmol, 1.00 equiv.), *n*-BuLi (2.5 M in hexane, 2.00 mL, 5.00 mmol, 1.00 equiv.) and methyl iodide (311 µL, 710 mg, 5.0 mmol, 1.00 equiv.). Flash column chromatography (1:9→1:4, Et_2O: pentane) afforded *N*-methyl-4-(methylthio)aniline (**P6**) (372 mg, 49%) as a colourless oil.

IR (film) ν_{max}/cm^{-1}: 3412, 2980, 2916, 1599, 1502, 1312, 813.

^{1}H NMR (400 MHz, $CDCl_3$) δ = 7.28 – 7.17 (m, 2H), 6.68 – 6.46 (m, 2H), 2.83 (s, 3H), 2.41 (s, 3H).

^{13}C NMR (101 MHz, $CDCl_3$) δ = 148.4, 131.8, 124.1, 113.1, 30.8, 19.4.

HRMS (ESI$^+$) $C_8H_{11}NS$ [M+H]$^+$ requires 154.0684; found 154.0685, Δ 0.5 ppm.

***N*-Methylbenzen-d_5-amine (P7)**

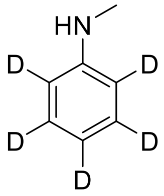

Prepared following **General Procedure A** using aniline-*d*7 (500 mg, 5.00 mmol, 1.00 equiv.), *n*-BuLi (2.5 M in hexane, 2.0 mL, 5.00 mmol, 1.00 equiv.) and methyl iodide (311 µL, 710 ,g, 5.00 mmol, 1.00 equiv.). Flash column chromatography eluting with 3% Et_2O-pentane afforded *N*-methylbenzen-d_5-amine (**P7**) (251 mg, 44%) as a colourless oil.

IR (film) ν_{max}/cm^{-1}: 3414, 3405, 2922, 1571, 1486, 1390.

^{1}H NMR (600 MHz, $CDCl_3$) δ = 2.85 (s, 3H).

^{13}C NMR (151 MHz, $CDCl_3$) δ = 149.3, 128.7 (t, *J* = 24.0 Hz, I = 1), 116.8 (t, *J* = 24.6 Hz, I = 1), 112.1 (t, *J* = 23.6 Hz, I = 1), 30.9.

HRMS (ESI$^+$) $C_7H_4D_5N$ [M+H]$^+$ requires 113.1122; found 113.1123, Δ 1.2 ppm.

2,4,6-Trichlorophenyl 4,5,6,7-tetrahydrooxepine-2-carboxylate (P8)

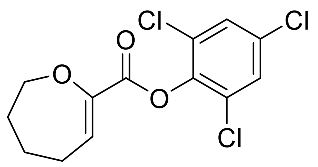

To a solution of ε-caprolactone (1.1 ml, 10 mmol, 1.0 equiv.) in anhydrous THF (20 mL) was added LiHMDS (1.0 M, 12 mL, 12 mmol, 1.2 equiv.) dropwise over 10 minutes at room temperature. The mixture was then cooled to -78 °C before phenyltriflimide (4.3 g, 12 mmol, 1.2 equiv.) in anhydrous THF (10 mL) was added. Stirring was continued for 2 h before the solution was allowed to warm to 0 °C, quenched with 10% aq. NaOH (25 mL), extracted with Et_2O (3 x 25 mL), dried over $MgSO_4$, filtered, and concentrated to afford an oil.

To a round bottom flask was added 2,4,6-trichlorophenyl formate (4.5 g, 20 mmol, 2.0 equiv.), $Pd(OAc)_2$ (67 mg, 0.3 mmol, 3 mol%), and Xantphos (347 mg, 0.6 mmol, 6 mol%). the flask was then evacuated and backfilled with argon three times before the crude triflate was added as a solution in sparged toluene (20 mL). The mixture was stirred for 5 minutes before Et_3N (2.8 mL, 20 mmol, 2.0 equiv.) was added dropwise over 10 min and the reaction was allowed to stir for 16 h. The crude reaction mixture was directly purified by flash column chromatography (1:3, EtOAc: pentane) to yield 2,4,6-trichlorophenyl 4,5,6,7-tetrahydrooxepine-2-carboxylate (**P8**) (2.5 g, 77% yield) as a white solid.

m.p. = 65-69 °C (EtOAc-pentane).

IR (film) ν_{max}/cm^{-1}: 3088, 1745, 1644, 1568, 1450, 1390, 1367, 1322, 1261, 1234, 1211, 1142, 1119, 1073, 1054, 979, 928, 892, 861, 843, 818, 803, 759, 745, 690.

^{1}H NMR (400 MHz, $CDCl_3$) δ = 7.37 (s, 2H), 6.74 (t, *J* = 6.2 Hz, 1H), 4.15 – 4.06 (m, 2H), 2.45 – 2.36 (m, 2H), 2.02 – 1.92 (m, 2H), 1.79 – 1.68 (m, 2H).

^{13}C NMR (101 MHz, $CDCl_3$) δ = 160.5, 147.8, 143.1, 131.9, 129.7, 128.6, 128.1, 126.1, 73.6, 31.4, 26.8, 24.5.

HRMS (ESI$^+$): $C_{13}H_{11}O_3Cl_3Na$ [M+Na]$^+$ requires 342.9666; found 342.9667, Δ 0.2 ppm.

3.4. Substrates

N-Methyl-*N*-phenyl-3,4-dihydro-2*H*-pyran-6-carboxamide (2a)

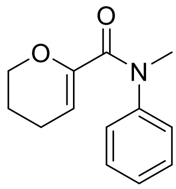

Prepared following **General Procedure B** using *N*-methylaniline (238 mg, 2.20 mmol, 1.10 equiv.) and 3,4-dihydro-2H-pyran-6-carboxylic acid (**P1**) (256, mg, 2.00 mmol, 1.00 equiv.). Flash column chromatography (1:4, EtOAc:pentane) afforded *N*-methyl-*N*-phenyl-3,4-dihydro-2*H*-pyran-6-carboxamide (**2a**) (279 mg, 64%) as a white solid.

m.p. = 56-58°C (EtOAc-pentane).

IR (film) ν_{max}/cm^{-1}: 2978, 2938, 1643, 1596, 1498, 1379, 1370, 1055, 920, 775, 740, 703.

^{1}H NMR (400 MHz, $CDCl_3$) δ = 7.41–7.32 (m, 2H), 7.32–7.22 (m, 1H), 7.25–7.15 (m, 2H), 5.42 (t, J = 4.0 Hz, 1H), 3.55–3.48 (m, 2H), 3.35 (s, 3H), 2.02 (td, J = 6.4, 4.0 Hz, 2H), 1.72–1.61 (m, 2H).

^{13}C NMR (101 MHz, $CDCl_3$) δ = 166.1, 148.6, 145.2, 129.0, 126.6, 125.7, 107.1, 65.8, 38.1, 21.7, 20.2.

HRMS (ESI$^+$) $C_{13}H_{15}NO_2$ [M+Na]$^+$ requires 240.0995; found 240.0997, Δ 1.0 ppm.

N-Benzyl-*N*-phenyl-3,4-dihydro-2*H*-pyran-6-carboxamide (5a)

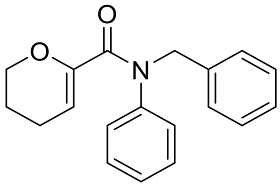

Prepared following **General Procedure B** using *N*-benzylaniline (201 mg, 1.10 mmol, 1.10 equiv.) and 3,4-dihydro-2*H*-pyran-6-carboxylic acid (**P1**) (128 mg, 1.00 mmol, 1.00 equiv.). Flash column chromatography (3:7, Et_2O:pentane) afforded *N*-benzyl-*N*-phenyl-3,4-dihydro-2*H*-pyran-6-carboxamide (**5a**) (195 mg, 66%) as a white solid.

m.p. = 60-62°C (Et_2O-pentane).

IR (film) ν_{max}/cm^{-1}: 2976, 2962, 1636, 1392, 751, 699.

^{1}H NMR (400 MHz, $CDCl_3$) δ = 7.32–7.23 (m, 7H), 7.23–7.16 (m, 1H), 7.08–7.02 (m, 2H), 5.45 (t, *J* = 4.0 Hz, 1H), 4.98 (s, 2H), 3.56–3.39 (m, 2H), 2.06–1.94 (m, 2H), 1.72–1.58 (m, 2H).

^{13}C NMR (101 MHz, $CDCl_3$) δ = 166.0, 148.6, 143.6, 137.5, 128.7, 128.6, 128.5, 127.4, 126.7, 126.6, 107.4, 65.8, 53.6, 21.7, 20.2.

HRMS (ESI$^+$) $C_{19}H_{19}NO_2$ [M+H]$^+$ requires 294.1489; found 294.1490, Δ 0.5 ppm.

***N*-Isobutyl-*N*-phenyl-3,4-dihydro-2*H*-pyran-6-carboxamide (6a)**

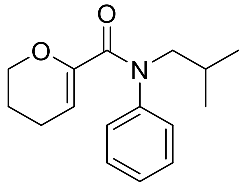

Prepared following **General Procedure B** using *N*-isobutylaniline (**P3**) (223 mg, 1.5 mmol, 1.00 equiv.) and 3,4-dihydro-2*H*-pyran-6-carboxylic acid (**P1**) (192 mg, 1.50 mmol, 1.00 equiv.). Flash column chromatography (1:4→3:7, EtOAc: pentane) afforded *N*-isobutyl-*N*-phenyl-3,4-dihydro-2*H*-pyran-6-carboxamide (**6a**) (305 mg, 78%) as a white solid.

m.p. = 70-72 °C (EtOAc/pentane).

IR (film) v_{max}/cm^{-1}: 2962, 1637, 1594, 1492, 1398, 1386, 1340, 1307, 1295, 1280, 1232, 1115, 1070, 1056, 916, 796, 745, 700, 684.

^{1}H NMR (400 MHz, $CDCl_3$) δ = 7.35 – 7.29 (m, 2H), 7.24 – 7.19 (m, 1H), 7.18 – 7.14 (m, 2H), 5.31 (t, *J* = 4.0 Hz, 1H), 3.62 (d, *J* = 7.5 Hz, 2H), 3.49 – 3.35 (m, 2H), 1.96 (td, *J* = 3.9, 6.4 Hz, 2H), 1.90 – 1.78 (m, 1H), 1.68 – 1.55 (m, 2H), 0.90 (d, *J* = 6.7 Hz, 7H).

^{13}C NMR (101 MHz, $CDCl_3$) δ = 166.2, 149.0, 143.8, 128.8, 126.6, 126.5, 106.5, 65.7, 56.6, 27.0, 21.7, 20.3, 20.1.

HRMS (ESI$^+$) $C_{16}H_{21}NO_2$ $[M+H]^+$ requires 260.1645; found 260.1645, Δ 0.1 ppm.

***N*-Phenyl-*N*-propyl-3,4-dihydro-2*H*-pyran-6-carboxamide (29)**

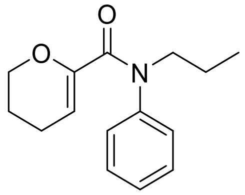

Prepared following **General Procedure B** using *N*-propylaniline (297 mg, 2.2 mmol, 1.1 equiv.) and 3,4-dihydro-2H-pyran-6-carboxylic acid (**P1**) (256 mg, 2.00 mmol, 1.00 equiv.). Flash column chromatography (1:4, EtOAc: pentane) afforded *N*-phenyl-*N*-propyl-3,4-dihydro-2*H*-pyran-6-carboxamide (**29**) (220 mg, 44%) as a white solid.

m.p. = 46-48°C (EtOAc-pentane).

IR (film) v_{max}/cm^{-1}: 1654, 1632, 1598, 1496, 1457, 1446, 1400, 1305, 1287, 1277, 1224, 1160, 1116, 1079, 1055, 919, 782, 767, 752, 702.

^{1}H NMR (400 MHz, $CDCl_3$) δ = 7.37 – 7.30 (m, 2H), 7.25 – 7.19 (m, 1H), 7.18 – 7.11 (m, 2H), 5.35 (t, *J* = 4.0 Hz, 1H), 3.86 – 3.61 (m, 2H), 3.59 – 3.19 (m, 2H), 1.97 (td, *J* = 6.4, 4.0 Hz, 2H), 1.74 – 1.49 (m, 4H), 0.88 (t, *J* = 7.4 Hz, 3H).

^{13}C NMR (101 MHz, $CDCl_3$) δ = 165.7, 148.7, 143.5, 128.7, 126.6, 126.5, 106.6, 65.6, 51.6, 21.6, 20.8, 20.0, 11.3.

HRMS (ESI$^+$) $C_{15}H_{20}NO_2$ $[M+H]^+$ requires 246.1489; found 246.1488, Δ -0.3 ppm.

***N*-Allyl-*N*-phenyl-3,4-dihydro-2*H*-pyran-6-carboxamide (8a)**

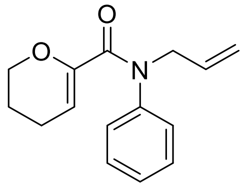

Prepared following **General Procedure B** using *N*-allylaniline (146 mg, 1.10 mmol, 1.10 equiv.) and 3,4-dihydro-2H-pyran-6-carboxylic acid (**P1**) (128 mg, 1.00 mmol, 1.00 equiv.). Flash column chromatography (1:4, EtOAc: pentane) afforded *N*-allyl-N-phenyl-3,4-dihydro-2H-pyran-6-carboxamide (**8a**) (146 mg, 60%) as a white solid.

m.p. = 42-44 °C (EtOAc-pentane).

IR (film) ν_{max}/cm^{-1}: 2970, 2939, 2921, 2867, 1634, 1493, 1387, 1277, 1052, 929, 917, 746, 699, 683.

^{1}H NMR (400 MHz, $CDCl_3$) δ = 7.36–7.29 (m, 2H), 7.25–7.19 (m, 1H), 7.17–7.11 (m, 2H), 5.89 (ddt, *J* = 17.5, 9.8, 6.0 Hz, 1H), 5.42 (t, *J* = 4.0 Hz, 1H), 5.16–5.11 (m, 1H), 5.11–5.07 (m, 1H), 4.36 (dt, *J* = 6.0, 1.4 Hz, 2H), 3.51–3.33 (m, 2H), 2.08–1.92 (m, 2H), 1.70–1.59 (m, 2H).

^{13}C NMR (101 MHz, $CDCl_3$) δ = 165.7, 148.6, 143.7, 133.2, 128.8, 126.6, 126.4, 117.7, 107.3, 65.8, 53.0, 21.7, 20.2.

HRMS (ESI$^+$) $C_{15}H_{17}NO_2$ [M+H]$^+$ requires 244.1332; found 244.1332, Δ 0.1 ppm.

(3,4-Dihydro-2*H*-pyran-6-yl)(3,4-dihydroquinolin-1(2*H*)-yl)methanone (9a)

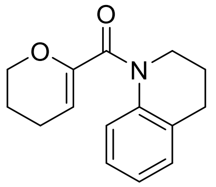

Prepared following **General Procedure B** using 1,2,3,4-tetrahydroquinoline (199 mg, 1.50 mmol, 1.00 equiv.) and 3,4-dihydro-2*H*-pyran-6-carboxylic acid (**P1**) (192 mg, 1.50 mmol, 1.00 equiv.). Flash column chromatography (1:4, EtOAc: pentane) afforded (3,4-dihydro-2*H*-pyran-6-yl)(3,4-dihydroquinolin-1(2*H*)-yl)methanone (**9a**) (195 mg, 66%) as a white solid.

m.p. = 62-64 °C (EtOAc-pentane).

IR (film) ν_{max}/cm^{-1}: 2975, 2961, 2931, 2875, 1629, 1579, 1492, 1376, 1361, 1338, 1327, 1281, 1046, 913, 762, 743.

^{1}H NMR (400 MHz, $CDCl_3$) δ = 7.29 – 7.20 (m, 1H), 7.14 – 7.07 (m, 2H), 7.07 – 7.00 (m, 1H), 5.44 (t, *J* = 4.0 Hz, 1H), 3.83 – 3.74 (m, 5H), 2.75 (t, *J* = 6.6 Hz, 2H), 2.10 (td, *J* = 6.4, 4.0 Hz, 2H), 1.97 (p, *J* = 6.6 Hz, 2H), 1.84 – 1.74 (m, 2H).

^{13}C NMR (101 MHz, $CDCl_3$) δ = 165.6, 149.3, 139.4, 131.0, 128.4, 125.8, 124.6, 123.2, 106.9, 66.2, 44.3, 26.9, 24.0, 21.8, 20.4.

HRMS (ESI$^+$) $C_{15}H_{17}NO_2$ [M+H]$^+$ requires 244.1332; found 244.1333, Δ 0.3 ppm.

***N*-(4-Methoxybenzyl)-*N*-phenyl-3,4-dihydro-2*H*-pyran-6-carboxamide (10a)**

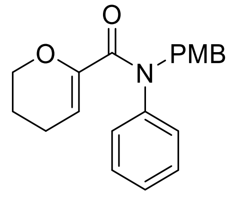

Prepared following **General Procedure B** using 4-methoxy-*N*-methylaniline (439 mg, 2.2 mmol, 1.1 equiv.) and 3,4-dihydro-2H-pyran-6-carboxylic acid (**P1**) (256 mg, 2.0 mmol, 1.0 equiv.). Flash column chromatography (3:17, EtOAc: pentane) afforded *N*-(4-methoxybenzyl)-*N*-phenyl-3,4-dihydro-2*H*-pyran-6-carboxamide (**10a**) (381 mg, 59%) as a white solid.

m.p. = 74-76°C (EtOAc-pentane).

IR (film) ν_{max}/cm^{-1}: 1624, 1596, 1586, 1512, 1495, 1430, 1399, 1320, 1304, 1294, 1276, 1244, 1230, 1186, 1135, 1070, 1059, 1026, 923, 820, 802, 756, 739, 694, 623.

^{1}H NMR (400 MHz, $CDCl_3$) δ = 7.28 – 7.09 (m, 5H), 7.03 – 6.95 (m, 2H), 6.80 – 6.72 (m, 2H), 5.39 (t, *J* = 4.0 Hz, 1H), 4.87 (s, 2H), 3.75 (s, 3H), 3.47 – 3.40 (m, 2H), 1.96 (td, *J* = 6.4, 4.0 Hz, 2H), 1.66 – 1.56 (m, 2H).

^{13}C NMR (101 MHz, $CDCl_3$) δ = 165.8, 158.8, 148.6, 143.4, 129.9, 129.5, 128.6, 126.7, 126.6, 113.7, 107.1, 65.6, 55.2, 52.9, 21.5, 20.1.

HRMS (ESI^+) $C_{20}H_{22}NO_3$ $[M+H]^+$ requires 324.1594; found 324.1591, Δ -1.1 ppm.

***N*-(3-Fluorophenyl)-*N*-methyl-3,4-dihydro-2*H*-pyran-6-carboxamide (11a)**

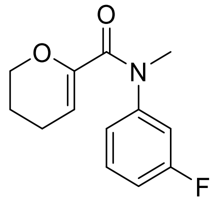

Prepared following **General Procedure B** using 3-fluoro-*N*-methylaniline (631 µL, 5.5 mmol, 1.1 equiv.) and 3,4-dihydro-2H-pyran-6-carboxylic acid (**P1**) (640 mg, 5.0 mmol, 1.0 equiv.). Flash column chromatography (1:4, EtOAc: pentane) afforded *N*-(3-fluorophenyl)-*N*-methyl-3,4-dihydro-2*H*-pyran-6-carboxamide (**11a**) (674 mg, 57%) as a yellow oil.

IR (film) ν_{max}/cm^{-1}: 1645, 1608, 1592, 1488, 1464, 1369, 1344, 1312, 1287, 1265, 1231, 1201, 1151, 1098, 1070, 1057, 1023, 967, 920, 857, 829, 786, 765, 749, 697.

^{1}H NMR (400 MHz, $CDCl_3$) δ = 7.24 – 7.18 (m, 1H), 6.92 – 6.74 (m, 3H), 5.39 (t, *J* = 4.0 Hz, 1H), 3.54 – 3.37 (m, 2H), 3.23 (s, 3H), 1.94 (td, *J* = 6.4, 4.0 Hz, 2H), 1.67 – 1.47 (m, 2H).

^{19}F NMR (377 MHz, $CDCl_3$) δ = -108.5 – -115.3 (m)

^{13}C NMR (101 MHz, $CDCl_3$) δ = 164.9 (d, *J* = 182.0 Hz), 161.5, 148.2, 146.5 (d, *J* = 9.6 Hz), 129.8 (d, *J* = 8.9 Hz), 121.2 (d, *J* = 3.1 Hz), 113.3 (d, *J* = 20.8 Hz), 112.8 (d, *J* = 22.5 Hz), 107.5, 65.7, 37.9, 21.5, 20.1

HRMS (ESI^+) $C_{13}H_{15}NO_2F$ $[M+H]^+$ requires 236.1081; found 236.1081, Δ -0.2 ppm.

***N*-(3-Chlorophenyl)-*N*-methyl-3,4-dihydro-2*H*-pyran-6-carboxamide (12a)**

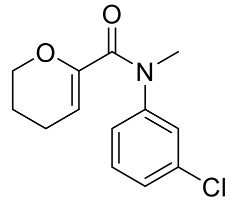

Prepared following **General Procedure B** using 3-chloro-*N*-methylaniline (213 mg, 1.5 mmol, 1.0 equiv.) and 3,4-dihydro-2H-pyran-6-carboxylic acid (**P1**) (211 mg, 1.65 mmol, 1.1 equiv.). Flash column chromatography (1:4, EtOAc: pentane) afforded *N*-(3-chlorophenyl)-*N*-methyl-3,4-dihydro-2*H*-pyran-6-carboxamide (**12a**) as a light yellow solid.

m.p. = 50-54°C (EtOAc-pentane).

IR (film) ν_{max}/cm^{-1}: 1660, 1641, 1590, 1576, 1479, 1464, 1447, 1434, 1418, 1383, 1368, 1345, 1311, 1296, 1286, 1258, 1228, 1165, 1112, 1092, 1079, 1050, 1020, 915, 903, 804, 784, 747, 734, 695.

^{1}H NMR (400 MHz, $CDCl_3$) δ = 7.27 (t, *J* = 7.9 Hz, 1H), 7.23 – 7.16 (m, 1H), 7.11 – 7.02 (m, 1H), 5.48 (t, *J* = 4.0 Hz, 1H), 3.71 – 3.44 (m, 2H), 3.31 (s, 3H), 2.03 (td, *J* = 6.4, 4.0 Hz, 2H), 1.74 – 1.54 (m, 2H).

^{13}C NMR (101 MHz, $CDCl_3$) δ = 165.6, 148.1, 146.1, 134.2, 129.7, 126.5, 125.7, 123.6, 107.6, 65.7, 37.8, 21.5, 20.1.

HRMS (ESI$^+$) $C_{13}H_{15}NO_2Cl$ $[M+H]^+$ requires 252.0786; found 252.0785, Δ -0.2 ppm

***N*-(3-Methoxyphenyl)-*N*-methyl-3,4-dihydro-2*H*-pyran-6-carboxamide (13a)**

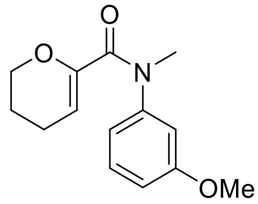

Prepared following **General Procedure B** using 3-methoxy-*N*-methylaniline (226 mg, 1.65 mmol, 1.10 equiv.) and 3,4-dihydro-2*H*-pyran-6-carboxylic acid (**P1**) (192, mg, 1.50 mmol, 1.00 equiv.). Flash column chromatography (2:3, EtOAc:pentane) afforded *N*-(3-methoxyphenyl)-*N*-methyl-3,4-dihydro-2*H*-pyran-6-carboxamide (**13a**) (203 mg, 55%) as a brown oil.

IR (film) ν_{max}/cm^{-1}: 2970, 2932, 1641, 1597, 1220, 1156, 1056, 1042, 697.

^{1}H NMR (400 MHz, $CDCl_3$) δ = 7.22 (dd, *J* = 8.1, 8.1 Hz, 1H), 6.79 – 6.73 (m, 2H), 6.71 (dd, *J* = 2.2, 2.2 Hz, 1H), 5.38 (t, *J* = 4.0 Hz, 1H), 3.79 (s, 3H), 3.60 – 3.50 (m, 2H), 3.31 (s, 3H), 2.03 – 1.92 (m, 2H), 1.70 – 1.59 (m, 2H).

^{13}C NMR (101 MHz, $CDCl_3$) δ = 166.1, 160.1, 148.6, 146.3, 129.6, 118.0, 112.2, 111.4, 106.9, 65.9, 55.5, 38.0, 21.7, 20.2.

HRMS (ESI$^+$) $C_{14}H_{17}O_3N$ $[M+H]^+$ requires 248.1281; found 248.1283, Δ 0.9 ppm.

***N*-Methyl-*N*-(*m*-tolyl)-3,4-dihydro-2*H*-pyran-6-carboxamide (14a)**

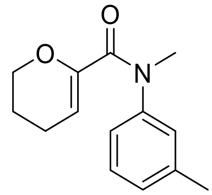

Prepared following **General Procedure B** using *N*,3-dimethylaniline (666 mg, 5.5 mmol, 1.1 equiv.) and 3,4-dihydro-2H-pyran-6-carboxylic acid (**P1**) (640 mg, 5.0 mmol, 1.0 equiv.). Flash column chromatography (1:4, EtOAc: pentane) afforded *N*-methyl-*N*-(*m*-tolyl)-3,4-dihydro-2*H*-pyran-6-carboxamide (**14a**) (546 mg, 47%) as a yellow oil.

IR (film) ν_{max}/cm^{-1}: 1643, 1605, 1588, 1492, 1464, 1447, 1370, 1344, 1305, 1287, 1231, 1191, 1160, 1105, 1083, 1060, 1028, 920, 789, 748, 702, 679.

^{1}H NMR (400 MHz, $CDCl_3$) δ = 7.21 (t, *J* = 7.7 Hz, 1H), 7.06 – 6.93 (m, 3H), 5.38 (t, *J* = 3.9 Hz, 1H), 3.58 – 3.47 (m, 2H), 3.31 (s, 3H), 2.35 (s, 3H), 2.00 (td, *J* = 6.4, 4.0 Hz, 2H), 1.73 – 1.52 (m, 4H).

^{13}C NMR (101 MHz, $CDCl_3$) δ = 166.0, 148.5, 144.9, 138.7, 128.6, 127.1, 126.1, 122.5, 106.8, 65.7, 37.9, 21.6, 21.3, 20.1.

HRMS (ESI$^+$) $C_{14}H_{18}NO_2$ [M+H]$^+$ requires 232.1332; found 232.1332, Δ -0.1 ppm.

***N*-(3,5-Difluorophenyl)-*N*-methyl-3,4-dihydro-2*H*-pyran-6-carboxamide (15a)**

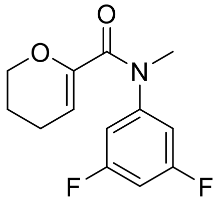

Prepared following **General Procedure B** using 3,5-difluoro-N-methylaniline (235 mg, 1.65 mmol, 1.10 equiv.) and 3,4-dihydro-2H-pyran-6-carboxylic acid (**P1**) (192 mg, 1.5 mmol, 1.00 equiv.). Flash column chromatography (1:4→3:7, EtOAc: pentane) afforded *N*-(3,5-difluorophenyl)-*N*-methyl-3,4-dihydro-2*H*-pyran-6-carboxamide (**15a**) (106 mg, 28%) as a colourless oil.

IR (film) ν_{max}/cm^{-1}: 2933, 1613, 1601, 1324, 1118, 989.

^{1}H NMR (500 MHz, $CDCl_3$) δ = 6.77 – 6.65 (m, 3H), 5.55 (t, *J* = 4.0 Hz, 1H), 3.63 – 3.58 (m, 2H), 3.31 (s, 3H), 2.10 – 2.02 (m, 2H), 1.73 – 1.67 (m, 2H).

^{13}C {^{1}H, ^{19}F} NMR (126 MHz, $CDCl_3$) δ = 165.6, 162.9, 148.1, 147.4, 108.8, 108.3, 102.0, 66.0, 37.9, 21.6, 20.3.

^{19}F NMR (471 MHz, $CDCl_3$) δ = -109.5.

HRMS (ESI$^+$) $C_{13}H_{13}NO_2F_2$ [M+H]$^+$ requires 254.0987; found 254.0986, Δ 0.3 ppm.

***N*-(4-Fluorophenyl)-*N*-methyl-3,4-dihydro-2*H*-pyran-6-carboxamide (16a)**

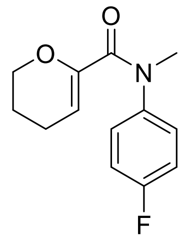

Prepared following **General Procedure B** using 4-fluoro-*N*-methylaniline (208 mg, 1.65 mmol, 1.10 equiv.) and 3,4-dihydro-2*H*-pyran-6-carboxylic acid (**P1**) (192 mg, 1.50 mmol, 1.00 equiv.). Flash column chromatography (1:4, EtOAc: pentane) afforded *N*-(4-fluorophenyl)-*N*-methyl-3,4-dihydro-2*H*-pyran-6-carboxamide (**16a**) (208 mg, 59%) as a white solid.

m.p. = 62-64 °C (EtOAc-pentane).

IR (film) ν_{max}/cm^{-1}: 2932, 1641, 1465, 1432, 1371, 1286, 1164, 1058, 841, 767, 721, 631.

^{1}H NMR (400 MHz, $CDCl_3$) δ = 7.16–7.10 (m, 2H), 7.07–6.96 (m, 2H), 5.41 (t, *J* = 4.0 Hz, 1H), 3.57–3.48 (m, 3H), 3.28 (s, 3H), 2.08–1.97 (m, 2H), 1.67–1.60 (m, 2H).

^{13}C NMR (101 MHz, $CDCl_3$) δ = 165.9, 161.0 (d, *J* = 246.1 Hz), 148.3, 140.9, 127.4 (d, *J* = 8.6 Hz), 115.6 (d, *J* = 22.7 Hz), 107.1, 65.7, 38.1, 21.5, 20.0.

^{19}F NMR (377 MHz, $CDCl_3$) δ = −115.4.

HRMS (ESI$^+$) $C_{13}H_{14}N_1O_2F$ $[M+Na]^+$ requires 258.0901; found 258.0903, Δ 0.9 ppm.

***N*-Benzyl-*N*-(4-chlorophenyl)-3,4-dihydro-2*H*-pyran-6-carboxamide (17a)**

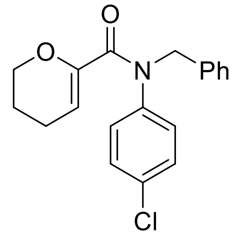

Prepared following **General Procedure B** using *N*-benzyl-4-chloroaniline (**P4**) (479 mg, 2.2 mmol, 1.1 equiv.) and 3,4-dihydro-2H-pyran-6-carboxylic acid (**P1**) (256 mg, 2.0 mmol, 1.0 equiv.). Flash column chromatography (3:17, EtOAc: pentane) afforded *N*-benzyl-*N*-(4-chlorophenyl)-3,4-dihydro-2*H*-pyran-6-carboxamide (**17a**) (381 mg, 59%) as a white solid.

m.p. = 66-69°C (EtOAc-pentane).

IR (film) ν_{max}/cm^{-1}: 1651, 1627, 1493, 1434, 1412, 1394, 1319, 1303, 1282, 1229, 1139, 1090, 1077, 1048, 1030, 1016, 920, 858, 817, 744, 722, 696, 643.

^{1}H NMR (400 MHz, $CDCl_3$) δ = 7.39 – 7.22 (m, 7H), 7.11 – 6.99 (m, 2H), 5.57 (t, *J* = 4.0 Hz, 1H), 5.00 (s, 2H), 3.58 (t, *J* = 5.1 Hz, 2H), 2.09 (td, *J* = 6.4, 4.0 Hz, 2H), 1.73 (dd, *J* = 6.4, 4.6 Hz, 2H).

^{13}C NMR (101 MHz, $CDCl_3$) δ = 165.7, 148.2, 142.0, 137.0, 132.2, 128.8 (2C), 128.5 (2C), 128.4 (2C), 127.8, 127.8, 127.4, 107.8, 65.7, 53.5, 21.5, 20.1.

HRMS (ESI$^+$) $C_{19}H_{19}NO_2Cl$ $[M+H]^+$ requires 328.1099; found 328.1097, Δ -0.5 ppm.

N-(4-Bromophenyl)-N-methyl-3,4-dihydro-2H-pyran-6-carboxamide (18a)

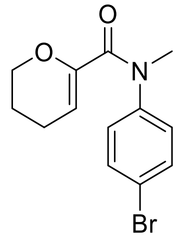

Prepared following **General Procedure B** using 4-bromo-*N*-methylaniline (280 mg, 1.5 mmol, 1.0 equiv.) and 3,4-dihydro-2H-pyran-6-carboxylic acid (**P1**) (211 mg, 1.65 mmol, 1.1 equiv.). Flash column chromatography (1:4, EtOAc: pentane) *N*-(4-bromophenyl)-*N*-methyl-3,4-dihydro-2*H*-pyran-6-carboxamide (**18a**) (262 mg, 59%) as a yellow solid.

m.p. = 67-70°C (EtOAc-pentane).

IR (film) ν_{max}/cm^{-1}: 1655, 1639, 1586, 1488, 1464, 1446, 1425, 1405, 1377, 1365, 1342, 1308, 1294, 1283, 1228, 1163, 1105, 1070, 1055, 1010, 920, 838, 821, 799, 749, 714.

^{1}H NMR (400 MHz, $CDCl_3$) δ = 7.51 – 7.38 (m, 2H), 7.10 – 6.97 (m, 2H), 5.45 (t, *J* = 4.0 Hz, 1H), 3.61 – 3.50 (m, 2H), 3.29 (s, 3H), 2.01 (td, *J* = 6.4, 4.0 Hz, 2H), 1.72 – 1.60 (m, 2H).

^{13}C NMR (101 MHz, $CDCl_3$) δ = 165.7, 148.2, 144.1, 132.0, 127.2, 119.8, 107.5, 65.7, 37.9, 21.5, 20.1.

HRMS (ESI$^+$) $C_{13}H_{15}NO_2Br$ [M+H]$^+$ requires 296.0281; found 296.0282, Δ 0.3 ppm.

N-Methyl-N-(p-tolyl)-3,4-dihydro-2H-pyran-6-carboxamide (19a)

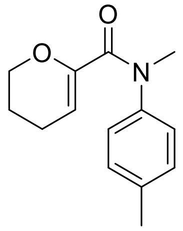

Prepared following **General Procedure B** using *N,*4-dimethylaniline (532 mg, 4.40 mmol, 1.10 equiv.) and 3,4-dihydro-2*H*-pyran-6-carboxylic acid (**P1**) (512 mg, 4.00 mmol, 1.00 equiv.). Flash column chromatography (1:4, EtOAc: pentane) afforded *N*-methyl-*N*-(p-tolyl)-3,4-dihydro-2H-pyran-6-carboxamide (**19a**) (693 mg, 75%) as a white solid.

m.p. = 46-48 °C (EtOAc-pentane).

IR (film) ν_{max}/cm^{-1}: 2977, 2931, 1633, 1511, 1366, 1053, 917, 826, 748.

^{1}H NMR (400 MHz, $CDCl_3$) δ = 7.16–7.10 (m, 2H), 7.08–7.01 (m, 2H), 5.34 (t, *J* = 4.0 Hz, 1H), 3.57–3.50 (m, 3H), 3.29 (s, 3H), 2.34 (s, 3H), 2.02–1.93 (m, 3H), 1.68–1.59 (m, 3H).

^{13}C NMR (101 MHz, $CDCl_3$) δ = 166.1, 148.5, 142.4, 136.2, 129.4, 125.4, 106.6, 65.7, 38.0, 21.6, 21.0, 20.0.

HRMS (ESI$^+$) $C_{14}H_{17}NO_2$ [M+H]$^+$ requires 232.1332; found 232.1332, Δ 0.2 ppm.

***N*-Methyl-*N*-(4-(trifluoromethyl)phenyl)-3,4-dihydro-2*H*-pyran-6-carboxamide (20a)**

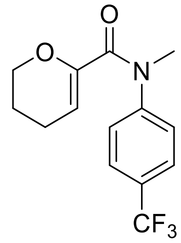

Prepared following **General Procedure B** using *N*-methyl-4-(trifluoromethyl)aniline (289 mg, 1.65 mmol, 1.1 equiv.) and 3,4-dihydro-2H-pyran-6-carboxylic acid (**P1**) (192 mg, 1.5 mmol, 1.0 equiv.). Flash column chromatography (1:4, EtOAc: pentane) afforded *N*-methyl-*N*-(4-(trifluoromethyl)phenyl)-3,4-dihydro-2*H*-pyran-6-carboxamide (**20a**) (280 mg, 75%) as a yellow oil.

IR (film) ν_{max}/cm^{-1}: 1743, 1649, 1612, 1519, 1371, 1325, 1230, 1165, 1122, 1068, 1017, 920, 849, 752.

^{1}H NMR (400 MHz, $CDCl_3$) δ = 7.57 – 7.49 (m, 2H), 7.22 (d, *J* = 8.3 Hz, 2H), 5.45 (t, *J* = 4.0 Hz, 1H), 3.47 – 3.40 (m, 4H), 3.27 (s, 3H), 1.96 (td, *J* = 6.4, 4.0 Hz, 3H), 1.64 – 1.54 (m, 3H).

^{19}F NMR (377 MHz, $CDCl_3$) δ = -62.4.

^{13}C NMR (101 MHz, $CDCl_3$) δ = 165.7, 148.2, 148.0, 128.3 (q, *J* = 32.7 Hz), 126.0 (q, *J* = 3.7 Hz), 125.5, 123.9 (q, *J* = 271.8 Hz), 108.1, 65.7, 37.8, 21.5, 20.1166.1, 158.0, 148.6, 137.8, 126.9, 114.0, 106.4, 65.7, 55.4, 38.1, 21.6, 20.0.

HRMS (ESI$^+$) $C_{14}H_{14}F_3NO_3Na$ [M+Na]$^+$ requires 308.0869; found 308.0880, Δ 3.6 ppm.

***N*-(4-(*Tert*-butyl)phenyl)-*N*-methyl-3,4-dihydro-2*H*-pyran-6-carboxamide (21a)**

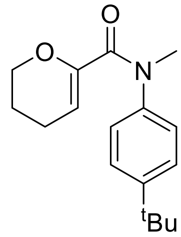

Prepared following **General Procedure B** using 4-(*tert*-butyl)-*N*-methylaniline (**P5**) (269 mg, 1.65 mmol, 1.10 equiv.) and 3,4-dihydro-2*H*-pyran-6-carboxylic acid (**P1**) (192 mg, 1.50 mmol, 1.00 equiv.). Flash chromatography (1:4→3:7, EtOAc: pentane) afforded *N*-(4-(*tert*-butyl)phenyl)-*N*-methyl-3,4-dihydro-2*H*-pyran-6-carboxamide (**21a**) (259 mg, 63%) as a colourless oil.

IR (film) ν_{max}/cm^{-1}: 2961, 1644, 1511, 1383, 1371, 1343, 1284, 1059, 920.

^{1}H NMR (400 MHz, $CDCl_3$) δ = 7.37–7.30 (m, 2H), 7.14–7.04 (m, 2H), 5.37 (t, *J* = 4.0 Hz, 1H), 3.52–3.43 (m, 2H), 3.31 (s, 3H), 2.02–1.93 (m, 2H), 1.73–1.58 (m, 2H), 1.31 (s, 9H).

^{13}C NMR (101 MHz, $CDCl_3$) δ = 166.2, 149.6, 148.7, 142.3, 125.7, 125.2, 106.8, 65.7, 38.0, 34.6, 31.5, 21.7, 20.2.

HRMS (ESI$^+$) $C_{17}H_{24}NO_2$ [M+H]$^+$ requires 274.1802; found 274.1802, Δ 0.2 ppm.

N -(4-Methoxyphenyl)-N-methyl-3,4-dihydro-2H-pyran-6-carboxamide (22a)

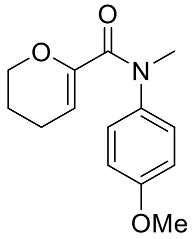

Prepared following **General Procedure B** using 4-methoxy-*N*-methylaniline (206 mg, 1.5 mmol, 1.0 equiv.) and 3,4-dihydro-2H-pyran-6-carboxylic acid (**P1**) (211 mg, 1.65 mmol, 1.1 equiv.). Flash column chromatography (1:4, EtOAc,pentane) afforded *N* -(4-methoxyphenyl)-*N*-methyl-3,4-dihydro-2*H*-pyran-6-carboxamide (**22a**) (280 mg, 75%) as a brown oil.

IR (film) ν_{max}/cm^{-1}: 1640, 1511, 1464, 1445, 1385, 1373, 1344, 1286, 1246, 1169, 1104, 1059, 1033, 920, 836, 749.

^{1}H NMR (400 MHz, $CDCl_3$) δ = 7.14 – 6.97 (m, 2H), 6.90 – 6.76 (m, 2H), 5.31 (t, *J* = 4.0 Hz, 1H), 3.79 (s, 3H), 3.52 (t, *J* = 5.1 Hz, 2H), 3.26 (s, 3H), 1.96 (td, *J* = 6.4, 4.0 Hz, 2H), 1.61 (q, *J* = 6.2, 5.8 Hz, 2H).

^{13}C NMR (101 MHz, $CDCl_3$) δ = 166.1, 158.0, 148.6, 137.8, 126.9, 114.0, 106.4, 65.7, 55.4, 38.1, 21.6, 20.0.

HRMS (ESI^+) $C_{14}H_{18}NO_3$ $[M+H]^+$ requires 248.1281; found 248.1281, Δ -0.2 ppm.

N-Methyl-N-(4-(methylthio)phenyl)-3,4-dihydro-2H-pyran-6-carboxamide (23a)

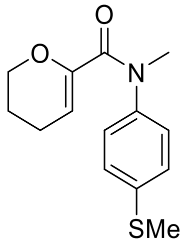

Prepared following **General Procedure B** using *N*-methyl-4-(methylthio)aniline (**P6**) (252 mg, 1.65 mmol, 1.10 equiv.) and 3,4-dihydro-2*H*-pyran-6-carboxylic acid (**P1**) (192 mg, 1.5 mmol, 1.00 equiv.). Flash chromatography (1:4→3:7, EtOAc: pentane) afforded *N*-methyl-*N*-(4-(methylthio)phenyl)-3,4-dihydro-2*H*-pyran-6-carboxamide (**23a**) (88.8 mg, 22%) as a colourless oil.

IR (film) ν_{max}/cm^{-1}: 2925, 1638, 1493, 1369, 1105, 1056, 919.

^{1}H NMR (400 MHz, $CDCl_3$) δ = 7.23 – 7.19 (m, 2H), 7.12 – 7.05 (m, 2H), 5.39 (t, *J* = 4.0 Hz, 1H), 3.56 – 3.51 (m, 2H), 3.29 (s, 3H), 2.48 (s, 3H), 2.03 – 1.97 (m, 2H), 1.69 – 1.62 (m, 2H).

^{13}C NMR (101 MHz, $CDCl_3$) δ = 166.0, 148.5, 142.3, 136.7, 127.1, 126.1, 107.1, 65.9, 38.1, 21.7, 20.2, 16.2.

HRMS (ESI^+) $C_{14}H_{17}O_2NS$ $[M+H]^+$ requires 264.1053; found 264.1053, Δ 0.2 ppm.

***N*-Methyl-*N*-phenyl-4,5-dihydrofuran-2-carboxamide (24a)**

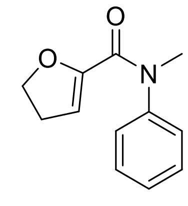

Prepared following **General Procedure B** using *N*-methylaniline (235 mg, 2.20 mmol, 1.10 equiv.) and 4,5-dihydrofuran-2-carboxylic acid (**P2**) (228, mg, 2.00 mmol, 1.00 equiv.). Flash column chromatography (3:7, EtOAc: pentane) afforded *N*-methyl-*N*-phenyl-4,5-dihydrofuran-2-carboxamide (**24a**) (208 mg, 51%) as an off-white solid.

m.p. = 48-50°C (EtOAc-pentane).

IR (film) ν_{max}/cm^{-1}: 2980, 2971, 1786, 1647, 1593, 1495, 1381, 932, 697.

^{1}H NMR (400 MHz, $CDCl_3$) δ = 7.40–7.33 (m, 2H), 7.31–7.25 (m, 1H), 7.22–7.15 (m, 2H), 5.08–4.95 (m, 1H), 4.14 (t, *J* = 9.6 Hz, 2H), 3.36 (s, 3H), 2.54 (td, *J* = 9.7, 2.9 Hz, 2H).

^{13}C NMR (101 MHz, $CDCl_3$) δ = 162.0, 150.9, 144.3, 129.2, 127.3, 126.4, 107.3, 70.1, 38.1, 30.4.

HRMS (ESI$^+$) $C_{12}H_{13}NO_2$ [M+H]$^+$ requires 204.1019; found 204.1021, Δ 0.8 ppm.

***N*-Methyl-*N*-(phenyl-*d*$_5$)-3,4-dihydro-2*H*-pyran-6-carboxamide (28)**

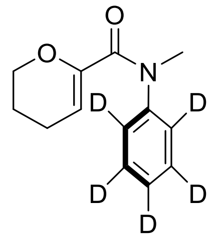

Prepared following **General Procedure B** using *N*-methylbenzen-*d*$_5$-amine (**P7**) (224 mg, 2.00 mmol, 1.00 equiv.) and 3,4-dihydro-2H-pyran-6-carboxylic acid (228 mg, 2.00 mmol, 1.00 equiv.). Flash column chromatography (1:4, EtOAc: pentane) afforded *N*-Methyl-*N*-(phenyl-*d*5)-3,4-dihydro-2*H*-pyran-6-carboxamide (**28**) (217 mg, 66%) as a white solid.

m.p. = 56-58 °C (EtOAc-pentane).

IR (film) ν_{max}/cm^{-1}: 2937, 1641, 1400, 1365, 1321, 1083, 1071, 1053, 919, 740.

^{1}H NMR (600 MHz, $CDCl_3$) δ = 5.38 (t, *J* = 4.0 Hz, 1H), 3.51–3.46 (m, 2H), 3.32 (s, 3H), 2.01–1.95 (m, 2H), 1.65–1.60 (m, 2H).

^{13}C NMR (151 MHz, $CDCl_3$) δ = 166.1, 148.6, 144.9, 128.4 (t, *J* = 24.4 Hz, I = 1), 126.0 (t, *J* = 24.5 Hz, I = 1), 125.3 (t, *J* = 24.4 Hz, I = 1), 107.0, 65.8, 38.0, 21.7, 20.2.

HRMS (ESI$^+$) $C_{13}H_{10}D_5NO_2$ [M+H]$^+$ requires 223.1489; found 223.1490, Δ 0.4 ppm.

***N*-Ethyl-*N*-phenyl-3,4-dihydro-2*H*-pyran-6-carboxamide (S1a)**

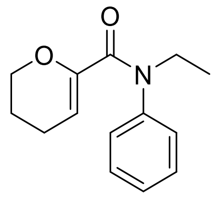

Prepared following **General Procedure B** using *N*-ethylaniline (244 mg, 2.20 mmol, 1.10 equiv.) and 3,4-dihydro-2H-pyran-6-carboxylic acid (**P1**) (256 mg, 2.00 mmol, 1.00 equiv.). Flash column chromatography (3:7, EtOAc: pentane) afforded *N*-ethyl-*N*-phenyl-3,4-dihydro-2H-pyran-6-carboxamide (**S1a**) (284 mg, 61%) as a white solid.

m.p. = 84-86 °C (EtOAc-pentane).

IR (film) ν_{max}/cm^{-1}: 2977, 2958, 2931, 2874, 1629, 1493, 1402, 1305, 1282, 768, 747, 700.

^{1}H NMR (400 MHz, $CDCl_3$) δ = 7.37–7.30 (m, 2H), 7.25–7.20 (m, 1H), 7.17–7.10 (m, 2H), 5.36 (t, *J* = 4.0 Hz, 1H), 3.80 (q, *J* = 7.1 Hz, 2H), 3.51–3.37 (m, 2H), 1.99–1.94(m, 2H), 1.65–1.58 (m, 2H), 1.13 (t, *J* = 7.1 Hz, 4H).

^{13}C NMR (101 MHz, $CDCl_3$) δ = 165.6, 148.8, 143.4, 128.9, 126.8, 126.7, 106.9, 65.7, 45.1, 21.7, 20.2, 13.0.

HRMS (ESI$^+$) $C_{14}H_{18}NO_2$ $[M+H]^+$ requires 232.1332; found 232.1333, Δ 0.4ppm.

***N*-(4-Chlorophenyl)-*N*-methyl-3,4-dihydro-2*H*-pyran-6-carboxamide (S2a)**

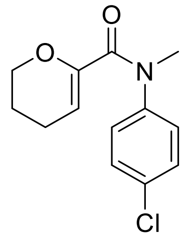

Prepared following **General Procedure B** using 4-chloro-*N*-methylaniline (234 mg, 1.65 mmol, 1.10 equiv.) and 3,4-dihydro-2H-pyran-6-carboxylic acid (**P1**) (192 mg, 1.5 mmol, 1.00 equiv.). Flash chromatography (1:4→3:7, EtOAc: pentane) afforded *N*-(4-chlorophenyl)-*N*-methyl-3,4-dihydro-2*H*-pyran-6-carboxamide (**S2a**) (184 mg, 49%) as a white solid.

m.p. = 50-52 °C (EtOAc/pentane).

IR (film) ν_{max}/cm^{-1}: 3004, 1654, 1634, 1491, 1364, 1282, 1103, 1087, 1053, 1013, 918, 837, 748.

^{1}H NMR (400 MHz, $CDCl_3$) δ = 7.32–7.28 (m, 2H), 7.13–7.07 (m, 2H), 5.45 (t, *J* = 4.0 Hz, 1H), 3.59–3.48 (m, 2H), 3.29 (s, 3H), 2.10–1.94 (m, 2H), 1.73–1.60 (m, 3H).

^{13}C NMR (101 MHz, $CDCl_3$) δ = 165.9, 148.3, 143.7, 132.1, 129.1, 127.0, 107.6, 65.9, 38.1, 21.7, 20.2.

HRMS (ESI$^+$) $C_{13}H_{14}NO_2Cl$ $[M+H]^+$ requires 252.0786; found 252.0784, Δ 0.7 ppm.

***N*-Methyl-*N*-phenyl-4,5,6,7-tetrahydrooxepine-2-carboxamide (S3a)**

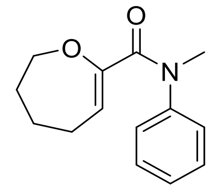

To a solution of 2,4,6-trichlorophenyl 4,5,6,7-tetrahydrooxepine-2-carboxylate (**P8**) (375 mg, 1.2 mmol, 1.0 equiv.) in THF (3 mL) was added Et_3N (280 µl, 2.4 mmol, 2.0 equiv.), DMAP (13 mg, 0.1 mmol, 0.1 equiv.), and *N*-methylaniline (650 µl, 6.0 mmol, 5.0 equiv.). The reaction was then warmed to 60 °C and allowed to stir for 72 h. Upon complete consumption of starting material, the mixture was allowed to cool and directly purified by flash column chromatography (1:4, EtOAc: pentane) to afford *N*-methyl-*N*-phenyl-4,5,6,7-tetrahydrooxepine-2-carboxamide (**S3a**) as a viscous yellow oil (257 mg, 93%).

IR (film) ν_{max}/cm^{-1}: 1624, 1596, 1586, 1512, 1495, 1430, 1399, 1320, 1304, 1294, 1276, 1244, 1230, 1186, 1135, 1070, 1059, 1026, 923, 820, 802, 756, 739, 694, 623.

^{1}H NMR (600 MHz, $CDCl_3$) δ = 7.40 – 7.33 (m, 2H), 7.27 – 7.21 (m, 1H), 7.20 – 7.14 (m, 2H), 5.77 (t, *J* = 5.8 Hz, 1H), 3.29 (s, 3H), 3.05 (t, *J* = 5.3 Hz, 2H), 2.17 – 2.11 (m, 2H), 1.59 (d, *J* = 6.2 Hz, 2H), 1.54 – 1.47 (m, 2H).

^{13}C NMR (151 MHz, $CDCl_3$) δ = 167.2, 153.1, 145.3, 129.1, 126.7, 126.1, 116.9, 72.1, 38.6, 30.7, 26.4, 25.1.

HRMS (ESI$^+$): $C_{14}H_{18}NO_2$ $[M+H]^+$ requires 232.1332; found 232.1332, Δ 0.0 ppm.

3.5. Photocyclization Products

(4a*R*,10b*S*)-6-Methyl-2,3,6,10b-tetrahydro-1*H*-pyrano[2,3-*c*]quinolin-5(4a*H*)-one (2)

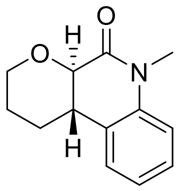

Prepared from *N*-methyl-*N*-phenyl-3,4-dihydro-2*H*-pyran-6-carboxamide (**2a**) (21.7 mg, 0.100 mmol) following **General Procedure C**. Purification by flash column chromatography (3:2, EtOAc: pentane) afforded (4a*R*,10b*S*)-6-methyl-2,3,6,10b-tetrahydro-1*H*-pyrano[2,3-c]quinolin-5(4a*H*)-one (**2**) (21.6 mg, 95%, >20:1 dr, 94:6 er) as an off-white solid.

- or –

Prepared from *N*-methyl-*N*-phenyl-3,4-dihydro-2*H*-pyran-6-carboxamide (**2a**) (435 mg, 2.0 mmol) following **General Procedure C,** but with 20 mL of preformed catalyst solution (5 mol%) and 4.0 mg (0.025 mol%) **4** in a 30 mL vial. Purification by flash column chromatography (3:2, EtOAc: pentane) afforded (4a*R*,10b*S*)-6-methyl-2,3,6,10b-tetrahydro-1*H*-pyrano[2,3-c]quinolin-5(4a*H*)-one (**2**) (288 mg, 66% (86% brsm), >20:1 dr, 80:20 er) as an off-white solid.

m.p. = 78-80 °C

IR (film) ν_{max}/cm^{-1}: 2935, 2920, 2849, 1688, 1602, 1459, 1380, 1253, 1110.

^{1}H NMR (400 MHz, $CDCl_3$) δ = 7.33 – 7.28 (m, 1H), 7.22 (d, *J* = 7.7 Hz, 1H), 7.10 (ddd, *J* = 7.5, 7.5, 1.2 Hz, 1H), 7.00 (d, *J* = 1.2 Hz, 1H), 4.32 – 4.22 (m, 1H), 3.71 (d, *J* = 13.1 Hz, 1H), 3.50 (ddd, *J* = 11.9, 11.8, 2.7 Hz, 1H), 3.40 (s, 3H), 2.97 – 2.75 (m, 1H), 2.61 – 2.47 (m, 1H), 1.95 – 1.73 (m, 2H), 1.70 – 1.54 (m, 1H).

^{13}C NMR (101 MHz, $CDCl_3$) δ = 168.3, 139.3, 128.0, 127.2, 124.6, 123.4, 114.8, 76.0, 68.3, 36.5, 30.1, 26.2, 24.8.

HRMS (ESI$^+$): $C_{13}H_{15}NO_2$ $[M+H]^+$ requires 218.1176; found 218.1177, Δ 0.8 ppm.

Chiral HPLC: (Chiralpak OD-H, 30% ^{i}PrOH, 70% hexane, 1.0 mL min^{-1}, λ = 210 nm) τ_R (major) = 11.1 min, τ_R (minor) = 16.5 min; er 95:5.

$[\alpha]_D^{25}$ = +159.1 (*c* = 0.50, $CHCl_3$).

(4a*R*,10b*S*)-6-Benzyl-2,3,6,10b-tetrahydro-1*H*-pyrano[2,3-*c*]quinolin-5(4a*H*)-one (5)

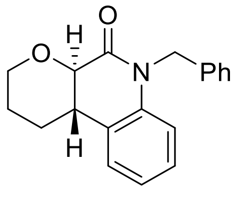

Prepared from *N*-benzyl-*N*-phenyl-3,4-dihydro-2*H*-pyran-6-carboxamide (**5a**) (29.3 mg, 0.100 mmol) following **General Procedure C**. Purification by flash column chromatography (7:3, EtOAc: pentane)

afforded (4a*R*,10b*S*)-6-benzyl-2,3,6,10b-tetrahydro-1*H*-pyrano[2,3-c]quinolin-5(4a*H*)-one (**5**) (28.3 mg, 97%, >20:1 dr, 93:7 er) as an off-white solid.

m.p. = 154-156 °C (EtOAc-pentane).

IR (film) ν_{max}/cm^{-1}: 2937, 2850, 1691, 1495, 1457, 1389, 1210, 1115, 754.

^{1}H NMR (400 MHz, $CDCl_3$) δ = 7.23–7.12 (m, 6H), 7.11–7.05 (m, 1H), 6.98 (td, *J* = 7.5, 1.2 Hz, 1H), 6.84 (dd, *J* = 8.1, 1.2 Hz, 1H), 5.34 (d, *J* = 16.1 Hz, 1H), 4.93 (d, *J* = 16.1 Hz, 1H), 4.29–4.19 (m, 1H), 3.83 (d, *J* = 13.2 Hz, 1H), 3.48 (ddd, *J* = 11.9, 11.8, 2.6 Hz, 1H), 2.94–2.82 (m, 1H), 2.54–2.42 (m, 1H), 1.90–1.69 (m, 2H), 1.65–1.50 (m, 1H).

^{13}C NMR (101 MHz, $CDCl_3$) δ = 168.7, 138.5, 136.9, 128.8, 128.1, 127.5, 127.3, 126.8, 124.8, 123.7, 115.8, 76.1, 68.4, 46.6, 36.7, 26.5, 24.9.

HRMS (ESI$^+$) $C_{19}H_{19}NO_2$ [M+H]$^+$ requires 294.1489; found 294.1488, 0.1 ppm.

Chiral HPLC: (Chiralpak OD-H, 30% ^{i}PrOH, 70% hexane, 1.0 mL min^{-1}, λ = 260 nm) τ_R (major) = 10.7 min, τ_R (minor) = 12.7 min; er 94:6.

$[\alpha]_D^{25}$ = +81.1 (c = 0.50, $CHCl_3$).

(4a*R*,10b*S*)-6-Isobutyl-2,3,6,10b-tetrahydro-1*H*-pyrano[2,3-*c*]quinolin-5(4a*H*)-one (6)

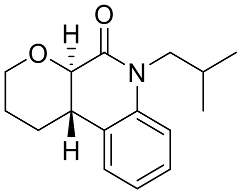

Prepared from *N*-isobutyl-*N*-phenyl-3,4-dihydro-2*H*-pyran-6-carboxamide (**6a**) (29.3 mg, 0.100 mmol) following **General Procedure C**. Purification by flash column chromatography (1:1, EtOAc: pentane) afforded (4a*R*,10b*S*)-6-isobutyl-2,3,6,10b-tetrahydro-1*H*-pyrano[2,3-c]quinolin-5(4a*H*)-one (**6**) (23.7 mg, 92%, >20:1 dr, 93:7 er) as a colourless oil.

IR (film) ν_{max}/cm^{-1}: 2980, 1691, 1458, 1386, 1145, 1114.

^{1}H NMR (400 MHz, $CDCl_3$) δ = 7.25–7.19 (m, 1H), 7.15 (d, *J* = 7.7 Hz, 1H), 7.02 (ddd, *J* = 7.5, 7.5, 1.2 Hz, 1H), 6.95 (d, *J* = 1.1 Hz, 1H), 4.25–4.17 (m, 1H), 4.08 (dd, *J* = 14.1, 9.5 Hz, 1H), 3.66 (d, *J* = 13.2 Hz, 1H), 3.53 (dd, *J* = 14.2, 5.7 Hz, 1H), 3.43 (ddd, *J* = 12.0, 11.9, 2.5 Hz, 1H), 2.85–2.72 (m, 1H), 2.51–2.40 (m, 1H), 2.03–1.89 (m, 1H), 1.89–1.66 (m, 2H), 1.61–1.45 (m, 1H), 0.88 (d, *J* = 6.8 Hz, 3H), 0.81 (d, *J* = 6.6 Hz, 3H).

^{13}C NMR (101 MHz, $CDCl_3$) δ = 168.7, 138.2, 128.0, 127.8, 125.0, 123.4, 115.6, 76.0, 68.3, 48.5, 36.5, 26.5, 26.2, 24.9, 20.3, 19.7.

HRMS (ESI$^+$) $C_{16}H_{21}NO_2$ [M+H]$^+$ requires 260.1645; found 260.1645, Δ 0.0 ppm.

Chiral HPLC: (Chiralpak OD-H, 30% ^{i}PrOH, 70% hexane, 1.0 mL min^{-1}, λ = 260 nm) τ_R (major) = 6.0 min, τ_R (minor) = 10.1 min; er 93:7.

$[\alpha]_D^{25}$ = +103.6 (*c* = 1.00, $CHCl_3$).

(4a*R*,10b*S*)-6-Propyl-2,3,6,10b-tetrahydro-1*H*-pyrano[2,3-*c*]quinolin-5(4a*H*)-one (7)

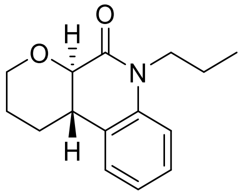

Prepared from *N*-phenyl-*N*-propyl-3,4-dihydro-2*H*-pyran-6-carboxamide (**7a**) (24.5 mg, 0.100 mmol) following **General Procedure C**. Purification by flash column chromatography (2:3, EtOAc: pentane) afforded (4a*R*,10b*S*)-6-propyl-2,3,6,10b-tetrahydro-1*H*-pyrano[2,3-c]quinolin-5(4a*H*)-one (**7**) (20.9 mg, 85%, >20:1 dr, 95:5 er) as an off-white solid.

m.p. = 69-73 °C

IR (film) ν_{max}/cm^{-1}: 1691, 1602, 1496, 1458, 1389, 1305, 1252, 1230, 1215, 1145, 1115, 1090, 963, 755, 668.

^{1}H NMR (400 MHz, $CDCl_3$) δ = 7.29 (ddt, *J* = 8.4, 7.3, 1.3 Hz, 1H), 7.22 (dt, *J* = 7.6, 1.5 Hz, 1H), 7.09 (td, *J* = 7.5, 1.1 Hz, 1H), 7.01 (dd, *J* = 8.3, 1.1 Hz, 1H), 4.32 – 4.23 (m, 1H), 4.05 – 3.84 (m, 2H), 3.72 (d, *J* = 13.2 Hz, 1H), 3.50 (td, *J* = 11.8, 2.6 Hz, 1H), 2.92 – 2.80 (m, 1H), 2.53 (dp, *J* = 12.8, 4.4, 3.0 Hz, 1H), 1.97 – 1.49 (m, 5H), 0.94 (t, *J* = 7.4 Hz, 3H).

^{13}C NMR (101 MHz, $CDCl_3$) δ = 168.1, 138.2, 127.9, 127.6, 124.8, 123.3, 115.1, 75.9, 68.2, 44.0, 36.5, 26.3, 24.8, 20.4, 11.1.

HRMS (ESI$^+$): $C_{15}H_{20}NO_2$ [M+H]$^+$ requires 246.1489; found 246.1488, Δ -0.4 ppm.

Chiral HPLC: (Chiralpak OD-H, 30% ^{i}PrOH, 70% hexane, 1.0 mL min^{-1}, λ = 210 nm) τ_R (major) = 7.1 min, τ_R (minor) = 12.0 min; er 95:5.

$[\alpha]_D^{25}$ = +161.5 (*c* = 0.10, $CHCl_3$).

(4a*R*,10b*S*)-6-Allyl-2,3,6,10b-tetrahydro-1*H*-pyrano[2,3-*c*]quinolin-5(4a*H*)-one (8)

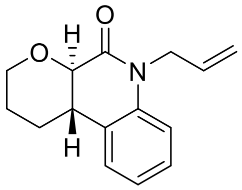

Prepared from *N*-allyl-*N*-phenyl-3,4-dihydro-2*H*-pyran-6-carboxamide (**8a**) (24.3 mg, 0.100 mmol) following **General Procedure C**. Purification by flash column chromatography (1:1, EtOAc: pentane) afforded (4a*R*,10b*S*)-6-allyl-2,3,6,10b-tetrahydro-1*H*-pyrano[2,3-c]quinolin-5(4a*H*)-one (**8**) (17.5 mg, 72%, >20:1 dr, 91:9 er) as an off-white solid.

m.p. = 80-82 °C (EtOAc-pentane).

IR (film) ν_{max}/cm^{-1}: 2980, 1691, 1386, 1211.

^{1}H NMR (400 MHz, $CDCl_3$) δ = 7.29 – 7.18 (m, 2H), 7.09 (ddd, *J* = 1.2, 7.5 Hz, 1H), 7.01 (dd, *J* = 1.1, 8.1 Hz, 1H), 5.87 (ddt, *J* = 4.9, 10.1, 17.2 Hz, 1H), 5.22 – 5.10 (m, 2H), 4.87 – 4.77 (m, 1H), 4.38 – 4.31 (m,

1H), 4.31 – 4.24 (m, 1H), 3.78 (d, J = 13.2 Hz, 1H), 3.51 (td, J = 2.7, 11.8 Hz, 1H), 3.10 – 2.78 (m, 1H), 2.63 – 2.42 (m, 1H), 1.94 – 1.73 (m, 2H), 1.70 – 1.52 (m, 1H).

^{13}C NMR (101 MHz, $CDCl_3$) δ = 168.1, 138.6, 132.6, 128.0, 127.4, 124.8, 123.6, 116.7, 115.6, 76.0, 68.4, 45.6, 36.7, 26.4, 24.9.

HRMS (ESI$^+$) $C_{15}H_{17}O_2N$ $[M+H]^+$ requires 244.1332; found 244.1333, Δ 0.2 ppm.

Chiral HPLC: (Chiralpak OD-H, 30% ^{i}PrOH, 70% hexane, 1.0 mL min^{-1}, λ = 210 nm) τ_R (major) = 9.1 min, τ_R (minor) = 12.0 min; er 93:7.

$[\alpha]_D^{25}$ = +138.4 (c = 1.00, $CHCl_3$).

(8a*R*,12a*S*)-5,6,10,11,12,12a-Hexahydro-4*H*-pyrano[2,3-*c*]pyrido[3,2,1-*ij*]quinolin-8(8a*H*)-one (9)

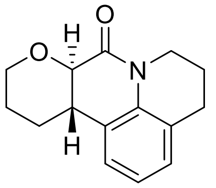

Prepared from (3,4-dihydro-2*H*-pyran-6-yl)(3,4-dihydroquinolin-1(2*H*)-yl)methanone (**9a**) (24.3 mg, 0.100 mmol) following **General Procedure C**. Purification by flash column chromatography (1:4→3:1, EtOAc: pentane) afforded (8a*R*,12a*S*)-5,6,10,11,12,12a-hexahydro-4*H*-pyrano[2,3-c]pyrido[3,2,1-ij]quinolin-8(8a*H*)-one (**9**) (12.7 mg, 52%, >20:1 dr, 82:18 er) as an off-white solid and recovered starting material (9.9 mg, 41%)

m.p. = 134-136 °C (EtOAc-pentane).

IR (film) ν_{max}/cm^{-1}: 2935, 2853, 2849, 1684, 1390, 1246, 1111.

^{1}H NMR (400 MHz, $CDCl_3$) δ = 7.08–6.95 (m, 3H), 4.37–4.21 (m, 2H), 3.74 (d, J = 13.3 Hz, 1H), 3.55–3.45 (m, 2H), 2.89–2.68 (m, 3H), 2.51 (ddt, J = 12.7, 5.6, 3.1 Hz, 1H), 2.03–1.73 (m, 4H), 1.67–1.53 (obs. m, 1H).

^{13}C NMR (101 MHz, $CDCl_3$) δ = 167.7, 134.9, 128.5, 126.7, 125.5, 123.0, 122.6, 76.2, 68.4, 41.3, 36.7, 27.4, 26.4, 25.1, 21.5.

HRMS (ESI$^+$) $C_{15}H_{17}NO_2$ $[M+Na]^+$ requires 266.1152; found 266.1153, 0.4 ppm.

Chiral HPLC: (Chiralpak OD-H, 30% ^{i}PrOH, 70% hexane, 1.0 mL min^{-1}, λ = 260 nm) τ_R (major) = 13.2 min, τ_R (minor) = 15.7 min; er 82:18.

$[\alpha]_D^{25}$ = +87.2 (c = 0.50, $CHCl_3$).

(4a*R*,10b*S*)-6-(4-Methoxybenzyl)-2,3,6,10b-tetrahydro-1*H*-pyrano[2,3-*c*]quinolin-5(4a*H*)-one (10)

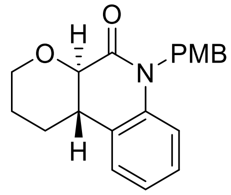

Prepared from *N*-(4-methoxybenzyl)-*N*-phenyl-3,4-dihydro-2*H*-pyran-6-carboxamide (**10a**) (32.3 mg, 0.100 mmol) following **General Procedure C**. Purification by flash column chromatography (3:7, EtOAc: pentane) afforded (4a*R*,10b*S*)-6-(4-methoxybenzyl)-2,3,6,10b-tetrahydro-1H-pyrano[2,3-*c*]quinolin-5(4a*H*)-one (**10**) (31.1 mg, 96%, >20:1 dr, 93:7 er) as an off-white solid.

m.p. = 138-140 °C

IR (film) ν_{max}/cm^{-1}: 1688, 1603, 1513, 1496, 1460, 1391, 1304, 1249, 1212, 1178, 1114, 1034, 754.

^{1}H NMR (400 MHz, $CDCl_3$) δ = 7.23 – 7.12 (m, 4H), 7.05 (td, *J* = 7.5, 1.2 Hz, 1H), 6.96 (dd, *J* = 8.2, 1.2 Hz, 1H), 6.85 – 6.76 (m, 2H), 5.30 (d, *J* = 15.8 Hz, 1H), 4.98 (d, *J* = 15.8 Hz, 1H), 4.31 (dd, *J* = 11.6, 4.4 Hz, 1H), 3.87 (d, *J* = 13.2 Hz, 1H), 3.75 (s, 3H), 3.54 (td, *J* = 11.8, 2.6 Hz, 1H), 2.99 – 2.87 (m, 1H), 2.54 (dd, *J* = 12.8, 3.8 Hz, 1H), 1.98 – 1.81 (m, 1H), 1.63 (tdd, *J* = 12.8, 11.4, 4.2 Hz, 1H).

^{13}C NMR (101 MHz, $CDCl_3$) δ = 168.7, 158.8, 138.4, 128.9, 128.1, 127.9, 127.4, 124.7, 123.5, 115.8, 114.1, 76.0, 68.3, 55.2, 45.9, 36.5, 26.3, 24.8.

HRMS (ESI$^+$): $C_{20}H_{22}NO_3$ $[M+H]^+$ requires 324.1594; found 324.1594, Δ -0.2 ppm.

Chiral HPLC: (Chiralpak OD-H, 30% ^{i}PrOH, 70% hexane, 1.0 mL min^{-1}, λ = 210 nm) τ_R (major) = 13.0 min, τ_R (minor) = 15.8 min; er 93:7.

$[\alpha]_D^{25}$ = +98.9 (*c* = 0.10, $CHCl_3$).

(4a*R*,10b*S*)-8-Fluoro-6-methyl-2,3,6,10b-tetrahydro-1*H*-pyrano[2,3-*c*]quinolin-5(4a*H*)-one (11), and (4a*R*,10b*S*)-10-Fluoro-6-methyl-2,3,6,10b-tetrahydro-1*H*-pyrano[2,3-*c*]quinolin-5(4a*H*)-one (11b)

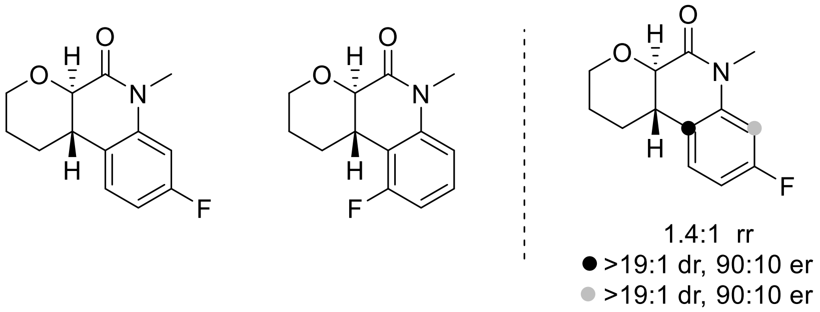

Prepared from *N*-(3-fluorophenyl)-*N*-methyl-3,4-dihydro-2H-pyran-6-carboxamide (**11a**) (23.5 mg, 0.100 mmol) following **General Procedure C**. Purification by flash column chromatography (3:7→1:1, EtOAc: pentane) afforded an inseparable mixture of (4a*R*,10b*S*)-8-fluoro-6-methyl-2,3,6,10b-tetrahydro-1*H*-pyrano[2,3-*c*]quinolin-5(4a*H*)-one (**11**) (>20:1 dr, 90:10 er) and (4a*R*,10b*S*)-10-fluoro-6-methyl-2,3,6,10b-tetrahydro-1*H*-pyrano[2,3-*c*]quinolin-5(4a*H*)-one (**11b**) (>20:1 dr, 90:10 er) (1.4:1 rr) as an off-white solid. The yield refers to the combined masses of the two products (8.3 mg, 35%). The diastereomeric ratio and regioisomeric ratio was determined by quantitative 1H NMR analysis of the crude reaction mixture.

(4a*R*,10b*S*)-8-Fluoro-6-methyl-2,3,6,10b-tetrahydro-1*H*-pyrano[2,3-*c*]quinolin-5(4a*H*)-one (11), and (4a*R*,10b*S*)-10-Fluoro-6-methyl-2,3,6,10b-tetrahydro-1*H*-pyrano[2,3-*c*]quinolin-5(4a*H*)-one (11b)

m.p. = 96-104 °C

IR (film) ν_{max}/cm^{-1}: 1697, 1613, 1509, 1470, 1437, 1378, 1346, 1312, 1253, 1237, 1218, 1142, 1110, 1086, 1061, 1003, 965, 850, 770, 667.

(4a*R*,10b*S*)-8-Fluoro-6-methyl-2,3,6,10b-tetrahydro-1*H*-pyrano[2,3-*c*]quinolin-5(4a*H*)-one (11)

^{1}H NMR (600 MHz, $CDCl_3$) δ = 7.15 (ddd, *J* = 8.1, 6.2, 1.3 Hz, 1H), 6.82 – 6.75 (m, 1H), 6.72 (dd, *J* = 10.4, 2.5 Hz, 1H), 4.32 – 4.22 (m, 1H), 3.69 (d, *J* = 13.1 Hz, 1H), 3.50 (td, *J* = 12.0, 2.6 Hz, 1H), 3.37 (s, 3H), 2.83 (tdd, *J* = 13.1, 4.0, 1.9 Hz, 1H), 2.53 – 2.47 (m, 1H), 1.91 – 1.75 (m, 2H), 1.65 – 1.55 (m, 1H).

^{19}F NMR (376 MHz, $CDCl_3$) δ = -113.06 – -113.22 (m).

^{13}C NMR (151 MHz, $CDCl_3$) δ = 168.3, 162.5 (d, *J* = 244.7 Hz), 140.7 (d, *J* = 10.1 Hz), 125.8 (d, *J* = 9.5 Hz), 122.7 (d, *J* = 3.2 Hz), 111.8 (d, *J* = 25.0 Hz), 109.5 (d, *J* = 21.1 Hz), 76.0, 68.3, 36.1, 30.1, 26.4, 24.7.

Chiral HPLC: (Chiralpak IB N-3, 25% ^{i}PrOH, 75% hexane, 1.0 mL min^{-1}, λ = 210 nm) τ_R (major) = 17.0 min, τ_R (minor) = 22.3 min; er 90:10.

(4a*R*,10b*S*)-10-Fluoro-6-methyl-2,3,6,10b-tetrahydro-1*H*-pyrano[2,3-*c*]quinolin-5(4a*H*)-one (11b)

^{1}H NMR (600 MHz, $CDCl_3$) δ = 7.26 – 7.20 (m, 1H), 6.82 – 6.75 (m, 2H), 4.32 – 4.22 (m, 1H), 3.81 (d, *J* = 12.8 Hz, 1H), 3.58 – 3.53 (m, 1H), 3.38 (s, 3H), 3.04 (td, *J* = 12.2, 4.1 Hz, 1H), 2.94 – 2.88 (m, 1H), 1.91 – 1.81 (m, 2H), 1.73 (ddt, *J* = 11.5, 3.8, 1.9 Hz, 1H).

^{19}F NMR (376 MHz, $CDCl_3$) δ = -113.60 (dt, *J* = 12.2, 6.4 Hz).

^{13}C NMR (151 MHz, $CDCl_3$) δ = 168.0, 161.5 (d, *J* = 247.4 Hz), 141.2 (d, *J* = 8.2 Hz), 128.8 (d, *J* = 10.8 Hz), 114.1 (d, *J* = 15.3 Hz), 111.1 (d, *J* = 2.8 Hz), 102.9 (d, *J* = 26.6 Hz), 75.6, 68.5, 36.5, 30.9, 27.4 (d, *J* = 13.2 Hz), 25.1 (d, *J* = 3.1 Hz).

Chiral HPLC: (Chiralpak IB N-3, 25% ^{i}PrOH, 75% hexane, 1.0 mL min^{-1}, λ = 210 nm) τ_R (major) = 15.3 min, τ_R (minor) = 18.8 min; er 90:10.

HRMS (ESI^{+}): $C_{13}H_{15}NO_2F$ $[M+H]^+$ requires 236.1081; found 236.1081, Δ -0.2 ppm.

$[\alpha]_D^{25}$ = +149.4 (*c* = 0.10, $CHCl_3$).

(4a*R*,10b*S*)-8-Chloro-6-methyl-2,3,6,10b-tetrahydro-1*H*-pyrano[2,3-*c*]quinolin-5(4a*H*)-one (12) (4a*R*,10b*S*)-10-chloro-6-methyl-2,3,6,10b-tetrahydro-1*H*-pyrano[2,3-*c*]quinolin-5(4a*H*)-one (12b), and (4a*S*,10b*S*)-10-chloro-6-methyl-2,3,6,10b-tetrahydro-1*H*-pyrano[2,3-*c*]quinolin-5(4a*H*)-one (12b')

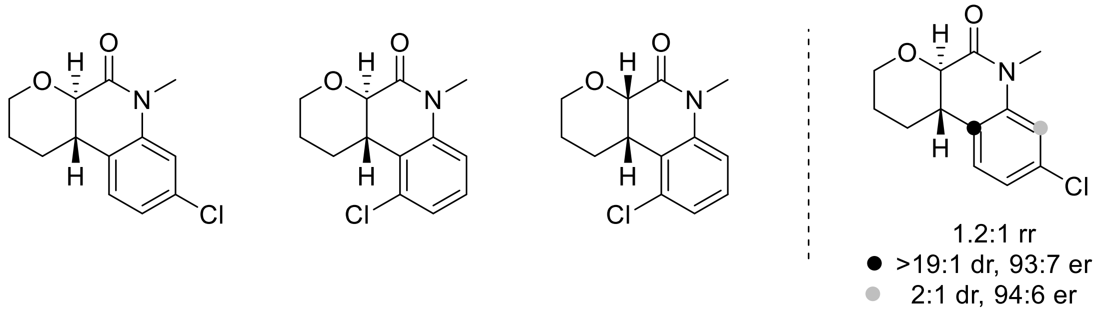

Prepared from *N*-(3-chlorophenyl)-*N*-methyl-3,4-dihydro-2*H*-pyran-6-carboxamide (**12a**) (25.2 mg, 0.100 mmol) following **General Procedure C**. Purification by flash column chromatography (1:4→1:1, EtOAc: pentane) afforded an inseparable mixture of (4a*R*,10b*S*)-8-chloro-6-methyl-2,3,6,10b-tetrahydro-1*H*-pyrano[2,3-c]quinolin-5(4a*H*)-one (**12**) (93:7 er) and (4a*R*,10b*S*)-10-chloro-6-methyl-2,3,6,10b-tetrahydro-1*H*-pyrano[2,3-c]quinolin-5(4a*H*)-one (**12b**) (94:6 er) (16.8 mg) as an off white solid and (4a*S*,10b*S*)-10-chloro-6-methyl-2,3,6,10b-tetrahydro-1*H*-pyrano[2,3-c]quinolin-5(4a*H*)-one (**12b'**) (3.5 mg, 93:7 er) as an off-white solid. The yield refers to the combined masses of the three products (20.3 mg, 81%). The diastereomeric ratio and regiosomeric ratio was determined by quantitative ^{1}H NMR analysis of the crude reaction mixture.

(4a*R*,10b*S*)-8-Chloro-6-methyl-2,3,6,10b-tetrahydro-1*H*-pyrano[2,3-*c*]quinolin-5(4a*H*)-one (12) and (4a*R*,10b*S*)-10-chloro-6-methyl-2,3,6,10b-tetrahydro-1*H*-pyrano[2,3-c]quinolin-5(4a*H*)-one (12b)

m.p. = 118-120 °C (EtOAc-pentane).

IR (film) ν_{max}/cm^{-1}: 2980, 1695, 1597, 1376, 1248, 1111, 1088.

(4a*R*,10b*S*)-8-Chloro-6-methyl-2,3,6,10b-tetrahydro-1*H*-pyrano[2,3-*c*]quinolin-5(4a*H*)-one (12)

^{1}H NMR (600 MHz, $CDCl_3$) δ = 7.12 (dd, *J* = 1.3, 8.2 Hz, 1H), 7.07 (d, *J* = 8.1 Hz, 1H), 6.98 (d, *J* = 2.0 Hz, 1H), 4.27 (ddt, *J* = 11.5, 4.6, 1.7 Hz, 1H), 3.68 (d, *J* = 13.1 Hz, 1H), 3.54 – 3.45 (m, 2H), 3.37 (s, 3H), 2.88 – 2.76 (m, 1H), 2.52 – 2.44 (m, 1H), 1.91 – 1.81 (m, 1H), 1.62 – 1.54 (m, 1H).

^{13}C NMR (101 MHz, $CDCl_3$) δ = 168.3, 140.5, 133.9, 125.9, 125.7, 123.3, 115.3, 75.9, 68.4, 36.3, 30.2, 26.3, 24.8.

Chiral HPLC: (Chiralpak OD-H, 30% ^{i}PrOH, 70% hexane, 1.0 mL min^{-1}, λ = 260 nm) τ_R (major) = 13.5 min, τ_R (minor) = 20.0 min; er 93:7.

(4a*R*,10b*S*)-10-Chloro-6-methyl-2,3,6,10b-tetrahydro-1*H*-pyrano[2,3-*c*]quinolin-5(4a*H*)-one (12b)

^{1}H NMR (600 MHz, $CDCl_3$) δ = 7.18 (td, *J* = 8.1, 0.9 Hz, 1H), 7.07 (d, *J* = 8.2 Hz, 1H), 6.91 (dd, *J* = 8.1, 1.2 Hz, 1H), 4.24 – 4.20 (m, 1H), 3.81 (d, *J* = 12.6 Hz, 1H), 3.48 – 3.41 (m, 1H), 3.37 (s, 3H), 3.12 (ddd, 1H), 1.82 – 1.74 (m, 4H).

^{13}C NMR (101 MHz, $CDCl_3$) δ = 168.0, 142.0, 133.6, 128.4, 127.2, 124.8, 114.7, 75.5, 68.3, 39.0, 31.5, 28.2, 25.4.

Chiral HPLC: (Chiralpak OD-H, 30% ^{i}PrOH, 70% hexane, 1.0 mL min^{-1}, λ = 254 nm) τ_R (major) = 8.5 min, τ_R (minor) = 14.4 min; er 94:6.

HRMS (ESI$^+$) $C_{13}H_{14}O_2NCl$ [M+Na]$^+$ requires 274.0605; found 274.0606, Δ 0.4 ppm.

$[\alpha]_D^{25}$ = +118.6 (*c* = 1.00, $CHCl_3$). (*trans*-mixture of regioisomers)

(4a*S*,10b*S*)-10-Chloro-6-methyl-2,3,6,10b-tetrahydro-1*H*-pyrano[2,3-*c*]quinolin-5(4a*H*)-one (12b')

m.p. = 120-122°C (EtOAc-pentane).

IR (film) ν_{max}/cm^{-1}: 2980, 1684, 1458, 1383, 1142, 1108, 952.

^{1}H NMR (600 MHz, $CDCl_3$) *δ* = 7.20 (t, *J* = 8.1 Hz, 1H), 7.10 (dd, *J* = 8.1, 1.1 Hz, 1H), 6.87 (d, *J* = 1.1 Hz, 1H), 4.58 (d, *J* = 6.6 Hz, 1H), 3.93 – 3.88 (m, 1H), 3.83 (ddd, *J* = 12.3, 12.2, 2.5 Hz, 1H), 3.62 – 3.56 (m, 1H), 3.39 (s, 3H), 2.02 – 1.96 (m, 1H), 1.93 – 1.84 (m, 1H), 1.70 – 1.63 (m, 1H), 1.43 (ddd, *J* = 13.1, 13.0, 3.7 Hz, 1H).

^{13}C NMR (101 MHz, $CDCl_3$) *δ* = 168.1, 140.1, 133.8, 128.7, 125.7, 124.5, 113.7, 72.4, 63.5, 36.0, 30.2, 25.8, 24.3.

HRMS (ESI$^+$) $C_{13}H_{14}O_2NCl$ [M+H]$^+$ requires 252.0786; found 252.0787, Δ 0.4 ppm.

Chiral HPLC: (Chiralpak OD-H, 30% ^{i}PrOH, 70% hexane, 1.0 mL min^{-1}, λ = 254 nm) τ_R (major) = 8.5 min, τ_R (minor) = 14.4 min; er 93:7.

$[\alpha]_D^{25}$ = +12.8 (*c* = 0.1, $CHCl_3$).

(4a*R*,10b*S*)-8-Methoxy-6-methyl-2,3,6,10b-tetrahydro-1*H*-pyrano[2,3-*c*]quinolin-5(4a*H*)-one (13), (4a*R*,10b*S*)-10-methoxy-6-methyl-2,3,6,10b-tetrahydro-1*H*-pyrano[2,3-*c*]quinolin-5(4a*H*)-one (13b), and (4a*S*,10b*S*)-8-methoxy-6-methyl-2,3,6,10b-tetrahydro-1*H*-pyrano[2,3-*c*]quinolin-5(4a*H*)-one (13b')

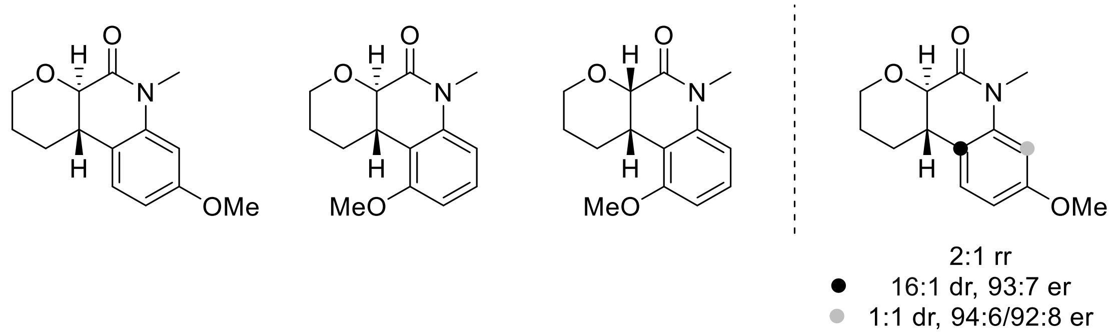

Prepared from *N*-(3-methoxyphenyl)-*N*-methyl-3,4-dihydro-2*H*-pyran-6-carboxamide (**13a**) (24.7 mg, 0.100 mmol) following **General Procedure C**. Purification by flash column chromatography (3:7→3:2, EtOAc: pentane) afforded an inseparable mixture of (4a*R*,10b*S*)-8-methoxy-6-methyl-2,3,6,10b-tetrahydro-1*H*-pyrano[2,3-*c*]quinolin-5(4a*H*)-one (**13**) and (4a*R*,10b*S*)-10-methoxy-6-methyl-2,3,6,10b-tetrahydro-1*H*-pyrano[2,3-*c*]quinolin-5(4a*H*)-one (**13b**) (17.1 mg, 2:1 rr, 93:7; 94:6 er) as an off-white solid, and (4a*S*,10b*S*)-8-methoxy-6-methyl-2,3,6,10b-tetrahydro-1*H*-pyrano[2,3-*c*]quinolin-5(4a*H*)-one (**13b'**) (2.9 mg, 92:8 er) as an off-white solid. The yield refers to the combined masses of all three products (20 mg, 81%). The diastereomeric ratio and regioisomeric ratio was determined by quantitative ^{1}H NMR analysis of the crude reaction mixture.

(4a*R*,10b*S*)-8-Methoxy-6-methyl-2,3,6,10b-tetrahydro-1*H*-pyrano[2,3-*c*]quinolin-5(4a*H*)-one (13) and (4a*R*,10b*S*)-10-methoxy-6-methyl-2,3,6,10b-tetrahydro-1*H*-pyrano[2,3-*c*]quinolin-5(4a*H*)-one (13b)

m.p. = 95-97 °C

IR (film) ν_{max}/cm^{-1}: 1691, 1614, 1590, 1514, 1469, 1451, 1377, 1343, 1314, 1255, 1220, 1141, 1110, 1088, 1064, 1042, 963, 765.

(4a*R*,10b*z*)-8-Methoxy-6-methyl-2,3,6,10b-tetrahydro-1H-pyrano[2,3-c]quinolin-5(4aH)-one (13)

^{1}H NMR (400 MHz, $CDCl_3$) δ = 7.10 (dd, *J* = 8.5, 1.3 Hz, 1H), 6.63 – 6.54 (m, 2H), 4.27 (ddt, *J* = 11.6, 4.6, 1.8 Hz, 1H), 3.82 (s, 3H), 3.67 (d, *J* = 13.0 Hz, 1H), 3.57 – 3.44 (m, 1H), 3.37 (s, 3H), 2.81 (dddd, *J* = 12.9, 11.4, 4.2, 1.3 Hz, 1H), 2.48 (dt, *J* = 12.6, 4.0 Hz, 1H), 1.92 – 1.73 (m, 2H), 1.65 – 1.49 (m, 1H).

^{13}C NMR (101 MHz, $CDCl_3$) δ = 168.6, 159.6, 140.3, 125.3, 119.5, 107.1, 102.6, 76.4, 68.4, 55.5, 36.0, 30.1, 26.4, 24.8.

Chiral HPLC: (Chiralpak IB N-3, 20% ^{i}PrOH, 80% hexane, 1.0 mL min^{-1}, λ = 210 nm) τ_R (major) = 19.5 min, τ_R (minor) = 25.2 min; er 93:7.

(4a*R*,10b*S*)-10-Methoxy-6-methyl-2,3,6,10b-tetrahydro-1*H*-pyrano[2,3-*c*]quinolin-5(4a*H*)-one (13b)

^{1}H NMR (400 MHz, $CDCl_3$) δ = 7.27 – 7.18 (m, 1H), 6.70 – 6.62 (m, 2H), 4.24 – 4.11 (m, 1H), 3.80 – 3.74 (m, 4H), 3.58 – 3.51 (m, 1H), 3.37 (s, 3H), 3.27 – 3.14 (m, 1H), 2.99 (ddd, *J* = 13.1, 11.2, 4.1 Hz, 1H), 1.75 – 1.63 (m, 3H).

^{13}C NMR (101 MHz, $CDCl_3$) δ = 168.3, 159.1, 141.1, 128.3, 114.9, 108.7, 107.6, 76.2, 68.6, 55.6, 37.4, 31.0, 28.3, 25.6.

Chiral HPLC: (Chiralpak IB N-3, 20% ^{i}PrOH, 80% hexane, 1.0 mL min^{-1}, λ = 210 nm) τ_R (major 1) = 16.2 min, τ_R (minor 1) = 22.1 min; er 94:6.

HRMS (ESI$^+$): $C_{14}H_{18}NO_3$ $[M+H]^+$ requires 248.1281; found 248.1283, Δ 0.8 ppm.

$[\alpha]_D^{25}$ = +154.2 (*c* = 0.10, $CHCl_3$).

(4a*R*,10b*R*)-8-Methoxy-6-methyl-2,3,6,10b-tetrahydro-1*H*-pyrano[2,3-*c*]quinolin-5(4a*H*)-one (13b')

m.p. = 94-97 °C

IR (film) ν_{max}/cm^{-1}: 1680, 1599, 1476, 1329, 1255, 1152, 1099, 1073, 775, 668.

^{1}H NMR (600 MHz, $CDCl_3$) δ = 7.21 (t, *J* = 8.3 Hz, 1H), 6.65 (dd, *J* = 8.3, 0.9 Hz, 1H), 6.60 (d, *J* = 8.0 Hz, 1H), 4.52 (d, *J* = 6.7 Hz, 1H), 3.92 – 3.79 (m, 5H), 3.52 (ddd, *J* = 12.7, 6.7, 4.0 Hz, 1H), 3.38 (s, 3H), 1.91 – 1.81 (m, 2H), 1.63 (d, *J* = 13.7 Hz, 1H), 1.40 (qd, *J* = 14.0, 13.4, 4.2 Hz, 1H).

^{13}C NMR (151 MHz, $CDCl_3$) δ = 168.4, 156.7, 139.5, 128.3, 115.8, 107.7, 106.0, 72.7, 63.4, 55.7, 31.8, 29.9, 25.9, 24.8.

HRMS (ESI$^+$): $C_{14}H_{18}NO_3$ $[M+H]^+$ requires 248.1281; found 248.1282, Δ 0.3 ppm.

Chiral HPLC: (Chiralpak IB N-3, 15% ^{i}PrOH, 85% hexane, 1.0 mL min^{-1}, λ = 222 nm) τ_R (major) = 12.5 min, τ_R (minor) = 24.9 min; er 92:8.

$[\alpha]_D^{25}$ = -6.9 (c = 0.10, $CHCl_3$).

(4a*R*,10b*S*)-6,8-Dimethyl-2,3,6,10b-tetrahydro-1*H*-pyrano[2,3-*c*]quinolin-5(4a*H*)-one (14), and (4a*R*,10b*S*)-6,10-dimethyl-2,3,6,10b-tetrahydro-1*H*-pyrano[2,3-*c*]quinolin-5(4a*H*) (14b')

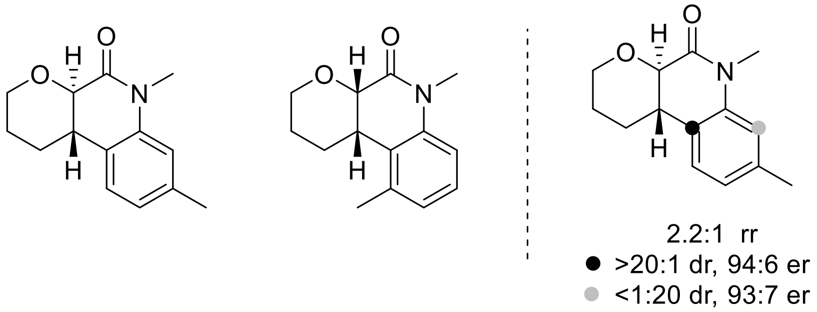

Prepared from *N*-methyl-*N*-(*m*-tolyl)-3,4-dihydro-2*H*-pyran-6-carboxamide (**14a**) (23.1 mg, 0.100 mmol) following **General Procedure C**. Purification by flash column chromatography (3:7→3:2, EtOAc: pentane) afforded (4a*R*,10b*S*)-6,8-dimethyl-2,3,6,10b-tetrahydro-1*H*-pyrano[2,3-*c*]quinolin-5(4a*H*)-one (**14**) (6.7 mg, >20:1 dr, 94:6 er) and (4a*R*,10b*S*)-6,10-dimethyl-2,3,6,10b-tetrahydro-1*H*-pyrano[2,3-*c*]quinolin-5(4a*H*) (**14b'**) (3.0 mg, >20:1 dr, 93:7 er) as off-white solids. The yield refers to the combined masses of the three products (9.7 mg, 42%). The diastereomeric ratio and regiosomeric ratio was determined by quantitative ^{1}H NMR analysis of the crude reaction mixture.

(4a*R*,10b*S*)-6,8-Dimethyl-2,3,6,10b-tetrahydro-1*H*-pyrano[2,3-*c*]quinolin-5(4a*H*)-one (14)

m.p. = 125-129 °C

IR (film) ν_{max}/cm^{-1}: 1691, 1615, 1469, 1423, 1376 1344, 1315, 1254, 1111, 1091, 963, 769.

^{1}H NMR (600 MHz, $CDCl_3$) δ = 7.09 (dd, J = 7.7, 1.2 Hz, 1H), 6.92 (dd, J = 7.7, 1.6 Hz, 1H), 6.81 (d, J = 1.6 Hz, 1H), 4.27 (dd, J = 11.5, 4.5 Hz, 1H), 3.68 (d, J = 13.1 Hz, 1H), 3.50 (td, J = 11.9, 2.4 Hz, 1H), 3.39 (s, 3H), 2.87 – 2.80 (m, 1H), 2.51 (dd, J = 12.6, 3.9 Hz, 1H), 2.37 (s, 3H), 1.85 (ddd, J = 17.0, 8.5, 4.2 Hz, 1H), 1.77 (dq, J = 13.7, 2.2 Hz, 1H), 1.58 (tdd, J = 12.9, 11.4, 4.1 Hz, 1H).

^{13}C NMR (151 MHz, $CDCl_3$) δ = 168.5, 139.1, 137.9, 124.4, 124.2, 124.0, 115.7, 76.3, 68.3, 36.2, 30.1, 26.3, 24.9, 21.4.

HRMS (ESI$^+$): $C_{14}H_{17}NO_2$ [M+H]$^+$ requires 232.1332; found 232.1333, Δ 0.5 ppm.

Chiral HPLC: (Chiralpak IG-3, 20% ^{i}PrOH, 80% hexane, 1.0 mL min^{-1}, λ = 210 nm) τ_R (major) = 21.4 min, τ_R (minor) = 18.6 min; er 94:6.

$[\alpha]_D^{25}$ = +145.3 (c = 0.10, $CHCl_3$).

(4a*R*,10b*S*)-6,8-Dimethyl-2,3,6,10b-tetrahydro-1*H*-pyrano[2,3-*c*]quinolin-5(4a*H*)-one (14b')

m.p. = 118-120 °C

IR (film) ν_{max}/cm^{-1}: 1699, 1684, 1653, 1559, 1541, 1507, 1473, 1457, 772, 668.

^{1}H NMR (600 MHz, $CDCl_3$) δ = 7.17 (t, J = 7.9 Hz, 1H), 6.92 (d, J = 7.6 Hz, 1H), 6.83 (d, J = 8.2 Hz, 1H), 4.56 (d, J = 6.3 Hz, 1H), 3.94 – 3.84 (m, 2H), 3.39 (s, 3H), 3.28 (ddd, J = 12.8, 6.4, 3.9 Hz, 1H), 2.35 (s, 3H), 1.90 – 1.81 (m, 2H), 1.70 – 1.64 (m, 1H), 1.49 (td, J = 13.3, 3.5 Hz, 1H).

^{13}C NMR (151 MHz, $CDCl_3$) δ = 168.0, 138.7, 136.0, 127.6, 126.0, 125.6, 113.1, 72.8, 63.2, 35.4, 30.0, 26.0, 24.4, 18.7.

HRMS (ESI$^+$): $C_{14}H_{17}NO_2$ $[M+H]^+$ requires 232.1332; found 232.1333, Δ 0.3 ppm.

Chiral HPLC: (Chiralpak IB N-3, 15% ^{i}PrOH, 85% hexane, 1.0 mL min^{-1}, λ = 222 nm) τ_R (major) = 19.0 min, τ_R (minor) = 23.3 min; er 93:7.

$[\alpha]_D^{25}$ = +14.3 (*c* = 0.10, $CHCl_3$).

(4a*R*,10b*S*)-8,10-Difluoro-6-methyl-2,3,6,10b-tetrahydro-1*H*-pyrano[2,3-*c*]quinolin-5(4a*H*)-one (15) and (4a*S*,10b*S*)-8,10-difluoro-6-methyl-2,3,6,10b-tetrahydro-1*H*-pyrano[2,3-*c*]quinolin-5(4a*H*)-one (15')

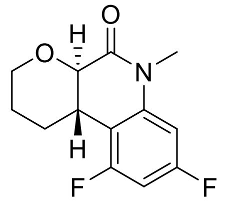

Prepared from *N*-(3,5-difluorophenyl)-*N*-methyl-3,4-dihydro-2*H*-pyran-6-carboxamide (**15a**) (23.5 mg, 0.100 mmol) following **General Procedure C**. Purification by flash column chromatography (3:7→1:1, EtOAc: pentane) afforded (4a*R*,10b*S*)-8,10-difluoro-6-methyl-2,3,6,10b-tetrahydro-1*H*-pyrano[2,3-*c*]quinolin-5(4a*H*)-one (**15 & 15'**; 4:1) as off-white solids. The yield refers to the combined masses of the two products (14 mg, 57%). The diastereomeric ratio and regiosomeric ratio was determined by quantitative ^{1}H NMR analysis of the crude reaction mixture.

(4a*R*,10b*S*)-8,10-Difluoro-6-methyl-2,3,6,10b-tetrahydro-1*H*-pyrano[2,3-*c*]quinolin-5(4a*H*)-one (15)

m.p. = 90-92 °C

IR (film) ν_{max}/cm^{-1}: 2980, 1382, 1251, 1151, 1072, 955.

^{1}H NMR (600 MHz, $CDCl_3$) δ = 6.61 – 6.48 (m, 2H), 4.28 – 4.23 (m, 1H), 3.80 (d, *J* = 12.8 Hz, 1H), 3.56 (td, *J* = 11.7, 2.6 Hz, 1H), 3.36 (s, 3H), 2.99 (dddt, *J* = 13.1, 11.4, 3.9, 1.8 Hz, 1H), 2.91 – 2.84 (m, 1H), 1.91 – 1.67 (m, 3H).

^{19}F NMR (565 MHz, $CDCl_3$) δ = -110.02 (q, *J* = 8.7 Hz), -110.23 (q, *J* = 8.5 Hz).

^{13}C NMR (151 MHz, $CDCl_3$) δ = 167.9, 162.7 (dd, *J* = 57.8, 14.8 Hz), 161.0 (dd, *J* = 61.5, 14.7 Hz), 142.9 – 141.6 (m), 109.8 (dd, *J* = 15.6, 4.1 Hz), 99.4 (dd, *J* = 29.1, 24.8 Hz), 99.4 (dd, *J* = 26.2, 3.3 Hz), 75.5, 68.5, 36.2 (d, *J* = 3.1 Hz), 30.8, 27.3 (d, *J* = 13.3 Hz), 24.9 (d, *J* = 3.1 Hz).

HRMS (ESI$^+$): $C_{13}H_{13}NO_2F_2$ $[M+H]^+$ requires 254.0987; found 254.0999, Δ 4.7 ppm.

Chiral HPLC: (Chiralpak OD-H, 30% ^{i}PrOH, 70% hexane, 1.0 mL min^{-1}, λ = 260 nm) τ_R (major) = 14.6 min, τ_R (minor) = 21.9 min; er 88:12

$[\alpha]_D^{25}$ = +82.4 (*c* = 0.10, $CHCl_3$).

(4a*S*,10b*S*)-8,10-Difluoro-6-methyl-2,3,6,10b-tetrahydro-1*H*-pyrano[2,3-c]quinolin-5(4a*H*)-one (15')

m.p. = 70-72 °C

IR (film) ν_{max}/cm^{-1}: 2980, 2970, 1383, 1150, 1066, 965.

^{1}H NMR (600 MHz, CDCl$_3$) δ = 6.56 (td, *J* = 8.9, 2.3 Hz, 1H), 6.51 (dt, *J* = 10.4, 1.9 Hz, 1H), 4.56 (d, *J* = 6.6 Hz, 1H), 3.93 – 3.87 (m, 1H), 3.79 (td, *J* = 12.3, 2.4 Hz, 1H), 3.43 (ddd, *J* = 12.9, 6.7, 4.1 Hz, 1H), 3.36 (s, 3H), 1.93 – 1.81 (m, 2H), 1.69 – 1.62 (m, 1H), 1.51 – 1.40 (m, 1H).

^{19}F NMR (565 MHz, CDCl$_3$) δ = -109.79, -116.28 (t, *J* = 8.5 Hz).

^{13}C NMR (151 MHz, CDCl$_3$) δ = 168.0, 162.0 (dd, *J* = 340.3, 14.5 Hz), 160.4 (dd, *J* = 340.1, 14.6 Hz), 141.1 – 140.7 (m), 110.8 (dd, *J* = 21.4, 3.7 Hz), 98.8 (dd, *J* = 26.5, 3.4 Hz), 98.5 (t, *J* = 25.9 Hz), 72.2, 63.3, 31.3 (d, *J* = 2.8 Hz), 30.0, 25.7, 25.6.

HRMS (ESI$^+$): $C_{13}H_{15}NO_2F_2$ [M+H]$^+$ requires 254.0987; found 254.0991, Δ 1.5 ppm.

Chiral HPLC: (Chiralpak OD-H, 30% ^{i}PrOH, 70% hexane, 1.0 mL min^{-1}, λ = 260 nm) τ_R (major) = 9.8 min, τ_R (minor) = 15.0 min; er 84:16.

$[\alpha]_D^{25}$ = -14.0 (*c* = 0.10, CHCl$_3$).

(4a*R*,10b*S*)-9-Fluoro-6-methyl-2,3,6,10b-tetrahydro-1*H*-pyrano[2,3-c]quinolin-5(4a*H*)-one (16)

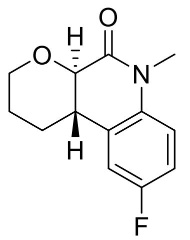

Prepared from *N*-(4-fluorophenyl)-*N*-methyl-3,4-dihydro-2*H*-pyran-6-carboxamide (**16a**) (23.5 mg, 0.100 mmol) following **General Procedure C**. Purification by flash column chromatography (3:2, EtOAc: pentane) afforded (4a*R*,10b*S*)-9-fluoro-6-methyl-2,3,6,10b-tetrahydro-1*H*-pyrano[2,3-c]quinolin-5(4a*H*)-one (**16**) (22.1 mg, 94%, >20:1 dr, 94:6 er) as a colourless oil.

IR (film) ν_{max}/cm^{-1}: 2938, 2852, 1689, 1499, 1436, 1110.

^{1}H NMR (400 MHz, CDCl$_3$) *δ* = 7.02–6.90 (m, 3H), 4.32–4.24 (m, 1H), 3.69 (d, *J* = 13.1 Hz, 1H), 3.50 (ddd, *J* = 11.8, 11.7, 2.9 Hz, 1H), 3.38 (d, *J* = 0.6 Hz, 3H), 2.86 (dddd, *J* = 13.0, 11.5, 4.2, 1.3 Hz, 1H), 2.53–2.40 (m, 1H), 1.91–1.74 (m, 2H), 1.63–1.50 (m, 1H).

^{13}C NMR (101 MHz, CDCl$_3$) *δ* = 168.0, 159.3 (d, *J* = 243.1 Hz), 135.6, 129.5 (d, *J* = 7.3 Hz), 116.2 (d, *J* = 8.2 Hz), 114.3 (d, *J* = 22.5 Hz), 112.2 (d, *J* = 23.9 Hz), 75.8, 68.4, 36.5, 30.5, 26.3, 24.8.

^{19}F NMR (101 MHz, CDCl$_3$) *δ* = −119.3.

HRMS (ESI$^+$) $C_{13}H_{14}FNO_2$ [M+Na]$^+$ requires 258.0901; found 258.0901, Δ 0.3 ppm.

Chiral HPLC: (Chiralpak OD-H, 30% ^{i}PrOH, 70% hexane, 1.0 mL min^{-1}, λ = 260 nm) τ_R (major) = 12.8 min, τ_R (minor) = 17.8 min; er 94:6.

$[\alpha]_D^{25}$ = +95.4 (*c* = 0.50, CHCl$_3$).

(4a*R*,10b*S*)-6-Benzyl-9-chloro-2,3,6,10b-tetrahydro-1*H*-pyrano[2,3-c]quinolin-5(4a*H*)-one (17)

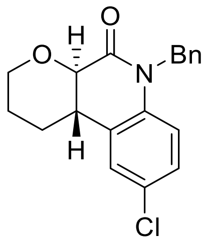

Prepared from *N*-benzyl-*N*-(4-chlorophenyl)-3,4-dihydro-2*H*-pyran-6-carboxamide (**17a**) (32.9 mg, 0.100 mmol) following **General Procedure C**. Purification by flash column chromatography (3:7, EtOAc: pentane) afforded (4a*R*,10b*S*)-6-benzyl-9-chloro-2,3,6,10b-tetrahydro-1*H*-pyrano[2,3-c]quinolin-5(4a*H*)-one (**17**) (28.9 mg, 88%, >20:1 dr, 94:6 er) as an off-white solid.

m.p. = 158-160 °C

IR (film) ν_{max}/cm^{-1}: 1694, 1489, 1455, 1422, 1376, 1303, 1253, 1212, 1182, 1122, 977, 963, 813, 771, 703, 677, 667, 633.

^{1}H NMR (400 MHz, $CDCl_3$) δ = 7.41 – 7.32 (m, 2H), 7.36 – 7.24 (m, 3H), 7.24 (dd, *J* = 2.4, 1.3 Hz, 1H), 7.19 (ddd, *J* = 8.7, 2.4, 1.0 Hz, 1H), 6.92 (d, *J* = 8.7 Hz, 1H), 5.42 (d, *J* = 16.2 Hz, 1H), 5.11 (d, *J* = 16.2 Hz, 1H), 4.44 – 4.35 (m, 1H), 3.96 (d, *J* = 13.2 Hz, 1H), 3.63 (td, *J* = 11.7, 2.8 Hz, 1H), 3.02 (dddt, *J* = 12.7, 11.4, 4.4, 1.1 Hz, 1H), 2.59 (dd, *J* = 12.6, 3.8 Hz, 1H), 2.06 – 1.85 (m, 2H), 1.71 (tdd, *J* = 12.7, 11.4, 4.3 Hz, 1H).

^{13}C NMR (101 MHz, $CDCl_3$) δ = 168.3, 136.9, 136.3, 129.3, 129.2, 129.0, 128.8, 127.8, 127.4, 126.6, 125.1, 117.0, 75.6, 68.3, 46.5, 36.5, 26.2, 24.6.

HRMS (ESI$^+$): $C_{19}H_{19}NO_2Cl$ [M+H]$^+$ requires 328.1099; found 328.1097, Δ -0.6 ppm.

Chiral HPLC: (Chiralpak IF-3, 50% ^{i}PrOH, 50% hexane, 1.0 mL min^{-1}, λ = 210 nm) τ_R (major) = 14.7 min, τ_R (minor) = 18.6 min; er 94:6.

$[\alpha]_D^{25}$ = +104.2 (*c* = 0.10, $CHCl_3$).

(4a*R*,10b*S*)-9-Bromo-6-methyl-2,3,6,10b-tetrahydro-1*H*-pyrano[2,3-*c*]quinolin-5(4a*H*)-one (18)

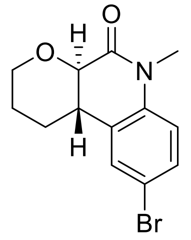

Prepared from *N*-(4-bromophenyl)-*N*-methyl-3,4-dihydro-2*H*-pyran-6-carboxamide (**18a**) (29.6 mg, 0.100 mmol) following **General Procedure C**. Purification by flash column chromatography (3:2, EtOAc: pentane) afforded (4a*R*,10b*S*)-9-bromo-6-methyl-2,3,6,10b-tetrahydro-1*H*-pyrano[2,3-*c*]quinolin-5(4a*H*)-one (**18**) (21.2 mg, 72%, >20:1 dr, 95:5 er) as an off-white solid.

m.p. = 130-134 °C

IR (film) ν_{max}/cm^{-1}: 1691,1489,1469,1415,1374,1252,1218,1112,1082,801,770.

^{1}H NMR (400 MHz, $CDCl_3$) δ = 7.41 (ddd, J = 8.6, 2.3, 0.8 Hz, 1H), 7.30 (dd, J = 2.3, 1.2 Hz, 1H), 6.86 (d, J = 8.6 Hz, 1H), 4.32 – 4.24 (m, 1H), 3.68 (d, J = 13.1 Hz, 1H), 3.50 (td, J = 11.5, 3.1 Hz, 1H), 3.36 (s, 3H), 2.86 (td, J = 12.3, 4.2 Hz, 1H), 2.48 (dd, J = 12.9, 3.8 Hz, 1H), 1.93 – 1.75 (m, 2H), 1.67 – 1.52 (m, 1H).

^{13}C NMR (101 MHz, $CDCl_3$) δ = 168.30, z138.5, 130.8, 129.3, 127.8, 116.5, 116.4, 75.6, 68.3, 36.4, 30.2, 26.1, 24.7.

HRMS (ESI$^+$): $C_{13}H_{15}NO_2Br$ $[M+H]^+$ requires 296.0281; found 296.0282, Δ 0.3 ppm.

Chiral HPLC: (Chiralpak OD-H, 30% ^{i}PrOH, 70% hexane, 1.0 mL min^{-1}, λ = 254 nm) τ_R (major) = 15.9 min, τ_R (minor) = 22.2 min; er 93:7.

$[\alpha]_D^{25}$ = +118.8 (c = 0.10, $CHCl_3$).

(4a*R*,10b*S*)-6,9-Dimethyl-2,3,6,10b-tetrahydro-1*H*-pyrano[2,3-c]quinolin-5(4a*H*)-one (19)

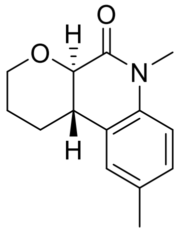

Prepared from *N*-methyl-*N*-(p-tolyl)-3,4-dihydro-2H-pyran-6-carboxamide (**19a**) (23.2 mg, 0.100 mmol) following **General Procedure C**. Purification by flash chromatography (7:3, EtOAc: pentane) afforded (4a*R*,10b*S*)-6,9-Dimethyl-2,3,6,10b-tetrahydro-1*H*-pyrano[2,3-c]quinolin-5(4a*H*)-one (**19**) (22.2 mg, 96%, >20:1 dr, 95:5 er) as a white solid.

m.p. = 112-114 °C (EtOAc-pentane).

IR (film) ν_{max}/cm^{-1}: 2980, 2970, 1682, 1503, 1385, 1375, 1258, 1158, 1138, 1104, 1088, 1063, 959, 819.

^{1}H NMR (400 MHz, $CDCl_3$) δ = 7.10 (d, J = 8.3 Hz, 1H), 7.01 (s, 1H), 6.88 (d, J = 8.2 Hz, 1H), 4.31–4.22 (m, 1H), 3.68 (d, J = 13.1 Hz, 1H), 3.49 (td, J = 11.7, 2.7 Hz, 1H), 3.37 (s, 3H), 2.85 (td, J = 12.3, 4.2 Hz, 1H), 2.55–2.47 (m, 1H), 2.34 (s, 3H), 1.93–1.71 (m, 2H), 1.60 (tdd, J = 12.8, 11.5, 4.3 Hz, 1H).

^{13}C NMR (101 MHz, $CDCl_3$) δ = 168.3, 137.0, 133.1, 128.4, 127.1, 125.4, 114.9, 76.3, 68.4, 36.6, 30.2, 26.4, 25.0, 21.0.

HRMS (ESI$^+$) $C_{14}H_{17}NO_2$ $[M+H]^+$ requires 232.1332; found 232.1335, Δ 1.5 ppm.

Chiral HPLC: (Chiralpak OD-H, 40% ^{i}PrOH, 60% hexane, 1.0 mL min^{-1}, λ = 260 nm) τ_R (major) = 8.0 min, τ_R (minor) = 11.2 min; er 95:5.

$[\alpha]_D^{25}$ = +155.6 (c = 0.50 $CHCl_3$).

(4a*R*,10b*S*)-6-Methyl-9-(trifluoromethyl)-2,3,6,10b-tetrahydro-1*H*-pyrano[2,3-*c*]quinolin-5(4a*H*)-one (20)

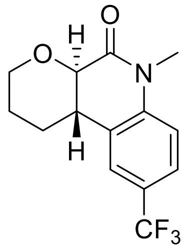

Prepared from *N*-methyl-*N*-(4-(trifluoromethyl)phenyl)-3,4-dihydro-2*H*-pyran-6-carboxamide (**20a**) (28.5 mg, 0.100 mmol) following **General Procedure C**. Purification by flash column chromatography (3:7, EtOAc: pentane) afforded (4a*R*,10b*S*)-6-methyl-9-(trifluoromethyl)-2,3,6,10b-tetrahydro-1*H*-pyrano[2,3-*c*]quinolin-5(4a*H*)-one (**20**) (28.9 mg, 28%, >20:1 dr, 92:8 er) as an off-white solid.

m.p. = 149-152 °C

IR (film) ν_{max}/cm^{-1}: 1700, 1619, 1508, 1378, 1335, 1307, 1289, 1276, 1253, 1218, 1168, 1149, 1112, 1097, 1082, 991, 963, 827, 804, 769, 668.

^{1}H NMR (400 MHz, $CDCl_3$) δ = 7.60 – 7.55 (m, 1H), 7.44 (s, 1H), 7.08 (d, *J* = 8.5 Hz, 1H), 3.73 (d, *J* = 13.2 Hz, 1H), 3.52 (td, *J* = 11.8, 2.8 Hz, 1H), 3.43 (s, 3H), 2.92 (td, *J* = 12.3, 4.2 Hz, 1H), 2.60 – 2.52 (m, 1H), 1.94 – 1.82 (m, 1H), 1.73 – 1.60 (m, 1H).

^{19}F NMR (565 MHz, $CDCl_3$) δ -62.0.

^{13}C {^{1}H, ^{19}F} NMR (126 MHz, $CDCl_3$) δ = 168.2, 142.1, 127.8, 125.5, 125.4, 124.1, 121.9, 114.8, 75.5, 68.3, 36.4, 30.2, 26.0, 24.6.

HRMS (ESI$^+$): $C_{14}H_{15}NO_2F_3$ $[M+H]^+$ requires 286.1049; found 286.1049, Δ -0.1 ppm.

Chiral HPLC: (Chiralpak OD-H, 30% ^{i}PrOH, 70% hexane, 1.0 mL min^{-1}, λ = 210 nm) τ_R (major) = 14.1 min, τ_R (minor) = 19.4 min; er 92:8.

$[\alpha]_D^{25}$ = +120.0 (*c* = 0.10, $CHCl_3$).

(4a*R*,10b*S*)-9-(*Tert*-butyl)-6-methyl-2,3,6,10b-tetrahydro-1*H*-pyrano[2,3-c]quinolin-5(4a*H*)-one (21)

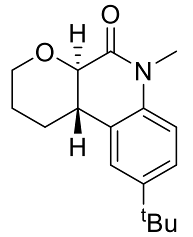

Prepared from *N*-(4-(*tert*-butyl)phenyl)-*N*-methyl-3,4-dihydro-2*H*-pyran-6-carboxamide (**21a**) (27.3 mg, 0.100 mmol) following **General Procedure C**. Purification by flash column chromatography (1:1, EtOAc: pentane) afforded (4a*R*,10b*S*)-9-(tert-butyl)-6-methyl-2,3,6,10b-tetrahydro-1*H*-pyrano[2,3-c]quinolin-5(4a*H*)-one (**21**) (25.1 mg, 92%, >20:1 dr, 95:5 er) as a colourless oil.

IR (film) ν_{max}/cm^{-1}: 2956, 2905, 2858, 1691, 1507, 1377, 1109, 1086.

^{1}**H NMR** (400 MHz, $CDCl_3$) δ = 7.34–7.28 (m, 1H), 7.24–7.19 (m, 1H), 6.93 (d, *J* = 8.4 Hz, 1H), 4.34–4.20 (m, 1H), 3.72 (d, *J* = 13.1 Hz, 1H), 3.51 (ddd, *J* = 11.8, 11.8, 2.7 Hz, 1H), 3.38 (s, 3H), 2.93–2.79 (m, 1H), 2.61–2.51 (m, 1H), 1.96–1.75 (m, 2H), 1.71–1.52 (m, 1H), 1.32 (s, 9H).

^{13}**C NMR** (101 MHz, $CDCl_3$) δ = 168.3, 146.3, 136.8, 126.6, 124.6, 121.5, 114.5, 76.2, 68.3, 36.7, 34.5, 31.4, 30.0, 29.7, 26.2, 24.9.

HRMS (ESI$^+$): $C_{17}H_{24}NO_2$ $[M+H]^+$ requires 274.1802; found 274.1801, Δ 0.0 ppm.

Chiral HPLC: (Chiralpak OD-H, 30% ^{i}PrOH, 70% hexane, 1.0 mL min^{-1}, λ = 270 nm) τ_R (major) = 9.8 min, τ_R (minor) = 13.3 min; er 95:5.

$[\alpha]_D^{25}$ = +134.7 (*c* = 0.50, $CHCl_3$)

(4a*R*,10b*S*)-9-Methoxy-6-methyl-2,3,6,10b-tetrahydro-1*H*-pyrano[2,3-*c*]quinolin-5(4a*H*)-one (22)

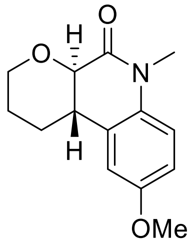

Prepared from *N*-(4-methoxyphenyl)-*N*-methyl-3,4-dihydro-2*H*-pyran-6-carboxamide (**22a**) (24.7 mg, 0.100 mmol) following **General Procedure C**. Purification by flash column chromatography (7:3, EtOAc: pentane) afforded (4a*R*,10b*S*)-9-Methoxy-6-methyl-2,3,6,10b-tetrahydro-1*H*-pyrano[2,3-*c*]quinolin-5(4a*H*)-one (**22**) (22.6 mg, 91%, >20:1 dr, 95:5 er) as an off-white solid.

m.p. = 124-126 °C (EtOAc-pentane).

IR (film) ν_{max}/cm^{-1}: 2980, 1683, 1502, 1236, 1111.

^{1}**H NMR** (400 MHz, $CDCl_3$) δ = 6.91 (d, *J* = 8.7 Hz, 1H), 6.85–6.73 (m, 2H), 4.34–4.21 (m, 1H), 3.80 (s, 3H), 3.68 (d, *J* = 13.1 Hz, 1H), 3.49 (td, *J* = 11.8, 2.7 Hz, 1H), 3.37 (s, 3H), 2.92–2.79 (m, 1H), 2.58–2.40 (m, 1H), 1.92–1.71 (m, 2H), 1.66–1.51 (m, 1H).

^{13}**C NMR** (101 MHz, $CDCl_3$) δ = 168.0, 156.0, 133.0, 129.0, 115.8, 111.8, 111.7, 76.1, 68.4, 55.7, 36.7, 30.4, 26.4, 25.0.

HRMS (ESI$^+$) $C_{14}H_{17}NO_3$ $[M+H]^+$ requires 248.1281; found 248.1280, Δ 0.5 ppm.

Chiral HPLC: (Chiralpak OD-H, 30% ^{i}PrOH, 70% hexane, 1.0 mL min^{-1}, λ = 260 nm) τ_R (major) = 19.4 min, τ_R (minor) = 14.0 min; er 95:5.

$[\alpha]_D^{25}$ = +141.6 (*c* = 1.00, $CHCl_3$).

(4a*R*,10b*S*)-9-(Methylthio)-6-methyl-2,3,6,10b-tetrahydro-1*H*-pyrano[2,3-*c*]quinolin-5(4a*H*)-one (23)

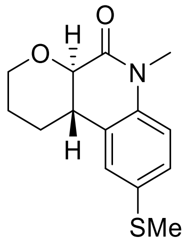

Prepared from *N*-(4-(methylthio)phenyl)-*N*-methyl-3,4-dihydro-2*H*-pyran-6-carboxamide (**23a**) (26.3 mg, 0.100 mmol) following **General Procedure C**. Purification by flash column chromatography (7:3, EtOAc: pentane) afforded (4a*R*,10b*S*)-9-(methylthio)-6-methyl-2,3,6,10b-tetrahydro-1*H*-pyrano[2,3-c]quinolin-5(4a*H*)-one (**23**) (22.7 mg, 86%, >20:1 dr, 94:6 er) as a colourless oil.

IR (film) ν_{max}/cm^{-1}: 2980, 1686, 1492, 1252, 1118, 1107.

^{1}H NMR (400 MHz, $CDCl_3$) δ = 7.22 (ddd, *J* = 8.4, 2.2, 0.9 Hz, 1H), 7.14 (dd, *J* = 2.2, 1.3 Hz, 1H), 6.93 (d, *J* = 8.4 Hz, 1H), 4.31 – 4.24 (m, 1H), 3.69 (d, *J* = 13.1 Hz, 1H), 3.49 (ddd, *J* = 11.7, 2.9 Hz, 1H), 3.37 (s, 3H), 2.91 – 2.80 (m, 1H), 2.55 – 2.50 (m, 1H), 2.48 (s, 3H), 1.91 – 1.74 (m, 2H), 1.66 – 1.54 (m, 2H).

^{13}C NMR (101 MHz, $CDCl_3$) δ = 168.2, 137.3, 132.9, 128.0, 127.1, 124.5, 115.6, 76.0, 68.4, 36.6, 30.2, 26.3, 24.9, 17.1.

HRMS (ESI$^+$) $C_{14}H_{17}O_2NS$ [M+H]$^+$ requires 264.1053; found 264.1054, Δ 0.4 ppm.

Chiral HPLC: (Chiralpak OD-H, 30% ^{i}PrOH, 70% hexane, 1.0 mL min^{-1}, λ = 260 nm) τ_R (major) = 16.6 min, τ_R (minor) = 22.5 min; er 94:6.

$[\alpha]_D^{25}$ = +147.7 (*c* = 1.00, $CHCl_3$).

(3a*R*,9b*S*)-5-Methyl-1,2,5,9b-tetrahydrofuro[2,3-c]quinolin-4(3a*H*)-one (24)

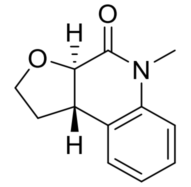

Prepared from *N*-methyl-*N*-phenyl-4,5-dihydrofuran-2-carboxamide (**24a**) (20.3 mg, 0.100 mmol) following **General Procedure C**. Purification by flash column chromatography (3:2, EtOAc: pentane) afforded (3a*R*,9b*S*)-5-methyl-1,2,5,9b-tetrahydrofuro[2,3-c]quinolin-4(3a*H*)-one (**24**) (17.0 mg, 84%, 8:1 dr, 58:42 er) as an off-white solid.

m.p. = 122-124 °C (EtOAc-pentane).

IR (film) ν_{max}/cm^{-1}: 2888, 1693, 1459, 1121, 1094, 1019.

^{1}H NMR (400 MHz, $CDCl_3$) δ = 7.34 – 7.29 (m, 1H), 7.17 (ddd, *J* = 7.4, 1.5, 1.5 Hz, 1H), 7.09 (ddd, *J* = 7.5, 7.4, 1.1 Hz, 1H), 7.03 (d, *J* = 1.1 Hz, 1H), 4.33 – 4.26 (m, 2H), 3.90 (dd, *J* = 13.7, 0.7 Hz, 1H), 3.39 (d, *J* = 0.6 Hz, 3H), 3.30 – 3.17 (m, 1H), 2.53 – 2.43 (m, 1H), 2.20 – 2.07 (m, 1H).

^{13}C NMR (101 MHz, $CDCl_3$) δ = 169.2, 141.0, 128.0, 127.3, 125.1, 123.3, 115.3, 79.1, 70.0, 42.2, 29.7, 27.5.

HRMS (ESI$^+$) $C_{12}H_{21}NO_2$ [M+Na]$^+$ requires 226.0838; found 226.0840, Δ 0.9 ppm.

Chiral HPLC: (Chiralpak IC, 40% ^{i}PrOH, 60% hexane, 1.0 mL min^{-1}, λ = 260 nm) τ_R (major) = 51.5 min, τ_R (minor) = 55.8 min; er 57:43.

$[\alpha]_D^{25}$ = +32.8 (*c* = 0.50, $CHCl_3$).

(4a*R*,10b*S*)-6-Methyl-2,3,6,10b-tetrahydro-1*H*-pyrano[2,3-*c*]quinolin-5(4a*H*)-one-4a,7,8,9,10-*d₅* (30)

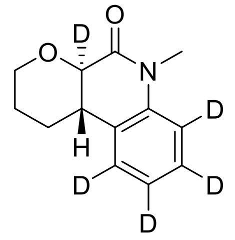

Prepared from *N*-Methyl-*N*-(phenyl-d_5)-3,4-dihydro-2*H*-pyran-6-carboxamide (**30a**) (22.2 mg, 0.100 mmol) following **General Procedure C**. Purification by flash column chromatography (2:3, EtOAc: pentane) afforded (4a*R*,10b*S*)-6-methyl-2,3,6,10b-tetrahydro-1*H*-pyrano[2,3-*c*]quinolin-5(4a*H*)-one-4a,7,8,9,10-d_5 **(30)** (22.0 mg, 99%) as an off-white solid.

m.p. = 76-80 °C (EtOAc-pentane).

IR (film) ν_{max}/cm^{-1}: 1693, 1579, 1473, 1433, 1390, 1337, 1280, 1218, 1098, 1074, 1028, 770, 668.

^{1}H NMR (600 MHz, $CDCl_3$) δ = 4.28 (ddd, *J* = 11.5, 4.3, 2.1 Hz, 1H), 3.51 (td, *J* = 11.8, 2.7 Hz, 1H), 3.40 (s, 3H), 2.89 (dd, *J* = 11.5, 4.1 Hz, 1H), 2.53 (dd, *J* = 12.6, 3.8 Hz, 1H), 1.95 – 1.73 (m, 2H), 1.69 – 1.59 (m, 1H).

^{13}C NMR (151 MHz, $CDCl_3$) δ = 168.4, 139.2, 127.9 – 127.2 (m), 127.1, 124.7 – 123.9 (m), 123.2 – 122.5 (m), 115.0 – 114.1 (m), 75.9 – 75.3 (m), 68.3, 36.4, 30.1, 26.2, 24.8.

HRMS (ESI$^+$) C_{13}H11D5NO_2 [M+H]$^+$ requires 223.1489; found 223.1491, Δ 0.6 ppm.

Chiral HPLC: (Chiralpak IB N-3, 30% ^{i}PrOH, 70% hexane, 1.0 mL min^{-1}, λ = 210 nm) τ_R (major) = 11.1 min, τ_R (minor) = 16.6 min; er 94:6.

$[\alpha]_D^{25}$ = +120.0 (*c* = 1.00, $CHCl_3$).

(4a*R*,10b*S*)-6-Ethyl-2,3,6,10b-tetrahydro-1*H*-pyrano[2,3-c]quinolin-5(4a*H*)-one (S1)

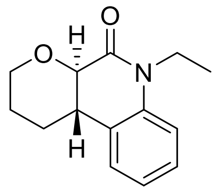

Prepared from *N*-ethyl-*N*-phenyl-3,4-dihydro-2*H*-pyran-6-carboxamide (**S1a**) (23.1 mg, 0.100 mmol) following **General Procedure C**. Purification by flash column chromatography (1:1, EtOAc: pentane) afforded (4a*R*,10b*S*)-6-ethyl-2,3,6,10b-tetrahydro-1*H*-pyrano[2,3-c]quinolin-5(4a*H*)-one (**S1**) (22.2 mg, 97%, >20:1 dr, 93:7 er) as an off-white solid.

m.p. = 80-82 °C

IR (film) ν_{max}/cm^{-1}: 2970, 2935, 2850, 1687, 1458, 1389, 1242, 1113.

^{1}H NMR (400 MHz, $CDCl_3$) δ = 7.32–7.27 (m, 1H), 7.22 (dt, *J* = 7.7, 1.5 Hz, 1H), 7.09 (ddd, *J* = 7.6, 7.5, 1.1 Hz, 1H), 7.04 (dd, *J* = 8.2, 1.1 Hz, 1H), 4.27 (ddt, *J* = 11.5, 4.5, 1.7 Hz, 1H), 4.16–4.03 (m, 1H), 4.02–3.85 (m, 1H), 3.71 (d, *J* = 13.2 Hz, 1H), 3.50 (td, *J* = 11.8, 2.7 Hz, 1H), 2.92–2.79 (m, 1H), 2.58–2.41 (m, 1H), 1.95–1.70 (m, 2H), 1.68–1.48 (m, 1H), 1.25 (t, *J* = 7.1 Hz, 3H).

^{13}C NMR (101 MHz, $CDCl_3$) δ = 167.9, 138.3, 128.1, 127.7, 125.0, 123.4, 114.9, 76.06, 68.4, 38.0, 36.7, 26.4, 24.9, 12.9.

HRMS (ESI$^+$): $C_{14}H_{17}NO_2$ $[M+Na]^+$ requires 254.1152; found 254.1153, Δ 0.6 ppm.

Chiral HPLC: (Chiralpak OD-H, 30% ^{i}PrOH, 70% hexane, 1.0 mL min^{-1}, λ = 260 nm) τ_R (major) = 8.6 min, τ_R (minor) = 12.4 min; er 93:7.

$[\alpha]_D^{25}$ = +154.7(*c* = 0.50, $CHCl_3$).

(4a*R*,10b*S*)-9-Chloro-6-methyl-2,3,6,10b-tetrahydro-1*H*-pyrano[2,3-c]quinolin-5(4a*H*)-one (S2)

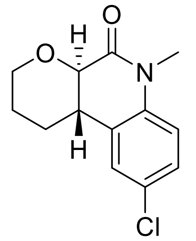

Prepared from *N*-(4-chlorophenyl)-*N*-methyl-3,4-dihydro-2*H*-pyran-6-carboxamide (**S2a**) (25.2 mg, 0.100 mmol) following **General Procedure C**. Purification by flash column chromatography (1:1, EtOAc: pentane) afforded (4a*R*,10b*S*)-9-chloro-6-methyl-2,3,6,10b-tetrahydro-1*H*-pyrano[2,3-c]quinolin-5(4a*H*)-one (**S2**) (24.5 mg, 97%, >20:1 dr, 94:6 er) as a yellow oil.

IR (film) ν_{max}/cm^{-1}: 2980, 2971, 1692, 1491, 1417, 1374, 1252, 1113.

^{1}H NMR (400 MHz, $CDCl_3$) δ = 7.29–7.23 (m, 1H), 7.19–7.10 (m, 1H), 6.91 (d, *J* = 8.6 Hz, 1H), 4.34–4.22 (m, 1H), 3.68 (d, *J* = 13.1 Hz, 1H), 3.49 (ddd, *J* = 11.7, 11.7, 3.1 Hz, 1H), 3.37 (s, 3H), 2.86 (ddd, *J* = 14.4, 11.6, 1.2 Hz, 1H), 2.55–2.40 (m, 1H), 1.95–1.74 (m, 2H), 1.71–1.51 (m, 1H).

^{13}C NMR (101 MHz, $CDCl_3$) δ = 168.1, 138.0, 129.1, 129.0, 128.0, 125.1, 116.2, 75.8, 68.4, 36.5, 30.3, 26.2, 24.8.

HRMS (ESI$^+$) $C_{13}H_{14}NO_2Cl$ $[M+H]^+$ requires 252.0786; found 252.0787, Δ 0.3 ppm.

Chiral HPLC: (Chiralpak OD-H, 30% ^{i}PrOH, 70% hexane, 1.0 mL min^{-1}, λ = 260 nm) τ_R (major) = 14.7 min, τ_R (minor) = 21.0 min; er 94:6.

$[\alpha]_D^{25}$ = +121.6 (*c* = 1.00, $CHCl_3$)

(5a*R*,11b*S*)-7-Methyl-1,2,3,4,7,11b-hexahydrooxepino[2,3-*c*]quinolin-6(5a*H*)-one (S3)

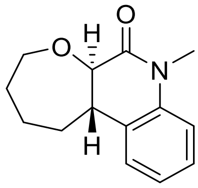

Prepared from *N*-methyl-*N*-phenyl-4,5,6,7-tetrahydrooxepine-2-carboxamide (**S3a**) (23. mg, 0.100 mmol) following **General Procedure C** using Ir(Fppy)$_3$ (1 mg, 0.001 mmol, 1 mol%). Purification by flash chromatography (3:7, EtOAc: pentane) afforded (5a*R*,11b*S*)-7-methyl-1,2,3,4,7,11b-hexahydrooxepino[2,3-*c*]quinolin-6(5a*H*)-one (**S3**) (5.5 mg, 24%, >20:1 dr) as a yellow oil.

IR (film) ν_{max}/cm^{-1}: 2927, 2857, 1685, 1602, 1559, 1541, 1497, 1458, 1419, 1352, 1263, 1127, 1051, 913, 740, 682, 669, 657, 618.

^{1}H NMR (600 MHz, $CDCl_3$) δ = 7.30 (ddt, *J* = 10.2, 7.8, 1.3 Hz, 2H), 7.10 (td, *J* = 7.6, 1.2 Hz, 1H), 7.02 – 6.95 (m, 1H), 4.08 (ddd, *J* = 12.4, 6.2, 4.7 Hz, 1H), 3.84 (d, *J* = 13.7 Hz, 1H), 3.80 – 3.73 (m, 1H), 3.39 (s, 3H), 3.08 (ddd, *J* = 13.4, 9.4, 3.7 Hz, 1H), 2.49 – 2.42 (m, 1H), 2.03 – 1.91 (m, 2H), 1.85 – 1.77 (m, 1H), 1.76 – 1.64 (m, 2H).

^{13}C NMR (151 MHz, $CDCl_3$) δ = 170.3, 139.6, 128.7, 127.7, 124.3, 123.4, 115.0, 80.0, 77.2, 77.0, 76.8, 69.2, 41.8, 30.3, 30.2, 27.7, 24.3.

HRMS (ESI$^+$): $C_{14}H_{18}NO_2$ $[M+H]^+$ requires 232.1332; found 232.1333, Δ 0.2 ppm.

Chiral HPLC: (Chiralpak IG-3, 25% ^{i}PrOH, 75% hexane, 1.0 mL min^{-1}, λ = 210 nm) τ_R (major) = 17.7 min, τ_R (minor) = 16.5 min; er 87:13.

$[\alpha]_D^{25}$ = +12.2 (*c* = 0.10, $CHCl_3$).

3.6. Unsuccessful Substrates

Failed Substrates (low or no conversion or decomposition)

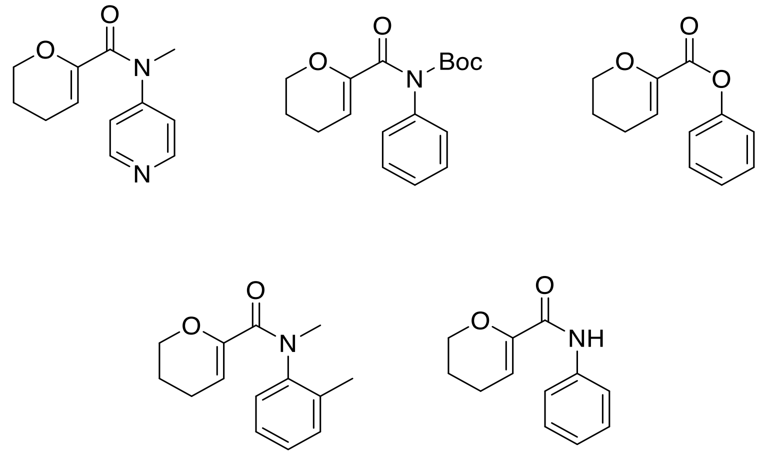

Unselective Substrates

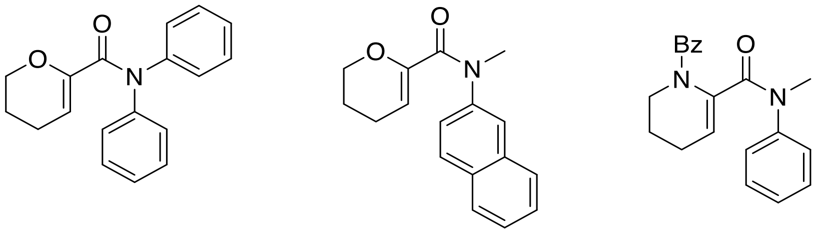

4. Characterisation of Novel Photocatalysts

4.1. Absorbance and Emission Spectra of Novel Photocatalysts

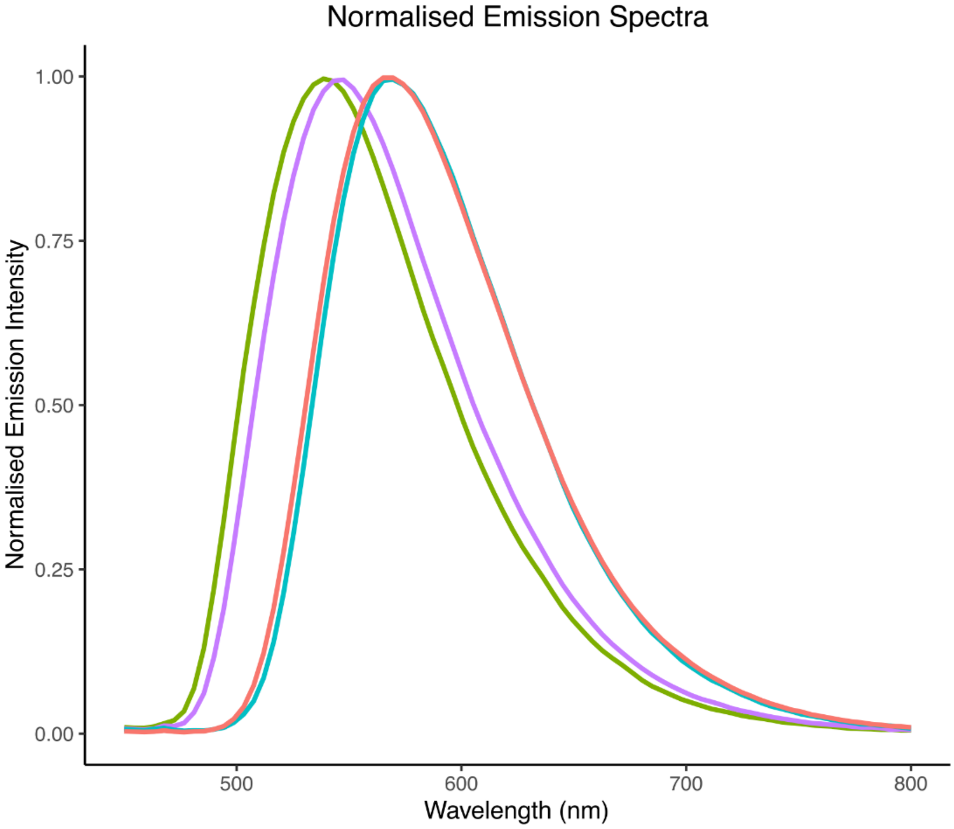

Figure S3: Emission spectra of Ir((5-F)ppy)$_3$ (**C1**) (green), Ir((5-F)(4'-*t*-Bu)ppy)$_3$ (**C2**) (purple), Ir((5-CF_3)ppy)$_3$ (**C3**) (red) and Ir((5-CF_3)(4'-*t*-Bu)ppy)$_3$ (**4**) (blue).

Photocatalyst	λ_{max} (nm)	$E_{T\ (\lambda max)}$ (kcal/mol)	$\lambda_{10\%}$ (nm)	$E_{T(\lambda 10\%)}$ (kcal/mol)
Ir((5-F)ppy)$_3$ (**C1**)	541	52.8	480	59.6
Ir((5-F,4'-*t*-Bu)ppy)$_3$ (**C2**)	547	52.3	486	58.8
Ir((5-CF_3)ppy)$_3$ (**C3**)	565	50.6	502	57.0
Ir((5-CF_3, 4'- *t*-Bu)ppy)$_3$ (**4**)	567	50.4	513	55.7

Table S3: Emission values and corresponding triplet energies of photocatalysts **C1-3** and **4**.

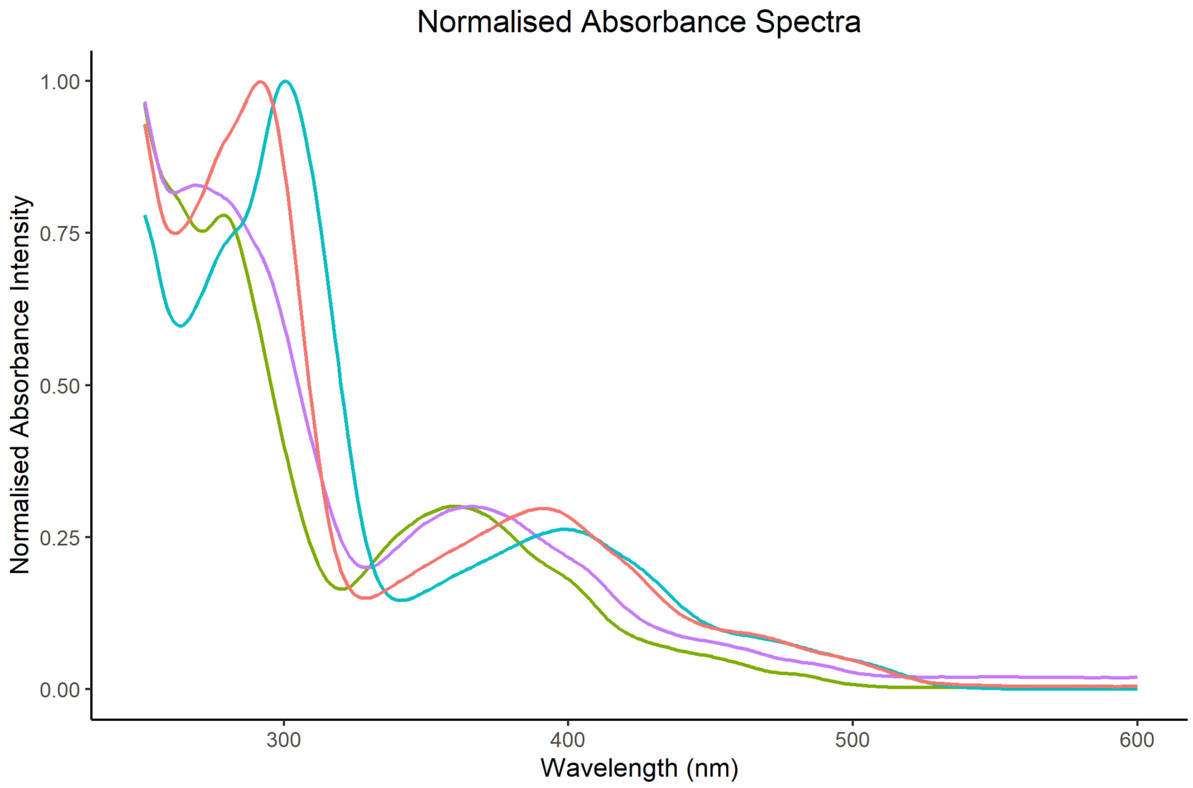

Figure S3.1: Absorbance spectra of Ir((5-F)ppy)$_3$ (**C1**) (green), Ir((5-F)(4'-t-Bu)ppy)$_3$ (**C2**) (purple), Ir((5-CF$_3$)ppy)$_3$ (**C3**) (red) and Ir((5-CF$_3$)(4'-t-Bu)ppy)$_3$ (**4**) (blue).

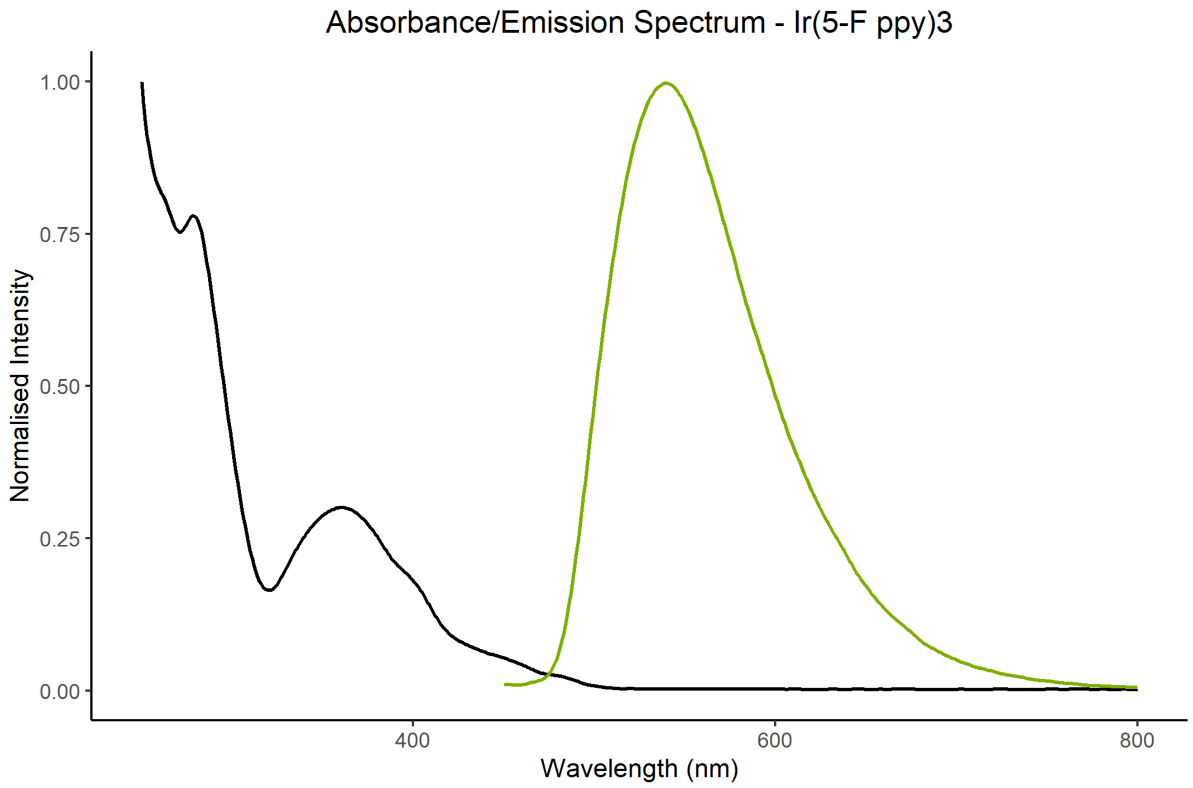

Figure S4: Absorbance (black) and emission (green) spectra of Ir((5-F)ppy)$_3$ (**C1**).

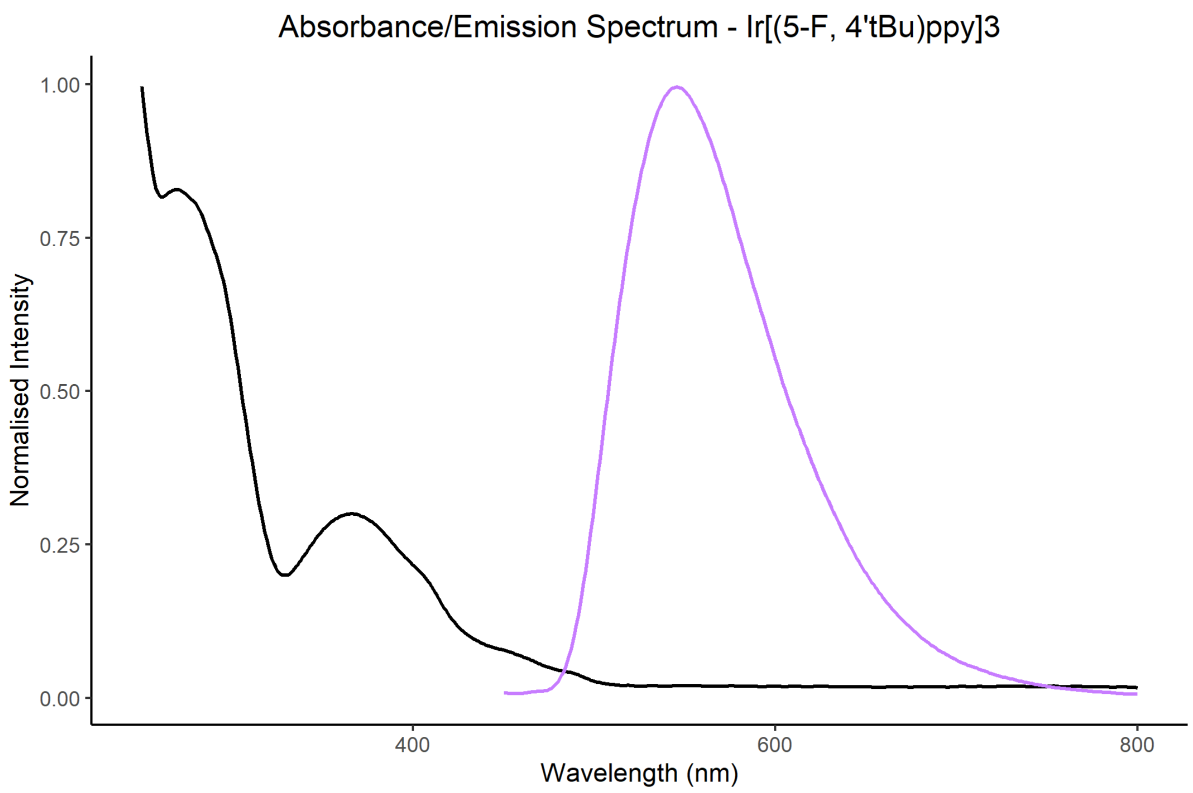

Figure S5: Absorbance (black) and emission (lilac) spectra of Ir((5-F)(4'-*t*-Bu)ppy)$_3$ (**C2**).

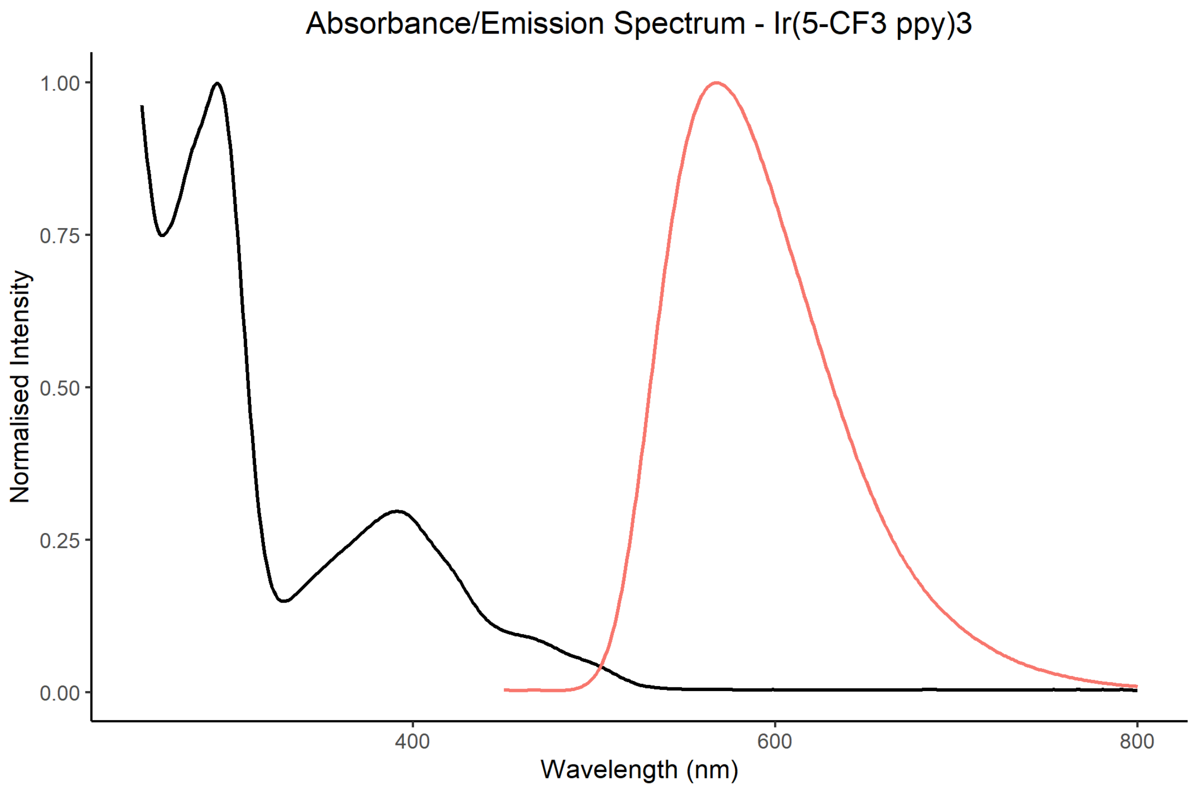

Figure S6: Absorbance (black) and emission (orange) spectra of Ir((5-CF$_3$)ppy)$_3$ (**C3**).

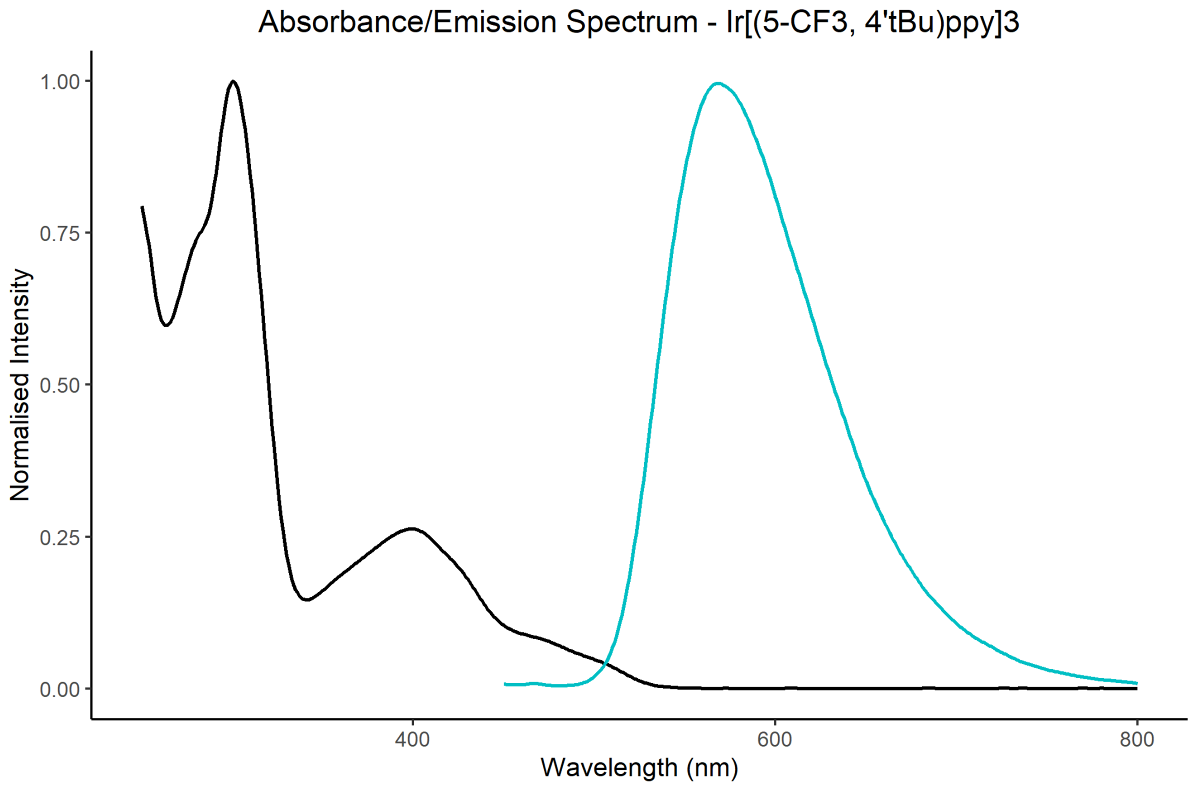

Figure S7: Absorbance (black) and emission (blue) spectra of Ir((5-CF_3)(4'-*t*-Bu)ppy)$_3$ (**4**).

4.2. Square-Wave Voltammograms of Novel Photocatalysts

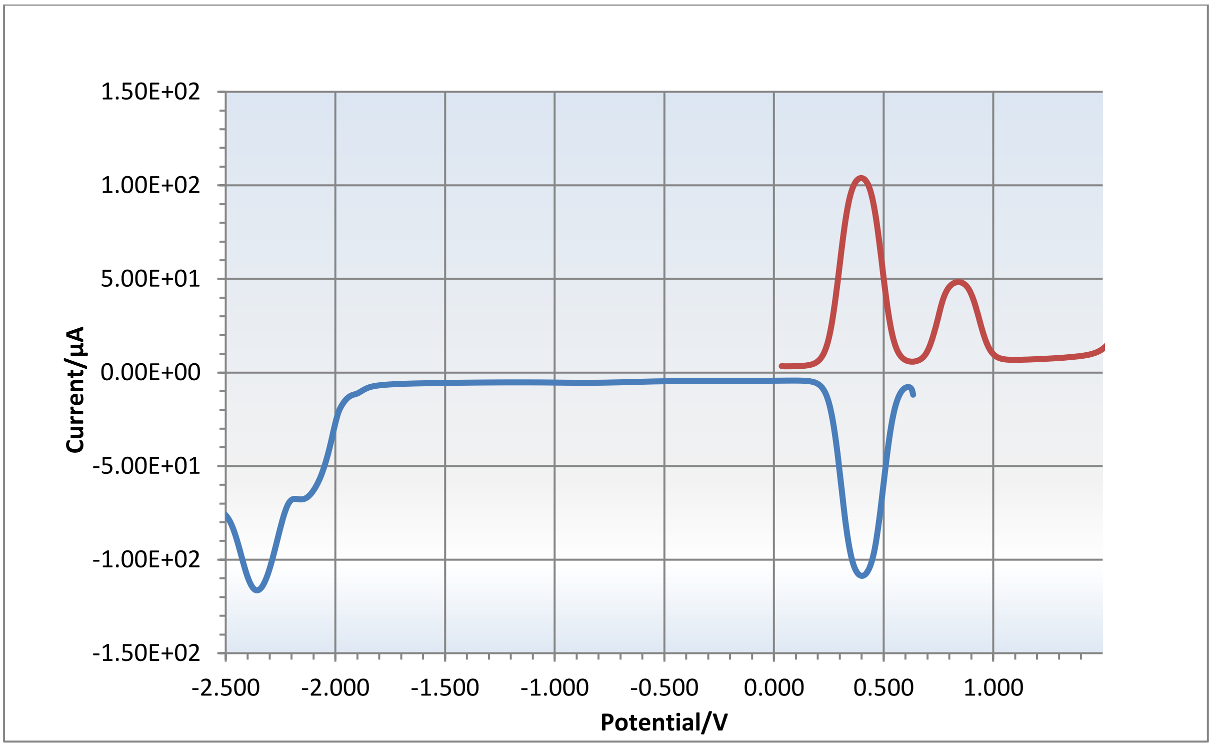

Figure S8: Square-wave voltammogram of Ir((5-F)ppy)$_3$ (**C1**) in MeCN.

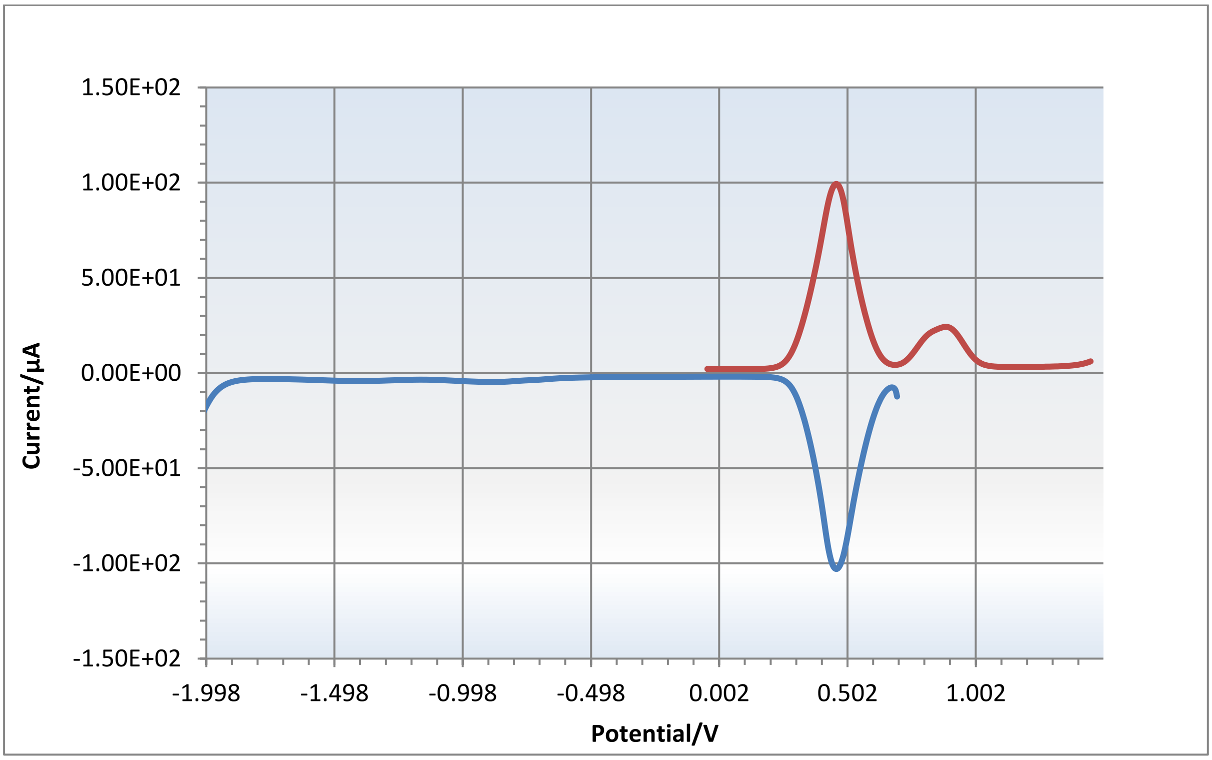

Figure S9: Square-wave voltammogram of Ir((5-F)ppy)$_3$ (**C1**) in CH_2Cl_2.

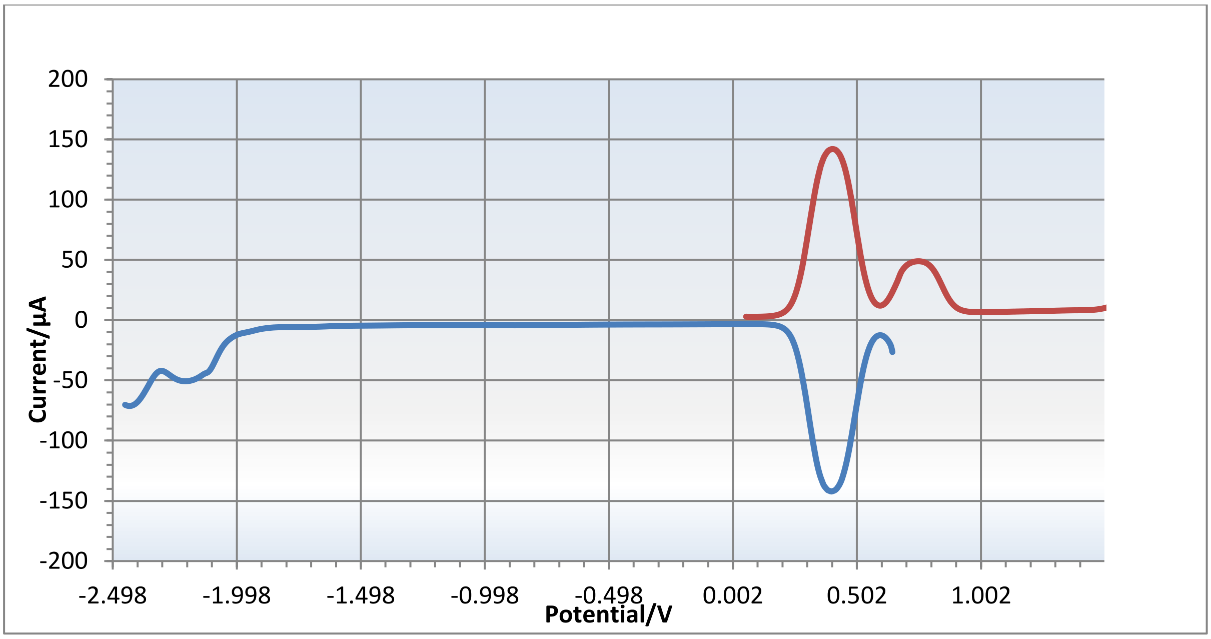

Figure S10: Square-wave voltammogram of Ir((5-F)(4'-*t*-Bu)ppy)$_3$ (**C2**) in MeCN.

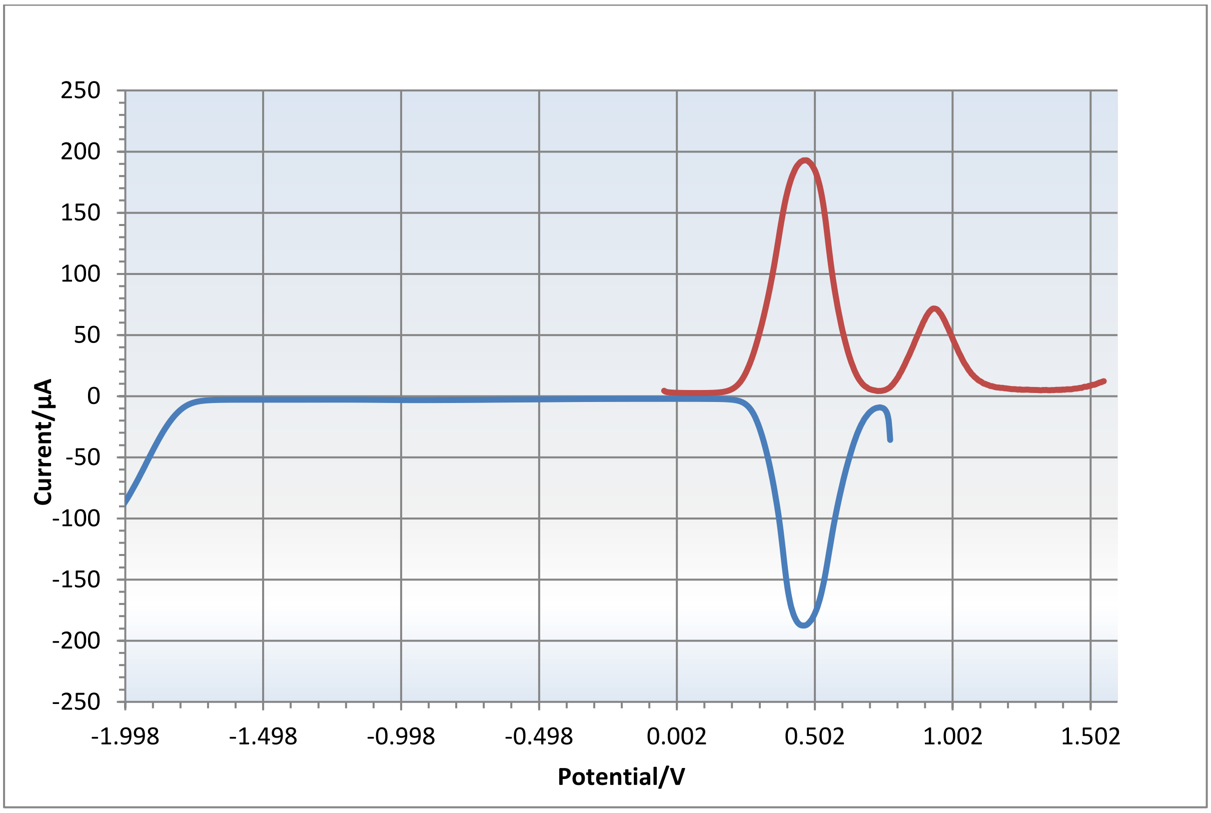

Figure S11: Square-wave voltammogram of Ir((5-F)(4'-*t*-Bu)ppy)$_3$ (**C2**) in CH_2Cl_2.

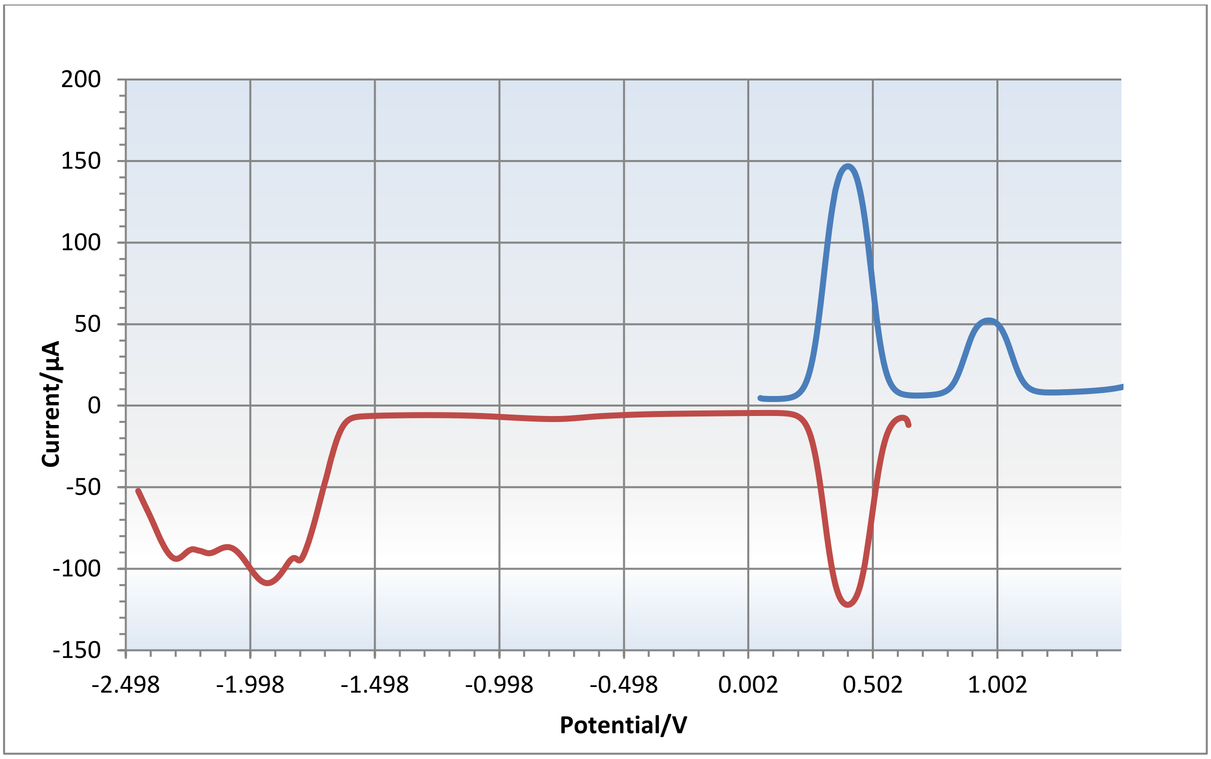

Figure S12: Square-wave voltammogram of Ir((5-CF_3)ppy)$_3$ (**C3**) in MeCN.

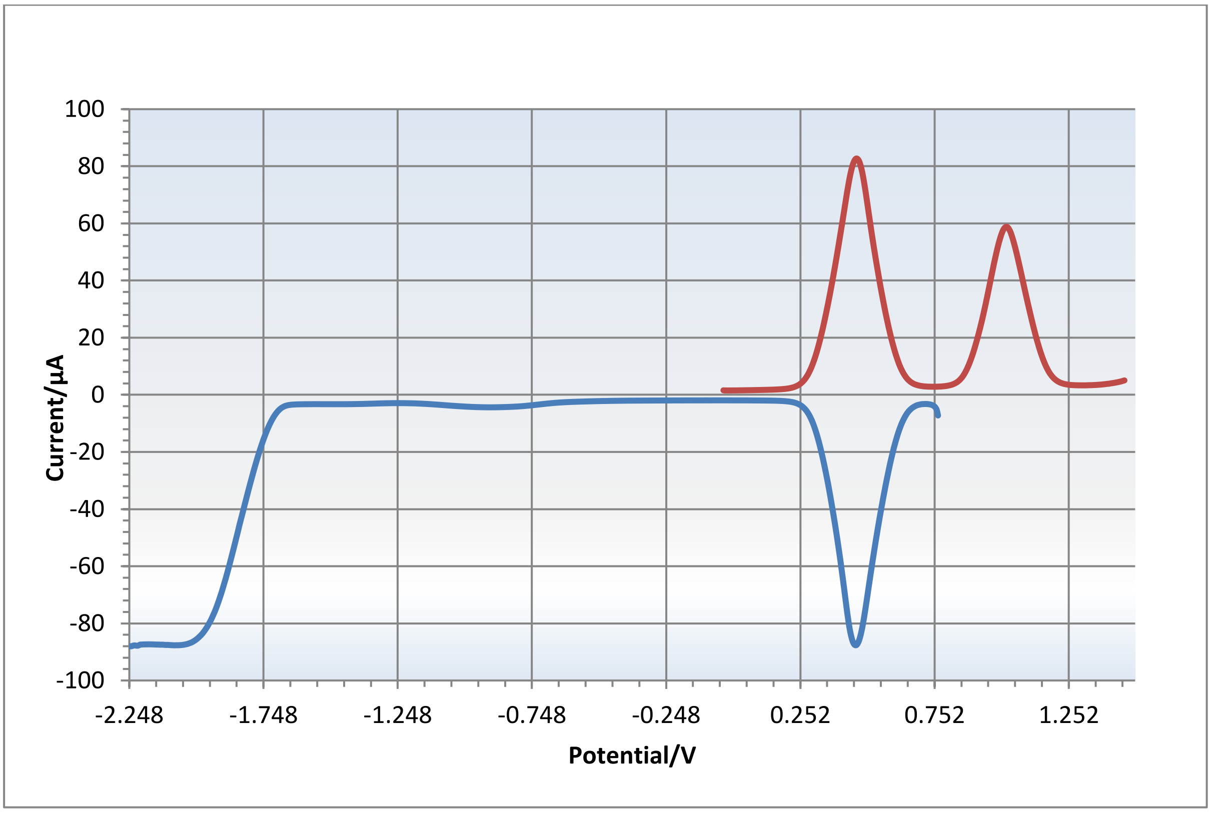

Figure S13: Square-wave voltammogram of Ir((5-CF_3)ppy)$_3$ (**C3**) in CH_2Cl_2.

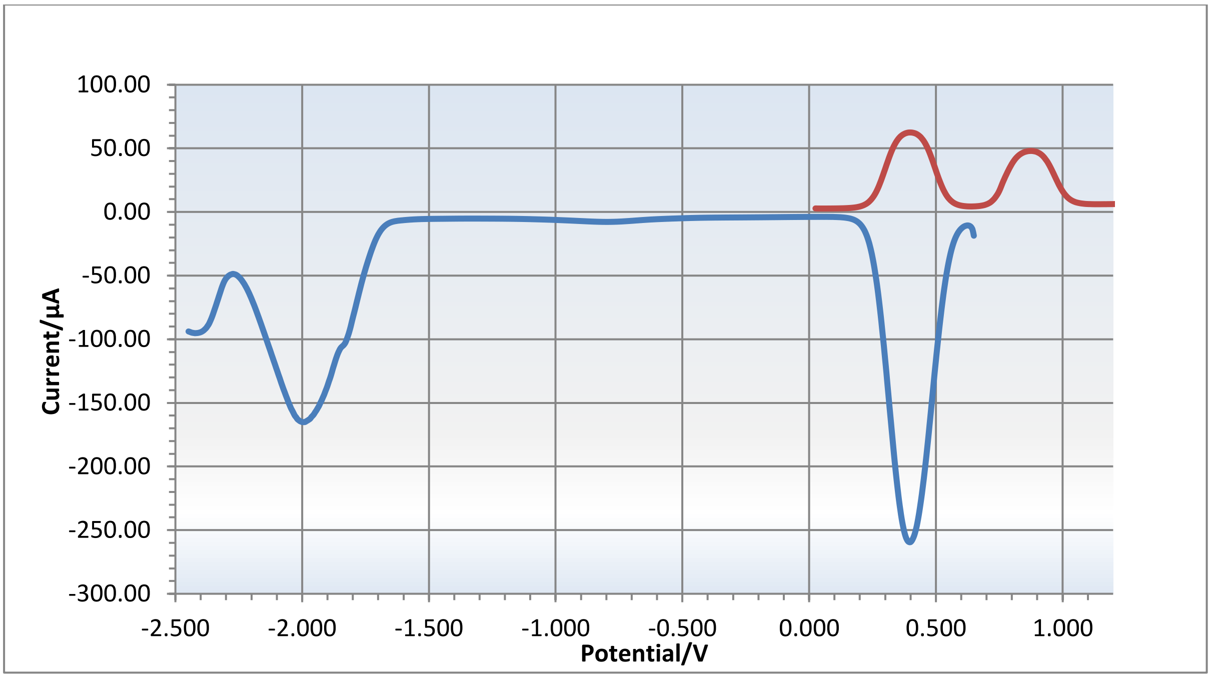

Figure S14: Square-wave voltammogram of Ir((5-CF_3)(4'-*t*-Bu)ppy)$_3$ (**4**) in MeCN.

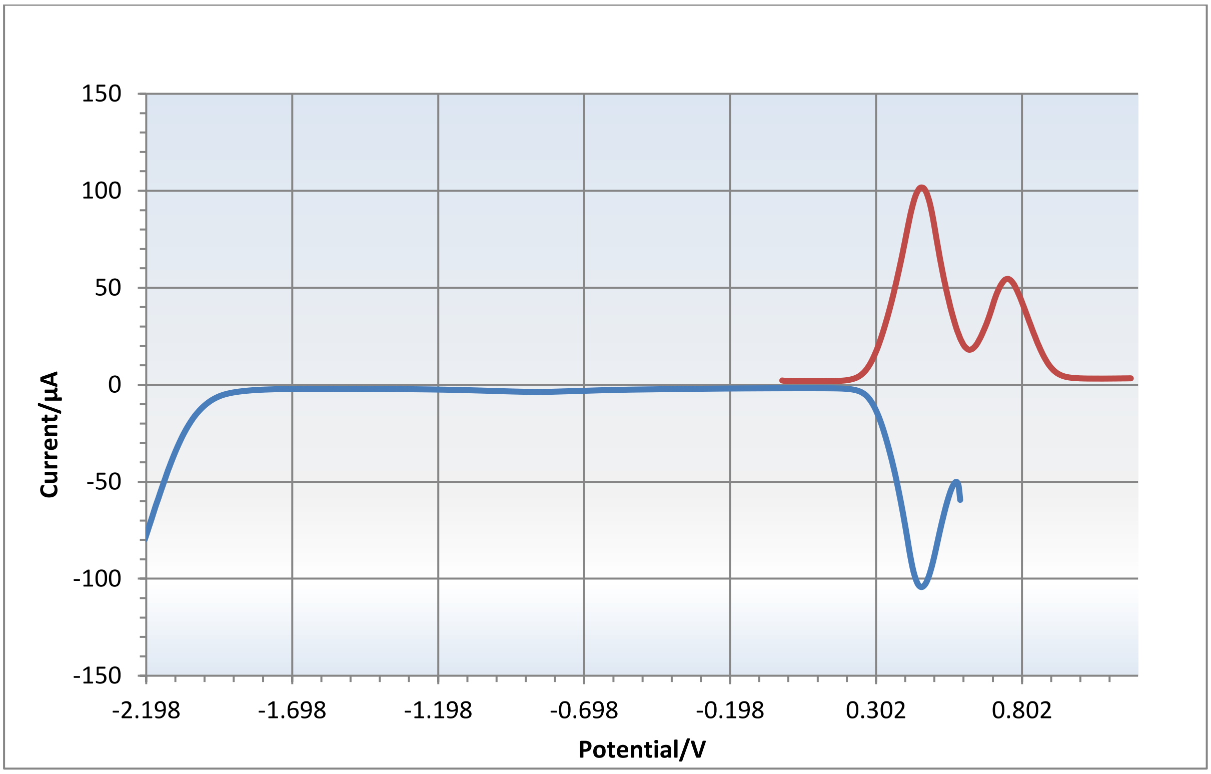

Figure S15: Square-wave voltammogram of Ir((5-CF_3)(4'-*t*-Bu)ppy)$_3$ (**4**) in CH_2Cl_2.

4.3. Estimation of Excited State Redox Potentials for Novel Photocatalysts

To be able to compare the properties of different photocatalysts, all excited state redox properties were calculated from ground state redox potentials in acetonitrile and λ (10%) values which were either recorded or available in the literature.[2,6-8]

The excited state redox properties were calculated using the Rehm-Weller equations:[5]

$$E_{1/2}^{red}\ Ir(IV)/{}^*(III) = E_{1/2}^{red}\ Ir(IV)/(III) - E^{0-0}$$

$$E_{1/2}^{red}\ Ir^*(III)/(II) = E_{1/2}^{red}\ Ir(III)/(II) + E^{0-0}$$

Where E^{0-0} is the energy gap between the zero-level vibrational levels of the ground and excited state; estimated as the high-energy onset of phosphorescence where the emission intensity is 10% of the observed maximum.

4.4. Summary of Photocatalyst Properties

Photocatalyst	$E_{1/2}^{red}$ vs. SCE in CH_2Cl_2 (V)		$E_{1/2}^{red}$ vs. SCE in MeCN (V)				Emission in MeCN (nm)		E_T (kcal/mol)	
	$Ir^{IV/III}$	$Ir^{IV/*III}$	$Ir^{IV/III}$	$Ir^{IV/*III}$	$Ir^{III/II}$	$Ir^{*III/II}$	λ (10%)*	λ (max)	λ (10%)	λ (max)
$Ir(ppy)_3$ [1]	- 0.69	- 1.88	- 0.77	-1.81	-2.23	+ 0.35	481	516	59.4	55.4
$Ir((4'\text{-}^tBu)ppy)_3$ [2]	- 0.60	- 1.97	- 0.66	-1.90	-2.33	+ 0.23	485	525	59.0	54.5
$Ir(Fppy)_3$ [1]	N/A	N/A	- 0.96	-1.74	-2.18	+ 0.52	459	488	62.3	58.6
$Ir(dFppy)_3$ [1]	N/A	N/A	- 0.94	-1.83	-1.87	+ 0.90	448	476	63.8	60.0
$Ir(dF(CF_3)ppy)_2(dtbbpy)(PF_6)$ [1]	N/A	N/A	- 1.73	-1.01	-1.40	+ 1.34	452	473	63.3	60.0
$Ir(dFppy)_2(dtbbpy)(PF_6)$ [1]	N/A	N/A	- 1.59	-1.15	-1.47	+ 1.27	452	516	63.3	55.4
$Ir(Fppy)_2(dtbbpy)(PF_6)$ [1]	N/A	N/A	- 1.45	-1.19	-1.50	+ 1.14	469	540	61.0	52.9
$Ir(ppy)_2(dtbbpy)(PF_6)$ [6,7]	N/A	N/A	- 1.33	-1.32	-1.34	+ 0.83	500	570	56.4	48.3
$Ir((3'\text{-}OMe)ppy)_3$ [2]	- 0.70	- 1.95	- 0.76	-1.89	-2.34	+ 0.26	517	557	55.3	51.3
$Ir((5\text{-}F)ppy)_3$ **C1**	- 0.88	- 1.70	- 0.84	-1.74	-2.17	+ 0.41	480	541	59.6	52.8
$Ir((5\text{-}F)(4'\text{-}^tBu)ppy)_3$ **C2**	- 0.75	- 1.80	- 0.75	-1.80	-2.22	+ 0.33	486	547	58.8	52.3
$Ir((5\text{-}CF_3)ppy)_3$ **C3**	- 1.02	- 1.45	- 0.97	-1.50	-1.80	+ 0.67	502	565	57.0	50.6
$Ir((5\text{-}CF_3)(4'\text{-}^tBu)ppy)_3$ **4**	- 0.93	- 1.49	- 0.87	-1.55	-1.83	+0.59	513	567	55.7	50.4

Table S4: Summary of photocatalyst properties.

* Where literature values are cited, these values were obtained by graphical extraction from published emission spectra.

5. Mechanistic Experiments and Photophysics

5.1. Labelling and Crossover Experiments

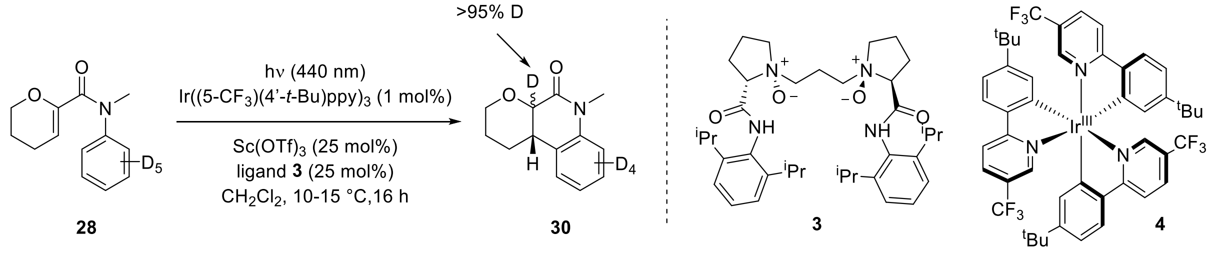

Figure S16: Deuterium labelling experiment to probe the proposed 1,5-H shift.

The photocyclization of pentadeuterated substrate (**28**) was performed according to **General Procedure C**. The crude reaction mixture was analysed by ^{1}H NMR and LC/MS to determine the degree of deuterium transfer from the aniline *ortho*-positions to the α-carbonyl position. Results indicate >95% incorporation of deuterium at the C17 position, consistent with a intramolecular 1,5-H-shift.

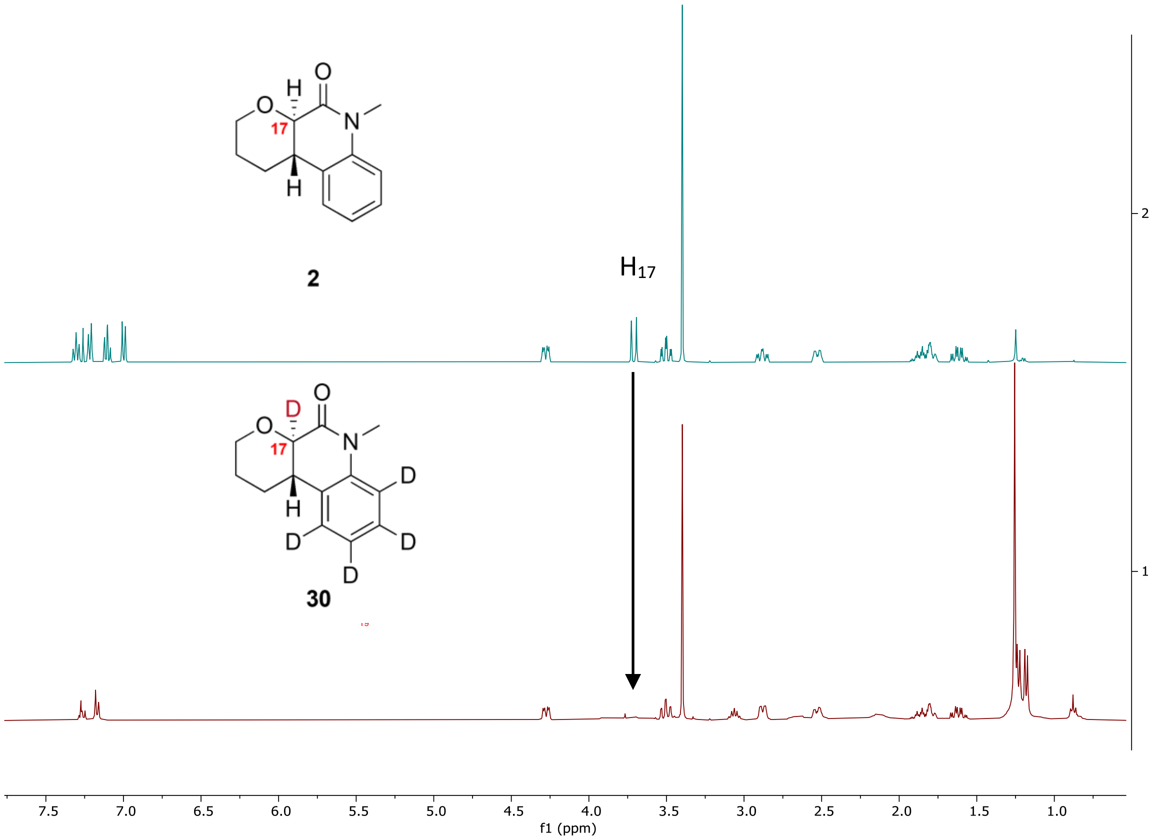

Figure S17: Top, purified ^{1}H NMR spectrum of (4a*R*,10b*S*)-6-methyl-2,3,6,10b-tetrahydro-1*H*-pyrano[2,3-c]quinolin-5(4a*H*)-one (**2**) for comparison. Bottom, Crude ^{1}H NMR spectrum of the product of the cyclization (**30**) using the corresponding -d_5 substrate (**28**).

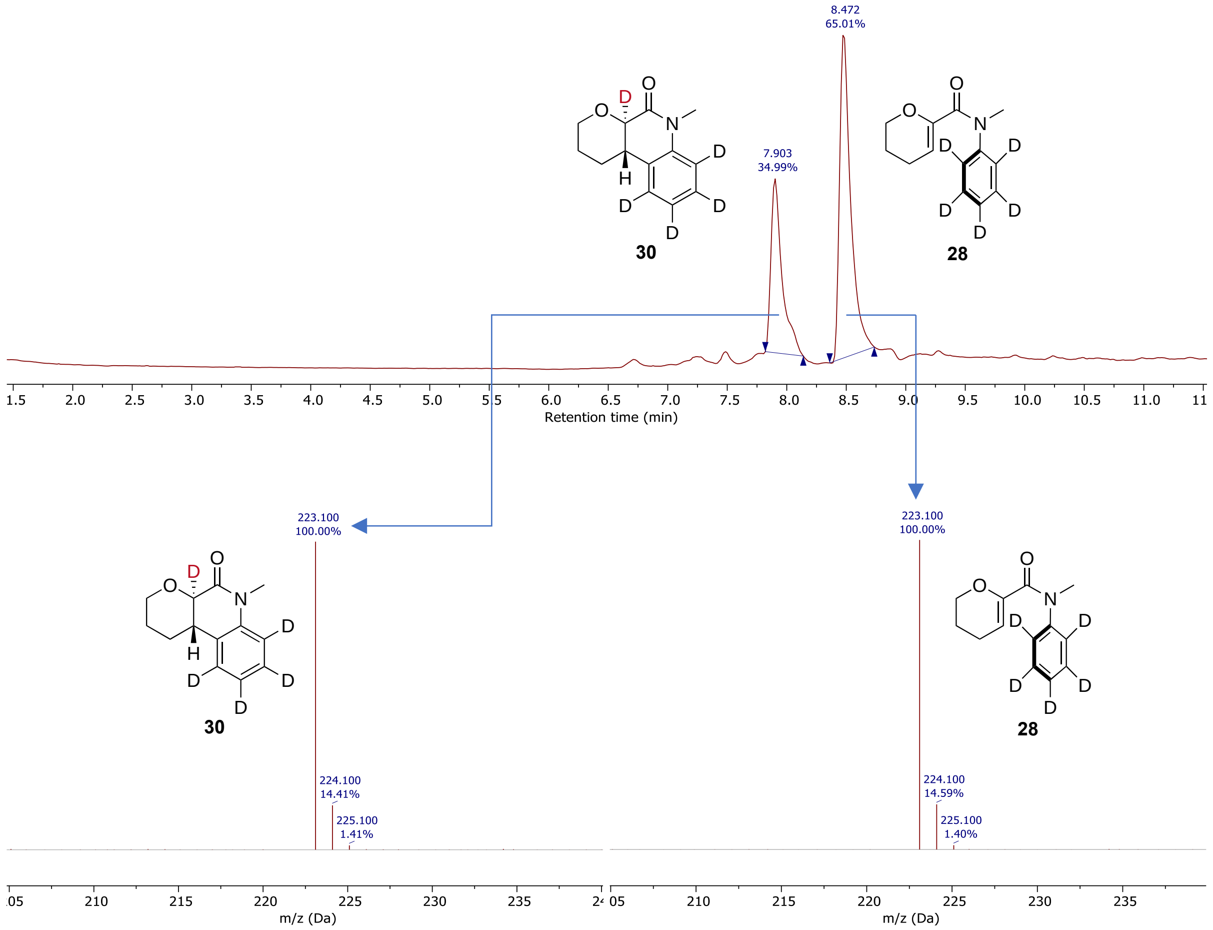

Figure S18: LC/MS (ESI) trace of crude reaction mixture of d_5-substrate at ≈ 60% conversion showing deuterated SM **28** and deuterated product **30**. Top: total ion chromatograph. Bottom: API-ES MS analysis of each component shows no evidence of deuterium loss in **28** or **30**.

Crossover Experiments

Crossover experiments were carried out to investigate the possibility of intermolecular hydrogen transfer. LC/MS analysis of the crude reaction mixtures was performed to separate the reaction components and determine their isotopic distribution. The identities of each (LC/MS) chromatogram peak were confirmed by separation of reaction components by flash column chromatography followed by re-analysis under the same separation conditions used to determine their retention times. Purified and separated products were also analysed by quantitative ^{1}H NMR (d1 = 60 s) to determine the extent of crossover.

In all cases, no exchange of deuterium between substrates and products was observed, ruling out the possibility of intermolecular proton transfer.

Crossover Experiment 1: crossover of *N*-methyl pentadeuterated substrate (28) and *N*-propyl substrate (29).

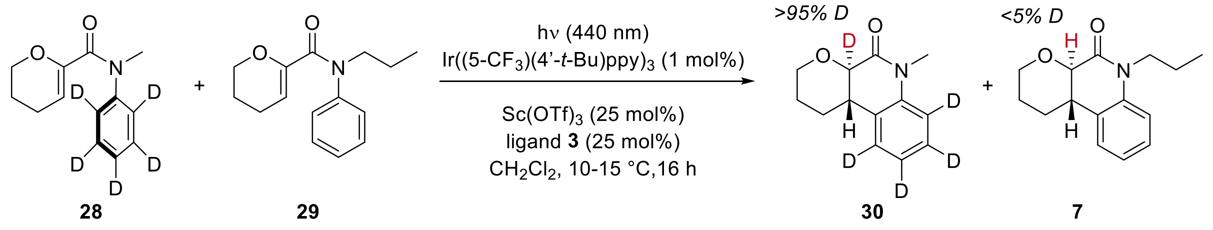

This experiment was carried out with **28** (0.5 mmol), and **29** (0.5 mmol) according to **General Procedure C** and the crude reaction mixture analysed before reaction completion.

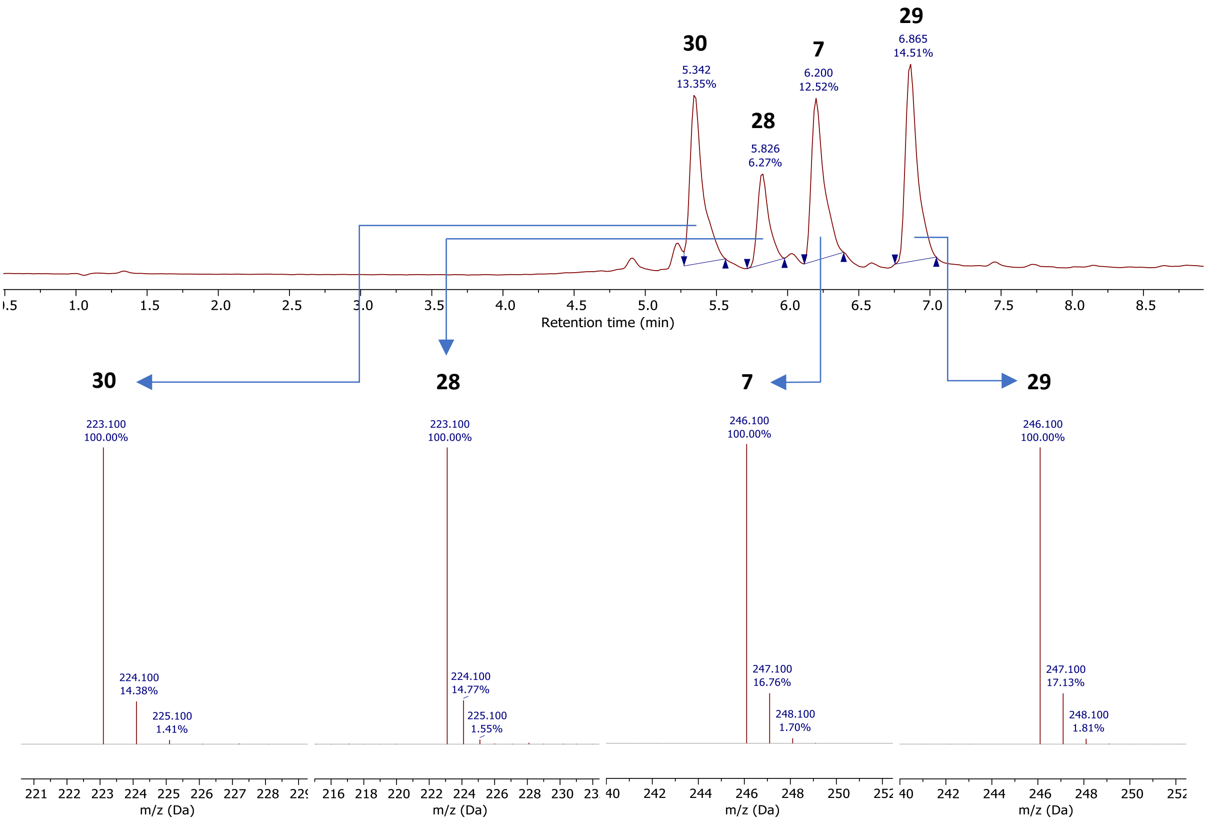

Figure S19: LC/MS analysis of crude crossover reaction mixture of d_5-substrate (**28**), and non-deuterated substrate (**29**) at ≈60% conversion to products **30** and **7**. Top: total ion chromatograph. Bottom: API-ES MS analysis of each component. No evidence for crossover was observed.

Crossover Experiment 2: crossover of *N*-methyl pentadeuterated substrate (28) and *N*-isobutyl substrate (29).

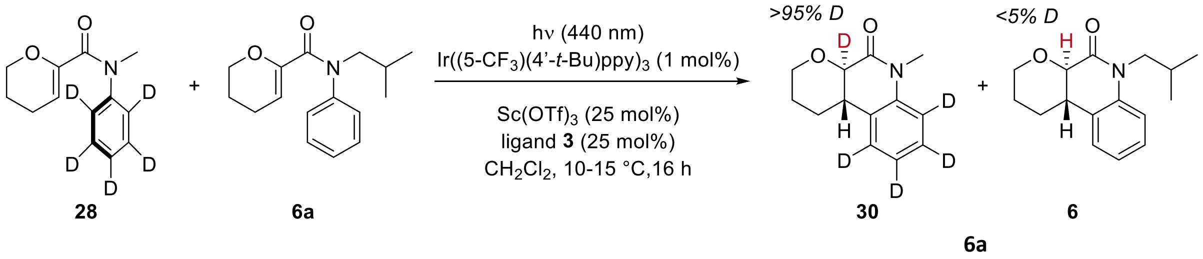

This experiment was carried out with **28** (0.5 mmol), and **6a** (0.5 mmol) according to **General Procedure C** and the crude reaction mixture analysed before reaction completion.

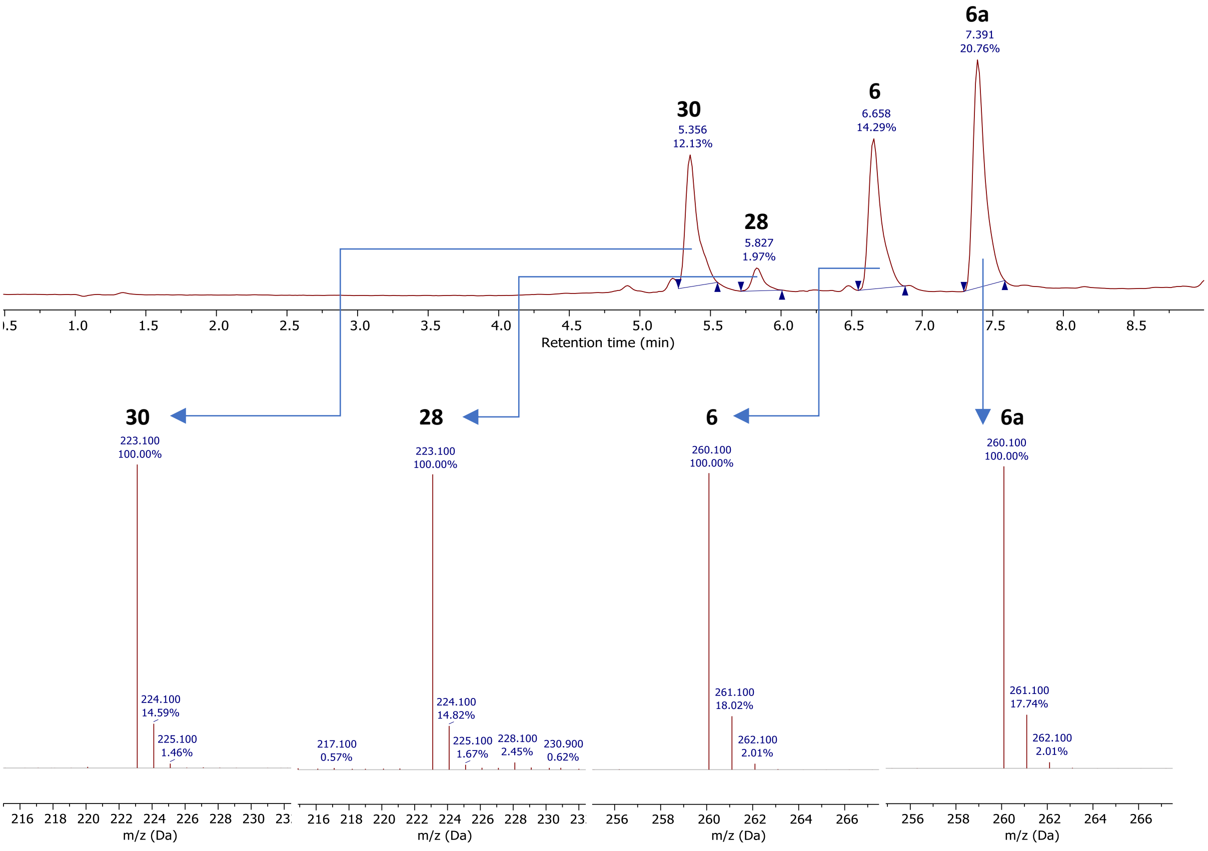

Figure S20: LC/MS analysis of crude crossover reaction mixture of d_5-substrate (**28**), and non-deuterated substrate (**6a**) at ≈90% conversion to products **30** and **6**. Top: total ion chromatograph. Bottom: API-ES MS analysis of each component. No evidence for crossover was observed.

Probing the diastereoselectivity for *meta*-substituted substrates: Exogenous deuterium incorporation study

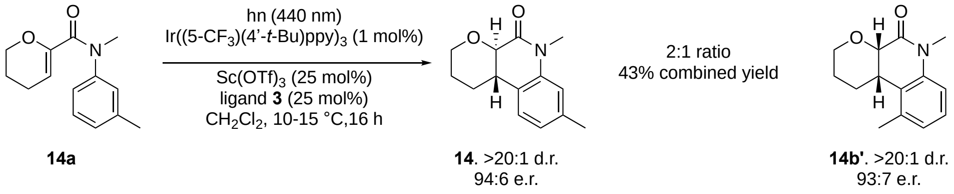

Figure S21. Cyclization of *meta*-substituted substrate leads to an otherwise unobserved *cis*- product

Cyclization of *meta*-substituted substrates occurs to give a mixture of 2 regioisomers. For the regioisomer where the substituent is proximal to the THP ring, the product is formed as a diastereoisomeric mixture containing the (otherwise unobserved) *cis*-isomer (Figure S21).

It was proposed that the formation of the major (*cis*-fused) diastereomer of the minor regioisomer **14b'** (fusion at the aniline 2-position) was due to a reaction pathway whereby intermediate **Db** could rearomatize to form **Db1**, followed by *intermolecular* protonation to form the *cis*-fused product, **14b'** (Figure S22).

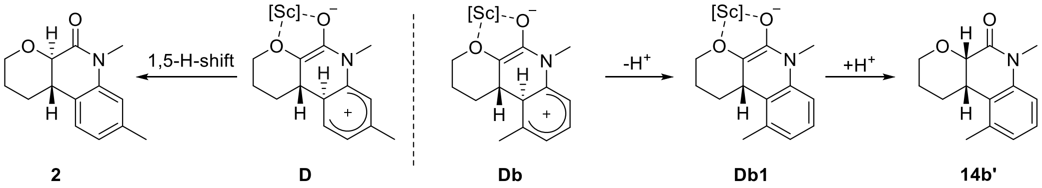

Figure S22. Left: the calculated mechanism for formation of the major diastereomer (*trans*-fused) of the major regioisomer (cyclisation to the aniline 6-position). Right: proposed mechanism for the formation of the major diastereomer (*cis*-fused) of the minor regioisomer (cyclisation to the aniline 2-position).

This contrasts with the *intramolecular* 1,5-H-shift predicted to be operating normally (**D** → **2**). If this deprotonation/protonation mechanism is in operation, protonation should be possible from an external H/D source whilst in the case of a 1,5-H-shift, this is unlikely.

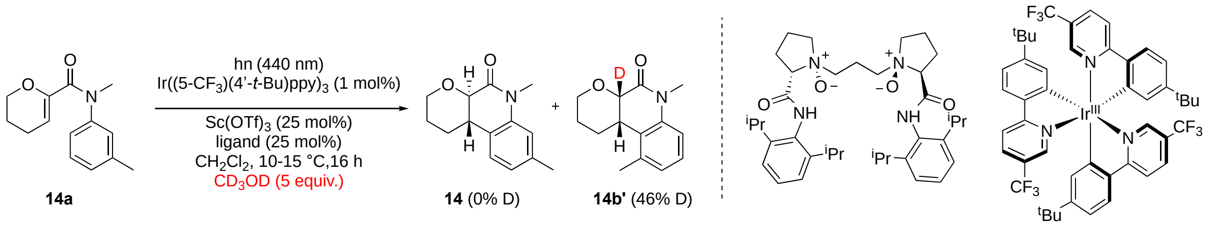

Figure S23. Inclusion of exogeneous D and H sources to probe the potential for a protonation/deprotonation mechanism in the formation of *cis*-fused diastereomers.

To probe the mechanism of formation for the *cis*-diastereomer, photocyclization of *meta*-methyl substrate **14a** was performed with 5 equiv. MeOH-*d*4 present in the reaction (Figure S23).

To determine a baseline/control for the reaction in the presence of methanol-*d*4, the reaction of **14a** was duplicated, but with the addition of 5 equiv. methanol. A control reaction of substrate **1** as an exemplar *trans*-selective cyclisation in the presence of 5 equiv. MeOH-*d*4 was also carried out.

The reaction components were separated by preparative TLC and analysed by LC/MS to determine the degree of deuterium incorporation (as determined by comparative abundance of M+1 peak by LC/MS analysis).

No deuterium incorporation was observed in the C6-linked *trans*-diastereomer **14**. However, 46% incorporation of D was observed in the C2-linked *cis*-diastereomer **14b'** (an M+1 peak intensity of 98.5% corresponds to 46%[†] deuterium incorporation; see Table S5). This finding is consistent with the proposal that the *cis*-diastereomer may not form via a 1,5-H shift, but instead by an alternative intermolecular deprotonation-reprotonation mechanism

**Substrate**		**14a**	**14a**	**1**
**Additive (5.0 equiv.)**		CH_3OH	CD_3OD	CD_3OD
**Natural M+1 Abundance $[M+1+H]^+$ (%)**		15.1		14.1
**M+1 Abundance (%)**	*Cis*-fused (C2-linked)	15.0	98.5	-
	Trans-fused (C6-linked)	15.5	17.7	14.5

Table S5. Tabulation of relative abundance of M+1 peak in LC/MS

These measurements indicate that within experimental error, deuterium incorporation only occurs in the *cis*-fused product. This observation is in line with observations made on similar systems.[9]

[†] Isotopic enrichment was calculated by subtracting the baseline M+1+H abundance from the measured M+1+H and expressing this as a percentage of the combined M+H and true M+1+H abundances. $\frac{98.5-15.0}{100+(98.5-15.0)} = 45.5\%$

5.2. Chemical Actinometry and Quantum Yield Measurement

Chemical Actinometry[8, 10,11]

To determine the quantum yield of the optimised reaction, a 430 nm LED (Thorlabs M430L5) was calibrated by ferric oxalate actinometry as follows:

3 mL of a freshly prepared solution of ferric oxalate (0.006 M) in aqueous H_2SO_4 (0.05 M) was placed in a quartz cuvette and irradiated for 8 seconds under stirring. This was carried out in triplicate, and an identical control sample was kept in the dark. A sample of each of the irradiated and control samples (0.30 mL) were transferred to vials containing 1.35 mL of a 0.1% phenanthroline solution (13.56 g CH_3COONa and 0.10 g phenanthroline in 100 mL 0.5 M H_2SO_4) and 1.35 mL H_2O. Samples were kept in the dark for 1 h before their absorbance was measured at 510 nm. The LED irradiation time was chosen to ensure that conversion did not exceed 5% and that absorbance did not exceed 0.5 – 1 ABS.

$$no.moles\ Fe^{2+} = \frac{v_1 v_3 \Delta A(510\ nm)}{10^3 v_2 l \varepsilon(510\ nm)}$$

Equation 1

The number of moles of Fe^{2+} produced from irradiation could be calculated from these measurements according to **Equation 1** where v_1 (mL) is the irradiated volume, v_2 (mL) is the aliquot of the irradiated solution transferred, v_3 (mL) is the final volume of complexation solution, l (cm) is the path length, ΔA is the difference in absorption (at 510 nm) between the irradiated solution and the solution stored in the dark, and ε(510 nm) is the molar absorption coefficient of $Fe(phen)_3^{2+}$ at 510 nm (11,100 M^{-1} cm^{-1}).

$$f\ (E\ s^{-1}) = \frac{no.moles\ Fe^{2+}}{\Phi_R t(1 - 10^{-A})}$$

Equation 2

$$f\ (mW) = 1000\ \times f(E\ s^{-1}) N_A h \frac{c}{\lambda}$$

Equation 3

Using the value calculated, the photon flux can then be calculated (first in E s^{-1} and subsequently in mW) according to the above equations, where Φ_R is the quantum yield of formation of Fe^{2+} in the ferric oxalate actinometer (well known for a range of wavelengths),[12] *t* is the irradiation time (s), A is the absorption of the 0.006 M ferric oxalate solution at the irradiation wavelength, N_A is Avogadro's number, *h* is planck's length (m), *c* is the speed of light (m s^{-1}), and λ is the irradiation wavelength (nm).

Quantum Yield Measurement

Using this methodology, our LED was determined to have an irradiation power of 73 mW using our setup. With the power known, the quantum yield was determined by preparing the reaction solution according to **General Procedure C** using substrate **1.** 3 mL of this solution were transferred to a quartz cuvette which was stirred and thermostatted to 15 °C. After 17 hours the conversion was measured to be 5.6% by rp-HPLC (calibrated using 1,3,5-trimethoxybenzene as an internal standard added after cessation of irradiation and averaged across three wavelengths).

$$\Phi = \frac{no.moles\ \mathbf{2}}{f\ t\ (1 - 10^{-A})} = 0.003$$

Using **Equation 2** and the previously measured photon flux, the quantum yield was determined to be 0.003, which is consistent with a non-chain process.

5.3. Weakly Reducing Photocatalyst Study

To probe whether the reaction was proceeding *via* a photoredox pathway or an energy transfer pathway, the reaction was carried out with a number of photocatalysts with appropriate triplet energies and redox properties.[13]

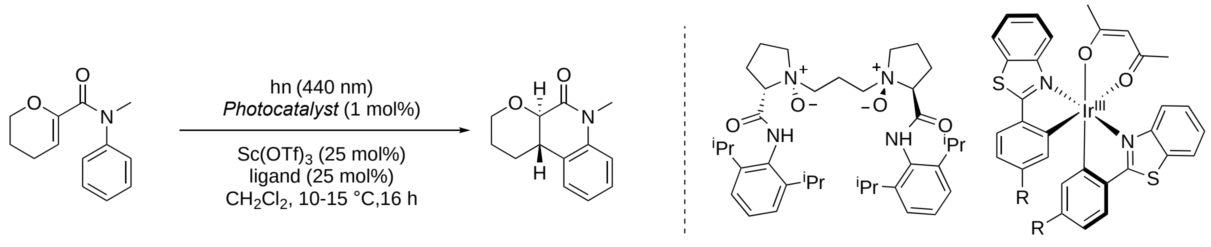

1 2 3 25-27

Photocatalyst	E_T 10%	E_{ox} (Ir^{*III}/Ir^{IV}) (V)	Yield	er
**25** (R = F)	56.3	1.12	33%	76:24
**26** (R = H)	54.8	1.27	15%	79:21
**27** (R = CF_3)	53.6	0.8	8%	88:12

Table S4: Reaction outcomes with weakly reducing photocatalysts **25-27**.

As can be seen from the above, increasing yield appears to correlate with increasing triplet energy, while increasing enantioselectivity appears to correlate with decreasing triplet energy. These observations are in line with expectations for a triplet energy transfer pathway wherein bound substrate (with a marginally lower triplet energy) selectively undergoes triplet energy transfer.

There is no correlation between excited state oxidation potential and yield or enantioselectivity, as could be expected if a selective photoredox pathway were in operation.

5.4. Electrochemical Measurements

Determination of Substrate (1) Redox Potentials and the Effect of $Sc(OTf)_3$

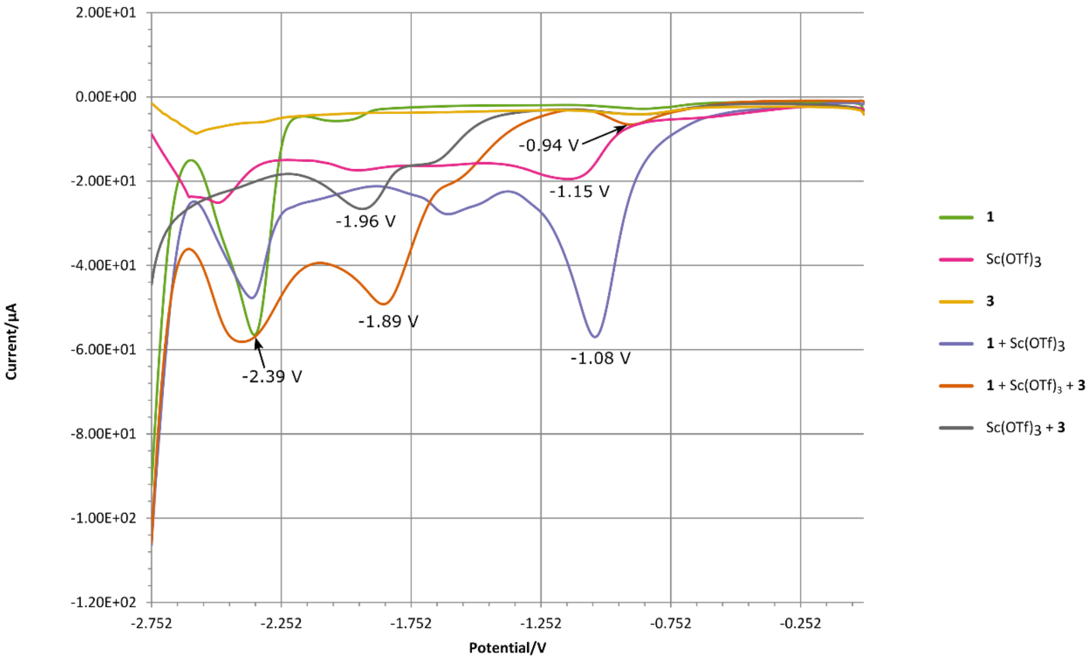

Figure S24: Square-wave voltammograms of reaction substrate (**1**) (green), the effect of $Sc(OTf)_3$ (lilac), $Sc(OTf)_3$ and **3** (orange), and of $Sc(OTf)_3$ alone (pink), **3** alone (yellow), and $Sc(OTf)_3$ and **3** (gray) quoted vs SCE.
Samples were measured at 0.01 M concentration in MeCN with tetra-*N*-butylammonium hexafluorophosphate, Bu_4NPF_6 ($TBAPF_6$) as supporting electrolyte.

5.5. Stern-Volmer Quenching

A stock solution of **4** in argon sparged dichloromethane (25 mL, 0.5 μM) was prepared. Stock solutions of substrate **1** (40 mM in **1**, 0.5 μm in **4**) and scandium + **3** complex (4 mM in complex, 0.5 μm in **4**) were then prepared using this solution. A fourth solution of scandium + **3** complex with **4** was prepared by combining equal volumes of the prepared solutions. A cloudy solution was obtained and so this solution was syringe filtered to yield a solution suitable for fluorometric studies.

For each study an initial emission spectrum of the catalyst solution was recorded. One of the other three solutions was then added in measured volumes and the emission spectrum was rerecorded. If the dopant quenches the excited state photocatalyst, we expect the emission intensity to decrease as the concentration of dopant increases.

The resulting data were plotted according to the Stern-Volmer relationship:

$$\frac{I_0}{I} = k_q[Q] + 1$$

Where I_0 is the emission intensity of the photocatalyst in the absence of quencher, and I is the emission of the photocatalyst at a known quencher concentration, a graph plotting (I0/I) against quencher concentration ([Q]) should give a straight line with y intercept of 1 and a gradient quantifying the rate of quenching (kq).

Addition of scandium + 3 complex

In the addition of scandium complex to the photocatalyst no decrease in emission was recorded even at high concentrations of proposed quencher.

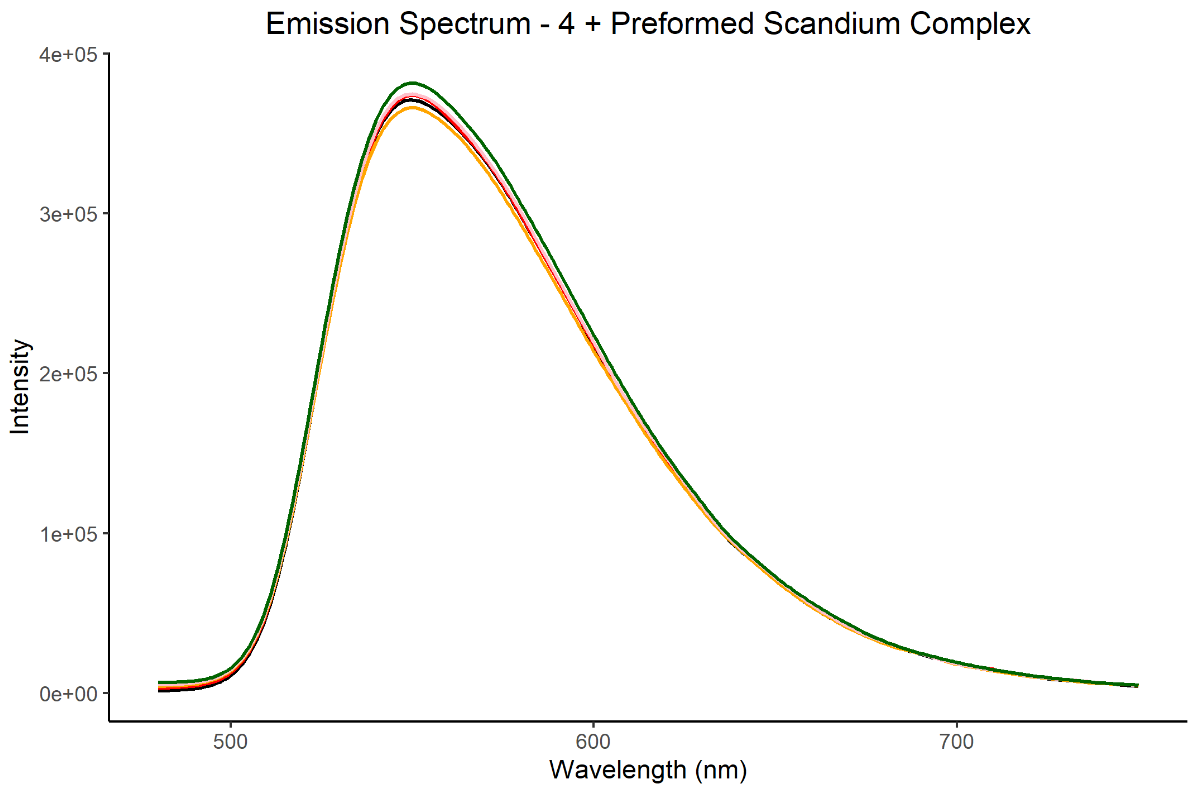

Figure S25 Stern-Volmer Quenching of PC (4) with preformed scandium complex. Concentrations of complex are: 0 mM (black), 0.67 mM (red), 1.14 mM (orange), 1.5 mM (pink), 1.78 mM (blue), 2 mM (green). **4** is 0.5 μM in all cases.

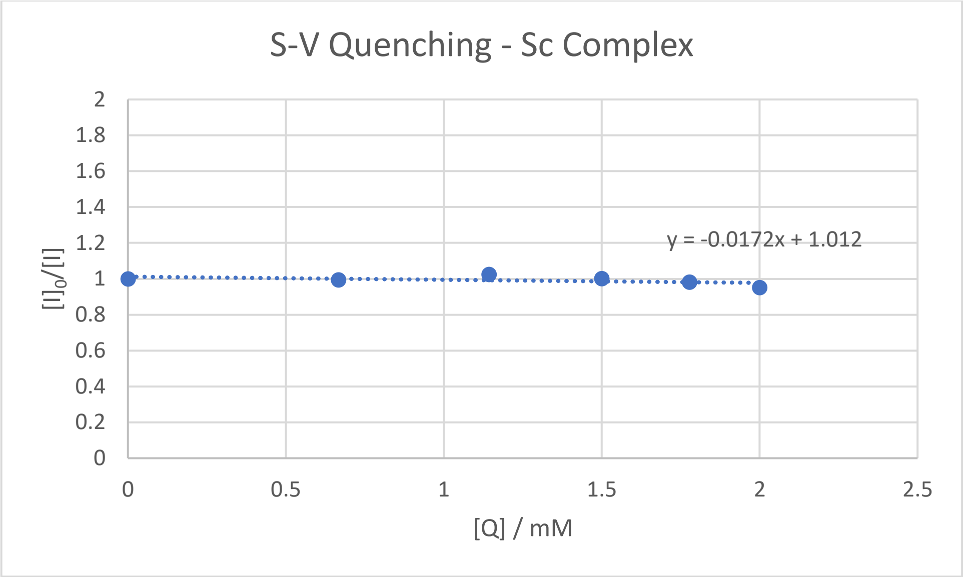

Figure 26 Stern-Volmer plot for **4** + preformed scandium complex

Addition of substrate (1) complex

In the addition of substrate to the photocatalyst it was noted that additional emission was observed from the substrate itself. In the region where the substrate is not emissive (λ > 650 nm) no change in emission was observed even at thigh concentrations

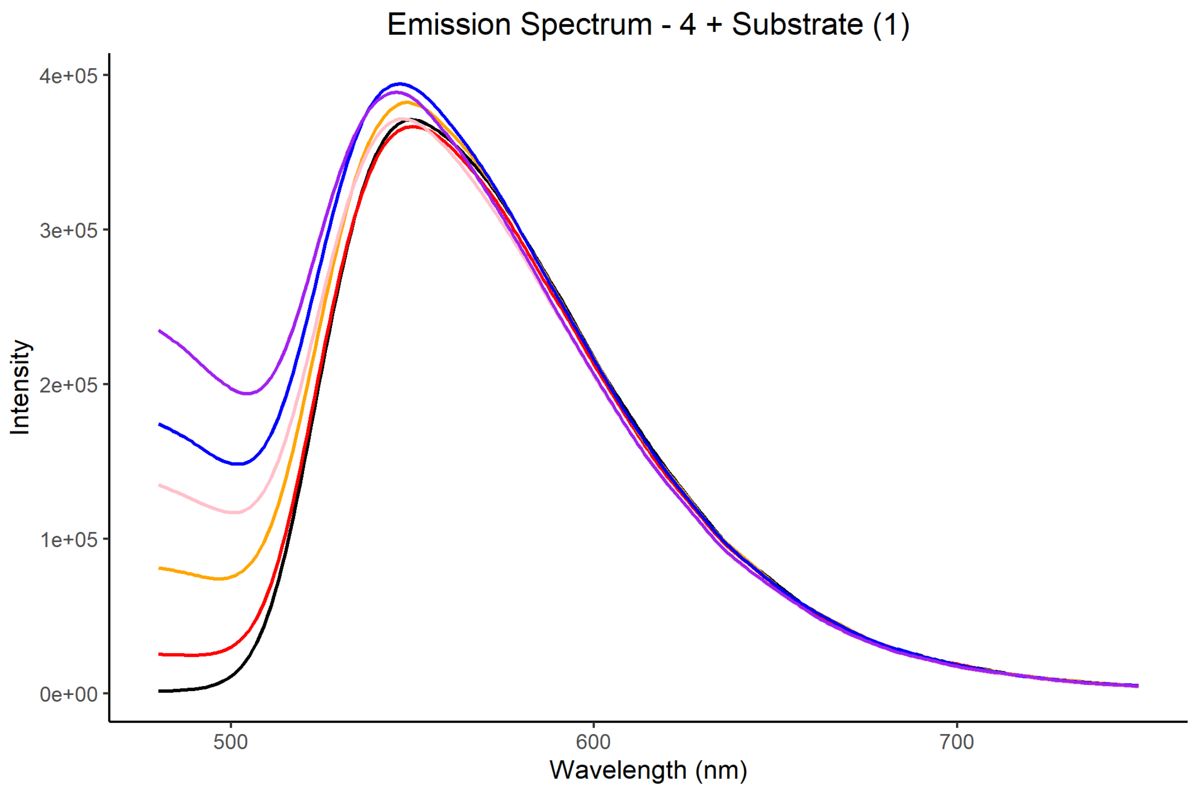

Figure S27 Stern-Volmer Quenching of PC (**4**) with substrate (**1**). Concentrations of **1** are: 0 mM (black), 1.9 mM (red), 6.7 mM (orange), 11.4 mM (pink), 15 mM (blue), 20 mM (purple). **4** is 0.5 µM in all cases.

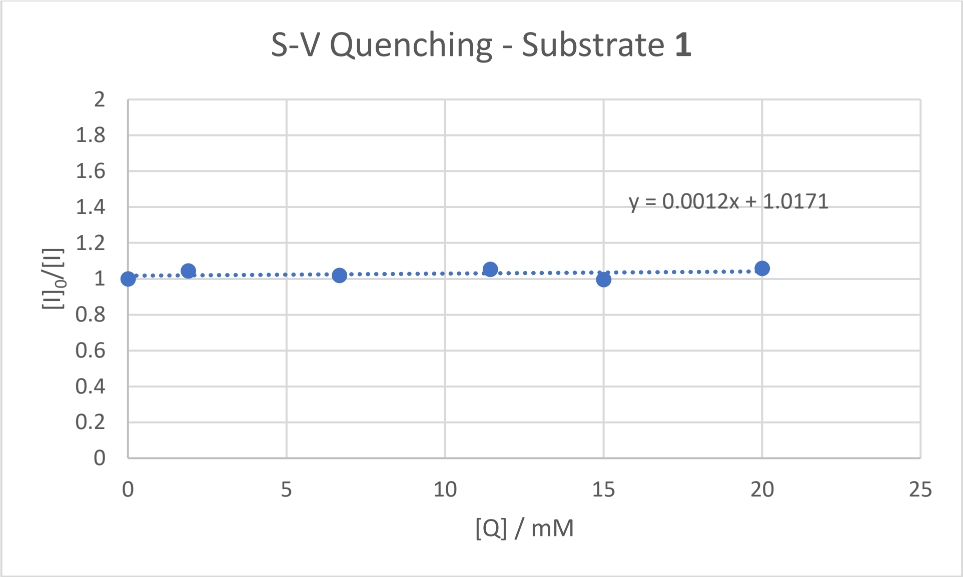

Figure 28 Stern-Volmer plot for **4** + substrate (**1**)

Addition of scandium + 3 complex + substrate (1)

Due to solubility issues, the concentration of scandium complex was limited (maximum 2 mM). The concentration of substrate (**1**) was tenfold. Emission arising from the substrate itself was again observed, but no quenching outside of the substrate's emission range (λ>650 nm) was observed.

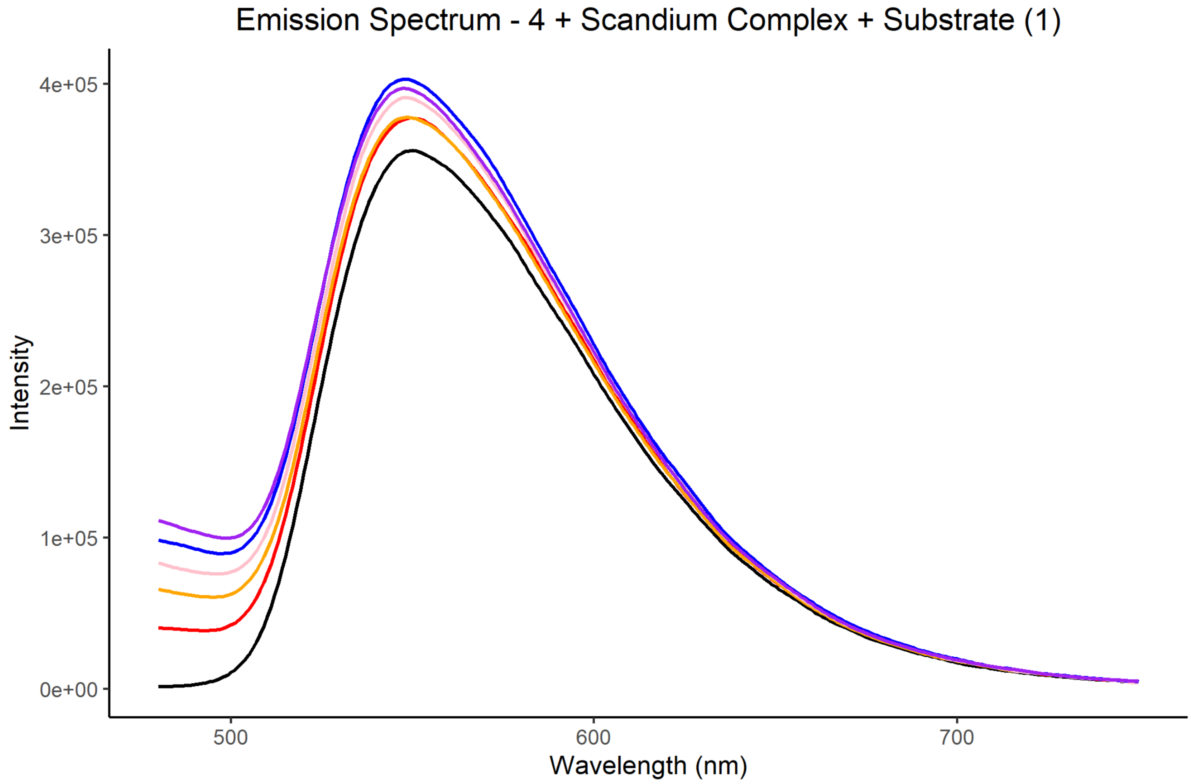

Figure 29 Stern-Volmer Quenching of PC (**4**) with scandium + **3** complex and substrate (**1**). Concentrations of 1 are: 0 (black), 0.17 (red), 0.29 (orange), 0.38 (pink), 0.44 (blue), 0.5 (purple). **4** is 0.5 µM in all cases.

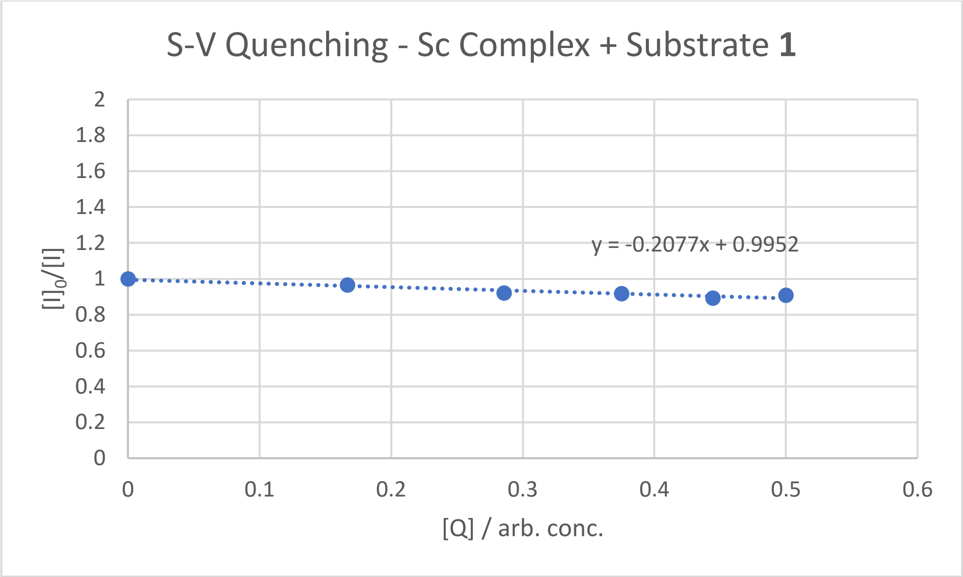

Figure 30 Stern-Volmer plot for **4** + preformed scandium complex + substrate (**1**)

5.6. UV-Vis Absorbance Spectra

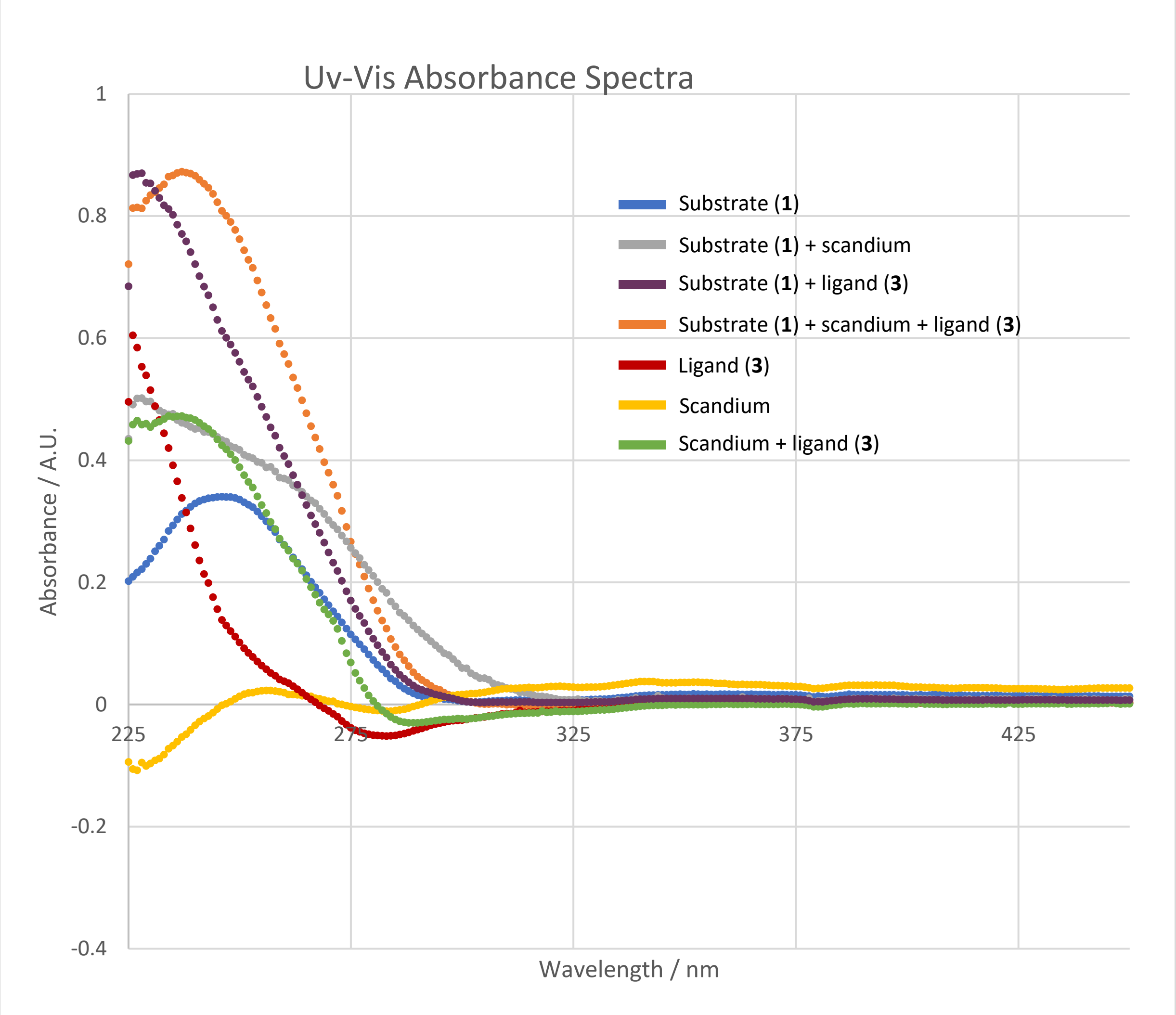

Figure 31 UV-Vis absorbance spectra of reaction components. substrate (**1**), blue, substrate (**1**) + scandium, grey, substrate (**1**) + ligand (**3**), purple, substrate (**1**) + scandium + ligand (**3**), orange, ligand (**3**), red, scandium, yellow, scandium + ligand (**3**), green.

6. X-ray Crystallography

Single crystal X-ray diffraction was carried out by Owen A. Smith using a (Rigaku) Oxford Diffraction/Agilent Supernovae A diffractometer (Cu-K_{α} radiation, λ = 1.54180 Å) equipped with a graphite monochromator within the Department of Chemistry, University of Oxford. Samples were mounted in perfluoropoly-ethyl ether oil and cooled to 150 K during the data collection by a N_2 Crysostream open-flow cooling device.[14] The raw frame data was integrated and reduced using CrysalisPro. CRYSTALS[15,16] was used to obtain an *ab initio* solution using SuperFlip[17] embedded within CRYSTALS and for structure refinement.

(4a*R*,10b*S*)-6-Benzyl-2,3,6,10b-tetrahydro-1*H*-pyrano[2,3-*c*]quinolin-5(4a*H*)-one (5)

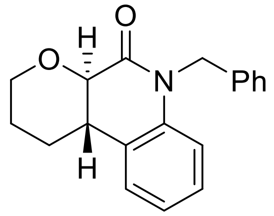

5

Crystals of x-ray quality were grown by vapour diffusion of pentane into a solution of **5** (94:6 e.r.) in ethyl acetate. The flack x parameter was refined to -0.14(17). Bayesian analysis of the Bijvoet pairs gave the Hooft y parameter as -0.11(6). While the measurement errors in the Flack x and Hooft y parameters are too large to allow the full determination of the absolute structure of **5**, it indicates that the above structure is the correct enantiomer.

Identification Code	023OS21 (CCDC 2192819)	
Empirical Formula	C19 H19 N O2	
M_r	293.36	
Temperature	150 K	
Wavelength	λ = 1.54180 Å (Cu K_α)	
Crystal System	Orthorhombic	
Space Group	P 21 21 21	
Unit Cell Dimensions	a = 5.48570(10) Å	α = 90°
	b = 15.4965(3) Å	β = 90°
	c = 17.5203(3) Å	γ = 90°
Volume	1489.39(5) (Å^3)	
Z	4	
Density (Calculated)	1.308 Mg m^{-3}	
Absorption Coefficient	0.672 mm^{-1}	
Crystal Size	0.05 x 0.11 x 0.19 mm^3	
Theta range for data collection	3.808 to 76.496°	
Index Ranges	-6<=h<=6 , -19<=k<=19 , -21<=l<=21	
Reflections Collected	20542	
Independent Reflections	3089 [R(int) = 0.030]	
Completeness to theta = 74.966°	99.9%	
Absorption Correction	multi-scan	
Refinement method	Full-matrix least-squares on F^2	
Goodness-of-fit on F^2	0.9794	
Final R indices [I>2sigma(I)]	R1 = 0.0284, wR2 = 0.0723	
R indices (all data)	R1 = 0.0293, wR2 = 0.0733	
Flack x parameter	-0.14(17)	
Parsons’ q parameter	-0.12(6)	
Hooft y parameter	-0.11(6)	

(4a*R*,10b*S*)-6-Benzyl-9-chloro-2,3,6,10b-tetrahydro-1*H*-pyrano[2,3-c]quinolin-5(4a*H*)-one (17)

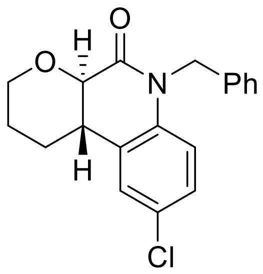

17

Crystals of x-ray quality were grown by vapour diffusion of pentane into a solution of **17** (94.5:5.5 e.r.) in CH_2Cl_2. A void in the crystal structure containing heavily disordered solvent was treated with PLATON SQUEEZE.[18] The flack x parameter was refined to 0.004(9). Bayesian analysis of the Bijvoet pairs gave the Hooft y parameter as 0.000(2) allowing the absolute structure determination of **17**.

Identification Code	031OS21 (CCDC 2192820)	
Empirical Formula	C19 H18 Cl N O2	
M_r	327.81	
Temperature	150 K	
Wavelength	λ = 1.54180 Å (Cu K_α)	
Crystal System	hexagonal	
Space Group	P 65	
Unit Cell Dimensions	a = 19.8511(2) Å	α = 90°
	b = 19.8511(2) Å	β = 90°
	c = 7.64350(10) Å	γ = 120°
Volume	2608.51(6) (Å^3)	
Z	6	
Density (Calculated)	1.252 Mg m^{-3}	
Absorption Coefficient	2.011 mm^{-1}	
Crystal Size	0.05 x 0.06 x 0.45 mm^3	
Theta range for data collection	4.455 to 72.827°	
Index Ranges	-24<=h<=23 , -24<=k<=24 , -9<=l<=9	
Reflections Collected	51007	
Independent Reflections	3468 [R(int) = 0.054]	
Completeness to theta = 72.827°	99.9%	
Absorption Correction	multi-scan	
Refinement method	Full-matrix least-squares on F^2	
Goodness-of-fit on F^2	1.0068	
Final R indices [I>2sigma(I)]	R1 = 0.0450, wR2 = 0.1160	
R indices (all data)	R1 = 0.0457, wR2 = 0.1171	
Flack x parameter	0.004(9)	
Parsons' q parameter	0.004(9)	
Hooft y parameter	0.000(2)	

7. Computational Details

7.1. Methods

The hybrid meta-GGA M06-2X[19] density functional in conjunction with Ahlrichs's split valence triple-ζ def2-TZVP[20] basis set for scandium atoms and Pople's double-ζ 6-31+G(d,p)[21] basis set for the remaining atoms, were used together with Grimme's zero-damped D3[22] correction to optimize all stationary point structures. Previous work from our group has shown that M06-2X in conjunction with triple-ζ basis sets perform well in the study of triplet state transformations; as well as in the prediction of radical properties.[23] For geometry optimization, solvation effects were accounted for using the conductor-like polarizable continuum model (CPCM).[24] To these optimized structures, single-point energy corrections (M06-2X-D3/def2-TZVPP) were applied which included the integral equation formalism variant of the polarizable continuum model (IEF-PCM), with the SMD solvation model to account for solvent effects.[25] *Gaussian 16*[26] version C.01 was employed for all density functional theory (DFT) calculations, using an "ultrafine" pruned (99,590) grid for numerical integration of the exchange-correlation functional and its derivatives. Octahedral distortion parameters were determined using *OctaDist*.[27] Molecular graphics were generated using *PyMol.*[28] Population analysis was performed using *NBO 6.0* interfaced with *Gaussian 16*.[29] Minimum energy crossing points were located using *EasyMECP,* a Python wrapper to the Fortran program originally developed by Harvey.[30]

Vibrational frequency calculations were performed to verify that stationary points were either minima or first-order saddle points on the potential energy surface (PES), and to calculate thermal corrections to Gibbs free energies (G). Intrinsic reaction coordinate (IRC) calculations[31] were performed to ensure that the transition structures connected to their corresponding starting and final geometries. The computed thermochemistry data were corrected following Grimme's quasi-harmonic (QHA) model for entropy[32] with a frequency cut-off value of 100.0 cm^{-1} using the *GoodVibes* program at 298.15 K (25˚C), unless otherwise stated. Also, *GoodVibes* applied (i) 1 M standard concentration corrections to all individual calculations to account for reactions in solution (i.e. change in standard concentration from 1 atm to 1 M)[33] and (ii) multi-conformational corrections (G_{conf}) to all final Boltzmann weighted G to include the entropic stabilization created by multiple accessible low-lying conformers.[34] G values of all the energy profiles correspond to the Boltzmann weighted G of all the conformers found in each step. Boltzmann weighted G (G_{av}) were calculated as:

$$G_{av} = \sum_i G_i \times p_i \tag{1}$$

where G_i is the relative Gibbs free energy of the corresponding conformers of a certain reaction step and pi is the probability of each conformer calculated as:

$$p_i = \frac{e^{\frac{-G_i}{RT}}}{\sum_i \left(e^{\frac{-G_i}{RT}} \right)} \tag{2}$$

7.2. Model $ScCl_3$ Energy Transfer Potential Energy Surface

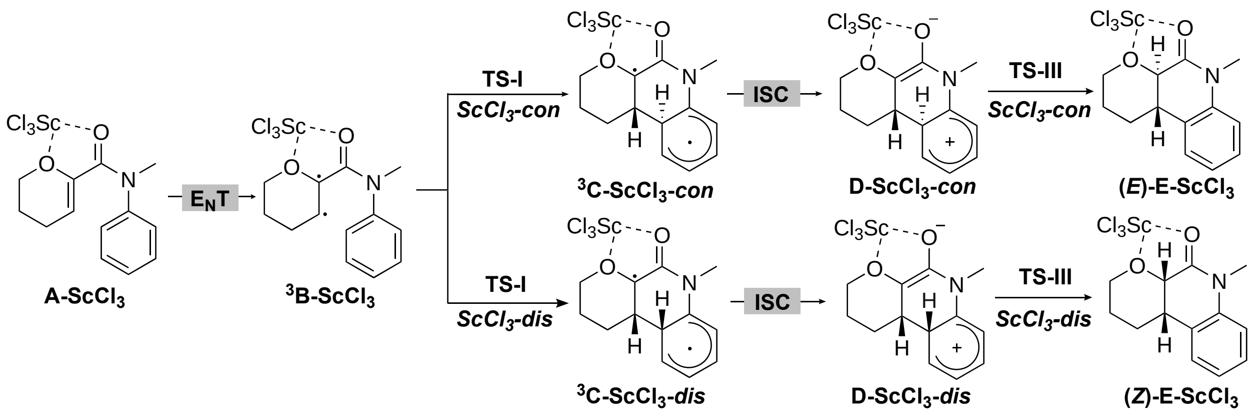

Figure S32: Proposed $ScCl_3$ mediated energy transfer cyclization mechanism.

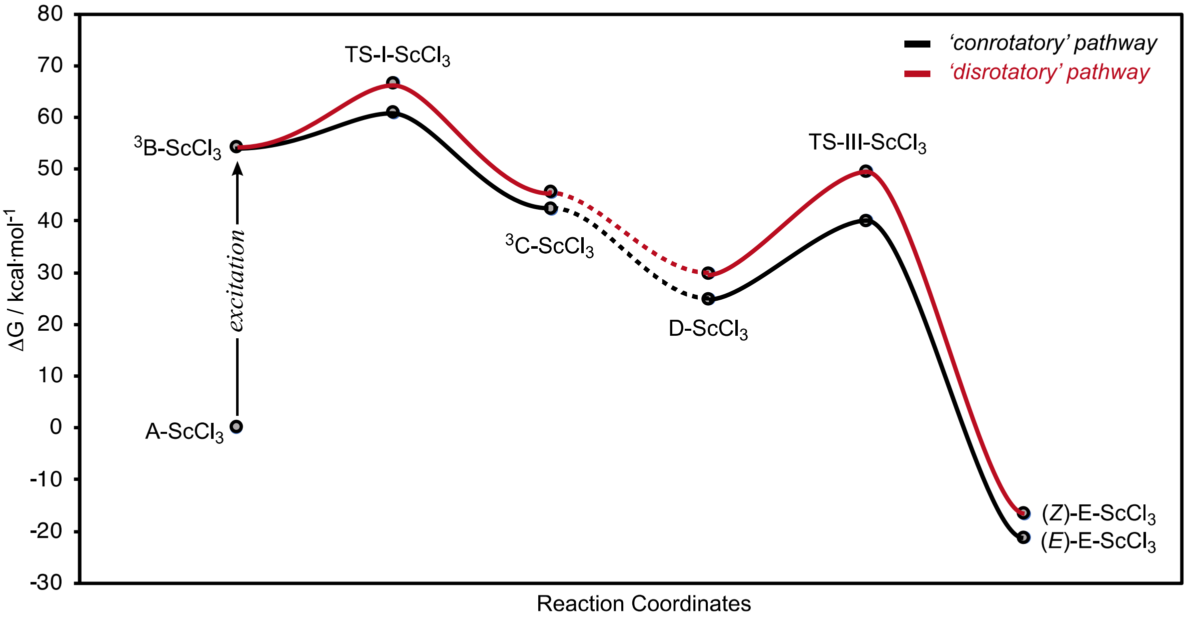

Figure S33: Potential energy surface for the $ScCl_3$ mediated energy transfer cyclization.

Entry	*ΔE*	*ΔZPE*	*ΔH*	*TΔS*	*Tqh-ΔS*	*ΔG(T)*	*qh-ΔG(T)*
A-ScCl₃	0.0	0.0	0.0	0.0	0.0	0.0	0.0
^{3}B-ScCl$_3$	56.2	-2.2	55.3	1.1	1.1	54.3	54.2
TS-I-ScCl$_3$-con	61.7	-1.7	60.2	-1.3	-0.6	61.5	60.8
^{3}C-ScCl$_3$-con	41.3	-0.2	41.5	-1.5	-0.7	43.0	42.2
D-ScCl$_3$-con	22.3	1.1	22.6	-3.2	-2.2	25.8	24.8
TS-III-ScCl$_3$-con	39.4	-1.5	37.1	-4.1	-2.8	41.2	39.9
(E)-E-ScCl$_3$	-25.8	2.4	-24.1	-4.0	-2.8	-20.1	-21.3
TS-I-ScCl$_3$-dis	67.6	-1.9	66.1	-1.4	-0.5	67.5	66.6
^{3}C-ScCl$_3$-dis	44.6	-0.2	44.6	-1.9	-0.9	46.5	45.5
D-ScCl$_3$-dis	27.5	1.2	27.9	-2.9	-1.8	30.8	29.8
TS-III-ScCl$_3$-dis	49.4	-1.6	47.0	-3.9	-2.5	50.8	49.5
(Z)-E-ScCl$_3$	-20.9	2.6	-19.3	-3.9	-2.6	-15.4	-16.7

Table S7: Boltzmann averaged thermochemical data measured relative to A-$ScCl_3$

Cyclization from ^{3}B-$ScCl_3$ to ^{3}C-$ScCl_3$ can give either the *E* or *Z* diastereoisomer of product E-$ScCl_3$ depending on the direction of cyclization via transition state TS-I-$ScCl_3$. The conrotatory cyclization transition state energy was found to be 6.8 kcal·mol^{-1} lower than the corresponding disrotatory transition state. This leads to the formation of the experimentally observed (*E*)-diastereoisomer. The preferential cyclization via the conrotatory transition state TS-I-$ScCl_3$-con was associated to the

minimization of eclipsing integrations between the substituent around the forming C-C bond (**Figure S34**).

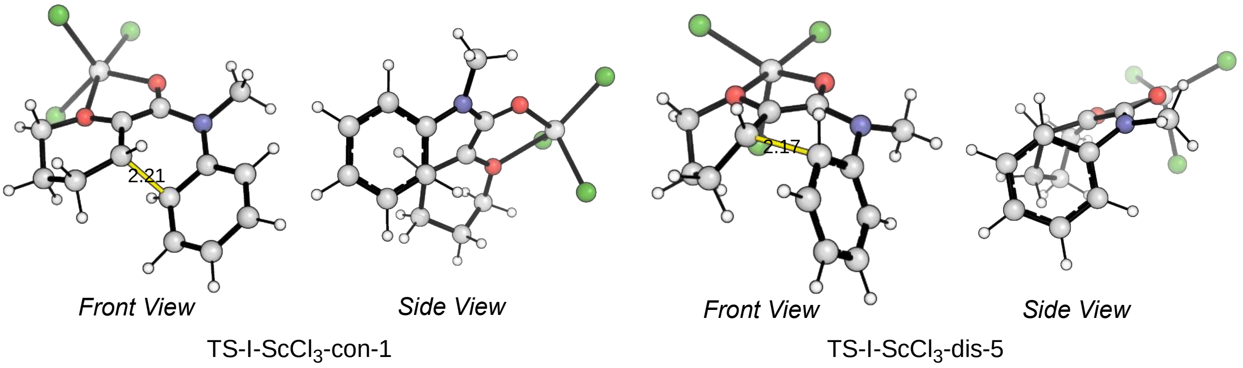

Figure S34: Lowest energy conformers of transition states TS-I-$ScCl_3$-con and TS-I-$ScCl_3$-dis

Upon cyclization from ^{3}B-$ScCl_3$ to ^{3}C-$ScCl_3$, a change in spin multiplicity is necessary to access the products (E-$ScCl_3$) in their ground state. Two MECPs for both the conrotatory and disrotatory pathways were obtained starting from the geometry of the two most stable triplet state conformers. The structures of the MECPs were obtained using EasyMECP at the M06-2X-D3/6-31+G(d,p);def2-TZVP[Sc](CPCM=CH_2Cl_2) level of theory (**Figure S35**). In all four cases, the MECPs were found to be less then 0.12 kcal·mol^{-1} higher in energy than their corresponding triplet state local minimum.

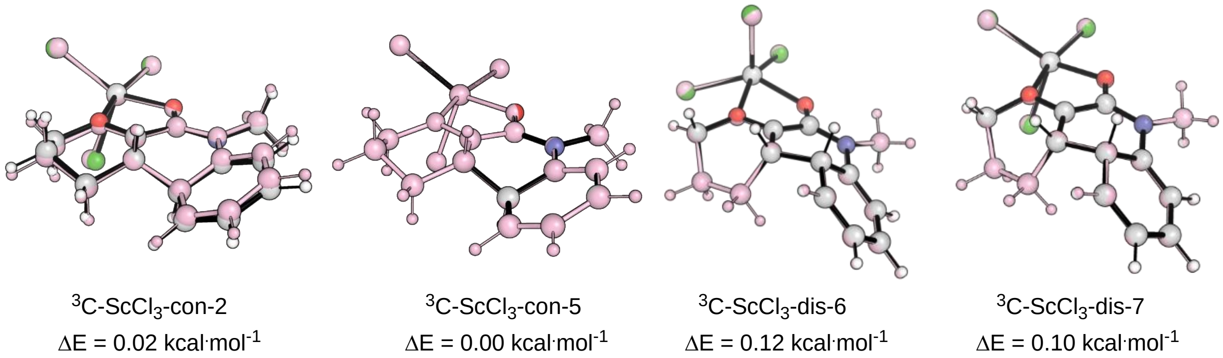

Figure S35: Structures of MECPs (grey) overlayed with their corresponding triplet state local minima (light pink). The difference in energy is reported at the M06-2X-D3/6-31+G(d,p);Def2-TZVP[Sc](CPCM=CH_2Cl_2) level of theory

7.3. Model $ScCl_3$ Redox Neutral Photocyclization Potential Energy Surface

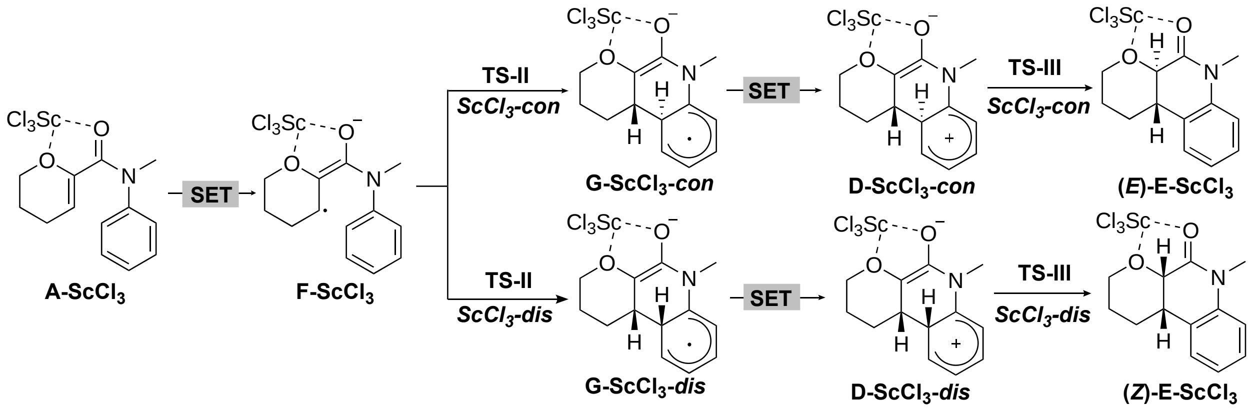

Figure S36: Potential energy surface studies for the $ScCl_3$ mediated redox-neutral cyclization

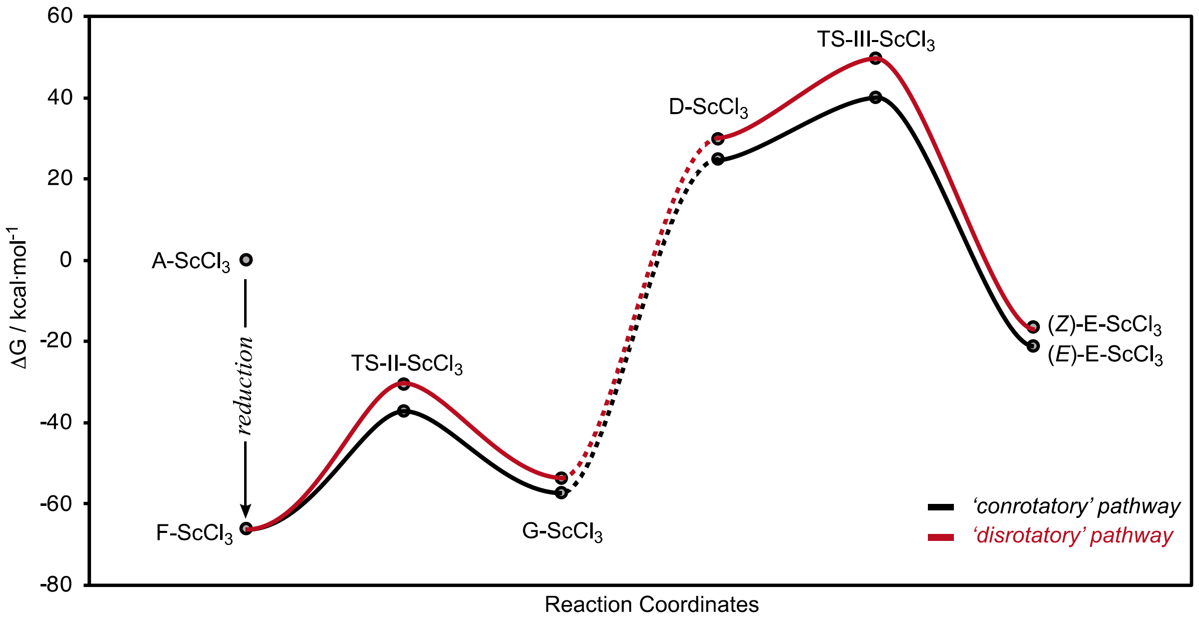

Figure S37: Potential energy surface for the $ScCl_3$ mediated energy transfer cyclization

Entry	*ΔE*	*ΔZPE*	*ΔH*	*TΔS*	*Tqh-ΔS*	*ΔG(T)*	*qh-ΔG(T)*
A-ScCl₃	0.0	0.0	0.0	0.0	0.0	0.0	0.0
F-ScCl₃	-67.0	-2.2	-65.3	1.5	1.0	-66.8	-66.3
TS-II-ScCl₃-con	-40.8	-2.3	-38.5	-2.1	-1.2	-36.4	-37.3
G-ScCl₃-con	-62.4	-0.7	-58.9	-2.6	-1.6	-56.4	-57.3
D-ScCl₃-con	22.3	1.1	22.6	-3.2	-2.2	25.8	24.8
TS-III-ScCl₃-con	39.4	-1.5	37.1	-4.1	-2.8	41.2	39.9
(E)-E-ScCl₃	-25.8	2.4	-24.1	-4.0	-2.8	-20.1	-21.3
TS-II-ScCl₃-dis	-33.9	-2.4	-32.0	-2.2	-1.4	-29.8	-30.6
G-ScCl₃-dis	-59.3	-0.7	-55.7	-2.7	-1.7	-52.9	-53.9
D-ScCl₃-dis	27.5	1.2	27.9	-2.9	-1.8	30.8	29.8
TS-III-ScCl₃-dis	49.4	-1.6	47.0	-3.9	-2.5	50.8	49.5
(Z)-E-ScCl₃	-20.9	2.6	-19.3	-3.9	-2.6	-15.4	-16.7

Table S8: Boltzmann averaged thermochemical data measured relative to A-$ScCl_3$

7.4. Potential Energy Surface Including Thermochemistry of the Photocatalyst

Thermochemical properties of Ir((5-CF_3)(4'-^{t}Bu)ppy)$_3$ were obtained from experimental measurements. The triplet state energy of Ir((5-CF_3)(4'-^{t}Bu)ppy)$_3$ was measured by emission spectroscopy to be 55.7 kcal·mol^{-1}. Similarly, the ground state reduction potential of the [Ir]$^+$/[Ir] couple was measured experimentally to be 0.78 V vs SCE in CH_2Cl_2. Using equation (3),[35] the absolute oxidation potential of [Ir]$^+$/[Ir] couple can be obtained, where $E^{abs}_{[Ir/Ir^+]}$ is the absolute redox potential of the [Ir]$^+$/[Ir] couple, $E^{SCE}_{[Ir/Ir^+]}$ is the redox potential of the [Ir]$^+$/[Ir] couple measured relative to SCE, and E_L is the interliquid potential. Previous studies have shown that the E_L is proportional to the dipole moment of the solvent.[36] As CH_2Cl_2 possesses a small dipole moment () and no experimental E_L values are available, contributions for the E_L term were assumed to be minimal and therefore it was removed from the calculation of the absolute potential.

$$E^{abs}_{[Ir/Ir^+]} = E^{SCE}_{[Ir/Ir^+]} + E^{abs}_{SCE} + E_L \qquad (3)$$

$$E^{abs}_{[Ir/Ir^+]} = 0.78\,V + 4.52\,V = 5.30\,V = 122.3\,kcal \cdot mol^{-1}$$

Therefore, a potential energy surface measure relative to the A-$ScCl_3$ and the Ir((5-CF_3)(4'-t-Bu)ppy)$_3$ species can be constructed by adding 55.7 kcal·mol^{-1} whenever the triplet iridium species is present, and 122.3 kcal·mol^{-1} whenever the oxidized photocatalyst is formed:

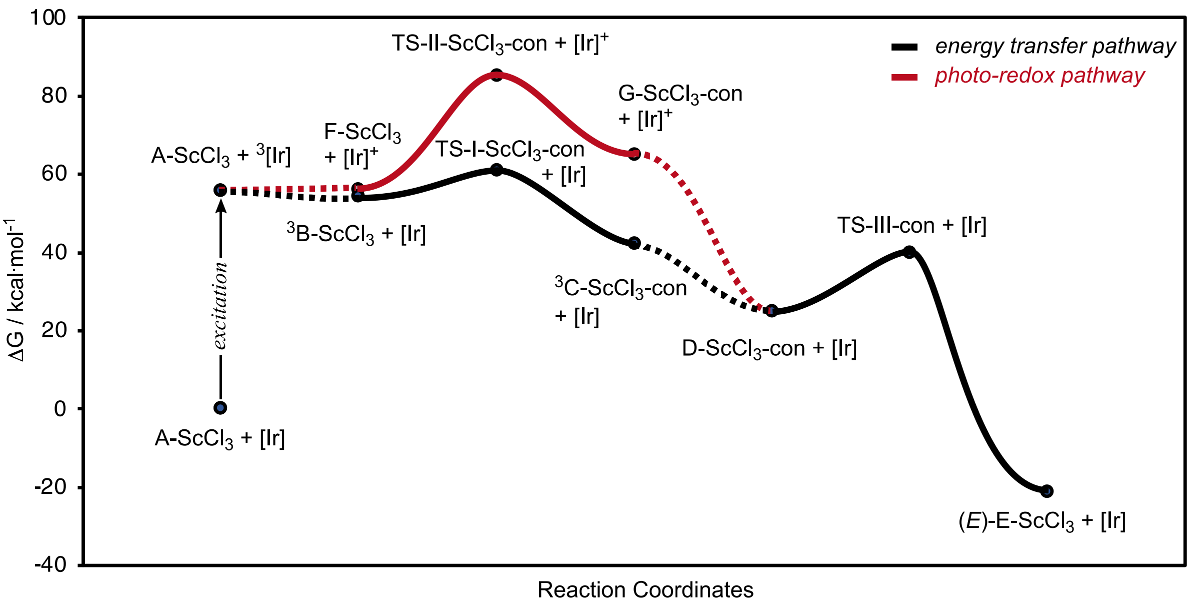

Figure S38: Reaction potential Energy surface when thermochemistry of the iridium photocatalyst is included; Unfavorable disrotatory pathways have been omitted for clarity

7.5. 3D Structures of $ScCl_3$ Model Stationary Points

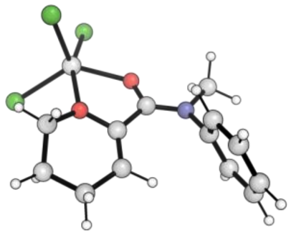

A-$ScCl_3$-6

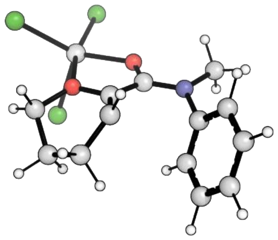

^{3}B-$ScCl_3$-3

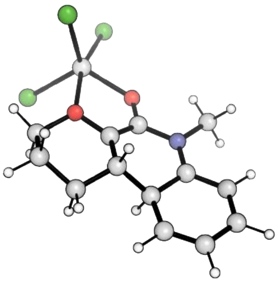

^{3}C-$ScCl_3$-con-5

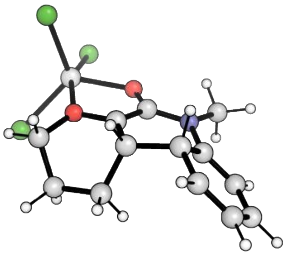

^{3}C-$ScCl_3$-dis-7

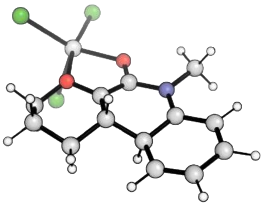

D-$ScCl_3$-con-6

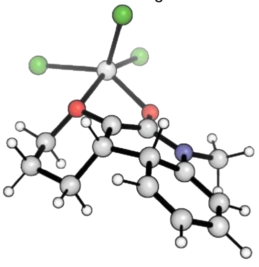

D-$ScCl_3$-dis-6

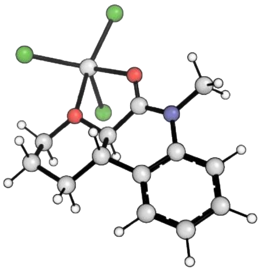

(*E*)-E-$ScCl_3$-4

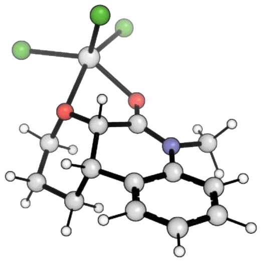

(*Z*)-E-$ScCl_3$-5

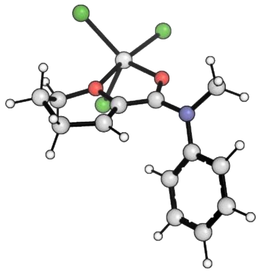

F-$ScCl_3$-5

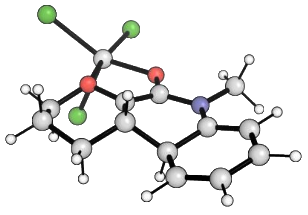

G-$ScCl_3$-con-5

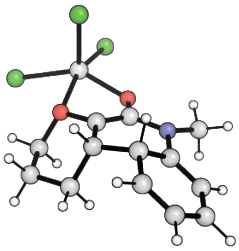

G-$ScCl_3$-dis-4

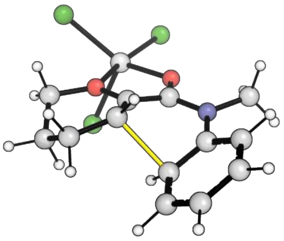

TS-I-$ScCl_3$-con-5

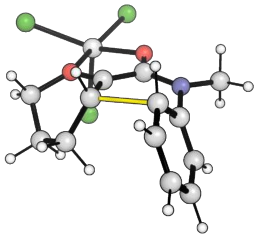

TS-I-$ScCl_3$-dis-1

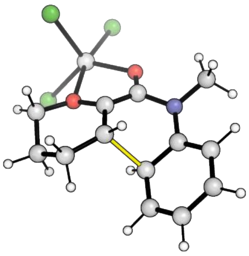

TS-II-$ScCl_3$-con-2

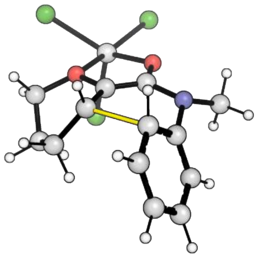

TS-II-$ScCl_3$-dis-1

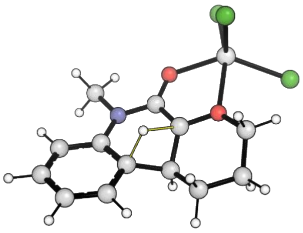

TS-III-$ScCl_3$-con-5

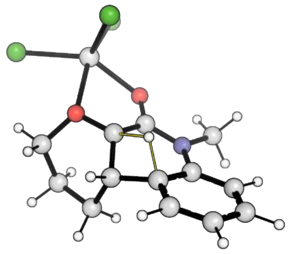

TS-III-$ScCl_3$-dis-6

Figure S39: Structures of stationary points for the $ScCl_3$ model

7.6. Computational Studies of the Chiral Ligand-Containing System

Geometries of the chiral Sc-Feng system were obtained starting by refining a previously reported crystal structure of the ligand **3** with $Sc(OTf)_3$ (CCDC ID: YOPREB)[3] and superimposing the substrate geometries obtained from the $ScCl_3$ model study. For the triplet state pathway, the enantioselectivity was studied at four additional levels of theory. Little variation in enantioselectivity was observed across the four functionals studied (**Table S9**).

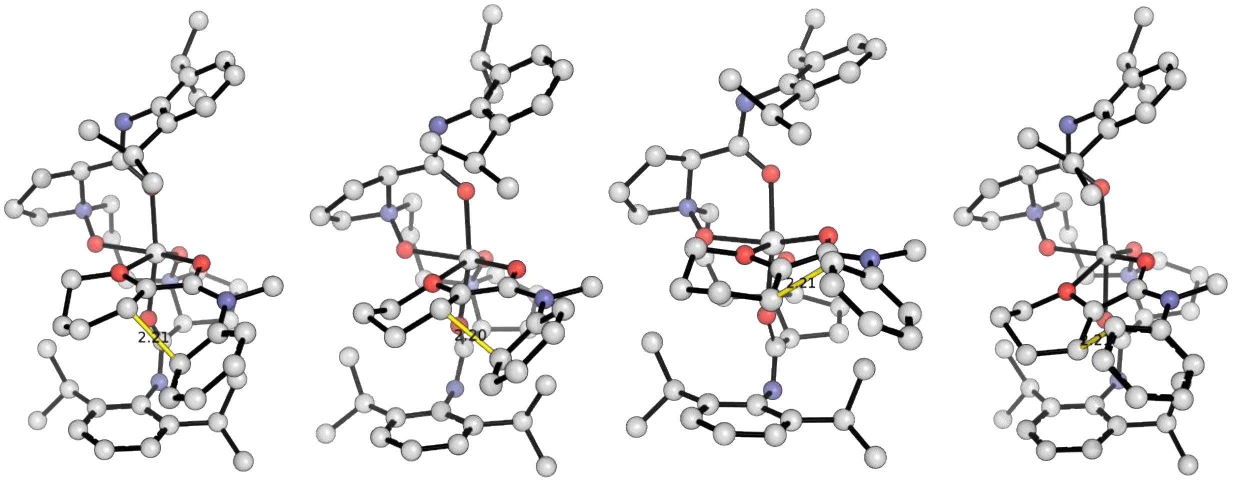

Figure SC9: Transition states for the triplet state cyclization; hydrogen atoms have been omitted for clarity

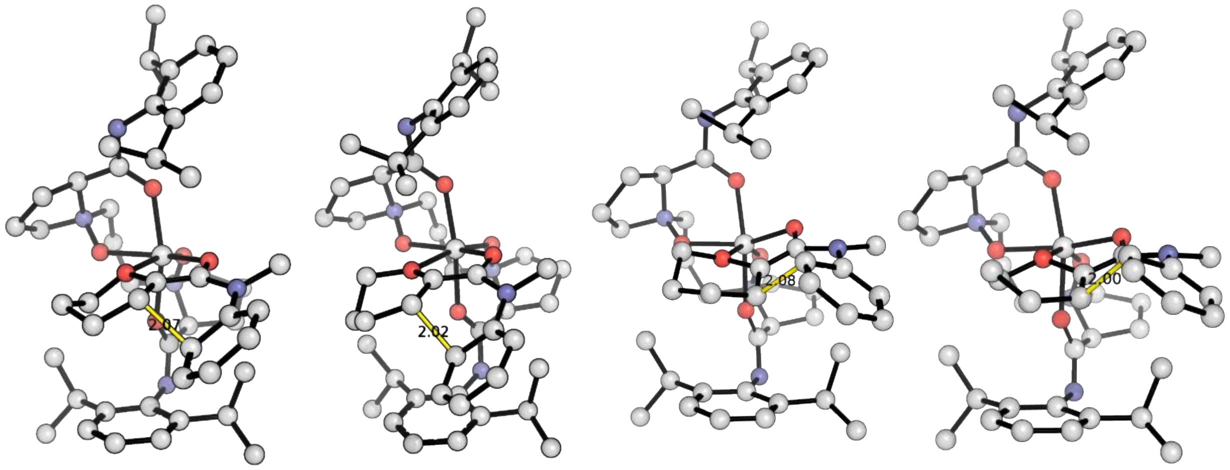

Figure S40: Transition states for the reduced state cyclization; hydrogen atoms have been omitted for clarity

Level of theory	*$\Delta\Delta G^{\ddagger}$ / kcal·mol^{-1}*
M06-2X-D3/Def2-TZVPP(SMD=CH_2Cl_2)	1.3
M06-L-D3/Def2-TZVPP(SMD=CH_2Cl_2)	1.3
B3LYP-D3(BJ)[37] /Def2-TZVPP(SMD=CH_2Cl_2)	1.2
ωB97X-D[38]/Def2-TZVPP(SMD=CH_2Cl_2)	1.1

Table S9: Comparison of predicted enantioselectivity in the triplet state against level of theory, using geometries optimized at the M06-2X-D3/6-31+G(d,p);Def2-TZVP[Sc](CPCM=CH_2Cl_2) level of theory

7.7. Population Analysis

Electron delocalization in substrates **^{3}B-ScCl$_3$** and **F-ScCl$_3$** was studied by population analysis using Natural Population Analysis with *NBO 6.0*. Spin density plots were generated using *PyMol*. In both species the spin density resides predominantly on the organic substrate. In triplet **^{3}B-ScCl$_3$**, one of the unpaired electrons is effectively localized on C3 (spin density = 0.92), while in radical anion **F** spin density is almost equally delocalized between C1 (0.49) and C3 (0.43).

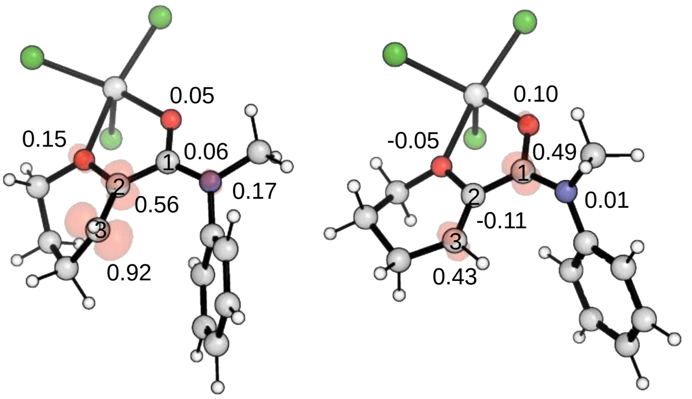

^{3}B-ScCl$_3$-3 **F-ScCl$_3$-5**

Figure S41: NBO spin density plots generated using an isovalue of 0.03

7.8. Thermochemical Data

Legend:
E_T = adiabatic triplet energy
E_{SPC} = energy obtained in the single-point energy corrections
E = energy obtained in the geometry optimizations
ZPE = zero-point energy
H_{SPC} = enthalpy corrected with E_{SPC}
T·S = temperature times entropy with no correction
T·qh-S = temperature times entropy with quasi-harmonic S correction
G(T) = Gibbs free energy corrected only with E_{SPC}
qh-G(T) = Gibbs free energy with E_{SPC} and quasi-harmonic S correction
ν_{imag} = imaginary frequencies

Lewis Acid	***E_T / kcal·mol^{-1}***
N/A	57.7
$ScCl_3$	54.2
[Sc-Feng]$^{3+}$	52.9

Table S10: Computed change in triplet energy upon binding of Lewis acids to substrate obtained at the M06-2X-D3/Def2-TZVPP(SMD=CH_2Cl_2)//M06-2X-D3/6-31+G(d,p);Def2-TZVP[Sc](CPCM=CH_2Cl_2) level of theory

**Name**	**E_{SPC}**	**E**	**ZPE**	**H_{SPC}**	**T·S**	**T·gh-S**	**G(T)**	**qh-G(T)**	**ν_{imag}**
**(E)-E-ScCl3-1**	-2851.285332	-2850.918047	0.270300	-2850.994034	0.070631	0.068028	-2851.064666	-2851.062062	N/A
**(E)-E-ScCl3-2**	-2851.295026	-2850.927447	0.270419	-2851.003951	0.069207	0.067135	-2851.073158	-2851.071086	N/A
**(E)-E-ScCl3-3**	-2851.294854	-2850.927246	0.270141	-2851.003783	0.070792	0.067909	-2851.074575	-2851.071692	N/A
**(E)-E-ScCl3-4**	-2851.296217	-2850.927524	0.270228	-2851.005143	0.070232	0.067562	-2851.075375	-2851.072705	N/A
**(E)-E-ScCl3-5**	-2851.287051	-2850.918265	0.269939	-2850.995916	0.071601	0.068474	-2851.067517	-2851.064391	N/A
**(E)-E-ScCl3-6**	-2851.286562	-2850.917817	0.270209	-2850.995336	0.070555	0.067963	-2851.065891	-2851.063299	N/A
**(Z)-E-ScCl3-1**	-2851.285912	-2850.919032	0.270756	-2850.994414	0.069667	0.067349	-2851.064082	-2851.061763	N/A
**(Z)-E-ScCl3-2**	-2851.286406	-2850.919311	0.270544	-2850.995140	0.069635	0.067319	-2851.064774	-2851.062459	N/A
**(Z)-E-ScCl3-3**	-2851.287082	-2850.918674	0.270573	-2850.995616	0.070373	0.067758	-2851.065989	-2851.063374	N/A

**(Z)-E-ScCl3-4**	-2851.287871	-2850.919624	0.270212	-2850.996614	0.071186	0.068245	-2851.067800	-2851.064859	N/A
**(Z)-E-ScCl3-5**	-2851.289176	-2850.919932	0.270599	-2850.997858	0.069601	0.067294	-2851.067458	-2851.065152	N/A
**(Z)-E-ScCl3-6**	-2851.284844	-2850.916469	0.270411	-2850.993603	0.070401	0.067699	-2851.064004	-2851.061302	N/A
**3B-1**	-709.502865	-709.253738	0.248331	-709.238432	0.059997	0.057819	-709.298428	-709.2962510	N/A
**3B-2**	-709.501807	-709.252103	0.248639	-709.237098	0.059942	0.057760	-709.297039	-709.2948580	N/A
**3B-Feng-1**	-3433.527157	-3432.543426	1.164869	-3432.299996	0.156864	0.145488	-3432.456860	-3432.445484	N/A
**3B-Feng-2**	-3433.527039	-3432.544378	1.164482	-3432.300075	0.158046	0.146039	-3432.458121	-3432.446114	N/A
**3B-Feng-3**	-3433.527416	-3432.544112	1.164656	-3432.300319	0.158315	0.146255	-3432.458633	-3432.446573	N/A
**3B-Feng-4**	-3433.529153	-3432.544699	1.164686	-3432.302172	0.157523	0.145873	-3432.459695	-3432.448045	N/A
**3B-ScCl3-1**	-2851.160712	-2850.793824	0.263412	-2850.874375	0.077288	0.073166	-2850.951663	-2850.947541	N/A
**3B-ScCl3-2**	-2851.163927	-2850.796868	0.262726	-2850.877972	0.078995	0.073981	-2850.956967	-2850.951952	N/A
**3B-ScCl3-3**	-2851.164749	-2850.797411	0.263022	-2850.878776	0.077347	0.073255	-2850.956123	-2850.952031	N/A
**3B-ScCl3-4**	-2851.163320	-2850.796574	0.263121	-2850.877281	0.077141	0.073170	-2850.954422	-2850.950450	N/A
**3B-ScCl3-5**	-2851.163203	-2850.795198	0.263371	-2850.876992	0.076887	0.072931	-2850.953879	-2850.949923	N/A
**3B-ScCl3-6**	-2851.163209	-2850.795149	0.263228	-2850.876998	0.077937	0.073420	-2850.954935	-2850.950418	N/A
**3C-ScCl3-con-1**	-2851.185087	-2850.817496	0.265896	-2850.897266	0.074975	0.071037	-2850.972241	-2850.968303	N/A
**3C-ScCl3-con-2**	-2851.188255	-2850.820944	0.266072	-2850.900474	0.073692	0.070432	-2850.974166	-2850.970906	N/A
**3C-ScCl3-con-3**	-2851.186142	-2850.818398	0.266136	-2850.898320	0.073546	0.070329	-2850.971866	-2850.968650	N/A
**3C-ScCl3-con-4**	-2851.184816	-2850.818168	0.266460	-2850.896871	0.072550	0.069845	-2850.969421	-2850.966716	N/A
**3C-ScCl3-con-5**	-2851.188462	-2850.820963	0.266042	-2850.900705	0.073771	0.070400	-2850.974477	-2850.971106	N/A
**3C-ScCl3-con-6**	-2851.187576	-2850.819796	0.266151	-2850.899743	0.073620	0.070357	-2850.973362	-2850.970099	N/A

**3C-ScCl3-con-7**	-2851.186952	-2850.820666	0.266173	-2850.899189	0.072951	0.070103	-2850.972140	-2850.969292	N/A
**3C-ScCl3-con-8**	-2851.185901	-2850.818299	0.266074	-2850.898111	0.073705	0.070394	-2850.971816	-2850.968506	N/A
**3C-ScCl3-con-2-MECP-S0**	N/A	-2850.820901	N/A	N/A	N/A	N/A	N/A	N/A	N/A
**3C-ScCl3-con-2-MECP-T1**	N/A	-2850.820909	N/A	N/A	N/A	N/A	N/A	N/A	N/A
**3C-ScCl3-con-5-MECP-S0**	N/A	-2850.820964	N/A	N/A	N/A	N/A	N/A	N/A	N/A
**3C-ScCl3-con-5-MECP-T1**	N/A	-2850.820962	N/A	N/A	N/A	N/A	N/A	N/A	N/A
**3C-ScCl3-dis-1**	-2851.181376	-2850.815019	0.266211	-2850.893534	0.073224	0.070256	-2850.966759	-2850.963790	N/A
**3C-ScCl3-dis-2**	-2851.178958	-2850.812221	0.265552	-2850.891639	0.073823	0.070548	-2850.965462	-2850.962187	N/A
**3C-ScCl3-dis-3**	-2851.180649	-2850.812941	0.265841	-2850.893206	0.072912	0.070144	-2850.966118	-2850.963350	N/A
**3C-ScCl3-dis-4**	-2851.179731	-2850.811887	0.265584	-2850.892451	0.073547	0.070368	-2850.965998	-2850.962819	N/A
**3C-ScCl3-dis-5**	-2851.181218	-2850.814868	0.266247	-2850.893408	0.072834	0.070070	-2850.966242	-2850.963478	N/A
**3C-ScCl3-dis-6**	-2851.182226	-2850.814224	0.265866	-2850.894529	0.074308	0.070832	-2850.968838	-2850.965361	N/A
**3C-ScCl3-dis-7**	-2851.183697	-2850.815916	0.266044	-2850.896025	0.073075	0.070191	-2850.969100	-2850.966217	N/A
**3C-ScCl3-dis-6-MECP-S0**	N/A	-2850.814035	N/A	N/A	N/A	N/A	N/A	N/A	N/A
**3C-ScCl3-dis-6-MECP-T1**	N/A	-2850.814026	N/A	N/A	N/A	N/A	N/A	N/A	N/A
**3C-ScCl3-dis-7-MECP-S0**	N/A	-2850.815754	N/A	N/A	N/A	N/A	N/A	N/A	N/A

**3C-ScCl3-dis-7-MECP-T1**	N/A	-2850.815752	N/A	N/A	N/A	N/A	N/A	N/A	N/A
**A-1**	-709.598925	-709.346757	0.251646	-709.331550	0.058755	0.056294	-709.390305	-709.3878440	N/A
**A-2**	-709.598749	-709.346610	0.251688	-709.331319	0.058783	0.056334	-709.390101	-709.3876530	N/A
**A-Feng-1**	-3433.618394	-3432.633942	1.168094	-3432.388284	0.156241	0.144311	-3432.544525	-3432.532595	N/A
**A-Feng-2**	-3433.620728	-3432.634496	1.168348	-3432.390434	0.155817	0.144051	-3432.546251	-3432.534486	N/A
**A-ScCl3-1**	-2851.253469	-2850.885421	0.266512	-2850.964496	0.075649	0.071420	-2851.040144	-2851.035916	N/A
**A-ScCl3-2**	-2851.255224	-2850.886356	0.266556	-2850.966213	0.075768	0.071332	-2851.041981	-2851.037545	N/A
**A-ScCl3-3**	-2851.255087	-2850.885861	0.266580	-2850.966081	0.075728	0.071433	-2851.041809	-2851.037514	N/A
**A-ScCl3-4**	-2851.252464	-2850.883745	0.266359	-2850.963576	0.075955	0.071549	-2851.039531	-2851.035125	N/A
**A-ScCl3-5**	-2851.254649	-2850.886749	0.266375	-2850.965758	0.075822	0.071517	-2851.041580	-2851.037275	N/A
**A-ScCl3-6**	-2851.255627	-2850.886526	0.266259	-2850.966753	0.076592	0.071826	-2851.043345	-2851.038578	N/A
**D-ScCl3-con-1**	-2851.213555	-2850.844174	0.268074	-2850.923838	0.072764	0.069397	-2850.996602	-2850.993235	N/A
**D-ScCl3-con-2**	-2851.218598	-2850.849086	0.268091	-2850.929089	0.072119	0.068892	-2851.001208	-2850.997981	N/A
**D-ScCl3-con-3**	-2851.213313	-2850.843834	0.267880	-2850.923649	0.073561	0.069830	-2850.997210	-2850.993479	N/A
**D-ScCl3-con-4**	-2851.212676	-2850.843010	0.267989	-2850.922957	0.073800	0.069838	-2850.996757	-2850.992795	N/A
**D-ScCl3-con-5**	-2851.218525	-2850.850201	0.268280	-2850.928971	0.071329	0.068514	-2851.000299	-2850.997485	N/A
**D-ScCl3-con-6**	-2851.220254	-2850.851384	0.268180	-2850.930740	0.071536	0.068610	-2851.002275	-2850.999350	N/A
**D-ScCl3-con-7**	-2851.214767	-2850.845591	0.268066	-2850.925248	0.071948	0.068902	-2850.997196	-2850.994151	N/A
**D-ScCl3-con-8**	-2851.213698	-2850.845432	0.268201	-2850.924079	0.071772	0.068786	-2850.995852	-2850.992865	N/A
**D-ScCl3-dis-1**	-2851.208691	-2850.840922	0.268075	-2850.919054	0.072552	0.069268	-2850.991606	-2850.988321	N/A

**D-ScCl3-dis-2**	-2851.211007	-2850.843610	0.268321	-2850.921519	0.070749	0.068202	-2850.992268	-2850.989721	N/A
**D-ScCl3-dis-3**	-2851.211070	-2850.841889	0.267984	-2850.921499	0.072562	0.069266	-2850.994061	-2850.990765	N/A
**D-ScCl3-dis-4**	-2851.209743	-2850.840236	0.268104	-2850.920143	0.072160	0.069051	-2850.992303	-2850.989194	N/A
**D-ScCl3-dis-5**	-2851.212370	-2850.843646	0.268437	-2850.922836	0.070475	0.068020	-2850.993311	-2850.990856	N/A
**D-ScCl3-dis-6**	-2851.210686	-2850.841910	0.268321	-2850.921119	0.071104	0.068487	-2850.992223	-2850.989606	N/A
**F-Feng-1**	-3433.739667	-3432.766388	1.164021	-3432.513324	0.158698	0.145874	-3432.672021	-3432.659197	N/A
**F-Feng-2**	-3433.739428	-3432.764945	1.164250	-3432.513035	0.156842	0.145019	-3432.669876	-3432.658054	N/A
**F-ScCl3-1**	-2851.355736	-2850.993326	0.263332	-2851.069840	0.076081	0.072023	-2851.145921	-2851.141863	N/A
**F-ScCl3-2**	-2851.355233	-2850.992813	0.263110	-2851.069457	0.076442	0.072243	-2851.145899	-2851.141701	N/A
**F-ScCl3-3**	-2851.356313	-2850.993137	0.263065	-2851.070510	0.076942	0.072495	-2851.147453	-2851.143005	N/A
**F-ScCl3-4**	-2851.354957	-2850.992596	0.262945	-2851.069228	0.077520	0.072794	-2851.146748	-2851.142022	N/A
**F-ScCl3-5**	-2851.356031	-2850.993197	0.262765	-2851.070289	0.079858	0.073925	-2851.150147	-2851.144213	N/A
**F-ScCl3-6**	-2851.356398	-2850.993240	0.262799	-2851.070720	0.077815	0.072967	-2851.148536	-2851.143687	N/A
**G-ScCl3-con-1**	-2851.341564	-2850.980373	0.265283	-2851.054580	0.073814	0.070232	-2851.128394	-2851.124813	N/A
**G-ScCl3-con-2**	-2851.345128	-2850.984273	0.265221	-2851.058361	0.072918	0.069661	-2851.131280	-2851.128022	N/A
**G-ScCl3-con-3**	-2851.341584	-2850.980380	0.265302	-2851.054568	0.073795	0.070167	-2851.128363	-2851.124736	N/A
**G-ScCl3-con-4**	-2851.339527	-2850.979038	0.265361	-2851.052474	0.073610	0.070089	-2851.126084	-2851.122564	N/A
**G-ScCl3-con-5**	-2851.347150	-2850.985959	0.265207	-2851.060424	0.072632	0.069560	-2851.133056	-2851.129984	N/A
**G-ScCl3-con-6**	-2851.347101	-2850.985979	0.265294	-2851.060350	0.072244	0.069395	-2851.132594	-2851.129745	N/A
**G-ScCl3-dis-1**	-2851.336428	-2850.976561	0.265153	-2851.049654	0.073134	0.069827	-2851.122788	-2851.119481	N/A
**G-ScCl3-dis-2**	-2851.340604	-2850.981427	0.265295	-2851.053967	0.071982	0.069152	-2851.125949	-2851.123119	N/A

**G-ScCl3-dis-3**	-2851.340442	-2850.979480	0.265199	-2851.053807	0.072381	0.069449	-2851.126188	-2851.123256	N/A
**G-ScCl3-dis-4**	-2851.342111	-2850.981041	0.265194	-2851.055492	0.072340	0.069375	-2851.127832	-2851.124867	N/A
**G-ScCl3-dis-5**	-2851.335904	-2850.976392	0.265100	-2851.049178	0.072986	0.069850	-2851.122163	-2851.119028	N/A
**G-ScCl3-dis-6**	-2851.335755	-2850.976183	0.265253	-2851.048940	0.072740	0.069642	-2851.121680	-2851.118583	N/A
**G-ScCl3-dis-7**	-2851.338249	-2850.977256	0.265156	-2851.051525	0.072690	0.069672	-2851.124215	-2851.121197	N/A
**TS-I-Feng-re-1**	-3433.518413	-3432.535508	1.165871	-3432.291660	0.151899	0.142181	-3432.443559	-3432.433841	-402.50
**TS-I-Feng-re-2**	-3433.519185	-3432.536531	1.165039	-3432.292706	0.155798	0.144115	-3432.448504	-3432.436822	-397.28
**TS-I-Feng-si-1**	-3433.519872	-3432.536673	1.165295	-3432.293392	0.154239	0.143408	-3432.447631	-3432.436800	-410.76
**TS-I-Feng-si-2**	-3433.521691	-3432.537483	1.165080	-3432.295312	0.154646	0.143520	-3432.449958	-3432.438832	-383.36
**TS-I-ScCl3-con-1**	-2851.153359	-2850.786077	0.264034	-2850.867661	0.073152	0.070461	-2850.940813	-2850.938121	-382.86
**TS-I-ScCl3-con-2**	-2851.155409	-2850.788256	0.264195	-2850.869683	0.072776	0.070153	-2850.942459	-2850.939836	-378.20
**TS-I-ScCl3-con-3**	-2851.152898	-2850.785564	0.263553	-2850.867402	0.074796	0.071190	-2850.942198	-2850.938592	-386.82
**TS-I-ScCl3-con-4**	-2851.154590	-2850.786870	0.263677	-2850.869056	0.074549	0.071074	-2850.943605	-2850.940130	-399.50
**TS-I-ScCl3-con-5**	-2851.156400	-2850.788430	0.263684	-2850.870900	0.074283	0.070943	-2850.945184	-2850.941844	-397.17
**TS-I-ScCl3-con-6**	-2851.155449	-2850.787489	0.263697	-2850.869898	0.074793	0.071017	-2850.944691	-2850.940916	-379.18
**TS-I-ScCl3-dis-1**	-2851.146727	-2850.778904	0.263301	-2850.861603	0.073955	0.070763	-2850.935559	-2850.932366	-478.54
**TS-I-ScCl3-dis-2**	-2851.143627	-2850.776332	0.263147	-2850.858546	0.074667	0.071145	-2850.933213	-2850.929691	-465.21
**TS-I-ScCl3-dis-3**	-2851.144864	-2850.777631	0.263285	-2850.859633	0.074628	0.071190	-2850.934261	-2850.930823	-473.26
**TS-I-ScCl3-dis-4**	-2851.142668	-2850.774907	0.262889	-2850.857735	0.075399	0.071534	-2850.933134	-2850.929268	-466.36
**TS-I-ScCl3-dis-5**	-2851.144265	-2850.778021	0.263271	-2850.859040	0.074644	0.071155	-2850.933684	-2850.930194	-474.80
**TS-I-ScCl3-dis-6**	-2851.146586	-2850.779122	0.263386	-2850.861416	0.073672	0.070677	-2850.935089	-2850.932093	-475.48

**TS-I-ScCl3-dis-7**	-2851.144164	-2850.777932	0.263554	-2850.858888	0.073401	0.070526	-2850.932289	-2850.929414	-473.47
**TS-II-Feng-re-1**	-3433.696117	-3432.726410	1.163515	-3432.471459	0.153033	0.142506	-3432.624492	-3432.613965	-634.29
**TS-II-Feng-re-2**	-3433.695632	-3432.725118	1.163621	-3432.470895	0.153049	0.142408	-3432.623944	-3432.613303	-752.06
**TS-II-Feng-si-1**	-3433.697584	-3432.727566	1.163840	-3432.472726	0.153998	0.142666	-3432.626725	-3432.615393	-674.35
**TS-II-Feng-si-2**	-3433.699705	-3432.728458	1.163516	-3432.475055	0.153649	0.142668	-3432.628704	-3432.617724	-744.13
**TS-II-ScCl3-con-1**	-2851.309090	-2850.948871	0.262708	-2851.024840	0.073118	0.069754	-2851.097958	-2851.094594	-637.93
**TS-II-ScCl3-con-2**	-2851.312466	-2850.951703	0.262776	-2851.028208	0.072615	0.069529	-2851.100823	-2851.097738	-749.19
**TS-II-ScCl3-con-3**	-2851.308612	-2850.948684	0.262646	-2851.024344	0.073389	0.069936	-2851.097733	-2851.094281	-648.84
**TS-II-ScCl3-con-4**	-2851.311056	-2850.949761	0.262657	-2851.026832	0.073026	0.069809	-2851.099857	-2851.096641	-649.71
**TS-II-ScCl3-con-5**	-2851.312049	-2850.951570	0.262748	-2851.027790	0.072849	0.069595	-2851.100639	-2851.097386	-749.41
**TS-II-ScCl3-con-6**	-2851.311939	-2850.951641	0.262770	-2851.027673	0.072902	0.069647	-2851.100575	-2851.097320	-746.54
**TS-II-ScCl3-dis-1**	-2851.300759	-2850.939313	0.262482	-2851.016725	0.072934	0.069665	-2851.089658	-2851.086390	-479.73
**TS-II-ScCl3-dis-2**	-2851.301964	-2850.940578	0.262571	-2851.017896	0.072805	0.069629	-2851.090701	-2851.087524	-466.17
**TS-II-ScCl3-dis-3**	-2851.298031	-2850.937883	0.262439	-2851.013891	0.074091	0.070247	-2851.087982	-2851.084138	-477.61
**TS-II-ScCl3-dis-4**	-2851.300813	-2850.940632	0.262359	-2851.016859	0.073326	0.069925	-2851.090185	-2851.086784	-471.55
**TS-III-ScCl3-con-1**	-2851.182055	-2850.813315	0.263797	-2850.897203	0.071095	0.068280	-2850.968298	-2850.965482	-1398.67
**TS-III-ScCl3-con-2**	-2851.191395	-2850.824011	0.264044	-2850.906696	0.069184	0.067146	-2850.975880	-2850.973842	-1356.84
**TS-III-ScCl3-con-3**	-2851.181576	-2850.812421	0.263865	-2850.896709	0.070663	0.067987	-2850.967372	-2850.964696	-1385.03
**TS-III-ScCl3-con-4**	-2851.191482	-2850.822756	0.263931	-2850.906756	0.069906	0.067536	-2850.976662	-2850.974292	-1359.90
**TS-III-ScCl3-con-5**	-2851.192319	-2850.823814	0.263880	-2850.907620	0.070215	0.067560	-2850.977836	-2850.975181	-1362.86
**TS-III-ScCl3-con-6**	-2851.182980	-2850.814504	0.263812	-2850.898209	0.070640	0.067909	-2850.968849	-2850.966118	-1329.19

**TS-III-ScCl3-con-7**	-2851.185664	-2850.816596	0.263969	-2850.900918	0.069707	0.067391	-2850.970625	-2850.968310	-1334.88
**TS-III-ScCl3-dis-1**	-2851.173941	-2850.807078	0.263908	-2850.889143	0.070336	0.067756	-2850.959479	-2850.956899	-1421.71
**TS-III-ScCl3-dis-2**	-2851.173458	-2850.804472	0.263967	-2850.888878	0.069197	0.067101	-2850.958076	-2850.955979	-1453.29
**TS-III-ScCl3-dis-3**	-2851.176045	-2850.807997	0.263975	-2850.891405	0.069475	0.067224	-2850.960880	-2850.958629	-1454.09
**TS-III-ScCl3-dis-4**	-2851.173640	-2850.804027	0.263723	-2850.889057	0.070493	0.067734	-2850.959550	-2850.956791	-1447.49
**TS-III-ScCl3-dis-5**	-2851.173690	-2850.805872	0.263835	-2850.889067	0.070317	0.067564	-2850.959384	-2850.956631	-1477.39
**TS-III-ScCl3-dis-6**	-2851.176576	-2850.807681	0.263833	-2850.891857	0.070271	0.067740	-2850.962128	-2850.959597	-1418.95
**TS-III-ScCl3-dis-7**	-2851.176376	-2850.807805	0.263898	-2850.891624	0.070017	0.067673	-2850.961641	-2850.959297	-1421.16

Table S11: Compiled thermochemical data for structures computed at the M06-2X-D3/Def2-TZVPP(SMD=CH_2Cl_2)//M06-2X-D3/6-31+G(d,p);Def2-TZVP[Sc](CPCM=CH_2Cl_2) level of theory.

7.9. XYZ Coordinates for Computed Structures

35
(E)-E-ScCl3-1 Eopt -
2850.918047
O 0.638798 1.098893 -0.105368
C -0.522226 -0.919357 -0.149503
C -0.644132 0.562695 -0.411904
C 0.662677 2.500899 0.288676
N -1.634788 -1.626347 -0.006327
C -1.750311 1.194224 0.418568
C -0.481055 3.245922 -0.370044
H 1.635436 2.873586 -0.035998
H 0.610273 2.537283 1.381274
C -2.918324 -0.989985 -0.040239
C -1.549170 -3.078795 0.184790
C -1.849153 2.693331 0.073145
H -1.462089 1.081828 1.474576
H -0.393538 4.305634 -0.119052
H -0.367373 3.166507 -1.456202
C -3.013134 0.393567 0.184944
C -4.059139 -1.759871 -0.260790
H -0.522731 -3.330784 0.436874
H -2.214776 -3.365950 0.998738
H -1.835972 -3.593928 -0.734146
H -2.219066 3.239401 0.944901
H -2.571746 2.837942 -0.735192
C -4.279113 0.974482 0.206886
H -0.853868 0.694754 -1.485426
C -5.314112 -1.154420 -0.239984
H -3.981375 -2.823125 -0.452507
C -5.427808 0.210836 0.001521
H -4.370909 2.039613 0.392741
H -6.198183 -1.758187 -0.413380
H -6.403054 0.684651 0.025784
O 0.624697 -1.432148 -0.088349
Sc 2.406304 -0.277966 -0.023926
Cl 2.953640 0.675754 2.090526
Cl 3.265254 0.898841 -1.917095
Cl 3.711821 -2.277103 -0.023013
35
(E)-E-ScCl3-2 Eopt -
2850.927447
O -0.654947 -1.392123 -0.387362
C 0.490892 -0.899385 -0.222595
C 0.607495 0.540584 0.209299
N 1.609159 -1.585723 -0.412924
O -0.643493 1.134643 -0.102603
C 1.762581 1.229188 -0.502418
C 1.534100 -2.993349 -0.819208
C -0.695853 2.519278 0.329661
C 1.783402 2.703616 -0.099746
H 1.563905 1.161981 -1.583642
H 0.546891 -3.177160 -1.235315
H 1.692844 -3.644488 0.042807
H 2.297525 -3.177896 -1.574612
C 0.402185 3.317490 -0.352970
H -1.692409 2.867090 0.056284
H -0.594629 2.524932 1.422102
H 2.045608 2.785751 0.963336
H 2.542246 3.245673 -0.670162
H 0.365089 4.343580 0.023410
H 0.200073 3.352507 -1.429123
C 2.884457 -0.981034 -0.157606
C 2.999613 0.419587 -0.187776
C 3.989147 -1.787281 0.105344
C 4.244967 0.988315 0.063201
H 0.761088 0.562608 1.302860
C 5.228546 -1.195049 0.345132
H 3.894294 -2.866217 0.135525
C 5.358887 0.189828 0.326580
H 4.349871 2.068174 0.050810
H 6.086698 -1.824932 0.552664
H 6.321113 0.651749 0.518924
Sc -2.410970 -0.280021 -0.000134
Cl -3.549661 1.236091 -1.455609
Cl -2.353192 0.182018 2.339519
Cl -3.754145 -2.250094 -0.059453
35
(E)-E-ScCl3-3 Eopt -
2850.927246
O 0.567460 -1.480658 0.061588
C -0.569619 -0.956374 -0.050098
C -0.671222 0.507799 -0.398023
N -1.700766 -1.623333 0.141121
O 0.621014 1.041901 -0.170476
C -1.739649 1.176336 0.456671
C -1.656281 -3.054743 0.458438
C 0.698748 2.461451 -0.455774
C -1.730819 2.679631 0.182714
H -1.467381 1.010247 1.510973
H -0.653336 -3.298572 0.799444
H -1.892610 -3.644977 -0.429282
H -2.379190 -3.259638 1.247935
C -0.311752 3.219471 0.387408
H 1.725669 2.744979 -0.225468
H 0.522502 2.589944 -1.530521
H -2.062911 2.864396 -0.847472
H -2.425854 3.193743 0.851870
H -0.260498 4.277311 0.115070
H -0.027766 3.135064 1.442469
C -2.964991 -0.959256 0.009997
C -3.025736 0.436725 0.166236
C -4.113859 -1.703861 -0.244639
C -4.262654 1.064400 0.051950
H -0.927927 0.603354 -1.466458
C -5.343355 -1.053917 -0.347088
H -4.061392 -2.778688 -0.371441
C -5.420571 0.327324 -0.200250
H -4.325831 2.141758 0.164064
H -6.236426 -1.635673 -0.547638
H -6.375716 0.834246 -0.284071
Sc 2.373625 -0.349265 -0.005414
Cl 3.662197 -2.356971 0.048193
Cl 3.235517 0.750298 -1.938554
Cl 3.106443 0.933765 1.858728
35
(E)-E-ScCl3-4 Eopt -
2850.927524
O -0.636277 -1.391744 -0.451979
C 0.499274 -0.890341 -0.259380
C 0.600905 0.557749 0.147301
N 1.627046 -1.576454 -0.396438
O -0.658067 1.141696 -0.159710
C 1.753967 1.236995 -0.579843
C 1.570047 -3.000611 -0.742769
C -0.698798 2.534583 0.254407
C 1.770215 2.721935 -0.218258
H 1.562934 1.138011 -1.659958
H 0.591104 -3.211157 -1.165555
H 1.719637 -3.611206 0.150234
H 2.347214 -3.213121 -1.476530
C 0.382829 3.320548 -0.466473
H -1.700257 2.880195 0.002945
H -0.567694 2.554844 1.343936
H 2.046512 2.834624 0.838344
H 2.518745 3.251208 -0.813880
H 0.344909 4.356406 -0.117897
H 0.162213 3.325349 -1.539672
C 2.891631 -0.951766 -0.143390
C 2.995731 0.447287 -0.230327
C 3.999059 -1.736645 0.168885
C 4.234621 1.035828 0.007579
H 0.760552 0.597437 1.239431
C 5.231048 -1.124937 0.396808
H 3.911308 -2.813776 0.246036
C 5.351770 0.258607 0.316543
H 4.332038 2.114841 -0.050303
H 6.091047 -1.738186 0.642962
H 6.308585 0.736173 0.497305
Sc -2.408929 -0.306664 -0.023881
Cl -2.132083 -0.130780 2.328913
Cl -3.710758 -2.264062 -0.428534
Cl -3.825967 1.442988 -0.834134
35
(E)-E-ScCl3-5 Eopt -
2850.918265
O 0.661318 1.086383 -0.266108
C -0.542767 -0.919638 -0.213591
C -0.643523 0.559350 -0.511664
C 0.697810 2.450002 0.250077
N -1.665188 -1.602631 -0.026734
C -1.721967 1.220295 0.335161
C -0.409815 3.266761 -0.383680
H 1.688838 2.819462 -0.012264
H 0.608983 2.396849 1.339966
C -2.937124 -0.946834 -0.068274
C -1.600391 -3.048015 0.216744
C -1.801310 2.717402 -0.011315
H -1.424242 1.104692 1.388322
H -0.303730 4.305480 -0.062116
H -0.268164 3.252793 -1.469264
C -3.003367 0.443246 0.122022
C -4.093028 -1.700501 -0.263442
H -0.577909 -3.305416 0.479537
H -2.270332 -3.294828 1.040366
H -1.894280 -3.593477 -0.682318
H -2.214066 3.260969 0.842586
H -2.484913 2.862065 -0.853194
C -4.257092 1.050481 0.128539
H -0.883815 0.674105 -1.580062
C -5.335670 -1.069713 -0.255880
H -4.035588 -2.769999 -0.426001
C -5.421097 0.304047 -0.054002
H -4.328084 2.121912 0.284027
H -6.232307 -1.660206 -0.409442
H -6.386638 0.797800 -0.042484
O 0.593644 -1.450919 -0.159604
Sc 2.398313 -0.324296 -0.084749
Cl 2.540758 0.125128 2.236299
Cl 3.633927 -2.314656 -0.558508
Cl 3.747334 1.366671 -1.101866
35
(E)-E-ScCl3-6 Eopt -
2850.917817
O -0.654708 1.122755 -0.117070
C 0.514986 -0.897393 -0.084864
C 0.611963 0.572759 0.245033
C -0.655439 2.560833 -0.376896
N 1.637733 -1.594876 -0.203415
C 1.763148 1.246064 -0.487009
C 0.464938 3.238476 0.385566
H -1.636372 2.911373 -0.061253
H -0.558134 2.694491 -1.458935
C 2.915716 -0.959759 -0.086343
C 1.565372 -3.040871 -0.441559

C 1.847261 2.722101 -0.049503
H 1.528081 1.197242 -1.560909
H 0.386070 4.315993 0.221734
H 0.310082 3.070030 1.456482
C 3.015510 0.432782 -0.240071
C 4.049414 -1.737872 0.143963
H 0.550627 -3.288120 -0.740774
H 2.263227 -3.302278 -1.237004
H 1.817902 -3.582389 0.472378
H 2.238589 3.321521 -0.875839
H 2.545425 2.820504 0.786601
C 4.280122 1.014890 -0.185074
H 0.749692 0.653765 1.335779
C 5.302498 -1.131254 0.201905
H 3.967629 -2.808918 0.282700
C 5.422162 0.243843 0.028756
H 4.376392 2.087643 -0.317031
H 6.180474 -1.741884 0.382219
H 6.396310 0.719156 0.064515
O -0.622505 -1.413534 -0.215289
Sc -2.413061 -0.282512 -0.036949
Cl -3.695046 -2.228021 -0.560766
Cl -3.721517 1.431509 -1.071996
Cl -2.481910 -0.099524 2.325975
35
(Z)-E-ScCl3-1 Eopt -
2850.919032
O 0.643518 1.082509 0.408064
C -0.570997 -0.863515 0.082648
C -0.620394 0.504651 0.739987
C 0.623481 2.527197 0.319548
N -1.699003 -1.490955 -0.215608
C -1.789023 1.356773 0.247974
C -0.208574 2.944532 -0.892551
H 1.665486 2.832733 0.232298
H 0.224044 2.910588 1.263236
C -2.971335 -0.869881 0.001326
C -1.644889 -2.842880 -0.784492
C -1.399150 1.984541 -1.098712
H -1.956421 2.162355 0.970441
H -0.564877 3.965415 -0.724883
H 0.422933 2.953578 -1.784137
C -3.038061 0.512909 0.214909
C -4.131624 -1.644301 -0.024134
H -0.621054 -3.048947 -1.082802
H -1.962330 -3.572690 -0.037181
H -2.302548 -2.885285 -1.652857
H -2.256999 2.510823 -1.523383
H -1.130730 1.187844 -1.803643
C -4.289266 1.101164 0.391602
C -5.371482 -1.034069 0.152429
H -4.081754 -2.715640 -0.173937
C -5.455798 0.340856 0.355891
H -4.341259 2.173253 0.561585
H -6.269011 -1.642573 0.133244
H -6.419861 0.817645 0.495020
O 0.560442 -1.369211 -0.140368
H -0.673589 0.346860 1.827691
Sc 2.375633 -0.296544 0.068476
Cl 3.649135 -2.253521 -0.422159
Cl 3.081635 1.193057 -1.655409
Cl 3.142388 0.287165 2.253477
35
(Z)-E-ScCl3-2 Eopt -
2850.919311
O 0.665239 -0.998214 -0.799331
C -0.471533 -0.599150 -0.428839
C -0.517788 0.597641 0.510597
N -1.591614 -1.213184 -0.782229
O 0.684267 1.337886 0.282045
C -1.812993 1.398967 0.417676
C -1.520162 -2.392534 -1.652476
C 0.644911 2.250999 -0.855750
C -1.832349 2.327524 -0.803727
H -1.878218 2.018549 1.318373
H -0.537409 -2.416916 -2.114619
H -2.290024 -2.309117 -2.419519
H -1.670705 -3.300969 -1.065600
C -0.560013 3.164452 -0.804108
H 1.584636 2.795700 -0.796847
H 0.634383 1.652580 -1.777823
H -1.893891 1.735139 -1.726994
H -2.728622 2.952087 -0.758212
H -0.520238 3.825494 -1.674447
H -0.511706 3.790652 0.093850
C -2.854166 -0.811073 -0.235325
C -2.974069 0.435934 0.394709
C -3.949682 -1.669797 -0.327424
C -4.209399 0.799716 0.926522
C -5.177091 -1.282004 0.206303
H -3.858965 -2.638760 -0.802022
C -5.311908 -0.047630 0.835466
H -4.302534 1.766337 1.414142
H -6.023848 -1.955415 0.130115
H -6.265428 0.253834 1.255011
Sc 2.422956 -0.255403 0.140813
Cl 3.901145 1.466568 -0.569390
Cl 2.075959 -0.558109 2.470552
Cl 3.635782 -2.217590 -0.433157
H -0.415862 0.186125 1.523583
35
(Z)-E-ScCl3-3 Eopt -
2850.918674
O 0.665622 1.142492 0.182894
C -0.549641 -0.813273 -0.110302
C -0.572116 0.537915 0.581029
C 0.609470 2.591321 0.171721
N -1.686787 -1.438775 -0.380964
C -1.781330 1.389632 0.195678
C -0.294195 3.044212 -0.973152
H 1.638020 2.923627 0.051087
H 0.246332 2.915369 1.151729
C -2.952608 -0.839897 -0.083284
C -1.646253 -2.774996 -0.986412
C -1.488007 2.081651 -1.143899
H -1.918088 2.160452 0.960930
H -0.645918 4.055390 -0.747187
H 0.281861 3.094979 -1.900488
C -3.020733 0.531039 0.194326
C -4.107049 -1.623780 -0.089485
H -0.633355 -2.965796 -1.328793
H -1.930079 -3.526635 -0.247225
H -2.336041 -2.800138 -1.830246
H -2.377478 2.616746 -1.484182
H -1.263127 1.320311 -1.900644
C -4.266791 1.098066 0.456870
C -5.342100 -1.034720 0.171922
H -4.056361 -2.686843 -0.289035
C -5.427737 0.328641 0.441644
H -4.319052 2.161045 0.676698
H -6.234613 -1.650856 0.166541
H -6.387878 0.788921 0.647524
O 0.573077 -1.309971 -0.383535
H -0.543590 0.349694 1.665542
Sc 2.388613 -0.287754 0.010583
Cl 3.646248 -2.164229 -0.769191
Cl 3.754625 1.543919 -0.693297
Cl 2.437418 -0.461868 2.374103
35
(Z)-E-ScCl3-4 Eopt -
2850.919624
O 0.665600 -1.060531 -0.648862
C -0.593105 0.875373 -0.358023
C -0.639575 -0.536831 -0.916548
C 0.690788 -2.492936 -0.415655
N -1.721966 1.497115 -0.047501
C -1.745184 -1.376233 -0.277569
C -0.063334 -2.834656 0.870699
H 1.746808 -2.756341 -0.367660
H 0.256047 -2.972020 -1.298033
C -2.984390 0.827641 -0.133097
C -1.675279 2.885371 0.426584
C -1.245858 -1.872059 1.087118
H -1.931986 -2.246294 -0.916003
H -0.424051 -3.864189 0.781727
H 0.614795 -2.789580 1.724932
C -3.017952 -0.569070 -0.230439
C -4.165654 1.568506 -0.086576
H -0.641095 3.141938 0.636895
H -2.069512 3.553056 -0.341800
H -2.271975 2.966996 1.335373
H -2.058588 -2.364896 1.625485
H -0.929733 -1.013014 1.693272
C -4.257397 -1.205502 -0.268957
C -5.393141 0.910645 -0.123385
H -4.141616 2.649415 -0.024571
C -5.443985 -0.478251 -0.209344
H -4.284685 -2.289112 -0.346273
H -6.307559 1.492616 -0.086073
H -6.398551 -0.992149 -0.237871
O 0.532216 1.420755 -0.225122
H -0.780758 -0.459118 -2.004100
Sc 2.351227 0.339304 -0.144259
Cl 3.640428 2.350524 -0.095316
Cl 2.308298 -0.407309 2.103290
Cl 3.811411 -1.164173 -1.302699
35
(Z)-E-ScCl3-5 Eopt -
2850.919932
O 0.628695 -1.063509 -0.782555
C -0.494539 -0.635882 -0.407001
C -0.513423 0.614712 0.460196
N -1.632091 -1.254085 -0.694243
O 0.709017 1.307669 0.196287
C -1.781338 1.446078 0.299935
C -1.599155 -2.490682 -1.482977
C 0.708816 2.124290 -1.014005
C -1.767596 2.270111 -0.994295
H -1.826635 2.139276 1.146559
H -0.626852 -2.564273 -1.961933
H -2.384091 -2.446487 -2.237913
H -1.752018 -3.354127 -0.832229
C -0.471968 3.070424 -1.053868
H 1.663697 2.646557 -0.986204
H 0.686065 1.453142 -1.884457
H -1.840484 1.604905 -1.865758
H -2.645484 2.921567 -1.008857
H -0.407077 3.651432 -1.978065
H -0.413666 3.770658 -0.213272
C -2.877575 -0.784790 -0.161629
C -2.966464 0.514422 0.359376
C -3.989626 -1.627270 -0.163007
C -4.187389 0.947870 0.871776
C -5.201987 -1.170425 0.350468
H -3.923394 -2.636101 -0.550941
C -5.306377 0.117395 0.868411
H -4.255318 1.954693 1.274864
H -6.061601 -1.831729 0.346092
H -6.248473 0.473035 1.271077
Sc 2.411011 -0.269464 0.108986
Cl 1.977083 -0.880695 2.348169
Cl 3.727307 -2.011060 -0.833393
H -0.432147 0.265125 1.497654

Cl 3.898604 1.601101 0.111356
35
(Z)-E-ScCl3-6 Eopt -2850.916469
O 0.593044 1.107700 0.610190
C -0.537163 0.672019 0.272803
C -0.566308 -0.589256 -0.579850
N -1.669956 1.290506 0.579276
O 0.651317 -1.286133 -0.312094
C -1.839230 -1.413126 -0.414465
C -1.620004 2.535615 1.353670
C 0.651095 -2.114484 0.890006
C -1.819188 -2.250096 0.872143
H -1.897196 -2.099606 -1.265779
H -0.635751 2.616614 1.806210
H -2.384478 2.497834 2.129680
H -1.792076 3.391665 0.697890
C -0.528872 -3.060466 0.915907
H 1.608883 -2.635092 0.858744
H 0.633947 -1.452576 1.765431
H -1.881122 -1.593212 1.750647
H -2.701994 -2.894851 0.886252
H -0.465967 -3.654590 1.831803
H -0.476611 -3.748377 0.064849
C -2.924954 0.819387 0.072225
C -3.022534 -0.477972 -0.451704
C -4.038196 1.660166 0.098064
C -4.252913 -0.910938 -0.941767
C -5.259817 1.203756 -0.393204
H -3.965935 2.667750 0.487964
C -5.372812 -0.082297 -0.913666
H -4.327645 -1.916720 -1.346314
H -6.119875 1.864076 -0.369357
H -6.322199 -0.437939 -1.298795
Sc 2.398815 0.264904 -0.185168
Cl 3.215987 2.426540 -0.733617
H -0.486485 -0.251936 -1.622142
Cl 3.391977 -1.002076 -1.955176
Cl 3.354364 -0.334815 1.888251
31
3B-1 Eopt -709.253738
O -2.704383 0.369567 -0.146854
C -0.737508 1.675839 -0.037204
C -1.446434 0.465655 0.326347
C -3.269923 -0.960846 -0.097912
N 0.631464 1.673073 0.212301
C -0.975124 -0.614641 1.202682
C -2.205292 -2.002782 -0.419933
H -4.075375 -0.946697 -0.832146
H -3.696595 -1.124875 0.898458
C 1.377868 0.471006 0.006912
C 1.346765 2.924907 -0.019558
C -1.132174 -2.014636 0.687119
H -0.892371 -0.424103 2.269614
H -2.675959 -2.983834 -0.524921
H -1.755206 -1.746136 -1.386127
C 2.267911 0.031065 0.989777
C 1.247287 -0.245553 -1.186851
H 0.780719 3.747607 0.416588
H 2.325213 2.859811 0.458432
H 1.482021 3.118560 -1.091195
H -1.436826 -2.670661 1.509488
H -0.183309 -2.410338 0.296097
C 3.007341 -1.131775 0.787014
C 1.982189 -1.414052 -1.381976
H 0.566641 0.115496 -1.953606
C 2.861592 -1.860545 -0.395589
H 3.690726 -1.475507 1.556955
H 1.871000 -1.970996 -2.307117
H 3.434104 -2.769788 -0.547875
O -1.315492 2.684475 -0.469043
H 2.356977 0.595766 1.913457
31
3B-2 Eopt -709.252103
O 1.096171 2.779536 0.329999
C 0.571974 1.700598 0.023968
C 1.357839 0.496644 -0.214137
N -0.792540 1.600807 -0.185527
O 2.662270 0.594953 0.119217
C 0.957997 -0.675142 -1.014744
C -1.571435 2.831534 -0.072418
C 3.278072 -0.632550 0.573288
C 1.463512 -1.986586 -0.509757
H 0.859774 -0.528357 -2.090277
H -1.054794 3.634400 -0.597492
H -1.703299 3.123376 0.976476
H -2.550286 2.667594 -0.523706
C 2.987850 -1.824618 -0.325409
H 4.343903 -0.409330 0.622753
H 2.904240 -0.820109 1.588167
H 1.009684 -2.219429 0.464405
H 1.235810 -2.802975 -1.198566
H 3.426343 -2.717570 0.132328
H 3.464107 -1.678089 -1.301073
C -1.487301 0.370356 0.016445
C -2.389131 -0.077086 -0.953133
C -1.299491 -0.368422 1.188240
C -3.077871 -1.271629 -0.762389
C -1.978992 -1.572518 1.366742
H -0.614196 -0.001401 1.947620
C -2.867475 -2.027668 0.393075
H -3.770244 -1.619925 -1.522154
H -1.819373 -2.149380 2.272328
H -3.398417 -2.963357 0.535133
H -2.526303 0.505508 -1.859604
133
3B-Feng-1 Eopt -3432.543426
O -2.642964 -0.350895 -0.164364
O -0.954305 -1.919734 1.245886
O -0.861177 -2.177513 -1.686243
O 1.422792 -1.368222 -0.509362
N -4.483769 0.246177 0.972430
H -5.128881 0.031899 1.728206
N -2.142719 -2.602857 1.460240
N 3.333750 -2.291619 -1.222156
H 3.787823 -2.963348 -1.834578
N -0.130039 -3.339412 -1.832133
C -4.739461 1.410007 0.153354
C -3.948381 2.551792 0.357333
C -4.211297 3.659440 -0.451344
H -3.625564 4.564642 -0.334390
C -5.225621 3.621247 -1.405528
H -5.417316 4.494561 -2.020627
C -5.996571 2.475777 -1.574423
H -6.784073 2.468348 -2.319894
C -5.765282 1.336204 -0.797692
C -6.545148 0.049236 -1.008544
H -6.655297 -0.444225 -0.035866
C -2.898683 2.596271 1.455656
C -3.435982 -0.536798 0.791779
C -3.238140 -1.630395 1.827752
H -4.156465 -2.222678 1.895461
C -2.817916 -1.122319 3.217228
H -3.700442 -0.860718 3.801865
H -2.198658 -0.227514 3.121678
C -2.019036 -2.296594 3.832427
H -2.501823 -2.690819 4.726350
C -1.965736 -3.373168 2.747364
H -2.793907 -4.081849 2.816990
C -0.153137 -4.163706 -0.571930
H 0.418407 -5.068585 -0.792180
H 0.375350 -3.582637 0.186092
C -0.676276 -4.031888 -3.058439
H -0.274477 -5.047502 -3.062602
C -0.152659 -3.175172 -4.218882
H 0.114562 -3.815108 -5.059694
C 1.078553 -2.413154 -3.663788
H 0.874384 -1.343937 -3.577333
H 1.975120 -2.549655 -4.268708
C 1.283298 -3.028055 -2.275160
H 1.783865 -3.997465 -2.357096
C 2.019582 -2.169003 -1.268309
C 4.135806 -1.560730 -0.268834
C 4.140896 -1.988507 1.066225
C 4.910511 -1.244830 1.968687
H 4.938609 -1.537349 3.014755
C 5.654983 -0.150225 1.543321
C 5.647414 0.231263 0.201900
H 6.242085 1.082034 -0.113099
C 4.874749 -0.460913 -0.732976
C 4.769836 -0.013375 -2.181364
C 3.377986 -3.213808 1.543100
H 2.965195 -3.726271 0.668110
H 4.682121 -0.906682 -2.810416
H -2.412912 1.613876 1.498457
H -0.915151 -2.471328 -4.550427
H -1.760305 -4.051152 -2.964177
H -1.017330 -3.902978 2.679778
H -1.012411 -1.977960 4.105673
C -1.792529 3.620212 1.199998
H -1.357025 3.499383 0.202114
H -2.158560 4.647362 1.291306
H -1.003749 3.491925 1.949592
C -3.558792 2.854250 2.818567
H -4.038597 3.838151 2.821365
H -4.322635 2.106200 3.050509
H -2.808270 2.834553 3.615120
C -5.753131 -0.894086 -1.928152
H -5.641510 -0.442295 -2.919591
H -4.750053 -1.095767 -1.540095
H -6.279115 -1.846675 -2.040914
C -7.952521 0.276585 -1.563643
H -8.504042 -0.667304 -1.556163
H -8.508749 1.004129 -0.965782
H -7.923984 0.629795 -2.599079
C 3.494977 0.825656 -2.362549
H 3.390314 1.150124 -3.402955
H 2.590257 0.276456 -2.079577
H 3.558533 1.717026 -1.728351
C 5.987268 0.775732 -2.665454
H 6.045623 1.757281 -2.183544
H 6.919368 0.238555 -2.469705
H 5.907646 0.944532 -3.742480
C 2.206269 -2.827503 2.455449
H 2.567879 -2.363568 3.379855
H 1.525864 -2.129720 1.956035
H 1.638290 -3.721534 2.733790
C 4.307694 -4.211853 2.245285
H 5.147165 -4.489861 1.602383
H 4.710580 -3.793668 3.172680
H 3.753068 -5.118978 2.501796
C -2.548571 -3.472412 0.294906
H -2.759544 -2.803816 -0.537273
H -3.477999 -3.955578 0.604081
C -1.546477 -4.556187 -0.092050
H -2.046456 -5.158449 -0.857424
H -1.389323 -5.237330 0.750118
Sc -0.573644 -0.683935 -0.355865
H 6.252692 0.406661 2.258005
O -0.171221 1.217646 -1.119367

C 0.743494 1.969230 -0.622183
C 1.315931 1.579554 0.639948
N 1.160670 3.038776 -1.314247
O 0.612930 0.555259 1.212091
C 2.592734 1.944281 1.254718
C 2.040503 4.026565 -0.768810
C 0.646073 3.198201 -2.682313
C 0.587079 0.525878 2.681937
C 2.569389 2.079571 2.741054
H 3.498547 1.622633 0.744526
C 1.742804 4.630257 0.454160
C 3.185385 4.389729 -1.482047
H -0.444564 3.243173 -2.658632
H 1.043439 4.125223 -3.088366
H 0.955212 2.350709 -3.298841
C 1.953200 0.776542 3.290128
H 0.196234 -0.464895 2.915103
H -0.133077 1.295499 2.983091
H 1.946855 2.933302 3.043998
H 3.574645 2.229019 3.138307
C 2.621838 5.569899 0.988592
H 0.825244 4.373973 0.974387
C 4.053765 5.334302 -0.943049
H 3.399035 3.930395 -2.442471
H 1.826621 0.828270 4.375936
H 2.619235 -0.061864 3.063673
C 3.779653 5.918337 0.295503
H 2.392690 6.034348 1.941791
H 4.948576 5.610206 -1.490555
H 4.462025 6.651648 0.711571
133
3B-Feng-2 Eopt -
3432.544378
O -2.599740 -0.394216 -0.143107
O -0.825094 -1.939302 1.206701
O -0.815724 -2.192796 -1.715425
O 1.464149 -1.370621 -0.529663
N -4.436719 0.137152 1.032927
H -5.055192 -0.098622 1.804329
N -1.997920 -2.630213 1.471263
N 3.383141 -2.277554 -1.234359
H 3.852898 -2.941414 -1.843415
N -0.069391 -3.343907 -1.870267
C -4.761695 1.281434 0.211527
C -4.013166 2.456517 0.385884
C -4.344908 3.545366 -0.423120
H -3.794728 4.475138 -0.328931
C -5.382503 3.457301 -1.348692
H -5.627263 4.317026 -1.964092
C -6.108877 2.279242 -1.488790
H -6.915047 2.232405 -2.212577
C -5.808575 1.156825 -0.710706
C -6.536682 -0.164418 -0.892564
H -6.597375 -0.656489 0.085149
C -2.934369 2.552811 1.452407
C -3.358132 -0.598645 0.837272
C -3.079250 -1.661222 1.886234
H -3.984077 -2.259734 2.035836
C -2.564081 -1.120895 3.229842
H -3.400185 -0.808656 3.855715
H -1.918116 -0.254379 3.066098
C -1.774782 -2.308216 3.834574
H -2.251957 -2.690835 4.736626
C -1.758341 -3.392456 2.752488
H -2.574433 -4.109984 2.861468
C -0.072792 -4.181146 -0.618102
H 0.492923 -5.084298 -0.859628
H 0.472300 -3.609845 0.134269
C -0.608694 -4.033023 -3.101324
H -0.195241 -5.043847 -3.114652
C -0.095979 -3.160656 -4.255273
H 0.177428 -3.790519 -5.101712
C 1.127691 -2.389220 -3.695586
H 0.912458 -1.322881 -3.601110
H 2.024972 -2.511645 -4.302394
C 1.340635 -3.013163 -2.312701
H 1.850559 -3.976834 -2.403918
C 2.068663 -2.161027 -1.293804
C 4.146244 -1.544942 -0.250265
C 4.177721 -2.038919 1.061700
C 4.851059 -1.266272 2.015039
H 4.890751 -1.604527 3.046592
C 5.484567 -0.080846 1.656877
C 5.471897 0.355390 0.332930
H 5.992853 1.270001 0.068778
C 4.790894 -0.364558 -0.651328
C 4.705364 0.124172 -2.088287
C 3.549962 -3.367066 1.457689
H 3.216544 -3.876581 0.547635
H 4.656647 -0.753963 -2.742005
H -2.389885 1.601172 1.476691
H -0.866615 -2.462630 -4.580195
H -1.692333 -4.065647 -3.005453
H -0.807526 -3.912122 2.646201
H -0.759308 -2.006462 4.093164
C -1.895247 3.637677 1.169997
H -1.482033 3.546595 0.159619
H -2.313907 4.642855 1.279240
H -1.080373 3.545188 1.895266
C -3.564462 2.772664 2.836273
H -4.089947 3.732757 2.859562
H -4.285091 1.989479 3.089004
H -2.789025 2.785301 3.608660
C -5.728884 -1.076988 -1.829336
H -5.669784 -0.629253 -2.827133
H -4.705752 -1.225843 -1.470968
H -6.211236 -2.054848 -1.918590
C -7.967768 -0.003311 -1.408701
H -8.475692 -0.971081 -1.382891
H -8.539437 0.700904 -0.797615
H -7.984162 0.346424 -2.445562
C 3.417975 0.937744 -2.290902
H 3.332939 1.269655 -3.330806
H 2.519948 0.364817 -2.036843
H 3.445078 1.826835 -1.650095
C 5.918507 0.952420 -2.517343
H 5.940133 1.923776 -2.012372
H 6.856528 0.432015 -2.305426
H 5.866907 1.144807 -3.592215
C 2.324840 -3.178837 2.363068
H 2.599687 -2.665743 3.291979
H 1.539415 -2.595686 1.870751
H 1.909162 -4.155951 2.631151
C 4.581448 -4.282682 2.130576
H 5.465898 -4.411978 1.500983
H 4.903707 -3.876192 3.094087
H 4.140699 -5.266578 2.314428
C -2.444463 -3.499947 0.321506
H -2.687080 -2.830349 -0.501054
H -3.360469 -3.986782 0.663493
C -1.454195 -4.578349 -0.108947
H -1.975949 -5.168554 -0.869207
H -1.274371 -5.271268 0.719174
Sc -0.530605 -0.683870 -0.397137
H 6.007208 0.500497 2.410077
O -0.211316 1.233520 -1.123031
C 0.664416 2.041991 -0.630715
C 1.299402 1.661482 0.593793
N 0.998214 3.141374 -1.330668
O 0.690009 0.576177 1.143395
C 2.504856 2.157297 1.258964
C 0.455137 3.264269 -2.690563
C 1.856375 4.160845 -0.817997
C 1.203912 0.159425 2.449738
C 2.464926 2.301103 2.750070
H 3.444357 2.137727 0.716946
H -0.634356 3.207503 -2.654199
H 0.833055 2.456164 -3.322369
H 0.759269 4.228620 -3.090514
C 2.965708 4.559592 -1.568932
C 1.582016 4.754949 0.415116
C 1.359807 1.390571 3.322455
H 0.462371 -0.552332 2.810232
H 2.156075 -0.352263 2.273489
H 3.443194 2.011127 3.146956
H 2.296852 3.343788 3.051954
C 3.818024 5.535519 -1.061936
H 3.164882 4.099586 -2.532390
C 2.444896 5.727938 0.916850
H 0.691626 4.468912 0.965778
H 1.602121 1.076265 4.340225
H 0.396784 1.911937 3.359495
C 3.564467 6.115312 0.183644
H 4.685148 5.839138 -1.638557
H 2.232478 6.186929 1.876680
H 4.233874 6.874185 0.574174
133
3B-Feng-3 Eopt -
3432.544112
O -2.435212 -0.463386 -0.085438
O -0.531571 -1.920972 1.178624
O -0.593833 -2.447504 -1.659824
O 1.624762 -1.289874 -0.619624
N -4.250406 0.006959 1.151014
H -4.786117 -0.211933 1.986741
N -1.658133 -2.597087 1.608930
N 3.586715 -2.283531 -1.023369
H 4.098756 -3.040757 -1.466680
N 0.205208 -3.573280 -1.672391
C -4.832229 0.933104 0.205291
C -4.259040 2.204274 0.042654
C -4.879093 3.061733 -0.870496
H -4.477275 4.053987 -1.038169
C -6.016562 2.667478 -1.570271
H -6.482403 3.355565 -2.268273
C -6.558502 1.400894 -1.382508
H -7.443362 1.110343 -1.937783
C -5.970944 0.499659 -0.490633
C -6.493252 -0.919151 -0.332293
H -6.347731 -1.227586 0.709445
C -3.049605 2.645834 0.855215
C -3.122462 -0.646903 0.950321
C -2.706427 -1.603863 2.053762
H -3.576155 -2.197074 2.354700
C -2.052243 -0.946177 3.275899
H -2.812685 -0.536240 3.940728
H -1.398256 -0.131319 2.953978
C -1.245229 -2.098439 3.922565
H -1.689171 -2.415151 4.866377
C -1.285888 -3.256194 2.916470
H -2.063794 -3.987428 3.145570
C 0.184957 -4.299823 -0.352868
H 0.755928 -5.217495 -0.511630
H 0.720147 -3.667777 0.355441
C -0.253500 -4.401938 -2.850002
H 0.198427 -5.390631 -2.746071
C 0.273740 -3.629035 -4.064292
H 0.592008 -4.327103 -4.838405
C 1.456383 -2.773741 -3.541389
H 1.218002 -1.708372 -3.579232
H 2.382048 -2.944759 -4.091715
C 1.621527 -3.228239 -2.085174
H 2.177729 -4.168697 -2.036744

C 2.275717 -2.207772 -1.175506
C 4.292502 -1.253574 -0.294865
C 4.635912 -1.505068 1.042553
C 5.193446 -0.447820 1.767129
H 5.470445 -0.593758 2.805977
C 5.395871 0.796656 1.172912
C 5.088172 0.995389 -0.170157
H 5.296567 1.957441 -0.631284
C 4.538939 -0.034080 -0.942045
C 4.268546 0.173886 -2.423194
C 4.376044 -2.860746 1.680386
H 4.402784 -3.614531 0.886089
H 3.961631 -0.783921 -2.854279
H -2.326097 1.818205 0.859010
H -0.503249 -2.987079 -4.477639
H -1.338114 -4.471068 -2.792673
H -0.330837 -3.756110 2.762341
H -0.218593 -1.789700 4.122466
C -2.344659 3.862169 0.250437
H -2.098195 3.705293 -0.804632
H -2.963211 4.761587 0.326970
H -1.418392 4.068754 0.793921
C -3.446448 2.943289 2.309640
H -4.141080 3.788802 2.341754
H -3.931330 2.088222 2.787893
H -2.560873 3.203117 2.898246
C -5.676024 -1.868553 -1.223578
H -5.840870 -1.623847 -2.278172
H -4.601705 -1.784985 -1.031336
H -5.979102 -2.906669 -1.058445
C -7.986211 -1.058203 -0.635474
H -8.318545 -2.066013 -0.372738
H -8.581399 -0.340435 -0.064047
H -8.195914 -0.912313 -1.699465
C 3.134120 1.176581 -2.665820
H 2.905196 1.229653 -3.734847
H 2.223935 0.881022 -2.133707
H 3.424627 2.181272 -2.338723
C 5.543241 0.609619 -3.157852
H 5.878467 1.594486 -2.818196
H 6.356483 -0.103088 -2.996484
H 5.348633 0.675164 -4.232075
C 2.978256 -2.902449 2.315515
H 2.926067 -2.202993 3.158596
H 2.196999 -2.621569 1.600833
H 2.759710 -3.906570 2.691764
C 5.439833 -3.247235 2.711131
H 6.447826 -3.164421 2.295671
H 5.382189 -2.618359 3.604728
H 5.282279 -4.281454 3.028869
C -2.177289 -3.555648 0.568334
H -2.466802 -2.954381 -0.291494
H -3.072080 -4.012016 0.998969
C -1.201663 -4.661230 0.170984
H -1.732176 -5.275840 -0.563172
H -1.026831 -5.321949 1.026080
Sc -0.405939 -0.773163 -0.532177
H 5.819422 1.609239 1.755174
O -0.162289 1.141442 -1.312060
C 0.385459 2.121784 -0.690356
C 0.682821 1.946449 0.707285
N 0.620834 3.265343 -1.343811
O 0.471323 0.655287 1.101706
C 1.026443 2.914335 1.750259
C 0.163689 3.364349 -2.736764
C 1.385063 4.334685 -0.774981
C 1.455064 0.140867 2.076977
C 2.106427 2.484406 2.687441
H 0.252905 3.600059 2.086205
H 0.733360 2.669381 -3.359691
H 0.332365 4.383345 -3.077304
H -0.896015 3.112694 -2.801271
C 2.672543 4.090876 -0.294324
C 0.829520 5.613384 -0.718207
C 1.695231 1.099198 3.223994
H 1.028281 -0.808331 2.393677
H 2.369543 -0.036132 1.498054
H 3.063355 2.387280 2.153988
H 2.235334 3.201279 3.499033
C 3.403875 5.133848 0.268989
H 3.090058 3.089251 -0.354168
C 1.569807 6.650660 -0.157645
H -0.174890 5.787048 -1.093552
H 2.490627 0.670585 3.843512
H 0.796341 1.186276 3.843917
C 2.853726 6.412828 0.338344
H 4.402516 4.943192 0.648656
H 1.140249 7.645015 -0.102475
H 3.424132 7.225843 0.774469
133
3B-Feng-4 Eopt -
3432.544699
O 2.600444 -0.308594 0.077043
O 0.914888 -1.937340 -1.250104
O 0.825508 -2.235305 1.664428
O -1.439937 -1.387638 0.405876
N 4.505274 0.176192 -0.999569
H 5.168329 -0.085616 -1.724163
N 2.099192 -2.611807 -1.500587
N -3.369221 -2.244583 1.174682
H -3.823064 -2.914561 1.789823
N 0.080308 -3.392629 1.767761
C 4.804128 1.324295 -0.172710
C 4.061979 2.501050 -0.367408
C 4.374989 3.587961 0.451717
H 3.828930 4.518490 0.345589
C 5.388339 3.498560 1.403779
H 5.618259 4.358197 2.025006
C 6.108091 2.319415 1.563563
H 6.894219 2.270681 2.308872
C 5.824819 1.197437 0.778531
C 6.539192 -0.127700 0.986499
H 6.635535 -0.620840 0.012070
C 3.007773 2.598882 -1.459570
C 3.413922 -0.549967 -0.849066
C 3.182216 -1.632114 -1.888622
H 4.097527 -2.221688 -2.001781
C 2.708203 -1.102978 -3.251615
H 3.564057 -0.801319 -3.855801
H 2.064708 -0.230655 -3.114393
C 1.927389 -2.287772 -3.869884
H 2.418451 -2.670419 -4.764397
C 1.892388 -3.372098 -2.789542
H 2.715104 -4.084607 -2.882025
C 0.131403 -4.203729 0.499689
H -0.433782 -5.115817 0.705663
H -0.392212 -3.622968 -0.260736
C 0.581842 -4.110480 2.999500
H 0.171837 -5.122424 2.973514
C 0.029912 -3.269999 4.158247
H -0.273313 -3.922469 4.976925
C -1.172375 -2.483261 3.576980
H -0.951150 -1.415663 3.516589
H -2.089476 -2.616670 4.151417
C -1.339703 -3.069125 2.169413
H -1.857068 -4.032036 2.215549
C -2.050031 -2.173284 1.172773
C -4.186110 -1.417865 0.318182
C -4.399108 -1.831867 -1.003012
C -5.152963 -0.980455 -1.819390
H -5.333630 -1.252121 -2.855004
C -5.669432 0.211952 -1.324129
C -5.479633 0.566197 0.011868
H -5.935152 1.475322 0.391067
C -4.736090 -0.247697 0.869436
C -4.551016 0.071383 2.347101
C -3.842899 -3.139793 -1.544522
H -3.550976 -3.763574 -0.693248
H -4.560853 -0.884843 2.883466
H 2.423476 1.669572 -1.452269
H 0.788553 -2.581860 4.529211
H 1.668056 -4.137706 2.938130
H 0.942097 -3.896865 -2.705213
H 0.917235 -1.983049 -4.144533
C 2.012055 3.740474 -1.248677
H 1.537248 3.695579 -0.262211
H 2.491565 4.718501 -1.352869
H 1.235124 3.679832 -2.016312
C 3.671768 2.739743 -2.838572
H 4.222202 3.684526 -2.892011
H 4.377045 1.929884 -3.044799
H 2.912318 2.739173 -3.626670
C 5.690972 -1.035167 1.892115
H 5.597547 -0.586942 2.887006
H 4.680616 -1.177182 1.496326
H 6.162592 -2.016444 1.998991
C 7.950238 0.024735 1.557424
H 8.452581 -0.946232 1.551305
H 8.549523 0.725131 0.968873
H 7.928549 0.374537 2.594141
C -3.199241 0.745799 2.634294
H -2.986948 0.721423 3.707745
H -2.366799 0.269055 2.105652
H -3.231890 1.795918 2.326918
C -5.684501 0.923760 2.921618
H -5.670902 1.937932 2.508446
H -6.663937 0.481101 2.720741
H -5.561601 1.011447 4.004474
C -2.590080 -2.901784 -2.401248
H -2.839050 -2.306158 -3.286829
H -1.812855 -2.374508 -1.838379
H -2.179465 -3.857840 -2.741576
C -4.893931 -3.924005 -2.338538
H -5.807153 -4.068046 -1.754849
H -5.158042 -3.412743 -3.269137
H -4.495720 -4.907098 -2.604757
C 2.527674 -3.486023 -0.348629
H 2.739906 -2.821314 0.486905
H 3.458834 -3.959181 -0.668049
C 1.536163 -4.579815 0.039665
H 2.036668 -5.170089 0.813921
H 1.397129 -5.268759 -0.799300
Sc 0.570146 -0.705833 0.373505
H -6.247518 0.860848 -1.974723
O 0.084305 1.129858 1.198199
C -0.639635 2.005126 0.589707
C -0.834511 1.850856 -0.820087
N -1.093586 3.067685 1.276570
O -0.296473 0.680100 -1.269929
C -1.449942 2.725351 -1.817479
C -0.612789 3.235005 2.655131
C -2.072918 3.965900 0.754016
C -0.715328 0.286375 -2.625406
C -2.434784 2.095933 -2.754584
H -1.050369 3.719773 -1.980135
H -0.869458 2.349379 3.241305
H 0.471466 3.364265 2.660629
H -1.099523 4.109850 3.079866
C -1.832658 5.340777 0.799938
C -3.260947 3.467510 0.213779
C -2.195994 0.578017 -2.769994
H -0.467778 -0.772452 -2.681106
H -0.102744 0.856987 -3.330620

H -2.290273 2.522621 -3.752308
H -3.472366 2.315497 -2.463272
C -2.785564 6.215756 0.287362
H -0.901191 5.714658 1.214200
C -4.206573 4.351702 -0.301570
H -3.444512 2.395571 0.199713
H -2.566280 0.128913 -3.695041
H -2.717130 0.093214 -1.935209
C -3.970943 5.724552 -0.266000
H -2.598629 7.283827 0.312188
H -5.127255 3.963017 -0.726020
H -4.708479 6.412713 -0.664644
35
3B-ScCl3-1 Eopt -
2850.793824
C 5.165836 -0.768160 0.670756
C 5.434188 -0.026650 -0.482027
C 4.433154 0.178856 -1.430815
C 3.160378 -0.350516 -1.228435
C 2.895999 -1.069419 -0.061404
C 3.895146 -1.294160 0.886004
H 5.944683 -0.928469 1.408622
H 6.425066 0.385227 -0.641950
H 4.641442 0.745474 -2.332214
H 2.372903 -0.208850 -1.963573
H 3.667526 -1.853110 1.788600
N 1.583500 -1.598566 0.158021
C 0.501650 -0.804914 0.140779
O -0.666125 -1.321850 0.017420
C 1.392884 -3.052752 0.141065
H 0.557150 -3.315592 0.787550
H 1.186759 -3.393549 -0.877651
H 2.308610 -3.518613 0.501631
Sc -2.442094 -0.282079 -0.048261
Cl -3.038653 1.084045 -1.921469
Cl -3.073910 0.398002 2.141725
Cl -3.730875 -2.240393 -0.459212
C 0.601353 0.613387 0.327388
C 1.722704 1.410203 0.836846
O -0.607496 1.213819 0.198016
C 1.844213 2.773584 0.242787
H 2.001967 1.248770 1.876157
C -0.623976 2.624764 -0.219326
C 0.468414 3.444138 0.439283
H 2.068338 2.711529 -0.830725
H 2.632671 3.350893 0.727544
H -1.623169 2.969746 0.042499
H -0.518558 2.606858 -1.307868
H 0.449329 4.438584 -0.017446
H 0.260532 3.560695 1.507516
35
3B-ScCl3-2 Eopt -
2850.796868
O -0.650566 1.120674 -0.442077
C 0.530855 -0.829147 -0.194682
C 0.585242 0.569996 -0.489523
C -0.702486 2.584680 -0.424601
N 1.631206 -1.598534 -0.149178
C 1.690470 1.449114 -0.867002
C 0.409035 3.094873 0.476355
H -1.698008 2.813714 -0.048014
H -0.604219 2.925528 -1.458922
C 2.935884 -1.030396 0.008497
C 1.473080 -3.050613 -0.019819
C 1.788546 2.748756 -0.121902
H 2.142546 1.323913 -1.845923
H 0.298248 4.174512 0.601579
H 0.283063 2.633064 1.461976
C 3.218674 -0.257400 1.135288
C 3.911642 -1.276680 -0.957880
H 1.275781 -3.317541 1.022559
H 0.643063 -3.382079 -0.641767
H 2.399125 -3.521617 -0.346184
H 2.114697 3.527774 -0.817076
H 2.542988 2.695683 0.674998
C 4.487082 0.301583 1.278770
H 2.447843 -0.096211 1.884365
C 5.177383 -0.718502 -0.803201
H 3.668444 -1.879146 -1.827855
C 5.464286 0.074436 0.310360
H 4.710107 0.910145 2.148873
H 5.937557 -0.895068 -1.556702
H 6.451222 0.510395 0.424000
O -0.627122 -1.368963 -0.045576
Sc -2.423994 -0.360634 0.003979
Cl -3.464964 0.695387 -1.869066
Cl -2.708974 1.016157 1.931853
Cl -3.750986 -2.278479 0.478509
35
3B-ScCl3-3 Eopt -
2850.797411
O -0.666265 1.113702 -0.461242
C 0.521062 -0.835170 -0.228861
C 0.569411 0.563082 -0.531541
C -0.718487 2.576870 -0.373261
N 1.625482 -1.597436 -0.166085
C 1.664218 1.436435 -0.950253
C 0.448512 3.059987 0.469903
H -1.687710 2.783186 0.078515
H -0.695031 2.963263 -1.395909
C 2.926301 -1.019970 -0.011738
C 1.472955 -3.046664 -0.001286
C 1.784255 2.747683 -0.230609
H 2.096188 1.289803 -1.935368
H 0.338957 4.133482 0.640952
H 0.393695 2.562329 1.444914
C 3.195913 -0.208209 1.091022
C 3.913101 -1.296802 -0.958505
H 1.268185 -3.286672 1.046300
H 0.649250 -3.398130 -0.620536
H 2.403657 -3.522053 -0.307098
H 2.028635 3.526833 -0.959478
H 2.602864 2.729817 0.501926
C 4.461530 0.358884 1.227838
H 2.417505 -0.023362 1.826884
C 5.176158 -0.731191 -0.809786
H 3.680607 -1.930042 -1.809351
C 5.449544 0.100890 0.278339
H 4.673980 0.998186 2.078374
H 5.944736 -0.932814 -1.548319
H 6.434234 0.543154 0.386950
O -0.634530 -1.378138 -0.084038
Sc -2.430352 -0.362356 -0.004194
Cl -2.459927 0.673447 2.135119
Cl -3.634044 1.080075 -1.493140
Cl -3.769041 -2.327929 0.084879
35
3B-ScCl3-4 Eopt -
2850.796574
O 0.634277 1.160799 0.356326
C -0.492749 -0.821473 0.138835
C -0.576385 0.568072 0.461450
C 0.647800 2.622089 0.442417
N -1.578828 -1.611649 0.081301
C -1.686637 1.388699 0.944402
C -0.530397 3.162148 -0.349656
H 1.611653 2.905492 0.023013
H 0.605181 2.884984 1.503130
C -2.889814 -1.060688 -0.078034
C -1.392992 -3.057366 -0.077349
C -1.859398 2.735061 0.305712
H -2.101055 1.170777 1.923621
H -0.454285 4.250701 -0.401979
H -0.457028 2.776387 -1.372430
C -3.161712 -0.227783 -1.164905
C -3.883839 -1.377860 0.848772
H -1.189380 -3.298367 -1.124873
H -0.557555 -3.385704 0.538849
H -2.309843 -3.553753 0.237057
H -2.155190 3.451541 1.077913
H -2.664344 2.723424 -0.441895
C -4.436092 0.317989 -1.305796
H -2.378655 -0.011429 -1.886942
C -5.155556 -0.833294 0.695772
H -3.650756 -2.024603 1.689195
C -5.431303 0.018811 -0.376227
H -4.649452 0.972620 -2.144361
H -5.929437 -1.066243 1.419394
H -6.422919 0.444559 -0.487784
O 0.677071 -1.332946 -0.027172
Sc 2.448160 -0.289399 -0.064181
Cl 3.788480 -2.189440 -0.581530
Cl 2.890769 1.246619 -1.857082
Cl 3.141172 0.348073 2.125742
35
3B-ScCl3-5 Eopt -
2850.795198
C -5.193517 -0.607527 -0.621756
C -5.349729 0.054503 0.598168
C -4.273757 0.162881 1.478569
C -3.037796 -0.384413 1.141211
C -2.885727 -1.021388 -0.091802
C -3.960251 -1.149660 -0.972102
H -6.030869 -0.693183 -1.306052
H -6.311844 0.479934 0.863486
H -4.394874 0.666998 2.431562
H -2.192894 -0.318606 1.821727
H -3.819662 -1.647551 -1.926705
N -1.611631 -1.570291 -0.448311
C -0.504831 -0.811953 -0.443624
O 0.650873 -1.365034 -0.434595
C -1.469514 -3.027591 -0.532584
H -0.674247 -3.273137 -1.234302
H -1.227196 -3.438975 0.451641
H -2.416228 -3.441334 -0.876565
Sc 2.413153 -0.357565 -0.045267
Cl 3.760769 -2.320002 -0.040818
Cl 2.123463 0.179639 2.248495
C -0.574587 0.620300 -0.515463
C -1.694992 1.476397 -0.919462
O 0.654392 1.188599 -0.401373
C -1.773563 2.789256 -0.214602
H -2.018254 1.398121 -1.955582
C 0.694798 2.540022 0.185360
C -0.385299 3.445120 -0.372995
H -1.982667 2.642881 0.853960
H -2.556445 3.421168 -0.636240
H 1.700981 2.892332 -0.037066
H 0.581762 2.390926 1.263558
H -0.336297 4.390416 0.176406
H -0.190407 3.658908 -1.428630
Cl 3.791346 1.332587 -1.031099
35
3B-ScCl3-6 Eopt -
2850.795149
C -5.191061 -0.611527 -0.735875
C -5.417213 0.075593 0.458867
C -4.397191 0.194275 1.402439
C -3.146568 -0.367151 1.152894
C -2.923985 -1.029327 -0.055308
C -3.942796 -1.168962 -0.997969
H -5.984920 -0.704784 -1.469291
H -6.390499 0.512920 0.655273

H -4.573321 0.718483 2.335794
H -2.343891 -0.291720 1.881579
H -3.746711 -1.686478 -1.932171
N -1.633717 -1.590957 -0.324353
C -0.527606 -0.832899 -0.308652
O 0.626649 -1.386213 -0.248181
C -1.492498 -3.050428 -0.356720
H -0.666188 -3.319225 -1.012538
H -1.297963 -3.431262 0.650101
H -2.423760 -3.472724 -0.731262
Sc 2.414683 -0.361483 -0.053629
Cl 3.772566 -2.315192 -0.086442
C -0.591160 0.597541 -0.424961
C -1.697656 1.455990 -0.860358
O 0.640543 1.161237 -0.312176
C -1.774216 2.779460 -0.174216
H -1.999947 1.369905 -1.902256
C 0.688984 2.519602 0.256389
C -0.379608 3.424416 -0.324111
H -1.998318 2.649162 0.893310
H -2.546829 3.411465 -0.614308
H 1.699473 2.861128 0.035317
H 0.568091 2.387410 1.335820
H -0.331077 4.376094 0.214196
H -0.171212 3.623842 -1.379969
Cl 3.659553 1.283302 -1.270791
Cl 2.423920 0.302709 2.223433
35
3C-ScCl3-con-1 Eopt -2850.817496
O 0.573158 1.117161 0.155563
C -0.493425 -0.872184 -0.284273
C -0.620101 0.517255 0.016756
C 0.532756 2.520852 0.550283
N -1.601151 -1.633052 -0.394177
C -1.900903 1.247751 0.183152
C -0.384982 3.245886 -0.411637
H 1.566918 2.853286 0.487770
H 0.188409 2.553583 1.588429
C -2.874442 -1.050313 -0.163595
C -1.464658 -3.078999 -0.563679
C -1.825683 2.707593 -0.332777
H -2.145446 1.258599 1.256010
H -0.358320 4.314659 -0.187373
H 0.022869 3.113276 -1.418832
C -2.997807 0.402232 -0.534013
C -3.915472 -1.785933 0.322704
H -0.505005 -3.284409 -1.031902
H -1.508605 -3.584248 0.405528
H -2.276029 -3.430651 -1.200930
H -2.427323 3.333697 0.329527
H -2.282038 2.757707 -1.326195
C -4.371921 0.942100 -0.279047
H -2.768164 0.497257 -1.611727
C -5.186495 -1.192616 0.528369
H -3.776700 -2.833851 0.566363
C -5.382285 0.177215 0.222206
H -4.543215 1.985943 -0.524949
H -5.998528 -1.790321 0.923093
H -6.359125 0.622174 0.384007
O 0.676156 -1.370866 -0.414891
Sc 2.436051 -0.326636 -0.037288
Cl 2.568384 -0.060679 2.312232
Cl 3.883496 -2.139035 -0.564054
Cl 3.462195 1.568457 -1.074566
35
3C-ScCl3-con-2 Eopt -2850.820944
O -0.681166 -1.411757 -0.014876
C 0.487438 -0.898577 -0.036194
C 0.619869 0.521317 -0.154908
N 1.590355 -1.670710 0.034503
O -0.573834 1.136445 -0.223916
C 1.930791 1.216876 -0.260182
C 1.448056 -3.126839 0.022098
C -0.655901 2.570603 0.072515
C 1.832669 2.667054 0.216582
H 2.232661 1.216225 -1.319836
H 0.442569 -3.380245 0.348243
H 2.182453 -3.553166 0.705606
H 1.610438 -3.516649 -0.986634
C 0.583448 3.304082 -0.389956
H -1.563685 2.892621 -0.436393
H -0.791995 2.640041 1.155668
H 1.774332 2.695117 1.312397
H 2.728162 3.214273 -0.085295
H 0.483880 4.346481 -0.075485
H 0.641675 3.293243 -1.483970
C 2.879186 -1.078956 0.006794
C 2.957328 0.334928 0.516069
C 3.966779 -1.770692 -0.439687
C 4.349224 0.885151 0.459884
H 2.613296 0.346622 1.566667
C 5.250900 -1.168818 -0.456016
H 3.857874 -2.788138 -0.799250
C 5.409626 0.163216 -0.002367
H 4.495055 1.893234 0.834927
H 6.101222 -1.733437 -0.817465
H 6.398012 0.612044 -0.011359
Sc -2.450718 -0.330668 -0.051704
Cl -3.820879 -2.273052 -0.052412
Cl -3.386640 1.102454 -1.714432
Cl -2.744823 0.638078 2.091491
35
3C-ScCl3-con-3 Eopt -2850.818398
O -0.588967 1.073659 -0.487186
C 0.502459 -0.902180 -0.055134
C 0.613364 0.502351 -0.297629
C -0.633988 2.534140 -0.511411
N 1.614804 -1.645811 0.106677
C 1.892379 1.258324 -0.353932
C 0.310004 3.059401 0.547275
H -1.677895 2.777156 -0.321079
H -0.359166 2.847663 -1.523242
C 2.890704 -1.037616 -0.004394
C 1.492726 -3.095716 0.255411
C 1.758248 2.701141 0.204357
H 2.204658 1.306074 -1.407509
H 0.190865 4.142956 0.622322
H 0.013964 2.625062 1.509140
C 2.959098 0.408323 0.404236
C 3.982329 -1.753409 -0.402439
H 0.502888 -3.322298 0.644097
H 1.624236 -3.593144 -0.709663
H 2.256506 -3.436825 0.954313
H 2.147049 3.403467 -0.536985
H 2.376340 2.803227 1.100970
C 4.337167 0.976347 0.245468
H 2.664838 0.471944 1.469336
C 5.257287 -1.141429 -0.490037
H 3.880704 -2.800420 -0.667252
C 5.400038 0.231588 -0.170081
H 4.470417 2.024571 0.495625
H 6.110615 -1.724825 -0.812354
H 6.376982 0.697144 -0.254747
O -0.658507 -1.432362 -0.014200
Sc -2.435737 -0.360135 -0.067847
Cl -3.800248 -2.302951 0.012236
Cl -3.577921 1.083832 -1.596016
Cl -2.476376 0.706401 2.048252
35
3C-ScCl3-con-4 Eopt -2850.818168
O 0.563297 1.090831 0.369990
C -0.495751 -0.890295 -0.116608
C -0.627919 0.502369 0.171202
C 0.546364 2.532652 0.591191
N -1.600369 -1.649482 -0.258600
C -1.911383 1.243784 0.258641
C -0.347418 3.154514 -0.460503
H 1.587316 2.839264 0.504003
H 0.191678 2.698538 1.612914
C -2.879559 -1.060282 -0.088679
C -1.461930 -3.096392 -0.419204
C -1.802031 2.691222 -0.292215
H -2.203267 1.284033 1.318619
H -0.279324 4.242433 -0.388715
H 0.045141 2.863819 -1.440979
C -2.981678 0.387045 -0.485003
C -3.944037 -1.788956 0.355699
H -0.484096 -3.306048 -0.846126
H -1.548320 -3.598894 0.548537
H -2.247000 -3.447010 -1.089107
H -2.331319 3.360686 0.389593
H -2.310322 2.747275 -1.259631
C -4.363282 0.934068 -0.292236
H -2.710419 0.463091 -1.554771
C -5.221749 -1.191401 0.494712
H -3.819036 -2.834672 0.615913
C -5.398170 0.176517 0.168314
H -4.520081 1.977008 -0.551439
H -6.053319 -1.783574 0.855632
H -6.379499 0.626294 0.282403
O 0.674778 -1.395213 -0.209157
Sc 2.436602 -0.311893 0.025741
Cl 3.082532 0.342890 2.225166
Cl 3.805812 -2.182491 -0.496623
Cl 3.024741 1.300181 -1.630057
35
3C-ScCl3-con-5 Eopt -2850.820963
O -0.685152 -1.416441 -0.090390
C 0.480970 -0.899150 -0.088837
C 0.611602 0.523732 -0.185430
N 1.585293 -1.669141 -0.014651
O -0.581347 1.140383 -0.274256
C 1.922773 1.223134 -0.265110
C 1.446216 -3.125415 -0.040922
C -0.665605 2.564386 0.071374
C 1.820651 2.664303 0.237911
H 2.233531 1.242176 -1.322153
H 0.441105 -3.384317 0.282167
H 2.181087 -3.555957 0.639382
H 1.610365 -3.505944 -1.052914
C 0.574864 3.312714 -0.362443
H -1.571303 2.903188 -0.429342
H -0.805280 2.596010 1.156072
H 1.755897 2.671079 1.333717
H 2.717754 3.217363 -0.048212
H 0.469833 4.346758 -0.023078
H 0.641007 3.328955 -1.455954
C 2.873197 -1.074822 -0.025789
C 2.944836 0.331198 0.504593
C 3.964857 -1.757850 -0.475766
C 4.336110 0.884882 0.466124
H 2.593328 0.326468 1.552821
C 5.247759 -1.153148 -0.475287
H 3.860075 -2.770085 -0.850991
C 5.400828 0.172196 -0.000421
H 4.477665 1.887504 0.857041
H 6.101476 -1.710420 -0.840126
H 6.388253 0.623227 0.003378

Sc -2.452359 -0.325629 -0.085362
Cl -3.858945 -2.244283 -0.098359
Cl -3.513443 1.244497 -1.539535
Cl -2.528474 0.442005 2.158514
35
3C-ScCl3-con-6 Eopt -
2850.819796
O 0.698332 -1.366961 -0.300118
C -0.473447 -0.869281 -0.197188
C -0.616767 0.539276 0.010702
N -1.571696 -1.650397 -0.264378
O 0.569500 1.170631 0.027287
C -1.932431 1.204783 0.211051
C -1.415020 -3.104125 -0.317434
C 0.625486 2.632480 -0.084808
C -1.867803 2.685943 -0.159435
H -2.198061 1.123984 1.277055
H -0.417941 -3.333024 -0.684559
H -2.166349 -3.510125 -0.994938
H -1.542226 -3.536434 0.679011
C -0.616323 3.288258 0.475520
H 1.534503 2.909658 0.447543
H 0.751957 2.839251 -1.150662
H -1.831341 2.799156 -1.250700
H -2.764369 3.194610 0.200572
H -0.542372 4.358204 0.263248
H -0.648182 3.168673 1.564123
C -2.864708 -1.077204 -0.162199
C -2.973532 0.361615 -0.589032
C -3.929795 -1.801819 0.286997
C -4.367345 0.893284 -0.458525
H -2.660667 0.438639 -1.646346
C -5.217148 -1.214038 0.381632
H -3.800323 -2.834971 0.590305
C -5.404156 0.137652 0.003330
H -4.534673 1.917570 -0.776338
H -6.048819 -1.804781 0.744754
H -6.395474 0.574970 0.070212
Sc 2.452666 -0.297755 -0.032689
Cl 3.882412 -2.141916 -0.474224
Cl 3.461579 1.450331 -1.303461
Cl 2.593392 0.045112 2.306085
35
3C-ScCl3-con-7 Eopt -
2850.820666
O 0.663211 -1.417714 -0.221899
C -0.502462 -0.902779 -0.144694
C -0.625733 0.511866 0.027224
N -1.611380 -1.667855 -0.203536
O 0.572743 1.118533 0.058288
C -1.925814 1.208486 0.215265
C -1.474551 -3.123965 -0.240405
C 0.661370 2.567592 -0.141404
C -1.833002 2.675685 -0.205922
H -2.182193 1.168798 1.286070
H -0.488930 -3.370108 -0.627287
H -2.246121 -3.528332 -0.895589
H -1.583753 -3.542884 0.763913
C -0.562544 3.278054 0.393257
H 1.580831 2.849975 0.370955
H 0.780383 2.709444 -1.219039
H -1.806279 2.749438 -1.300811
H -2.714953 3.215627 0.144802
H -0.468259 4.335073 0.130417
H -0.588818 3.210382 1.486522
C -2.894365 -1.072513 -0.093990
C -2.990470 0.359984 -0.547025
C -3.961456 -1.774366 0.385434
C -4.375361 0.911952 -0.402549
H -2.695933 0.410496 -1.611215
C -5.239036 -1.168061 0.490937
H -3.839754 -2.803634 0.705061
C -5.413899 0.179227 0.091103
H -4.534700 1.932866 -0.735042
H -6.071993 -1.740769 0.879123
H -6.397738 0.631545 0.167743
Sc 2.434803 -0.346713 0.009786
Cl 3.786097 -2.303732 -0.007275
Cl 3.013926 0.787270 2.021032
Cl 3.220172 0.999668 -1.777338
35
3C-ScCl3-con-8 Eopt -
2850.818299
Sc -2.433069 -0.368869 -0.101407
O -0.659561 -1.447776 -0.060427
C 0.503477 -0.918086 -0.086601
C 0.618390 0.490291 -0.301873
N 1.616040 -1.664519 0.061732
O -0.576585 1.063417 -0.517669
C 1.893527 1.254032 -0.301398
C 1.499621 -3.119115 0.157664
C 2.890332 -1.046813 -0.018139
C -0.617295 2.522999 -0.536745
C 1.737334 2.674157 0.307727
H 2.229453 1.344023 -1.344915
H 0.507782 -3.364319 0.529674
H 1.641407 -3.580187 -0.823959
H 2.259812 -3.482140 0.849369
C 3.988680 -1.737638 -0.440465
C 2.946491 0.381528 0.448879
C 0.273191 3.039159 0.572280
H -1.669010 2.767782 -0.397199
H -0.291487 2.841479 -1.531813
H 2.184006 3.399782 -0.376163
H 2.295998 2.728834 1.246768
C 5.260770 -1.114610 -0.489004
H 3.895450 -2.772291 -0.752966
C 4.324704 0.960325 0.342400
H 2.628065 0.403147 1.508371
H 0.155385 4.122836 0.647427
H -0.077230 2.600484 1.513110
C 5.394978 0.240987 -0.097987
H 6.119953 -1.677068 -0.832332
H 4.449977 1.994424 0.649449
H 6.371523 0.712588 -0.146095
Cl -3.880050 -2.202834 0.316823
Cl -3.482090 0.955715 -1.785807
Cl -2.479915 0.815948 1.953869
35
3C-ScCl3-dis-1 Eopt -
2850.815019
O -0.559518 -1.127148 0.223624
C 0.460030 0.945009 0.195685
C 0.593072 -0.458989 0.393135
C -0.397836 -2.562147 -0.014266
N 1.574991 1.698320 0.112574
C 1.841557 -1.204722 0.641405
C 0.720691 -2.752169 -1.031133
H -1.366483 -2.887997 -0.389417
H -0.196506 -3.032752 0.951281
C 2.840257 1.049408 0.070857
C 1.455197 3.125674 -0.179317
C 2.069970 -2.160976 -0.552970
H 1.700036 -1.815759 1.544129
H 0.811385 -3.821224 -1.237109
H 0.401207 -2.269101 -1.959890
C 2.968382 -0.176856 0.935412
C 3.880373 1.563947 -0.646782
H 0.502403 3.479687 0.208388
H 2.277048 3.647790 0.310520
H 1.492364 3.304224 -1.257909
H 2.759971 -2.948634 -0.240734
H 2.547629 -1.606114 -1.367146
C 4.333363 -0.791916 0.861347
C 5.146694 0.926741 -0.641386
H 3.745218 2.466346 -1.233904
C 5.341297 -0.254459 0.119366
H 4.497421 -1.687724 1.453722
H 5.958274 1.345426 -1.223223
H 6.315438 -0.733573 0.113518
O -0.709560 1.442887 0.062749
H 2.787106 0.147600 1.976615
Sc -2.443420 0.268314 0.011905
Cl -3.205722 -0.882535 1.950678
Cl -2.919527 -1.040379 -1.919265
Cl -3.951885 2.091842 -0.142353
35
3C-ScCl3-dis-2 Eopt -
2850.812221
O 0.786761 -1.352795 -0.399887
C -0.380150 -0.898868 -0.165645
C -0.537075 0.490108 0.166081
N -1.477024 -1.668015 -0.266203
O 0.571200 1.230719 -0.064849
C -1.775830 1.131872 0.661746
C -1.344024 -3.059156 -0.699343
C 0.333791 2.451110 -0.863115
C -2.131560 2.270763 -0.308075
H -1.544618 1.591084 1.635056
H -0.370988 -3.429386 -0.383503
H -1.420616 -3.131312 -1.787889
H -2.137983 -3.641509 -0.232649
C -0.908911 3.189021 -0.401140
H 1.242664 3.035745 -0.742024
H 0.247277 2.112713 -1.900212
H -2.392626 1.857293 -1.290692
H -2.998057 2.822703 0.064958
H -1.083313 4.000702 -1.113654
H -0.720557 3.646726 0.576101
C -2.756843 -1.059770 -0.143420
C -2.848380 0.028771 0.890111
C -3.828570 -1.503033 -0.861920
C -4.233225 0.589766 1.006405
C -5.105557 -0.916875 -0.681657
H -3.709374 -2.307594 -1.580466
C -5.276601 0.129598 0.261700
H -4.380669 1.378871 1.738391
H -5.945115 -1.272435 -1.265836
H -6.262130 0.564656 0.395619
H -2.575090 -0.431036 1.857665
Sc 2.477105 -0.187518 0.007786
Cl 3.991660 -2.002728 -0.226696
Cl 3.584876 1.576968 -1.175783
Cl 2.203764 -0.001679 2.359924
35
3C-ScCl3-dis-3 Eopt -
2850.812941
O -0.718112 -1.450389 -0.267634
C 0.450399 -0.947098 -0.314427
C 0.594650 0.476440 -0.410737
N 1.560314 -1.700166 -0.226807
O -0.561808 1.144783 -0.202293
C 1.839113 1.210969 -0.738983
C 1.436566 -3.142580 -0.018088
C -0.452981 2.331766 0.673235
C 2.050887 2.286341 0.338520
H 1.668096 1.740738 -1.688932
H 0.518813 -3.484087 -0.492397
H 1.396318 -3.374944 1.049934
H 2.298901 -3.630383 -0.471691
C 0.785876 3.150125 0.366568
H -1.380534 2.875191 0.497879
H -0.438285 1.935910 1.692475

H 2.229824 1.813570 1.312578
H 2.924458 2.895570 0.094369
H 0.856330 3.921925 1.138614
H 0.667225 3.659203 -0.595945
C 2.818933 -1.047242 -0.111783
C 2.982083 0.177903 -0.968939
C 3.821297 -1.562745 0.657241
C 4.345492 0.787282 -0.837777
C 5.084556 -0.925015 0.712475
H 3.655266 -2.465642 1.236075
C 5.316258 0.251351 -0.047284
H 4.543542 1.676279 -1.429892
H 5.867031 -1.339179 1.335957
H 6.290821 0.727358 -0.000776
H 2.850416 -0.155213 -2.014850
Sc -2.446578 -0.256280 -0.113146
Cl -3.945543 -2.026060 -0.629140
Cl -3.523452 1.689509 -1.003453
Cl -2.512869 0.021645 2.236100
35
3C-ScCl3-dis-4 Eopt -2850.811887
O 0.741415 -1.377807 -0.418509
C -0.406451 -0.897104 -0.160134
C -0.518697 0.503680 0.143571
N -1.522644 -1.644866 -0.198993
O 0.612127 1.207988 -0.114082
C -1.746645 1.208428 0.570218
C -1.425143 -3.058275 -0.563688
C 0.402051 2.334375 -1.052944
C -2.072260 2.255936 -0.509631
H -1.518310 1.751782 1.499276
H -0.445455 -3.425326 -0.265257
H -1.544379 -3.187223 -1.643003
H -2.207595 -3.603664 -0.036439
C -0.831718 3.137844 -0.685816
H 1.321004 2.912528 -0.989335
H 0.312988 1.879582 -2.044414
H -2.335966 1.752109 -1.448567
H -2.930660 2.857430 -0.199369
H -0.985602 3.870868 -1.483372
H -0.642973 3.694523 0.238339
C -2.788882 -1.008751 -0.078612
C -2.840167 0.148787 0.880827
C -3.887481 -1.486208 -0.732121
C -4.209419 0.750918 0.976297
C -5.150629 -0.867355 -0.563950
H -3.800535 -2.343233 -1.392004
C -5.279287 0.254796 0.294913
H -4.323458 1.600559 1.643703
H -6.010779 -1.253605 -1.096361
H -6.252343 0.721126 0.413968
H -2.565076 -0.252041 1.874195
Sc 2.463015 -0.224998 0.001960
Cl 3.921292 -1.962994 -0.703262
Cl 2.144216 -0.311846 2.348962
Cl 3.732395 1.743060 -0.502902
35
3C-ScCl3-dis-5 Eopt -2850.814868
O -0.556420 -1.186721 0.313611
C 0.417309 0.906670 0.239586
C 0.584165 -0.490709 0.453129
C -0.359580 -2.621896 0.105817
N 1.509856 1.688040 0.127113
C 1.860135 -1.202754 0.664811
C 0.727421 -2.807445 -0.945282
H -1.329511 -2.986870 -0.225534
H -0.108112 -3.060028 1.074812
C 2.787950 1.069827 0.045525
C 1.343002 3.108901 -0.173757
C 2.071997 -2.158800 -0.532785
H 1.767858 -1.812068 1.574694
H 0.846169 -3.879099 -1.121759
H 0.359168 -2.364350 -1.875802
C 2.973907 -0.147657 0.911287
C 3.787396 1.602663 -0.715046
H 0.399898 3.444761 0.251936
H 2.172581 3.655050 0.274796
H 1.328581 3.276876 -1.254665
H 2.806934 -2.915415 -0.247418
H 2.490321 -1.591014 -1.370422
C 4.349032 -0.732456 0.788041
C 5.066796 0.993627 -0.755125
H 3.608421 2.497320 -1.302329
C 5.315777 -0.177867 0.004630
H 4.556183 -1.620785 1.378100
H 5.845732 1.425935 -1.370671
H 6.299208 -0.635801 -0.036114
O -0.764397 1.377691 0.117461
H 2.825176 0.179246 1.956808
Sc -2.470060 0.200299 -0.015940
Cl -3.083391 -0.450388 2.183727
Cl -2.928764 -1.343703 -1.790147
Cl -3.843824 2.075462 -0.526229
35
3C-ScCl3-dis-6 Eopt -2850.814224
O 0.560761 1.176890 0.026811
C -0.437177 -0.910929 -0.024735
C -0.572914 0.483423 0.239176
C 0.355677 2.621035 -0.110802
N -1.547540 -1.672477 -0.089287
C -1.817896 1.196439 0.590102
C -0.819624 2.851589 -1.051945
H 1.296815 2.992555 -0.509445
H 0.193567 3.024121 0.892052
C -2.819052 -1.038361 -0.039496
C -1.427673 -3.086822 -0.438986
C -2.128911 2.201950 -0.542080
H -1.636591 1.766441 1.512297
H -0.939571 3.930150 -1.178979
H -0.546018 2.440422 -2.028288
C -2.915548 0.139452 0.892858
C -3.885914 -1.525964 -0.736314
H -0.457850 -3.446178 -0.101385
H -2.225790 -3.634034 0.062536
H -1.505521 -3.226086 -1.521096
H -2.822854 2.954089 -0.159062
H -2.631949 1.673568 -1.358667
C -4.289686 0.738147 0.919815
C -5.156104 -0.904713 -0.638131
H -3.768538 -2.393731 -1.377215
C -5.325991 0.229744 0.196583
H -4.435115 1.597276 1.568633
H -5.990027 -1.299888 -1.204655
H -6.304213 0.695742 0.263415
O 0.727045 -1.390932 -0.231639
H -2.675587 -0.234069 1.905216
Sc 2.467250 -0.238699 -0.044722
Cl 3.927578 -2.041489 -0.561937
Cl 3.520478 1.630716 -1.099615
Cl 2.587955 0.035599 2.302797
35
3C-ScCl3-dis-7 Eopt -2850.815916
O 0.599708 -1.149477 -0.460396
C -0.437239 0.918118 -0.443774
C -0.568413 -0.494573 -0.573251
C 0.466654 -2.554706 -0.061537
N -1.551863 1.669853 -0.339702
C -1.826707 -1.256999 -0.688369
C -0.588131 -2.652309 1.032839
H 1.460586 -2.834762 0.284834
H 0.218337 -3.124158 -0.960769
C -2.807090 1.019325 -0.184407
C -1.421956 3.109284 -0.119975
C -1.972485 -2.131328 0.580255
H -1.741444 -1.926635 -1.555654
H -0.645617 -3.696265 1.350344
H -0.223392 -2.071292 1.885261
C -2.979519 -0.254002 -0.969183
C -3.800869 1.565382 0.574587
H -0.503602 3.450181 -0.593136
H -2.281983 3.604794 -0.570045
H -1.379639 3.335825 0.949473
H -2.655809 -2.954740 0.358302
H -2.425071 -1.530860 1.376401
C -4.330554 -0.872220 -0.768549
C -5.057700 0.920399 0.691586
H -3.634336 2.498618 1.102440
C -5.291005 -0.302056 0.011274
H -4.525037 -1.801321 -1.296950
H -5.831173 1.364336 1.305735
H -6.256802 -0.787591 0.111449
O 0.732107 1.429375 -0.390129
H -2.869947 0.010407 -2.036938
Sc 2.446383 0.269952 -0.086325
Cl 2.219014 -0.422981 2.168347
Cl 3.896963 2.153569 -0.057560
Cl 3.638111 -1.449387 -1.240480
31
A-1 Eopt -709.346757
O -2.308752 0.389722 -0.835303
C -0.491247 1.673031 -0.026622
C -1.350744 0.447545 0.129818
C -3.400532 -0.486123 -0.532893
N 0.852802 1.533966 0.170254
C -1.236171 -0.412803 1.148486
C -2.902555 -1.829027 -0.020707
H -3.964485 -0.578808 -1.461583
H -4.029191 0.003194 0.223304
C 1.544964 0.298274 -0.031087
C 1.650486 2.761795 0.181580
C -2.128016 -1.617188 1.281673
H -0.444115 -0.246945 1.871718
H -3.750639 -2.503390 0.125148
H -2.248371 -2.272868 -0.779811
C 1.354190 -0.433492 -1.204713
C 2.438022 -0.155498 0.940441
H 1.134187 3.521119 0.768117
H 2.620292 2.546064 0.628038
H 1.796244 3.138384 -0.836611
H -2.818829 -1.487212 2.124132
H -1.518471 -2.496881 1.514549
C 2.033921 -1.635946 -1.389434
H 0.669509 -0.062396 -1.962463
C 3.127761 -1.349813 0.743240
H 2.574847 0.418738 1.852333
C 2.922858 -2.096339 -0.417989
H 1.875091 -2.206758 -2.298704
H 3.817498 -1.702369 1.503313
H 3.455941 -3.029740 -0.566229
O -1.005841 2.764731 -0.253170
31
A-2 Eopt -709.346610
O -0.969318 2.750218 -0.274006
C -0.460419 1.656306 -0.044210
C -1.327090 0.434570 0.085609
N 0.878705 1.512971 0.179604
O -2.254632 0.381196 -0.910969
C -1.258000 -0.418383 1.114839
C 1.681032 2.737164 0.216823

C -2.968745 -0.858446 -0.978675
C -2.271589 -1.511269 1.317750
H -0.452529 -0.291737 1.831348
H 1.148858 3.498372 0.786375
H 1.862010 3.113037 -0.795928
H 2.634711 2.517356 0.695053
C -3.469250 -1.281402 0.393687
H -3.782447 -0.686000 -1.683868
H -2.291530 -1.620112 -1.389648
H -1.813399 -2.488240 1.116194
H -2.589195 -1.529209 2.365304
H -4.078000 -2.184596 0.298951
H -4.108918 -0.487281 0.794264
C 1.567328 0.274255 -0.012328
C 1.391976 -0.453523 -1.190994
C 2.437854 -0.189374 0.974816
C 2.065309 -1.660970 -1.366189
H 0.724619 -0.074683 -1.960474
C 3.121335 -1.388910 0.787410
H 2.562154 0.381311 1.890718
C 2.932198 -2.130773 -0.379454
H 1.918884 -2.228379 -2.279650
H 3.793528 -1.749230 1.559451
H 3.460291 -3.068190 -0.520029
133
A-Feng-1 Eopt -
3432.633942
O 2.431703 -0.536653 0.078044
O 0.510493 -2.108331 -0.969070
O 0.485513 -2.159026 1.922705
O -1.666775 -1.159441 0.616203
N 4.322509 -0.402937 -1.125505
H 4.889533 -0.801669 -1.868717
N 1.607444 -2.913217 -1.225866
N -3.666984 -2.031567 1.113193
H -4.211395 -2.674472 1.681325
N -0.365165 -3.227624 2.122429
C 4.835823 0.745293 -0.414916
C 4.252803 1.997000 -0.659883
C 4.783012 3.088730 0.032263
H 4.367482 4.078534 -0.122352
C 5.845702 2.926158 0.917372
H 6.245676 3.788620 1.440738
C 6.398531 1.668054 1.137334
H 7.224293 1.562161 1.832130
C 5.898739 0.542739 0.476370
C 6.427544 -0.854573 0.753620
H 6.386935 -1.426584 -0.180855
C 3.131117 2.164780 -1.671756
C 3.154244 -0.955462 -0.861192
C 2.730313 -2.078641 -1.788869
H 3.572382 -2.763301 -1.931374
C 2.174012 -1.618139 -3.145780
H 2.991857 -1.438724 -3.844113
H 1.613199 -0.687386 -3.028046
C 1.249112 -2.780952 -3.585915
H 1.618358 -3.273861 -4.484956
C 1.238011 -3.766991 -2.414446
H 2.001564 -4.542556 -2.506354
C -0.362936 -4.171089 0.949334
H -0.985084 -5.018179 1.246807
H -0.850597 -3.642549 0.129395
C 0.039596 -3.846650 3.439992
H -0.453393 -4.818795 3.506976
C -0.473375 -2.843773 4.482361
H -0.852645 -3.378107 5.353263
C -1.591005 -2.031482 3.777784
H -1.280326 -0.998244 3.608438
H -2.531393 -2.027549 4.329091
C -1.769732 -2.754420 2.439767
H -2.359089 -3.666230 2.572321
C -2.365724 -1.929522 1.317979
C -4.314435 -1.260570 0.075316
C -4.418514 -1.827882 -1.203015
C -4.950838 -1.024430 -2.216761
H -5.042540 -1.421406 -3.223053
C -5.358901 0.279429 -1.954236
C -5.269046 0.800882 -0.665649
H -5.610609 1.813484 -0.475173
C -4.754260 0.035561 0.384386
C -4.664975 0.599503 1.793426
C -3.949213 -3.240352 -1.509492
H -3.807310 -3.769620 -0.561712
H -4.602631 -0.241234 2.492334
H 2.493003 1.275766 -1.630152
H 0.328703 -2.181961 4.806911
H 1.120854 -3.968303 3.420449
H 0.267155 -4.213496 -2.205572
H 0.241670 -2.415230 -3.789933
C 2.221596 3.351001 -1.355049
H 1.902873 3.321118 -0.306982
H 2.719861 4.309622 -1.532127
H 1.333156 3.317665 -1.994122
C 3.698825 2.275159 -3.093820
H 4.326193 3.168131 -3.180814
H 4.309711 1.405189 -3.353485
H 2.885465 2.352173 -3.822003
C 5.518848 -1.556450 1.776061
H 5.562663 -1.031237 2.736103
H 4.473109 -1.568493 1.453206
H 5.845299 -2.588797 1.932625
C 7.880150 -0.872674 1.232481
H 8.234830 -1.905592 1.281925
H 8.534579 -0.316999 0.554982
H 7.977758 -0.445435 2.235284
C -3.395140 1.446683 1.954510
H -3.338645 1.865607 2.964790
H -2.491971 0.855630 1.774850
H -3.410168 2.271769 1.233118
C -5.901594 1.420397 2.174198
H -5.949921 2.358687 1.612927
H -6.823701 0.862097 1.991381
H -5.857825 1.676304 3.236327
C -2.598562 -3.212467 -2.240189
H -2.690848 -2.682368 -3.195903
H -1.827360 -2.705635 -1.649663
H -2.263272 -4.234019 -2.449396
C -4.984765 -4.028707 -2.319238
H -5.962894 -4.017055 -1.830933
H -5.099955 -3.621342 -3.328128
H -4.660858 -5.068469 -2.418763
C 2.047407 -3.703195 -0.019235
H 2.352574 -2.978174 0.733419
H 2.925375 -4.268581 -0.340966
C 1.011479 -4.680714 0.530566
H 1.496574 -5.186600 1.371469
H 0.820783 -5.467066 -0.206678
Sc 0.385204 -0.692717 0.526934
H -5.763842 0.889462 -2.755904
O 0.212560 1.253393 1.267130
C -0.413989 2.213528 0.719536
C -0.966572 1.939691 -0.635716
N -0.525747 3.353844 1.387432
O -0.489724 0.698697 -1.065176
C -1.813605 2.671263 -1.370975
C 0.044766 3.389616 2.748423
C -1.130351 4.565882 0.894242
C -1.283662 0.103425 -2.132914
C -2.243754 2.291164 -2.754232
H -2.203649 3.592459 -0.959717
H -0.400420 2.599798 3.356294
H -0.178372 4.364651 3.174600
H 1.125506 3.240592 2.698923
C -2.372905 4.949050 1.395609
C -0.446984 5.358863 -0.024616
C -1.450499 1.092619 -3.264663
H -0.734838 -0.793137 -2.417829
H -2.249187 -0.177043 -1.692019
H -3.318467 2.071372 -2.734601
H -2.119339 3.161811 -3.405962
C -2.960661 6.124016 0.930002
H -2.871715 4.333375 2.137285
C -1.044744 6.529082 -0.487966
H 0.530973 5.056688 -0.381163
H -1.965814 0.600868 -4.093741
H -0.461872 1.403083 -3.620367
C -2.302909 6.907590 -0.018534
H -3.931441 6.424412 1.309178
H -0.523017 7.145959 -1.211672
H -2.765264 7.818731 -0.382866
133
A-Feng-2 Eopt -
3432.634496
O 2.523079 -0.496760 0.132045
O 0.698085 -2.127995 -0.975558
O 0.532545 -2.032236 1.946216
O -1.604651 -1.195137 0.489158
N 4.433382 -0.317750 -1.037610
H 5.040349 -0.726210 -1.743430
N 1.814141 -2.938441 -1.114631
N -3.616549 -1.952098 1.137485
H -4.144019 -2.531099 1.784690
N -0.305967 -3.104039 2.176720
C 4.856604 0.907505 -0.401393
C 4.190815 2.095954 -0.733333
C 4.640779 3.267148 -0.118142
H 4.160466 4.213258 -0.342911
C 5.706459 3.240115 0.777312
H 6.043430 4.162408 1.239390
C 6.342634 2.041083 1.085261
H 7.169745 2.040935 1.786508
C 5.925434 0.841088 0.503679
C 6.548308 -0.493710 0.877148
H 6.557352 -1.128321 -0.016760
C 3.068014 2.124138 -1.758162
C 3.295205 -0.925192 -0.763092
C 2.977182 -2.129726 -1.630424
H 3.840164 -2.803235 -1.624004
C 2.573160 -1.785940 -3.076464
H 3.462404 -1.742176 -3.705313
H 2.087081 -0.808676 -3.118688
C 1.606730 -2.923120 -3.490288
H 1.966852 -3.466682 -4.363274
C 1.532173 -3.853841 -2.281026
H 2.310649 -4.620437 -2.287346
C -0.276691 -4.090010 1.038741
H -0.913814 -4.921485 1.348560
H -0.734951 -3.588382 0.184373
C 0.086812 -3.666287 3.522895
H -0.401729 -4.637806 3.622609
C -0.445543 -2.624564 4.516584
H -0.832883 -3.125145 5.403842
C -1.558454 -1.847688 3.765520
H -1.252838 -0.818392 3.565989
H -2.506134 -1.830887 4.304164
C -1.713360 -2.626295 2.455033
H -2.298023 -3.535732 2.622754
C -2.306142 -1.869398 1.281762
C -4.319880 -1.302326 0.056587
C -4.365533 -1.942929 -1.188688
C -5.038977 -1.279015 -2.220699
H -5.092231 -1.734831 -3.204893
C -5.630868 -0.039911 -2.006534

C -5.581895 0.560324 -0.748706
H -6.066926 1.518739 -0.597061
C -4.931023 -0.064387 0.316640
C -4.868404 0.563293 1.700659
C -3.704356 -3.286533 -1.449146
H -3.415085 -3.721957 -0.486812
H -4.877470 -0.248662 2.437078
H 2.525701 1.173973 -1.704539
H 0.348852 -1.945345 4.823606
H 1.168924 -3.781729 3.522904
H 0.555088 -4.303781 -2.113643
H 0.617858 -2.524112 -3.721659
C 2.038757 3.215187 -1.465426
H 1.714769 3.161873 -0.419828
H 2.440402 4.216965 -1.647635
H 1.164196 3.093175 -2.113415
C 3.635176 2.262822 -3.177641
H 4.177835 3.208324 -3.277198
H 4.326204 1.447788 -3.414382
H 2.825465 2.250424 -3.914068
C 5.681616 -1.189165 1.939509
H 5.673953 -0.596906 2.860658
H 4.644202 -1.303918 1.610124
H 6.082891 -2.180340 2.170364
C 7.993864 -0.376863 1.363296
H 8.419749 -1.376543 1.483210
H 8.614122 0.177809 0.653594
H 8.051320 0.121402 2.335981
C -3.558596 1.345334 1.874711
H -3.496737 1.771534 2.881654
H -2.677729 0.716397 1.710264
H -3.527014 2.162309 1.145533
C -6.061616 1.472068 2.004625
H -6.031175 2.387731 1.405268
H -7.012058 0.966150 1.814153
H -6.034716 1.769051 3.056417
C -2.431654 -3.117318 -2.293859
H -2.670425 -2.670912 -3.266041
H -1.693302 -2.478905 -1.795764
H -1.973628 -4.095366 -2.476629
C -4.668032 -4.274076 -2.118222
H -5.592365 -4.377033 -1.543423
H -4.928326 -3.953422 -3.131442
H -4.196364 -5.257887 -2.193508
C 2.173429 -3.669671 0.155366
H 2.435113 -2.910373 0.890225
H 3.067378 -4.249822 -0.084446
C 1.106227 -4.623386 0.684688
H 1.553097 -5.112947 1.555816
H 0.936374 -5.426130 -0.039816
Sc 0.455961 -0.671678 0.462195
H -6.143887 0.460897 -2.821712
O 0.107879 1.220947 1.295286
C -0.536313 2.152917 0.718961
C -1.014477 1.851661 -0.658308
N -0.769513 3.275164 1.383460
O -0.320317 0.729389 -1.117208
C -2.014780 2.417159 -1.343148
C -0.322830 3.325995 2.788999
C -1.368820 4.460101 0.824927
C -0.541126 0.382998 -2.514332
C -2.519414 1.871142 -2.643485
H -2.499309 3.293276 -0.930739
H -0.746600 2.484700 3.340004
H 0.767128 3.272495 2.829627
H -0.665396 4.266789 3.212786
C -0.688612 5.192231 -0.145174
C -2.612169 4.873252 1.301430
C -2.018078 0.446328 -2.838563
H -0.133580 -0.623575 -2.612664
H 0.044730 1.088937 -3.114226
H -2.189526 2.527264 -3.459021
H -3.612695 1.906316 -2.636537
C -1.287248 6.330317 -0.681199
H 0.289903 4.869798 -0.482270
C -3.199582 6.017147 0.764253
H -3.114314 4.303388 2.077281
H -2.177468 0.097545 -3.862095
H -2.553255 -0.230592 -2.163971
C -2.543530 6.739297 -0.233184
H -0.766375 6.899810 -1.443368
H -4.170224 6.340205 1.124631
H -3.006026 7.626260 -0.652794
35
A-ScCl3-1 Eopt -2850.885421
O -0.693006 1.144115 0.060167
C 0.623275 -0.752234 -0.211578
C 0.592836 0.737033 -0.257760
C -1.008993 2.535600 -0.221822
N 1.731131 -1.482634 -0.200557
C 1.567987 1.594251 -0.579986
C 0.115850 3.431710 0.249597
H -1.947253 2.716020 0.302466
H -1.167964 2.613972 -1.301987
C 3.052650 -0.956398 0.013610
C 1.587591 -2.948917 -0.217919
C 1.387076 3.081933 -0.518591
H 2.533365 1.205184 -0.874624
H -0.171545 4.472995 0.086844
H 0.265399 3.288495 1.324657
C 3.397489 -0.466031 1.271132
C 3.972785 -0.985352 -1.031673
H 0.834963 -3.233894 -0.951498
H 2.553742 -3.374090 -0.481752
H 1.284068 -3.302665 0.770837
H 1.352403 3.484242 -1.538548
H 2.270060 3.520265 -0.043489
C 4.678717 0.044052 1.470691
H 2.664438 -0.470493 2.072038
C 5.253935 -0.478858 -0.820366
H 3.677851 -1.380440 -1.999134
C 5.603873 0.042421 0.425990
H 4.953544 0.439042 2.442907
H 5.974580 -0.485917 -1.631068
H 6.599837 0.442267 0.584822
O -0.499196 -1.340780 -0.180377
Sc -2.360863 -0.414926 0.076224
Cl -3.238401 0.647573 -1.862654
Cl -3.183681 0.734345 1.998052
Cl -3.556159 -2.472803 0.133687
35
A-ScCl3-2 Eopt -2850.886356
O 0.483471 -1.377547 -0.109992
C -0.628407 -0.774968 -0.033404
C -0.559510 0.709346 0.028643
N -1.751327 -1.477245 0.026186
O 0.669277 1.099891 -0.484327
C -1.415489 1.559909 0.604099
C -1.645495 -2.945159 0.082297
C 0.792692 2.527544 -0.733020
C -1.110075 3.016870 0.785858
H -2.360460 1.178295 0.971395
H -0.856734 -3.227011 0.778179
H -1.413218 -3.337773 -0.910819
H -2.603578 -3.336806 0.417775
C 0.361563 3.303368 0.495208
H 1.838564 2.678287 -0.991600
H 0.160261 2.749036 -1.599313
H -1.762315 3.597796 0.121471
H -1.375713 3.306396 1.807001
H 0.528995 4.368864 0.320775
H 0.986421 2.996510 1.340161
C -3.062453 -0.905879 -0.121687
C -3.431606 -0.336509 -1.338091
C -3.950691 -0.964063 0.950480
C -4.703512 0.217681 -1.468630
H -2.726042 -0.320602 -2.163201
C -5.222634 -0.413613 0.807991
H -3.638165 -1.417086 1.886660
C -5.596477 0.182778 -0.397247
H -4.996925 0.670918 -2.409471
H -5.917279 -0.444769 1.640533
H -6.585851 0.615132 -0.502984
Sc 2.346041 -0.411197 -0.121398
Cl 3.712055 0.886963 -1.595694
Cl 2.484728 0.410522 2.100372
Cl 3.539984 -2.470657 -0.008262
35
A-ScCl3-3 Eopt -2850.885861
O 0.730144 1.172418 -0.083310
C -0.626915 -0.707233 0.146201
C -0.577598 0.781884 0.169971
C 1.052922 2.536058 0.307235
N -1.737311 -1.434382 0.162670
C -1.553924 1.659484 0.429527
C 0.005910 3.484649 -0.237225
H 2.045777 2.715409 -0.102879
H 1.093045 2.552590 1.401778
C -3.063778 -0.916637 -0.034338
C -1.590430 -2.900238 0.206386
C -1.346804 3.143905 0.381930
H -2.540288 1.290642 0.676046
H 0.299171 4.511212 -0.005711
H -0.032574 3.385028 -1.326763
C -3.449688 -0.494605 -1.304610
C -3.952429 -0.899414 1.038253
H -0.853535 -3.171732 0.961214
H -2.561256 -3.323110 0.455929
H -1.262862 -3.269704 -0.768754
H -1.425143 3.544276 1.400236
H -2.167744 3.591142 -0.187414
C -4.742453 -0.009579 -1.492704
H -2.739795 -0.535455 -2.125117
C -5.244869 -0.417669 0.838857
H -3.624809 -1.242741 2.014902
C -5.637258 0.032228 -0.422705
H -5.050637 0.329722 -2.475876
H -5.942151 -0.390896 1.669343
H -6.643079 0.409884 -0.573385
O 0.488446 -1.306010 0.115925
Sc 2.367602 -0.424315 -0.120208
Cl 3.393945 -2.555485 -0.422805
Cl 3.542337 0.995483 -1.644861
Cl 2.973281 0.258047 2.064755
35
A-ScCl3-4 Eopt -2850.883745
O -0.742210 1.257702 -0.092131
C 0.539632 -0.634283 -0.532425
C 0.536763 0.851024 -0.436344
C -1.011378 2.684085 -0.199682
N 1.633297 -1.385820 -0.537817
C 1.538258 1.699088 -0.692072
C 0.160945 3.490492 0.317637
H -1.918869 2.842093 0.379676
H -1.214009 2.890739 -1.255677
C 2.942452 -0.922841 -0.162360
C 1.460466 -2.838990 -0.712468
C 1.401583 3.180937 -0.514200
H 2.490892 1.298013 -1.013484

H -0.097665 4.550720 0.265559
H 0.335974 3.239383 1.368874
C 3.176819 -0.532488 1.154508
C 3.961499 -0.921766 -1.111678
H 0.753947 -3.028385 -1.519134
H 2.432771 -3.263712 -0.952697
H 1.081221 -3.279094 0.213542
H 1.346583 3.663914 -1.497577
H 2.306847 3.561592 -0.031801
C 4.449832 -0.095716 1.514554
H 2.367564 -0.560357 1.878308
C 5.233513 -0.490463 -0.739697
H 3.752044 -1.238709 -2.128950
C 5.475856 -0.071115 0.568981
H 4.639903 0.219079 2.535103
H 6.031917 -0.477789 -1.473875
H 6.466117 0.268452 0.853768
O -0.592100 -1.196992 -0.618637
Sc -2.375598 -0.359996 0.056533
Cl -2.014246 -0.411320 2.407711
Cl -3.543670 -2.392544 -0.391386
Cl -3.903479 1.256879 -0.844941
35
A-ScCl3-5 Eopt -2850.886749
O -0.458802 -1.351790 -0.336120
C 0.656991 -0.752544 -0.292379
C 0.596336 0.732000 -0.216468
N 1.782392 -1.451207 -0.352146
O -0.668219 1.072494 0.231813
C 1.490930 1.636948 -0.625279
C 1.684122 -2.907155 -0.548549
C -0.856817 2.465617 0.592897
C 1.182160 3.104423 -0.675986
H 2.466594 1.298071 -0.951668
H 0.928955 -3.122119 -1.302875
H 1.408000 -3.389993 0.392407
H 2.657857 -3.267001 -0.874867
C -0.315980 3.353359 -0.509007
H -1.930430 2.576351 0.739958
H -0.335007 2.619484 1.543204
H 1.752891 3.612308 0.111667
H 1.544869 3.507010 -1.626350
H -0.515844 4.397556 -0.257614
H -0.850970 3.121767 -1.436043
C 3.074534 -0.905799 -0.034642
C 3.332740 -0.472651 1.264085
C 4.053691 -0.852382 -1.024402
C 4.585142 0.058121 1.565687
H 2.557035 -0.542729 2.020704
C 5.305584 -0.326548 -0.711227
H 3.827626 -1.200225 -2.027968
C 5.569027 0.134832 0.579387
H 4.791867 0.405811 2.572227
H 6.071375 -0.271135 -1.477461
H 6.543170 0.548684 0.817727
Sc -2.324080 -0.441909 0.043353
Cl -3.383736 1.088838 -1.442462
Cl -3.502845 -2.480423 -0.323393
Cl -2.979517 0.201569 2.234169
35
A-ScCl3-6 Eopt -2850.886526
C -5.271342 -0.335633 -0.834694
C -5.598566 0.132632 0.438789
C -4.663788 0.066007 1.472508
C -3.396010 -0.461955 1.236142
C -3.074245 -0.901866 -0.045753
C -4.003929 -0.858833 -1.082632
H -5.999287 -0.288622 -1.637503
H -6.584677 0.544108 0.626715
H -4.921107 0.419496 2.465253
H -2.657094 -0.523494 2.029290
H -3.726899 -1.213722 -2.070791
N -1.768779 -1.448269 -0.298732
C -0.648311 -0.741529 -0.246132
O 0.467877 -1.341243 -0.264143
C -1.658360 -2.909947 -0.441622
H -0.894151 -3.147110 -1.180099
H -1.388002 -3.356410 0.518737
H -2.626689 -3.287582 -0.763817
Sc 2.344476 -0.429245 -0.003129
Cl 3.496278 -2.441976 -0.568102
C -0.587083 0.745156 -0.202868
C -1.497445 1.652456 -0.570942
O 0.696124 1.095624 0.185253
C -1.195242 3.121639 -0.623079
H -2.488159 1.319898 -0.854007
C 0.862117 2.472781 0.608766
C 0.300155 3.391165 -0.457111
H -1.771911 3.621681 0.165272
H -1.564928 3.521742 -1.572083
H 1.933804 2.597427 0.757417
H 0.337939 2.576224 1.564782
H 0.482080 4.430098 -0.172589
H 0.834470 3.200752 -1.393679
Cl 3.543548 1.254514 -1.210587
Cl 2.810900 -0.079006 2.296148
35
D-ScCl3-con-1 Eopt -2850.844174
O 0.632975 1.176426 -0.061188
C -0.479863 -0.812467 -0.208105
C -0.576612 0.511506 -0.010739
C 0.618539 2.435515 0.654443
N -1.667649 -1.606401 -0.135746
C -1.836627 1.279934 0.203710
C -0.414473 3.384984 0.046995
H 1.634003 2.822274 0.573902
H 0.406515 2.206124 1.703544
C -2.858633 -1.039498 -0.137360
C -1.498995 -3.062692 -0.000538
C -1.670439 2.650286 -0.471521
H -2.033877 1.423401 1.279228
H -0.685052 4.108432 0.821095
H 0.037826 3.944019 -0.775194
C -2.958313 0.440425 -0.427176
C -4.051252 -1.809601 0.037834
H -0.437956 -3.277726 -0.081244
H -1.871755 -3.384750 0.973055
H -2.040135 -3.566890 -0.801478
H -2.553670 3.272599 -0.312719
H -1.577625 2.479469 -1.550037
C -4.318446 1.019530 -0.206198
H -2.779312 0.477570 -1.525312
C -5.256374 -1.181149 0.110890
H -3.995273 -2.883782 0.152692
C -5.399374 0.254177 0.020240
H -4.409456 2.098171 -0.281951
H -6.146830 -1.781981 0.267029
H -6.381190 0.695227 0.145947
O 0.669202 -1.414644 -0.410675
Sc 2.379866 -0.346988 -0.120642
Cl 2.474923 -0.068491 2.246217
Cl 3.860805 -2.174382 -0.587722
Cl 3.565325 1.500488 -1.129366
35
D-ScCl3-con-2 Eopt -2850.849086
O 0.685592 -1.318938 -0.486790
C -0.467524 -0.736932 -0.277577
C -0.589828 0.582007 -0.027087
N -1.627261 -1.572011 -0.200759
O 0.622167 1.237610 0.096429
C -1.880656 1.257507 0.317351
C -1.413937 -3.026959 -0.120776
C 0.621296 2.629429 -0.329591
C -1.872262 2.722077 -0.137289
H -2.026764 1.237611 1.411072
H -0.347733 -3.208928 -0.208391
H -1.941904 -3.516733 -0.939420
H -1.776534 -3.394917 0.840141
C -0.552384 3.370750 0.275686
H 1.579316 3.024448 0.000581
H 0.582014 2.637819 -1.425164
H -1.978413 2.767146 -1.229804
H -2.711418 3.263176 0.306068
H -0.512833 4.409009 -0.065838
H -0.456270 3.377891 1.367521
C -2.838033 -1.047623 -0.162957
C -2.976247 0.434455 -0.382453
C -4.002380 -1.860631 -0.000601
C -4.354283 0.964803 -0.164760
H -2.765153 0.544626 -1.470360
C -5.226243 -1.270856 0.092706
H -3.912629 -2.935424 0.081963
C -5.413020 0.159906 0.032666
H -4.481632 2.040642 -0.223701
H -6.097858 -1.901804 0.235682
H -6.409966 0.566977 0.153803
Sc 2.394130 -0.298670 -0.061991
Cl 3.797620 -2.113697 -0.764582
Cl 3.720979 1.565034 -0.827685
Cl 2.463920 -0.395592 2.316029
35
D-ScCl3-con-3 Eopt -2850.843834
O 0.627098 1.144245 0.055306
C -0.497838 -0.833959 -0.068402
C -0.589263 0.494119 0.097644
C 0.620903 2.421703 0.734883
N -1.693565 -1.616264 -0.015714
C -1.848111 1.279713 0.246538
C -0.397951 3.367131 0.094867
H 1.641131 2.795346 0.652616
H 0.399569 2.223399 1.788380
C -2.877769 -1.039312 -0.083715
C -1.542713 -3.069610 0.163744
C -1.638562 2.630430 -0.455413
H -2.087713 1.451091 1.309130
H -0.691342 4.093964 0.857398
H 0.075064 3.922938 -0.717794
C -2.949546 0.434310 -0.412396
C -4.084456 -1.795428 0.050826
H -0.479765 -3.290716 0.158434
H -1.981609 -3.367020 1.117198
H -2.031766 -3.590771 -0.659804
H -2.520203 3.266166 -0.349783
H -1.502518 2.430740 -1.524420
C -4.313368 1.029304 -0.272543
H -2.719702 0.442100 -1.501794
C -5.286086 -1.156208 0.046227
H -4.043275 -2.867354 0.190708
C -5.411337 0.277740 -0.084976
H -4.390644 2.106445 -0.378929
H -6.188901 -1.746458 0.168002
H -6.394530 0.728959 -0.021653
O 0.651930 -1.446058 -0.231949
Sc 2.374195 -0.355732 -0.118487
Cl 2.812960 0.136144 2.174682
Cl 3.780635 -2.236371 -0.618680
Cl 3.381547 1.415539 -1.404239
35

D-ScCl3-con-4 Eopt -
2850.843010
O -0.672677 1.141295 -0.504905
C 0.462766 -0.831243 -0.333144
C 0.550340 0.507425 -0.406357
C -0.577385 2.497248 -1.000962
N 1.667912 -1.600891 -0.325969
C 1.804151 1.312169 -0.346236
C 0.282368 3.346917 -0.069870
H -1.603358 2.854509 -1.062212
H -0.157455 2.442124 -2.010202
C 2.833730 -1.023352 -0.111980
C 1.551922 -3.039441 -0.615627
C 1.514335 2.584775 0.469772
H 2.148175 1.592157 -1.355582
H 0.589553 4.233074 -0.632062
H -0.324822 3.691048 0.770603
C 2.851123 0.417719 0.338867
C 4.058699 -1.755996 -0.200359
H 0.494491 -3.272828 -0.693291
H 2.054090 -3.264030 -1.557832
H 1.997435 -3.610661 0.199128
H 2.385889 3.242807 0.476323
H 1.326672 2.275771 1.504353
C 4.212258 1.033375 0.375455
H 2.518713 0.332237 1.398025
C 5.245012 -1.108913 -0.037294
H 4.046395 -2.812625 -0.432230
C 5.334429 0.309649 0.224171
H 4.263389 2.098144 0.578333
H 6.164587 -1.678420 -0.128736
H 6.311909 0.773283 0.286889
O -0.686575 -1.463332 -0.331195
Sc -2.366756 -0.357430 0.010092
Cl -3.788906 -2.289515 0.054158
Cl -3.807245 1.248850 -1.068517
Cl -2.054299 0.269487 2.290743
35
D-ScCl3-con-5 Eopt -
2850.850201
O 0.699385 -1.365061 -0.256187
C -0.466004 -0.778830 -0.131133
C -0.598487 0.548094 0.062983
N -1.626704 -1.611372 -0.077341
O 0.606828 1.203343 0.213910
C -1.895308 1.230246 0.362774
C -1.417184 -3.065174 0.031603
C 0.630379 2.590548 -0.216827
C -1.867613 2.688844 -0.109698
H -2.065878 1.226852 1.453467
H -0.349538 -3.249463 -0.029986
H -1.927834 -3.567243 -0.790392
H -1.800949 -3.417359 0.990275
C -0.560566 3.340739 0.344073
H 1.581204 2.979903 0.147496
H 0.632577 2.595753 -1.312447
H -1.935180 2.721384 -1.205599
H -2.719943 3.237475 0.297892
H -0.508964 4.375804 -0.005056
H -0.503389 3.356779 1.438476
C -2.838303 -1.086080 -0.090564
C -2.972918 0.391889 -0.344779
C -4.006416 -1.897516 0.050240
C -4.356102 0.924184 -0.167925
H -2.738222 0.478596 -1.430256
C -5.232860 -1.307996 0.102617
H -3.918245 -2.970689 0.153154
C -5.418781 0.121473 0.017388
H -4.482230 1.999005 -0.245652
H -6.107182 -1.937903 0.232980
H -6.418377 0.529508 0.109965
Sc 2.403788 -0.290360 -0.006076
Cl 3.790150 -2.228422 -0.266738
Cl 3.041724 0.446642 2.186733
Cl 3.151703 1.082367 -1.817738
35
D-ScCl3-con-6 Eopt -
2850.851384
O -0.679586 -1.428680 -0.055300
C 0.473743 -0.811583 -0.090333
C 0.580904 0.528268 -0.191563
N 1.656918 -1.611808 -0.147424
O -0.635044 1.163490 -0.354838
C 1.872274 1.254645 -0.398118
C 1.489843 -3.059354 -0.360897
C -0.702545 2.521804 0.160704
C 1.792216 2.684439 0.150160
H 2.089834 1.314141 -1.478858
H 0.425082 -3.267162 -0.388122
H 1.952104 -3.603762 0.463117
H 1.949372 -3.341793 -1.309104
C 0.486580 3.330733 -0.315773
H -1.652885 2.908575 -0.208142
H -0.730609 2.454366 1.253921
H 1.818146 2.658766 1.248075
H 2.645915 3.272608 -0.194681
H 0.396536 4.342670 0.088989
H 0.467562 3.408133 -1.408890
C 2.852676 -1.061566 -0.040450
C 2.937099 0.400217 0.309411
C 4.046291 -1.836822 -0.169987
C 4.313304 0.972997 0.235689
H 2.647809 0.414895 1.385161
C 5.258579 -1.218396 -0.122934
H 3.990189 -2.903700 -0.339187
C 5.403944 0.206889 0.057684
H 4.408021 2.043354 0.386005
H 6.153923 -1.820001 -0.244166
H 6.396484 0.641560 0.042720
Sc -2.399019 -0.341782 -0.055828
Cl -3.830765 -2.266302 -0.054262
Cl -3.586137 1.172316 -1.511484
Cl -2.494211 0.422533 2.203808
35
D-ScCl3-con-7 Eopt -
2850.845591
Sc -2.387496 -0.371753 -0.057216
O -0.660387 -1.438998 0.009180
C 0.493641 -0.817823 -0.055893
C 0.580208 0.508304 -0.275728
N 1.684201 -1.605135 -0.022866
O -0.646776 1.093651 -0.499967
C 1.843497 1.277919 -0.456037
C 1.531077 -3.069423 -0.055689
C 2.872975 -1.034402 -0.001569
C -0.698344 2.543099 -0.413753
C 1.741479 2.703694 0.158483
H 2.056420 1.368800 -1.533775
H 0.470057 -3.290247 0.000720
H 1.942980 -3.458875 -0.987833
H 2.047180 -3.504951 0.800109
C 4.076148 -1.803241 -0.076235
C 2.951538 0.453500 0.221957
C 0.311028 3.022175 0.608197
H -1.724992 2.776186 -0.132030
H -0.498380 2.940178 -1.414650
H 2.066538 3.436348 -0.585072
H 2.414480 2.793822 1.017087
C 5.277696 -1.167558 -0.153867
H 4.033064 -2.883452 -0.117105
C 4.307365 1.037072 -0.008059
H 2.748316 0.534527 1.314172
H 0.189053 4.098888 0.750614
H 0.086776 2.533314 1.563167
C 5.401702 0.271819 -0.163086
H 6.178625 -1.767018 -0.238525
H 4.380835 2.120507 -0.007619
H 6.380439 0.715924 -0.301728
Cl -2.489463 0.659208 2.093625
Cl -3.790182 -2.315379 0.022081
Cl -3.608951 1.058568 -1.575233
35
D-ScCl3-con-8 Eopt -
2850.845432
Sc -2.396286 -0.324749 -0.049013
O -0.693159 -1.412737 0.040804
C 0.472826 -0.811005 -0.032914
C 0.580469 0.513821 -0.251763
N 1.650119 -1.617463 -0.018006
O -0.633835 1.118334 -0.482953
C 1.855582 1.266747 -0.413351
C 1.474245 -3.077511 -0.096615
C 2.847523 -1.064390 0.007911
C -0.649257 2.569030 -0.453581
C 1.769815 2.686014 0.218525
H 2.078011 1.368674 -1.488201
H 0.411197 -3.284985 -0.028476
H 1.863500 -3.440791 -1.049107
H 1.998824 -3.549459 0.734076
C 4.039317 -1.846558 -0.099454
C 2.944878 0.417035 0.264127
C 0.328983 3.053840 0.596421
H -1.679456 2.842601 -0.224277
H -0.395042 2.923951 -1.457926
H 2.164048 3.415526 -0.493840
H 2.396845 2.736287 1.114529
C 5.249297 -1.225018 -0.159964
H 3.981037 -2.923924 -0.178535
C 4.310176 0.987392 0.060777
H 2.727386 0.482132 1.354270
H 0.229780 4.136565 0.706575
H 0.050017 2.595604 1.551909
C 5.393770 0.211921 -0.117042
H 6.141540 -1.833240 -0.269988
H 4.399401 2.068978 0.099528
H 6.379283 0.646528 -0.236419
Cl -3.855356 -2.181704 0.363552
Cl -3.421883 0.745202 -1.961828
Cl -2.550113 0.861494 2.034743
35
D-ScCl3-dis-1 Eopt -
2850.840922
O -0.627302 -1.229996 0.414653
C 0.440960 0.778911 0.177106
C 0.565018 -0.532000 0.449090
C -0.457404 -2.624272 0.031697
N 1.611115 1.562939 -0.072481
C 1.833017 -1.297400 0.516859
C 0.529678 -2.709454 -1.124407
H -1.452139 -2.962453 -0.254293
H -0.125367 -3.176173 0.916459
C 2.816481 1.039648 0.051596
C 1.411064 2.930935 -0.578947
C 1.938891 -2.189086 -0.739916
H 1.825209 -1.943927 1.401912
H 0.578335 -3.746255 -1.466330
H 0.116773 -2.116574 -1.947154
C 2.955412 -0.272940 0.775267
C 3.987826 1.729544 -0.389591
H 0.361658 3.177983 -0.452866
H 2.029055 3.619473 -0.003596
H 1.674421 2.974540 -1.637384
H 2.620948 -3.020670 -0.543148

H 2.357269 -1.610867 -1.570697
C 4.316494 -0.878844 0.720348
C 5.202293 1.118418 -0.302577
H 3.907292 2.709104 -0.841853
C 5.378206 -0.210448 0.237447
H 4.418652 -1.879984 1.129915
H 6.075036 1.644260 -0.676848
H 6.367194 -0.653452 0.235683
O -0.731491 1.345706 0.008324
H 2.804554 0.043540 1.829787
Sc -2.422560 0.224900 0.076513
Cl -3.165088 -0.400165 2.270503
Cl -2.937990 -1.184408 -1.818582
Cl -3.814581 2.117176 -0.425253
35
D-ScCl3-dis-2 Eopt -
2850.843610
O 0.788427 -1.206871 -0.596619
C -0.367187 -0.687532 -0.263993
C -0.497842 0.567436 0.206627
N -1.540180 -1.479814 -0.505129
O 0.651755 1.348245 0.131810
C -1.773347 1.243137 0.553325
C -1.366235 -2.737801 -1.249891
C 0.470180 2.412971 -0.854358
C -2.044788 2.325999 -0.502115
H -1.663267 1.741386 1.525479
H -0.305370 -2.965990 -1.268187
H -1.734247 -2.615792 -2.270581
H -1.909519 -3.533803 -0.741952
C -0.793618 3.211810 -0.568511
H 1.367065 3.025662 -0.783873
H 0.430107 1.937605 -1.841999
H -2.235286 1.857208 -1.476468
H -2.919748 2.927769 -0.239707
H -0.894455 3.967316 -1.353137
H -0.672699 3.743424 0.382670
C -2.737580 -1.033013 -0.171892
C -2.829711 0.140425 0.763728
C -3.931432 -1.693589 -0.594308
C -4.203862 0.677994 0.967052
C -5.144673 -1.160972 -0.273978
H -3.876966 -2.580326 -1.211434
C -5.296487 0.048238 0.500277
H -4.285161 1.589264 1.553091
H -6.039168 -1.658412 -0.635845
H -6.291471 0.434968 0.686283
H -2.549465 -0.332419 1.729512
Sc 2.425699 -0.216929 0.095509
Cl 3.898599 -2.095480 -0.142102
Cl 3.642517 1.449633 -1.149488
Cl 2.164724 -0.156815 2.475300
35
D-ScCl3-dis-3 Eopt -
2850.841889
O 0.654949 -1.169293 -0.502346
C -0.479651 0.809874 -0.348313
C -0.565500 -0.519230 -0.530824
C 0.562993 -2.532960 0.006152
N -1.672454 1.564545 -0.108814
C -1.803071 -1.333967 -0.527169
C -0.411335 -2.577000 1.175824
H 1.578637 -2.790376 0.307004
H 0.260541 -3.177310 -0.825273
C -2.859663 0.990352 -0.165728
C -1.508130 2.962674 0.321556
C -1.849864 -2.165926 0.773423
H -1.787600 -2.023295 -1.379515
H -0.399063 -3.585597 1.596642
H -0.025595 -1.900560 1.944421
C -2.969115 -0.365791 -0.811266
C -4.042898 1.658037 0.278056
H -0.474767 3.241880 0.141009
H -2.174292 3.598115 -0.260909
H -1.731724 3.049786 1.386554
H -2.480608 -3.046275 0.622931
H -2.299266 -1.573984 1.578070
C -4.304214 -1.016553 -0.682077
C -5.234354 0.997475 0.265422
H -3.986655 2.662635 0.675803
C -5.375485 -0.363640 -0.199185
H -4.380075 -2.040898 -1.036342
H -6.114411 1.509005 0.642444
H -6.345969 -0.842375 -0.141536
O 0.675538 1.426541 -0.267800
H -2.858792 -0.105125 -1.885686
Sc 2.386870 0.314011 -0.153860
Cl 2.460921 -0.419640 2.117251
Cl 3.833164 2.225848 -0.174706
Cl 3.658878 -1.326191 -1.386481
35
D-ScCl3-dis-4 Eopt -
2850.840236
O 0.652410 1.245770 0.185855
C -0.449129 -0.746772 -0.083665
C -0.543377 0.549165 0.265871
C 0.457934 2.649616 -0.158883
N -1.643359 -1.510324 -0.288893
C -1.799718 1.316476 0.448680
C -0.605974 2.760511 -1.241993
H 1.433208 2.993224 -0.497471
H 0.183663 3.182008 0.757192
C -2.832065 -0.989714 -0.048604
C -1.492500 -2.853263 -0.873258
C -1.989675 2.250937 -0.766441
H -1.727559 1.929662 1.354393
H -0.665147 3.802994 -1.564401
H -0.263380 2.176919 -2.102926
C -2.908140 0.288037 0.742660
C -4.038161 -1.655672 -0.429262
H -0.436870 -3.104698 -0.850046
H -2.059842 -3.568973 -0.278861
H -1.847268 -2.848247 -1.905584
H -2.639018 3.086353 -0.490756
H -2.482082 1.707598 -1.580093
C -4.264422 0.903281 0.812372
C -5.238213 -1.043530 -0.226598
H -3.998973 -2.616677 -0.924577
C -5.363924 0.262360 0.379484
H -4.327798 1.885918 1.271496
H -6.140172 -1.550133 -0.555468
H -6.346715 0.710251 0.468034
O 0.699861 -1.312762 -0.357537
H -2.685121 -0.077378 1.768325
Sc 2.408583 -0.258169 0.008132
Cl 3.828726 -2.044581 -0.732878
Cl 3.696401 1.644448 -0.744338
Cl 2.505011 -0.383848 2.385069
35
D-ScCl3-dis-5 Eopt -
2850.843646
O 0.735343 -1.252558 -0.584665
C -0.401201 -0.700460 -0.250040
C -0.497676 0.576419 0.166709
N -1.594205 -1.481997 -0.419838
O 0.676067 1.317592 0.045892
C -1.753305 1.312225 0.457559
C -1.453919 -2.808245 -1.042955
C 0.529533 2.310999 -1.020414
C -1.988194 2.319711 -0.679257
H -1.636882 1.878223 1.391100
H -0.395125 -3.044223 -1.078147
H -1.860253 -2.786046 -2.055840
H -1.980384 -3.546298 -0.438101
C -0.712932 3.163462 -0.806149
H 1.444103 2.900747 -0.989551
H 0.475909 1.760994 -1.968356
H -2.186950 1.782050 -1.615792
H -2.847069 2.964377 -0.470845
H -0.788977 3.856429 -1.649222
H -0.581562 3.765395 0.100521
C -2.778118 -0.986741 -0.107321
C -2.841593 0.258204 0.733926
C -3.988984 -1.663728 -0.448443
C -4.201624 0.845370 0.889449
C -5.189085 -1.082607 -0.164629
H -3.957999 -2.606487 -0.977923
C -5.310761 0.198061 0.490417
H -4.259213 1.809065 1.387812
H -6.096307 -1.597829 -0.464783
H -6.296117 0.621678 0.644457
H -2.583615 -0.154326 1.733755
Sc 2.412685 -0.266324 0.068916
Cl 3.870338 -2.035196 -0.636282
Cl 2.098502 -0.464814 2.417804
Cl 3.812299 1.640405 -0.432133
35
D-ScCl3-dis-6 Eopt -
2850.841910
O -0.687107 -1.360679 0.071662
C 0.468563 -0.772151 -0.095229
C 0.584049 0.545265 -0.340363
N 1.651234 -1.565689 0.086490
O -0.602647 1.260676 -0.228415
C 1.843248 1.302636 -0.548885
C 1.474129 -2.936417 0.592511
C -0.509904 2.281089 0.815386
C 2.010806 2.304185 0.603362
H 1.760004 1.886588 -1.475378
H 0.425446 -3.193985 0.482282
H 1.754506 -2.981096 1.646853
H 2.089377 -3.617363 0.005428
C 0.728592 3.146381 0.642481
H -1.433460 2.855378 0.730218
H -0.492873 1.758697 1.778246
H 2.148973 1.768570 1.551414
H 2.880416 2.948809 0.447547
H 0.754211 3.856004 1.474488
H 0.643535 3.728399 -0.282535
C 2.849474 -1.035988 -0.076718
C 2.961890 0.270750 -0.813282
C 4.036313 -1.720495 0.326711
C 4.323253 0.874985 -0.820584
C 5.246710 -1.108578 0.189788
H 3.973608 -2.694534 0.793280
C 5.403947 0.211841 -0.372350
H 4.410246 1.868941 -1.250361
H 6.132255 -1.630361 0.539146
H 6.392796 0.652685 -0.416555
H 2.785154 -0.066957 -1.856682
Sc -2.409962 -0.280007 -0.128482
Cl -3.735445 -2.222967 -0.588131
Cl -3.467658 1.323535 -1.592428
Cl -2.995839 0.329973 2.095992
133
F-Feng-1 Eopt -
3432.766388
O -2.446403 -0.298001 -0.094323
O -0.890531 -2.110466 1.201767
O -0.651958 -2.148778 -1.764495
O 1.559459 -1.570520 -0.304606
N -4.426735 0.128123 0.861023
H -5.144198 -0.174455 1.513376

N -2.114589 -2.738356 1.329061
N 3.512517 -2.217962 -1.179071
H 4.013020 -2.782429 -1.858532
N 0.044897 -3.332931 -1.907162
C -4.674297 1.302273 0.055596
C -3.992640 2.491363 0.367462
C -4.245395 3.594990 -0.451417
H -3.738554 4.534017 -0.262151
C -5.144326 3.512111 -1.512702
H -5.326441 4.385414 -2.130933
C -5.808855 2.321945 -1.785743
H -6.505734 2.277003 -2.615510
C -5.580070 1.183033 -1.007412
C -6.229187 -0.150817 -1.338302
H -6.388041 -0.695568 -0.400816
C -3.056013 2.580552 1.565319
C -3.320740 -0.590110 0.751356
C -3.176004 -1.740812 1.731605
H -4.117177 -2.298774 1.768989
C -2.735946 -1.322675 3.141189
H -3.593963 -0.994043 3.728037
H -2.025134 -0.495771 3.073964
C -2.063813 -2.592838 3.718160
H -2.630526 -3.004887 4.553090
C -2.023455 -3.600330 2.564494
H -2.884620 -4.272148 2.560309
C -0.097372 -4.224327 -0.701051
H 0.450220 -5.138789 -0.941526
H 0.399991 -3.713267 0.122762
C -0.433036 -3.957124 -3.196308
H -0.081656 -4.991180 -3.207185
C 0.217184 -3.096384 -4.286437
H 0.527835 -3.726617 -5.119865
C 1.424782 -2.400420 -3.611248
H 1.248937 -1.328318 -3.506328
H 2.361124 -2.548849 -4.150711
C 1.497844 -3.057682 -2.226739
H 1.982549 -4.037443 -2.289480
C 2.189131 -2.232598 -1.159150
C 4.239888 -1.386680 -0.250470
C 4.336483 -1.798830 1.085022
C 4.948224 -0.913396 1.979945
H 5.030730 -1.184720 3.028656
C 5.462990 0.302611 1.542955
C 5.391244 0.657765 0.196545
H 5.824081 1.596037 -0.132314
C 4.761060 -0.173563 -0.730788
C 4.614445 0.206436 -2.197033
C 3.837529 -3.152446 1.567053
H 3.523825 -3.734909 0.694590
H 4.729989 -0.711528 -2.786507
H -2.382908 1.710329 1.534739
H -0.486185 -2.353318 -4.660567
H -1.520867 -3.925402 -3.185833
H -1.097022 -4.167541 2.492823
H -1.056380 -2.367823 4.069883
C -2.169499 3.826486 1.549805
H -1.570444 3.890429 0.635450
H -2.765904 4.739330 1.648463
H -1.477119 3.786164 2.394635
C -3.850466 2.542766 2.881259
H -4.488600 3.429494 2.955274
H -4.491009 1.661093 2.963557
H -3.165227 2.543647 3.734978
C -5.279509 -0.985850 -2.212571
H -5.125310 -0.488539 -3.176208
H -4.298251 -1.109827 -1.744332
H -5.703708 -1.976887 -2.400159
C -7.593415 -0.011737 -2.017078
H -8.062864 -0.995924 -2.097600
H -8.260919 0.641951 -1.448440
H -7.499492 0.388653 -3.031210
C 3.216524 0.779392 -2.487484
H 3.045361 0.829191 -3.567756
H 2.411641 0.193394 -2.034847
H 3.133088 1.792815 -2.083117
C 5.684148 1.190811 -2.673882
H 5.552721 2.173733 -2.208195
H 6.693697 0.833079 -2.451755
H 5.597630 1.328047 -3.755287
C 2.626291 -3.021160 2.500221
H 2.876604 -2.422501 3.383495
H 1.782291 -2.548194 1.990642
H 2.309694 -4.011684 2.842851
C 4.964858 -3.937277 2.251785
H 5.839093 -4.024780 1.600644
H 5.278452 -3.450442 3.180518
H 4.618739 -4.943995 2.503435
C -2.508320 -3.494295 0.086937
H -2.638716 -2.752351 -0.699496
H -3.475399 -3.951504 0.309640
C -1.533460 -4.589306 -0.337490
H -2.011681 -5.104600 -1.176697
H -1.455249 -5.342100 0.453628
Sc -0.386429 -0.735525 -0.295864
H 5.938312 0.974041 2.251432
O 0.086557 1.042119 -1.078917
C 0.604188 2.017212 -0.343825
C 0.682900 1.834310 1.059928
N 0.890937 3.225665 -0.978209
O 0.334565 0.503492 1.393257
C 1.007229 2.729461 2.040966
C -0.092113 3.714807 -1.941637
C 2.049643 3.976899 -0.749508
C 0.805662 0.056261 2.683118
C 1.038916 2.390474 3.502454
H 1.256908 3.737077 1.731649
H -0.823890 2.927188 -2.112277
H 0.390398 3.962036 -2.892223
H -0.609453 4.605760 -1.565416
C 3.173947 3.429477 -0.097277
C 2.127307 5.307853 -1.198359
C 0.374417 1.038987 3.754798
H 0.372056 -0.936050 2.813519
H 1.899482 -0.025499 2.626226
H 2.077893 2.364376 3.862603
H 0.537854 3.174746 4.081517
C 4.315207 4.196272 0.104342
H 3.148808 2.402688 0.251105
C 3.283246 6.060874 -0.994321
H 1.286478 5.766825 -1.703958
H 0.635113 0.638934 4.738584
H -0.716844 1.147878 3.717465
C 4.386303 5.518968 -0.339295
H 5.163129 3.751244 0.617920
H 3.310751 7.085514 -1.352467
H 5.281374 6.109632 -0.177270
133
F-Feng-2 Eopt -3432.764945
O -2.522477 -0.227600 -0.103481
O -1.132883 -2.069967 1.316469
O -0.803178 -2.100769 -1.669959
O 1.454827 -1.628109 -0.253786
N -4.497455 0.353123 0.777441
H -5.261193 0.111209 1.402165
N -2.386690 -2.653190 1.361853
N 3.357062 -2.315955 -1.210787
H 3.804470 -2.884774 -1.922786
N -0.163902 -3.315196 -1.831104
C -4.616632 1.544668 -0.031966
C -3.850873 2.672547 0.309920
C -3.980251 3.794981 -0.511800
H -3.404180 4.688030 -0.298966
C -4.842879 3.788645 -1.606074
H -4.929233 4.675170 -2.226217
C -5.591965 2.657464 -1.909952
H -6.258114 2.671263 -2.765658
C -5.487412 1.501980 -1.129404
C -6.228802 0.225516 -1.491610
H -6.475924 -0.301295 -0.562970
C -2.952557 2.679178 1.539453
C -3.449595 -0.452339 0.705720
C -3.445179 -1.621574 1.675844
H -4.402493 -2.146812 1.595870
C -3.167348 -1.241085 3.139382
H -4.101249 -0.982647 3.639552
H -2.504944 -0.375562 3.189156
C -2.496406 -2.497536 3.745339
H -3.074089 -2.908563 4.573077
C -2.417094 -3.510861 2.603613
H -3.305061 -4.143858 2.540842
C -0.322848 -4.199022 -0.622247
H 0.207619 -5.125134 -0.856206
H 0.183582 -3.694807 0.200331
C -0.698430 -3.916240 -3.108777
H -0.394519 -4.965277 -3.126771
C -0.035150 -3.085649 -4.214163
H 0.231346 -3.729919 -5.052114
C 1.213911 -2.440645 -3.565470
H 1.085174 -1.362235 -3.455559
H 2.130824 -2.626899 -4.126026
C 1.290302 -3.100904 -2.182287
H 1.735309 -4.098986 -2.252999
C 2.036105 -2.297311 -1.135537
C 4.176109 -1.500344 -0.348757
C 4.269749 -1.824237 1.010852
C 5.048331 -0.981279 1.812886
H 5.137187 -1.188135 2.875732
C 5.724219 0.103691 1.266162
C 5.627708 0.382185 -0.096580
H 6.167314 1.227284 -0.508869
C 4.828619 -0.399624 -0.930932
C 4.612301 -0.062939 -2.398351
C 3.587298 -3.036057 1.623024
H 3.088843 -3.595131 0.825248
H 4.602083 -1.004477 -2.961521
H -2.359718 1.752243 1.525550
H -0.714140 -2.314224 -4.575670
H -1.783235 -3.835587 -3.074386
H -1.513750 -4.117985 2.600698
H -1.498105 -2.258346 4.112561
C -1.957656 3.840359 1.559924
H -1.333551 3.859760 0.660500
H -2.472707 4.801850 1.654792
H -1.291599 3.729700 2.419699
C -3.789678 2.706006 2.828646
H -4.349747 3.645007 2.887839
H -4.507450 1.883588 2.883194
H -3.134936 2.643091 3.703707
C -5.312115 -0.687896 -2.321850
H -5.072651 -0.207508 -3.276646
H -4.367129 -0.892636 -1.809488
H -5.808543 -1.640400 -2.530441
C -7.543634 0.474755 -2.233358
H -8.087665 -0.467911 -2.337988
H -8.181559 1.181796 -1.695429
H -7.369412 0.864396 -3.241072
C 3.249108 0.625187 -2.596206
H 2.999143 0.673896 -3.661039
H 2.433534 0.117669 -2.070473
H 3.287833 1.647191 -2.206180
C 5.720683 0.806784 -2.993035

H 5.713862 1.810505 -2.554260
H 6.709947 0.366520 -2.837985
H 5.559073 0.919006 -4.068603
C 2.517326 -2.618815 2.639976
H 2.971421 -2.119188 3.502787
H 1.796667 -1.935717 2.181568
H 1.978666 -3.499080 3.005830
C 4.612087 -3.981453 2.263552
H 5.371685 -4.290452 1.539941
H 5.119338 -3.500760 3.105924
H 4.110071 -4.876949 2.641726
C -2.731744 -3.409137 0.103319
H -2.805806 -2.670621 -0.693485
H -3.719488 -3.840698 0.281534
C -1.767133 -4.532532 -0.263305
H -2.240099 -5.070678 -1.090978
H -1.711308 -5.258175 0.554331
Sc -0.468921 -0.743262 -0.159937
H 6.335489 0.735508 1.903397
O 0.094028 1.001620 -0.942500
C 0.719234 1.926228 -0.227635
C 0.815225 1.749775 1.174561
N 1.125392 3.087044 -0.886046
O 0.388011 0.444399 1.531086
C 1.246259 2.629764 2.128102
C 0.187939 3.658139 -1.849588
C 2.349115 3.730235 -0.666909
C 0.187956 0.244441 2.947982
C 1.467788 2.259389 3.564952
H 1.463659 3.640107 1.802284
H -0.608942 2.936433 -2.020609
H -0.251157 4.591073 -1.475349
H 0.691646 3.861431 -2.799499
C 2.540688 5.051176 -1.110048
C 3.425542 3.078785 -0.030614
C 1.391953 0.742804 3.720636
H 0.004316 -0.824069 3.062710
H -0.707479 0.810608 3.239884
H 0.714687 2.735625 4.210518
H 2.440608 2.634776 3.902492
C 3.759939 5.698379 -0.910440
H 1.739673 5.585712 -1.606178
C 4.630279 3.741704 0.169085
H 3.315343 2.051638 0.301135
H 1.298025 0.445754 4.768751
H 2.293185 0.269755 3.312545
C 4.813892 5.057070 -0.264692
H 3.875788 6.718582 -1.263574
H 5.439524 3.218812 0.670822
H 5.758426 5.565507 -0.104194
35
F-ScCl3-1 Eopt -
2850.993326
O -0.821547 1.230094 -0.048156
C 0.624078 -0.513641 -0.440974
C 0.449010 0.886462 -0.533802
C -1.262969 2.573078 -0.333418
N 1.834724 -1.147527 -0.723360
C 1.329154 1.838888 -0.967349
C -0.167974 3.564028 0.010304
H -2.161147 2.708491 0.271443
H -1.527887 2.618556 -1.396664
C 3.029245 -0.820353 -0.082103
C 1.810172 -2.227571 -1.702676
C 1.042373 3.311026 -0.889211
H 2.276994 1.492720 -1.364707
H -0.552464 4.579907 -0.115862
H 0.104186 3.435849 1.064133
C 3.038633 -0.058163 1.103887
C 4.259901 -1.266106 -0.597245
H 0.769791 -2.485263 -1.897243
H 2.285500 -1.927721 -2.644622
H 2.323315 -3.112322 -1.313198
H 0.847926 3.721909 -1.890666
H 1.919858 3.845114 -0.506989
C 4.237074 0.244579 1.737647
H 2.100380 0.285659 1.525577
C 5.454078 -0.955816 0.053397
H 4.291061 -1.850402 -1.509585
C 5.458849 -0.197779 1.221925
H 4.213494 0.829243 2.652700
H 6.389053 -1.313737 -0.367716
H 6.390365 0.042202 1.723367
O -0.426543 -1.267364 -0.150837
Sc -2.234050 -0.523788 0.220729
Cl -3.274609 0.181838 -1.841814
Cl -2.924672 0.621495 2.252048
Cl -3.296217 -2.674604 0.549054
35
F-ScCl3-2 Eopt -
2850.992813
O 0.402723 -1.245050 0.179508
C -0.650193 -0.466381 0.379185
C -0.463030 0.937012 0.359812
N -1.855231 -1.068934 0.744169
O 0.841197 1.223021 -0.065972
C -1.336096 1.932702 0.700935
C -1.825269 -1.989614 1.874504
C 1.122962 2.594865 -0.407593
C -0.948551 3.383806 0.715914
H -2.338277 1.638810 0.991793
H -0.785642 -2.247014 2.073479
H -2.373843 -2.906385 1.638047
H -2.260068 -1.537700 2.774918
C 0.572348 3.523752 0.656652
H 2.207903 2.653160 -0.496887
H 0.671322 2.792198 -1.387373
H -1.399545 3.915582 -0.134892
H -1.341037 3.866013 1.617964
H 0.869677 4.549679 0.421952
H 1.019494 3.255223 1.620570
C -3.062578 -0.789536 0.106764
C -3.091496 -0.175623 -1.162157
C -4.286477 -1.136983 0.706874
C -4.301378 0.080700 -1.793997
H -2.158270 0.090543 -1.646954
C -5.492773 -0.875526 0.056830
H -4.303026 -1.604768 1.684368
C -5.516512 -0.263697 -1.194169
H -4.292965 0.550246 -2.773359
H -6.422227 -1.154429 0.544675
H -6.457426 -0.061340 -1.694590
Sc 2.234354 -0.547078 -0.177458
Cl 2.987694 0.417901 -2.277103
Cl 3.254879 0.255703 1.859384
Cl 3.236957 -2.743325 -0.383569
35
F-ScCl3-3 Eopt -
2850.993137
O 0.802372 1.220912 -0.016226
C -0.640388 -0.538925 0.335328
C -0.466418 0.858499 0.466493
C 1.241405 2.549535 0.339856
N -1.846243 -1.186166 0.608749
C -1.344745 1.794597 0.938660
C 0.151301 3.557032 0.030878
H 2.147899 2.712036 -0.244166
H 1.489451 2.542429 1.408259
C -3.057537 -0.821984 0.021182
C -1.804382 -2.300896 1.548108
C -1.063917 3.269456 0.912664
H -2.287649 1.432715 1.333627
H 0.537703 4.565724 0.201498
H -0.114858 3.474012 -1.028956
C -3.098141 -0.011389 -1.131686
C -4.275385 -1.277294 0.558170
H -0.761689 -2.576272 1.700716
H -2.247527 -2.030166 2.514324
H -2.339565 -3.164949 1.142595
H -0.877086 3.645055 1.929321
H -1.941329 3.813707 0.544535
C -4.313667 0.330216 -1.710614
H -2.170648 0.341008 -1.569556
C -5.487150 -0.928156 -0.037845
H -4.282904 -1.898790 1.446029
C -5.522549 -0.120474 -1.172091
H -4.314456 0.953166 -2.600381
H -6.411365 -1.294107 0.399778
H -6.467627 0.150681 -1.630424
O 0.412067 -1.283272 0.032060
Sc 2.237784 -0.533011 -0.236177
Cl 3.289052 -2.681351 -0.620120
Cl 3.093301 0.076331 1.924430
Cl 3.285047 0.791357 -1.984899
35
F-ScCl3-4 Eopt -
2850.992596
O -0.911961 1.267728 -0.210188
C 0.590122 -0.377322 -0.770833
C 0.387703 1.017469 -0.668242
C -1.350107 2.641041 -0.229250
N 1.833632 -0.931688 -1.081747
C 1.273515 2.031946 -0.905018
C -0.263237 3.546903 0.315717
H -2.256879 2.661222 0.375830
H -1.604180 2.896152 -1.265134
C 2.959648 -0.739398 -0.281694
C 1.897994 -1.837710 -2.221647
C 0.965560 3.467503 -0.590148
H 2.245594 1.756716 -1.299201
H -0.649375 4.568134 0.377103
H -0.010361 3.220449 1.331182
C 2.853064 -0.190011 1.012440
C 4.235109 -1.108823 -0.745695
H 0.879627 -2.042718 -2.550427
H 2.458126 -1.396631 -3.055045
H 2.371188 -2.783781 -1.939354
H 0.781640 4.041667 -1.509944
H 1.828411 3.937608 -0.104887
C 3.984090 -0.018524 1.800500
H 1.877673 0.089425 1.396752
C 5.360117 -0.932055 0.059726
H 4.354951 -1.529848 -1.737328
C 5.250573 -0.385000 1.336045
H 3.870657 0.400556 2.796088
H 6.332075 -1.227190 -0.324762
H 6.128600 -0.249430 1.958489
O -0.447129 -1.188601 -0.619103
Sc -2.177985 -0.576561 0.152347
Cl -1.959647 -0.091295 2.508858
Cl -3.166903 -2.785678 0.229127
Cl -3.914489 0.580392 -1.097602
35
F-ScCl3-5 Eopt -
2850.993197
O -0.405504 -1.214257 -0.462531
C 0.640634 -0.409789 -0.580240
C 0.428511 0.985854 -0.481116
N 1.867019 -0.971944 -0.936770
O -0.895733 1.227112 -0.088962
C 1.293476 2.016887 -0.723118
C 1.879549 -1.884218 -2.074349
C -1.172604 2.543897 0.426306

C 0.891418 3.460695 -0.617606
H 2.309396 1.760456 -1.002013
H 0.847604 -2.119451 -2.331367
H 2.396860 -2.813844 -1.817179
H 2.370870 -1.434077 -2.945530
C -0.630078 3.590435 -0.528311
H -2.257658 2.591991 0.527270
H -0.708301 2.618191 1.417329
H 1.351876 3.922238 0.268392
H 1.269964 4.019665 -1.480753
H -0.923877 4.584017 -0.178576
H -1.087556 3.431528 -1.511410
C 3.043728 -0.733115 -0.227771
C 3.019606 -0.161797 1.060966
C 4.290594 -1.081005 -0.778896
C 4.201546 0.055106 1.757853
H 2.068451 0.101922 1.510831
C 5.467443 -0.859512 -0.064026
H 4.347572 -1.519358 -1.768427
C 5.439180 -0.288067 1.206020
H 4.152216 0.492438 2.750816
H 6.415228 -1.138253 -0.515297
H 6.357553 -0.116680 1.757310
Sc -2.203864 -0.597870 0.125843
Cl -3.859428 0.692378 -1.107647
Cl -3.210441 -2.797473 -0.016891
Cl -2.226973 -0.161786 2.500477
35
F-ScCl3-6 Eopt -
2850.993240
C -5.492900 -0.823534 -0.038345
C -5.453837 -0.264752 1.236963
C -4.210078 0.054192 1.790054
C -3.032911 -0.175447 1.089552
C -3.067738 -0.735967 -0.203753
C -4.320618 -1.057762 -0.756953
H -6.445744 -1.082002 -0.491054
H -6.368582 -0.083925 1.791248
H -4.152611 0.481977 2.786731
H -2.076546 0.069481 1.539336
H -4.385761 -1.484923 -1.750921
N -1.894934 -0.989162 -0.912670
C -0.663869 -0.430594 -0.563039
O 0.376070 -1.240248 -0.430883
C -1.921618 -1.876562 -2.069068
H -0.893570 -2.130648 -2.324562
H -2.461681 -2.799015 -1.834336
H -2.396783 -1.397686 -2.934287
Sc 2.196717 -0.613671 0.097288
Cl 3.203840 -2.812530 0.008143
C -0.444585 0.965264 -0.485446
C -1.308563 1.997672 -0.726564
O 0.885660 1.206439 -0.114672
C -0.900117 3.440929 -0.638149
H -2.330086 1.743305 -0.986127
C 1.172550 2.525861 0.389078
C 0.622669 3.567140 -0.566535
H -1.348977 3.910486 0.249639
H -1.287029 3.994740 -1.500902
H 2.258678 2.568610 0.478636
H 0.719233 2.607489 1.384559
H 0.922683 4.562250 -0.226500
H 1.069129 3.400718 -1.553459
Cl 3.881935 0.683927 -1.073840
Cl 2.346400 -0.071269 2.438364
35
G-ScCl3-con-1 Eopt -
2850.980373
O 0.628477 1.146077 0.004819
C -0.502278 -0.853381 -0.174361
C -0.596790 0.458796 0.098161
C 0.617742 2.365989 0.771076
N -1.659105 -1.654497 -0.258124
C -1.861682 1.251835 0.178811
C -0.417407 3.343295 0.200846
H 1.633323 2.760071 0.718615
H 0.395003 2.094092 1.808972
C -2.894221 -1.040511 -0.163081
C -1.526000 -3.073633 0.030303
C -1.593915 2.612798 -0.476370
H -2.188304 1.409678 1.219068
H -0.779029 3.965180 1.025784
H 0.057568 4.011230 -0.522827
C -2.940477 0.423456 -0.567527
C -4.034877 -1.726290 0.209521
H -0.499404 -3.368069 -0.175105
H -1.763675 -3.293045 1.079662
H -2.200867 -3.643192 -0.612850
H -2.486462 3.243586 -0.447699
H -1.353568 2.432937 -1.531338
C -4.315452 1.012854 -0.441103
H -2.624418 0.464072 -1.629573
C -5.290634 -1.090145 0.267398
H -3.969630 -2.774608 0.478325
C -5.401822 0.289647 -0.052681
H -4.423816 2.063662 -0.694240
H -6.163627 -1.652824 0.577421
H -6.371844 0.774570 0.016012
O 0.676700 -1.423721 -0.391035
Sc 2.340715 -0.328026 -0.124572
Cl 2.586172 -0.071536 2.245047
Cl 3.861611 -2.136185 -0.651734
Cl 3.511215 1.522755 -1.186465
35
G-ScCl3-con-2 Eopt -
2850.984273
O 0.723045 -1.241473 -0.651698
C -0.466136 -0.711684 -0.409127
C -0.596319 0.577407 -0.034795
N -1.584064 -1.565888 -0.497726
O 0.628141 1.249830 0.144946
C -1.894551 1.250384 0.274991
C -1.362949 -3.001871 -0.420611
C 0.626076 2.618064 -0.327762
C -1.868998 2.716490 -0.177287
H -2.096807 1.229359 1.359224
H -0.346723 -3.212873 -0.742734
H -2.067821 -3.514003 -1.079681
H -1.501073 -3.371357 0.603983
C -0.557094 3.374108 0.245834
H 1.578844 3.037406 -0.009986
H 0.587711 2.595735 -1.424811
H -1.954140 2.755026 -1.272831
H -2.721278 3.259158 0.241535
H -0.508212 4.408590 -0.108593
H -0.475368 3.395262 1.339354
C -2.840003 -1.049551 -0.239627
C -2.990279 0.439992 -0.466911
C -3.910114 -1.842753 0.126628
C -4.377344 0.932016 -0.171073
H -2.766858 0.614065 -1.539390
C -5.189146 -1.293101 0.348664
H -3.770119 -2.909067 0.263449
C -5.393451 0.104569 0.201045
H -4.559654 1.995355 -0.296275
H -6.006302 -1.938099 0.650269
H -6.379913 0.519860 0.389235
Sc 2.346389 -0.268316 0.002318
Cl 3.818024 -2.061311 -0.700293
Cl 3.732863 1.572163 -0.777121
Cl 2.385251 -0.512019 2.384287
35
G-ScCl3-con-3 Eopt -
2850.980380
O 0.624282 1.151809 0.041936
C -0.497320 -0.850546 -0.152882
C -0.598356 0.459248 0.128840
C 0.610499 2.368275 0.812504
N -1.649848 -1.655891 -0.247742
C -1.867241 1.247047 0.201532
C -0.431672 3.343902 0.250443
H 1.624144 2.767563 0.757458
H 0.394155 2.091951 1.850565
C -2.888104 -1.046895 -0.163523
C -1.513546 -3.075126 0.038844
C -1.597114 2.612244 -0.443737
H -2.205465 1.398032 1.239016
H -0.804605 3.950278 1.081831
H 0.040179 4.026875 -0.461076
C -2.934716 0.418714 -0.561565
C -4.030290 -1.738635 0.192849
H -0.483142 -3.364212 -0.154878
H -1.762802 -3.297843 1.084798
H -2.177831 -3.646586 -0.613575
H -2.492152 3.239766 -0.421892
H -1.344020 2.438875 -1.496823
C -4.313375 1.002266 -0.449809
H -2.605729 0.465463 -1.619432
C -5.289131 -1.107527 0.237755
H -3.964199 -2.787924 0.457625
C -5.401714 0.273067 -0.078245
H -4.422919 2.053527 -0.700509
H -6.163749 -1.674971 0.534204
H -6.374546 0.753731 -0.020394
O 0.684773 -1.414555 -0.370084
Sc 2.342913 -0.310984 -0.118244
Cl 2.639909 -0.039436 2.247460
Cl 3.857925 -2.134478 -0.612862
Cl 3.451018 1.489980 -1.325919
35
G-ScCl3-con-4 Eopt -
2850.979038
O -0.640059 1.161950 -0.348420
C 0.477299 -0.854577 -0.242460
C 0.590964 0.478520 -0.364688
C -0.542406 2.457355 -0.969898
N 1.624183 -1.666855 -0.134656
C 1.856157 1.266612 -0.250000
C 0.417002 3.357870 -0.182891
H -1.556193 2.853414 -1.000385
H -0.194556 2.296607 -1.996728
C 2.861780 -1.057595 -0.053962
C 1.518317 -3.058110 -0.543461
C 1.527186 2.552025 0.519719
H 2.272626 1.531409 -1.235280
H 0.851963 4.073917 -0.887375
H -0.138367 3.934586 0.561742
C 2.868865 0.358840 0.495974
C 4.032953 -1.716094 -0.378569
H 0.477744 -3.361491 -0.454155
H 1.849067 -3.197468 -1.581095
H 2.134211 -3.679150 0.111401
H 2.417170 3.175109 0.642937
H 1.188519 2.260691 1.521629
C 4.248718 0.949229 0.545752
H 2.463723 0.293441 1.526252
C 5.288013 -1.087739 -0.259243
H 3.992998 -2.734514 -0.748665
C 5.366949 0.256738 0.193851
H 4.332483 1.972296 0.901194
H 6.186723 -1.626311 -0.536860
H 6.338367 0.739639 0.257161
O -0.712720 -1.441793 -0.205512

Sc -2.337658 -0.292573 0.041358
Cl -3.830950 -2.202212 0.090748
Cl -3.635426 1.107555 -1.468841
Cl -2.365593 0.394665 2.340622
35
G-ScCl3-con-5 Eopt -2850.985959
O 0.674525 -1.425049 -0.044490
C -0.505773 -0.828268 -0.008107
C -0.606167 0.505286 0.158774
N -1.652385 -1.642493 -0.080958
O 0.633052 1.141630 0.361585
C -1.891891 1.243087 0.338968
C -1.508399 -3.073675 0.131318
C 0.710618 2.481021 -0.176961
C -1.779954 2.675636 -0.196574
H -2.158270 1.301094 1.408790
H -0.470204 -3.345360 -0.039440
H -2.147219 -3.614325 -0.571646
H -1.791164 -3.353632 1.154141
C -0.473948 3.309732 0.284533
H 1.664251 2.875928 0.178458
H 0.731145 2.403641 -1.271059
H -1.786965 2.652119 -1.295827
H -2.636408 3.272689 0.129436
H -0.363254 4.322161 -0.116010
H -0.465234 3.383456 1.378832
C -2.902858 -1.057676 -0.023713
C -2.966645 0.409158 -0.404528
C -4.041899 -1.779781 0.279224
C -4.349376 0.978418 -0.266908
H -2.658050 0.468024 -1.468980
C -5.311707 -1.169727 0.316596
H -3.964362 -2.835285 0.514304
C -5.434596 0.220960 0.055937
H -4.468619 2.036855 -0.479106
H -6.184107 -1.759569 0.572894
H -6.413680 0.688511 0.118432
Sc 2.355075 -0.338790 0.084676
Cl 3.791283 -2.290063 0.096245
Cl 3.553153 1.115950 1.619922
Cl 2.722458 0.521057 -2.121627
35
G-ScCl3-con-6 Eopt -2850.985979
O -0.670657 -1.428157 0.059844
C 0.509122 -0.829764 0.015690
C 0.607524 0.503042 -0.157365
N 1.656834 -1.642784 0.087665
O -0.631945 1.138444 -0.359054
C 1.892838 1.241897 -0.338391
C 1.513451 -3.073844 -0.127100
C -0.710324 2.478555 0.178437
C 1.779975 2.674907 0.196100
H 2.158852 1.299262 -1.408261
H 0.476252 -3.346618 0.048034
H 2.155822 -3.614994 0.572067
H 1.792000 -3.351120 -1.151826
C 0.473184 3.307513 -0.284474
H -1.664403 2.872262 -0.176807
H -0.730621 2.401567 1.272468
H 1.787568 2.652246 1.295359
H 2.635898 3.272105 -0.130891
H 0.361404 4.320083 0.115578
H 0.463752 3.380739 -1.378755
C 2.906348 -1.056656 0.027090
C 2.968473 0.410355 0.406458
C 4.045722 -1.776611 -0.280061
C 4.350406 0.981310 0.269326
H 2.658971 0.470249 1.470653
C 5.314247 -1.164319 -0.320195
H 3.969545 -2.832002 -0.515949
C 5.436085 0.225931 -0.056946
H 4.468659 2.039307 0.484272
H 6.186869 -1.752580 -0.579551
H 6.414554 0.694657 -0.119562
Sc -2.351803 -0.345365 -0.088688
Cl -3.787300 -2.295763 -0.090242
Cl -3.554902 1.113218 -1.620559
Cl -2.742891 0.537298 2.103402
35
G-ScCl3-dis-1 Eopt -2850.976561
O -0.632953 -1.200725 0.418317
C 0.464824 0.832775 0.324776
C 0.580449 -0.492222 0.506213
C -0.448193 -2.549062 -0.082632
N 1.606132 1.662368 0.232476
C 1.839366 -1.269119 0.595916
C 0.596100 -2.549687 -1.192464
H -1.427421 -2.864897 -0.443166
H -0.154800 -3.183032 0.761148
C 2.842940 1.044443 0.151942
C 1.427675 2.998670 -0.312033
C 1.993098 -2.112180 -0.686058
H 1.792940 -1.951849 1.456498
H 0.632265 -3.548790 -1.636203
H 0.240903 -1.864509 -1.969734
C 2.971837 -0.257693 0.917844
C 3.925142 1.615129 -0.489168
H 0.434856 3.351833 -0.044248
H 2.179951 3.663751 0.117367
H 1.525781 3.008191 -1.406113
H 2.627748 -2.982333 -0.488078
H 2.493233 -1.512952 -1.456329
C 4.333938 -0.874886 0.795127
C 5.185945 0.983889 -0.506518
H 3.808809 2.562319 -1.004455
C 5.361238 -0.271992 0.136210
H 4.477187 -1.835785 1.283551
H 6.011145 1.448203 -1.034060
H 6.331778 -0.759005 0.093969
O -0.731152 1.393885 0.186972
H 2.792826 0.005474 1.978890
Sc -2.360707 0.228153 0.067670
Cl -3.420468 -0.841707 1.971021
Cl -2.757928 -0.906453 -2.030531
Cl -3.878580 2.095333 -0.237824
35
G-ScCl3-dis-2 Eopt -2850.981427
O 0.801670 -1.224658 -0.603122
C -0.374133 -0.745893 -0.221595
C -0.497821 0.515483 0.244717
N -1.491982 -1.594144 -0.335949
O 0.646628 1.327276 0.102759
C -1.764214 1.183957 0.635687
C -1.346053 -2.829400 -1.089539
C 0.403746 2.341151 -0.912626
C -2.091719 2.246516 -0.421295
H -1.625514 1.701458 1.597792
H -0.317402 -3.170027 -1.001325
H -1.583916 -2.686675 -2.151858
H -2.018452 -3.583016 -0.673824
C -0.854540 3.138134 -0.591611
H 1.292382 2.971341 -0.922777
H 0.312218 1.824506 -1.876946
H -2.338635 1.749093 -1.369503
H -2.956339 2.850273 -0.125164
H -1.002262 3.866852 -1.395249
H -0.685197 3.704036 0.332888
C -2.750874 -1.046616 -0.138646
C -2.819250 0.074170 0.878076
C -3.887928 -1.557733 -0.730114
C -4.204041 0.626667 1.042937
C -5.161343 -1.011571 -0.465100
H -3.809012 -2.385756 -1.426159
C -5.290399 0.094417 0.418596
H -4.312566 1.472175 1.717976
H -6.034498 -1.421814 -0.959145
H -6.273702 0.523117 0.592381
H -2.502299 -0.382283 1.836889
Sc 2.373941 -0.176041 0.082341
Cl 3.946794 -1.993844 -0.229008
Cl 3.619756 1.495289 -1.164521
Cl 2.238823 -0.158742 2.484851
35
G-ScCl3-dis-3 Eopt -2850.979480
O -0.699433 -1.404728 -0.075946
C 0.484604 -0.838002 -0.236581
C 0.598323 0.491843 -0.418020
N 1.625210 -1.667365 -0.188430
O -0.599905 1.217489 -0.258830
C 1.845828 1.259237 -0.657212
C 1.464916 -3.007073 0.354475
C -0.470420 2.196507 0.810068
C 2.043780 2.251750 0.494757
H 1.734833 1.856352 -1.576767
H 0.476775 -3.373346 0.086806
H 1.563200 -3.018274 1.448427
H 2.226331 -3.659796 -0.077351
C 0.755357 3.077620 0.612149
H -1.398544 2.772156 0.789881
H -0.402311 1.644534 1.755201
H 2.233929 1.702143 1.426776
H 2.899803 2.909567 0.311591
H 0.805517 3.770074 1.458580
H 0.624360 3.679900 -0.295256
C 2.862740 -1.042424 -0.133923
C 2.977470 0.232089 -0.944805
C 3.952124 -1.593589 0.508356
C 4.342740 0.848755 -0.869256
C 5.213812 -0.963029 0.485392
H 3.842311 -2.524262 1.054491
C 5.380135 0.267640 -0.206105
H 4.482505 1.788358 -1.398185
H 6.046917 -1.409488 1.015863
H 6.352680 0.752356 -0.200175
H 2.786133 -0.076481 -1.991342
Sc -2.359247 -0.254926 -0.141864
Cl -3.843305 -2.081329 -0.701468
Cl -3.575059 1.565356 -1.218431
Cl -2.841664 0.081925 2.177079
35
G-ScCl3-dis-4 Eopt -2850.981041
O 0.731178 -1.302900 -0.453366
C -0.429908 -0.777009 -0.103067
C -0.527862 0.516136 0.275635
N -1.565109 -1.607263 -0.165683
O 0.641393 1.285551 0.076021
C -1.785833 1.243300 0.586371
C -1.432352 -2.912948 -0.791686
C 0.428651 2.235954 -1.008216
C -2.068718 2.233043 -0.551131
H -1.660797 1.827083 1.511641
H -0.418526 -3.272165 -0.633192
H -1.628532 -2.869128 -1.871016
H -2.141985 -3.602842 -0.329785
C -0.810612 3.083855 -0.758616
H 1.333651 2.840692 -1.052564
H 0.327235 1.656213 -1.935596

H -2.307333 1.673590 -1.466477
H -2.926104 2.873293 -0.317651
H -0.927563 3.760313 -1.611549
H -0.639968 3.705847 0.128802
C -2.813680 -1.016490 -0.046616
C -2.879055 0.181591 0.877903
C -3.947011 -1.546478 -0.628335
C -4.252264 0.781865 0.952218
C -5.211749 -0.948585 -0.448411
H -3.869418 -2.429778 -1.253096
C -5.334340 0.229349 0.337765
H -4.357553 1.682831 1.551785
H -6.081126 -1.374502 -0.935873
H -6.309245 0.697633 0.443289
H -2.606528 -0.208174 1.878767
Sc 2.368852 -0.214256 0.019365
Cl 3.860423 -1.986333 -0.681133
Cl 2.440841 -0.318442 2.401487
Cl 3.747647 1.663988 -0.690855
35
G-ScCl3-dis-5 Eopt -
2850.976392
O -0.632407 -1.229762 0.409366
C 0.439780 0.816198 0.246081
C 0.567542 -0.498792 0.486863
C -0.425171 -2.575792 -0.091228
N 1.572587 1.655046 0.145743
C 1.836927 -1.255497 0.602245
C 0.628302 -2.561744 -1.191790
H -1.397119 -2.904946 -0.459070
H -0.128374 -3.205314 0.754720
C 2.820930 1.057227 0.116669
C 1.390082 2.982479 -0.418089
C 2.014735 -2.113059 -0.668072
H 1.791585 -1.926305 1.472025
H 0.678928 -3.558171 -1.640086
H 0.274452 -1.876013 -1.969385
C 2.951683 -0.224430 0.916494
C 3.912649 1.635345 -0.501812
H 0.384369 3.322780 -0.184023
H 2.118884 3.663784 0.026531
H 1.521132 2.982308 -1.508516
H 2.649228 -2.978645 -0.450183
H 2.525076 -1.520762 -1.437094
C 4.322630 -0.828463 0.831274
C 5.183099 1.024420 -0.472895
H 3.796113 2.571485 -1.036867
C 5.358454 -0.220713 0.190514
H 4.465198 -1.781524 1.335065
H 6.015871 1.493939 -0.983661
H 6.335683 -0.695939 0.178139
O -0.758799 1.358227 0.059102
H 2.752896 0.063084 1.967972
Sc -2.381979 0.184632 0.066238
Cl -3.275864 -0.629742 2.166884
Cl -2.776831 -1.005553 -2.007285
Cl -3.926757 2.013932 -0.306778
35
G-ScCl3-dis-6 Eopt -
2850.976183
O 0.635919 1.237829 0.336865
C -0.433511 -0.798642 0.058212
C -0.557623 0.492200 0.411352
C 0.393079 2.616635 -0.044797
N -1.563981 -1.634783 -0.074386
C -1.830156 1.225632 0.618576
C -0.692332 2.672078 -1.112073
H 1.348401 2.991216 -0.409215
H 0.108244 3.168456 0.857679
C -2.816638 -1.049613 -0.013738
C -1.391425 -2.916288 -0.738677
C -2.056670 2.166931 -0.582679
H -1.766444 1.833255 1.532317
H -0.767482 3.698857 -1.481327
H -0.357053 2.052354 -1.951521
C -2.927920 0.164046 0.888060
C -3.924500 -1.589294 -0.638015
H -0.376234 -3.263471 -0.562916
H -2.100853 -3.634396 -0.321478
H -1.558396 -2.838166 -1.821301
H -2.695376 3.005781 -0.286588
H -2.582540 1.621937 -1.375853
C -4.304530 0.761552 0.893163
C -5.197021 -0.993813 -0.520430
H -3.819159 -2.480258 -1.247407
C -5.357283 0.195764 0.241503
H -4.436462 1.672024 1.472855
H -6.043506 -1.430082 -1.038108
H -6.337101 0.662490 0.297055
O 0.757810 -1.314564 -0.215891
H -2.693803 -0.203019 1.906986
Sc 2.388872 -0.183156 0.044972
Cl 3.900325 -1.988653 -0.534508
Cl 3.266913 1.484262 -1.496926
Cl 2.849743 -0.003635 2.400527
35
G-ScCl3-dis-7 Eopt -
2850.977256
O 0.653804 -1.171368 -0.496101
C -0.476880 0.843520 -0.423334
C -0.574792 -0.487758 -0.564981
C 0.516765 -2.501757 0.069385
N -1.631890 1.654996 -0.329570
C -1.818453 -1.290508 -0.624743
C -0.526009 -2.495419 1.181115
H 1.508341 -2.765848 0.440341
H 0.248814 -3.183755 -0.744996
C -2.853445 1.013028 -0.204437
C -1.465778 3.001055 0.194676
C -1.938168 -2.120115 0.669664
H -1.768688 -1.983593 -1.477134
H -0.527946 -3.480661 1.656505
H -0.191965 -1.774747 1.934336
C -2.975287 -0.305036 -0.944136
C -3.928231 1.570170 0.460134
H -0.489204 3.371611 -0.107422
H -2.244518 3.645028 -0.219253
H -1.530664 3.021668 1.291193
H -2.540056 -3.016283 0.485931
H -2.458910 -1.529328 1.432838
C -4.323426 -0.943323 -0.781934
C -5.175145 0.914258 0.521426
H -3.816729 2.526312 0.959726
C -5.344023 -0.351918 -0.102534
H -4.461635 -1.912444 -1.255346
H -5.994171 1.368813 1.066799
H -6.303955 -0.855976 -0.029161
O 0.712004 1.427017 -0.333549
H -2.822091 -0.059231 -2.013119
Sc 2.350463 0.273827 -0.125768
Cl 2.435400 -0.433659 2.165290
Cl 3.878993 2.150137 -0.133900
Cl 3.675470 -1.353494 -1.357140
133
TS-I-Feng-re-1 Eopt -
3432.535508
O -2.694046 -0.373079 -0.207007
O -0.986332 -1.902142 1.225537
O -0.892035 -2.195973 -1.710470
O 1.381830 -1.356698 -0.526950
N -4.443422 0.335913 1.006909
H -5.072673 0.157119 1.784892
N -2.174617 -2.581301 1.452621
N 3.301003 -2.290510 -1.206079
H 3.760851 -2.975281 -1.799523
N -0.155900 -3.357259 -1.833854
C -4.683256 1.492279 0.171926
C -3.861003 2.619534 0.340256
C -4.108649 3.711660 -0.494367
H -3.501498 4.605665 -0.409509
C -5.134905 3.675317 -1.436250
H -5.311123 4.537830 -2.070905
C -5.936990 2.547349 -1.566185
H -6.734407 2.540901 -2.301133
C -5.724113 1.422427 -0.762884
C -6.545601 0.154967 -0.931548
H -6.631376 -0.327787 0.048633
C -2.794306 2.671523 1.425307
C -3.448270 -0.503509 0.788911
C -3.260504 -1.598950 1.824969
H -4.185959 -2.179868 1.895999
C -2.826023 -1.091509 3.210220
H -3.699291 -0.817440 3.803019
H -2.196940 -0.204497 3.104257
C -2.037151 -2.271682 3.825891
H -2.525672 -2.662974 4.718007
C -1.990310 -3.348460 2.740650
H -2.818394 -4.056621 2.816217
C -0.191332 -4.166772 -0.564317
H 0.376449 -5.077211 -0.770942
H 0.336698 -3.580719 0.189995
C -0.686701 -4.066780 -3.057467
H -0.283155 -5.081619 -3.043759
C -0.151458 -3.224820 -4.223349
H 0.128279 -3.875678 -5.051619
C 1.070319 -2.451253 -3.663850
H 0.860362 -1.382145 -3.592445
H 1.974267 -2.590942 -4.257064
C 1.261662 -3.047595 -2.265201
H 1.766074 -4.016298 -2.328861
C 1.986889 -2.170929 -1.265084
C 4.092174 -1.534476 -0.262595
C 4.126927 -1.964358 1.071227
C 4.871414 -1.190308 1.968786
H 4.921665 -1.483789 3.013696
C 5.553144 -0.055793 1.542340
C 5.521868 0.324640 0.201278
H 6.076018 1.201650 -0.116440
C 4.786149 -0.408188 -0.732476
C 4.693082 0.009634 -2.190458
C 3.410540 -3.216652 1.550795
H 3.042623 -3.759771 0.674188
H 4.623888 -0.898912 -2.799548
H -2.249741 1.716389 1.423747
H -0.911839 -2.528198 -4.574398
H -1.771676 -4.086714 -2.975090
H -1.042429 -3.878807 2.668291
H -1.029451 -1.960812 4.103581
C -1.750372 3.766612 1.201168
H -1.301259 3.705573 0.203976
H -2.181631 4.765044 1.321213
H -0.960123 3.666341 1.952717
C -3.439902 2.851117 2.808442
H -3.960468 3.813014 2.852639
H -4.166919 2.066382 3.033452
H -2.674201 2.838953 3.590470
C -5.818497 -0.818022 -1.873609
H -5.730463 -0.375803 -2.871780
H -4.808535 -1.049617 -1.522305
H -6.378745 -1.753703 -1.960175
C -7.967337 0.418367 -1.432530
H -8.545241 -0.508800 -1.392511
H -8.477384 1.167961 -0.820928

H -7.970802 0.760196 -2.472086
C 3.415836 0.831924 -2.416473
H 3.318600 1.105574 -3.471965
H 2.512154 0.291675 -2.113711
H 3.472823 1.753468 -1.826063
C 5.911938 0.797712 -2.674195
H 5.954894 1.790669 -2.214571
H 6.845092 0.273049 -2.451472
H 5.847691 0.941224 -3.755967
C 2.199890 -2.868163 2.428000
H 2.518192 -2.370500 3.351134
H 1.500992 -2.209722 1.901115
H 1.665751 -3.781833 2.709687
C 4.364056 -4.160814 2.293712
H 5.235478 -4.406015 1.680712
H 4.717157 -3.714745 3.228356
H 3.844935 -5.090251 2.544445
C -2.587887 -3.457043 0.294001
H -2.799611 -2.793781 -0.541916
H -3.517681 -3.935512 0.609467
C -1.589616 -4.547137 -0.087515
H -2.091307 -5.149024 -0.852066
H -1.438736 -5.226860 0.756928
Sc -0.619445 -0.685931 -0.394588
H 6.124832 0.530190 2.255167
O -0.236595 1.219216 -1.143638
C 0.680788 1.965346 -0.636375
C 1.238057 1.594957 0.630638
N 1.129547 3.035459 -1.327142
O 0.542007 0.571358 1.199553
C 2.438677 2.131870 1.261664
C 2.091481 3.919875 -0.785980
C 0.667480 3.199654 -2.710897
C 0.579816 0.455362 2.661091
C 2.479480 2.080608 2.760720
H 3.367812 1.964859 0.714745
C 2.047303 4.208464 0.600526
C 3.132587 4.399978 -1.580364
H -0.402825 3.000948 -2.749421
H 0.863300 4.224598 -3.018078
H 1.186303 2.500064 -3.372948
C 1.966914 0.705031 3.212388
H 0.218600 -0.554853 2.852069
H -0.139585 1.190852 3.039962
H 1.834103 2.860492 3.190518
H 3.496119 2.255908 3.117561
C 3.048196 5.042589 1.149728
H 1.105205 4.094663 1.130224
C 4.132806 5.178146 -1.004599
H 3.179230 4.146038 -2.634644
H 1.907239 0.645494 4.303529
H 2.648843 -0.076799 2.865036
C 4.093066 5.493488 0.363421
H 2.985549 5.318974 2.197124
H 4.947199 5.539994 -1.622480
H 4.869179 6.115828 0.795587
133
TS-I-Feng-re-2 Eopt -3432.536531
O -2.614260 -0.367737 -0.123160
O -0.828820 -1.904856 1.228665
O -0.853609 -2.206225 -1.688790
O 1.442776 -1.353309 -0.544442
N -4.439943 0.183090 1.059525
H -5.053420 -0.039913 1.838622
N -2.002356 -2.584008 1.518976
N 3.352001 -2.288964 -1.239732
H 3.813306 -2.970964 -1.835126
N -0.108828 -3.360070 -1.830404
C -4.771245 1.311082 0.217873
C -4.025309 2.491821 0.366210
C -4.361408 3.561950 -0.465548
H -3.813413 4.494803 -0.392686
C -5.400271 3.451647 -1.387487
H -5.647732 4.297368 -2.020961
C -6.124331 2.269472 -1.500697
H -6.931721 2.204894 -2.221796
C -5.819760 1.164711 -0.699209
C -6.545515 -0.161405 -0.852087
H -6.594287 -0.637631 0.133990
C -2.945678 2.613798 1.429641
C -3.363389 -0.556582 0.867238
C -3.074066 -1.602058 1.930445
H -3.979135 -2.193800 2.103519
C -2.537406 -1.040160 3.256255
H -3.362566 -0.705886 3.885247
H -1.883184 -0.185116 3.066668
C -1.754567 -2.224206 3.875384
H -2.230884 -2.588929 4.785301
C -1.751962 -3.325806 2.810331
H -2.568489 -4.039512 2.939495
C -0.105649 -4.177018 -0.565082
H 0.453335 -5.086963 -0.796319
H 0.449344 -3.596451 0.172520
C -0.656894 -4.068368 -3.046714
H -0.244356 -5.079671 -3.046950
C -0.151641 -3.215200 -4.218047
H 0.116207 -3.858911 -5.055803
C 1.075719 -2.435387 -3.678755
H 0.860465 -1.367967 -3.598134
H 1.968665 -2.566471 -4.290083
C 1.298825 -3.037525 -2.287763
H 1.807784 -4.002747 -2.367196
C 2.037690 -2.166452 -1.292359
C 4.126516 -1.527712 -0.286164
C 4.211331 -2.009945 1.028197
C 4.886558 -1.209263 1.956057
H 4.968802 -1.538235 2.987977
C 5.463293 -0.002442 1.572865
C 5.397379 0.423581 0.247520
H 5.879351 1.353345 -0.037545
C 4.724159 -0.332767 -0.715214
C 4.621973 0.123613 -2.161900
C 3.625669 -3.349083 1.450995
H 3.323267 -3.891734 0.549455
H 4.541908 -0.770172 -2.790539
H -2.387337 1.670036 1.461332
H -0.924133 -2.522312 -4.549453
H -1.739848 -4.098433 -2.942615
H -0.803449 -3.849441 2.702812
H -0.735829 -1.926382 4.125015
C -1.923852 3.713427 1.139177
H -1.495840 3.615353 0.135697
H -2.364299 4.711183 1.227624
H -1.117609 3.649768 1.876568
C -3.577674 2.838926 2.812144
H -4.111265 3.794746 2.827063
H -4.292033 2.052771 3.072770
H -2.802298 2.865191 3.584124
C -5.744605 -1.086595 -1.782326
H -5.698550 -0.655708 -2.788216
H -4.716864 -1.224903 -1.433059
H -6.223754 -2.067798 -1.849534
C -7.982467 -0.012694 -1.355693
H -8.487460 -0.981209 -1.308425
H -8.549509 0.700186 -0.750378
H -8.010896 0.319688 -2.397975
C 3.353697 0.962758 -2.375840
H 3.259546 1.245582 -3.429150
H 2.447249 0.423527 -2.079954
H 3.415310 1.882542 -1.781215
C 5.852103 0.905575 -2.629891
H 5.910713 1.887399 -2.149229
H 6.777254 0.362257 -2.419123
H 5.789199 1.073597 -3.708308
C 2.381332 -3.175759 2.333973
H 2.631392 -2.638642 3.256503
H 1.589741 -2.617976 1.822215
H 1.984461 -4.157266 2.613686
C 4.673176 -4.213903 2.164138
H 5.574445 -4.326490 1.555564
H 4.961281 -3.778551 3.125755
H 4.261513 -5.208049 2.359753
C -2.465752 -3.470361 0.388937
H -2.715759 -2.813222 -0.441458
H -3.379308 -3.948453 0.749464
C -1.484017 -4.559067 -0.035750
H -2.016523 -5.159122 -0.780681
H -1.297933 -5.239581 0.801290
Sc -0.552608 -0.675380 -0.399387
H 5.986925 0.602249 2.306954
O -0.249310 1.225314 -1.154712
C 0.631534 2.032793 -0.668004
C 1.281384 1.676608 0.551538
N 0.974263 3.144500 -1.360725
O 0.674454 0.610524 1.133745
C 2.438489 2.315858 1.166653
C 0.489654 3.272758 -2.739753
C 1.861575 4.095424 -0.820490
C 1.261575 0.146206 2.386743
C 2.599868 2.216968 2.663197
H 3.346544 2.325518 0.565190
H -0.556159 2.970367 -2.772713
H 1.069920 2.633172 -3.411631
H 0.580779 4.314345 -3.040357
C 2.844548 4.679691 -1.621196
C 1.818022 4.348776 0.576014
C 1.500298 1.345273 3.280130
H 0.534874 -0.566983 2.773469
H 2.193705 -0.371036 2.130967
H 3.579429 1.771842 2.872443
H 2.607178 3.212840 3.123126
C 3.777997 5.535223 -1.046276
H 2.898766 4.444415 -2.679344
C 2.744926 5.269800 1.121903
H 0.897164 4.146002 1.115753
H 1.793657 1.000109 4.274391
H 0.559819 1.898968 3.385224
C 3.728535 5.828124 0.328441
H 4.548808 5.977264 -1.667850
H 2.672035 5.529980 2.172681
H 4.448440 6.516150 0.758245
133
TS-I-Feng-si-1 Eopt -3432.536673
O -2.455184 -0.499944 -0.111817
O -0.541521 -1.933683 1.159545
O -0.580728 -2.453510 -1.690194
O 1.619884 -1.279146 -0.621803
N -4.237588 0.006114 1.158204
H -4.765296 -0.206983 2.000417
N -1.662468 -2.628670 1.575598
N 3.597362 -2.233470 -1.044875
H 4.120711 -2.975467 -1.500512
N 0.233945 -3.567542 -1.705537
C -4.814068 0.951537 0.228523
C -4.216917 2.211769 0.065219
C -4.834193 3.087988 -0.831984
H -4.414483 4.072687 -1.000220
C -5.990972 2.722264 -1.515498
H -6.453579 3.424674 -2.201279
C -6.556290 1.466088 -1.327102
H -7.456150 1.198032 -1.869429

C	-5.973208	0.546746	-0.450881
C	-6.524589	-0.861053	-0.290892
H	-6.369353	-1.177941	0.746933
C	-2.982900	2.621631	0.857503
C	-3.130348	-0.674682	0.933235
C	-2.723951	-1.651536	2.023136
H	-3.594818	-2.254544	2.300503
C	-2.093046	-1.007391	3.265207
H	-2.867557	-0.627200	3.931427
H	-1.455029	-0.171481	2.965971
C	-1.267059	-2.153606	3.897406
H	-1.703936	-2.488512	4.838197
C	-1.290990	-3.298919	2.877726
H	-2.063567	-4.039597	3.094231
C	0.214651	-4.299047	-0.388841
H	0.796411	-5.210077	-0.547147
H	0.738825	-3.663629	0.324920
C	-0.208411	-4.396359	-2.889445
H	0.257100	-5.379045	-2.788859
C	0.312443	-3.609530	-4.097613
H	0.644514	-4.298965	-4.873669
C	1.479769	-2.739257	-3.565287
H	1.225158	-1.677501	-3.597380
H	2.410028	-2.892087	-4.113181
C	1.646823	-3.200053	-2.111491
H	2.215234	-4.133395	-2.066785
C	2.285025	-2.177414	-1.192413
C	4.289780	-1.219276	-0.282225
C	4.602579	-1.499223	1.057254
C	5.161202	-0.463429	1.811435
H	5.416201	-0.632441	2.852494
C	5.393080	0.788020	1.243817
C	5.108204	1.017365	-0.099198
H	5.330628	1.987425	-0.535965
C	4.560670	0.009863	-0.900741
C	4.312216	0.253821	-2.380618
C	4.314010	-2.861220	1.669308
H	4.316734	-3.600026	0.860794
H	4.049396	-0.701247	-2.846302
H	-2.275135	1.781101	0.835501
H	-0.472351	-2.977364	-4.511314
H	-1.292146	-4.481124	-2.836770
H	-0.330665	-3.788448	2.723366
H	-0.245087	-1.830660	4.099861
C	-2.267344	3.832292	0.254041
H	-2.034790	3.680738	-0.804646
H	-2.871226	4.740166	0.344989
H	-1.333713	4.018615	0.791625
C	-3.338338	2.911131	2.324125
H	-4.010640	3.773126	2.382832
H	-3.832469	2.063354	2.805759
H	-2.430990	3.142726	2.891459
C	-5.745511	-1.825918	-1.199718
H	-5.915548	-1.567399	-2.250185
H	-4.666847	-1.777884	-1.019757
H	-6.078138	-2.855742	-1.039658
C	-8.025306	-0.963975	-0.569755
H	-8.376908	-1.964514	-0.304389
H	-8.593945	-0.234209	0.013406
H	-8.249039	-0.809830	-1.629732
C	3.143656	1.220486	-2.608823
H	2.961876	1.344801	-3.680953
H	2.225252	0.845619	-2.146399
H	3.369967	2.208969	-2.191493
C	5.579835	0.762986	-3.078953
H	5.859148	1.758191	-2.719613
H	6.423679	0.088619	-2.910077
H	5.405150	0.835437	-4.156076
C	2.920134	-2.882974	2.314722
H	2.886524	-2.192196	3.166002
H	2.138522	-2.579319	1.609788
H	2.686653	-3.887459	2.681292
C	5.376664	-3.292356	2.683814
H	6.382807	-3.225624	2.261060
H	5.341690	-2.679012	3.589264
H	5.197043	-4.328345	2.983674
C	-2.163099	-3.584731	0.522890
H	-2.455808	-2.980124	-0.333431
H	-3.054720	-4.055272	0.944556
C	-1.172115	-4.676844	0.124199
H	-1.691693	-5.292104	-0.617218
H	-0.996233	-5.341728	0.975837
Sc	-0.419910	-0.786887	-0.552824
H	5.818757	1.583358	1.848118
O	-0.189964	1.112374	-1.353753
C	0.404540	2.081541	-0.754360
C	0.744106	1.924575	0.630060
N	0.663537	3.219667	-1.424611
O	0.479368	0.661489	1.066571
C	1.249686	2.940195	1.551304
C	0.167072	3.341598	-2.800540
C	1.384448	4.280667	-0.824012
C	1.369646	0.152135	2.121824
C	2.157417	2.455125	2.643017
H	0.566845	3.757302	1.782642
H	0.399444	2.423247	-3.340967
H	0.670758	4.183725	-3.270019
H	-0.915232	3.496113	-2.810758
C	2.470622	3.963485	0.028046
C	0.955884	5.596607	-0.979705
C	1.570341	1.162818	3.228355
H	0.885030	-0.764334	2.450868
H	2.313603	-0.088763	1.617000
H	3.152723	2.227128	2.233420
H	2.277430	3.223490	3.408068
C	3.166146	5.022831	0.654340
H	2.957763	2.994906	-0.071951
C	1.626240	6.617593	-0.310529
H	0.091850	5.818835	-1.598390
H	2.255913	0.719717	3.958395
H	0.621129	1.364351	3.736993
C	2.730419	6.328154	0.507372
H	4.040139	4.794887	1.256294
H	1.288138	7.641532	-0.424221
H	3.256148	7.134887	1.006689

133

TS-I-Feng-si-2 Eopt -
3432.537483

O	-2.636318	-0.267089	-0.126073
O	-0.980115	-1.908919	1.211892
O	-0.836793	-2.209804	-1.718072
O	1.402598	-1.375349	-0.408659
N	-4.506637	0.276534	0.985179
H	-5.165765	0.031242	1.719166
N	-2.171383	-2.581690	1.436541
N	3.343079	-2.277275	-1.091071
H	3.808367	-2.959923	-1.682620
N	-0.097395	-3.372460	-1.804012
C	-4.793076	1.432818	0.165531
C	-4.019394	2.591472	0.343121
C	-4.327228	3.686717	-0.466929
H	-3.757312	4.604146	-0.373710
C	-5.365750	3.622458	-1.393470
H	-5.591093	4.488311	-2.007686
C	-6.116283	2.460517	-1.537013
H	-6.921644	2.431308	-2.262544
C	-5.839873	1.331193	-0.760329
C	-6.591034	0.023708	-0.950333
H	-6.677163	-0.466296	0.026597
C	-2.934920	2.660662	1.407587
C	-3.446923	-0.489480	0.807261
C	-3.256973	-1.604354	1.821178
H	-4.180319	-2.189308	1.880888
C	-2.832303	-1.118179	3.217350
H	-3.714677	-0.876451	3.810690
H	-2.224426	-0.214616	3.134859
C	-2.020885	-2.295760	3.809009
H	-2.486054	-2.696414	4.709403
C	-1.988793	-3.363966	2.715723
H	-2.823686	-4.064707	2.788604
C	-0.189179	-4.183538	-0.538954
H	0.370703	-5.101692	-0.731609
H	0.321736	-3.608625	0.235123
C	-0.569862	-4.082891	-3.051217
H	-0.167334	-5.097509	-3.016962
C	0.020367	-3.242105	-4.190760
H	0.335492	-3.893185	-5.006034
C	1.217591	-2.473223	-3.575243
H	1.011654	-1.402236	-3.520067
H	2.148446	-2.621097	-4.123694
C	1.337019	-3.063078	-2.164711
H	1.844483	-4.031818	-2.198200
C	2.026583	-2.177906	-1.144804
C	4.141373	-1.452636	-0.216207
C	4.239825	-1.808457	1.135282
C	4.977259	-0.953188	1.962355
H	5.068856	-1.179532	3.020668
C	5.595717	0.181721	1.448380
C	5.519821	0.475135	0.086713
H	6.041789	1.343874	-0.301413
C	4.787906	-0.339599	-0.779791
C	4.684862	-0.059369	-2.272169
C	3.599107	-3.068020	1.697346
H	3.284720	-3.694438	0.856072
H	4.649291	-1.027564	-2.785031
H	-2.372619	1.718090	1.375430
H	-0.721007	-2.542701	-4.575355
H	-1.657511	-4.102985	-3.020595
H	-1.045617	-3.901917	2.635204
H	-1.009406	-1.978570	4.064855
C	-1.922781	3.783736	1.176639
H	-1.472235	3.732205	0.179169
H	-2.382896	4.769563	1.294290
H	-1.127133	3.710028	1.924153
C	-3.557766	2.803970	2.805244
H	-4.086889	3.759308	2.881147
H	-4.273299	2.006648	3.024197
H	-2.777097	2.781613	3.572189
C	-5.786862	-0.905582	-1.873927
H	-5.705530	-0.460834	-2.871411
H	-4.771291	-1.072924	-1.501983
H	-6.285353	-1.874788	-1.968269
C	-8.010856	0.210118	-1.488398
H	-8.536597	-0.748199	-1.468551
H	-8.578737	0.925815	-0.887249
H	-8.004967	0.557885	-2.526006
C	3.390539	0.696765	-2.613174
H	3.228858	0.706992	-3.695524
H	2.506871	0.256998	-2.135757
H	3.471859	1.735566	-2.276482
C	5.891297	0.702006	-2.825007
H	5.924125	1.728456	-2.445301
H	6.831981	0.207449	-2.567817
H	5.818843	0.759161	-3.914395
C	2.351317	-2.744881	2.531888
H	2.615986	-2.131921	3.401108
H	1.604033	-2.206420	1.940346
H	1.895421	-3.670354	2.898565
C	4.599474	-3.889066	2.520364
H	5.504767	-4.102975	1.945825
H	4.890030	-3.364301	3.435516
H	4.144181	-4.839000	2.814229
C	-2.590748	-3.439353	0.267934

H -2.783114 -2.764207 -0.563983
H -3.532549 -3.903992 0.567914
C -1.608658 -4.543446 -0.114435
H -2.101538 -5.115748 -0.906870
H -1.498605 -5.245389 0.717949
Sc -0.605357 -0.687225 -0.416712
H 6.162925 0.831503 2.107813
O -0.081200 1.107668 -1.290123
C 0.694813 1.951403 -0.698118
C 0.867122 1.853283 0.714960
N 1.264332 2.957340 -1.405126
O 0.283462 0.725757 1.206434
C 1.586275 2.777554 1.580637
C 0.791812 3.202201 -2.772907
C 2.298227 3.735217 -0.853964
C 0.520954 0.435354 2.623553
C 2.201715 2.240655 2.844940
H 1.244696 3.810737 1.571793
H 0.599591 2.242786 -3.251833
H -0.129283 3.791121 -2.759466
H 1.570394 3.734160 -3.316516
C 2.403291 5.095246 -1.149024
C 3.174188 3.125894 0.084463
C 1.968683 0.734281 2.945110
H 0.250800 -0.614457 2.726321
H -0.166514 1.063281 3.201270
H 1.747542 2.746200 3.705486
H 3.274089 2.471606 2.881810
C 3.390451 5.854771 -0.531458
H 1.702192 5.561330 -1.833112
C 4.200682 3.913030 0.657224
H 3.290297 2.042825 0.074677
H 2.201295 0.365119 3.947349
H 2.602826 0.189329 2.234254
C 4.288945 5.263029 0.374764
H 3.463994 6.913654 -0.753103
H 4.920614 3.440087 1.318220
H 5.068565 5.864163 0.829906
35
TS-I-ScCl3-con-1 Eopt -
2850.786077
O 0.667094 -1.355373 -0.002757
C -0.508857 -0.834273 0.052692
C -0.624269 0.574983 0.242293
N -1.604123 -1.633714 0.004981
O 0.582249 1.177489 0.188670
C -1.848168 1.322203 0.509445
C -1.428970 -3.078469 0.188247
C 0.665655 2.633761 0.021399
C -1.826315 2.770597 0.127403
H -2.382818 1.039100 1.415509
H -0.463188 -3.366700 -0.220687
H -2.226403 -3.594005 -0.344578
H -1.462167 -3.334667 1.251071
C -0.506006 3.360795 0.646880
H 1.619341 2.901737 0.475190
H 0.716888 2.798588 -1.058136
H -1.865657 2.885917 -0.964831
H -2.687072 3.290674 0.550286
H -0.413997 4.417079 0.376448
H -0.461762 3.288142 1.738124
C -2.896736 -1.095505 -0.148143
C -3.053620 0.093304 -0.906123
C -3.986768 -1.664183 0.512094
C -4.344601 0.654426 -1.026921
H -2.291556 0.357781 -1.635112
C -5.239511 -1.066722 0.411542
H -3.854043 -2.552482 1.120184
C -5.417364 0.097634 -0.353497
H -4.479516 1.528970 -1.654966
H -6.082422 -1.507535 0.932491
H -6.402845 0.543224 -0.437252
Sc 2.433087 -0.317401 -0.066025
Cl 3.761605 -2.238930 -0.532977
Cl 3.078855 0.344070 2.122536
Cl 3.098935 1.201764 -1.786221
35
TS-I-ScCl3-con-2 Eopt -
2850.788256
O 0.584069 1.152222 0.284398
C -0.476355 -0.858866 0.101202
C -0.602368 0.526984 0.397923
C 0.618265 2.596447 0.492613
N -1.574204 -1.659879 0.012077
C -1.824589 1.247132 0.715421
C -0.582056 3.220122 -0.189501
H 1.569072 2.904776 0.060495
H 0.622673 2.764825 1.573905
C -2.849638 -1.105982 -0.159675
C -1.411376 -3.107882 0.172026
C -1.862623 2.734416 0.492110
H -2.416771 0.842930 1.533609
H -0.499618 4.307797 -0.128131
H -0.565818 2.946298 -1.250672
C -2.964490 0.143210 -0.833449
C -3.976027 -1.713101 0.402626
H -0.408342 -3.378907 -0.149033
H -1.546509 -3.392814 1.219388
H -2.148645 -3.614062 -0.449910
H -1.971459 3.231823 1.464027
H -2.749038 3.010315 -0.092713
C -4.249694 0.720795 -0.971433
H -2.181954 0.434550 -1.529608
C -5.216339 -1.096163 0.292585
H -3.879463 -2.647425 0.944437
C -5.353019 0.126869 -0.390123
H -4.354876 1.635026 -1.546393
H -6.085315 -1.566182 0.740080
H -6.331780 0.584635 -0.485459
O 0.706639 -1.362674 -0.013809
Sc 2.448034 -0.290188 -0.081082
Cl 3.813094 -2.175246 -0.592364
Cl 3.090886 0.365665 2.117408
Cl 2.903947 1.264242 -1.853544
35
TS-I-ScCl3-con-3 Eopt -
2850.785564
O 0.692912 -1.319945 -0.269376
C -0.483935 -0.813083 -0.149720
C -0.603973 0.585513 0.110670
N -1.571635 -1.620147 -0.199283
O 0.597700 1.197062 0.049480
C -1.819155 1.315332 0.457109
C -1.370199 -3.070986 -0.118441
C 0.677737 2.659202 -0.050949
C -1.814005 2.776285 0.124773
H -2.305456 1.004549 1.381509
H -0.453060 -3.326050 -0.645369
H -2.218393 -3.563233 -0.590972
H -1.285845 -3.387678 0.925007
C -0.482314 3.354175 0.630665
H 1.642414 2.904578 0.392548
H 0.707839 2.873562 -1.122788
H -1.883526 2.929213 -0.961253
H -2.664452 3.277483 0.589433
H -0.403315 4.420803 0.399571
H -0.409240 3.239188 1.716766
C -2.876956 -1.088772 -0.240865
C -3.093699 0.129483 -0.933356
C -3.911829 -1.693791 0.472780
C -4.394206 0.681910 -0.939674
H -2.383273 0.434627 -1.697895
C -5.172366 -1.103969 0.488476
H -3.728096 -2.604070 1.033553
C -5.411986 0.088108 -0.214124
H -4.579595 1.580958 -1.518296
H -5.971956 -1.572040 1.052191
H -6.403511 0.528155 -0.207463
Sc 2.449190 -0.287962 -0.028598
Cl 3.814098 -2.175118 -0.525825
Cl 2.583573 0.018785 2.325034
Cl 3.374350 1.374852 -1.494261
35
TS-I-ScCl3-con-4 Eopt -
2850.786870
O -0.659077 -1.388412 -0.161873
C 0.507721 -0.849266 -0.165443
C 0.604902 0.570109 -0.301316
N 1.610058 -1.633658 -0.112714
O -0.616285 1.152406 -0.284630
C 1.817686 1.351546 -0.525270
C 1.448019 -3.081188 -0.288098
C -0.709477 2.573044 0.074467
C 1.776891 2.775622 -0.057695
H 2.351270 1.130644 -1.449714
H 0.509475 -3.385581 0.170559
H 2.280442 -3.582137 0.202929
H 1.429735 -3.337844 -1.351010
C 0.434936 3.380084 -0.501566
H -1.681958 2.877529 -0.310127
H -0.717151 2.598606 1.168465
H 1.842313 2.823385 1.038543
H 2.619075 3.335643 -0.467130
H 0.329581 4.406629 -0.137850
H 0.368967 3.401847 -1.594044
C 2.899032 -1.080871 0.039640
C 3.048035 0.090573 0.824701
C 3.985058 -1.617783 -0.650645
C 4.334145 0.664674 0.942842
H 2.292691 0.323220 1.571858
C 5.231892 -1.006385 -0.552963
H 3.853113 -2.492064 -1.279214
C 5.404269 0.139740 0.239658
H 4.467310 1.524796 1.590891
H 6.072770 -1.421952 -1.097348
H 6.384691 0.596859 0.320972
Sc -2.429512 -0.346977 -0.029185
Cl -2.465557 0.446734 2.211333
Cl -3.794315 -2.300852 -0.011328
Cl -3.572407 1.168894 -1.486696
35
TS-I-ScCl3-con-5 Eopt -
2850.788430
O -0.615544 1.102571 -0.383745
C 0.507334 -0.874363 -0.174988
C 0.599262 0.520702 -0.450677
C -0.687476 2.562782 -0.411385
N 1.619302 -1.650154 -0.080054
C 1.804036 1.287605 -0.733497
C 0.508494 3.140053 0.316770
H -1.637478 2.791382 0.069720
H -0.723721 2.860672 -1.463316
C 2.888843 -1.070753 0.071614
C 1.479317 -3.104468 -0.202867
C 1.790783 2.759869 -0.424024
H 2.397929 0.949379 -1.580652
H 0.395876 4.225625 0.367569
H 0.516258 2.759440 1.345106
C 2.991606 0.169364 0.760593
C 4.011996 -1.647884 -0.524343
H 0.505298 -3.392275 0.186477
H 1.554391 -3.408756 -1.250752
H 2.266379 -3.579803 0.380682

H 1.852595 3.321486 -1.364818
H 2.679207 3.037550 0.156767
C 4.268751 0.768025 0.882233
H 2.215759 0.435032 1.474807
C 5.243861 -1.009834 -0.430916
H 3.919104 -2.575618 -1.078459
C 5.370948 0.203736 0.268436
H 4.369964 1.675102 1.469043
H 6.111634 -1.456497 -0.903877
H 6.342606 0.678832 0.351249
O -0.661736 -1.408536 -0.087211
Sc -2.427004 -0.359256 0.007384
Cl -3.798295 -2.304368 0.075300
Cl -3.578449 1.104653 -1.499557
Cl -2.481027 0.669869 2.149652
35
TS-I-ScCl3-con-6 Eopt -
2850.787489
O 0.584790 1.170737 0.059619
C -0.483536 -0.842242 -0.135431
C -0.592877 0.532632 0.217937
C 0.617723 2.601115 0.356834
N -1.584804 -1.641185 -0.192775
C -1.795007 1.230640 0.646816
C -0.617777 3.252584 -0.229622
H 1.546428 2.942890 -0.095711
H 0.668777 2.700624 1.445841
C -2.870465 -1.086331 -0.252991
C -1.410722 -3.093994 -0.099405
C -1.859639 2.725200 0.490782
H -2.325733 0.785983 1.486163
H -0.534834 4.336086 -0.116734
H -0.656500 3.033965 -1.302769
C -3.035199 0.185232 -0.870270
C -3.951301 -1.714983 0.371859
H -0.438417 -3.351995 -0.512462
H -1.458416 -3.417255 0.944478
H -2.198111 -3.577975 -0.675686
H -1.924929 3.178084 1.487859
H -2.776002 3.016715 -0.037803
C -4.326678 0.764175 -0.892427
H -2.302341 0.508261 -1.605402
C -5.195834 -1.096822 0.378126
H -3.813732 -2.666133 0.874094
C -5.383333 0.148162 -0.250457
H -4.473970 1.698300 -1.424513
H -6.028121 -1.582648 0.875675
H -6.366159 0.607178 -0.254779
O 0.690496 -1.342076 -0.327474
Sc 2.440926 -0.302212 -0.087387
Cl 3.415503 1.463032 -1.374389
Cl 2.588240 0.047319 2.258345
Cl 3.886952 -2.136163 -0.539919
35
TS-I-ScCl3-dis-1 Eopt -
2850.778904
O -0.593484 1.156237 -0.478258
C 0.446326 -0.905502 -0.547406
C 0.588996 0.516137 -0.683176
C -0.451988 2.557402 -0.055267
N 1.525198 -1.697664 -0.436796
C 1.774272 1.338133 -0.915718
C 0.718606 2.663608 0.911414
H -1.408039 2.798791 0.406544
H -0.317308 3.155077 -0.960522
C 2.802478 -1.090923 -0.218477
C 1.330872 -3.126451 -0.180015
C 2.059560 2.281882 0.233323
H 1.820279 1.771793 -1.916400
H 0.752990 3.684776 1.299492
H 0.512617 1.996868 1.755643
C 3.282632 -0.193263 -1.203679
C 3.472712 -1.277040 0.979986
H 0.552520 -3.503536 -0.842166
H 2.272359 -3.635337 -0.380156
H 1.027758 -3.295868 0.857504
H 2.578649 3.165685 -0.144360
H 2.717661 1.797252 0.966645
C 4.540798 0.420781 -0.983207
C 4.688203 -0.618267 1.197946
H 3.054171 -1.929412 1.740205
C 5.212437 0.231479 0.214716
H 4.964620 1.048601 -1.759907
H 5.220106 -0.768900 2.130676
H 6.165478 0.722872 0.381466
O -0.731927 -1.395953 -0.495386
H 2.900564 -0.284492 -2.216439
Sc -2.418593 -0.231607 -0.097209
Cl -2.062765 0.129019 2.221335
Cl -3.709509 1.646963 -0.833149
Cl -3.927284 -2.061093 -0.266583
35
TS-I-ScCl3-dis-2 Eopt -
2850.776332
O -0.726783 -1.434850 -0.386553
C 0.457071 -0.962811 -0.444635
C 0.626610 0.467114 -0.501433
N 1.532584 -1.760002 -0.424823
O -0.525578 1.123848 -0.190327
C 1.779132 1.252668 -0.947235
C 1.365663 -3.209710 -0.310493
C -0.350279 2.341801 0.622944
C 2.103095 2.375856 -0.001967
H 1.719508 1.542668 -2.000065
H 0.512447 -3.516160 -0.913978
H 1.190077 -3.497499 0.730250
H 2.275389 -3.683808 -0.676356
C 0.805041 3.198978 0.136356
H -1.311619 2.849379 0.551202
H -0.192037 1.986003 1.645268
H 2.405826 1.977244 0.975470
H 2.915014 2.996024 -0.388421
H 0.932328 4.009564 0.861069
H 0.552644 3.651594 -0.827919
C 2.810133 -1.153691 -0.177603
C 3.319396 -0.270370 -1.160575
C 3.431511 -1.308352 1.050366
C 4.564195 0.357966 -0.905955
C 4.631451 -0.633026 1.302191
H 2.985360 -1.945796 1.807680
C 5.188000 0.199445 0.322008
H 5.012548 0.975192 -1.677363
H 5.127648 -0.758583 2.258040
H 6.128983 0.703691 0.516927
H 2.971316 -0.379523 -2.183409
Sc -2.411649 -0.230385 -0.070122
Cl -2.390332 0.059554 2.280291
Cl -3.495521 1.722651 -0.933557
Cl -3.977447 -1.970385 -0.495019
35
TS-I-ScCl3-dis-3 Eopt -
2850.777631
O 0.524860 1.152868 0.107836
C -0.455927 -0.932510 0.187768
C -0.614547 0.476258 0.400445
C 0.347874 2.601944 -0.014569
N -1.525203 -1.742898 0.149009
C -1.780336 1.250314 0.823976
C -0.893917 2.870678 -0.854669
H 1.263527 2.946582 -0.490473
H 0.275335 3.007241 0.998085
C -2.825134 -1.150018 0.049185
C -1.336545 -3.167960 -0.128048
C -2.178236 2.298739 -0.190557
H -1.732451 1.585303 1.861953
H -0.979524 3.949788 -1.004689
H -0.737174 2.415197 -1.837346
C -3.254324 -0.318497 1.112628
C -3.566502 -1.279433 -1.114017
H -0.483435 -3.530263 0.444332
H -2.241436 -3.691887 0.176145
H -1.149196 -3.336242 -1.192604
H -2.751958 3.084474 0.305211
H -2.821311 1.850624 -0.958931
C -4.530662 0.288396 1.005419
C -4.801673 -0.629327 -1.218313
H -3.187520 -1.881569 -1.934234
C -5.273180 0.156188 -0.157959
H -4.912560 0.866098 1.840586
H -5.389968 -0.736942 -2.122733
H -6.240103 0.642251 -0.237989
O 0.723767 -1.393952 0.008386
H -2.807568 -0.459793 2.092525
Sc 2.439565 -0.219160 -0.052551
Cl 3.904069 -2.035890 -0.506325
Cl 3.234249 1.448416 -1.573393
Cl 2.881126 0.424639 2.184609
35
TS-I-ScCl3-dis-4 Eopt -
2850.774907
O 0.738213 -1.433790 -0.187995
C -0.430899 -0.974833 0.027590
C -0.590314 0.444390 0.240071
N -1.505263 -1.773119 0.033127
O 0.517957 1.145787 -0.141280
C -1.702023 1.173184 0.853793
C -1.350340 -3.206624 -0.220257
C 0.228822 2.398957 -0.865442
C -2.142685 2.346580 0.023765
H -1.542044 1.389077 1.913883
H -0.450182 -3.559167 0.281437
H -1.262981 -3.402775 -1.292885
H -2.227006 -3.715625 0.177945
C -0.880186 3.201200 -0.207668
H 1.178765 2.927884 -0.879033
H -0.040546 2.093661 -1.881209
H -2.548265 2.001829 -0.937037
H -2.914759 2.925123 0.535941
H -1.097495 4.049491 -0.864913
H -0.528412 3.603059 0.747996
C -2.801530 -1.160520 -0.025088
C -3.209848 -0.380426 1.084099
C -3.545480 -1.209146 -1.192097
C -4.475695 0.253798 1.016921
C -4.767239 -0.528998 -1.256039
H -3.177312 -1.768564 -2.046774
C -5.222015 0.202057 -0.150553
H -4.844794 0.791112 1.884149
H -5.358954 -0.571858 -2.163614
H -6.179482 0.710481 -0.200363
H -2.757877 -0.579728 2.051289
Sc 2.426638 -0.189148 -0.070327
Cl 3.924688 -1.988793 -0.489197
Cl 3.535570 1.628548 -1.162785
Cl 2.458332 0.150613 2.274205
35
TS-I-ScCl3-dis-5 Eopt -
2850.778021
O 0.549870 -1.137143 -0.169760
C -0.461736 0.931281 -0.243539
C -0.606510 -0.481030 -0.442333
C 0.394757 -2.578924 0.037354
N -1.537327 1.733499 -0.206728

C -1.766237 -1.281752 -0.828176
C -0.834192 -2.814230 0.906980
H 1.320325 -2.883861 0.523398
H 0.318896 -3.043630 -0.949012
C -2.834381 1.135354 -0.113752
C -1.355764 3.160056 0.068538
C -2.134787 -2.304473 0.224010
H -1.723504 -1.650265 -1.854953
H -0.901956 -3.882702 1.126041
H -0.673805 -2.294621 1.857020
C -3.241678 0.275527 -1.163409
C -3.598852 1.293621 1.030864
H -0.505635 3.525882 -0.506017
H -2.263922 3.679115 -0.234044
H -1.166191 3.330100 1.132382
H -2.684441 -3.124265 -0.243114
H -2.790891 -1.848974 0.977056
C -4.517914 -0.332588 -1.064308
C -4.833946 0.642618 1.128405
H -3.238218 1.918944 1.841921
C -5.283070 -0.172176 0.080603
H -4.882561 -0.931793 -1.892019
H -5.440281 0.773301 2.017710
H -6.250660 -0.657830 0.154208
O 0.714203 1.405212 -0.073258
H -2.779506 0.396172 -2.138975
Sc 2.434249 0.228307 0.029417
Cl 3.206406 -0.971317 -1.881863
Cl 2.943411 -1.026228 1.986508
Cl 3.828869 2.161146 0.085618
35
TS-I-ScCl3-dis-6 Eopt -2850.779122
O 0.594523 -1.152638 -0.440078
C -0.446824 0.902567 -0.517752
C -0.587087 -0.518579 -0.646680
C 0.465034 -2.555085 -0.026509
N -1.526323 1.699188 -0.452406
C -1.769230 -1.349136 -0.864786
C -0.692615 -2.669936 0.956023
H 1.427487 -2.794810 0.423529
H 0.320982 -3.148473 -0.933027
C -2.808302 1.100835 -0.235275
C -1.335613 3.135378 -0.239346
C -2.043830 -2.273178 0.302747
H -1.811795 -1.804534 -1.855823
H -0.726244 -3.695601 1.332008
H -0.470111 -2.014590 1.804582
C -3.279488 0.174667 -1.197685
C -3.492243 1.325366 0.948930
H -0.536125 3.486681 -0.890490
H -2.268871 3.638537 -0.487328
H -1.064199 3.342290 0.800053
H -2.587142 -3.152356 -0.050344
H -2.675388 -1.766256 1.044231
C -4.538380 -0.434891 -0.969461
C -4.709522 0.673387 1.175247
H -3.082135 2.002389 1.691960
C -5.222205 -0.209046 0.215154
H -4.953393 -1.088396 -1.729590
H -5.251726 0.854689 2.096512
H -6.175814 -0.697087 0.388334
O 0.731611 1.392997 -0.433764
H -2.888099 0.234513 -2.209150
Sc 2.427670 0.243295 -0.058132
Cl 2.176233 -0.432032 2.203484
Cl 3.875147 2.133987 -0.025453
Cl 3.630887 -1.474749 -1.204546
35
TS-I-ScCl3-dis-7 Eopt -2850.777932
O -0.539881 -1.185209 0.262454
C 0.427871 0.906119 0.292073
C 0.605360 -0.499452 0.504705
C -0.349811 -2.627101 0.084945
N 1.484133 1.732601 0.229549
C 1.798818 -1.267756 0.854110
C 0.849028 -2.848231 -0.828457
H -1.283804 -2.971890 -0.353969
H -0.216674 -3.062554 1.078485
C 2.789185 1.161499 0.085430
C 1.262116 3.153363 -0.046166
C 2.158392 -2.282650 -0.208884
H 1.805144 -1.632176 1.883001
H 0.940890 -3.919446 -1.024196
H 0.634207 -2.357756 -1.783204
C 3.259751 0.317091 1.120495
C 3.496806 1.324917 -1.094659
H 0.428135 3.505456 0.559887
H 2.170644 3.692332 0.218034
H 1.027031 3.313596 -1.102446
H 2.761378 -3.076806 0.236191
H 2.760746 -1.805748 -0.992902
C 4.540574 -0.269653 0.965480
C 4.737477 0.695367 -1.245767
H 3.087050 1.937472 -1.891971
C 5.248757 -0.104347 -0.214958
H 4.953672 -0.857636 1.778325
H 5.298931 0.829965 -2.163534
H 6.219752 -0.574677 -0.331206
O -0.760260 1.354046 0.132971
H 2.840217 0.434713 2.115393
Sc -2.449793 0.168591 -0.041426
Cl -3.067971 -0.483802 2.159378
Cl -2.927525 -1.356340 -1.832270
Cl -3.823058 2.050191 -0.546887
133
TS-II-Feng-re-1 Eopt -3432.726410
O 2.662496 -0.340364 0.208811
O 1.013992 -1.954727 -1.203437
O 0.828728 -2.088511 1.768386
O -1.432656 -1.381636 0.486235
N 4.462540 0.303746 -0.967538
H 5.118953 0.083617 -1.710780
N 2.211389 -2.630432 -1.355479
N -3.357571 -2.282229 1.175078
H -3.834274 -2.934388 1.789736
N 0.111924 -3.258275 1.919106
C 4.689206 1.491487 -0.177976
C 3.844327 2.597481 -0.371209
C 4.087522 3.723530 0.418977
H 3.459414 4.600807 0.315504
C 5.132532 3.740986 1.340229
H 5.304742 4.629424 1.939379
C 5.956283 2.631757 1.497760
H 6.766069 2.665882 2.218285
C 5.745854 1.474112 0.742412
C 6.581360 0.221081 0.947355
H 6.689329 -0.277973 -0.022651
C 2.750509 2.585127 -1.430129
C 3.448923 -0.519638 -0.747231
C 3.301109 -1.659560 -1.740676
H 4.232489 -2.235192 -1.756192
C 2.908447 -1.221433 -3.162806
H 3.802411 -1.003239 -3.748012
H 2.298573 -0.315354 -3.124768
C 2.107068 -2.418629 -3.730418
H 2.575111 -2.837550 -4.621104
C 2.077240 -3.457514 -2.609641
H 2.928943 -4.140958 -2.643440
C 0.184281 -4.117689 0.684844
H -0.390259 -5.019506 0.911219
H -0.317356 -3.561432 -0.108868
C 0.628171 -3.913226 3.176843
H 0.249080 -4.937648 3.190850
C 0.047095 -3.045263 4.301790
H -0.242825 -3.674830 5.143187
C -1.172884 -2.313179 3.685670
H -0.974767 -1.245291 3.572902
H -2.087360 -2.440747 4.265745
C -1.323166 -2.968720 2.309719
H -1.805715 -3.946438 2.402807
C -2.041777 -2.152458 1.256535
C -4.114523 -1.555754 0.182653
C -4.042718 -1.973031 -1.153552
C -4.751240 -1.218501 -2.095909
H -4.718105 -1.505457 -3.143551
C -5.496770 -0.109757 -1.712134
C -5.559353 0.268616 -0.371835
H -6.149411 1.134163 -0.089883
C -4.861953 -0.444401 0.605019
C -4.849199 -0.014946 2.062080
C -3.251006 -3.191618 -1.598784
H -2.843131 -3.684383 -0.710199
H -4.829510 -0.920047 2.680895
H 2.217863 1.626676 -1.355361
H 0.785230 -2.323589 4.649929
H 1.714977 -3.910109 3.120608
H 1.144461 -4.013597 -2.534233
H 1.092336 -2.113123 -3.988869
C 1.702200 3.680599 -1.234220
H 1.255151 3.643628 -0.235520
H 2.128183 4.676079 -1.395945
H 0.902597 3.546164 -1.969689
C 3.358990 2.697126 -2.837212
H 3.865621 3.661402 -2.947771
H 4.088256 1.908422 -3.041486
H 2.572758 2.636694 -3.597028
C 5.849725 -0.744070 1.894108
H 5.743301 -0.286642 2.883590
H 4.846743 -0.990142 1.532514
H 6.416488 -1.673548 2.003737
C 7.991284 0.508387 1.467146
H 8.578736 -0.413671 1.456594
H 8.505203 1.250222 0.849191
H 7.972873 0.871904 2.499311
C -3.575989 0.791691 2.359205
H -3.532845 1.049460 3.422629
H -2.661258 0.250917 2.094499
H -3.580713 1.720981 1.777696
C -6.082897 0.792546 2.469584
H -6.087058 1.780477 1.997123
H -7.010118 0.277417 2.202650
H -6.075522 0.948680 3.551709
C -2.071395 -2.797219 -2.497896
H -2.426806 -2.357366 -3.436611
H -1.414458 -2.075727 -2.001797
H -1.480037 -3.684561 -2.749317
C -4.154889 -4.212944 -2.301572
H -4.993947 -4.504026 -1.663501
H -4.559777 -3.805524 -3.233217
H -3.581528 -5.110811 -2.550476
C 2.595432 -3.438152 -0.140961
H 2.763769 -2.726978 0.665183
H 3.542112 -3.920046 -0.396355
C 1.595282 -4.516899 0.265194
H 2.077402 -5.078141 1.072233
H 1.470760 -5.237041 -0.549710
Sc 0.546533 -0.630578 0.339643
H -6.036835 0.463238 -2.459445
O 0.364127 1.236191 1.110628
C -0.649136 1.928907 0.613365

C -1.226509 1.561691 -0.567226
N -1.069308 3.044467 1.351646
O -0.517340 0.500285 -1.216897
C -2.388443 2.153066 -1.213098
C -2.075515 3.871996 0.821130
C -0.864686 3.022368 2.794451
C -0.421373 0.594018 -2.647401
C -2.387779 2.140372 -2.724160
H -3.358220 2.031291 -0.728793
C -2.076366 4.115874 -0.595502
C -3.112102 4.366846 1.604013
H 0.078784 2.522851 3.002806
H -0.815437 4.050376 3.159232
H -1.670713 2.496708 3.323345
C -1.790951 0.832676 -3.258155
H 0.010417 -0.357973 -2.965551
H 0.267375 1.415266 -2.894884
H -1.774038 2.974428 -3.099408
H -3.400976 2.289108 -3.107090
C -3.085490 4.957507 -1.130316
H -1.124875 4.064536 -1.117248
C -4.151175 5.110574 1.024824
H -3.139348 4.158727 2.668505
H -1.686711 0.867050 -4.347494
H -2.445425 -0.008043 -3.004654
C -4.142817 5.395481 -0.345874
H -3.032880 5.229912 -2.180611
H -4.956857 5.474691 1.653938
H -4.936666 5.993376 -0.782101
133
TS-II-Feng-re-2 Eopt -3432.725118
O 2.628549 -0.317129 0.140222
O 0.904969 -1.987018 -1.113082
O 0.790156 -2.037443 1.838840
O -1.446171 -1.361065 0.459167
N 4.464457 0.167115 -1.053923
H 5.090446 -0.103074 -1.806971
N 2.089105 -2.675279 -1.301068
N -3.385800 -2.258531 1.123344
H -3.873420 -2.890376 1.750436
N 0.053519 -3.190617 2.017380
C 4.771812 1.352074 -0.287926
C 3.978748 2.493970 -0.479372
C 4.296339 3.621284 0.281834
H 3.709379 4.526660 0.173789
C 5.362519 3.601408 1.178047
H 5.595179 4.490150 1.755910
C 6.130438 2.452454 1.340182
H 6.955514 2.457045 2.043979
C 5.844009 1.293571 0.612612
C 6.608597 -0.001774 0.829909
H 6.676940 -0.522608 -0.132356
C 2.865540 2.516681 -1.513815
C 3.398489 -0.578088 -0.812775
C 3.162252 -1.726873 -1.776354
H 4.080864 -2.318137 -1.854628
C 2.667788 -1.308272 -3.171342
H 3.516665 -1.071152 -3.813465
H 2.037044 -0.418604 -3.097816
C 1.859929 -2.532566 -3.670943
H 2.298533 -2.972134 -4.566670
C 1.887694 -3.540217 -2.519710
H 2.733794 -4.228918 -2.577804
C 0.138073 -4.104711 0.823424
H -0.423147 -5.002731 1.093877
H -0.373915 -3.592810 0.007471
C 0.527520 -3.794681 3.316644
H 0.134531 -4.812461 3.366704
C -0.072396 -2.868300 4.384741
H -0.399427 -3.456045 5.242632
C -1.260622 -2.143482 3.700418
H -1.036992 -1.086764 3.539380
H -2.192410 -2.223421 4.261151
C -1.387166 -2.865424 2.356400
H -1.882280 -3.832100 2.489537
C -2.074503 -2.104195 1.241607
C -4.107864 -1.576401 0.075795
C -4.028084 -2.084897 -1.227807
C -4.651315 -1.345222 -2.239235
H -4.607235 -1.699246 -3.265487
C -5.328884 -0.166360 -1.948518
C -5.422340 0.290003 -0.634501
H -5.976938 1.198507 -0.424644
C -4.814408 -0.409529 0.410014
C -4.898107 0.061007 1.853403
C -3.336898 -3.397590 -1.562752
H -3.007240 -3.863641 -0.628220
H -4.935743 -0.832570 2.488059
H 2.366179 1.541378 -1.505355
H 0.667172 -2.144384 4.725011
H 1.615378 -3.807439 3.288776
H 0.958403 -4.089648 -2.379057
H 0.832014 -2.246033 -3.897292
C 1.777578 3.546575 -1.215150
H 1.374283 3.422840 -0.204270
H 2.140805 4.573172 -1.326985
H 0.958802 3.408795 -1.927821
C 3.449615 2.739260 -2.916960
H 3.931394 3.721001 -2.972540
H 4.196602 1.980983 -3.171943
H 2.654705 2.702471 -3.668665
C 5.830419 -0.905530 1.800108
H 5.768153 -0.429345 2.784455
H 4.808020 -1.089861 1.456158
H 6.336779 -1.868649 1.914648
C 8.037768 0.210694 1.332211
H 8.569062 -0.744888 1.335812
H 8.589344 0.907930 0.695116
H 8.050315 0.595103 2.356872
C -3.652680 0.864732 2.252246
H -3.693285 1.104949 3.319784
H -2.720097 0.327411 2.051688
H -3.611966 1.804771 1.688677
C -6.157742 0.880107 2.144561
H -6.121338 1.856711 1.650611
H -7.062962 0.360675 1.817441
H -6.234450 1.061551 3.220047
C -2.098128 -3.191494 -2.445136
H -2.363970 -2.689976 -3.382929
H -1.334488 -2.591171 -1.941039
H -1.659967 -4.163285 -2.698066
C -4.319107 -4.371031 -2.229113
H -5.210810 -4.517453 -1.613237
H -4.637997 -4.000332 -3.208370
H -3.838677 -5.342231 -2.379389
C 2.526967 -3.437961 -0.076145
H 2.722852 -2.697320 0.696546
H 3.466118 -3.924107 -0.351001
C 1.551106 -4.504918 0.412982
H 2.054026 -5.006290 1.246397
H 1.428342 -5.275722 -0.354758
Sc 0.540687 -0.593402 0.405450
H -5.802565 0.395182 -2.747940
O 0.213334 1.175132 1.333837
C -0.736351 1.936640 0.815967
C -1.274171 1.632133 -0.406313
N -1.108606 3.078865 1.527764
O -0.461444 0.676729 -1.100449
C -2.316172 2.381162 -1.079563
C -1.037279 3.037572 2.982122
C -1.948936 4.023898 0.907547
C -1.026788 0.225971 -2.343482
C -2.467337 2.225980 -2.581235
H -3.273860 2.418012 -0.558498
H -0.211836 2.388021 3.266650
H -1.962866 2.658508 3.434087
H -0.848748 4.045333 3.358802
C -2.982707 4.639856 1.594278
C -1.803490 4.232742 -0.521276
C -1.318263 1.434566 -3.210454
H -0.285492 -0.451052 -2.771801
H -1.946203 -0.333480 -2.119660
H -3.405238 1.691533 -2.778290
H -2.571403 3.209469 -3.054403
C -3.877389 5.497731 0.929267
H -3.125899 4.444587 2.651902
C -2.649965 5.209807 -1.124766
H -0.808427 4.117920 -0.940828
H -1.584183 1.110735 -4.220860
H -0.405003 2.037489 -3.285474
C -3.712007 5.772938 -0.434085
H -4.685433 5.958410 1.487735
H -2.468973 5.483463 -2.160225
H -4.383650 6.463825 -0.934128
133
TS-II-Feng-si-1 Eopt -3432.727566
O 2.537343 -0.347410 0.184014
O 0.748807 -1.981363 -1.091841
O 0.697180 -2.230811 1.828613
O -1.527694 -1.390981 0.499446
N 4.269811 0.252445 -1.118230
H 4.813377 0.038575 -1.949413
N 1.933502 -2.619305 -1.392584
N -3.481412 -2.301814 1.102884
H -3.977852 -2.984524 1.666971
N -0.044615 -3.390876 1.904281
C 4.792888 1.267069 -0.233333
C 4.101013 2.478093 -0.072867
C 4.681726 3.422278 0.780316
H 4.187674 4.371849 0.947090
C 5.891010 3.169906 1.421800
H 6.321165 3.924503 2.072559
C 6.549676 1.958970 1.237927
H 7.488852 1.778131 1.748703
C 6.007884 0.974639 0.407947
C 6.662864 -0.389982 0.258602
H 6.519886 -0.733759 -0.772753
C 2.800689 2.766205 -0.811660
C 3.228266 -0.516282 -0.847458
C 2.943320 -1.603461 -1.870558
H 3.869281 -2.158242 -2.059043
C 2.327818 -1.104694 -3.186044
H 3.106695 -0.739151 -3.855883
H 1.634958 -0.284392 -2.979060
C 1.587311 -2.344613 -3.745141
H 2.046544 -2.708945 -4.664176
C 1.684513 -3.413690 -2.651433
H 2.534778 -4.085513 -2.790041
C 0.066171 -4.219238 0.651128
H -0.468598 -5.148249 0.862908
H -0.462881 -3.670364 -0.127958
C 0.402607 -4.094913 3.162803
H -0.002026 -5.108880 3.131514
C -0.204748 -3.245664 4.286330
H -0.523225 -3.888615 5.106972
C -1.400736 -2.494154 3.647358
H -1.207452 -1.420763 3.589945
H -2.338471 -2.651089 4.181429
C -1.491408 -3.088122 2.236644
H -1.996181 -4.058743 2.260468
C -2.163788 -2.201944 1.206052

C -4.207819 -1.442262 0.197185
C -4.315875 -1.830413 -1.145565
C -4.901083 -0.915216 -2.027365
H -4.991403 -1.166878 -3.079890
C -5.368565 0.314758 -1.573868
C -5.295761 0.644014 -0.221728
H -5.692909 1.595821 0.117750
C -4.715245 -0.232841 0.698527
C -4.618550 0.110902 2.176601
C -3.827691 -3.182798 -1.642074
H -3.654267 -3.821852 -0.769869
H -4.558986 -0.831017 2.733083
H 2.152520 1.884996 -0.717267
H 0.527363 -2.535466 4.668967
H 1.490592 -4.118064 3.150417
H 0.770288 -3.984651 -2.497361
H 0.544660 -2.105573 -3.959103
C 2.033025 3.947658 -0.216146
H 1.849034 3.806163 0.853352
H 2.574543 4.888903 -0.356100
H 1.066051 4.042808 -0.714399
C 3.053551 3.013118 -2.306998
H 3.653591 3.918916 -2.444249
H 3.580260 2.181146 -2.782271
H 2.098965 3.151315 -2.826101
C 5.972315 -1.398714 1.191162
H 6.139832 -1.114756 2.235459
H 4.890295 -1.432767 1.029815
H 6.377329 -2.403401 1.037360
C 8.170637 -0.377190 0.516830
H 8.591708 -1.353528 0.262296
H 8.676133 0.382465 -0.086103
H 8.396548 -0.188920 1.570922
C -3.344255 0.915201 2.471645
H -3.216489 1.041926 3.551872
H -2.445029 0.436626 2.069668
H -3.412204 1.911120 2.020724
C -5.850140 0.863799 2.688307
H -5.905865 1.872672 2.267439
H -6.775836 0.335756 2.442450
H -5.790082 0.967221 3.775381
C -2.498740 -3.059887 -2.401292
H -2.613203 -2.402760 -3.272100
H -1.707113 -2.645486 -1.769391
H -2.178480 -4.044431 -2.758073
C -4.880846 -3.877959 -2.513452
H -5.845419 -3.936077 -2.001589
H -5.027149 -3.350567 -3.461137
H -4.552905 -4.894459 -2.749022
C 2.437843 -3.447337 -0.239285
H 2.649300 -2.752936 0.571377
H 3.373997 -3.900248 -0.575520
C 1.490180 -4.557141 0.215340
H 2.013550 -5.081626 1.021386
H 1.380793 -5.296986 -0.584140
Sc 0.466517 -0.670722 0.517907
H -5.810471 1.016155 -2.275221
O 0.035868 1.082048 1.400957
C -0.581707 2.023574 0.702452
C -0.725154 1.904638 -0.647908
N -1.072201 3.124833 1.411113
O -0.283963 0.636416 -1.141359
C -1.359266 2.866188 -1.537496
C -0.406401 3.529211 2.639451
C -1.970557 4.005495 0.794012
C -1.126849 0.089833 -2.180407
C -2.046999 2.319351 -2.766963
H -0.885341 3.841474 -1.650890
H 0.116911 2.666610 3.046901
H -1.153907 3.870646 3.360879
H 0.315394 4.339130 2.472028
C -2.836795 3.491370 -0.236886
C -1.981457 5.364924 1.085857
C -1.307912 1.090393 -3.304078
H -0.624427 -0.821049 -2.504215
H -2.093904 -0.172124 -1.726448
H -3.066984 1.999077 -2.501792
H -2.141750 3.096039 -3.529334
C -3.750056 4.388720 -0.849258
H -3.121915 2.442489 -0.190330
C -2.833562 6.237084 0.390970
H -1.314469 5.766976 1.840275
H -1.882240 0.611662 -4.104683
H -0.328210 1.368065 -3.711995
C -3.708514 5.751012 -0.587272
H -4.474729 3.987858 -1.553646
H -2.813398 7.295777 0.627924
H -4.378844 6.429624 -1.104911
133
TS-II-Feng-si-2 Eopt -3432.728458
O -2.618671 -0.283417 -0.181831
O -1.002369 -1.934299 1.231622
O -0.753074 -2.199172 -1.728592
O 1.453163 -1.415219 -0.337279
N -4.496953 0.281871 0.910932
H -5.168875 0.032415 1.630978
N -2.201849 -2.601709 1.398774
N 3.414040 -2.276649 -0.999722
H 3.902803 -2.935688 -1.597399
N -0.021745 -3.367860 -1.790153
C -4.772327 1.442968 0.096748
C -4.025116 2.613723 0.311849
C -4.325649 3.709842 -0.501039
H -3.773904 4.635179 -0.383651
C -5.330746 3.636979 -1.463044
H -5.547832 4.504160 -2.078538
C -6.058778 2.465570 -1.639583
H -6.840107 2.429872 -2.390775
C -5.789434 1.335316 -0.862020
C -6.522011 0.021242 -1.080852
H -6.625524 -0.477328 -0.110009
C -2.968674 2.693258 1.406506
C -3.440343 -0.495900 0.738362
C -3.297326 -1.623966 1.747116
H -4.226129 -2.203448 1.757689
C -2.934276 -1.156355 3.167448
H -3.842364 -0.943532 3.732587
H -2.340607 -0.240803 3.123654
C -2.122973 -2.329140 3.769351
H -2.590781 -2.729852 4.668468
C -2.077178 -3.395710 2.676039
H -2.926642 -4.081537 2.719054
C -0.153488 -4.177460 -0.528025
H 0.416299 -5.093812 -0.699729
H 0.325537 -3.597841 0.262538
C -0.463276 -4.080495 -3.045792
H -0.075145 -5.100150 -2.994762
C 0.171669 -3.255913 -4.173731
H 0.511460 -3.918185 -4.970123
C 1.350593 -2.487405 -3.524322
H 1.142385 -1.416800 -3.472577
H 2.296945 -2.634814 -4.045983
C 1.427090 -3.076164 -2.111277
H 1.926323 -4.050036 -2.128797
C 2.094345 -2.194740 -1.074353
C 4.176297 -1.437325 -0.108913
C 4.145040 -1.705929 1.265580
C 4.854724 -0.833027 2.098779
H 4.847054 -0.995311 3.173062
C 5.575890 0.232099 1.571470
C 5.613290 0.449598 0.194501
H 6.188083 1.281716 -0.197753
C 4.903007 -0.376609 -0.677001
C 4.861829 -0.132807 -2.177270
C 3.408723 -2.896151 1.859241
H 3.029081 -3.512153 1.037339
H 4.843958 -1.110181 -2.674392
H -2.327346 1.803148 1.318804
H -0.551192 -2.556630 -4.592383
H -1.551481 -4.086836 -3.048899
H -1.141241 -3.949590 2.624505
H -1.113200 -2.005548 4.025319
C -2.054070 3.913351 1.280675
H -1.570083 3.964051 0.300839
H -2.608576 4.841485 1.454910
H -1.264906 3.851104 2.036074
C -3.623264 2.699743 2.798050
H -4.230629 3.602855 2.917591
H -4.271041 1.836105 2.966811
H -2.854051 2.702655 3.577284
C -5.689760 -0.894261 -1.992989
H -5.589236 -0.440907 -2.984923
H -4.681951 -1.056278 -1.599244
H -6.178673 -1.866481 -2.106537
C -7.931363 0.197886 -1.649422
H -8.447927 -0.765764 -1.648613
H -8.519832 0.903573 -1.056163
H -7.905836 0.553589 -2.684094
C 3.577811 0.620808 -2.562159
H 3.450870 0.629761 -3.649625
H 2.678467 0.186179 -2.111053
H 3.639529 1.657762 -2.216530
C 6.085224 0.619695 -2.703073
H 6.101549 1.653486 -2.342350
H 7.018600 0.133895 -2.404356
H 6.050246 0.655783 -3.795381
C 2.207703 -2.454201 2.705355
H 2.530957 -1.839947 3.553620
H 1.498993 -1.876541 2.105835
H 1.684717 -3.330666 3.103025
C 4.358706 -3.777553 2.680947
H 5.222280 -4.090294 2.087369
H 4.726065 -3.246021 3.564356
H 3.832797 -4.672920 3.025141
C -2.578650 -3.442367 0.204996
H -2.728909 -2.754720 -0.625384
H -3.534641 -3.906067 0.459290
C -1.584868 -4.544170 -0.151380
H -2.050143 -5.112869 -0.963002
H -1.501224 -5.250295 0.680749
Sc -0.542540 -0.675535 -0.380025
H 6.123184 0.893777 2.236017
O -0.069042 0.988400 -1.403143
C 0.583040 1.941111 -0.754983
C 0.615592 1.968218 0.612322
N 1.301119 2.873530 -1.506446
O 0.165263 0.728741 1.169463
C 1.411332 2.905195 1.374655
C 0.705263 3.375975 -2.734590
C 2.400864 3.526546 -0.939850
C 0.243719 0.633246 2.602024
C 1.759636 2.570639 2.806174
H 1.174546 3.955267 1.206845
H -0.017221 2.640970 -3.085937
H 0.196334 4.335896 -2.577490
H 1.479463 3.504342 -3.496002
C 2.802714 4.783495 -1.370370
C 3.051965 2.911353 0.203360
C 1.618385 1.073553 3.067248
H 0.021245 -0.409395 2.832181
H -0.533953 1.282284 3.032316
H 1.084354 3.115430 3.480855

H 2.771734 2.923807 3.034301
C 3.846175 5.465922 -0.720488
H 2.294964 5.264184 -2.199002
C 4.167419 3.598369 0.763709
H 3.084806 1.821239 0.239873
H 1.737537 0.843662 4.130242
H 2.377516 0.504739 2.513490
C 4.516589 4.878726 0.358572
H 4.131166 6.452421 -1.071055
H 4.737544 3.100983 1.543694
H 5.334319 5.402462 0.843441
35
TS-II-ScCl3-con-1 Eopt -
2850.948871
O 0.690856 -1.356363 -0.279464
C -0.501417 -0.823300 -0.060887
C -0.620526 0.482417 0.295378
N -1.611866 -1.677597 -0.222201
O 0.630284 1.157669 0.280047
C -1.858822 1.204034 0.552658
C -1.460106 -3.025619 0.311411
C 0.605913 2.540600 -0.123464
C -1.856256 2.677405 0.224552
H -2.443055 0.909967 1.423859
H -0.437085 -3.349953 0.132092
H -2.144896 -3.700802 -0.205994
H -1.666017 -3.065171 1.390740
C -0.501344 3.296406 0.587422
H 1.595324 2.929977 0.120555
H 0.468785 2.576574 -1.212063
H -2.014399 2.816965 -0.855927
H -2.676479 3.184914 0.737959
H -0.457049 4.345124 0.276152
H -0.336279 3.259183 1.670073
C -2.891850 -1.105602 -0.243546
C -3.042274 0.199752 -0.835825
C -3.985979 -1.704250 0.371758
C -4.341330 0.764954 -0.894673
H -2.322245 0.500045 -1.592753
C -5.236296 -1.064436 0.388544
H -3.881041 -2.665157 0.861936
C -5.412341 0.173855 -0.236992
H -4.477692 1.696015 -1.437542
H -6.070878 -1.549427 0.884701
H -6.388382 0.648947 -0.242243
Sc 2.358216 -0.277206 -0.032836
Cl 3.787774 -2.181440 -0.505981
Cl 3.124916 0.327705 2.178180
Cl 3.149578 1.201943 -1.773920
35
TS-II-ScCl3-con-2 Eopt -
2850.951703
O 0.595319 1.100400 0.197378
C -0.473418 -0.919174 0.250325
C -0.576213 0.403324 0.591344
C 0.629083 2.495936 0.531107
N -1.612741 -1.706983 0.064161
C -1.838237 1.089132 0.754181
C -0.623879 3.163336 -0.007527
H 1.541030 2.880360 0.072211
H 0.698868 2.589649 1.623110
C -2.839346 -1.087173 -0.206409
C -1.579309 -3.079067 0.547171
C -1.845860 2.601097 0.718391
H -2.489787 0.670986 1.519288
H -0.553346 4.245406 0.136086
H -0.683966 2.973064 -1.085902
C -2.820642 0.255269 -0.771979
C -4.045709 -1.658153 0.171028
H -0.542045 -3.411081 0.546587
H -1.985742 -3.162340 1.563955
H -2.157719 -3.722296 -0.120530
H -1.845894 2.975294 1.751305
H -2.778517 2.955235 0.264154
C -4.082836 0.864200 -1.041425
H -2.009872 0.494623 -1.458512
C -5.259070 -0.969764 -0.014947
H -4.062442 -2.636802 0.637601
C -5.273024 0.298346 -0.607902
H -4.093066 1.810124 -1.575849
H -6.186230 -1.438768 0.298162
H -6.214733 0.813710 -0.770240
O 0.703767 -1.447203 -0.031199
Sc 2.337981 -0.279033 -0.132532
Cl 3.814705 -2.129304 -0.666327
Cl 3.104842 0.355192 2.056005
Cl 3.017695 1.291875 -1.853028
35
TS-II-ScCl3-con-3 Eopt -
2850.948684
O 0.721034 -1.314332 -0.352939
C -0.475172 -0.802610 -0.106214
C -0.605318 0.490295 0.292853
N -1.577807 -1.660507 -0.290135
O 0.633755 1.186332 0.266597
C -1.853798 1.180371 0.582443
C -1.413843 -3.027844 0.187133
C 0.575522 2.583246 -0.081206
C -1.879383 2.667713 0.324791
H -2.429201 0.835571 1.440625
H -0.384314 -3.330429 0.006905
H -2.082595 -3.689622 -0.367416
H -1.632374 -3.116170 1.260864
C -0.526264 3.289968 0.686159
H 1.563196 2.980664 0.153022
H 0.409984 2.659724 -1.163951
H -2.064547 2.856210 -0.743801
H -2.695391 3.137049 0.879512
H -0.503475 4.352149 0.421902
H -0.336229 3.206460 1.762149
C -2.863707 -1.102800 -0.304176
C -3.023403 0.225391 -0.843104
C -3.957143 -1.740245 0.271798
C -4.329076 0.777854 -0.888926
H -2.304915 0.561506 -1.586393
C -5.215348 -1.116281 0.302777
H -3.845898 -2.720443 0.720595
C -5.399424 0.146897 -0.268467
H -4.471494 1.729774 -1.392750
H -6.049274 -1.632631 0.767349
H -6.380651 0.611179 -0.262531
Sc 2.372275 -0.236004 -0.055243
Cl 3.845986 -2.076632 -0.633775
Cl 2.856759 -0.059934 2.297882
Cl 3.261602 1.414854 -1.602311
35
TS-II-ScCl3-con-4 Eopt -
2850.949761
O -0.676762 -1.426155 -0.017847
C 0.509294 -0.853273 -0.139040
C 0.608591 0.476003 -0.405095
N 1.634431 -1.685000 0.026148
O -0.659313 1.120455 -0.391087
C 1.833593 1.238849 -0.590482
C 1.535537 -3.025090 -0.536813
C -0.669890 2.465888 0.124127
C 1.787589 2.696333 -0.195971
H 2.449902 0.999031 -1.456330
H 0.515351 -3.377688 -0.400211
H 2.218591 -3.692062 -0.006493
H 1.780726 -3.040854 -1.608366
C 0.417229 3.298694 -0.530016
H -1.671141 2.845603 -0.088487
H -0.524162 2.414813 1.211150
H 1.941347 2.789961 0.889962
H 2.594571 3.248957 -0.683262
H 0.343053 4.324435 -0.154844
H 0.257398 3.324175 -1.613859
C 2.899167 -1.086653 0.122913
C 2.998993 0.195318 0.776256
C 4.027637 -1.637166 -0.474420
C 4.283113 0.784899 0.901235
H 2.251460 0.442929 1.525767
C 5.263317 -0.971477 -0.421523
H 3.960809 -2.580084 -1.004762
C 5.388707 0.245015 0.257149
H 4.380641 1.695324 1.485941
H 6.125505 -1.418918 -0.905594
H 6.352502 0.741245 0.314511
Sc -2.354362 -0.327837 -0.021645
Cl -2.515501 0.481931 2.233596
Cl -3.830962 -2.250323 -0.003444
Cl -3.629104 1.147467 -1.482626
35
TS-II-ScCl3-con-5 Eopt -
2850.951570
O -0.606439 1.065189 -0.272389
C 0.492203 -0.935092 -0.318606
C 0.586804 0.392571 -0.639318
C -0.650060 2.476282 -0.525725
N 1.636938 -1.716104 -0.128478
C 1.840619 1.101144 -0.767399
C 0.590795 3.126178 0.059250
H -1.570162 2.824406 -0.053934
H -0.714076 2.633185 -1.610554
C 2.854479 -1.085332 0.157929
C 1.625824 -3.074737 -0.649100
C 1.823509 2.610901 -0.682538
H 2.511792 0.716149 -1.532953
H 0.510798 4.213280 -0.031137
H 0.643256 2.882609 1.127373
C 2.815057 0.243245 0.753055
C 4.070315 -1.628898 -0.229666
H 0.593959 -3.423218 -0.658991
H 2.034725 -3.124120 -1.667310
H 2.213688 -3.726422 0.001867
H 1.819563 3.020812 -1.701913
H 2.749118 2.964072 -0.213516
C 4.066443 0.863221 1.044710
H 1.994583 0.457940 1.436102
C 5.272552 -0.926595 -0.023874
H 4.103384 -2.595617 -0.719607
C 5.266286 0.325936 0.601218
H 4.060530 1.795583 1.602470
H 6.207459 -1.373252 -0.346496
H 6.199813 0.850814 0.779569
O -0.684916 -1.478492 -0.060458
Sc -2.321784 -0.317014 0.100340
Cl -3.842539 -2.156549 0.520885
Cl -3.363222 0.687004 -1.837349
Cl -2.774100 1.053503 2.043782
35
TS-II-ScCl3-con-6 Eopt -
2850.951641
O 0.600705 1.118099 0.139673
C -0.460127 -0.909395 0.106728
C -0.566423 0.396558 0.504396
C 0.622108 2.499859 0.530630
N -1.596437 -1.703918 -0.081235
C -1.828899 1.067238 0.726214
C -0.638916 3.175750 0.023158
H 1.529569 2.909226 0.085655
H 0.692263 2.548418 1.625657

C -2.838093 -1.085171 -0.275868
C -1.537430 -3.083608 0.378623
C -1.849956 2.579684 0.739793
H -2.457175 0.619503 1.494008
H -0.575181 4.252979 0.201794
H -0.705605 3.020742 -1.060453
C -2.852529 0.274543 -0.797257
C -4.023191 -1.674831 0.138741
H -0.500046 -3.411324 0.329214
H -1.899867 -3.183922 1.410635
H -2.141628 -3.718623 -0.273716
H -1.844516 2.920621 1.784004
H -2.789494 2.940179 0.305001
C -4.128392 0.881451 -0.996471
H -2.068719 0.545536 -1.502732
C -5.247033 -0.988751 0.028876
H -4.014390 -2.666562 0.576894
C -5.294825 0.295296 -0.526786
H -4.167426 1.842411 -1.501824
H -6.156430 -1.471930 0.371183
H -6.245766 0.808512 -0.632291
O 0.715090 -1.418354 -0.216926
Sc 2.351349 -0.252552 -0.133607
Cl 3.190499 1.466309 -1.649011
Cl 2.841998 0.051027 2.201320
Cl 3.877915 -2.040538 -0.711442
35
TS-II-ScCl3-dis-1 Eopt -2850.939313
O 0.662366 -1.185184 -0.486015
C -0.485847 0.794863 -0.699324
C -0.603530 -0.558143 -0.747300
C 0.555672 -2.522088 0.054756
N -1.576907 1.691298 -0.673616
C -1.753373 -1.435936 -0.913769
C -0.637973 -2.606131 0.992898
H 1.503833 -2.710829 0.560280
H 0.453562 -3.220087 -0.785522
C -2.812333 1.111212 -0.319721
C -1.275592 3.016235 -0.137946
C -1.942492 -2.454082 0.188580
H -1.985198 -1.780873 -1.920894
H -0.614308 -3.559436 1.529107
H -0.540652 -1.806657 1.735820
C -3.289336 0.059808 -1.152510
C -3.500412 1.452512 0.841675
H -0.452617 3.450454 -0.702256
H -2.163592 3.639135 -0.254763
H -0.988652 2.986940 0.921981
H -2.237825 -3.419632 -0.236550
H -2.760771 -2.137625 0.854223
C -4.531429 -0.547352 -0.827351
C -4.692630 0.797065 1.173462
H -3.112779 2.225629 1.496899
C -5.200046 -0.208068 0.337971
H -4.944505 -1.292792 -1.500508
H -5.220705 1.071968 2.080587
H -6.136381 -0.697894 0.587760
O 0.714912 1.340279 -0.584389
H -2.961777 0.075042 -2.187762
Sc 2.310840 0.237733 -0.074897
Cl 2.051740 -0.109214 2.287791
Cl 3.778396 -1.537784 -0.886313
Cl 3.856380 2.100235 -0.229450
35
TS-II-ScCl3-dis-2 Eopt -2850.940578
O -0.688883 -1.368363 -0.383924
C 0.505835 -0.822036 -0.529213
C 0.634160 0.529654 -0.564154
N 1.594431 -1.717447 -0.587852
O -0.602766 1.198295 -0.277136
C 1.763259 1.392266 -0.895118
C 1.323707 -3.058082 -0.074197
C -0.440006 2.250932 0.702269
C 1.989523 2.509531 0.086280
H 1.870431 1.680456 -1.943423
H 0.480503 -3.483659 -0.615317
H 1.080901 -3.060629 0.996965
H 2.209743 -3.671431 -0.244603
C 0.645512 3.234905 0.289660
H -1.418508 2.730415 0.778243
H -0.191923 1.777347 1.660366
H 2.325627 2.094480 1.047455
H 2.755057 3.206745 -0.267357
H 0.723936 4.000917 1.068920
H 0.349942 3.734451 -0.640158
C 2.846962 -1.139079 -0.274019
C 3.320309 -0.112858 -1.137309
C 3.552137 -1.460807 0.881238
C 4.567953 0.495883 -0.846031
C 4.753678 -0.806107 1.180023
H 3.170103 -2.218805 1.557425
C 5.253398 0.177654 0.316859
H 4.975069 1.222765 -1.542513
H 5.296048 -1.067489 2.082748
H 6.196264 0.666846 0.541664
H 2.964746 -0.132113 -2.162626
Sc -2.327533 -0.243457 -0.096019
Cl -2.434443 -0.031419 2.290157
Cl -3.676669 1.637697 -0.877222
Cl -3.832189 -2.084876 -0.572682
35
TS-II-ScCl3-dis-3 Eopt -2850.937883
O 0.596587 1.195527 0.248763
C -0.477452 -0.814740 0.497902
C -0.625433 0.533583 0.608722
C 0.450030 2.607045 0.007513
N -1.544731 -1.734864 0.509621
C -1.785652 1.360951 0.896278
C -0.768154 2.829964 -0.874033
H 1.379615 2.918203 -0.468675
H 0.343575 3.113789 0.975046
C -2.805242 -1.184841 0.194686
C -1.233662 -3.066017 -0.002716
C -2.048772 2.481036 -0.087347
H -1.981177 1.605659 1.939163
H -0.792372 3.866081 -1.223946
H -0.663266 2.187587 -1.755790
C -3.290443 -0.160520 1.057946
C -3.503835 -1.525713 -0.959438
H -0.386946 -3.471267 0.548831
H -2.106355 -3.701164 0.155867
H -0.976456 -3.060193 -1.069977
H -2.413667 3.364946 0.446087
H -2.846182 2.181953 -0.784820
C -4.549076 0.429721 0.754084
C -4.714175 -0.891049 -1.264317
H -3.107709 -2.281284 -1.630357
C -5.225860 0.094503 -0.406728
H -4.969128 1.156280 1.443498
H -5.251632 -1.164270 -2.166338
H -6.174463 0.570546 -0.636471
O 0.722033 -1.318582 0.256802
H -2.970577 -0.215422 2.094050
Sc 2.329584 -0.164677 -0.035248
Cl 3.741266 -2.074661 -0.558451
Cl 2.905550 1.198643 -1.976097
Cl 3.219419 0.484943 2.106716
35
TS-II-ScCl3-dis-4 Eopt -2850.940632
O 0.736505 -1.289349 -0.318849
C -0.436200 -0.805109 0.059434
C -0.564119 0.511767 0.370533
N -1.500149 -1.729632 0.075762
O 0.610077 1.277237 0.056317
C -1.706430 1.302515 0.827565
C -1.274536 -2.964614 -0.670277
C 0.304696 2.281680 -0.943782
C -2.090637 2.401596 -0.123859
H -1.742922 1.560428 1.888114
H -0.365478 -3.440269 -0.306861
H -1.166860 -2.795575 -1.750158
H -2.123730 -3.626475 -0.493271
C -0.817732 3.197715 -0.472204
H 1.230955 2.831530 -1.107581
H 0.023162 1.756119 -1.866165
H -2.502582 1.956213 -1.041623
H -2.851842 3.061840 0.303063
H -1.015437 3.929915 -1.262934
H -0.476905 3.748862 0.411892
C -2.793469 -1.160844 0.025920
C -3.168728 -0.305502 1.096482
C -3.647074 -1.335903 -1.059400
C -4.457953 0.279787 1.078287
C -4.895577 -0.702314 -1.084225
H -3.344912 -1.963122 -1.891880
C -5.293912 0.111378 -0.016732
H -4.780611 0.871663 1.929576
H -5.553163 -0.848424 -1.934990
H -6.271555 0.583386 -0.030822
H -2.653812 -0.438096 2.043156
Sc 2.358095 -0.160274 0.014582
Cl 3.859954 -2.009406 -0.473764
Cl 3.591565 1.530681 -1.239078
Cl 2.481075 -0.020736 2.409769
35
TS-III-ScCl3-con-1 Eopt -2850.813315
O -0.575573 1.147328 0.125871
C 0.536610 -0.860234 -0.150351
C 0.654416 0.529988 -0.000067
C -0.672325 2.384155 -0.640086
N 1.676463 -1.593942 -0.364203
C 1.827037 1.281641 -0.564730
C 0.408728 3.374266 -0.227927
H -1.674391 2.760939 -0.436745
H -0.603750 2.109359 -1.696670
C 2.887446 -0.999301 -0.144920
C 1.555295 -3.045854 -0.546311
C 1.788373 2.709819 -0.013062
H 1.876852 1.276988 -1.663601
H 0.459022 4.131403 -1.014966
H 0.105062 3.883179 0.689600
C 2.904886 0.427103 0.109826
C 4.062177 -1.768760 0.013700
H 0.529475 -3.261428 -0.830884
H 2.231524 -3.361806 -1.339586
H 1.793360 -3.563015 0.385882
H 2.580318 3.309145 -0.467777
H 1.992133 2.659205 1.062805
C 4.170175 1.009455 0.484756
H 1.937866 0.378544 1.047999
C 5.223998 -1.157804 0.421536
H 4.045081 -2.837445 -0.157070
C 5.279663 0.237259 0.676531
H 4.222108 2.083599 0.623082
H 6.115488 -1.760686 0.558345
H 6.214374 0.688616 0.989375
O -0.603195 -1.442443 -0.033452

Sc -2.356453 -0.358541 0.204273
Cl -3.367805 1.365163 1.535104
Cl -3.668099 -2.293715 0.699277
Cl -2.870153 0.212133 -2.042782
35
TS-III-ScCl3-con-2 Eopt -2850.824011
O -0.609779 -1.442535 -0.183707
C 0.538289 -0.869529 -0.231870
C 0.656341 0.522969 -0.078617
N 1.677980 -1.609372 -0.416629
O -0.590757 1.106744 -0.047012
C 1.833345 1.234811 -0.690243
C 1.554420 -3.061875 -0.594885
C -0.615288 2.518763 0.291105
C 1.811410 2.707811 -0.285439
H 1.870913 1.144612 -1.786667
H 0.534706 -3.274545 -0.902455
H 1.767973 -3.574835 0.345438
H 2.248374 -3.384271 -1.369995
C 0.411282 3.274473 -0.532601
H -1.637213 2.836123 0.083833
H -0.415043 2.596154 1.365710
H 2.047068 2.801170 0.783407
H 2.566066 3.265015 -0.846386
H 0.369440 4.329178 -0.247464
H 0.148231 3.206690 -1.594410
C 2.888503 -1.018732 -0.183262
C 2.905869 0.412760 0.034636
C 4.057739 -1.788072 0.004941
C 4.172534 1.004806 0.388207
H 1.952934 0.405782 0.977359
C 5.219996 -1.166726 0.397239
H 4.038409 -2.861250 -0.135030
C 5.280140 0.235967 0.604788
H 4.226691 2.083337 0.488931
H 6.109806 -1.767140 0.554583
H 6.216906 0.694209 0.900891
Sc -2.361099 -0.353155 0.062061
Cl -3.702608 -2.328644 0.045521
Cl -3.350707 1.028045 -1.614638
Cl -2.881463 0.643153 2.163065
35
TS-III-ScCl3-con-3 Eopt -2850.812421
O -0.656614 1.256885 -0.244851
C 0.450399 -0.738161 -0.684982
C 0.564694 0.612890 -0.331530
C -0.660740 2.510884 -0.991575
N 1.607804 -1.464293 -0.840370
C 1.810364 1.391629 -0.656504
C 0.392577 3.474679 -0.447244
H -1.671608 2.899134 -0.887987
H -0.478792 2.255811 -2.039917
C 2.777675 -0.937687 -0.368582
C 1.501766 -2.878296 -1.221183
C 1.684008 2.760810 0.008643
H 2.013344 1.488964 -1.733357
H 0.609188 4.191839 -1.243956
H -0.022357 4.038433 0.390950
C 2.771573 0.446473 0.065342
C 3.908174 -1.752604 -0.132936
H 0.530830 -3.025976 -1.685336
H 2.290746 -3.114204 -1.933896
H 1.584183 -3.513945 -0.336703
H 2.558935 3.377884 -0.208294
H 1.647365 2.608519 1.094149
C 3.967965 0.944828 0.699574
H 1.696973 0.303945 0.846784
C 4.995619 -1.228908 0.525004
H 3.910484 -2.790838 -0.438988
C 5.024388 0.121775 0.962650
H 4.002800 1.991717 0.980951
H 5.848784 -1.869607 0.721687
H 5.901925 0.500858 1.473962
O -0.699551 -1.304604 -0.788993
Sc -2.326019 -0.335071 0.071228
Cl -3.790133 -2.203421 -0.205249
Cl -3.866278 1.499251 -0.173872
Cl -1.585501 -0.474895 2.326030
35
TS-III-ScCl3-con-4 Eopt -2850.822756
O 0.614963 -1.421350 0.039068
C -0.534534 -0.855319 0.124722
C -0.664635 0.539450 0.005177
N -1.666142 -1.607141 0.315859
O 0.576364 1.136828 -0.044559
C -1.842165 1.222065 0.651772
C -1.528901 -3.060438 0.476170
C 0.578101 2.566636 -0.310454
C -1.843487 2.705677 0.292927
H -1.864390 1.099438 1.745453
H -0.505358 -3.268294 0.774207
H -1.745802 -3.565203 -0.467758
H -2.214353 -3.396523 1.253108
C -0.448537 3.275103 0.552710
H 1.596322 2.889035 -0.100470
H 0.368907 2.694804 -1.378443
H -2.085438 2.830600 -0.771366
H -2.601819 3.234929 0.875468
H -0.416034 4.340765 0.309868
H -0.177184 3.166915 1.609059
C -2.884948 -1.024332 0.108494
C -2.915759 0.410368 -0.081384
C -4.050715 -1.799047 -0.079601
C -4.190171 1.000125 -0.410731
H -1.975556 0.432338 -1.034540
C -5.221238 -1.178999 -0.448977
H -4.022471 -2.874353 0.041287
C -5.293670 0.226793 -0.631452
H -4.252964 2.079841 -0.491734
H -6.108469 -1.782747 -0.608103
H -6.236387 0.683047 -0.911360
Sc 2.367141 -0.329798 -0.117334
Cl 3.672569 -2.278524 -0.569916
Cl 2.947915 0.316674 2.091542
Cl 3.362302 1.239428 -1.641860
35
TS-III-ScCl3-con-5 Eopt -2850.823814
O -0.637974 -1.409457 -0.259574
C 0.512878 -0.843664 -0.291622
C 0.638196 0.546965 -0.124905
N 1.649629 -1.589740 -0.473321
O -0.606573 1.142529 -0.093743
C 1.819093 1.256656 -0.733240
C 1.519013 -3.041880 -0.650102
C -0.597712 2.542562 0.301562
C 1.815323 2.727040 -0.316580
H 1.854137 1.173738 -1.830304
H 0.497711 -3.250278 -0.955138
H 1.732090 -3.554186 0.290733
H 2.210115 -3.368707 -1.425904
C 0.416335 3.309747 -0.525916
H -1.619204 2.883377 0.139991
H -0.357009 2.571283 1.370655
H 2.072568 2.809630 0.747981
H 2.563995 3.282284 -0.887431
H 0.389070 4.358473 -0.217798
H 0.129999 3.266570 -1.582930
C 2.862805 -1.006584 -0.235962
C 2.888731 0.423981 -0.015322
C 4.027844 -1.782862 -0.049206
C 4.159822 1.008408 0.335348
H 1.937016 0.418628 0.928779
C 5.194764 -1.168494 0.340183
H 4.002246 -2.855802 -0.189477
C 5.264044 0.233714 0.547364
H 4.221149 2.086474 0.435803
H 6.081549 -1.774084 0.494755
H 6.204472 0.686964 0.839475
Sc -2.375951 -0.319168 0.037330
Cl -3.742250 -2.216249 -0.460192
Cl -3.640301 1.434496 -1.012200
Cl -2.425847 -0.041881 2.396336
35
TS-III-ScCl3-con-6 Eopt -2850.814504
Sc 2.381954 -0.288378 -0.106665
O 0.653871 -1.427392 -0.049873
C -0.496593 -0.877436 0.127965
C -0.644345 0.512971 0.140482
N -1.627125 -1.645680 0.281054
O 0.561302 1.155162 0.007522
C -1.824490 1.109159 0.877234
C -1.479901 -3.106058 0.323180
C -2.846411 -1.055542 0.105030
C 0.520750 2.608140 -0.158718
C -1.786166 2.641720 0.790418
H -1.874435 0.790447 1.927594
H -0.457093 -3.329644 0.613034
H -2.169144 -3.510595 1.063032
H -1.683256 -3.534207 -0.660885
C -4.004851 -1.818718 -0.161614
C -2.880156 0.394911 0.020594
C -0.899994 3.103200 -0.367485
H 1.171651 2.823148 -1.005337
H 0.954805 3.026063 0.754327
H -1.353284 3.014975 1.724077
H -2.790329 3.065241 0.728229
C -5.164219 -1.179491 -0.531124
H -3.975757 -2.899417 -0.106268
C -4.146938 0.999164 -0.319204
H -1.941862 0.471023 -0.906758
H -0.861887 4.195062 -0.408239
H -1.285961 2.758992 -1.333883
C -5.233284 0.235349 -0.635040
H -6.043611 -1.773247 -0.757036
H -4.217374 2.080408 -0.347824
H -6.164835 0.704899 -0.930247
Cl 2.906546 0.330770 2.125753
Cl 3.347595 1.318360 -1.606924
Cl 3.725824 -2.209944 -0.578401
35
TS-III-ScCl3-con-7 Eopt -2850.816596
Sc -2.369852 -0.315194 -0.014129
O -0.681413 -1.450903 -0.428889
C 0.468725 -0.877893 -0.485673
C 0.593488 0.512785 -0.425300
N 1.625068 -1.619980 -0.572997
O -0.626443 1.142906 -0.389784
C 1.814893 1.168865 -1.023318
C 1.510688 -3.080681 -0.668988
C 2.811850 -1.015704 -0.267078
C -0.619715 2.544653 0.044340
C 1.714272 2.689497 -0.840100
H 1.963861 0.920517 -2.083307
H 0.521542 -3.312424 -1.053767
H 2.270135 -3.452308 -1.355498
H 1.634109 -3.533826 0.317283
C 3.958349 -1.769281 0.069825

C 2.811450 0.430248 -0.119939
C 0.791463 3.035400 0.330617
H -1.263816 2.591411 0.924674
H -1.077561 3.101373 -0.775561
H 1.282301 3.103696 -1.757141
H 2.699650 3.146986 -0.731827
C 5.069809 -1.126372 0.560845
H 3.955728 -2.847277 -0.028199
C 4.032470 1.041485 0.347891
H 1.797200 0.442168 0.728172
H 0.735627 4.118525 0.468202
H 1.162420 2.612689 1.271233
C 5.104331 0.284056 0.723112
H 5.937949 -1.714878 0.838267
H 4.078167 2.122256 0.419681
H 5.999786 0.756262 1.111057
Cl -3.728385 1.522189 -0.757375
Cl -2.021617 0.029321 2.314048
Cl -3.850896 -2.178252 -0.198648
35
TS-III-ScCl3-dis-1 Eopt -2850.807078
O 0.587631 1.206653 0.524674
C -0.546308 -0.656751 -0.253468
C -0.654732 0.590212 0.402509
C 0.540665 2.594935 0.051205
N -1.682398 -1.309394 -0.654583
C -1.832852 1.471490 0.148202
C -0.197925 2.660968 -1.282838
H 1.582825 2.890098 -0.048465
H 0.069703 3.195718 0.833473
C -2.895158 -0.831531 -0.240418
C -1.559042 -2.617437 -1.310749
C -1.688236 2.221435 -1.175761
H -1.911797 2.188082 0.970297
H -0.117812 3.681312 -1.666378
H 0.339609 2.013655 -1.984617
C -2.912166 0.427314 0.462572
C -4.070439 -1.608732 -0.341233
H -0.536344 -2.721091 -1.660896
H -1.786384 -3.414585 -0.599554
H -2.244254 -2.656864 -2.156625
H -2.360376 3.082559 -1.201007
H -1.948036 1.571197 -2.016977
C -4.167900 0.860154 1.015790
C -5.231585 -1.157410 0.242117
H -4.057431 -2.562742 -0.852444
C -5.284306 0.076657 0.940464
H -4.200743 1.833749 1.495470
H -6.126648 -1.766157 0.169629
H -6.218482 0.404780 1.381732
O 0.602723 -1.198114 -0.439647
H -1.921759 0.083523 1.332663
Sc 2.356201 -0.283172 0.171293
Cl 3.637347 -2.218818 -0.403615
Cl 3.389917 1.332925 -1.260977
Cl 2.808710 -0.112165 2.509185
35
TS-III-ScCl3-dis-2 Eopt -2850.804472
O -0.625713 1.017885 -0.820158
C 0.518472 0.529431 -0.511950
C 0.601871 -0.650039 0.268899
N 1.668776 1.181638 -0.871006
O -0.625599 -1.322419 0.287478
C 1.833311 -1.493101 0.255625
C 1.574027 2.412230 -1.666186
C -0.670392 -2.274329 -0.839054
C 1.872047 -2.402327 -0.965590
H 1.823806 -2.116012 1.156861
H 0.590877 2.435645 -2.127165
H 2.342694 2.396870 -2.438080
H 1.698310 3.285806 -1.022491
C 0.550273 -3.186520 -0.895897
H -1.592175 -2.836261 -0.690326
H -0.754119 -1.679437 -1.756681
H 1.926084 -1.809854 -1.887519
H 2.728087 -3.081684 -0.942778
H 0.426035 -3.826089 -1.774574
H 0.558870 -3.839885 -0.016187
C 2.842328 0.805843 -0.271630
C 2.830308 -0.370098 0.563046
C 3.990046 1.625962 -0.326651
C 4.024149 -0.671626 1.304722
C 5.090899 1.301206 0.433117
H 4.000416 2.517714 -0.939781
C 5.110395 0.156604 1.269850
H 4.036279 -1.584106 1.893047
H 5.962707 1.945907 0.394340
H 5.996625 -0.070022 1.851231
H 1.724499 0.003613 1.271511
Sc -2.312992 0.320940 0.209920
Cl -3.209013 2.525899 0.413667
Cl -3.595807 -0.698363 -1.500953
Cl -2.934797 -0.593216 2.338401
35
TS-III-ScCl3-dis-3 Eopt -2850.807997
O 0.711310 -0.911335 -0.949257
C -0.438006 -0.443052 -0.619113
C -0.535492 0.696035 0.213763
N -1.579796 -1.091825 -1.012716
O 0.687011 1.371615 0.295446
C -1.775298 1.526806 0.233211
C -1.465665 -2.289979 -1.853699
C 0.737802 2.397275 -0.765469
C -1.809367 2.494877 -0.941471
H -1.777286 2.105413 1.163659
H -0.483385 -2.280019 -2.316831
H -2.236300 -2.259188 -2.623006
H -1.573329 -3.188740 -1.242328
C -0.499519 3.289625 -0.805421
H 1.641168 2.966939 -0.555355
H 0.855501 1.864886 -1.718159
H -1.840040 1.948588 -1.892528
H -2.674098 3.162248 -0.899737
H -0.364394 3.973308 -1.648686
H -0.537765 3.899585 0.104105
C -2.759038 -0.757282 -0.403116
C -2.762881 0.381663 0.481468
C -3.895701 -1.590598 -0.493844
C -3.963486 0.637642 1.230398
C -5.001770 -1.312491 0.275817
H -3.891612 -2.457452 -1.141813
C -5.038550 -0.202030 1.157677
H -3.989343 1.525120 1.855426
H -5.864088 -1.967675 0.209838
H -5.929522 -0.010561 1.744494
H -1.657238 -0.016741 1.174908
Sc 2.344610 -0.306744 0.188106
Cl 3.626563 -2.203132 -0.508105
Cl 3.932114 1.461806 -0.109637
Cl 1.629483 -0.895999 2.376218
35
TS-III-ScCl3-dis-4 Eopt -2850.804027
O -0.585019 -1.087903 0.727812
C 0.552763 -0.571492 0.447380
C 0.625974 0.632489 -0.296135
N 1.712616 -1.213097 0.799256
O -0.614030 1.281652 -0.317589
C 1.838028 1.500594 -0.243830
C 1.632145 -2.470888 1.552091
C -0.697168 2.223866 0.816933
C 1.838725 2.384947 0.996244
H 1.827703 2.144498 -1.130325
H 0.644413 -2.527736 2.000158
H 2.391556 -2.467548 2.333225
H 1.780029 -3.320200 0.881354
C 0.508009 3.152944 0.912040
H -1.624152 2.773926 0.650948
H -0.789905 1.619092 1.727049
H 1.880928 1.775243 1.907440
H 2.685015 3.076700 1.005324
H 0.356280 3.775260 1.798736
H 0.525178 3.821103 0.043839
C 2.886270 -0.794807 0.229322
C 2.862537 0.407474 -0.567204
C 4.049729 -1.593723 0.272306
C 4.060218 0.757201 -1.281314
C 5.153841 -1.222386 -0.460923
H 4.070389 -2.505339 0.855238
C 5.162256 -0.050054 -1.258592
H 4.061777 1.689142 -1.838539
H 6.037548 -1.851164 -0.431739
H 6.051610 0.213371 -1.819367
H 1.777584 0.031562 -1.302313
Sc -2.309888 -0.333151 -0.219817
Cl -3.145274 -2.497922 -0.769010
Cl -3.468965 0.343647 1.728063
Cl -3.291890 0.951507 -1.997368
35
TS-III-ScCl3-dis-5 Eopt -2850.805872
O 0.655198 -1.006059 -0.848926
C -0.492261 -0.518005 -0.544150
C -0.585381 0.651281 0.250967
N -1.635219 -1.168904 -0.925489
O 0.636237 1.334535 0.289619
C -1.827064 1.481301 0.255032
C -1.527632 -2.385087 -1.741348
C 0.663993 2.339613 -0.792529
C -1.877556 2.424448 -0.938502
H -1.825162 2.080124 1.172507
H -0.536342 -2.402883 -2.184528
H -2.281954 -2.353770 -2.526809
H -1.665869 -3.269591 -1.115759
C -0.573168 3.229168 -0.828733
H 1.569346 2.914525 -0.609075
H 0.768945 1.790692 -1.736812
H -1.912107 1.859693 -1.878574
H -2.747905 3.084674 -0.900840
H -0.449182 3.901114 -1.683028
H -0.602683 3.851524 0.072750
C -2.817457 -0.808663 -0.333765
C -2.818116 0.346120 0.528253
C -3.965248 -1.624846 -0.430609
C -4.023575 0.630667 1.256837
C -5.080761 -1.314350 0.313343
H -3.966018 -2.500220 -1.066992
C -5.112545 -0.191212 1.178238
H -4.045642 1.529323 1.865845
H -5.954929 -1.952603 0.238148
H -6.010469 0.026113 1.745064
H -1.700205 -0.032327 1.234449
Sc 2.332385 -0.312287 0.169454
Cl 3.801945 1.282732 -0.816837
Cl 2.331698 -0.213283 2.552126
Cl 3.496696 -2.362181 -0.221771
35
TS-III-ScCl3-dis-6 Eopt -2850.807681
O 0.664045 1.268368 0.296412

C -0.515503 -0.585980 -0.439917
C -0.584101 0.649944 0.244124
C 0.607721 2.617630 -0.287056
N -1.679844 -1.231996 -0.771861
C -1.776063 1.531997 0.064605
C -0.241911 2.610291 -1.554371
H 1.645459 2.874112 -0.487629
H 0.215030 3.288525 0.481862
C -2.859472 -0.764199 -0.261938
C -1.604442 -2.524292 -1.464825
C -1.730944 2.258756 -1.281158
H -1.794850 2.257031 0.882934
H -0.152335 3.591412 -2.027483
H 0.198072 1.883652 -2.246724
C -2.828482 0.487117 0.455866
C -4.038268 -1.542170 -0.286690
H -0.611550 -2.614563 -1.895501
H -1.772854 -3.339243 -0.757513
H -2.353191 -2.547671 -2.255791
H -2.352188 3.157175 -1.249322
H -2.115841 1.616969 -2.079878
C -4.041909 0.911883 1.103125
C -5.154271 -1.099675 0.385046
H -4.061511 -2.490429 -0.808115
C -5.158974 0.126276 1.100109
H -4.040575 1.879549 1.595655
H -6.051167 -1.709908 0.370936
H -6.059414 0.446010 1.612011
O 0.618088 -1.119397 -0.712094
H -1.791287 0.139551 1.251565
Sc 2.352425 -0.297018 0.100832
Cl 3.730647 -2.016853 -0.830054
Cl 3.852647 1.588763 0.089579
Cl 1.990756 -0.914736 2.361798
35
TS-III-ScCl3-dis-7 Eopt -
2850.807805
O 0.592674 -1.136800 -0.531501
C -0.599445 0.751570 0.054073
C -0.673101 -0.566897 -0.449167
C 0.629317 -2.472194 0.077884
N -1.755195 1.400690 0.403482
C -1.804963 -1.466538 -0.082409
C -0.106222 -2.467704 1.417289
H 1.690246 -2.689091 0.201306
H 0.205145 -3.174361 -0.644600
C -2.954808 0.824939 0.084626
C -1.676140 2.782044 0.894711
C -1.610063 -2.080874 1.301594
H -1.865043 -2.263758 -0.828705
H 0.009081 -3.460370 1.860588
H 0.413696 -1.764329 2.073681
C -2.934217 -0.505168 -0.474576
C -4.162711 1.555565 0.138789
H -0.647523 2.974776 1.184799
H -1.971453 3.476095 0.104762
H -2.329683 2.891472 1.759358
H -2.258400 -2.951511 1.426093
H -1.869388 -1.358306 2.081760
C -4.184287 -1.050881 -0.932295
C -5.318956 0.990688 -0.347671
H -4.180382 2.560202 0.541313
C -5.334605 -0.314987 -0.902663
H -4.185891 -2.073304 -1.298334
H -6.239614 1.563258 -0.308494
H -6.265672 -0.732673 -1.268471
O 0.533093 1.348090 0.142451
H -1.977620 -0.218312 -1.402548
Sc 2.327423 0.360030 -0.265519
Cl 3.622338 2.368098 -0.283129
Cl 2.910844 -0.569243 1.841502
Cl 3.481997 -1.076085 -1.811296
35
3C-ScCl3-con-2-MECP Eopt -
2850.820901
O -0.685549 -1.418838 -0.109727
C 0.480061 -0.900381 -0.111111
C 0.609039 0.522664 -0.204913
N 1.586352 -1.669022 -0.046286
O -0.585113 1.138316 -0.278170
C 1.921607 1.220127 -0.277668
C 1.453718 -3.124269 -0.112063
C -0.667029 2.567619 0.044114
C 1.817549 2.664970 0.212767
H 2.246104 1.231056 -1.330602
H 0.446001 -3.396126 0.191744
H 2.182740 -3.569064 0.565803
H 1.632720 -3.475572 -1.132619
C 0.574893 3.305618 -0.402149
H -1.572795 2.898297 -0.461896
H -0.805746 2.617979 1.128183
H 1.746616 2.681991 1.308212
H 2.716307 3.215072 -0.073693
H 0.470577 4.346370 -0.084374
H 0.642689 3.299171 -1.495798
C 2.873061 -1.073553 -0.030608
C 2.931799 0.329014 0.509461
C 3.974242 -1.751535 -0.464821
C 4.321474 0.886260 0.499593
H 2.560063 0.319297 1.550729
C 5.256426 -1.145323 -0.433423
H 3.878188 -2.760881 -0.849989
C 5.397029 0.177538 0.051833
H 4.451714 1.888282 0.895904
H 6.118361 -1.697771 -0.786294
H 6.383334 0.629985 0.079415
Sc -2.452001 -0.325256 -0.072330
Cl -3.855648 -2.246638 -0.066415
Cl -3.526006 1.232444 -1.528185
Cl -2.512889 0.452847 2.168442
35
3C-ScCl3-con-5-MECP Eopt -
2850.820964
O -0.685221 -1.416502 -0.090332
C 0.480859 -0.898904 -0.088707
C 0.611520 0.523628 -0.185251
N 1.585619 -1.668922 -0.014758
O -0.581366 1.140385 -0.274383
C 1.922815 1.223091 -0.265073
C 1.446288 -3.125421 -0.040976
C -0.665609 2.564325 0.071365
C 1.820633 2.664291 0.237930
H 2.233506 1.242233 -1.322211
H 0.441122 -3.384262 0.282169
H 2.181088 -3.555965 0.639383
H 1.610360 -3.505952 -1.052926
C 0.574840 3.312704 -0.362436
H -1.571326 2.903189 -0.429333
H -0.805304 2.596015 1.156084
H 1.755866 2.671085 1.333728
H 2.717738 3.217381 -0.048212
H 0.469806 4.346760 -0.023073
H 0.640979 3.328957 -1.455951
C 2.872923 -1.074951 -0.025716
C 2.944776 0.331260 0.504469
C 3.965137 -1.757824 -0.475698
C 4.336142 0.884874 0.466062
H 2.593292 0.326460 1.552823
C 5.247466 -1.153339 -0.475299
H 3.860107 -2.770089 -0.850997
C 5.400727 0.172377 -0.000298
H 4.477630 1.887523 0.857023
H 6.101419 -1.710357 -0.840120
H 6.388248 0.623259 0.003388
Sc -2.452197 -0.325625 -0.085430
Cl -3.858967 -2.244317 -0.098351
Cl -3.513488 1.244493 -1.539550
Cl -2.528494 0.441978 2.158551
35
3C-ScCl3-dis-6-MECP Eopt -
2850.814035
O 0.567006 1.179061 0.039961
C -0.439952 -0.901705 -0.015235
C -0.570799 0.487331 0.248246
C 0.364343 2.621627 -0.109711
N -1.554800 -1.664460 -0.096181
C -1.818106 1.205171 0.578988
C -0.802074 2.849139 -1.062287
H 1.308761 2.988650 -0.503424
H 0.196157 3.034090 0.888378
C -2.822737 -1.040795 -0.037188
C -1.425218 -3.073194 -0.468859
C -2.117534 2.208055 -0.558893
H -1.650038 1.777694 1.501812
H -0.917209 3.927042 -1.198708
H -0.521873 2.429966 -2.033398
C -2.916962 0.150321 0.879823
C -3.903210 -1.546782 -0.707280
H -0.454844 -3.431872 -0.131616
H -2.221706 -3.636417 0.017111
H -1.494475 -3.194102 -1.553641
H -2.809696 2.965805 -0.184022
H -2.618123 1.680511 -1.377389
C -4.287928 0.753943 0.906093
C -5.166098 -0.920336 -0.609414
H -3.797118 -2.426838 -1.333276
C -5.329838 0.234403 0.201686
H -4.425827 1.626001 1.539794
H -6.005153 -1.321666 -1.164606
H -6.306328 0.703983 0.263225
O 0.724227 -1.388356 -0.221866
H -2.679563 -0.218356 1.895032
Sc 2.462748 -0.241474 -0.037845
Cl 3.922520 -2.045226 -0.562354
Cl 3.518154 1.617507 -1.110425
Cl 2.597545 0.035306 2.309376
35
3C-ScCl3-dis-7-MECP-S0 Eopt
-2850.815754
O 0.604867 -1.155152 -0.469343
C -0.438042 0.907161 -0.452101
C -0.565769 -0.500702 -0.580653
C 0.473610 -2.556658 -0.059222
N -1.554921 1.661727 -0.332785
C -1.824760 -1.264475 -0.678317
C -0.573852 -2.648055 1.042459
H 1.469238 -2.834026 0.283157
H 0.221276 -3.134424 -0.952089
C -2.808187 1.020651 -0.186836
C -1.414008 3.098851 -0.098794
C -1.961765 -2.134426 0.594421
H -1.751705 -1.937017 -1.544181
H -0.626546 -3.689169 1.369849
H -0.205511 -2.058785 1.887517
C -2.977706 -0.262551 -0.957378
C -3.816887 1.581056 0.548341
H -0.494445 3.436948 -0.571843
H -2.270565 3.608419 -0.539539
H -1.364966 3.313284 0.972661
H -2.642546 -2.961813 0.380073
H -2.412931 -1.533456 1.390884
C -4.326277 -0.883999 -0.756113
C -5.067877 0.933079 0.664178

H	-3.660968	2.523133	1.063607
C	-5.293739	-0.304910	0.005921
H	-4.512512	-1.823138	-1.270196
H	-5.847629	1.382531	1.266730
H	-6.257859	-0.792209	0.110841
O	0.731337	1.424289	-0.400823
H	-2.870775	-0.002427	-2.026841
Sc	2.443109	0.271795	-0.088676
Cl	2.210503	-0.415226	2.168421
Cl	3.891252	2.159057	-0.056838
Cl	3.644687	-1.443882	-1.239997

8. HPLC Traces for Chiral Compounds

(4a*R*,10b*S*)-6-Methyl-2,3,6,10b-tetrahydro-1*H*-pyrano[2,3-*c*]quinolin-5(4a*H*)-one (2)

Chiral HPLC: (Chiralpak OD-H, 30% ^{i}PrOH, 70% hexane, 1.0 mL min^{-1}, λ = 210 nm) τ_R (major) = 11.1 min, τ_R (minor) = 16.5 min.

Racemic

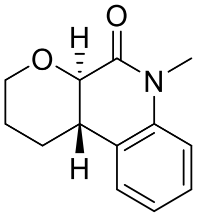

(rac)-**2**

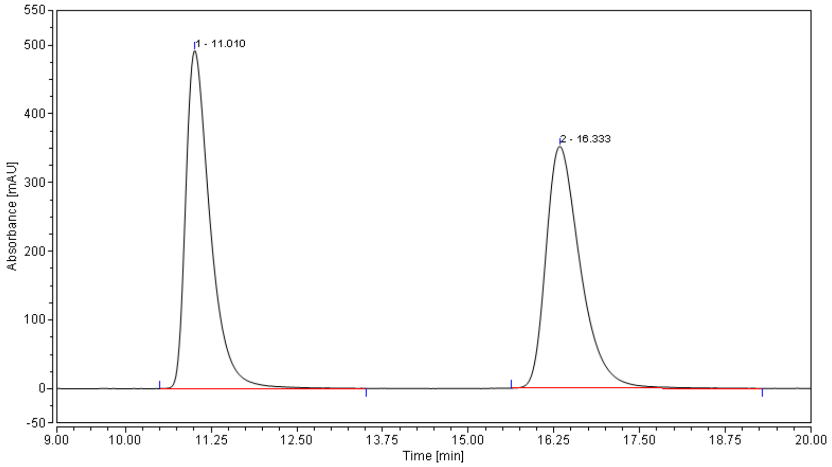

No.	Retention Time (min)	Area (mAU*min)	Height (mAU)	Relative Area (%)
1	11.010	203.568	491.786	49.86
2	16.333	204.734	351.968	50.14
Total:		408.302	843.754	100.00

Enantioselective

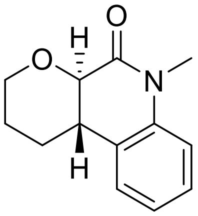

2

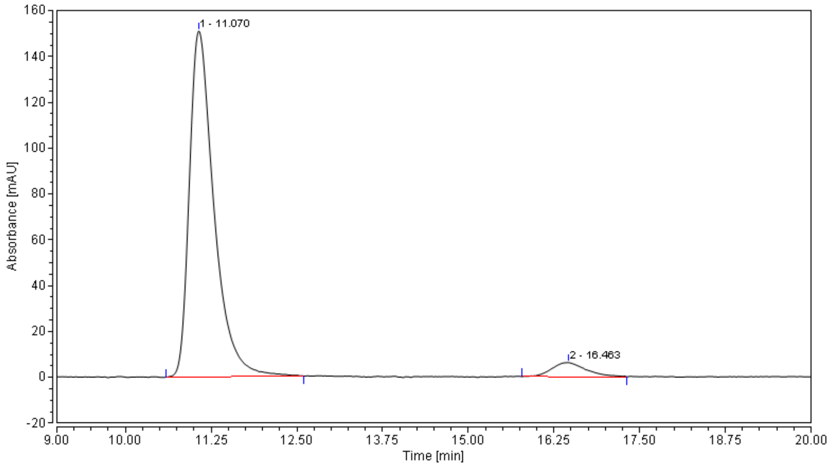

No.	Retention Time (min)	Area (mAU*min)	Height (mAU)	Relative Area (%)
1	11.070	62.899	151.094	94.55
2	16.463	3.627	6.211	5.45
Total:		66.526	157.305	100.00

(4aR,10bS)-6-Benzyl-2,3,6,10b-tetrahydro-1H-pyrano[2,3-c]quinolin-5(4aH)-one (5)

Chiral HPLC: (Chiralpak OD-H, 30% ^{i}PrOH, 70% hexane, 1.0 mL min^{-1}, λ = 260 nm) τ_R (major) = 10.7 min, τ_R (minor) = 12.7 min.

Racemic

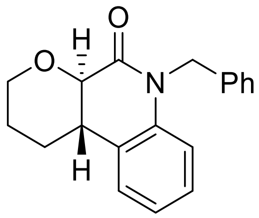

(rac)-**5**

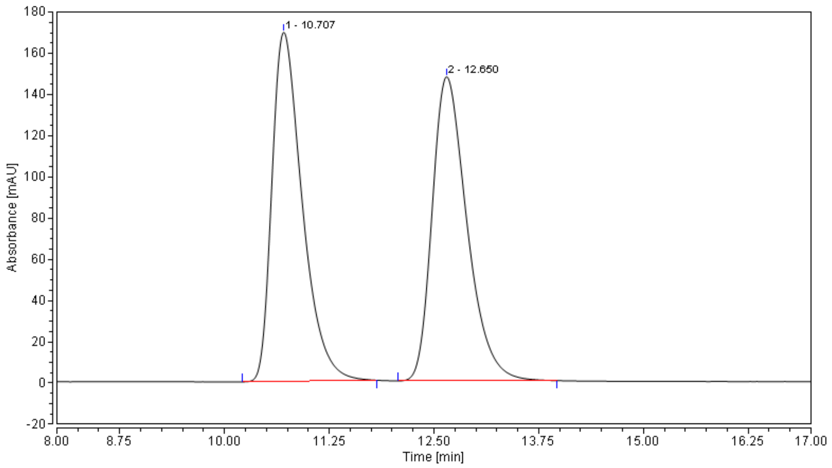

No.	Retention Time (min)	Area (mAU*min)	Height (mAU)	Relative Area (%)
1	10.707	70.918	169.482	49.99
2	12.650	70.948	147.583	50.01
**Total:**		141.866	317.066	100.00

Enantioselective

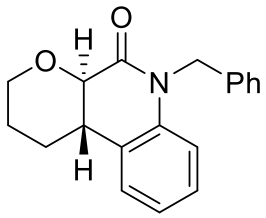

5

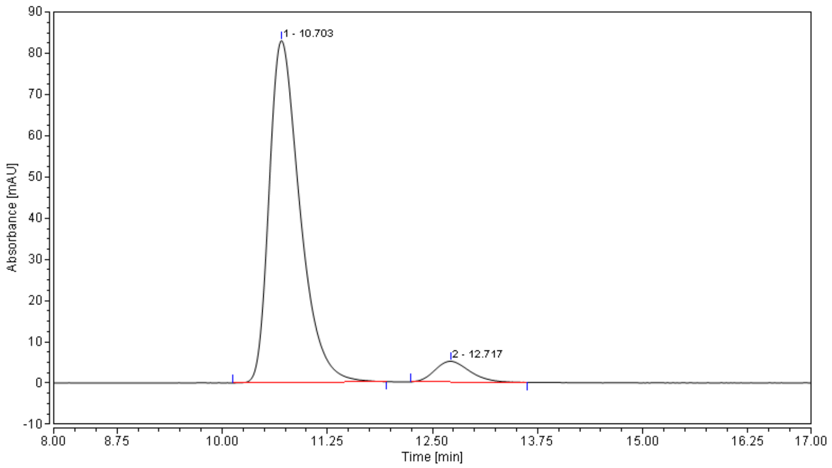

No.	Retention Time (min)	Area (mAU*min)	Height (mAU)	Relative Area (%)
1	10.703	35.111	83.001	93.55
2	12.717	2.421	5.061	6.45
**Total:**		37.532	88.062	100.00

(4a*R*,10b*S*)-6-Isobutyl-2,3,6,10b-tetrahydro-1*H*-pyrano[2,3-*c*]quinolin-5(4a*H*)-one (6)

Chiral HPLC: (Chiralpak OD-H, 30% ^{i}PrOH, 70% hexane, 1.0 mL min^{-1}, λ = 260 nm) τ_R (major) = 6.0 min, τ_R (minor) = 10.1 min.

Racemic

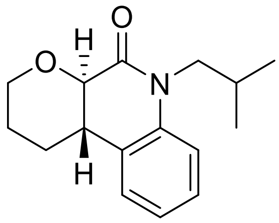

(*rac*)-**6**

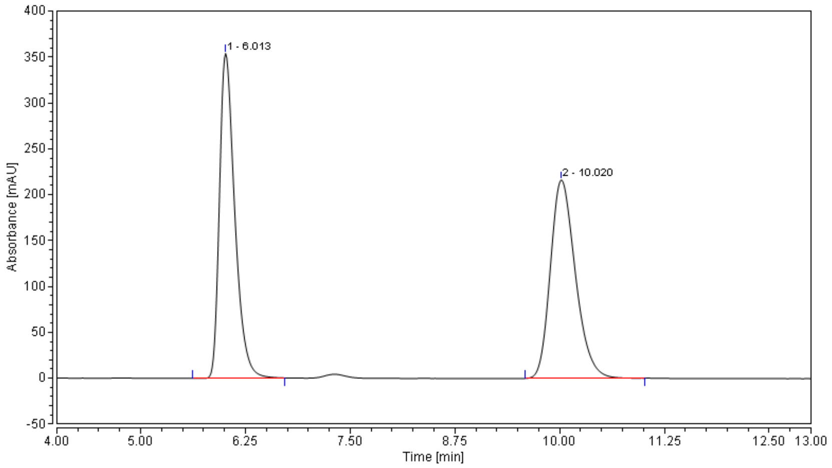

No.	Retention Time (min)	Area (mAU*min)	Height (mAU)	Relative Area (%)
1	6.013	74.810	354.179	50.04
2	10.020	74.681	216.199	49.96
**Total:**		149.491	570.377	100.00

Enantioselective

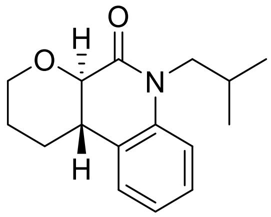

6

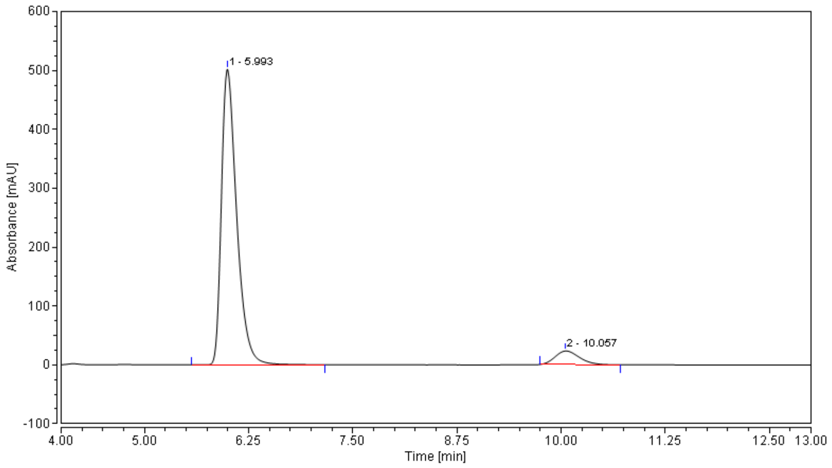

No.	Retention Time (min)	Area (mAU*min)	Height (mAU)	Relative Area (%)
1	5.993	106.310	502.170	93.20
2	10.057	7.753	23.069	6.80
**Total:**		114.063	525.239	100.00

(4a*R*,10b*S*)-6-Propyl-2,3,6,10b-tetrahydro-1*H*-pyrano[2,3-*c*]quinolin-5(4a*H*)-one (7)

Chiral HPLC: (Chiralpak OD-H, 30% ^{i}PrOH, 70% hexane, 1.0 mL min^{-1}, λ = 210 nm) τ_R (major) = 7.1 min, τ_R (minor) = 12.0 min.

Racemic

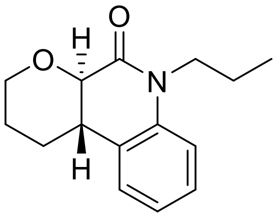

(rac)-**7**

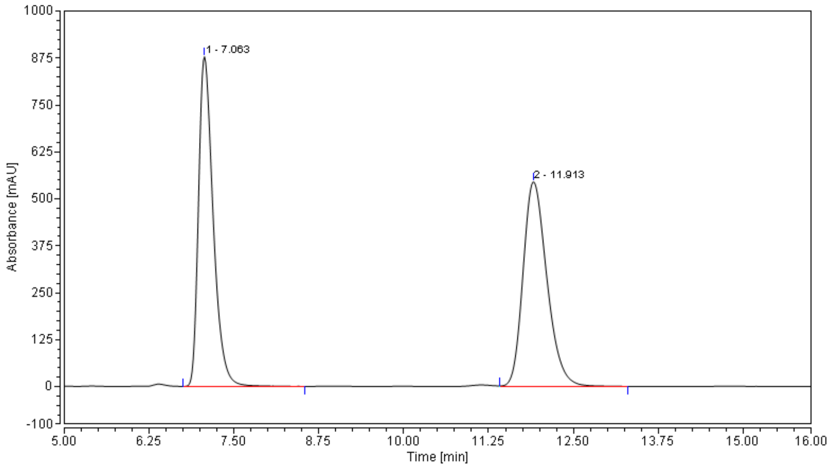

No.	Retention Time (min)	Area (mAU*min)	Height (mAU)	Relative Area (%)
1	7.063	220.115	878.849	49.74
2	11.913	222.381	545.415	50.26
Total:		442.495	1424.264	100.00

Enantioselective

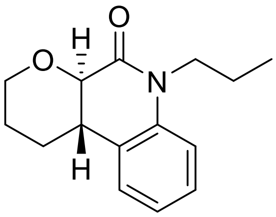

7

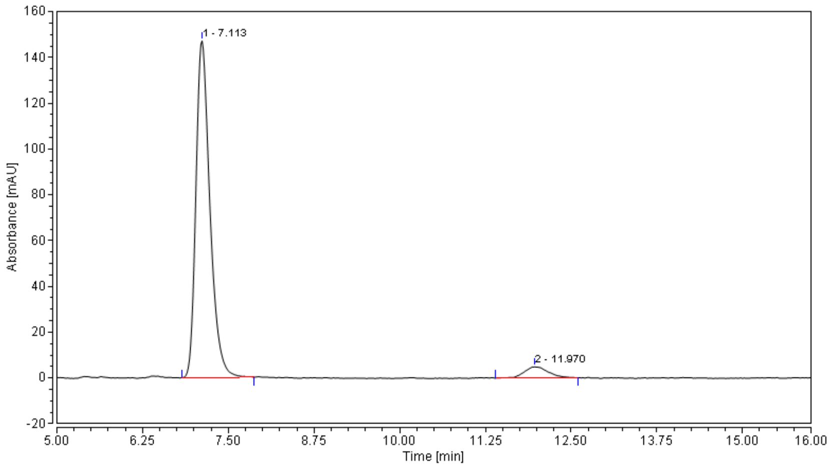

No.	Retention Time (min)	Area (mAU*min)	Height (mAU)	Relative Area (%)
1	7.113	34.889	147.221	94.52
2	11.970	2.025	4.924	5.48
Total:		36.913	152.146	100.00

(4a*R*,10b*S*)-6-Allyl-2,3,6,10b-tetrahydro-1*H*-pyrano[2,3-*c*]quinolin-5(4a*H*)-one (8)

Chiral HPLC: (Chiralpak OD-H, 30% ^{i}PrOH, 70% hexane, 1.0 mL min^{-1}, λ = 210 nm) τ_R (major) = 9.2 min, τ_R (minor) = 12.0 min.

<u>Racemic</u>

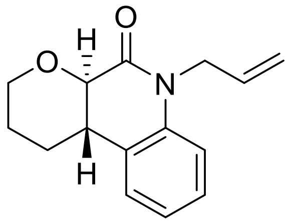

(rac)-**8**

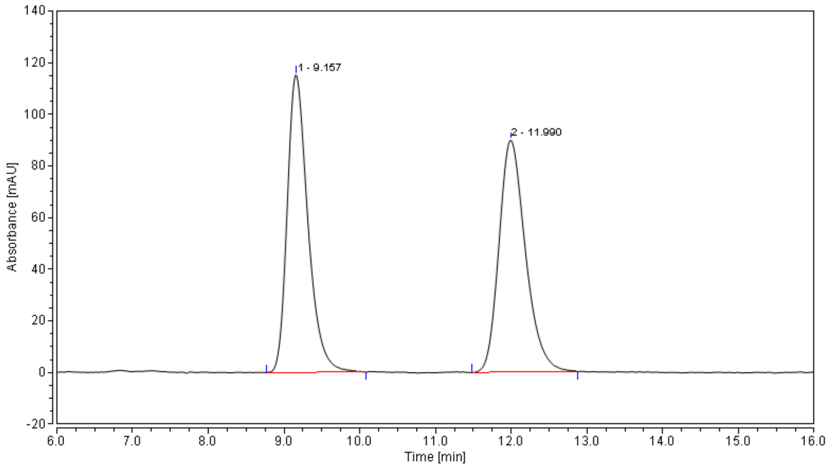

No.	Retention Time (min)	Area (mAU*min)	Height (mAU)	Relative Area (%)
1	9.157	36.484	115.459	50.03
2	11.990	36.445	89.948	49.97
Total:		72.929	205.406	100.00

<u>Enantioselective</u>

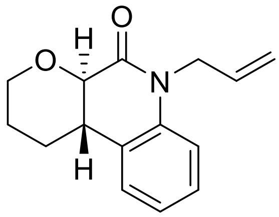

8

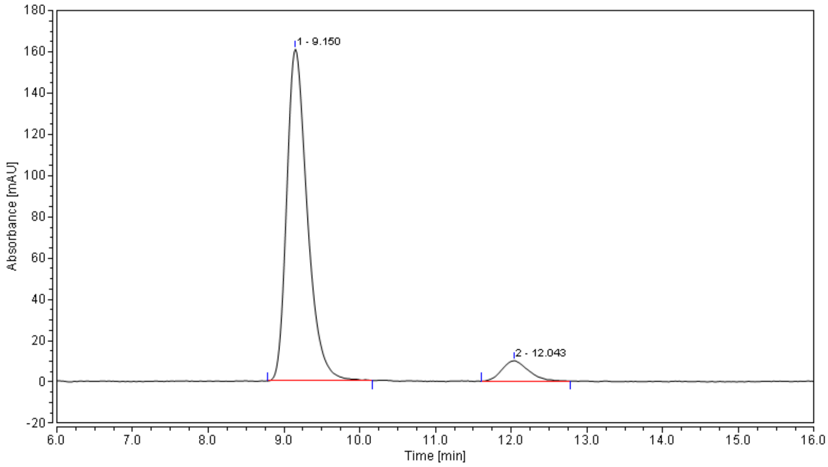

No.	Retention Time (min)	Area (mAU*min)	Height (mAU)	Relative Area (%)
1	9.150	50.640	160.888	92.53
2	12.043	4.086	9.948	7.47
Total:		54.726	170.836	100.00

(8a*R*,12a*S*)-5,6,10,11,12,12a-Hexahydro-4*H*-pyrano[2,3-*c*]pyrido[3,2,1-*ij*]quinolin-8(8a*H*)-one (9)

Chiral HPLC: (Chiralpak OD-H, 30% ^{i}PrOH, 70% hexane, 1.0 mL min^{-1}, λ = 260 nm) τ_R (major) = 13.2 min, τ_R (minor) = 15.7 min.

Racemic

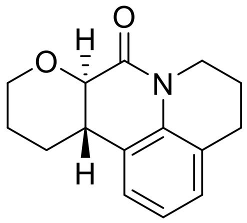

(rac)-**9**

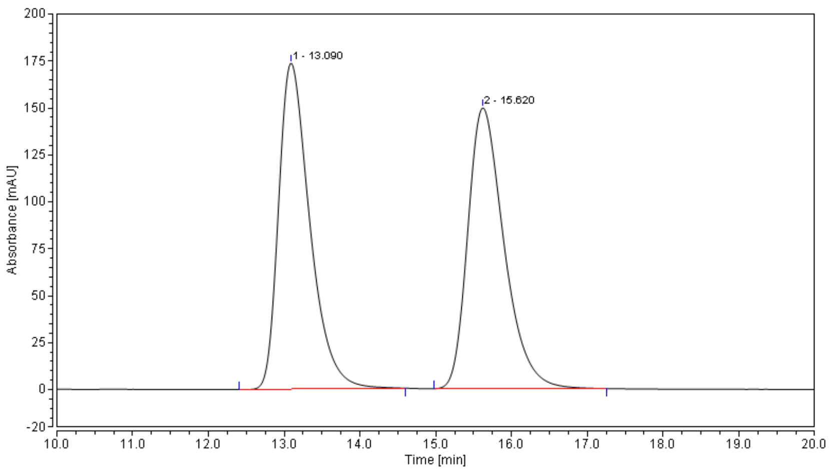

No.	Retention Time (min)	Area (mAU*min)	Height (mAU)	Relative Area (%)
1	13.090	82.891	173.703	50.00
2	15.620	82.876	149.671	50.00
**Total:**		165.767	323.374	100.00

Enantioselective

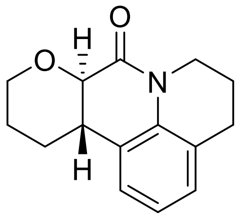

9

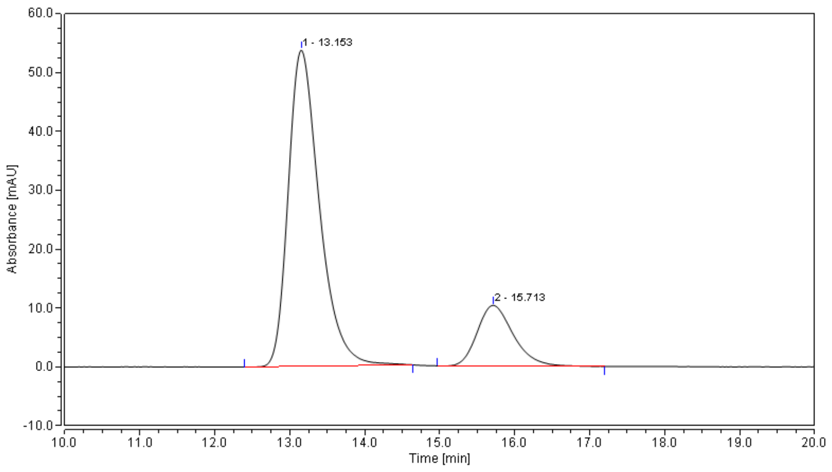

No.	Retention Time (min)	Area (mAU*min)	Height (mAU)	Relative Area (%)
1	13.153	25.892	53.714	81.78
2	15.713	5.767	10.324	18.22
**Total:**		31.659	64.038	100.00

(4a*R*,10b*S*)-6-(4-Methoxybenzyl)-2,3,6,10b-tetrahydro-1*H*-pyrano[2,3-*c*]quinolin-5(4a*H*)-one (10)

Chiral HPLC: (Chiralpak OD-H, 30% ^{i}PrOH, 70% hexane, 1.0 mL min^{-1}, λ = 210 nm) τ_R (major) = 13.0 min, τ_R (minor) = 15.8 min.

Racemic

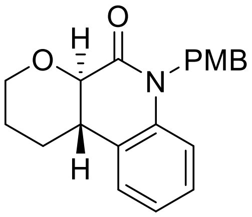

(rac)-**10**

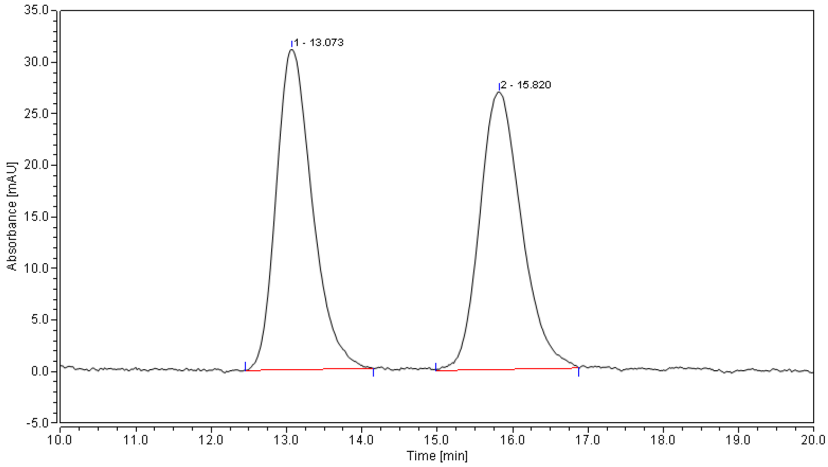

No.	Retention Time (min)	Area (mAU*min)	Height (mAU)	Relative Area (%)
1	13.073	17.247	31.079	49.84
2	15.820	17.357	26.922	50.16
Total:		34.604	58.001	100.00

Enantioselective

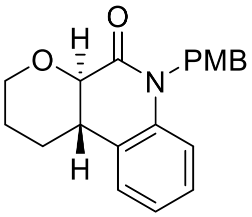

10

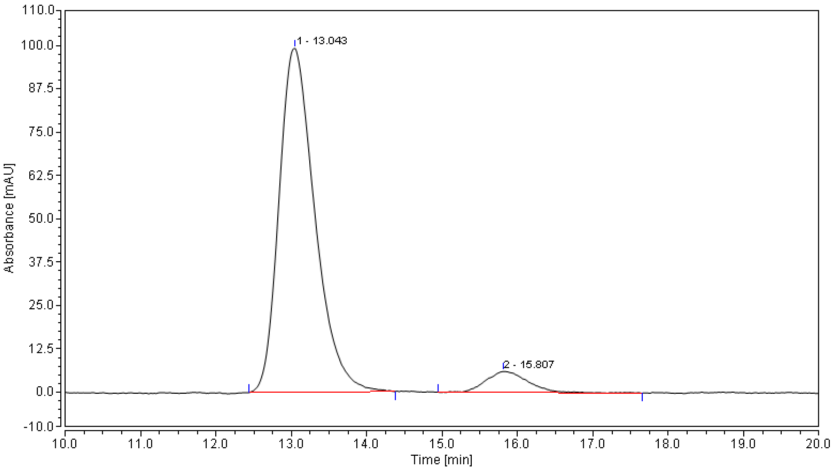

No.	Retention Time (min)	Area (mAU*min)	Height (mAU)	Relative Area (%)
1	13.043	54.693	99.249	93.22
2	15.807	3.979	6.043	6.78
Total:		58.672	105.291	100.00

(4a*R*,10b*S*)-8-Fluoro-6-methyl-2,3,6,10b-tetrahydro-1*H*-pyrano[2,3-*c*]quinolin-5(4a*H*)-one (11), and (4a*R*,10b*S*)-10-Fluoro-6-methyl-2,3,6,10b-tetrahydro-1*H*-pyrano[2,3-*c*]quinolin-5(4a*H*)-one (11b)

(4a*R*,10b*S*)-8-Fluoro-6-methyl-2,3,6,10b-tetrahydro-1*H*-pyrano[2,3-*c*]quinolin-5(4a*H*)-one (11)

Chiral HPLC: (Chiralpak IB N-3, 25% ^{i}PrOH, 75% hexane, 1.0 mL min^{-1}, λ = 210 nm) τ_R (major) = 17.0 min, τ_R (minor) = 22.3 min.

Racemic

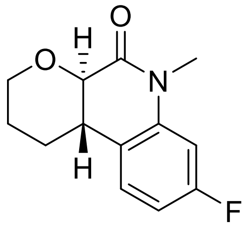

(rac)-**11**

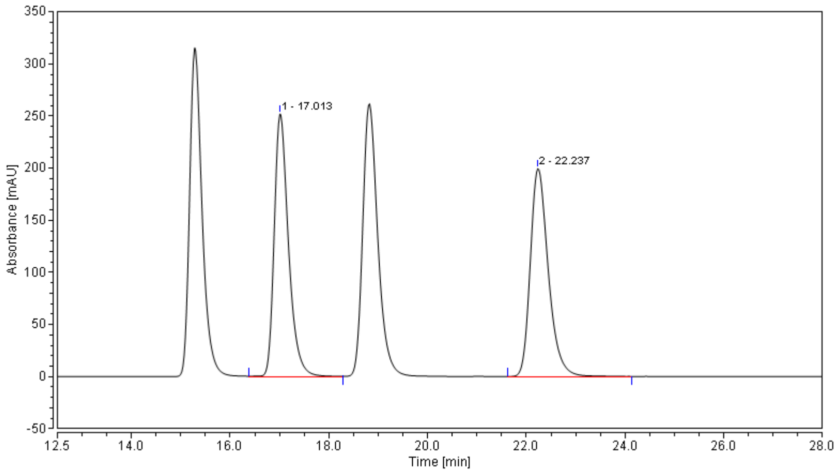

No.	Retention Time (min)	Area (mAU*min)	Height (mAU)	Relative Area (%)
1	17.013	84.238	251.994	50.02
2	22.237	84.182	199.732	49.98
**Total:**		168.42	451.726	100.00

Enantioselective

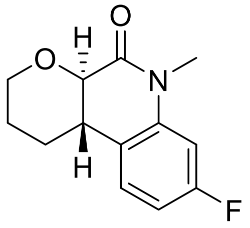

11

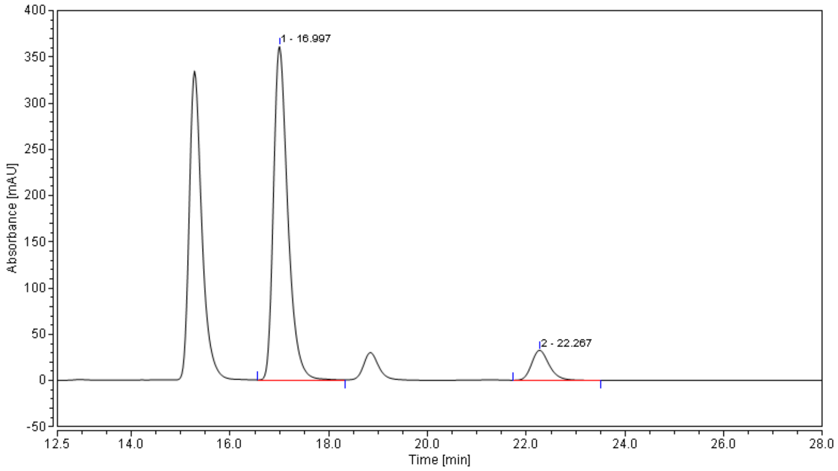

No.	Retention Time (min)	Area (mAU*min)	Height (mAU)	Relative Area (%)
1	16.997	121.490	360.833	90.03
2	22.267	13.452	32.464	9.97
**Total:**		134.942	393.297	100.00

(4a*R*,10b*S*)-10-Fluoro-6-methyl-2,3,6,10b-tetrahydro-1*H*-pyrano[2,3-*c*]quinolin-5(4a*H*)-one (11b)

Chiral HPLC: (Chiralpak IB N-3, 25% ^{i}PrOH, 75% hexane, 1.0 mL min^{-1}, λ = 210 nm) τ_R (major) = 15.3 min, τ_R (minor) = 18.8 min.

Racemic

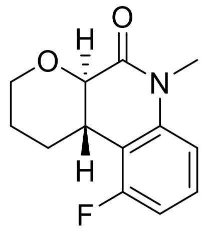

(rac)-**11b**

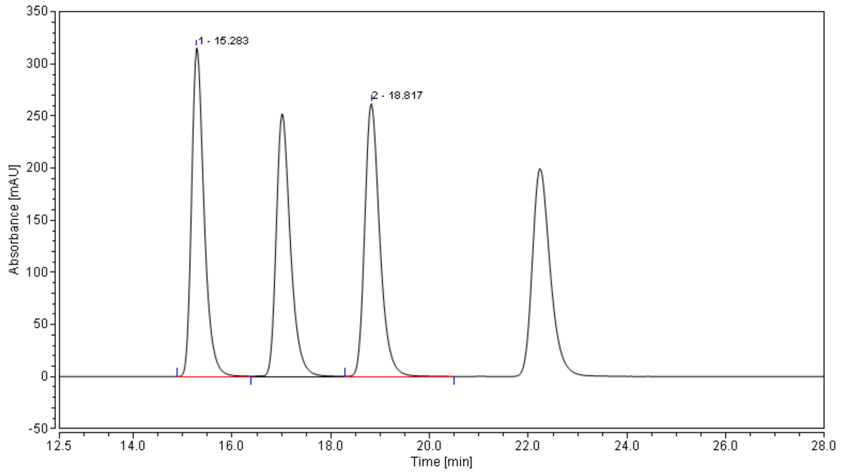

No.	Retention Time (min)	Area (mAU*min)	Height (mAU)	Relative Area (%)
1	15.283	92.385	315.391	49.96
2	18.817	92.534	262.069	50.04
**Total:**		184.919	577.460	100.00

Enantioselective

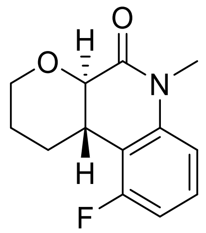

11b

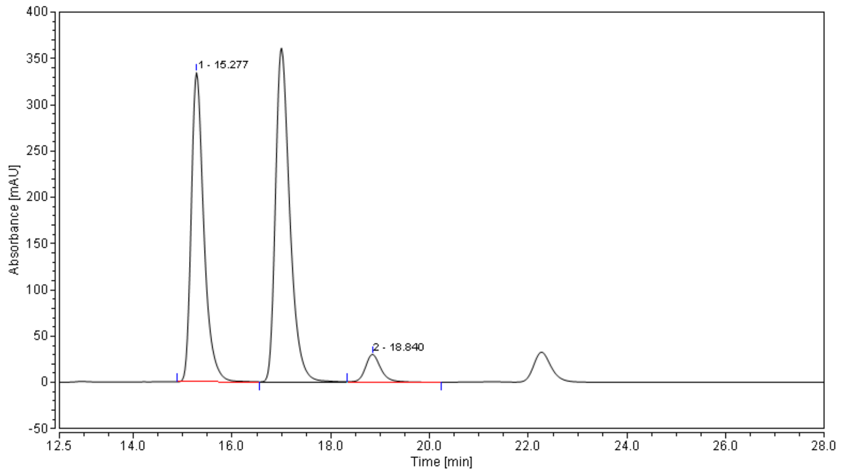

No.	Retention Time (min)	Area (mAU*min)	Height (mAU)	Relative Area (%)
1	15.277	99.067	334.192	90.09
2	18.840	10.901	29.982	9.91
**Total:**		109.968	364.174	100.00

(4a*R*,10b*S*)-8-Chloro-6-methyl-2,3,6,10b-tetrahydro-1*H*-pyrano[2,3-*c*]quinolin-5(4a*H*)-one (12), (4a*R*,10b*S*)-10-chloro-6-methyl-2,3,6,10b-tetrahydro-1*H*-pyrano[2,3-*c*]quinolin-5(4a*H*)-one (12b), and (4a*S*,10b*S*)-10-chloro-6-methyl-2,3,6,10b-tetrahydro-1*H*-pyrano[2,3-*c*]quinolin-5(4a*H*)-one (12b')

(4a*R*,10b*S*)-8-Chloro-6-methyl-2,3,6,10b-tetrahydro-1*H*-pyrano[2,3-*c*]quinolin-5(4a*H*)-one (12)

Chiral HPLC: (Chiralpak IB N-3, 30% ^{i}PrOH, 70% hexane, 1.0 mL min^{-1}, λ = 210 nm) τ_R (major) = 16.1 min, τ_R (minor) = 22.7 min.

Racemic

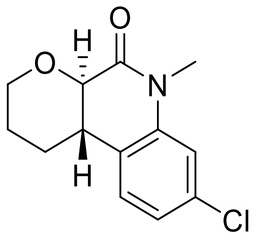

(rac)-**12**

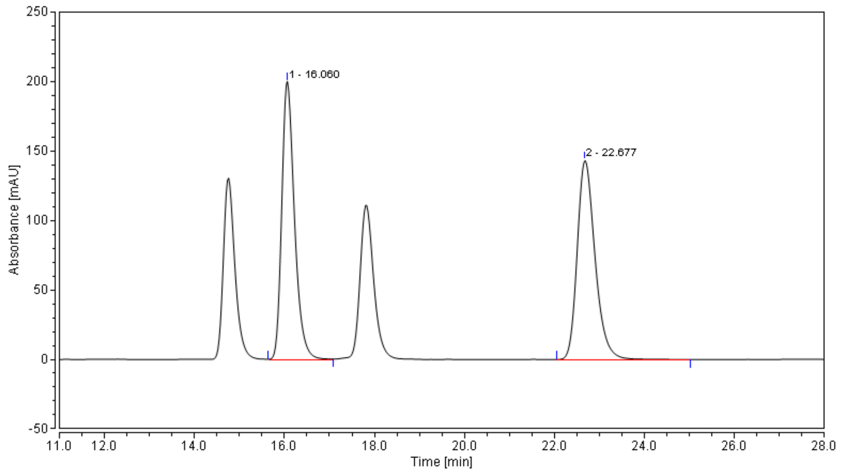

No.	Retention Time (min)	Area (mAU*min)	Height (mAU)	Relative Area (%)
1	16.060	65.652	200.297	49.91
2	22.677	65.887	143.459	50.09
**Total:**		131.539	343.756	100.00

Enantioselective

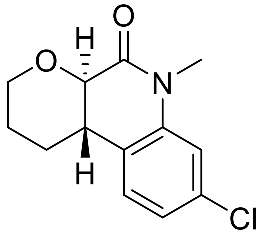

12

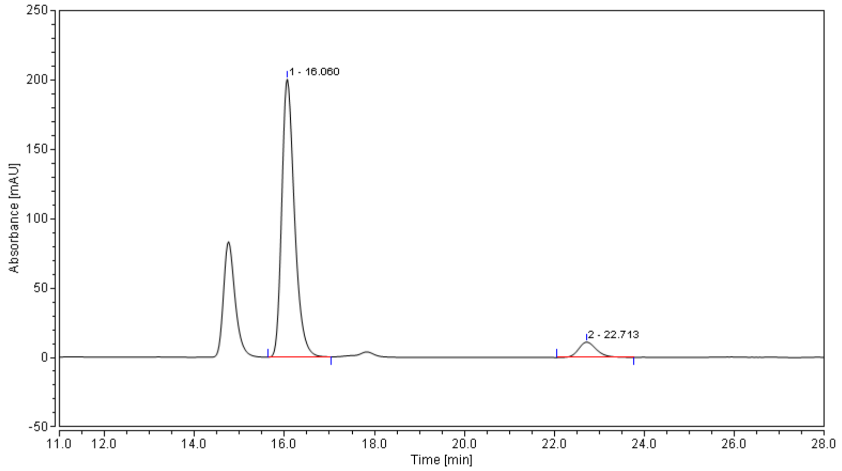

No.	Retention Time (min)	Area (mAU*min)	Height (mAU)	Relative Area (%)
1	16.060	66.042	200.526	92.77
2	22.713	5.150	11.074	7.23
**Total:**		71.192	211.600	100.00

(4a*R*,10b*S*)-10-chloro-6-methyl-2,3,6,10b-tetrahydro-1*H*-pyrano[2,3-*c*]quinolin-5(4a*H*)-one (12b)

Chiral HPLC: (Chiralpak IB N-3, 30% ^{i}PrOH, 70% hexane, 1.0 mL min^{-1}, λ = 254 nm) τ_R (major) = 14.8 min, τ_R (minor) = 17.8 min.

Racemic

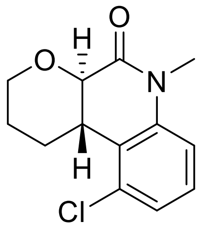

(rac)-**12b**

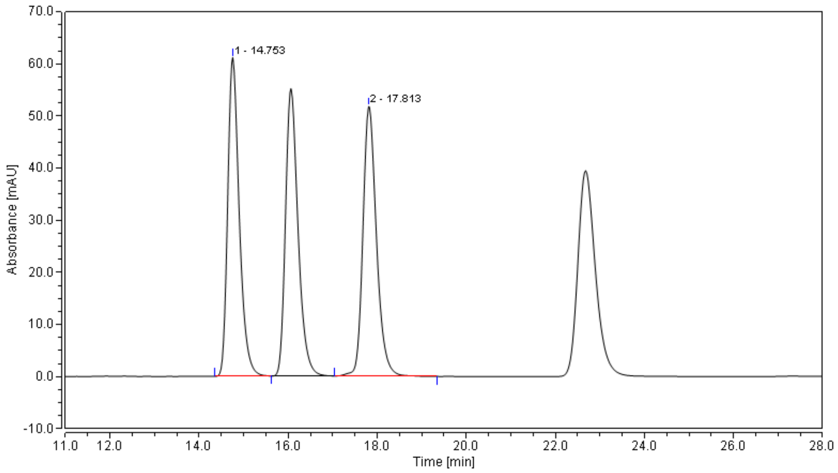

No.	Retention Time (min)	Area (mAU*min)	Height (mAU)	Relative Area (%)
1	14.753	18.403	61.194	50.07
2	17.813	18.351	51.830	49.93
**Total:**		36.754	113.024	100.00

Enantioselective

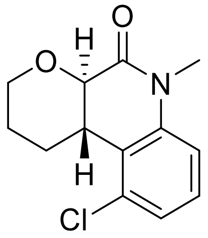

12b

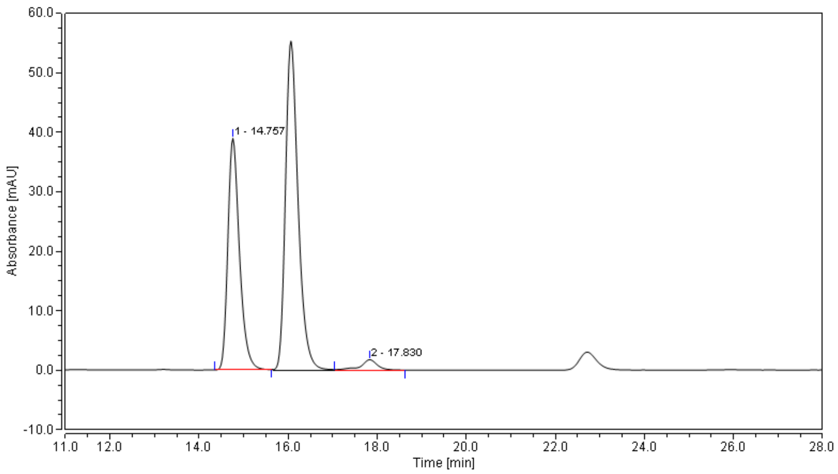

No.	Retention Time (min)	Area (mAU*min)	Height (mAU)	Relative Area (%)
1	14.757	11.803	38.972	93.87
2	17.830	0.771	1.753	6.13
**Total:**		12.574	40.725	100.00

(4a*S*,10b*S*)-10-Chloro-6-methyl-2,3,6,10b-tetrahydro-1*H*-pyrano[2,3-*c*]quinolin-5(4a*H*)-one (12b')

Chiral HPLC: (Chiralpak IB N-3, 30% ^{i}PrOH, 70% hexane, 1.0 mL min^{-1}, λ = 254 nm) τ_R (major) = 9.3 min, τ_R (minor) = 14.4 min.

Racemic

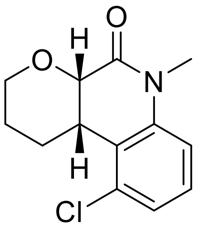

(*rac*)-**12b'**

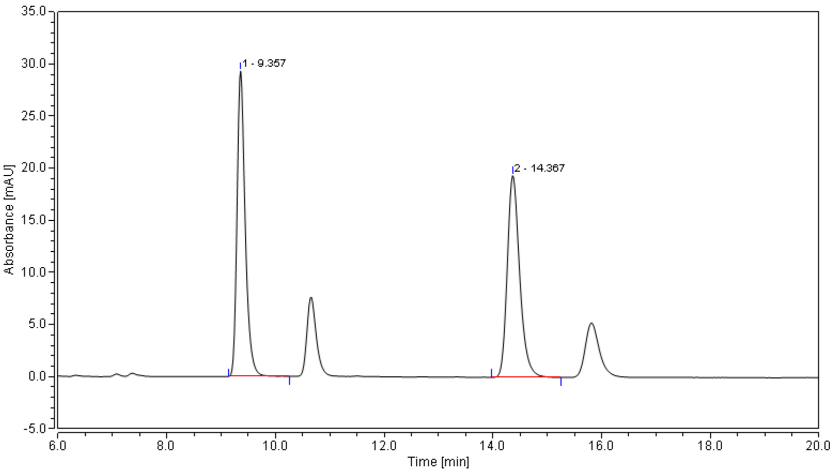

No.	Retention Time (min)	Area (mAU*min)	Height (mAU)	Relative Area (%)
1	9.357	5.138	29.304	50.05
2	14.367	5.128	19.385	49.95
**Total:**		10.267	48.690	100.00

Enantioselective

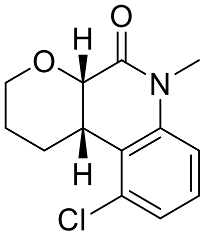

12b'

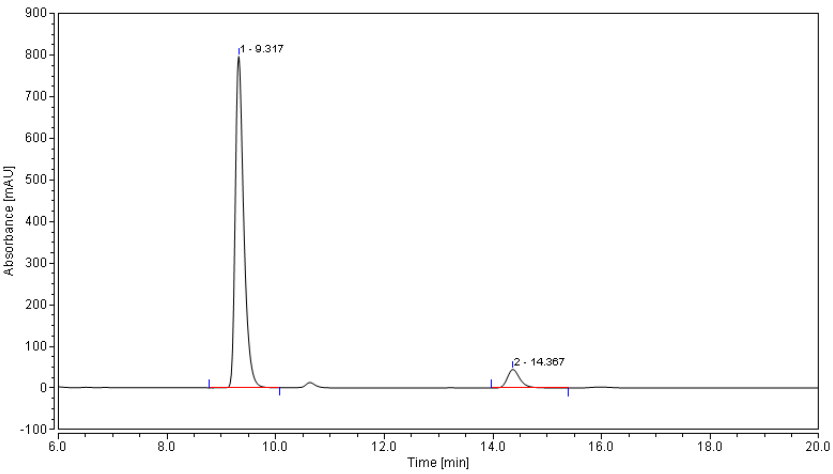

No.	Retention Time (min)	Area (mAU*min)	Height (mAU)	Relative Area (%)
1	9.317	144.784	796.819	92.36
2	14.367	11.983	44.515	7.64
**Total:**		156.767	841.335	100.00

(4a*R*,10b*S*)-8-Methoxy-6-methyl-2,3,6,10b-tetrahydro-1*H*-pyrano[2,3-*c*]quinolin-5(4a*H*)-one (13), (4a*R*,10b*S*)-10-methoxy-6-methyl-2,3,6,10b-tetrahydro-1*H*-pyrano[2,3-*c*]quinolin-5(4a*H*)-one (13b), and (4a*R*,10b*R*)-8-methoxy-6-methyl-2,3,6,10b-tetrahydro-1*H*-pyrano[2,3-*c*]quinolin-5(4a*H*)-one (13b')

(4a*R*,10b*S*)-8-Methoxy-6-methyl-2,3,6,10b-tetrahydro-1*H*-pyrano[2,3-*c*]quinolin-5(4a*H*)-one (13)

Chiral HPLC: (Chiralpak IB N-3, 20% ^{i}PrOH, 80% hexane, 1.0 mL min^{-1}, λ = 210 nm) τ_R (major) = 19.5 min, τ_R (minor) = 21.2 min.

__Racemic__

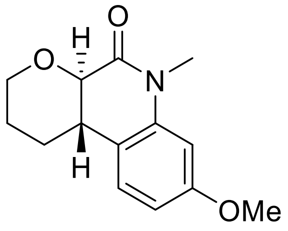

(*rac*)-**13**

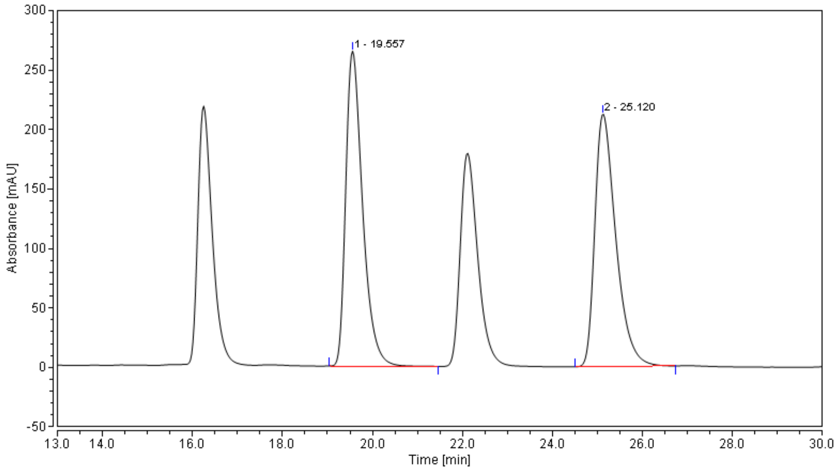

No.	Retention Time (min)	Area (mAU*min)	Height (mAU)	Relative Area (%)
1	19.557	114.771	265.069	50.10
2	25.120	114.305	212.464	49.90
**Total:**		229.076	477.533	100.00

__Enantioselective__

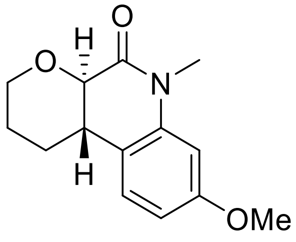

13

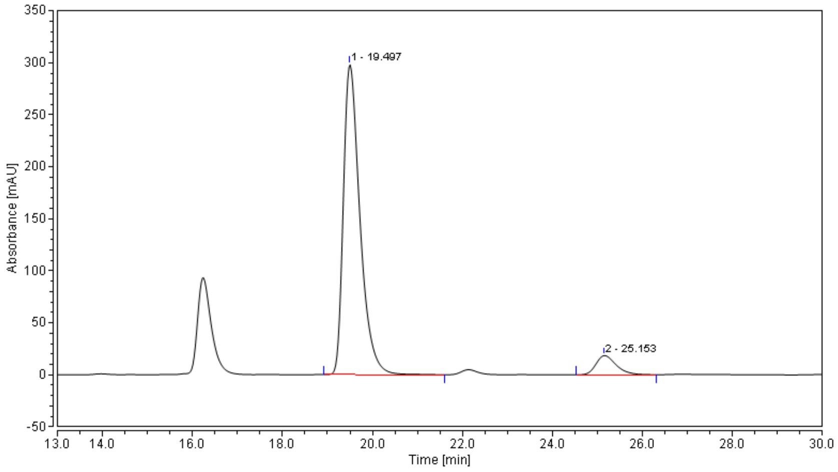

No.	Retention Time (min)	Area (mAU*min)	Height (mAU)	Relative Area (%)
1	19.497	127.178	298.120	92.99
2	25.153	9.584	18.427	7.01
**Total:**		136.762	316.547	100.00

(4a*R*,10b*S*)-10-methoxy-6-methyl-2,3,6,10b-tetrahydro-1*H*-pyrano[2,3-*c*]quinolin-5(4a*H*)-one (13b)

Chiral HPLC: (Chiralpak IB N-3, 20% ^{i}PrOH, 80% hexane, 1.0 mL min^{-1}, λ = 210 nm) τ_R (major) = 16.2 min, τ_R (minor) = 22.1 min.

Racemic

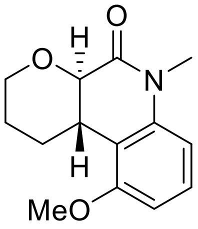

(*rac*)-**13b**

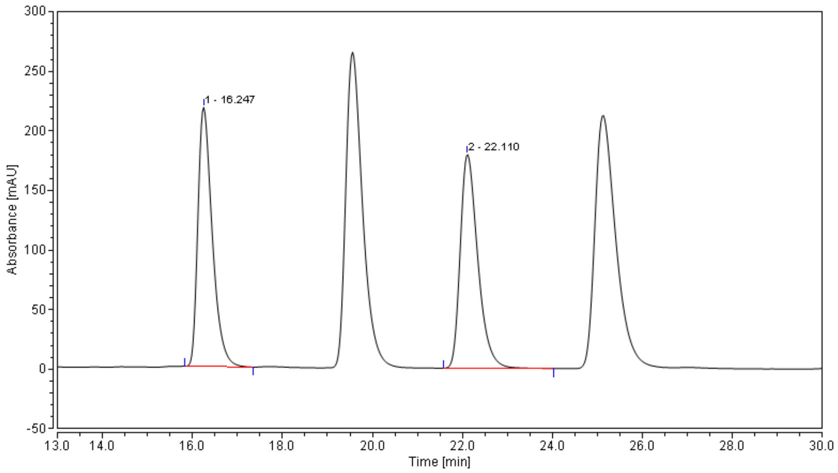

No.	Retention Time (min)	Area (mAU*min)	Height (mAU)	Relative Area (%)
1	16.247	80.269	217.656	49.79
2	22.110	80.960	179.626	50.21
**Total:**		161.229	397.282	100.00

Enantioselective

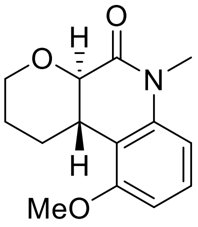

13b

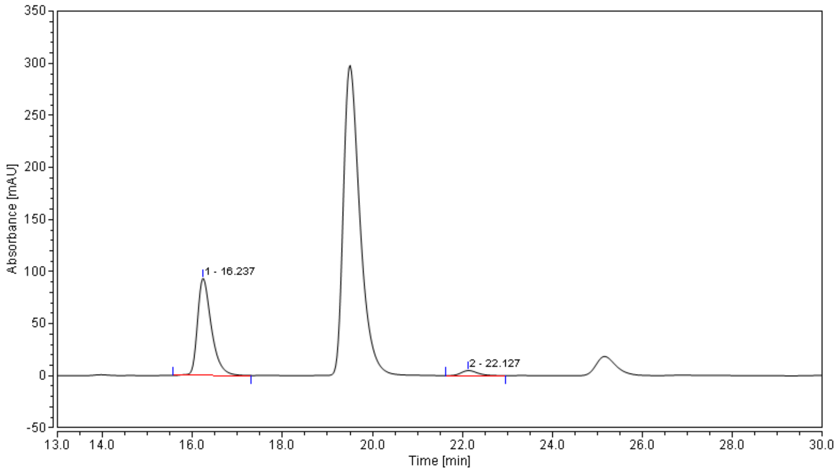

No.	Retention Time (min)	Area (mAU*min)	Height (mAU)	Relative Area (%)
1	16.237	33.250	93.335	93.90
2	22.127	2.161	4.953	6.10
**Total:**		35.411	98.289	100.00

(4a*R*,10b*R*)-8-methoxy-6-methyl-2,3,6,10b-tetrahydro-1*H*-pyrano[2,3-*c*]quinolin-5(4a*H*)-one (13b')

Chiral HPLC: (Chiralpak IB N-3, 15% ^{i}PrOH, 85% hexane, 1.0 mL min^{-1}, λ = 222 nm) τ_R (major) = 12.4 min, τ_R (minor) = 24.9 min.

Racemic

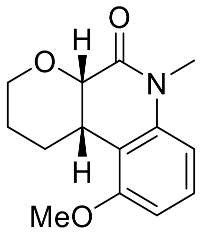

(*rac*)-**13b'**

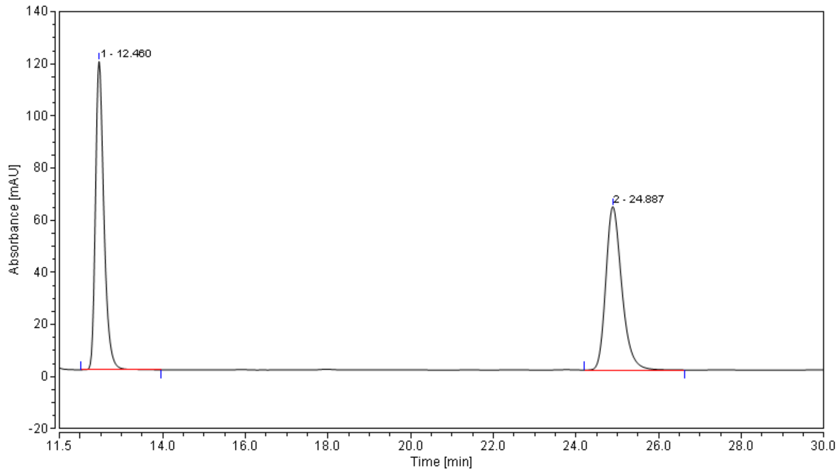

No.	Retention Time (min)	Area (mAU*min)	Height (mAU)	Relative Area (%)
1	12.460	28.070	118.343	49.99
2	24.887	28.086	62.740	50.01
**Total:**		56.156	181.083	100.00

Enantioselective

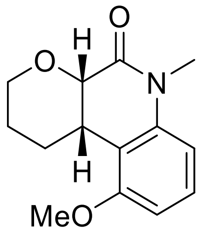

13b'

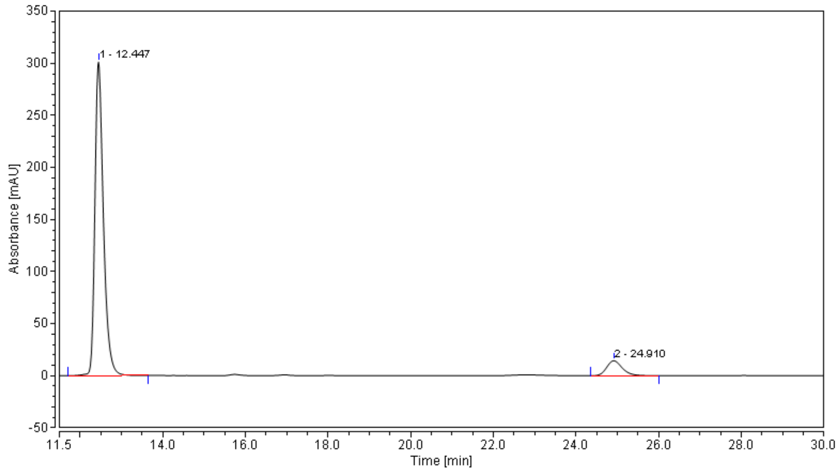

No.	Retention Time (min)	Area (mAU*min)	Height (mAU)	Relative Area (%)
1	12.447	71.994	301.301	91.87
2	24.910	6.374	14.437	8.13
**Total:**		78.369	315.738	100.00

(4a*R*,10b*S*)-6,8-Dimethyl-2,3,6,10b-tetrahydro-1*H*-pyrano[2,3-*c*]quinolin-5(4a*H*)-one (14), and (4a*R*,10b*S*)-6,10-dimethyl-2,3,6,10b-tetrahydro-1*H*-pyrano[2,3-*c*]quinolin-5(4a*H*) (14b')

(4a*R*,10b*S*)-6,8-Dimethyl-2,3,6,10b-tetrahydro-1*H*-pyrano[2,3-*c*]quinolin-5(4a*H*)-one (14)

Chiral HPLC: (Chiralpak IG-3, 20% ^{i}PrOH, 80% hexane, 1.0 mL min^{-1}, λ = 210 nm) τ_R (major) = 18.6 min, τ_R (minor) = 21.4 min.

<u>**Racemic**</u>

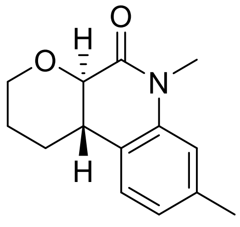

(*rac*)-**14**

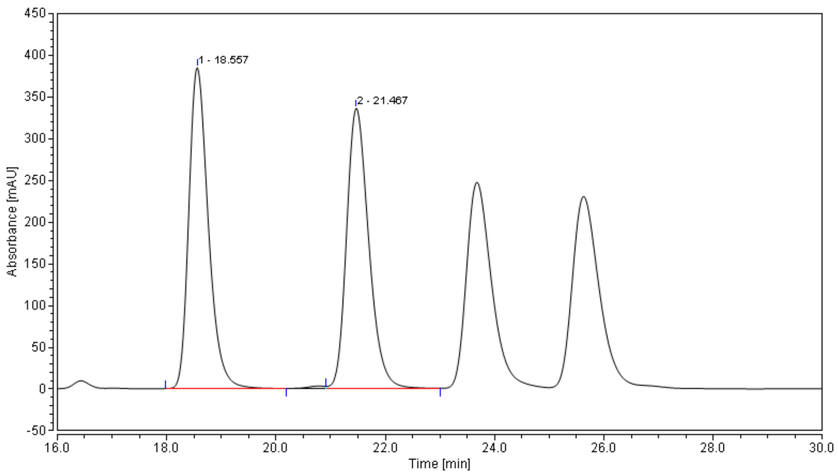

No.	Retention Time (min)	Area (mAU*min)	Height (mAU)	Relative Area (%)
1	18.557	159.021	385.017	50.15
2	21.467	158.075	336.339	49.85
**Total:**		317.096	721.357	100.00

<u>**Enantioselective**</u>

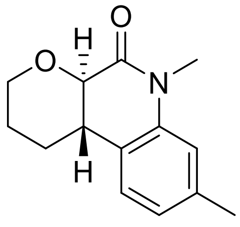

14

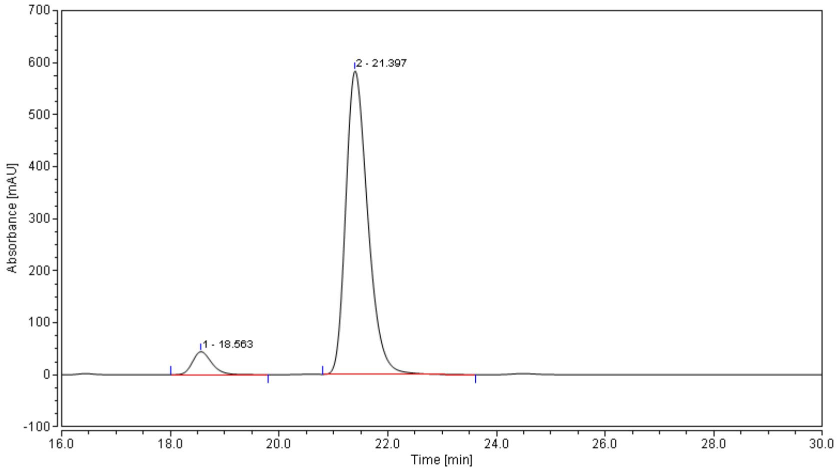

No.	Retention Time (min)	Area (mAU*min)	Height (mAU)	Relative Area (%)
1	18.563	18.138	44.307	6.20
2	21.397	274.554	583.765	93.80
**Total:**		292.692	628.072	100.00

(4a*R*,10b*S*)-6,8-Dimethyl-2,3,6,10b-tetrahydro-1*H*-pyrano[2,3-*c*]quinolin-5(4a*H*)-one (14b')

Chiral HPLC: (Chiralpak IB N-3, 15% ^{i}PrOH, 85% hexane, 1.0 mL min^{-1}, λ = 222 nm) τ_R (major) = 19.0 min, τ_R (minor) = 23.3 min..

Racemic

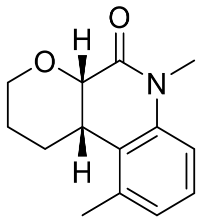

(*rac*)-**14b'**

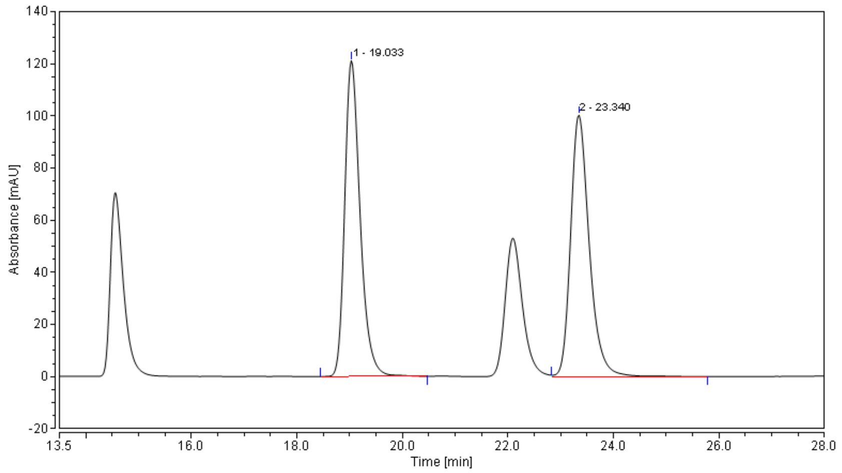

No.	Retention Time (min)	Area (mAU*min)	Height (mAU)	Relative Area (%)
1	19.033	41.036	121.263	49.97
2	23.340	41.084	100.422	50.03
**Total:**		82.120	221.685	100.00

Enantioselective

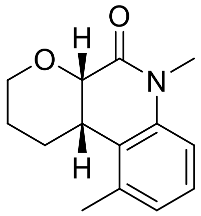

14b'

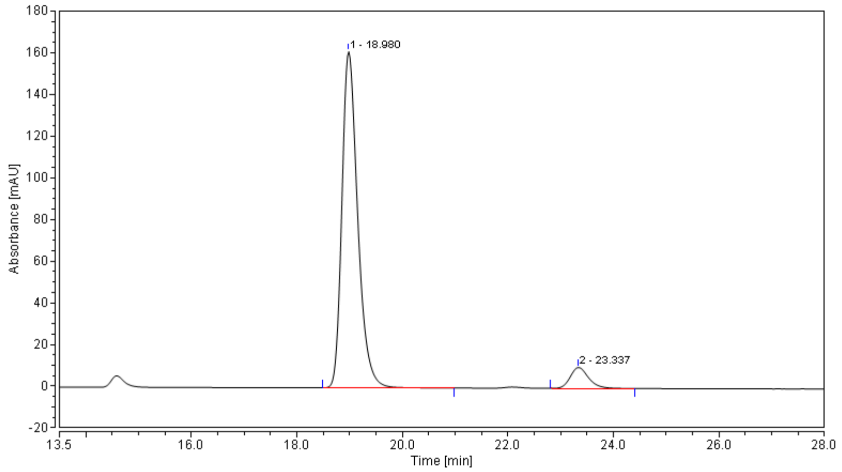

No.	Retention Time (min)	Area (mAU*min)	Height (mAU)	Relative Area (%)
1	18.980	55.884	161.512	93.21
2	23.337	4.068	10.082	6.79
**Total:**		59.952	171.594	100.00

(4a*R*,10b*S*)-8,10-Difluoro-6-methyl-2,3,6,10b-tetrahydro-1*H*-pyrano[2,3-*c*]quinolin-5(4a*H*)-one (15) and (4a*S*,10b*S*)-8,10-difluoro-6-methyl-2,3,6,10b-tetrahydro-1*H*-pyrano[2,3-*c*]quinolin-5(4a*H*)-one (15')

(4a*R*,10b*S*)-8,10-Difluoro-6-methyl-2,3,6,10b-tetrahydro-1*H*-pyrano[2,3-*c*]quinolin-5(4a*H*)-one (15)

Chiral HPLC: (Chiralpak OD-H, 30% ^{i}PrOH, 70% hexane, 1.0 mL min^{-1}, λ = 260 nm) τ_R (major) = 14.6 min, τ_R (minor) = 21.9 min.

<u>Racemic</u>

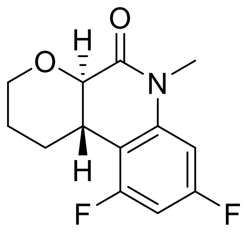

(*rac*)-**15**

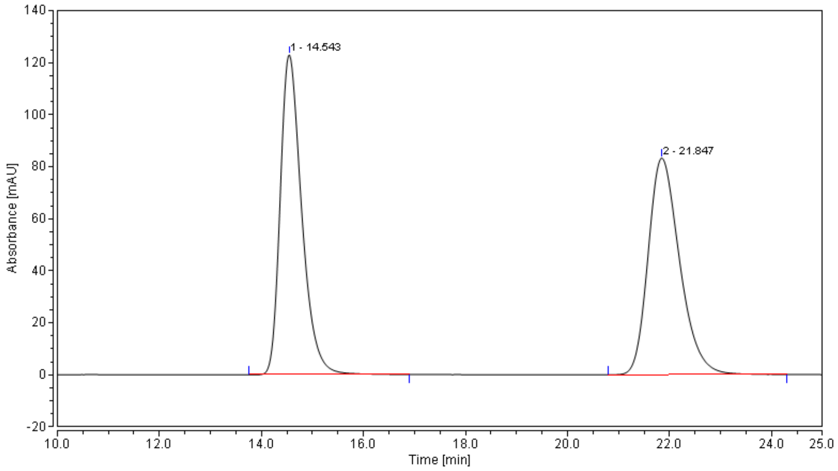

No.	Retention Time (min)	Area (mAU*min)	Height (mAU)	Relative Area (%)
1	14.543	60.019	123.074	50.15
2	21.847	59.665	83.344	49.85
**Total:**		119.684	206.418	100.00

<u>Enantioselective</u>

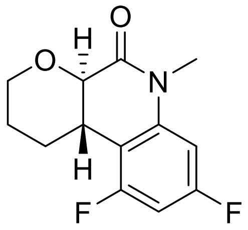

15

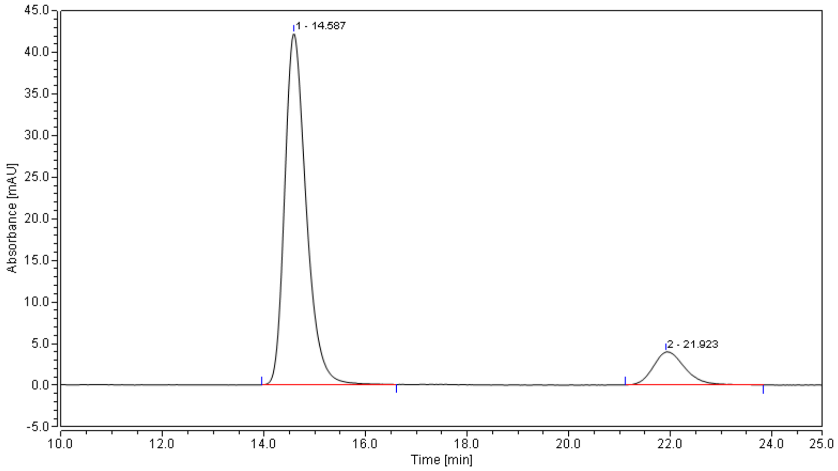

No.	Retention Time (min)	Area (mAU*min)	Height (mAU)	Relative Area (%)
1	14.587	20.746	42.220	87.73
2	21.923	2.902	3.989	12.27
**Total:**		23.647	46.210	100.00

(4a*S*,10b*S*)-8,10-Difluoro-6-methyl-2,3,6,10b-tetrahydro-1*H*-pyrano[2,3-*c*]quinolin-5(4a*H*)-one (15’)

Chiral HPLC: (Chiralpak OD-H, 30% ^{i}PrOH, 70% hexane, 1.0 mL min^{-1}, λ = 260 nm) τ_R (major) = 9.8 min, τ_R (minor) = 15.0 min.

Racemic

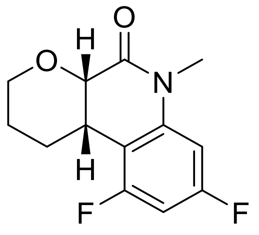

(rac)-**15'**

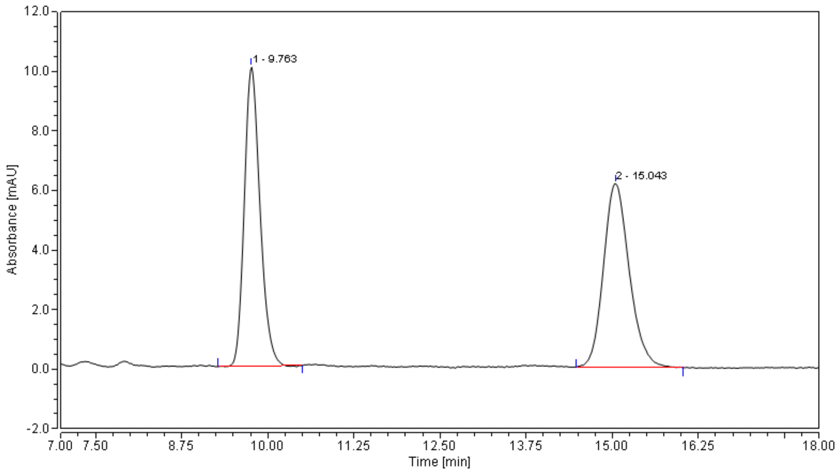

No.	Retention Time (min)	Area (mAU*min)	Height (mAU)	Relative Area (%)
1	9.763	2.659	10.054	49.73
2	15.043	2.689	6.183	50.27
**Total:**		5.348	16.237	100.00

Enantioselective

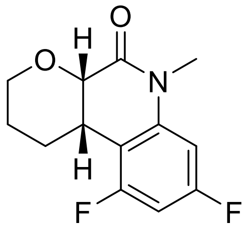

15'

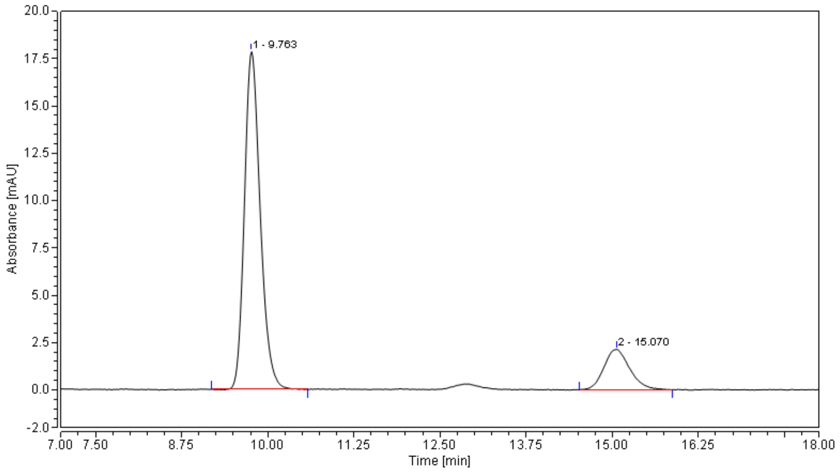

No.	Retention Time (min)	Area (mAU*min)	Height (mAU)	Relative Area (%)
1	9.763	4.746	17.842	83.52
2	15.070	0.936	2.145	16.48
**Total:**		5.683	19.987	100.00

(4a*R*,10b*S*)-9-Fluoro-6-methyl-2,3,6,10b-tetrahydro-1*H*-pyrano[2,3-c]quinolin-5(4a*H*)-one (16)

Chiral HPLC: (Chiralpak OD-H, 30% ^{i}PrOH, 70% hexane, 1.0 mL min^{-1}, λ = 260 nm) τ_R (major) = 12.8 min, τ_R (minor) = 17.9 min.

Racemic

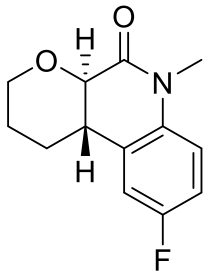

(rac)-**16**

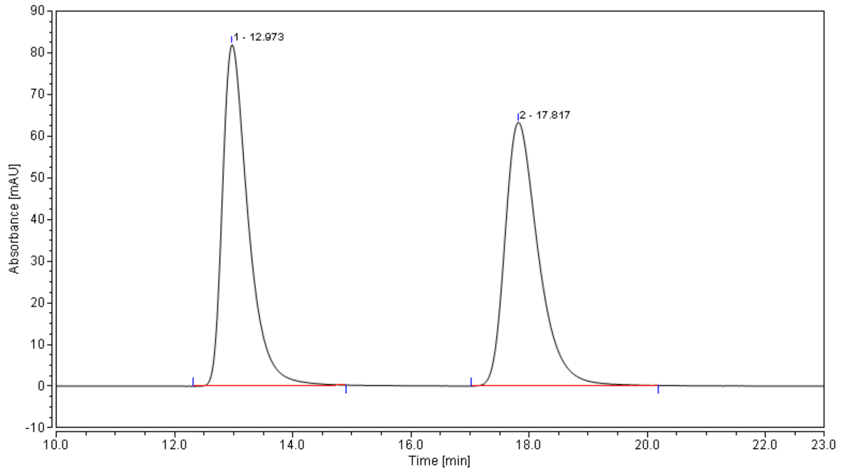

No.	Retention Time (min)	Area (mAU*min)	Height (mAU)	Relative Area (%)
1	12.973	41.206	81.953	49.91
2	17.817	41.357	63.287	50.09
**Total:**		82.563	145.241	100.00

Enantioselective

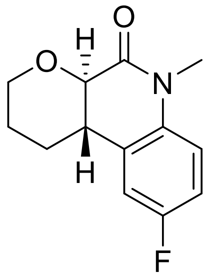

16

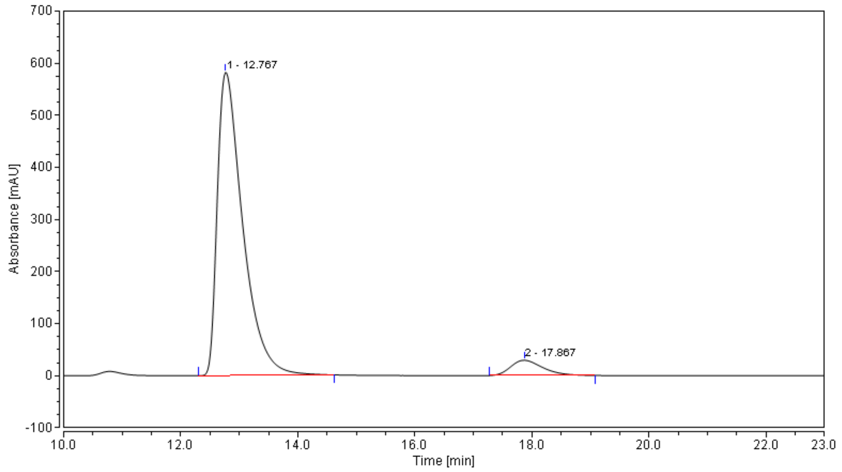

No.	Retention Time (min)	Area (mAU*min)	Height (mAU)	Relative Area (%)
1	12.767	292.724	582.854	94.00
2	17.867	18.694	29.350	6.00
**Total:**		311.418	612.204	100.00

(4a*R*,10b*S*)-6-Benzyl-9-chloro-2,3,6,10b-tetrahydro-1*H*-pyrano[2,3-c]quinolin-5(4a*H*)-one (17)

Chiral HPLC: (Chiralpak IF-3, 50% ^{i}PrOH, 50% hexane, 1.0 mL min^{-1}, λ = 210 nm) τ_R (major) = 14.7 min, τ_R (minor) = 18.5 min.

Racemic

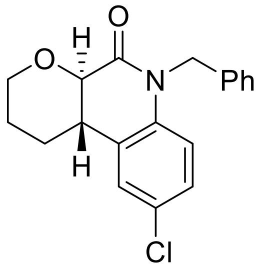

(*rac*)-**17**

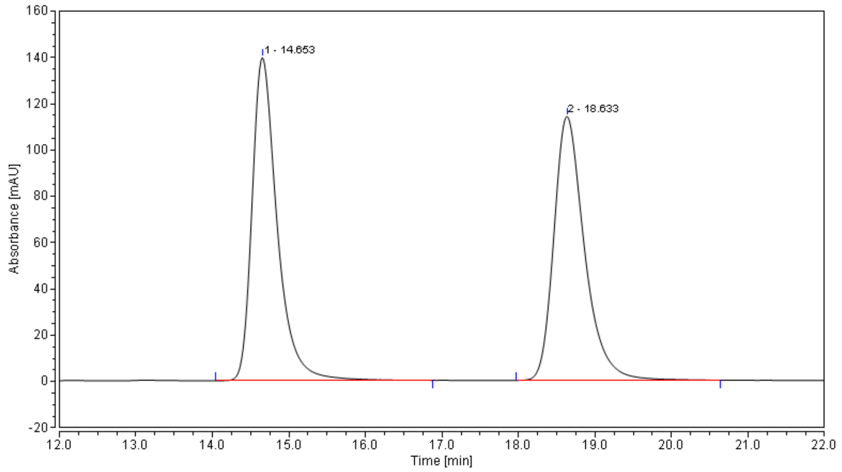

No.	Retention Time (min)	Area (mAU*min)	Height (mAU)	Relative Area (%)
1	14.653	53.151	139.649	50.11
2	18.633	52.919	114.096	49.89
**Total:**		106.071	253.746	100.00

Enantioselective

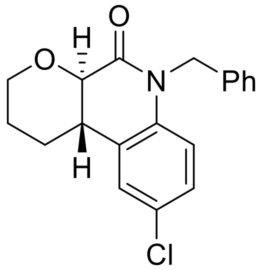

17

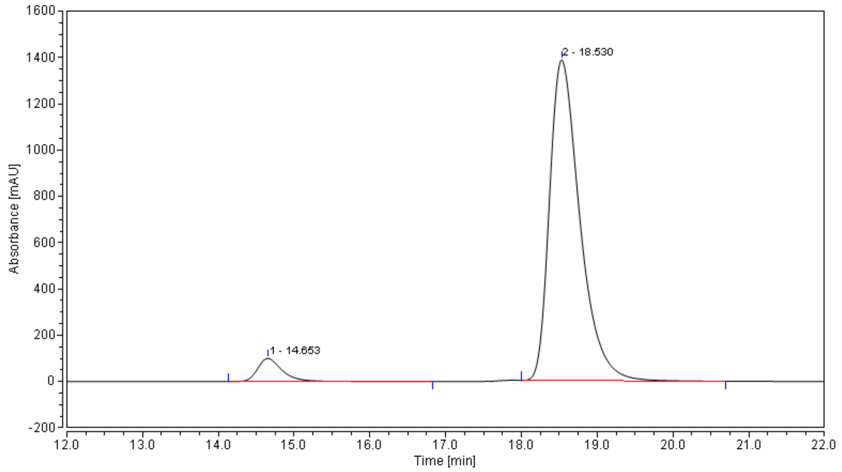

No.	Retention Time (min)	Area (mAU*min)	Height (mAU)	Relative Area (%)
1	14.653	37.916	99.892	5.58
2	18.530	641.915	1385.648	94.42
**Total:**		679.831	1485.540	100.00

(4a*R*,10b*S*)-9-Bromo-6-methyl-2,3,6,10b-tetrahydro-1*H*-pyrano[2,3-*c*]quinolin-5(4a*H*)-one (18)

Chiral HPLC: (Chiralpak OD-H, 30% ^{i}PrOH, 70% hexane, 1.0 mL min^{-1}, λ = 254 nm) τ_R (major) = 15.9 min, τ_R (minor) = 22.1 min.

Racemic

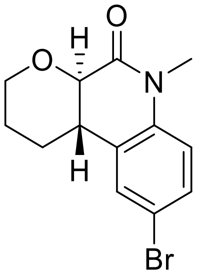

(*rac*)-**18**

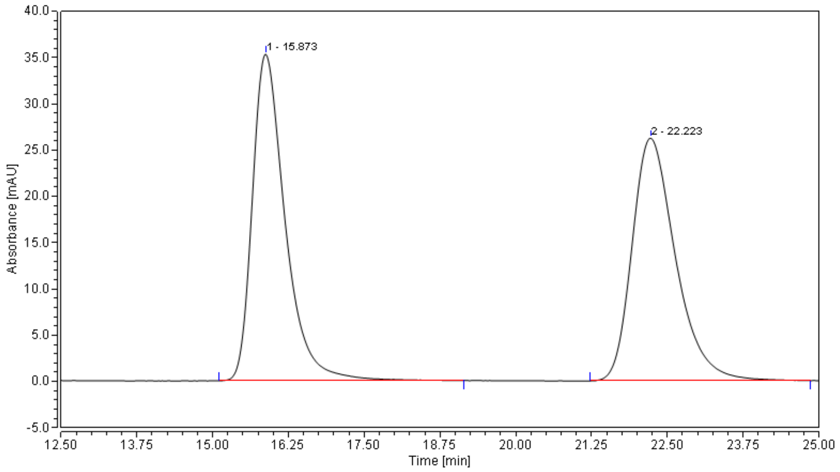

No.	Retention Time (min)	Area (mAU*min)	Height (mAU)	Relative Area (%)
1	15.873	22.118	35.283	50.05
2	22.223	21.788	26.215	49.30
**Total:**		44.190	62.071	100.00

Enantioselective

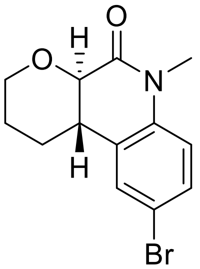

18

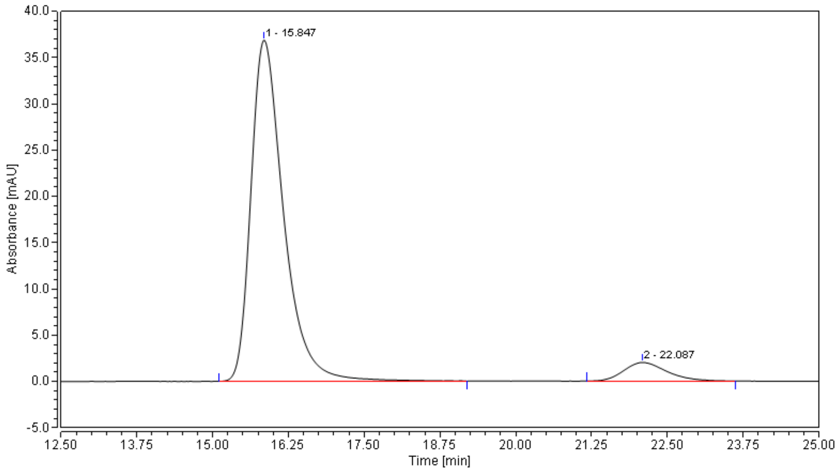

No.	Retention Time (min)	Area (mAU*min)	Height (mAU)	Relative Area (%)
1	15.847	23.017	36.865	93.22
2	22.087	1.674	2.019	6.78
**Total:**		24.691	38.884	100.00

(4a*R*,10b*S*)-6,9-Dimethyl-2,3,6,10b-tetrahydro-1*H*-pyrano[2,3-c]quinolin-5(4a*H*)-one (19)

Chiral HPLC: (Chiralpak OD-H, 30% ^{i}PrOH, 70% hexane, 1.0 mL min^{-1}, λ = 260 nm) τ_R (major) = 9.8 min, τ_R (minor) = 14.4 min.

Racemic

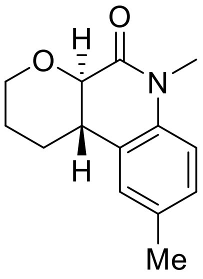

(rac)-**19**

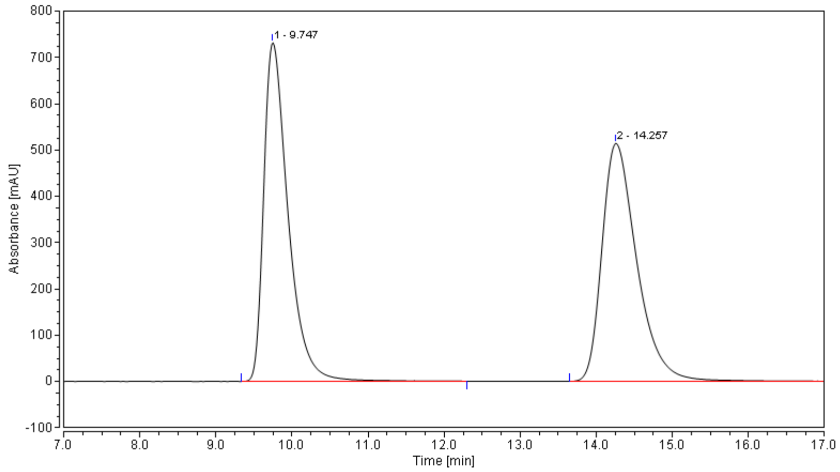

No.	Retention Time (min)	Area (mAU*min)	Height (mAU)	Relative Area (%)
1	9.747	271.226	732.107	49.89
2	14.257	272.409	514.365	50.11
**Total:**		543.634	1246.472	100.00

Enantioselective

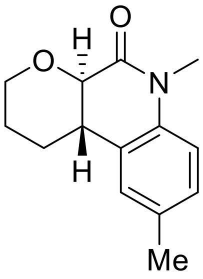

19

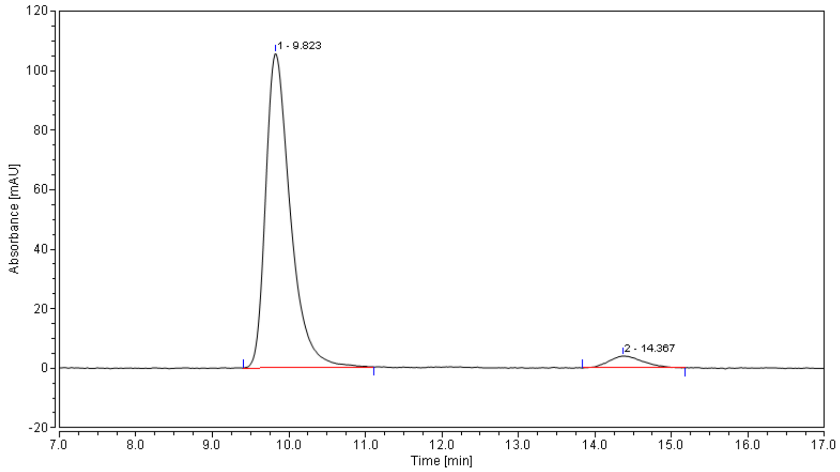

No.	Retention Time (min)	Area (mAU*min)	Height (mAU)	Relative Area (%)
1	9.823	39.541	105.756	94.85
2	14.367	2.147	4.016	5.15
**Total:**		41.688	109.772	100.00

(4a*R*,10b*S*)-6-Methyl-9-(trifluoromethyl)-2,3,6,10b-tetrahydro-1*H*-pyrano[2,3-*c*]quinolin-5(4a*H*)-one (20)

Chiral HPLC: (Chiralpak OD-H, 30% ^{i}PrOH, 70% hexane, 1.0 mL min^{-1}, λ = 210 nm) τ_R (major) = 14.2 min, τ_R (minor) = 19.4 min.

Racemic

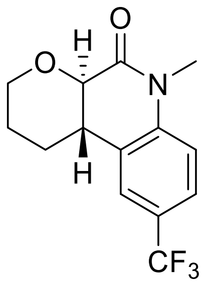

(*rac*)-**20**

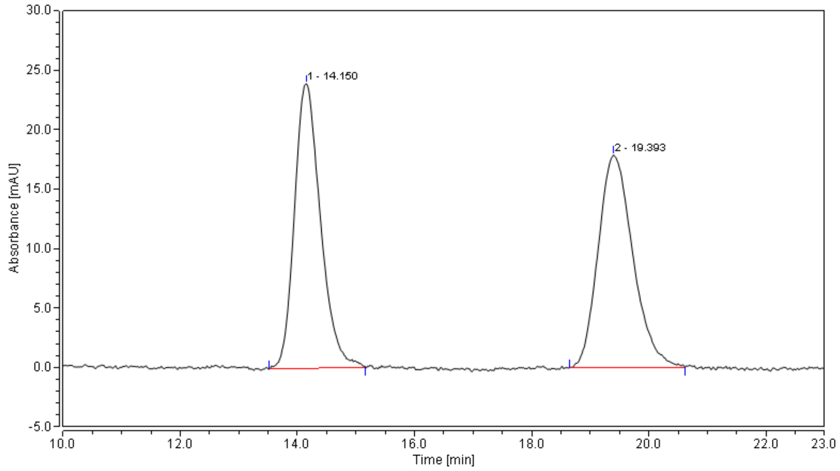

No.	Retention Time (min)	Area (mAU*min)	Height (mAU)	Relative Area (%)
1	14.150	12.348	24.000	50.10
2	19.393	12.301	17.913	49.90
**Total:**		24.649	41.913	100.00

Enantioselective

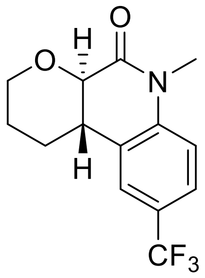

20

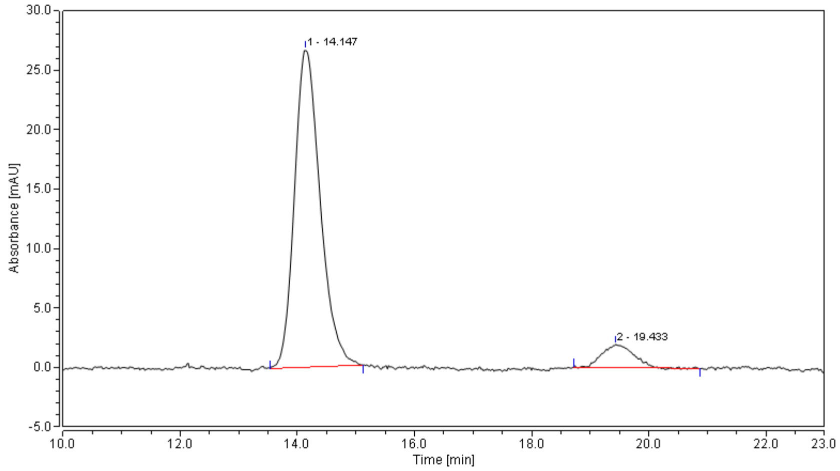

No.	Retention Time (min)	Area (mAU*min)	Height (mAU)	Relative Area (%)
1	14.147	13.590	26.717	91.82
2	19.433	1.211	1.988	8.18
**Total:**		14.801	28.705	100.00

(4a*R*,10b*S*)-9-(*Tert*-butyl)-6-methyl-2,3,6,10b-tetrahydro-1*H*-pyrano[2,3-c]quinolin-5(4a*H*)-one (21)

Chiral HPLC: (Chiralpak OD-H, 30% ^{i}PrOH, 70% hexane, 1.0 mL min^{-1}, λ = 270 nm) τ_R (major) = 9.8 min, τ_R (minor) = 13.2 min.

Racemic

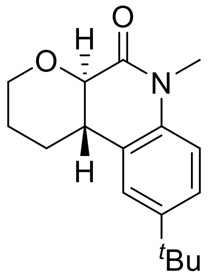

(*rac*)-**21**

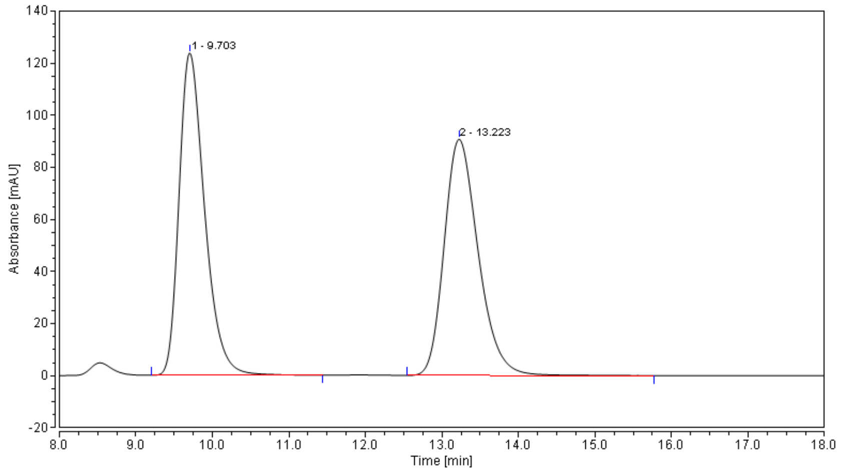

No.	Retention Time (min)	Area (mAU*min)	Height (mAU)	Relative Area (%)
1	9.703	47.894	123.868	50.61
2	13.223	46.748	90.741	49.39
**Total:**		94.642	214.609	100.00

Enantioselective

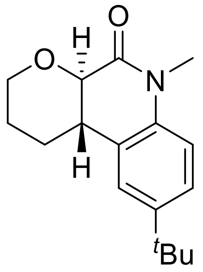

21

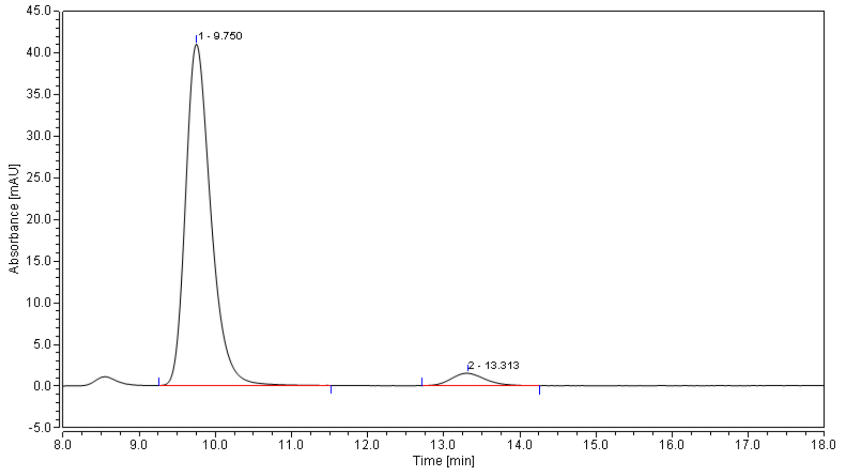

No.	Retention Time (min)	Area (mAU*min)	Height (mAU)	Relative Area (%)
1	9.750	15.681	41.024	95.28
2	13.313	0.776	1.497	4.72
**Total:**		16.457	42.521	100.00

(4a*R*,10b*S*)-9-Methoxy-6-methyl-2,3,6,10b-tetrahydro-1*H*-pyrano[2,3-*c*]quinolin-5(4a*H*)-one (22)

Chiral HPLC: (Chiralpak OD-H, 30% ^{i}PrOH, 70% hexane, 1.0 mL min^{-1}, λ = 260 nm) τ_R (major) = 14.0 min, τ_R (minor) = 19.4 min.

Racemic

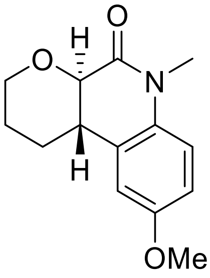

(rac)-**22**

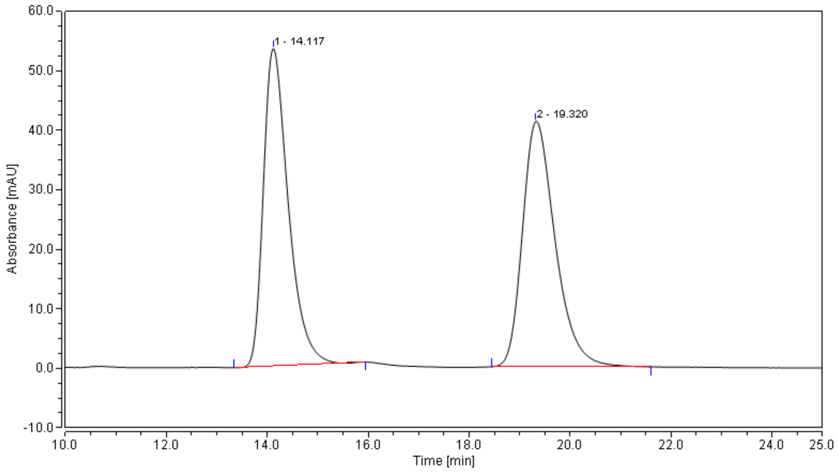

No.	Retention Time (min)	Area (mAU*min)	Height (mAU)	Relative Area (%)
1	14.117	30.411	53.345	49.58
2	19.320	30.931	41.269	50.42
**Total:**		61.342	94.613	100.00

Enantioselective

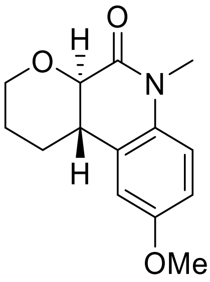

22

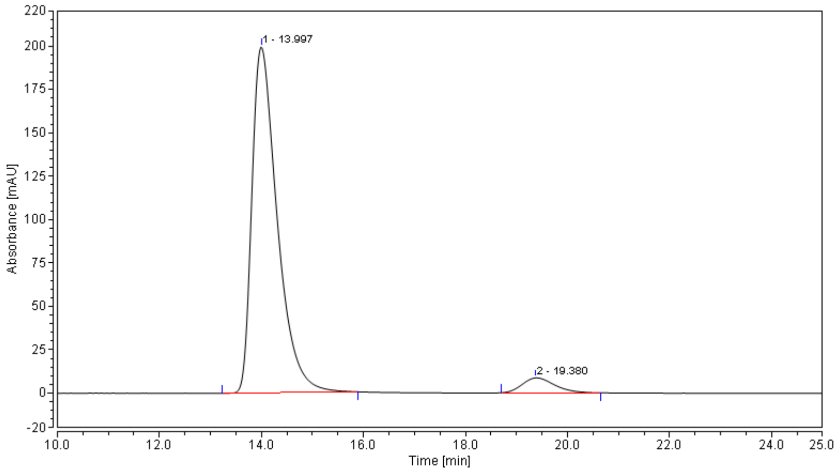

No.	Retention Time (min)	Area (mAU*min)	Height (mAU)	Relative Area (%)
1	13.997	114.556	199.362	94.82
2	19.380	6.262	8.573	5.18
**Total:**		120.819	207.935	100.00

(4a*R*,10b*S*)-9-(Methylthio)-6-methyl-2,3,6,10b-tetrahydro-1*H*-pyrano[2,3-*c*]quinolin-5(4aH)-one (23)

Chiral HPLC: (Chiralpak OD-H, 30% ^{i}PrOH, 70% hexane, 1.0 mL min^{-1}, λ = 260 nm) τ_R (major) = 16.6 min, τ_R (minor) = 22.5 min.

Racemic

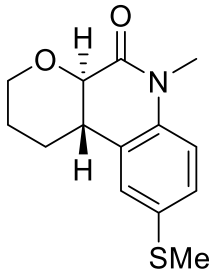

(rac)-**23**

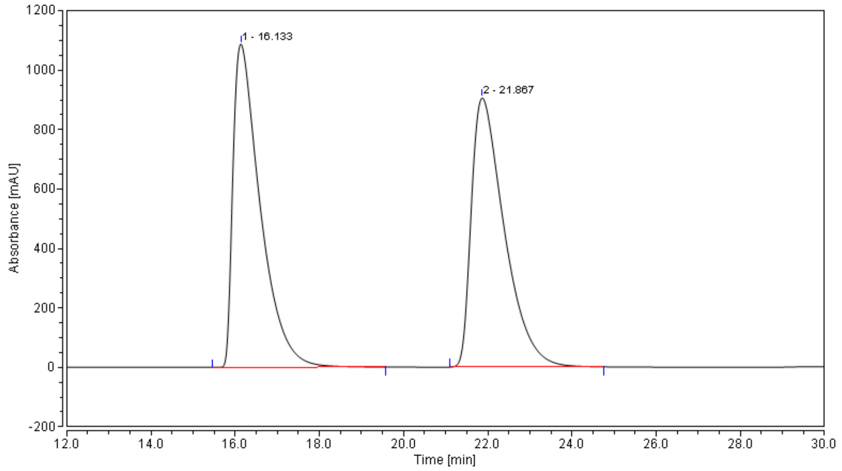

No.	Retention Time (min)	Area (mAU*min)	Height (mAU)	Relative Area (%)
1	16.133	815.466	1088.209	49.86
2	21.867	819.921	905.823	50.14
**Total:**		1635.387	1994.032	100.00

Enantioselective

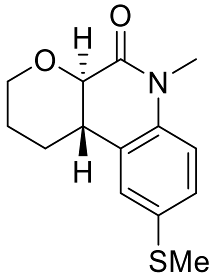

23

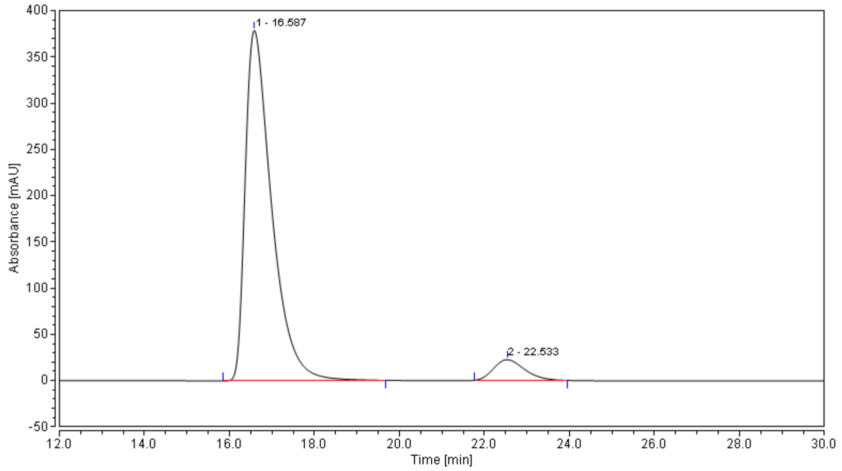

No.	Retention Time (min)	Area (mAU*min)	Height (mAU)	Relative Area (%)
1	16.587	272.414	379.134	93.54
2	22.533	18.804	22.309	6.46
**Total:**		291.218	401.443	100.00

(3a*R*,9b*S*)-5-Methyl-1,2,5,9b-tetrahydrofuro[2,3-c]quinolin-4(3a*H*)-one (24)

Chiral HPLC: (Chiralpak IC, 40% ^{i}PrOH, 60% hexane, 1.0 mL min^{-1}, λ = 260 nm) τ_R (major) = 51.5 min, τ_R (minor) = 55.8 min.

Racemic

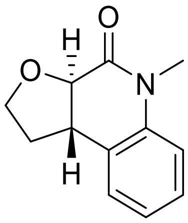

(rac)-**24**

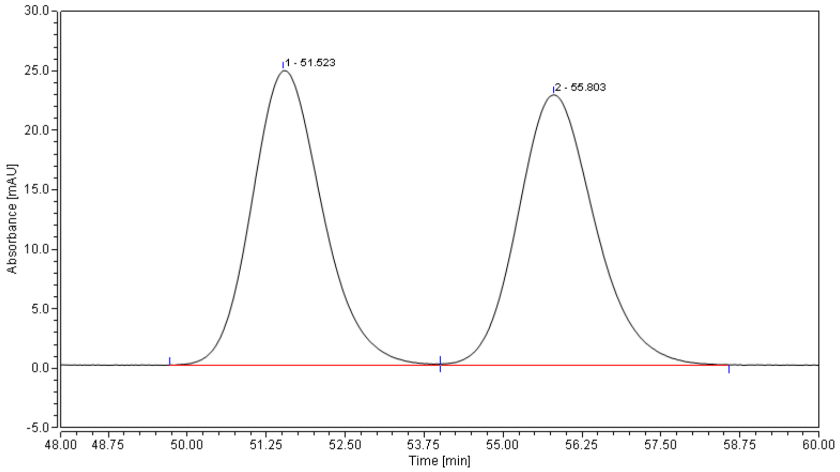

No.	Retention Time (min)	Area (mAU*min)	Height (mAU)	Relative Area (%)
1	51.523	32.102	24.753	50.02
2	55.803	32.081	22.712	49.98
**Total:**		64.183	47.465	100.00

Enantioselective

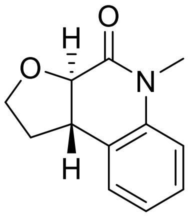

24

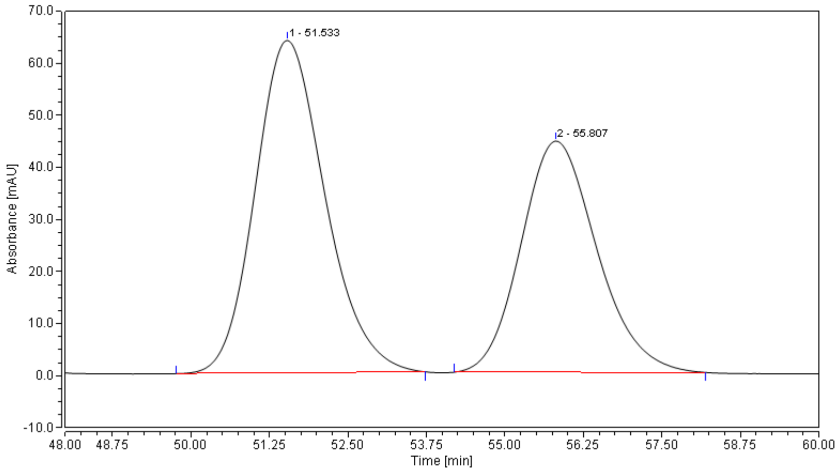

No.	Retention Time (min)	Area (mAU*min)	Height (mAU)	Relative Area (%)
1	51.533	82.285	63.836	57.01
2	55.807	62.044	44.401	42.99
**Total:**		144.328	108.236	100.00

(4a*R*,10b*S*)-6-Methyl-2,3,6,10b-tetrahydro-1*H*-pyrano[2,3-*c*]quinolin-5(4a*H*)-one-4a,7,8,9,10-d_5

Chiral HPLC: (Chiralpak IB N-3, 30% ^{i}PrOH, 70% hexane, 1.0 mL min^{-1}, λ = 210 nm) τ_R (major) = 11.1 min, τ_R (minor) = 16.6 min.

Racemic

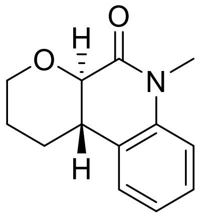

(rac)-**2**

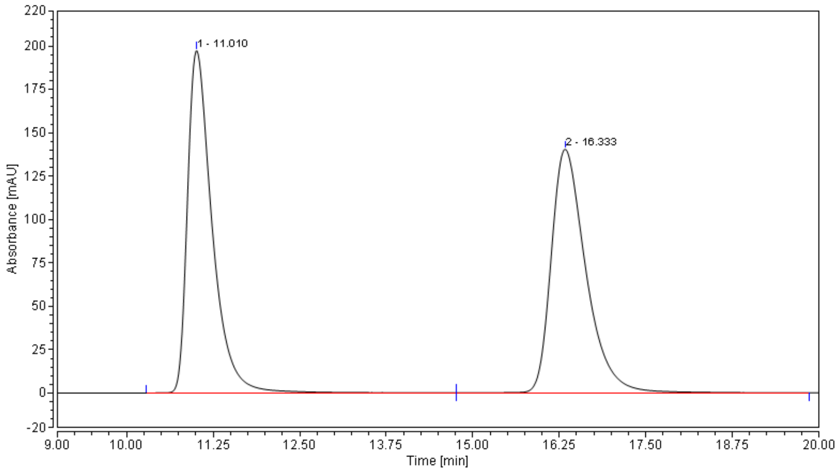

No.	Retention Time (min)	Area (mAU*min)	Height (mAU)	Relative Area (%)
1	11.010	81.682	197.420	50.00
2	16.333	81.678	140.439	50.00
**Total:**		163.360	337.859	100.00

Enantioselective

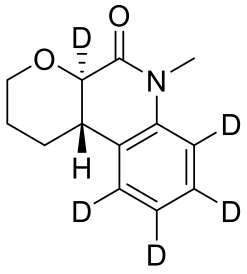

30

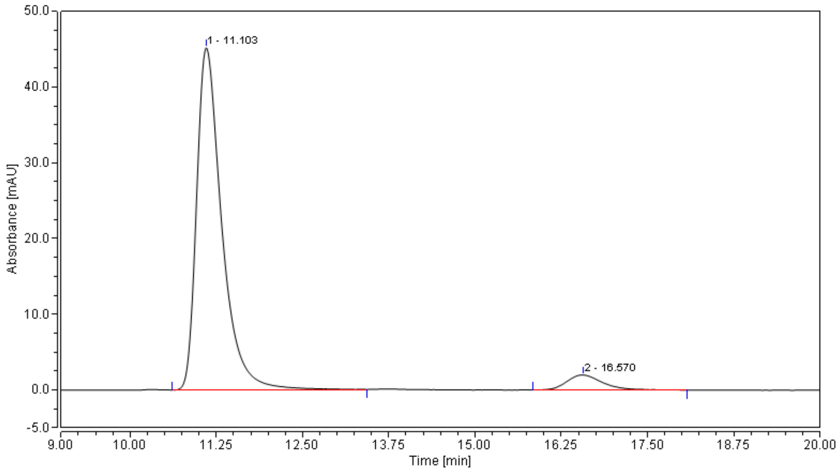

No.	Retention Time (min)	Area (mAU*min)	Height (mAU)	Relative Area (%)
1	11.103	19.074	45.196	94.06
2	16.570	1.204	1.987	5.94
**Total:**		20.278	47.182	100.00

(4a*R*,10b*S*)-6-Ethyl-2,3,6,10b-tetrahydro-1*H*-pyrano[2,3-c]quinolin-5(4a*H*)-one (S1)

Chiral HPLC: (Chiralpak OD-H, 30% ^{i}PrOH, 70% hexane, 1.0 mL min^{-1}, λ = 260 nm) τ_R (major) = 8.6 min, τ_R (minor) = 12.4 min.

Racemic

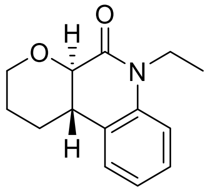

(*rac*)-**S1**

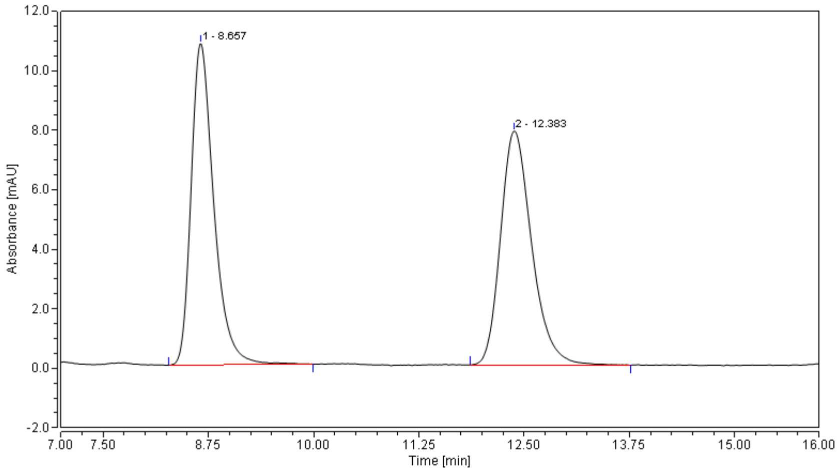

No.	Retention Time (min)	Area (mAU*min)	Height (mAU)	Relative Area (%)
1	8.657	3.388	10.813	50.07
2	12.383	3.378	7.866	49.93
**Total:**		6.766	18.679	100.00

Enantioselective

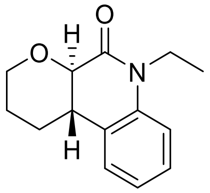

S1

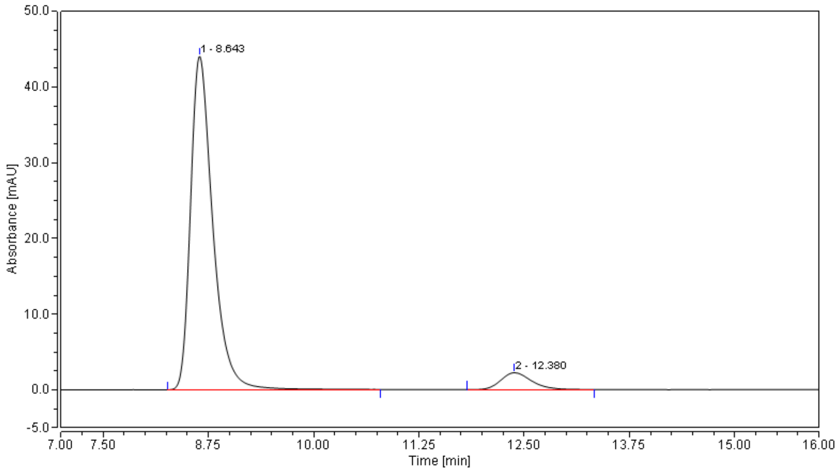

No.	Retention Time (min)	Area (mAU*min)	Height (mAU)	Relative Area (%)
1	8.643	13.508	44.052	93.40
2	12.380	0.954	2.235	6.60
**Total:**		14.462	46.286	100.00

(4a*R*,10b*S*)-9-Chloro-6-methyl-2,3,6,10b-tetrahydro-1*H*-pyrano[2,3-c]quinolin-5(4a*H*)-one (S2)

Chiral HPLC: (Chiralpak OD-H, 30% ^{i}PrOH, 70% hexane, 1.0 mL min^{-1}, λ = 260 nm) τ_R (major) = 14.7 min, τ_R (minor) = 21.0 min.

Racemic

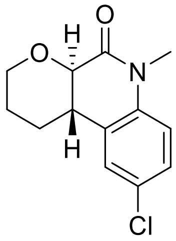

(*rac*)-**S2**

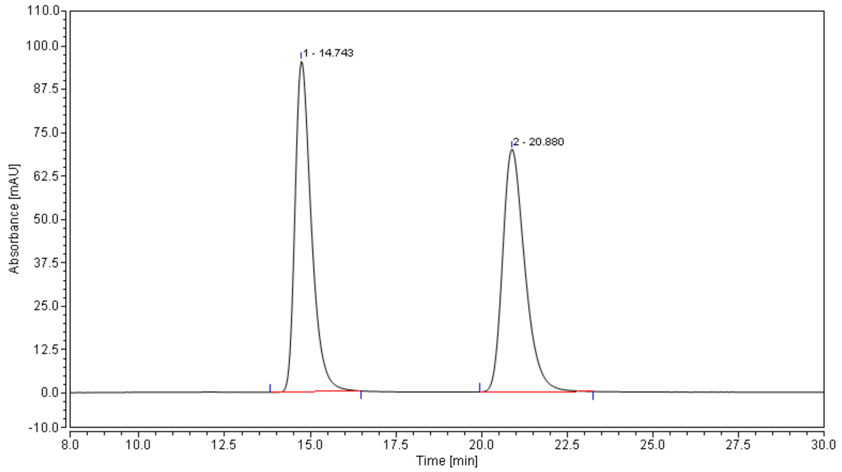

No.	Retention Time (min)	Area (mAU*min)	Height (mAU)	Relative Area (%)
1	14.743	52.469	95.311	49.92
2	20.880	52.628	69.932	50.08
**Total:**		105.096	165.243	100.00

Enantioselective

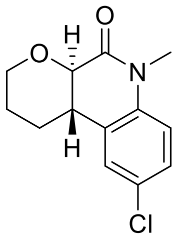

S2

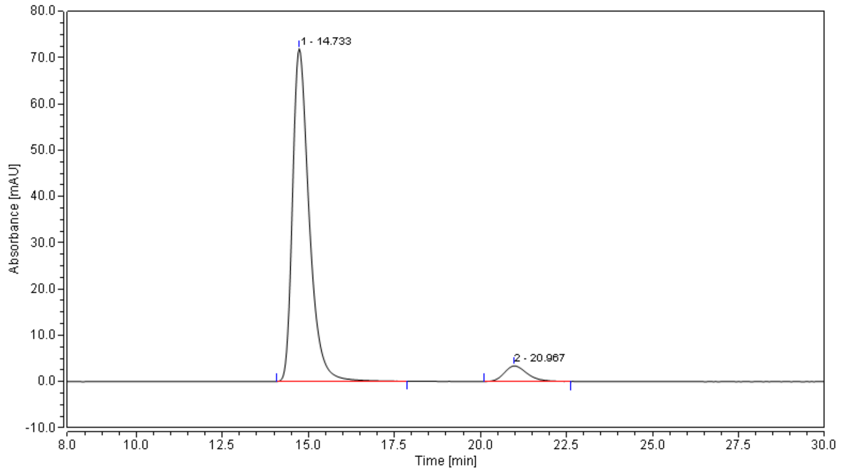

No.	Retention Time (min)	Area (mAU*min)	Height (mAU)	Relative Area (%)
1	14.733	40.330	71.954	93.92
2	20.967	2.609	3.373	6.08
**Total:**		42.939	75.328	100.00

8.1. HPLC Traces for Cyclisation of S3

Using 4

(5a*R*,11b*S*)-7-Methyl-1,2,3,4,7,11b-hexahydrooxepino[2,3-*c*]quinolin-6(5a*H*)-one (S3)

Chiral HPLC: (Chiralpak IG-3, 25% ^{i}PrOH, 75% hexane, 1.0 mL min^{-1}, λ = 210 nm) τ_R (major) = 16.5 min, τ_R (minor) = 17.7 min.

Racemic

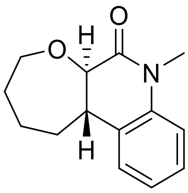

(*rac*)-**S3**

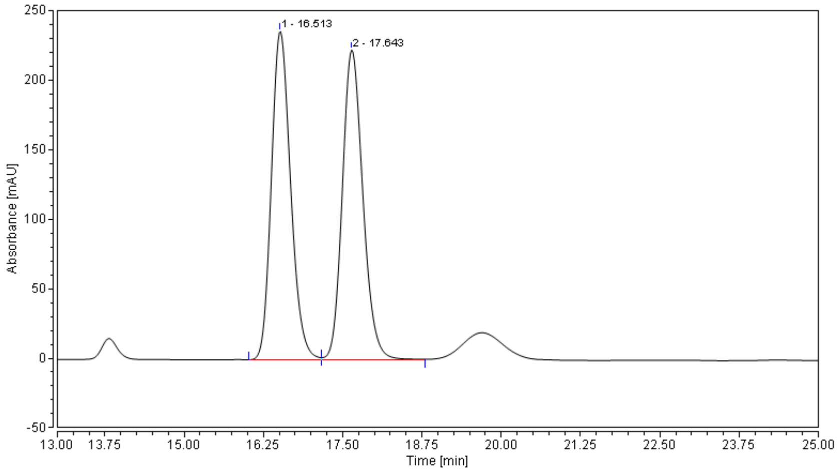

No.	Retention Time (min)	Area (mAU*min)	Height (mAU)	Relative Area (%)
1	16.513	83.015	236.480	49.88
2	17.643	83.399	222.767	50.12
**Total:**		166.414	459.247	100.00

Enantioselective

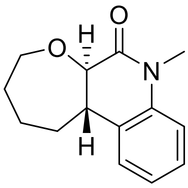

S3

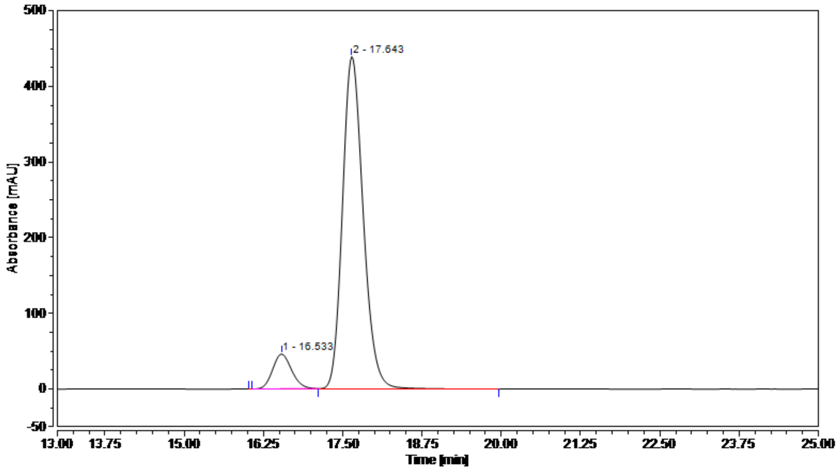

No.	Retention Time (min)	Area (mAU*min)	Height (mAU)	Relative Area (%)
1	16.533	15.893	45.898	8.71
2	17.643	166.666	439.575	91.29
**Total:**		182.559	485.473	100.00

Using Ir(Fppy)$_3$

(5a*R*,11b*S*)-7-Methyl-1,2,3,4,7,11b-hexahydrooxepino[2,3-*c*]quinolin-6(5a*H*)-one (S3)

Chiral HPLC: (Chiralpak IG-3, 25% ^{i}PrOH, 75% hexane, 1.0 mL min^{-1}, λ = 210 nm) τ_R (major) = 16.5 min, τ_R (minor) = 17.7 min.

Racemic

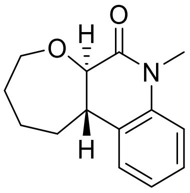

(*rac*)-**S3**

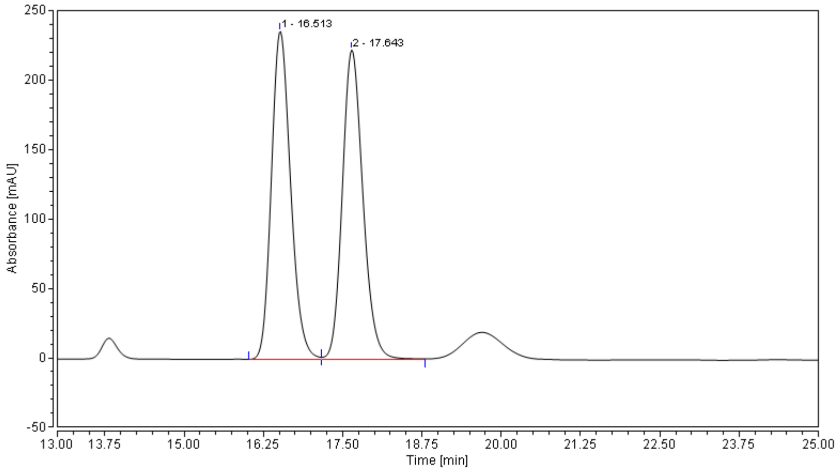

No.	Retention Time (min)	Area (mAU*min)	Height (mAU)	Relative Area (%)
1	16.513	83.015	236.480	49.88
2	17.643	83.399	222.767	50.12
**Total:**		166.414	459.247	100.00

Enantioselective

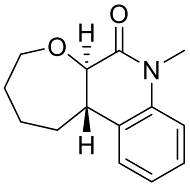

S3

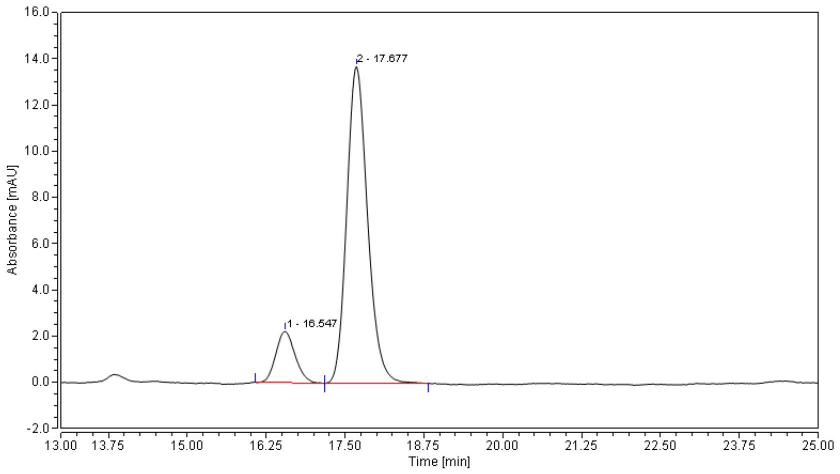

No.	Retention Time (min)	Area (mAU*min)	Height (mAU)	Relative Area (%)
1	16.547	0.789	2.214	13.01
2	17.677	5.271	13.718	86.99
**Total:**		6.060	15.932	100.0

9. NMR Spectra for Compounds

9.1. Photocatalysts and Phenylpyridines

5-Fluoro-2-phenylpyridine (PP1)

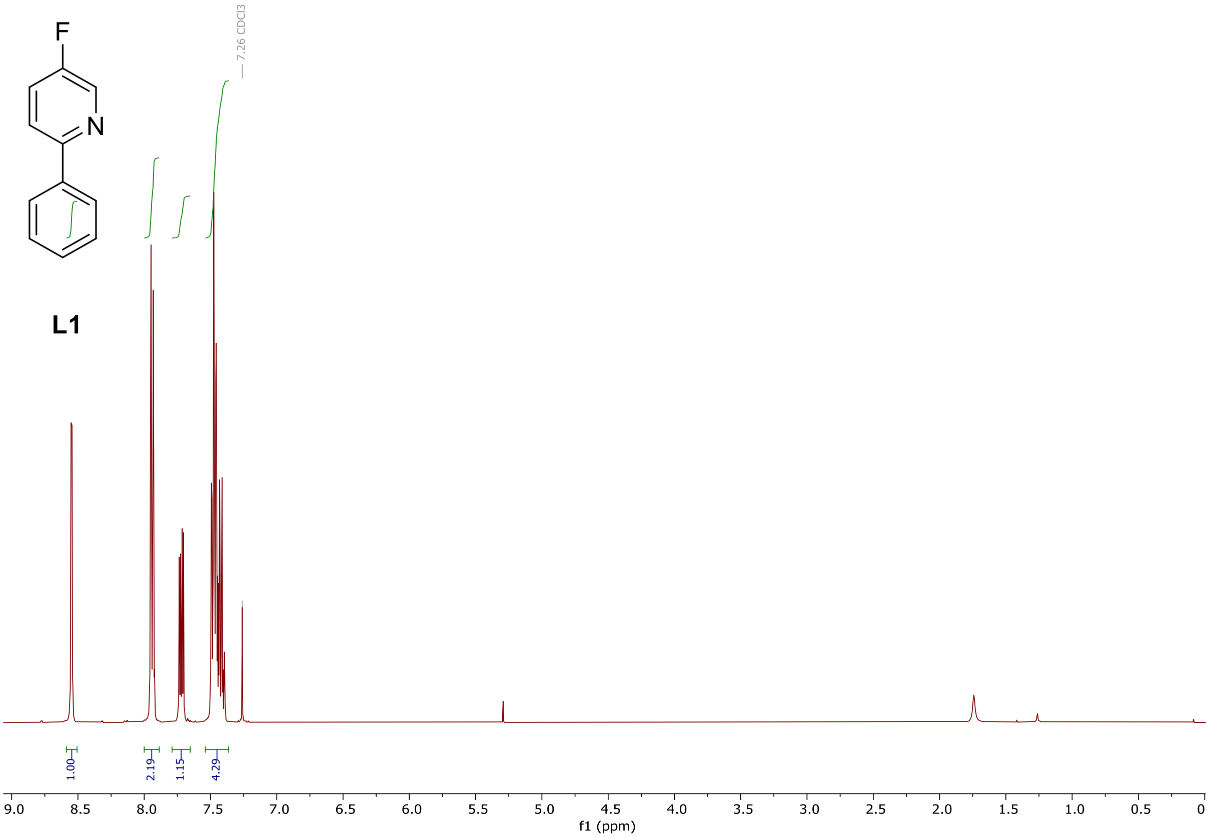

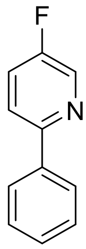

PP1

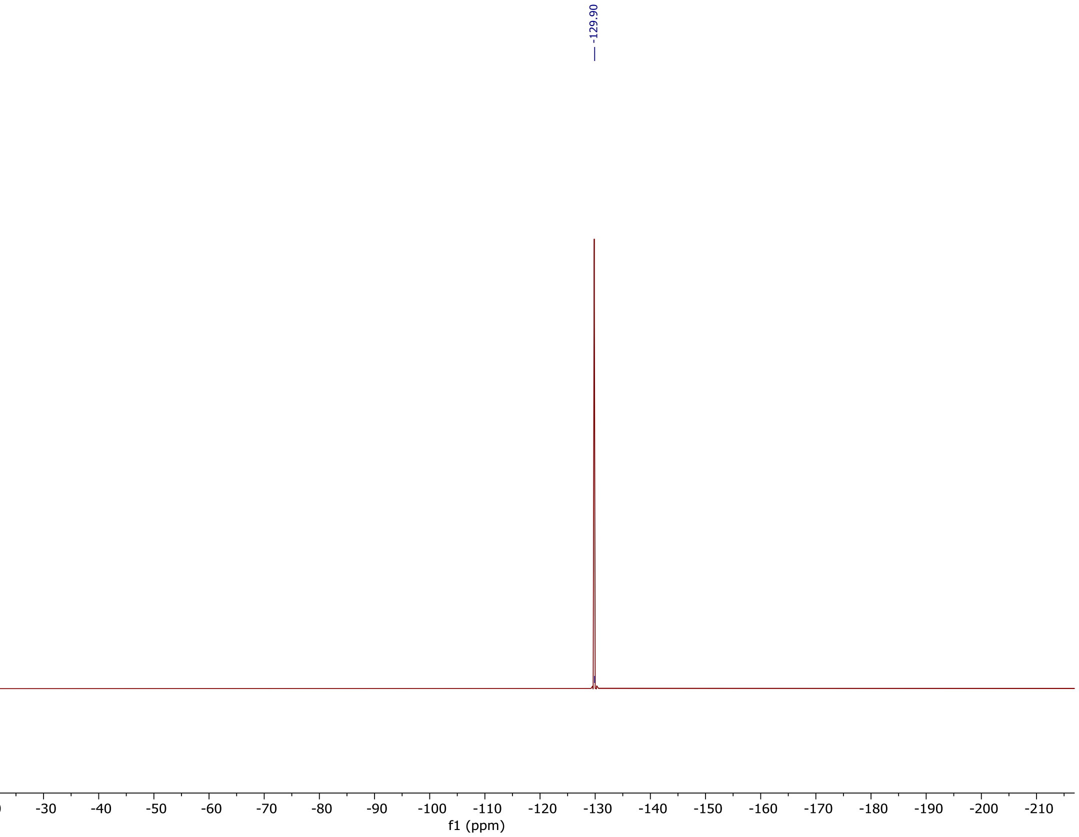

-129.90
0
-10
-20
-30
-40
-50
-60
-70
-80
-90
-100
-110
-120
-130
-140
-150
-160
-170
-180
-190
-200
-210
f1 (ppm)

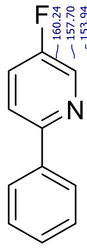

PP1

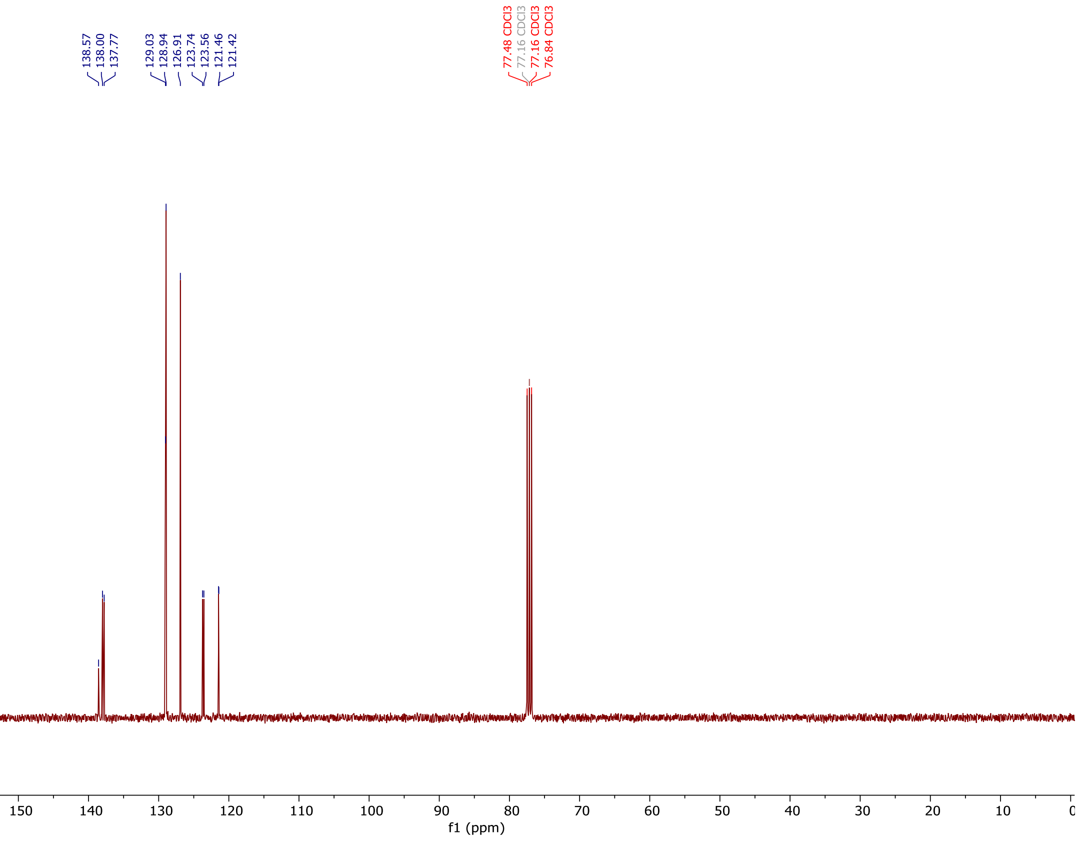

160.24
157.70
153.94
153.91
138.57
138.00
137.77
129.03
128.94
126.91
123.74
123.56
121.46
121.42
77.48 CDCl3
77.16 CDCl3
77.16 CDCl3
76.84 CDCl3
160
150
140
130
120
110
100
90
80
70
60
50
40
30
20
10
f1 (ppm)

2-(4-(*tert*-Butyl)phenyl)-5-fluoropyridine (PP2)

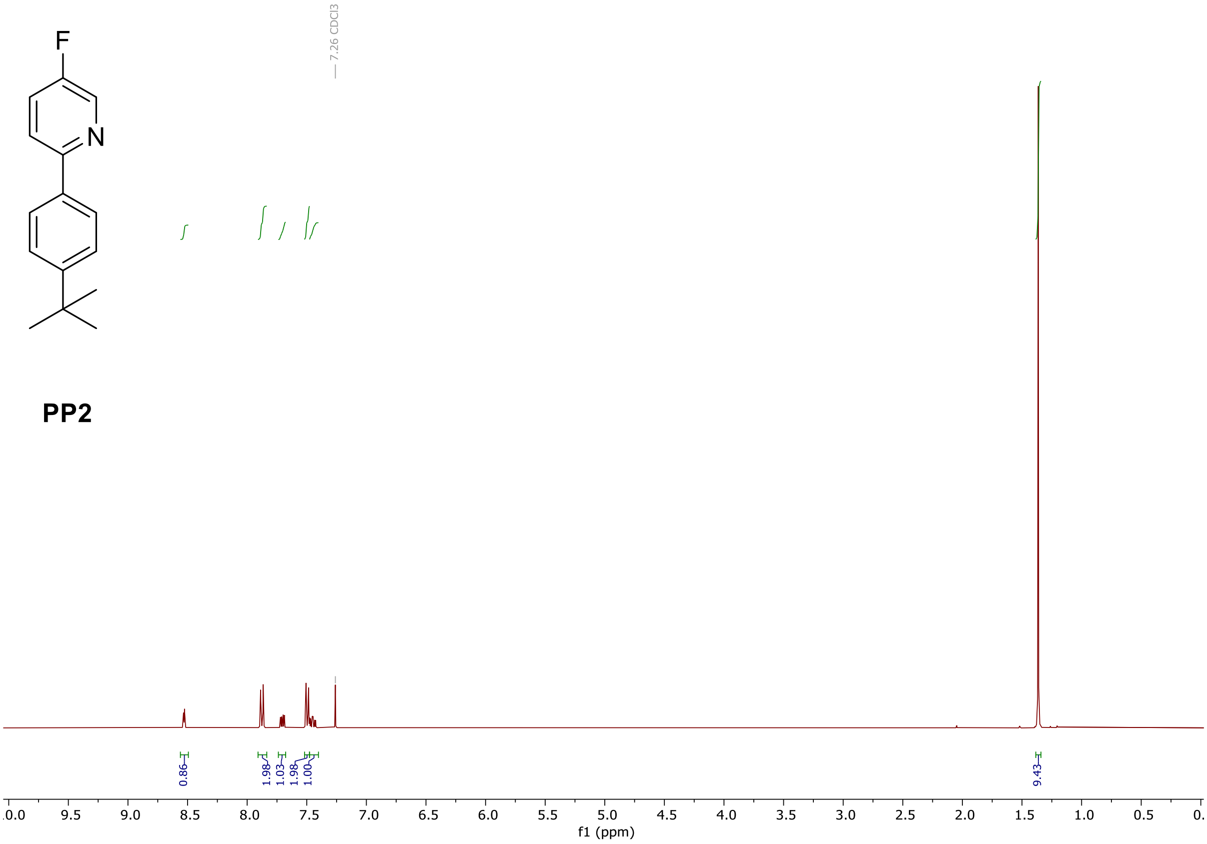

PP2

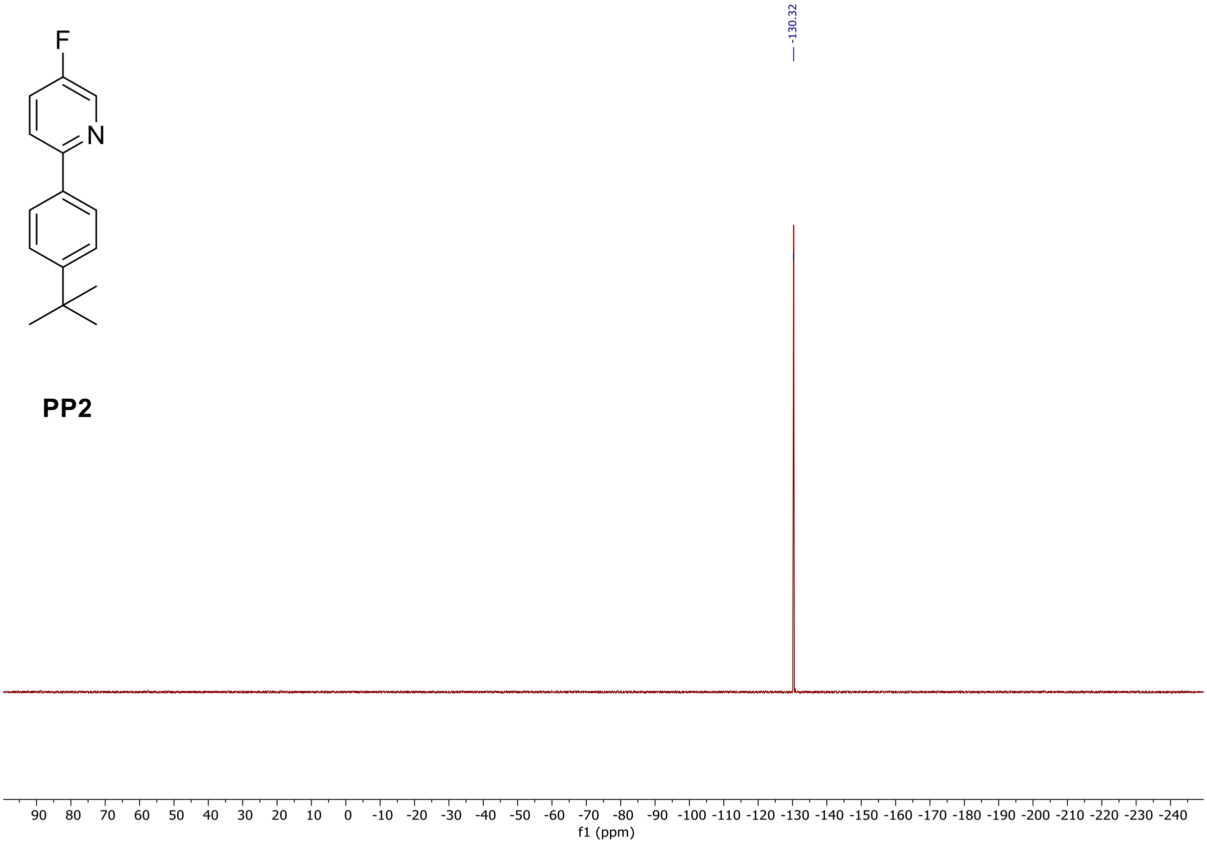

PP2

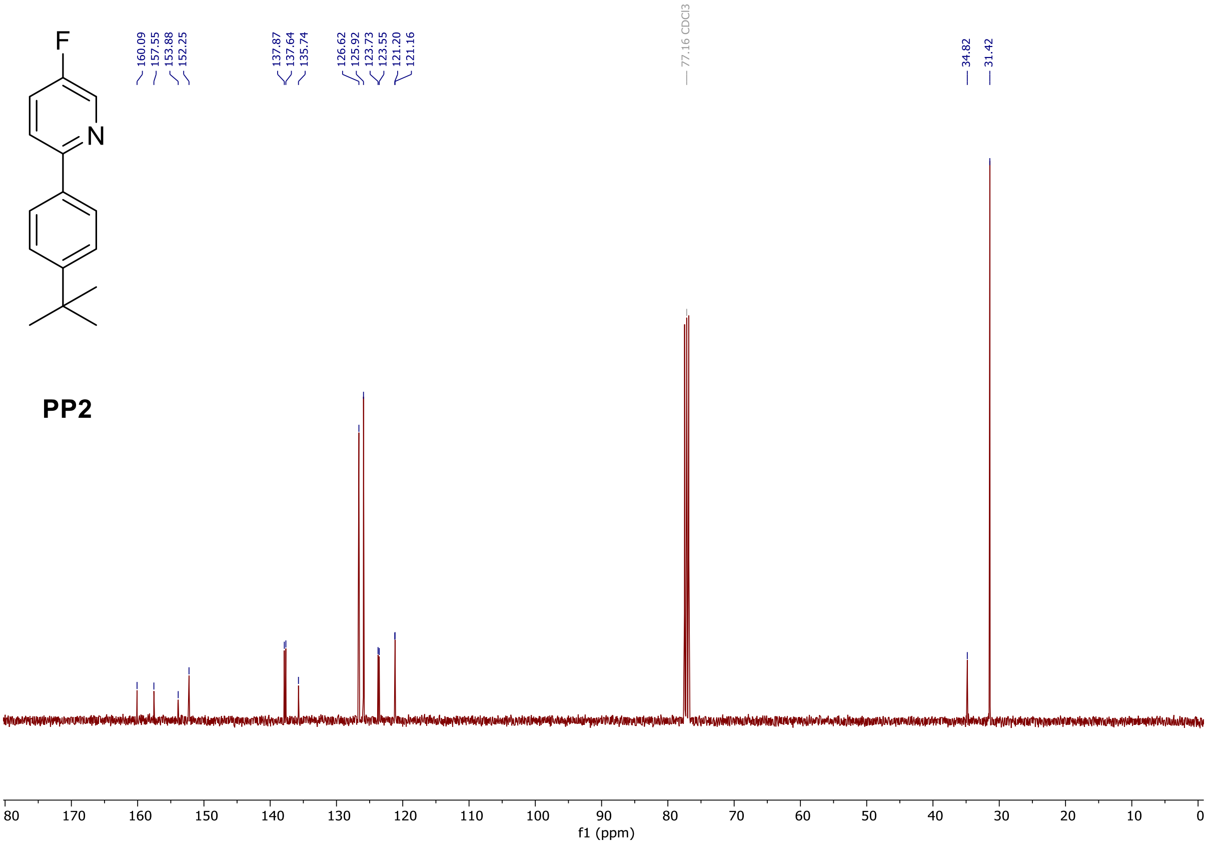

PP2

2-Phenyl-5-(trifluoromethyl)pyridine (PP3)

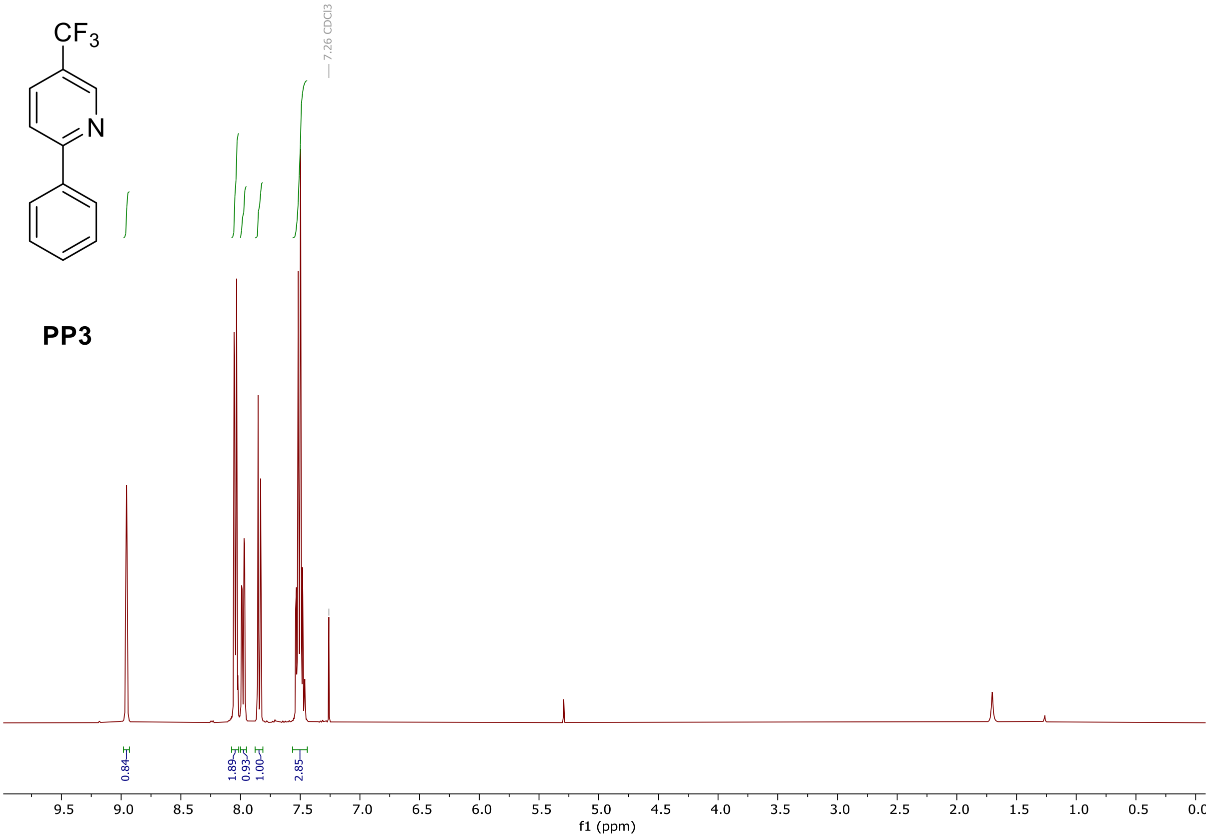

PP3

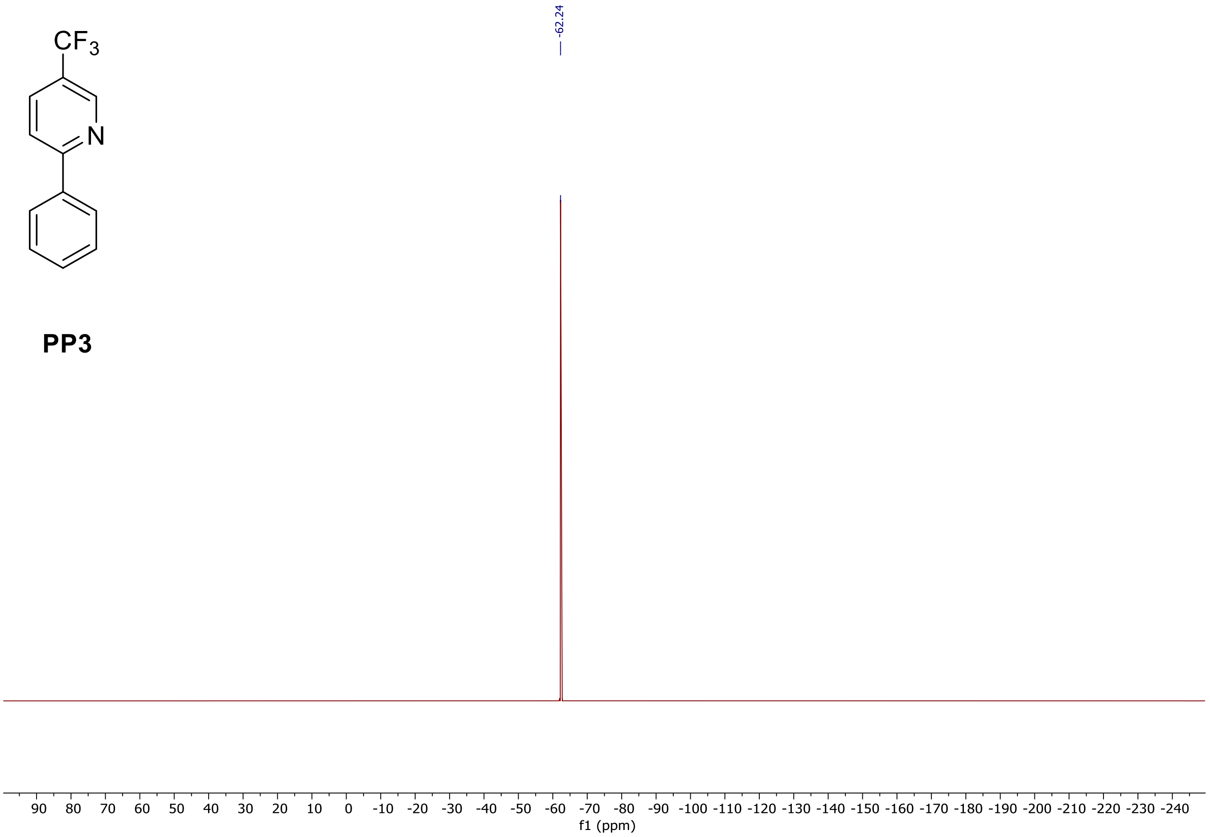
CF3
N
PP3
-62.24
90 80 70 60 50 40 30 20 10 0 -10 -20 -30 -40 -50 -60 -70 -80 -90 -100 -110 -120 -130 -140 -150 -160 -170 -180 -190 -200 -210 -220 -230 -240
f1 (ppm)

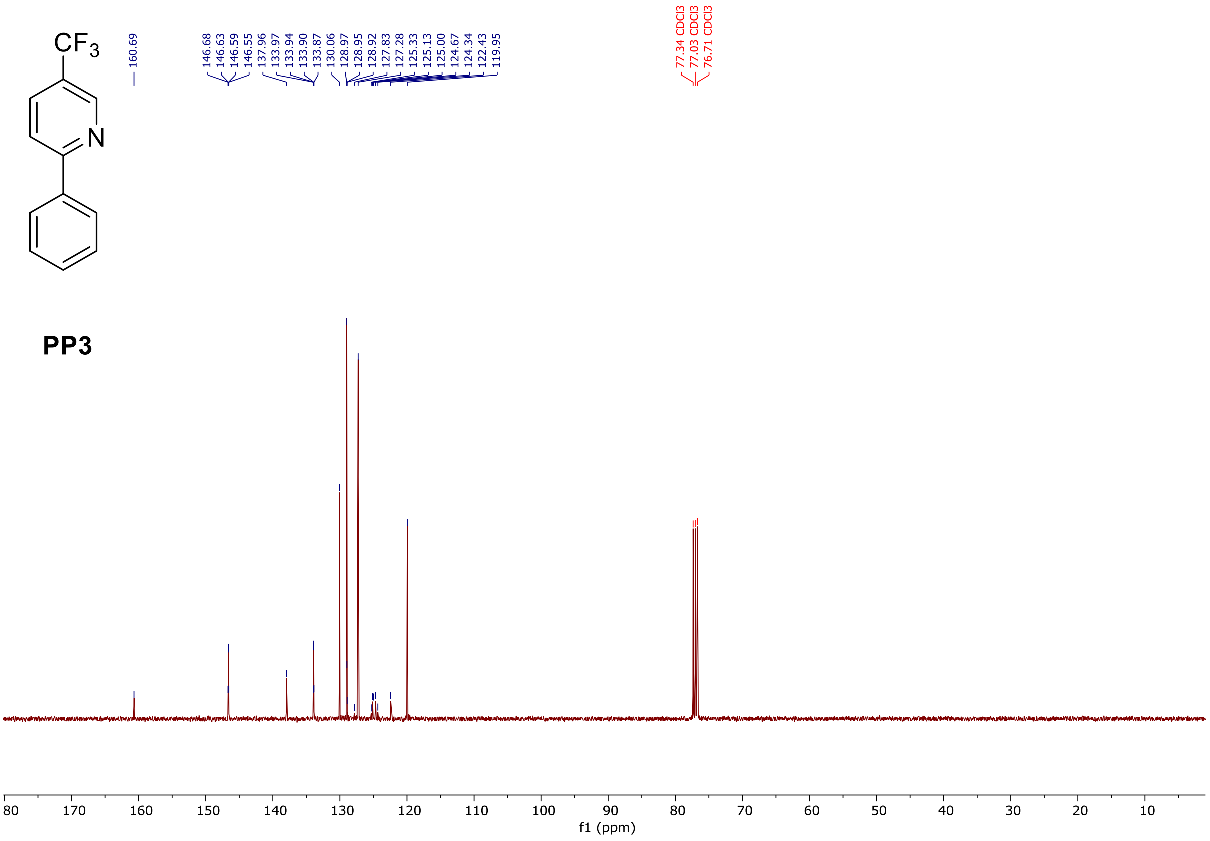
CF3
N
PP3
160.69
146.68
146.63
146.59
146.55
137.96
133.97
133.94
133.90
133.87
130.06
128.97
128.95
128.92
127.83
127.28
125.33
125.13
125.00
124.67
124.34
122.43
119.95
77.34 CDCl3
77.03 CDCl3
76.71 CDCl3
80 170 160 150 140 130 120 110 100 90 80 70 60 50 40 30 20 10
f1 (ppm)

2-(4-(*tert*-Butyl)phenyl)-5-(trifluoromethyl)pyridine (PP4)

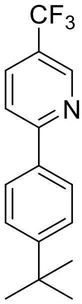

PP4

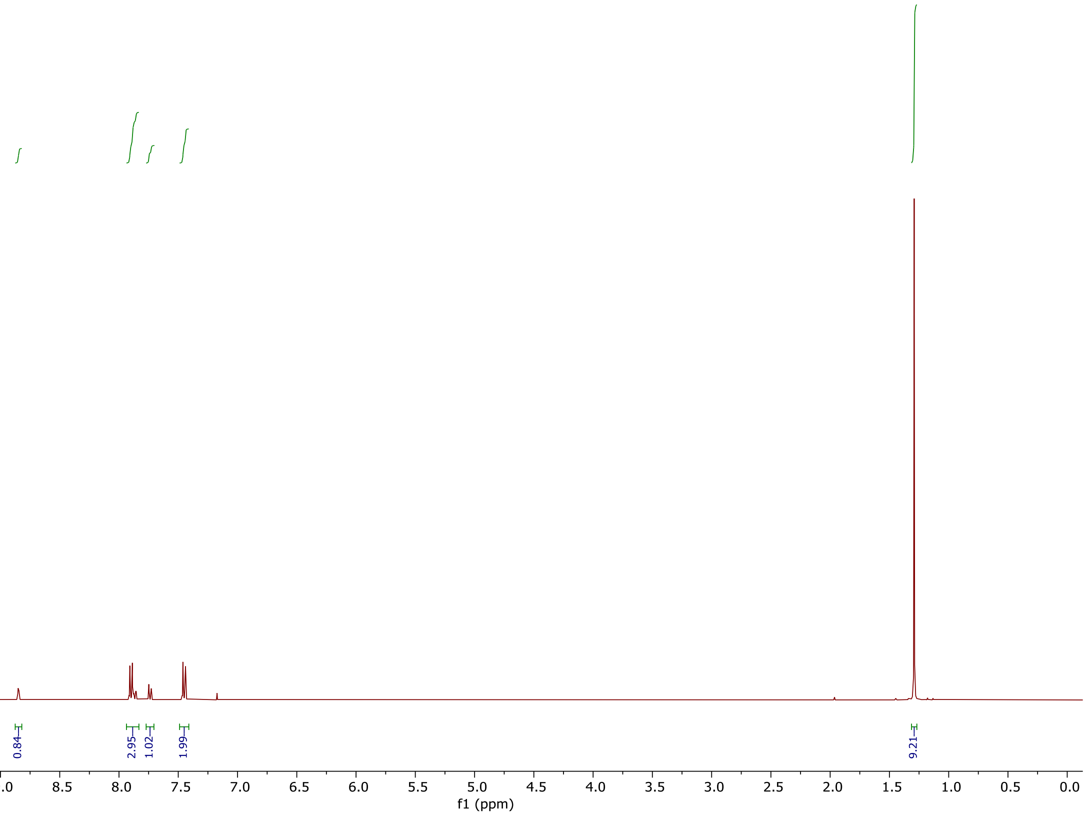

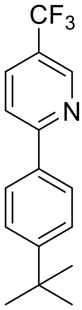

PP4

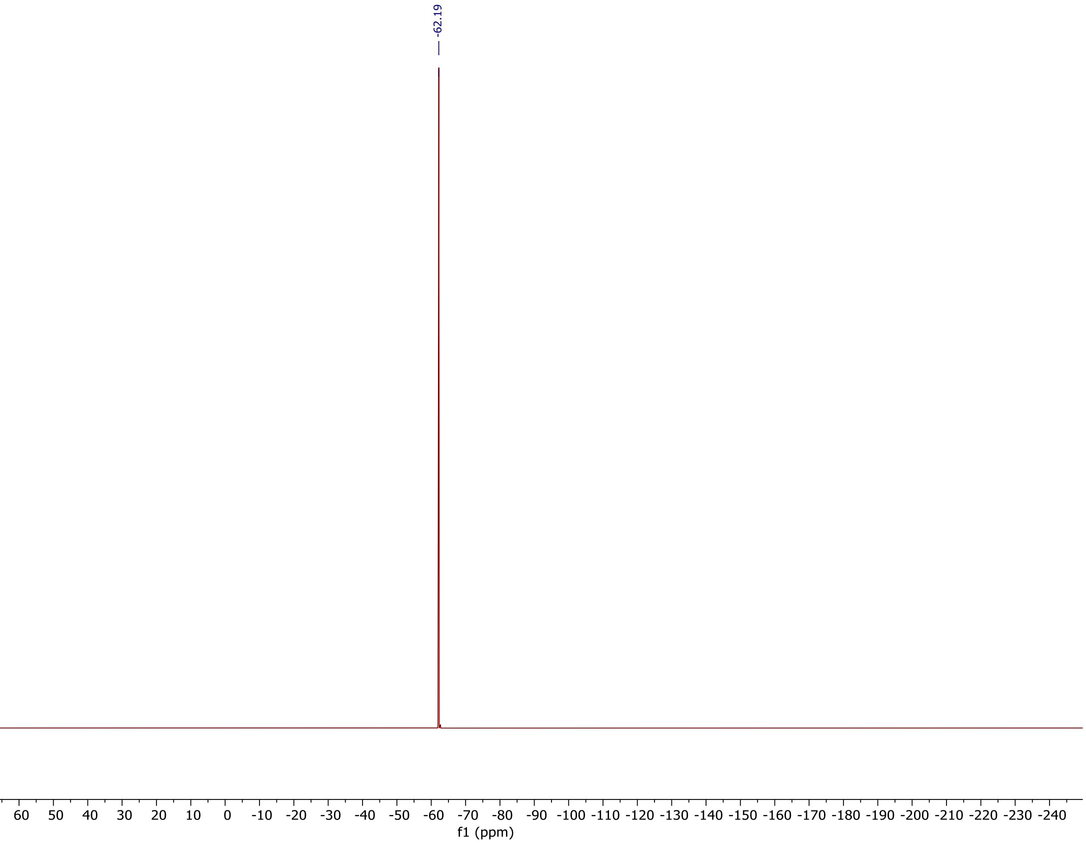

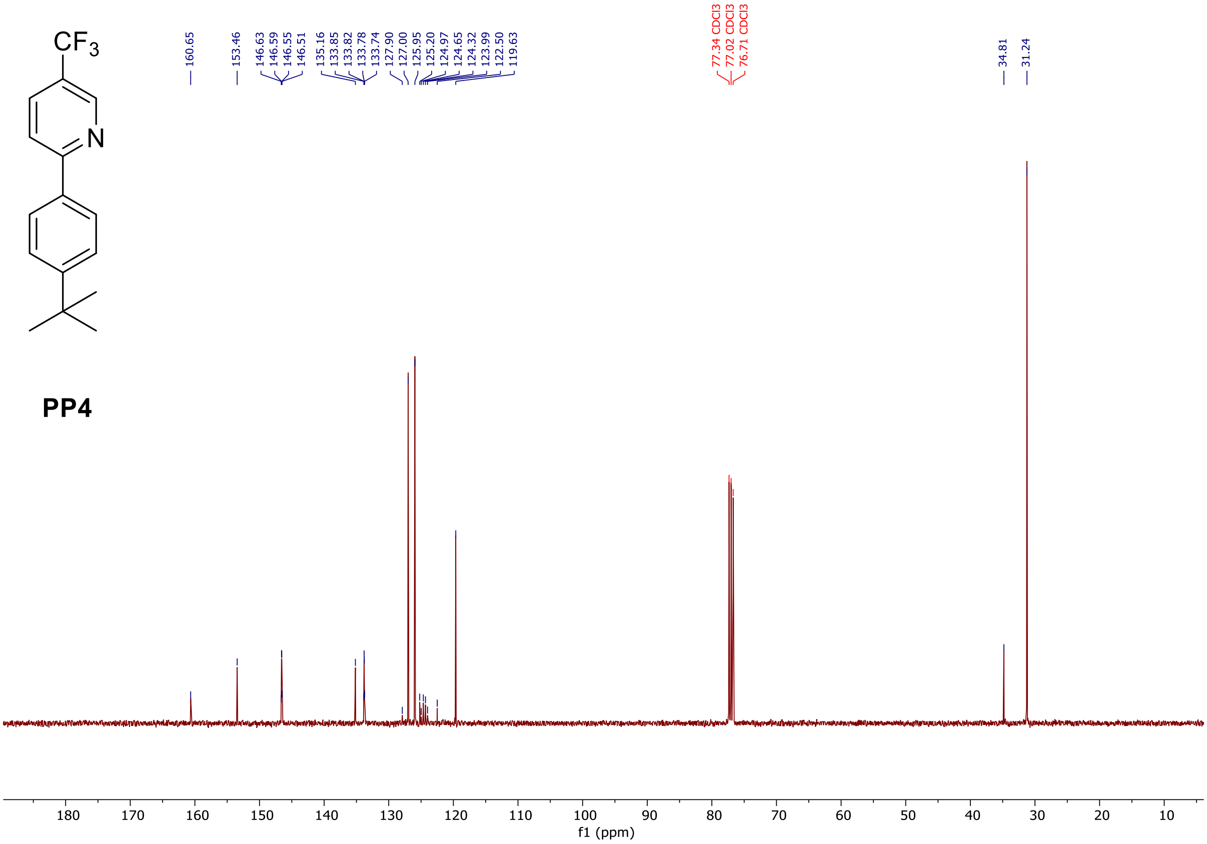
CF3
N
PP4
160.65
153.46
146.63
146.59
146.55
146.51
135.16
133.85
133.82
133.78
133.74
127.90
127.00
125.95
125.20
124.97
124.65
124.32
123.99
122.50
119.63
77.34 CDCl3
77.02 CDCl3
76.71 CDCl3
34.81
31.24
180 170 160 150 140 130 120 110 100 90 80 70 60 50 40 30 20 10
f1 (ppm)

Ir [(5-F)ppy]$_3$ (C1)

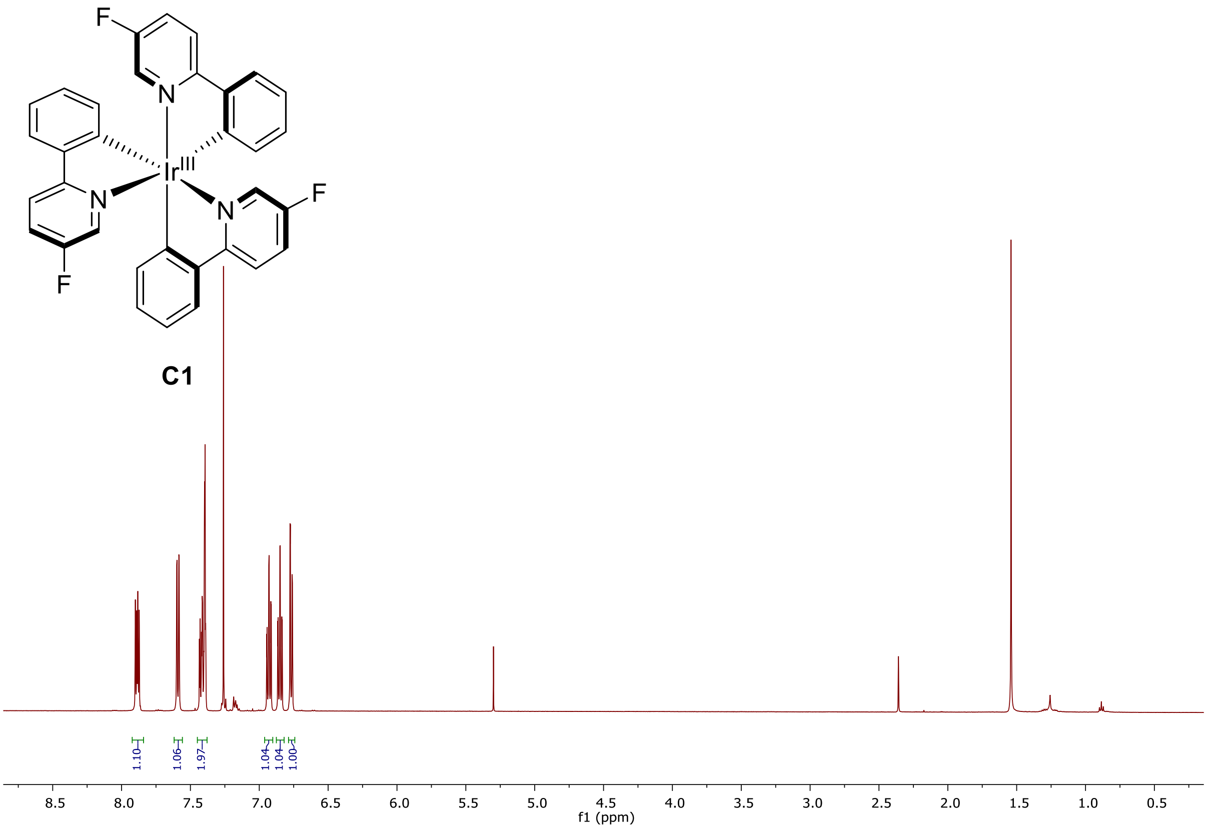
F
N
Ir
III
N
F
N
F
C1
1.10
1.06
1.97
1.04
1.04
1.00
8.5 8.0 7.5 7.0 6.5 6.0 5.5 5.0 4.5 4.0 3.5 3.0 2.5 2.0 1.5 1.0 0.5
f1 (ppm)

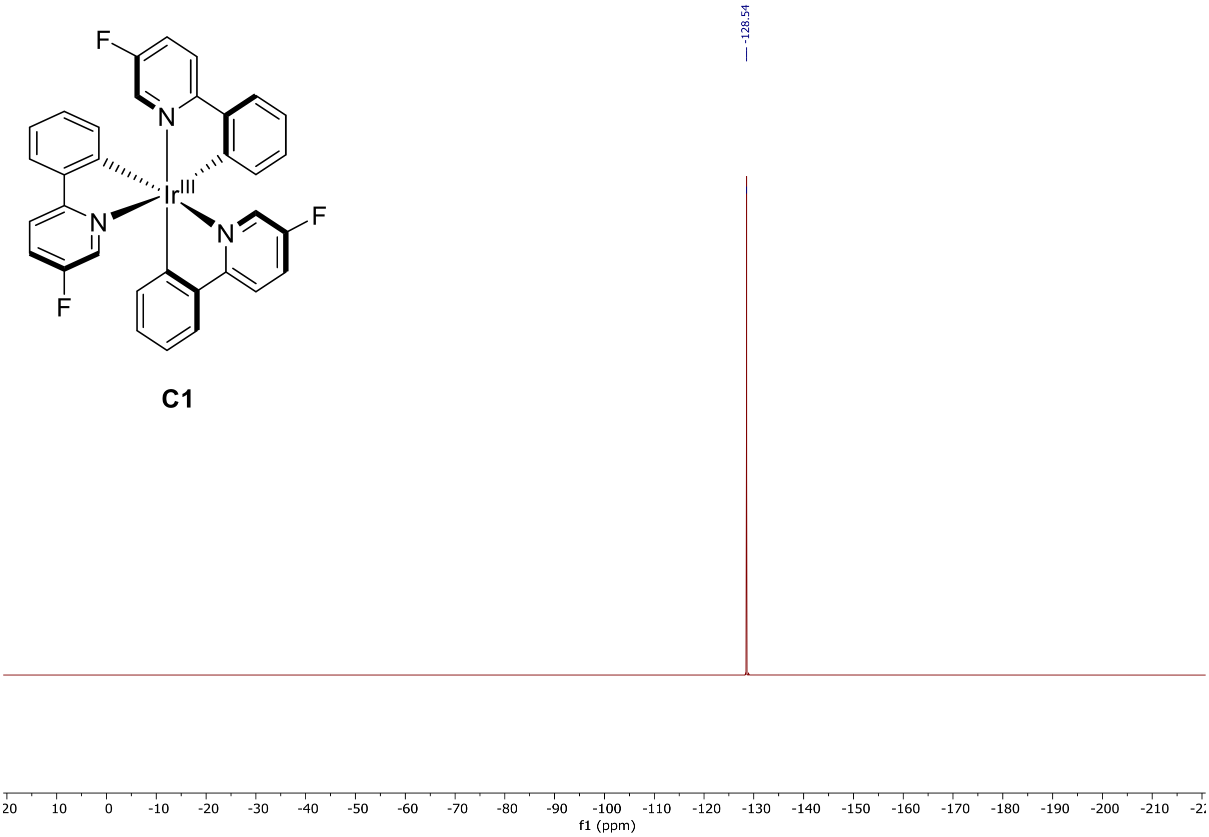

-128.54
C1
f1 (ppm)

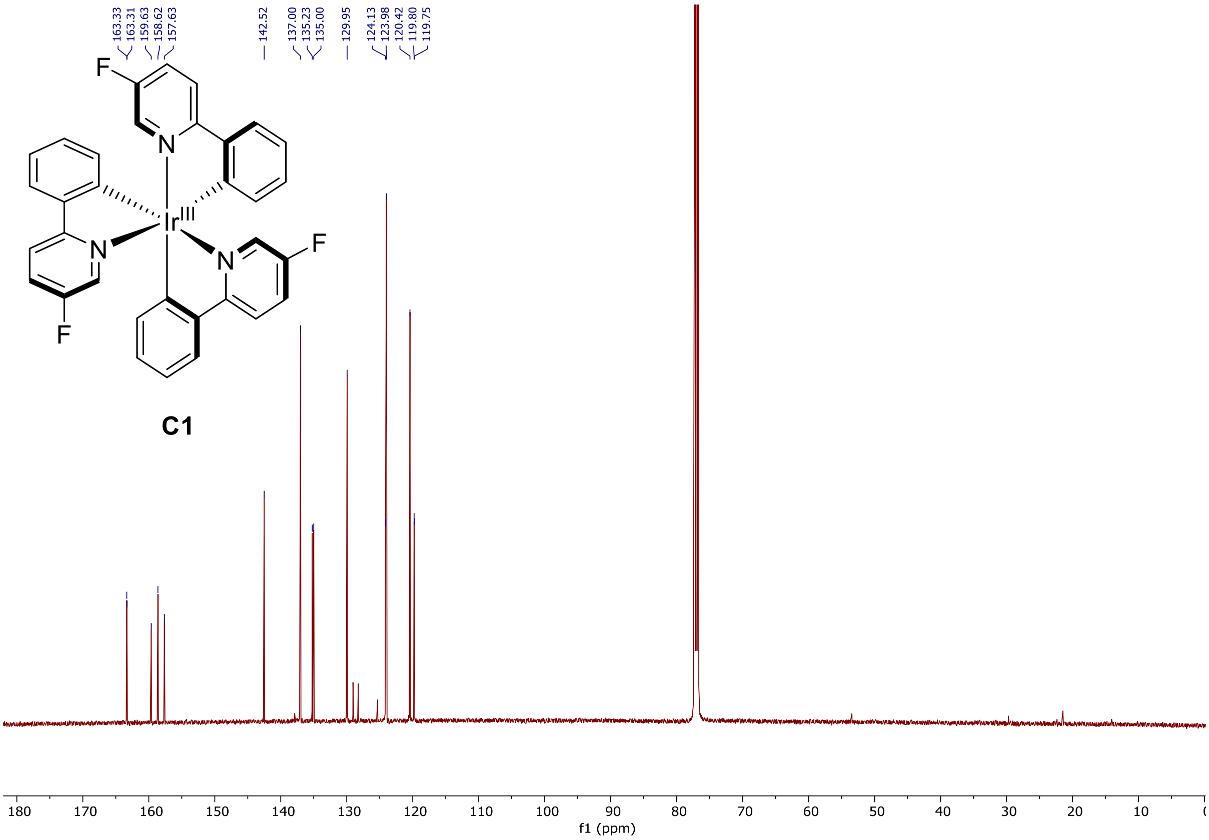

163.33
163.31
159.63
158.62
157.63
142.52
137.00
135.23
135.00
129.95
124.13
123.98
120.42
119.80
119.75
C1
f1 (ppm)

Ir((5-F, 4'-^{t}Bu)ppy)$_3$ (C2)

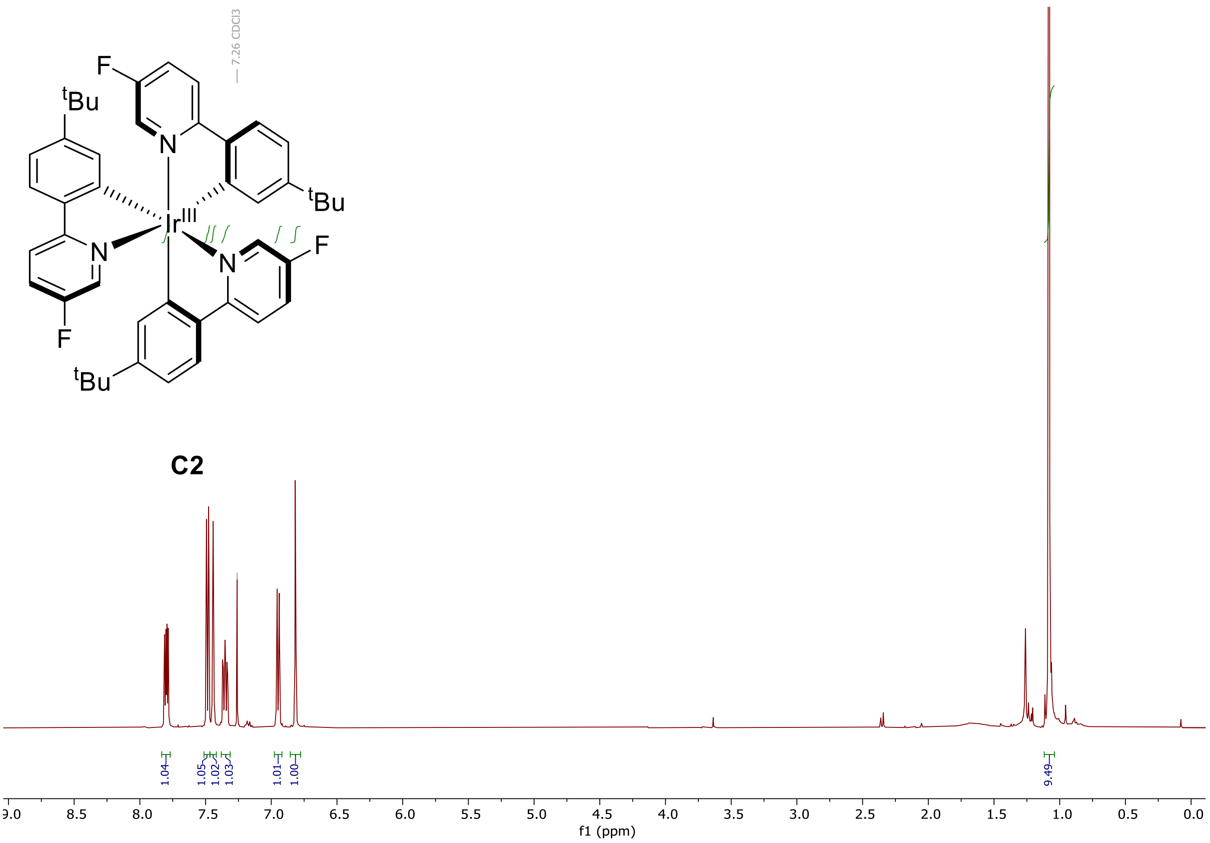

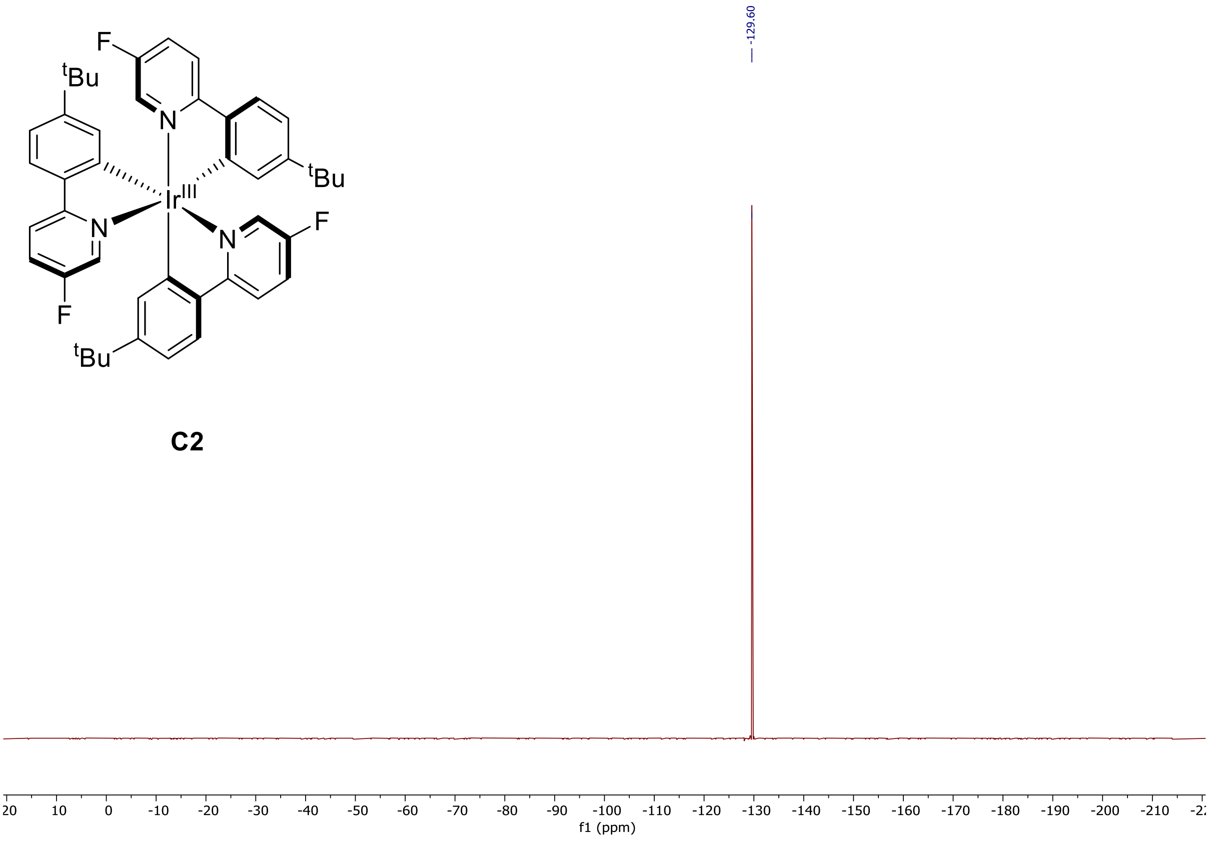

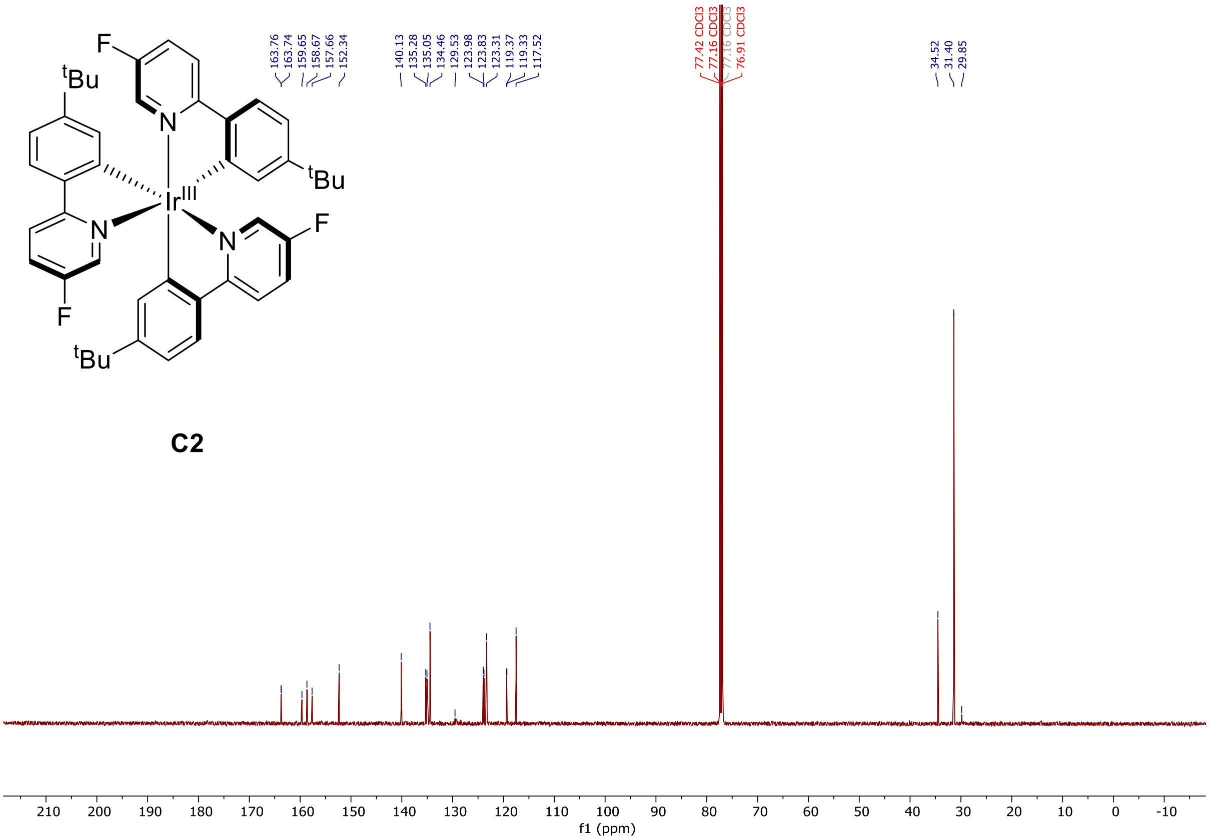

Ir((5-CF$_3$)ppy)$_3$ (C3)

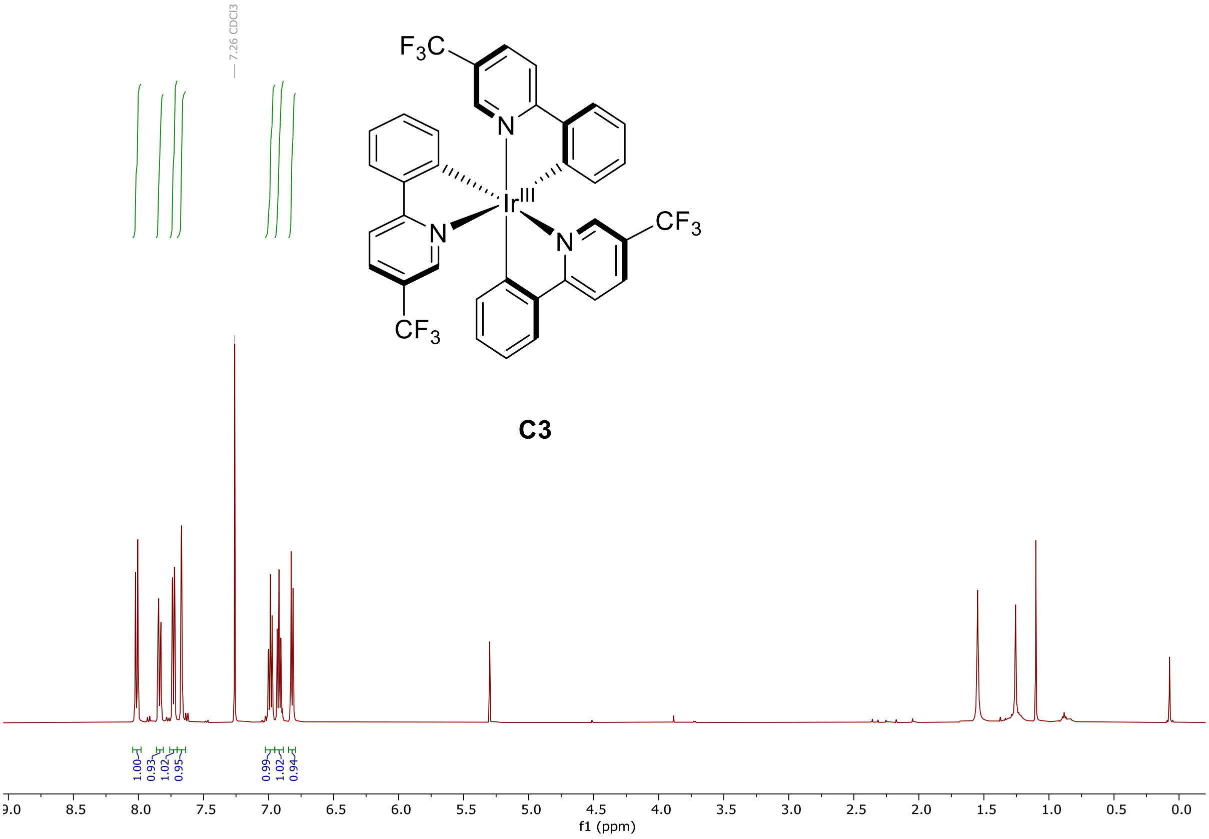

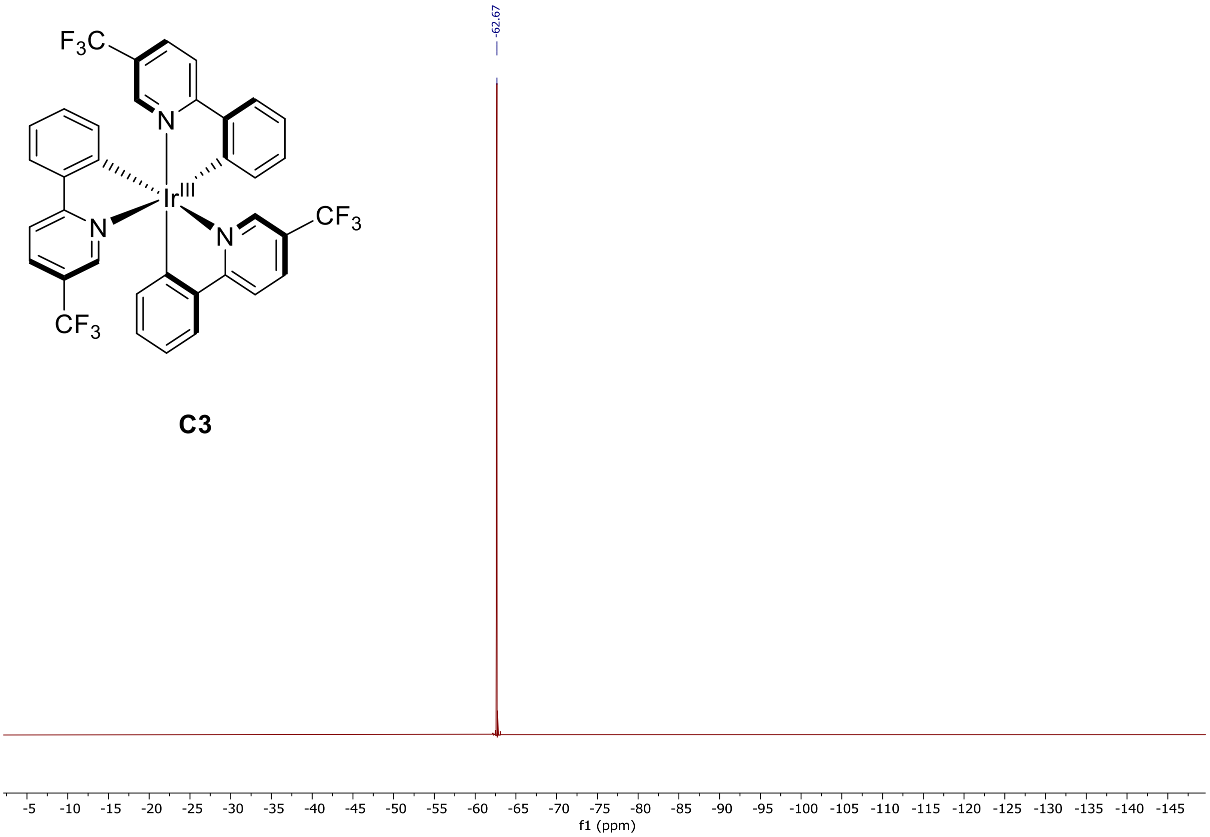
F3C
N
Ir III
N
N
CF3
CF3
C3
—62.67
-5 -10 -15 -20 -25 -30 -35 -40 -45 -50 -55 -60 -65 -70 -75 -80 -85 -90 -95 -100 -105 -110 -115 -120 -125 -130 -135 -140 -145
f1 (ppm)

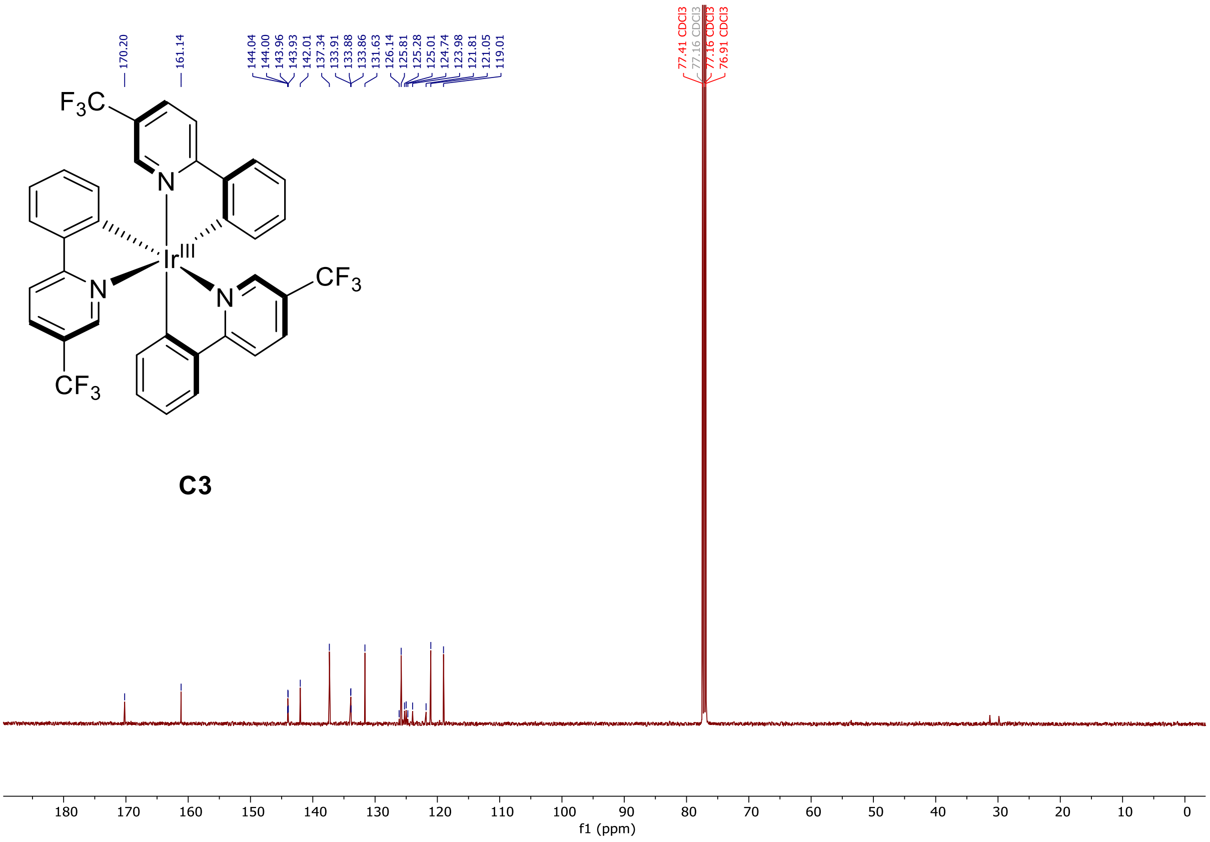
170.20
161.14
144.04
144.00
143.96
143.93
142.01
137.34
133.91
133.88
133.86
131.63
126.14
125.81
125.28
125.01
124.74
123.98
121.81
121.05
119.01
77.41 CDCl3
77.16 CDCl3
77.16 CDCl3
76.91 CDCl3
F3C
N
Ir III
N
N
CF3
CF3
C3
180 170 160 150 140 130 120 110 100 90 80 70 60 50 40 30 20 10 0
f1 (ppm)

Ir((5-CF$_3$, 4'-^{t}Bu)ppy$_3$ (4)

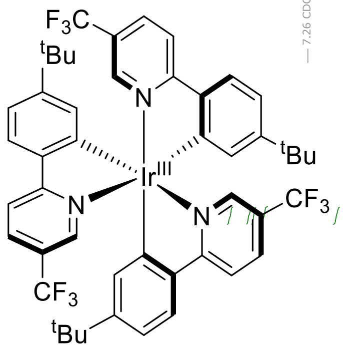

4

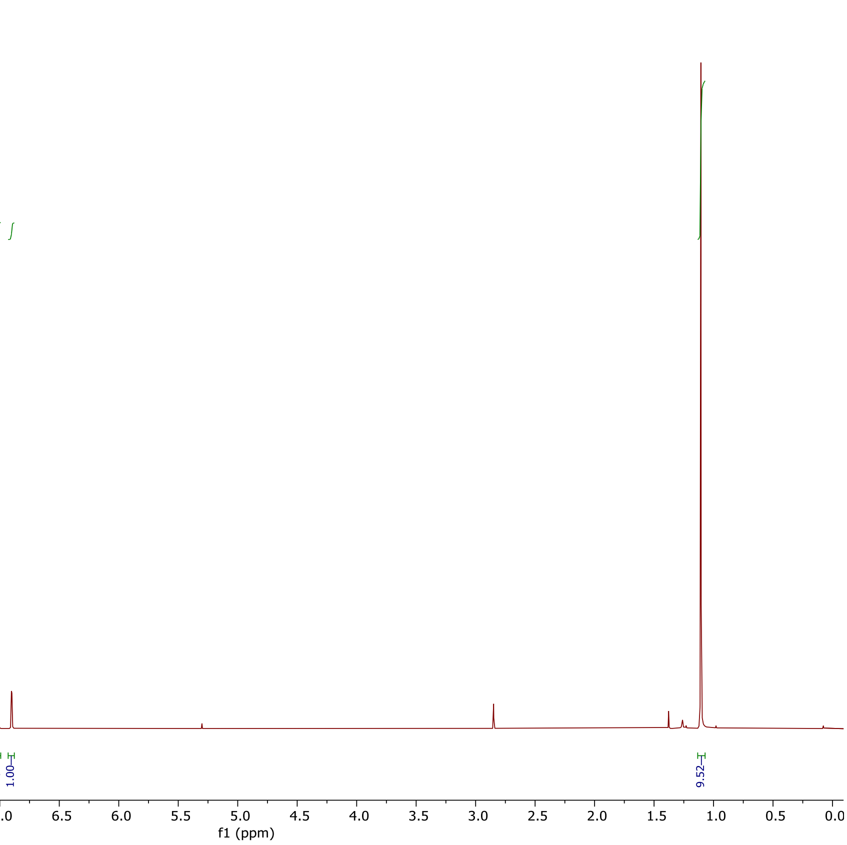

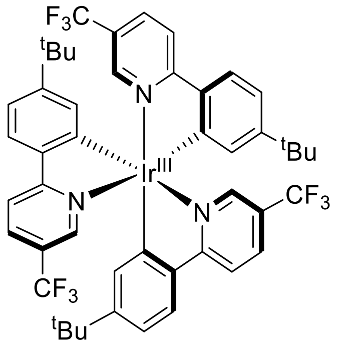

4

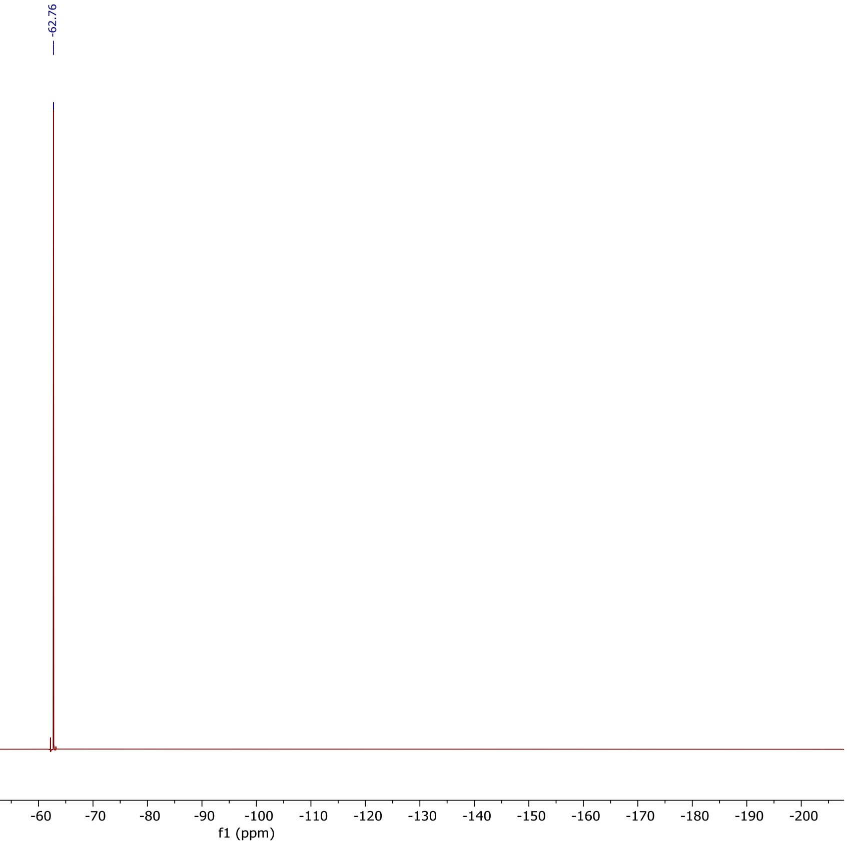

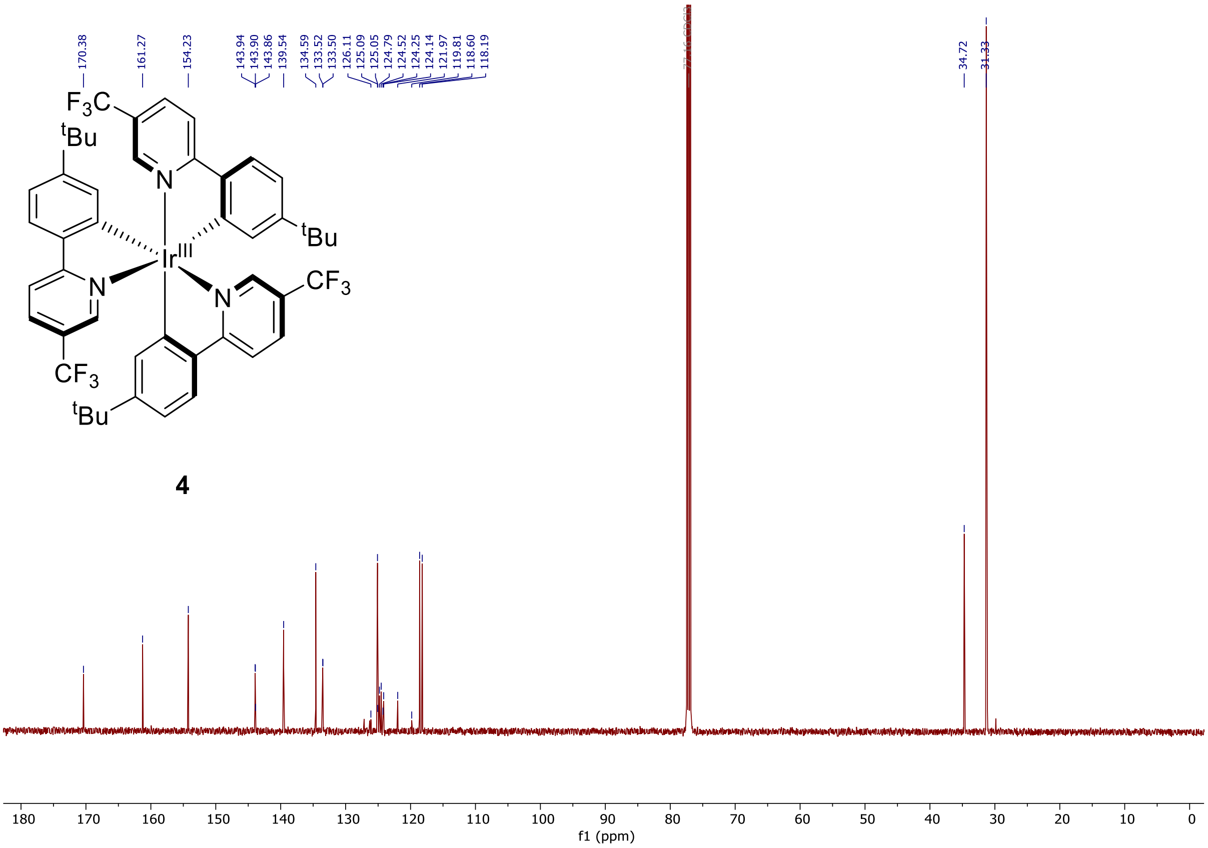

9.2. Precursors

3,4-Dihydro-2H-pyran-6-carboxylic acid (P1)

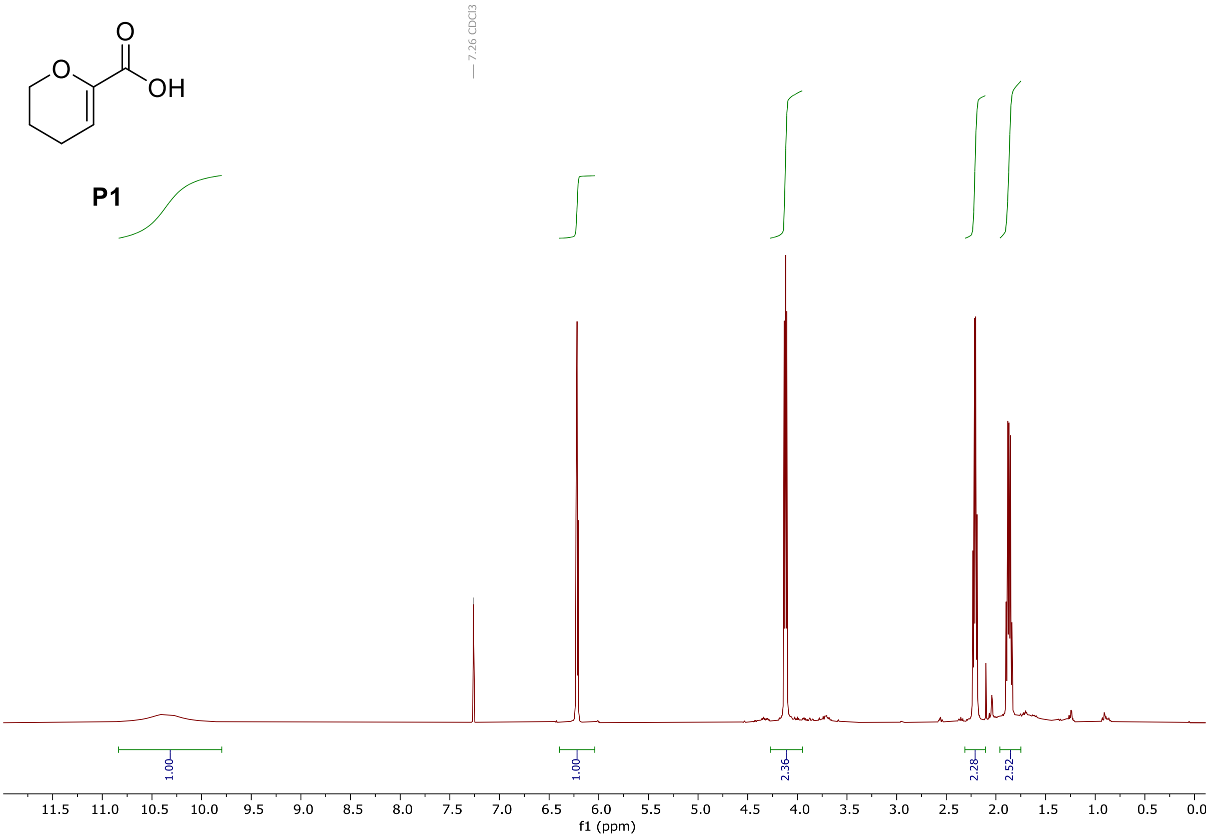

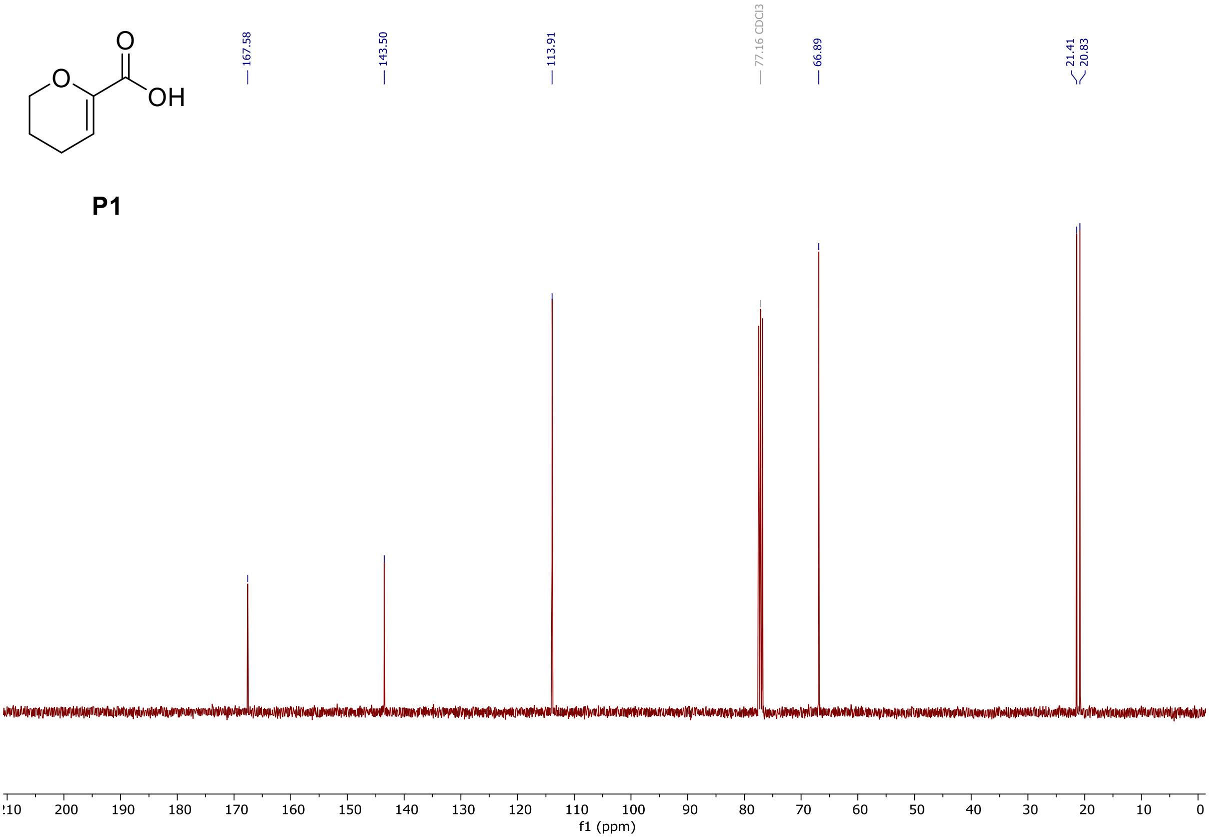

P1

4,5-Dihydrofuran-2-carboxylic acid (P2)

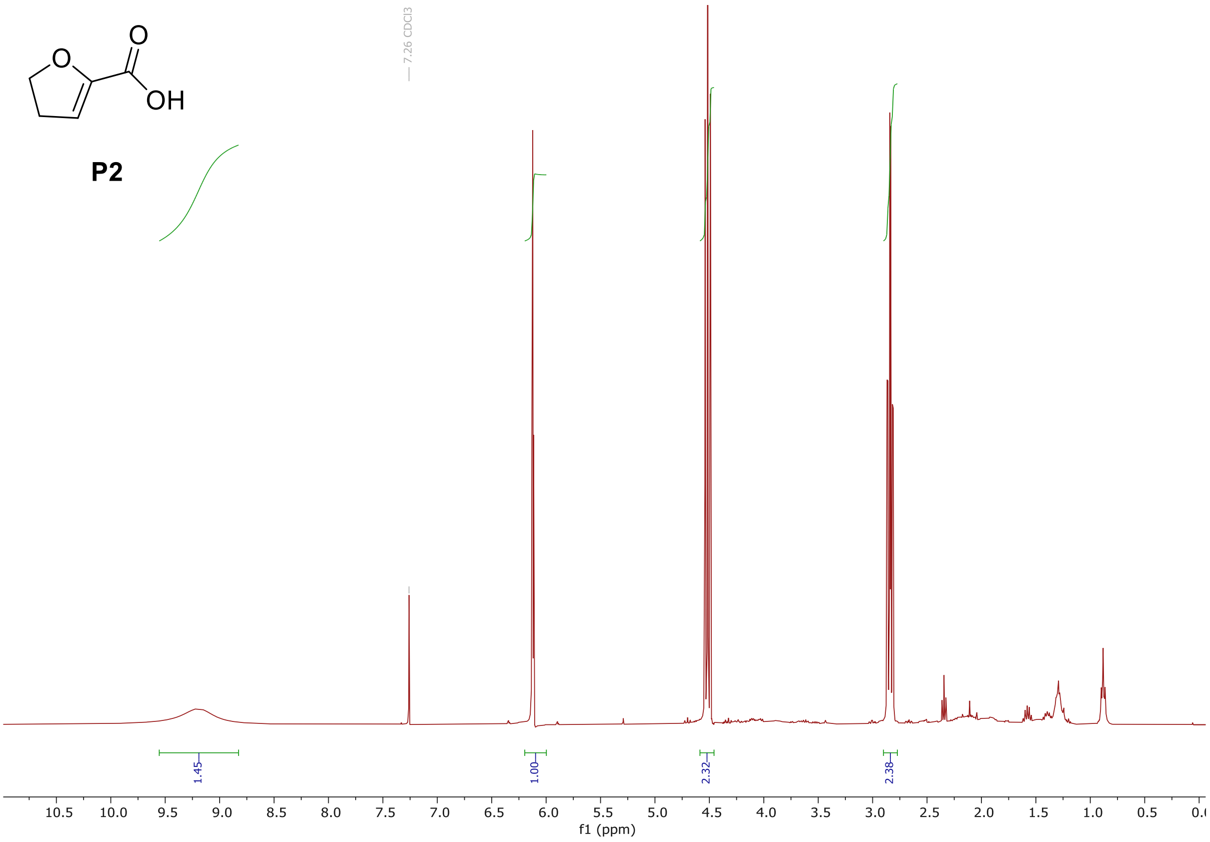

P2

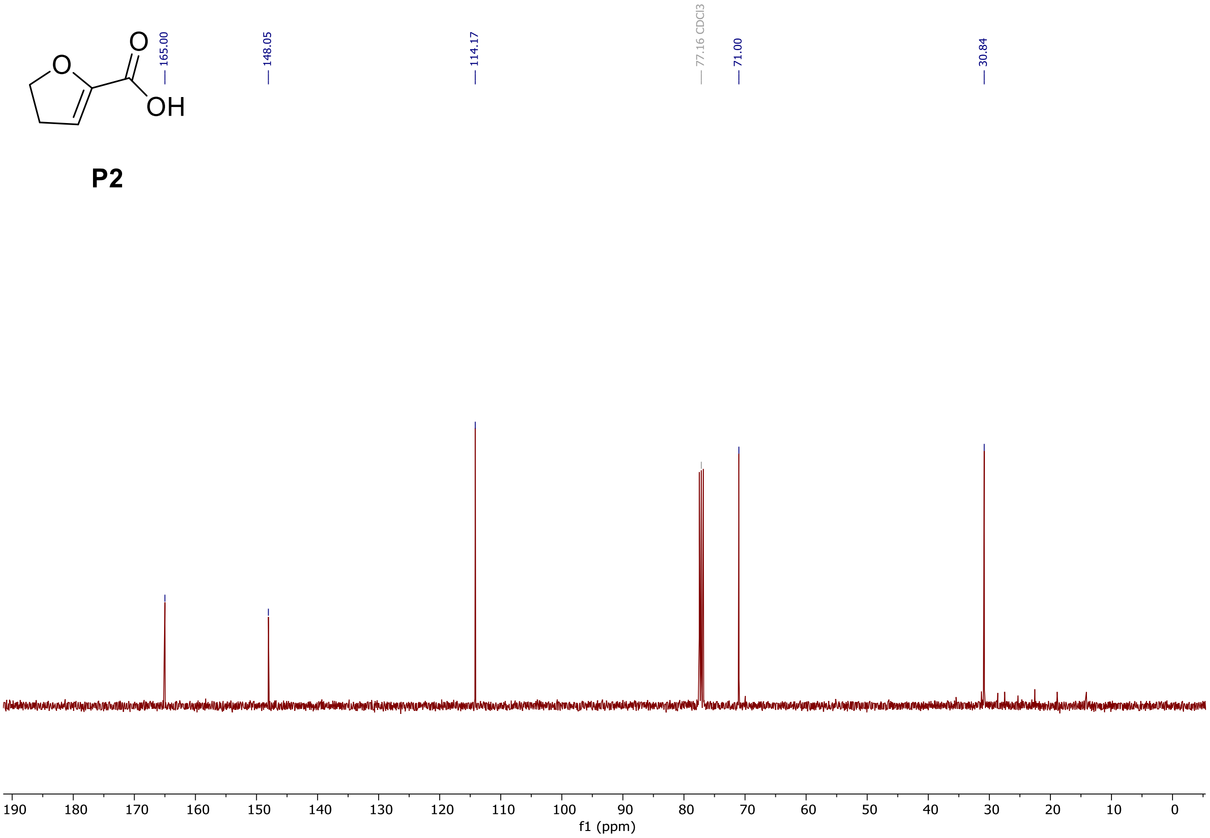

N-Isobutylaniline (P3)

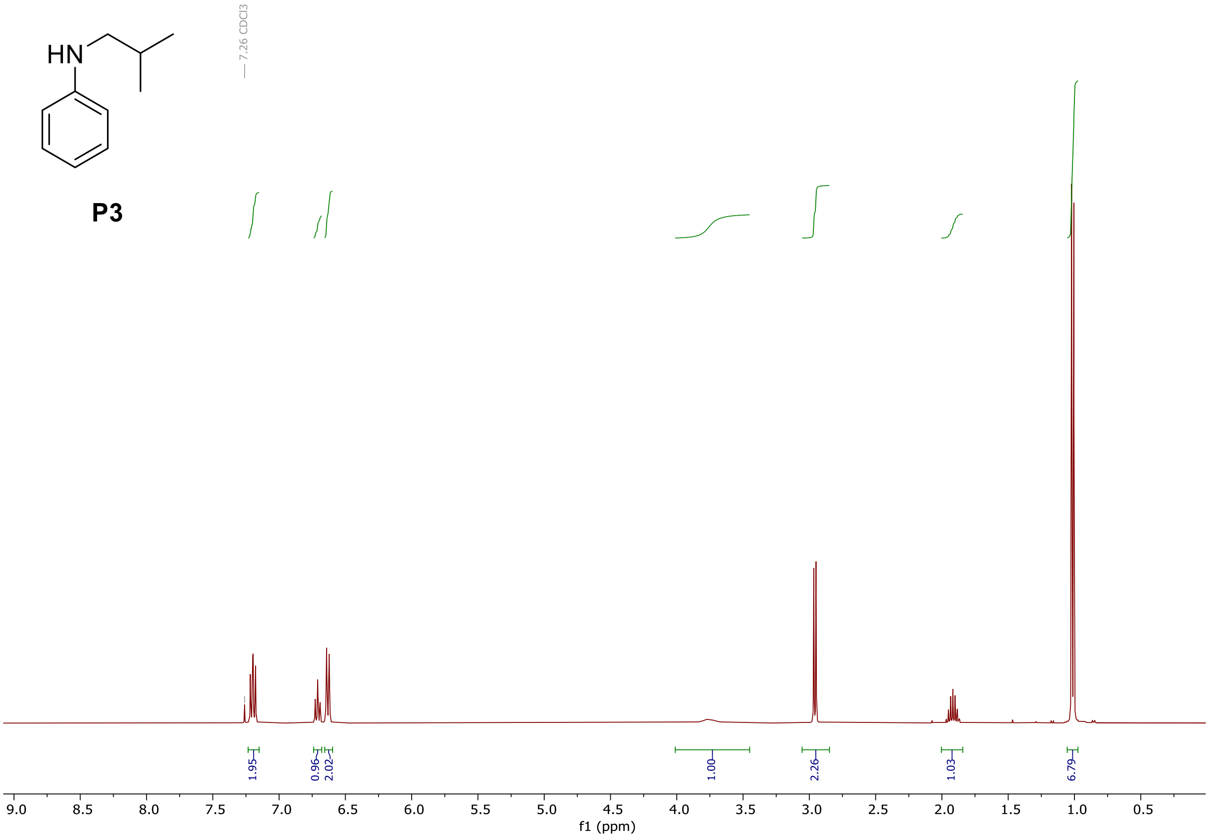

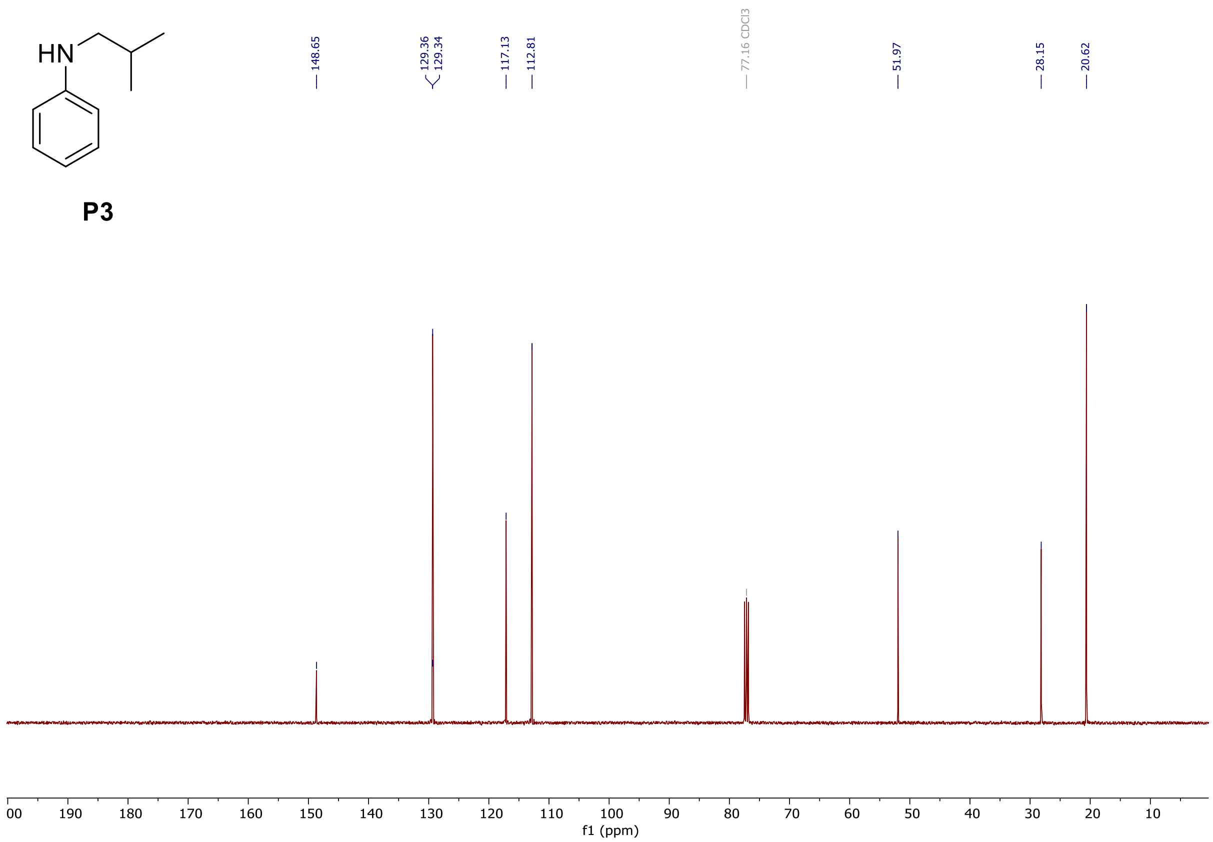

P3

N-Benzyl-4-chloroaniline (P4)

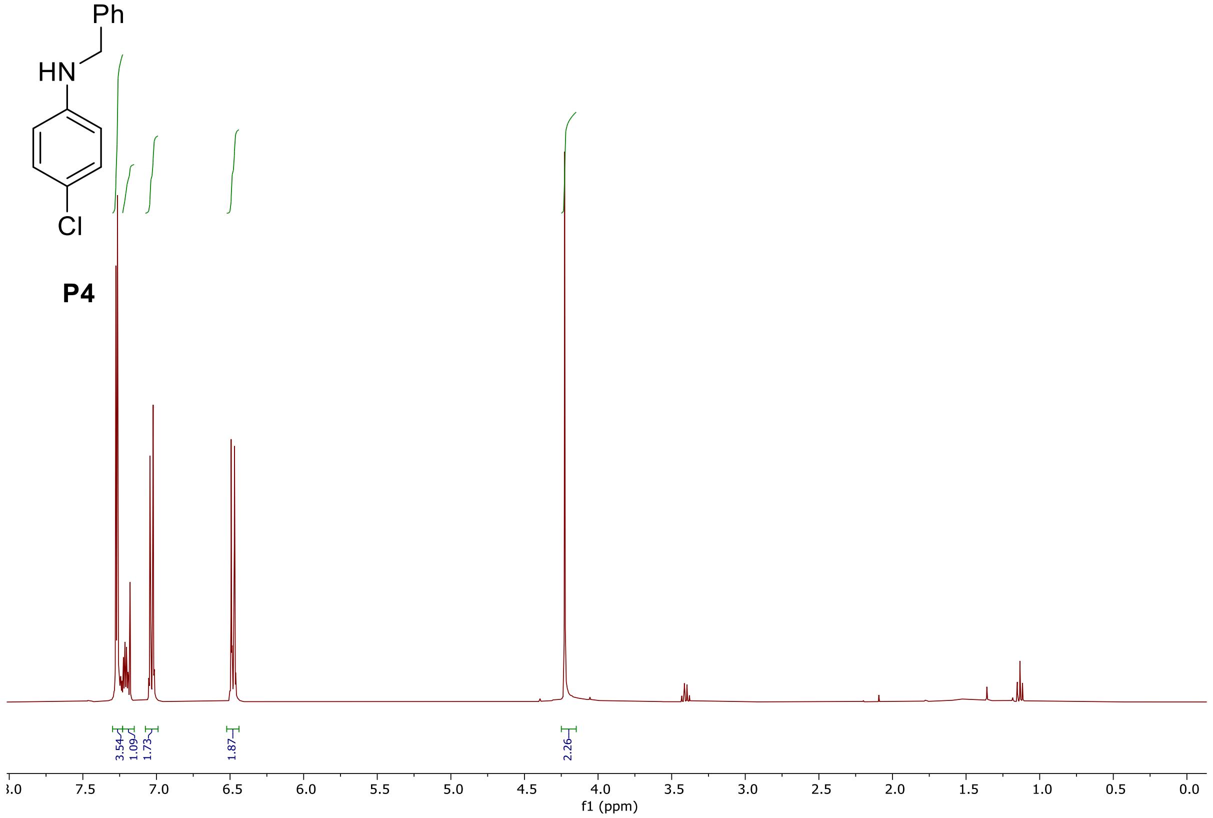

P4

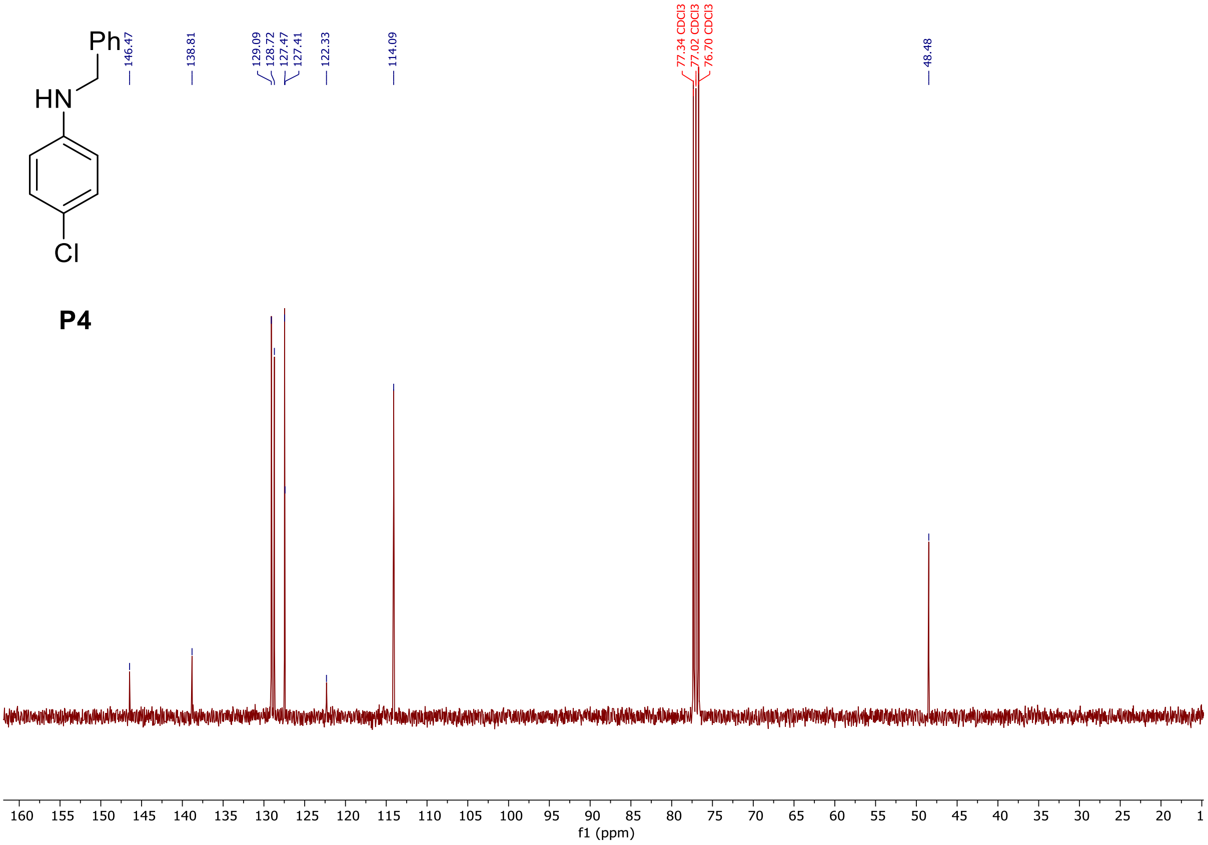
Ph
HN
Cl
146.47
138.81
129.09
128.72
127.47
127.41
122.33
114.09
77.34 CDCl3
77.02 CDCl3
76.70 CDCl3
48.48
f1 (ppm)

P4

4-(*tert*-Butyl)-*N*-methylaniline (P5)

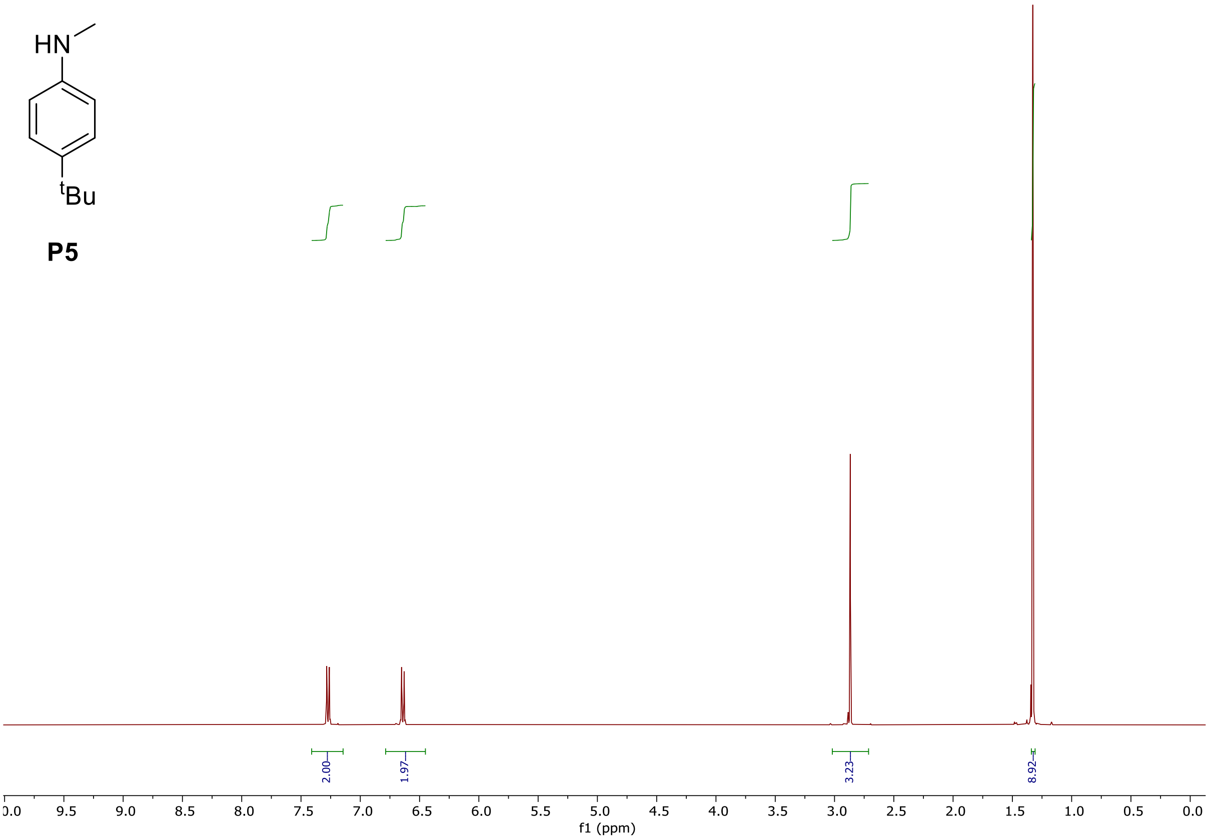

P5

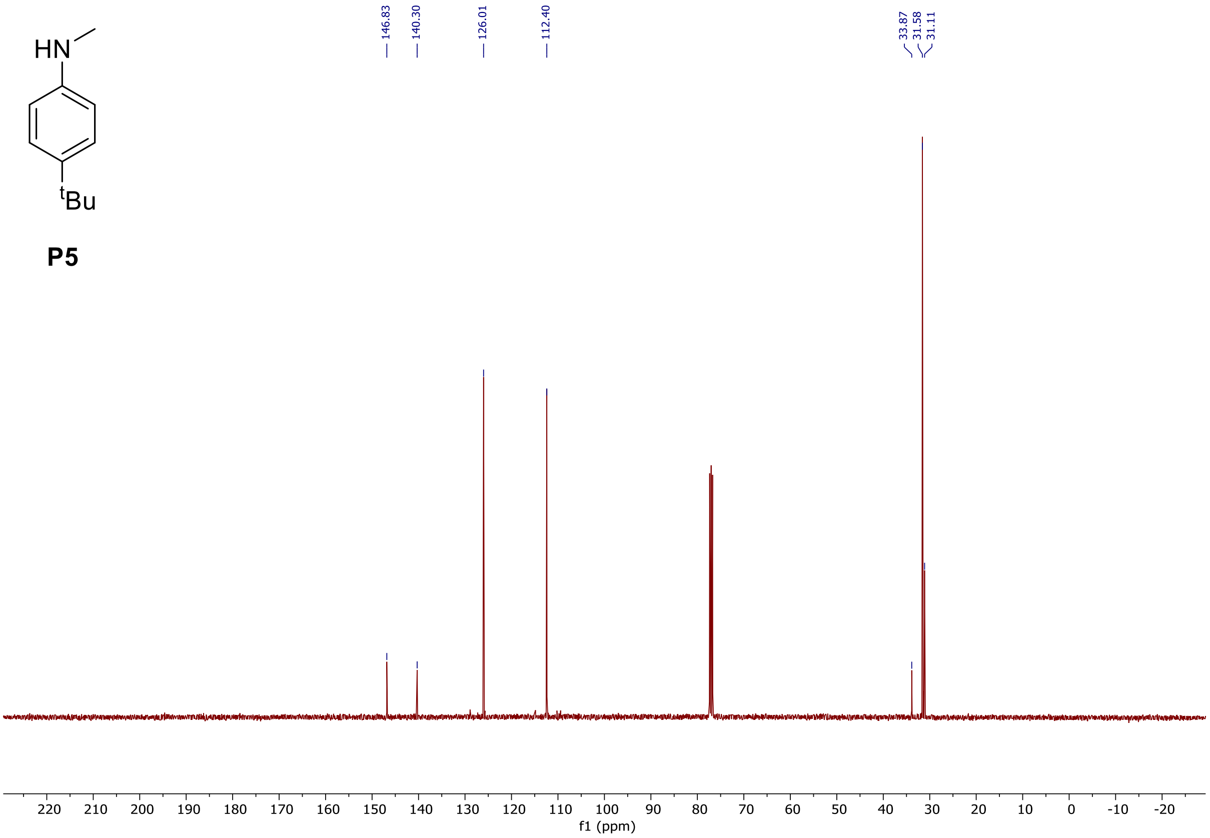

P5

N-Methyl-4-(methylthio)aniline (P6)

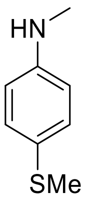

P6

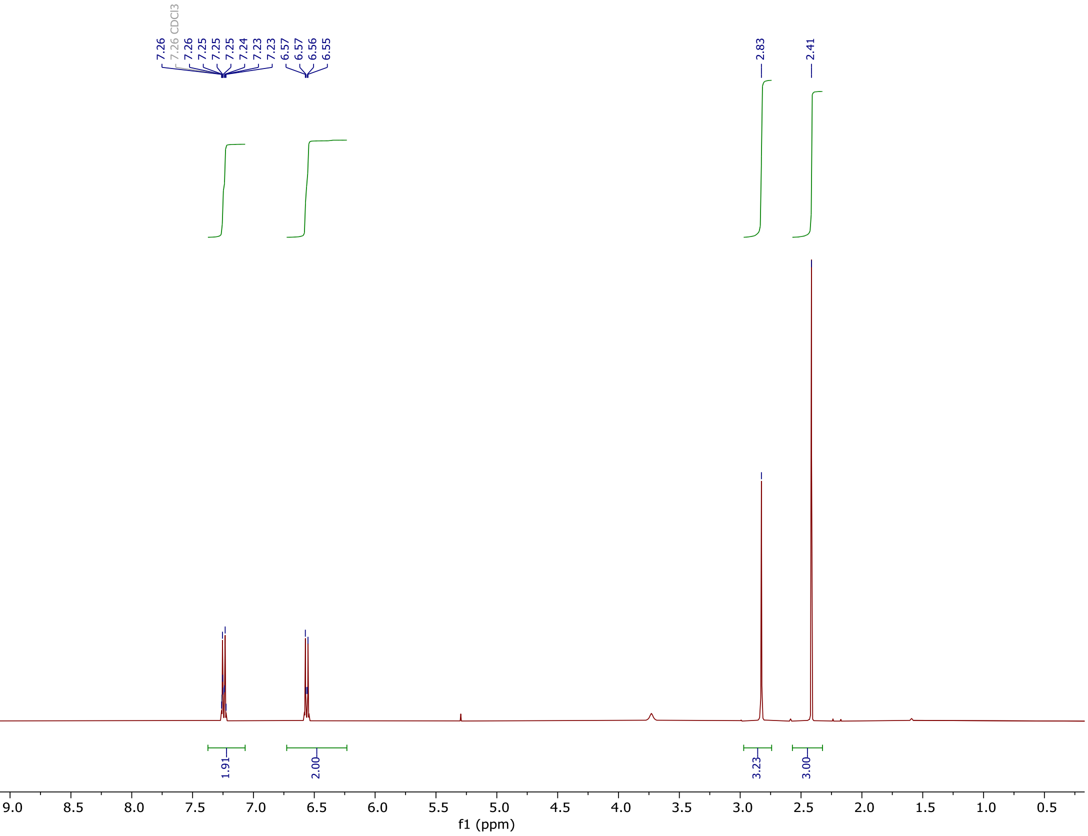

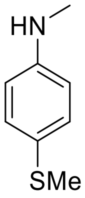

P6

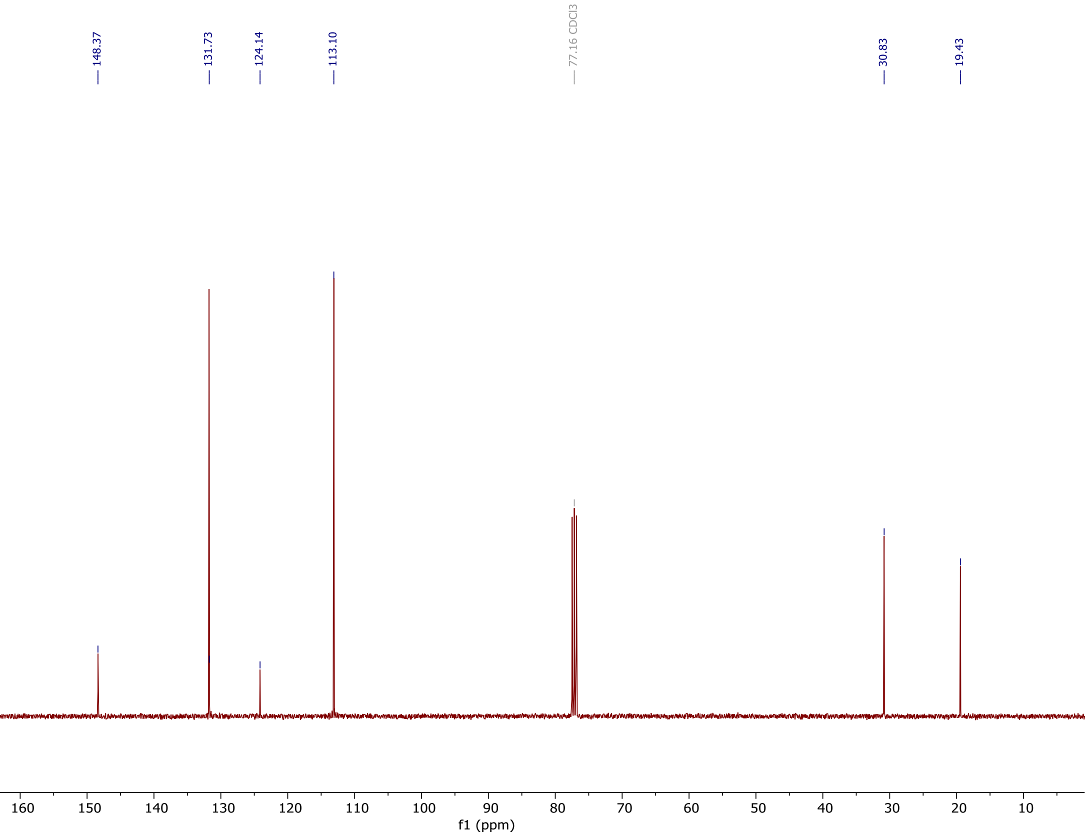

N-Methylbenzen-d_5-amine (P7)

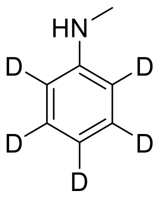

P7

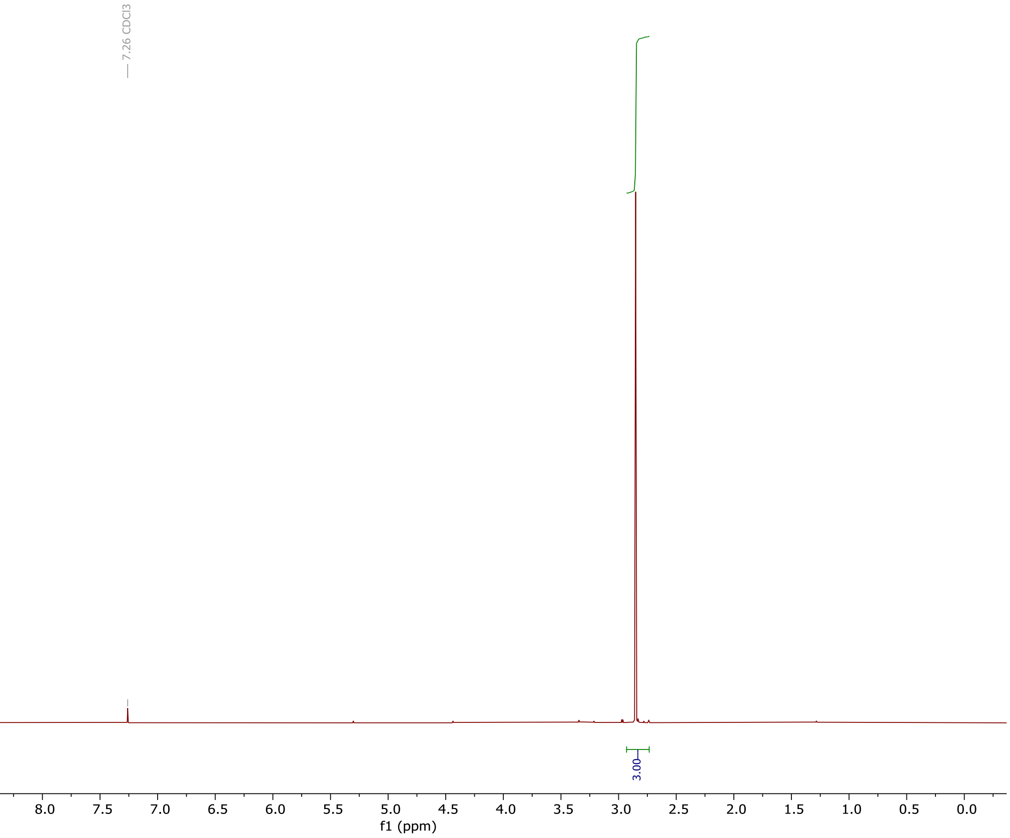

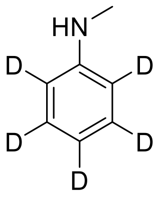

P7

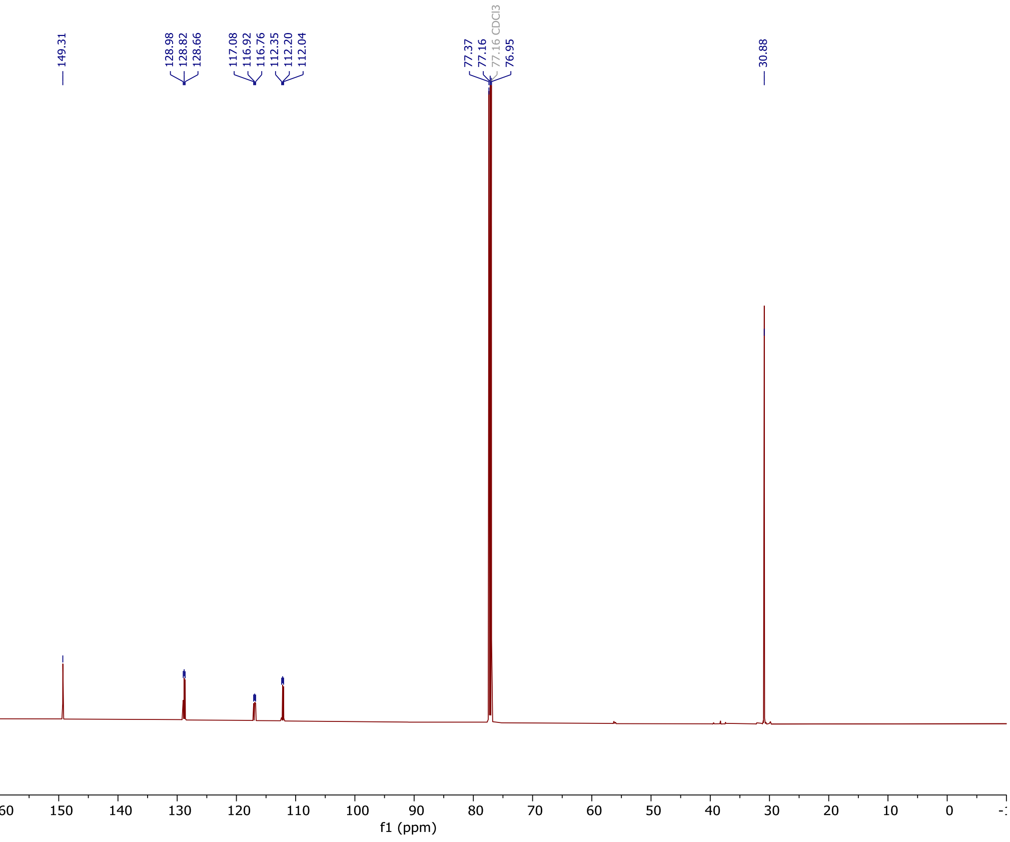

2,4,6-Trichlorophenyl 4,5,6,7-tetrahydrooxepine-2-carboxylate (P8)

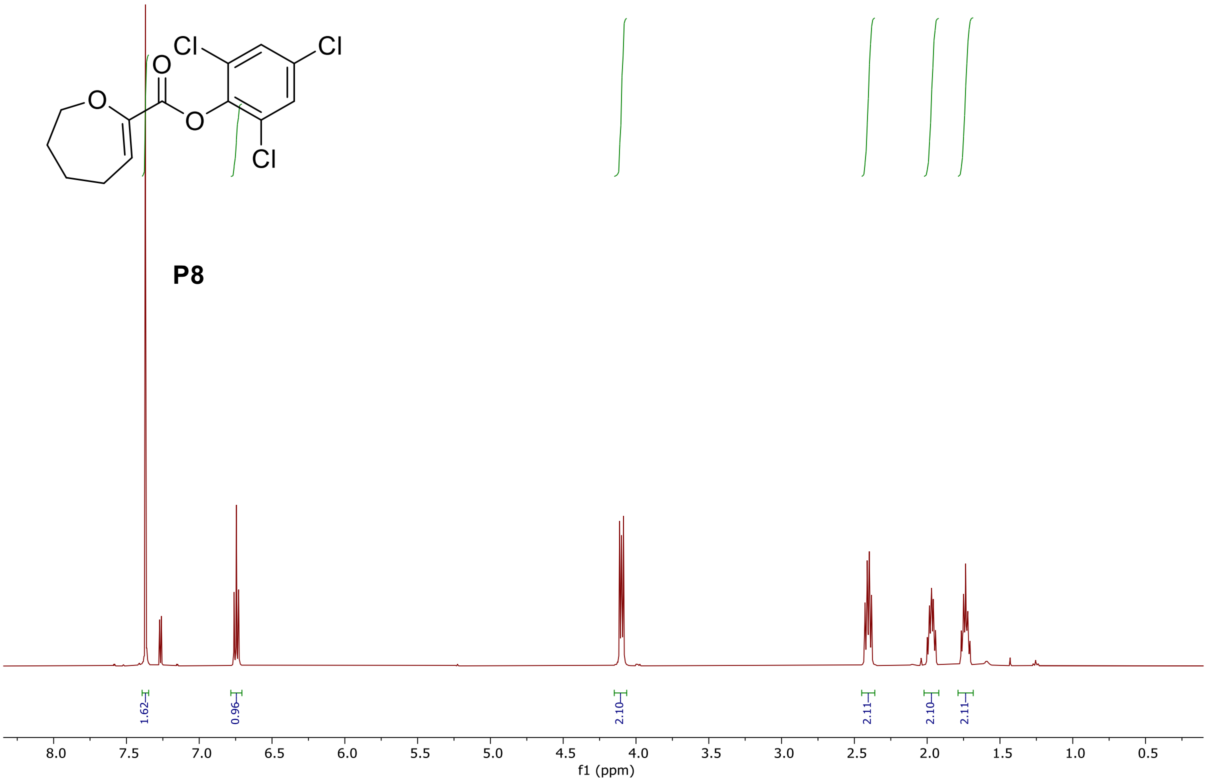

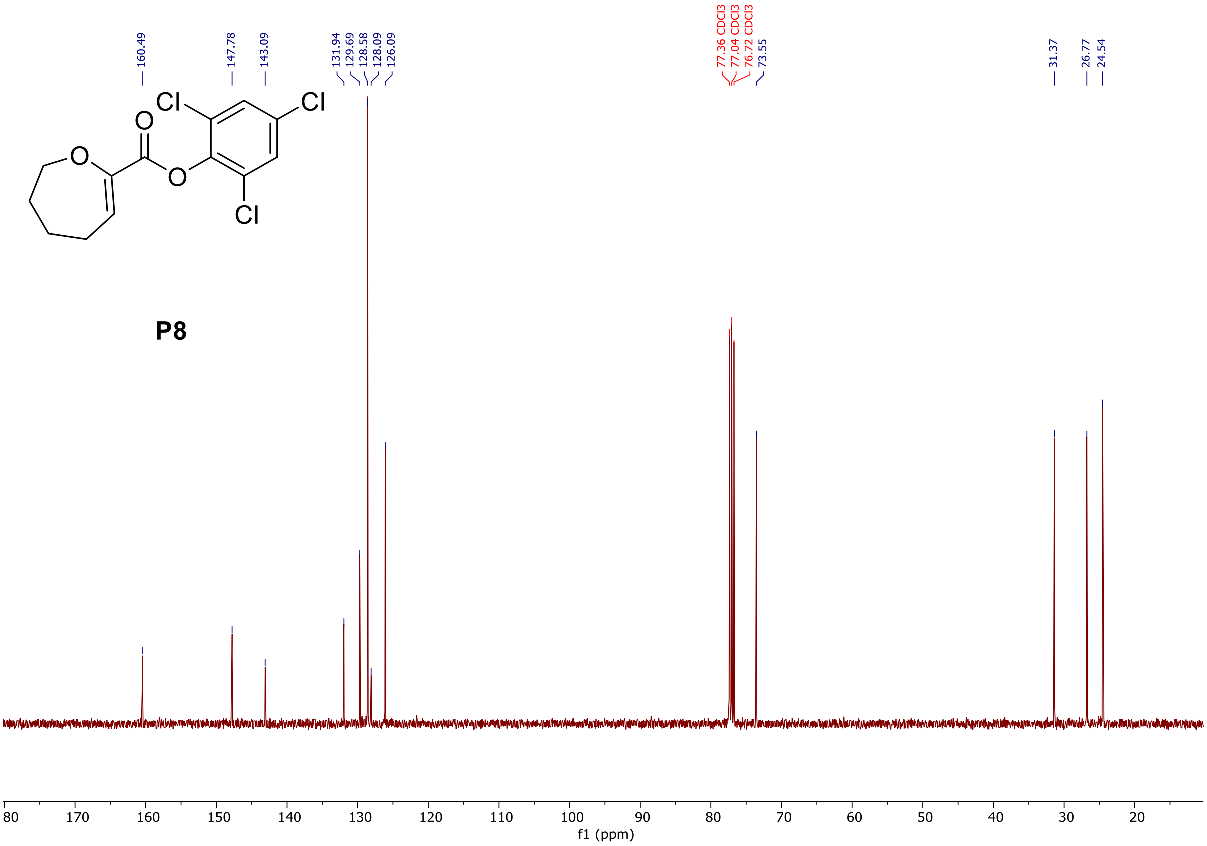

9.3. Substrates

N-Methyl-*N*-phenyl-3,4-dihydro-2*H*-pyran-6-carboxamide (2a)

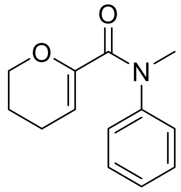

2a

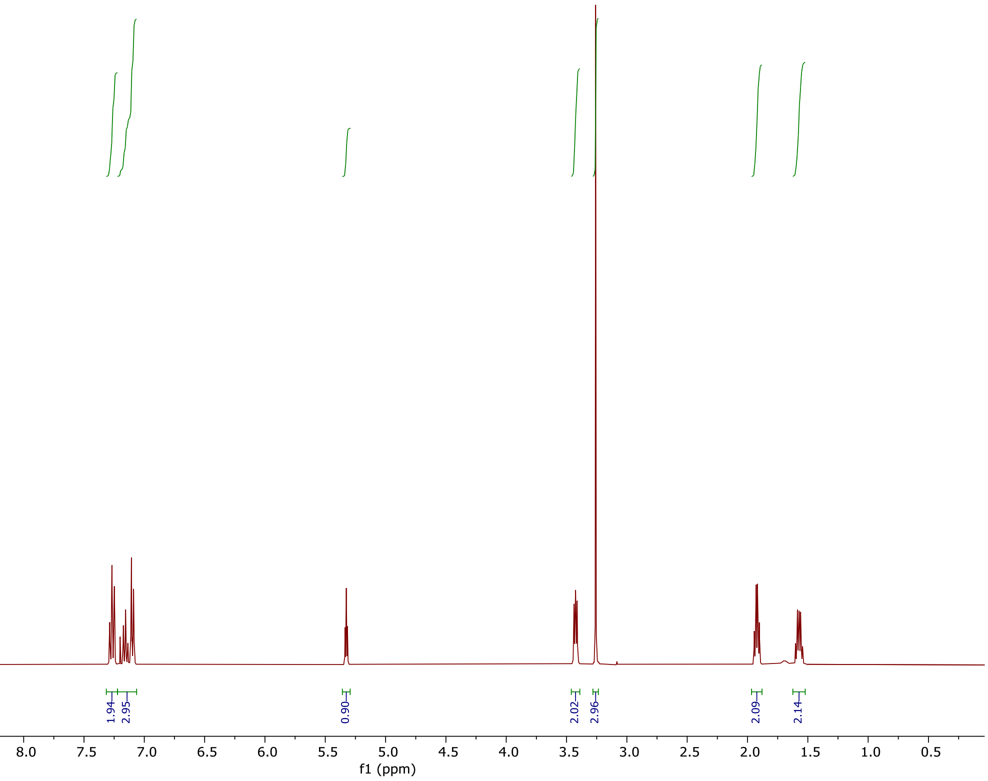

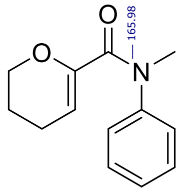

2a

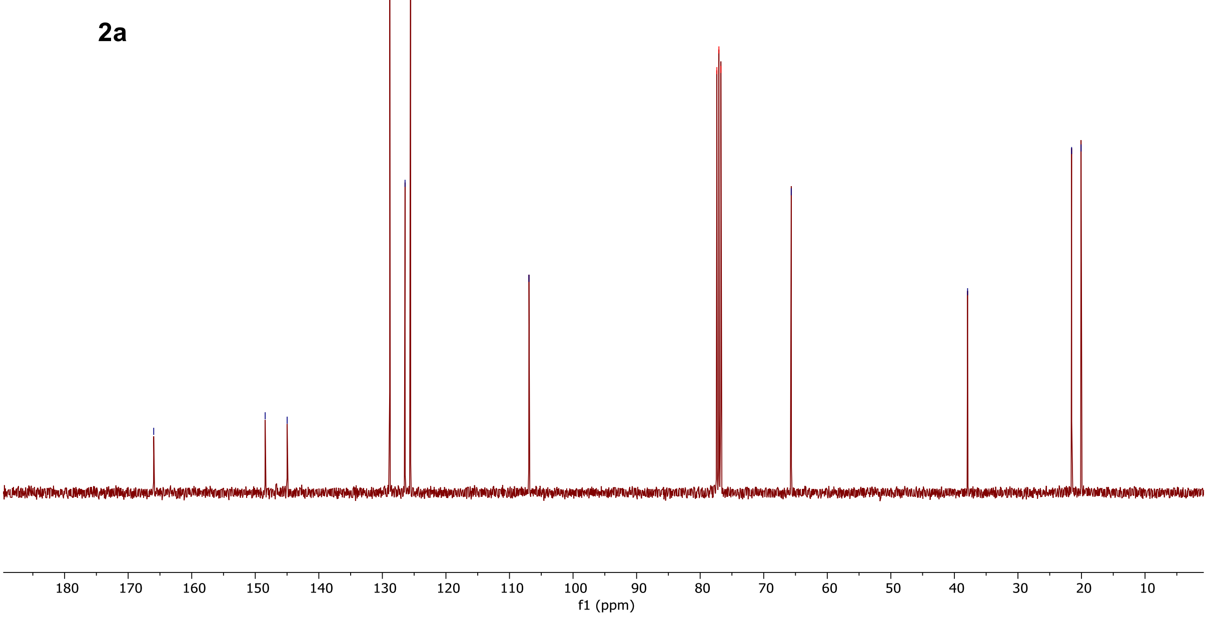

N-Benzyl-*N*-phenyl-3,4-dihydro-2*H*-pyran-6-carboxamide (5a)

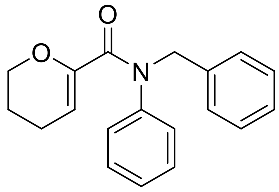

5a

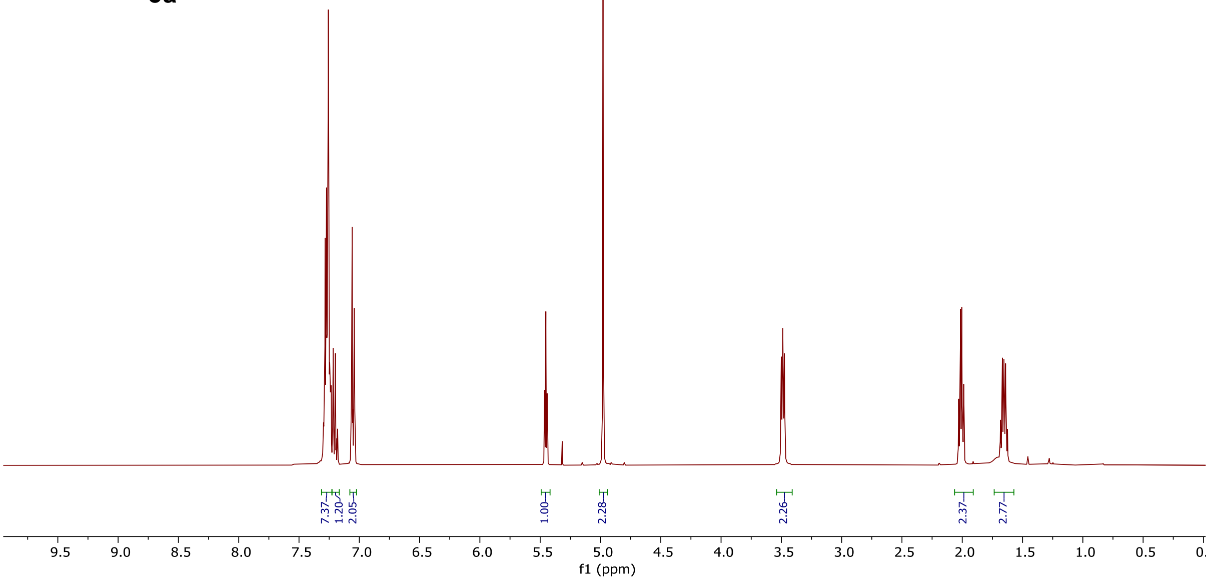

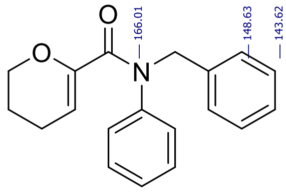

5a

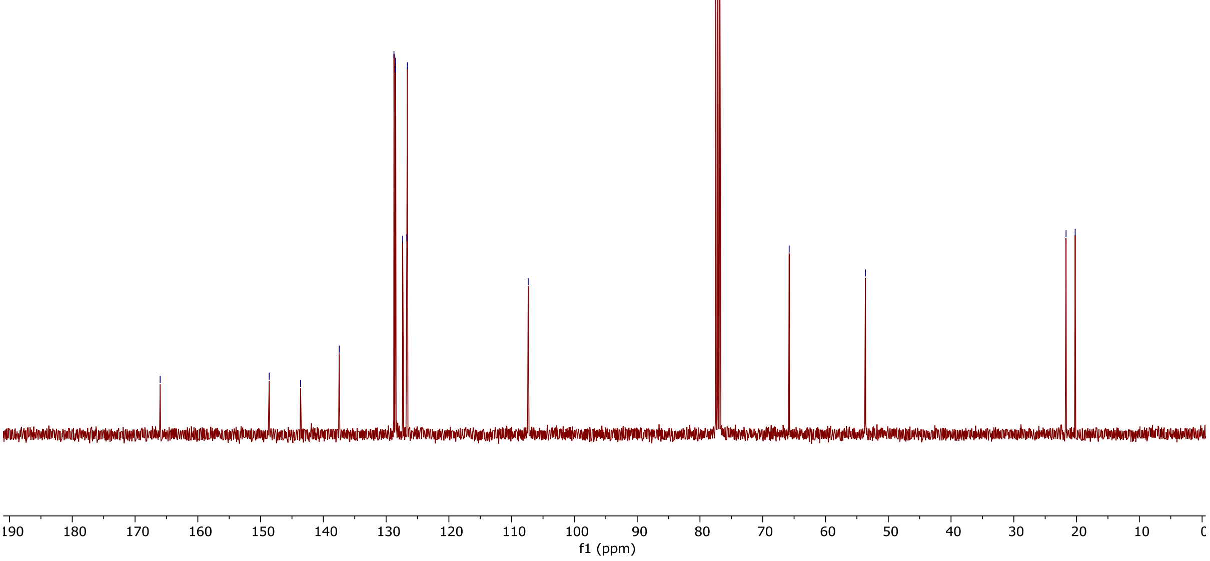

_N_-Isobutyl-_N_-phenyl-3,4-dihydro-2_H_-pyran-6-carboxamide (6a)

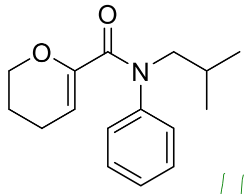

6a

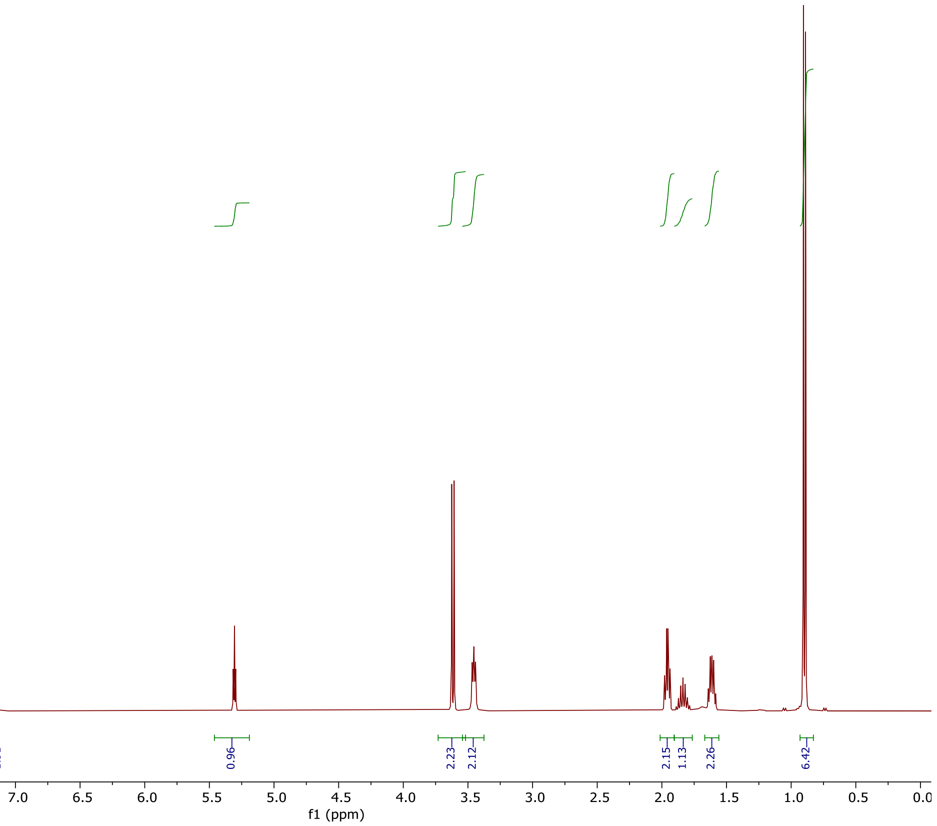

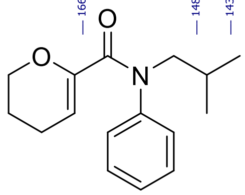

6a

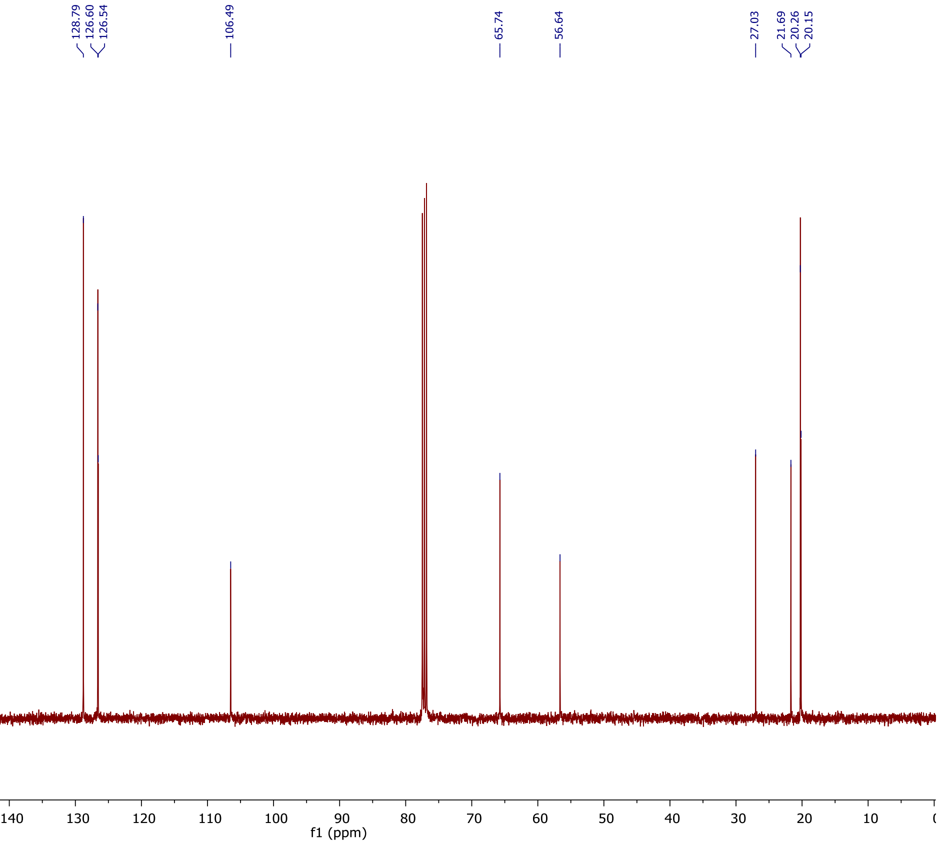

_N_-Phenyl-_N_-propyl-3,4-dihydro-2_H_-pyran-6-carboxamide (29)

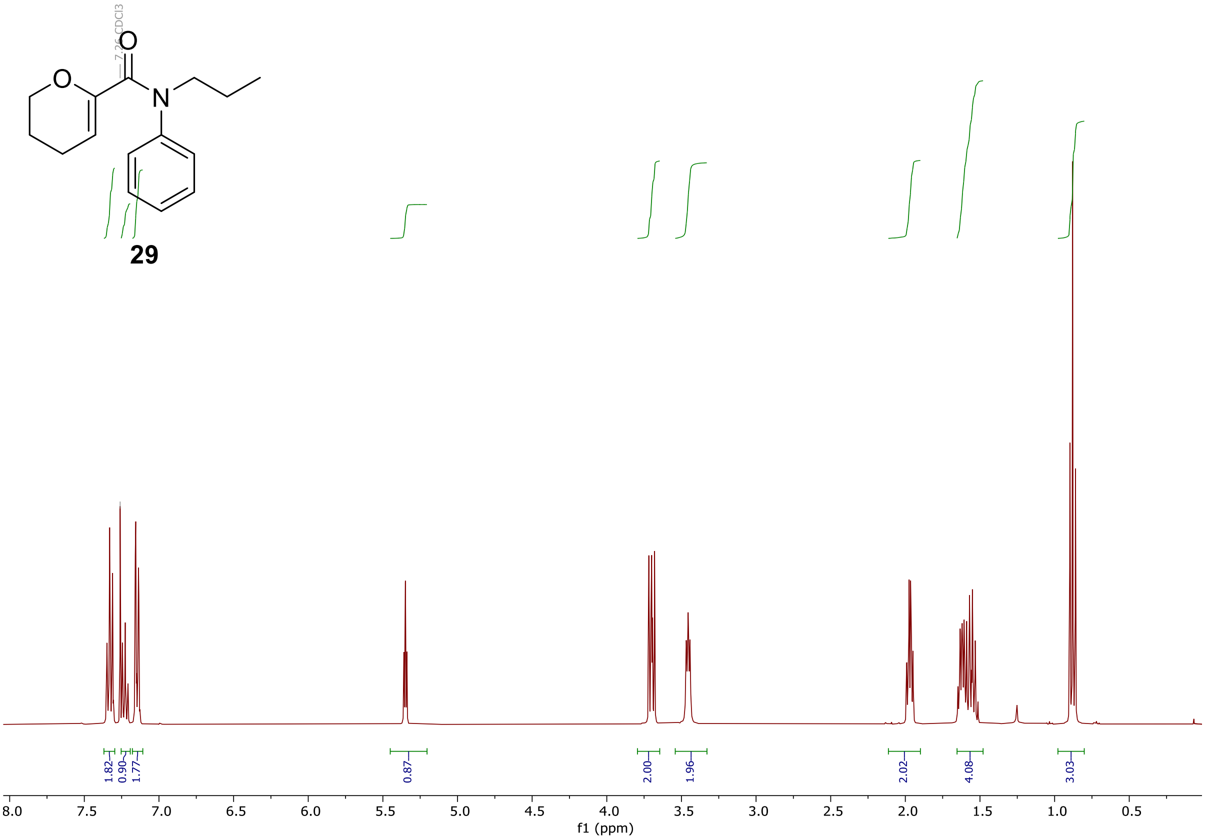

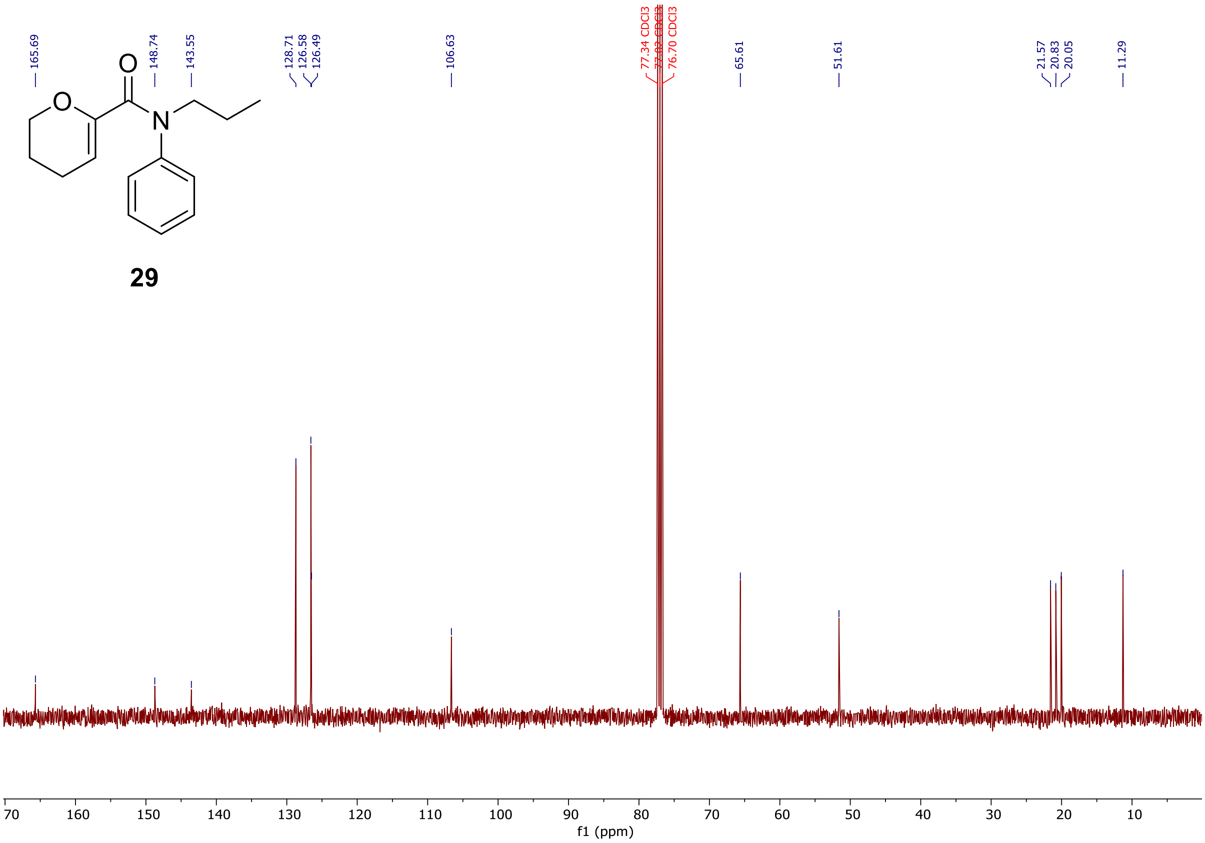

N-Allyl-*N*-phenyl-3,4-dihydro-2*H*-pyran-6-carboxamide (8a)

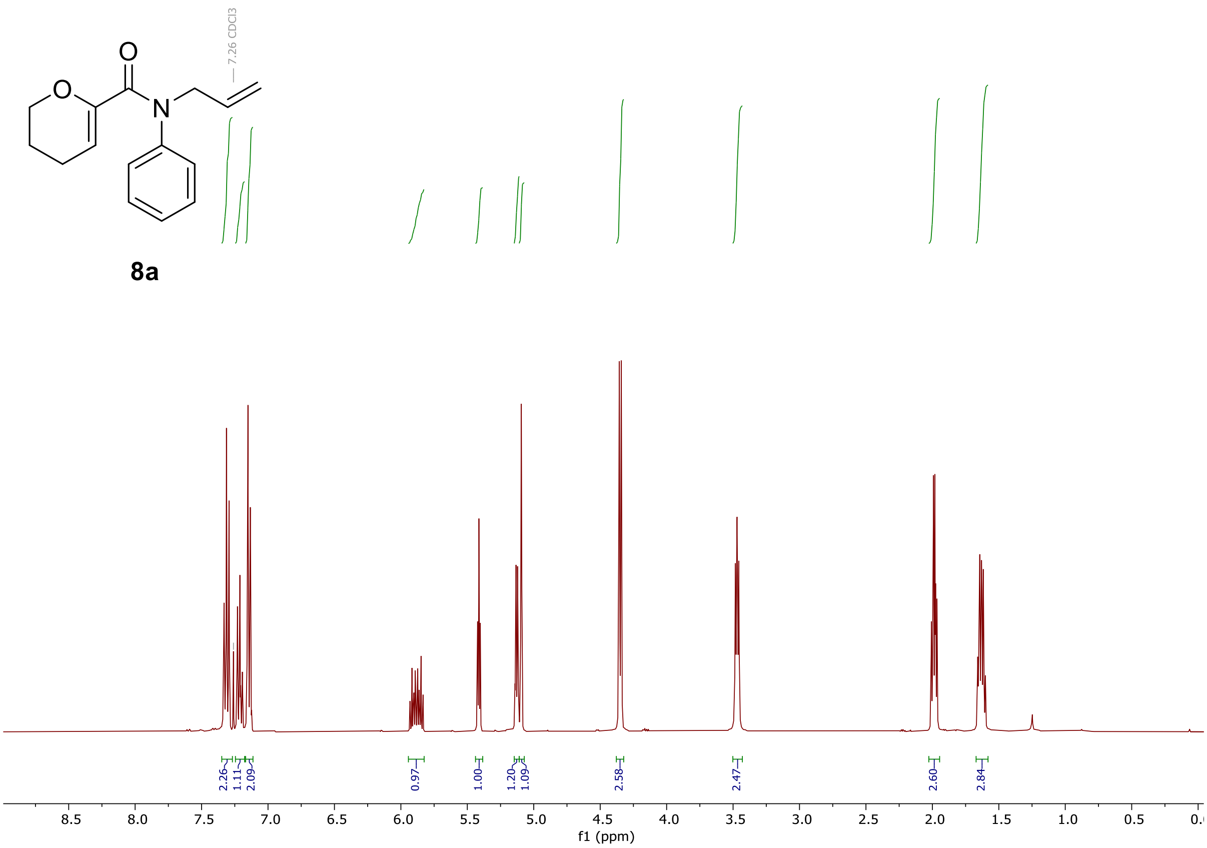

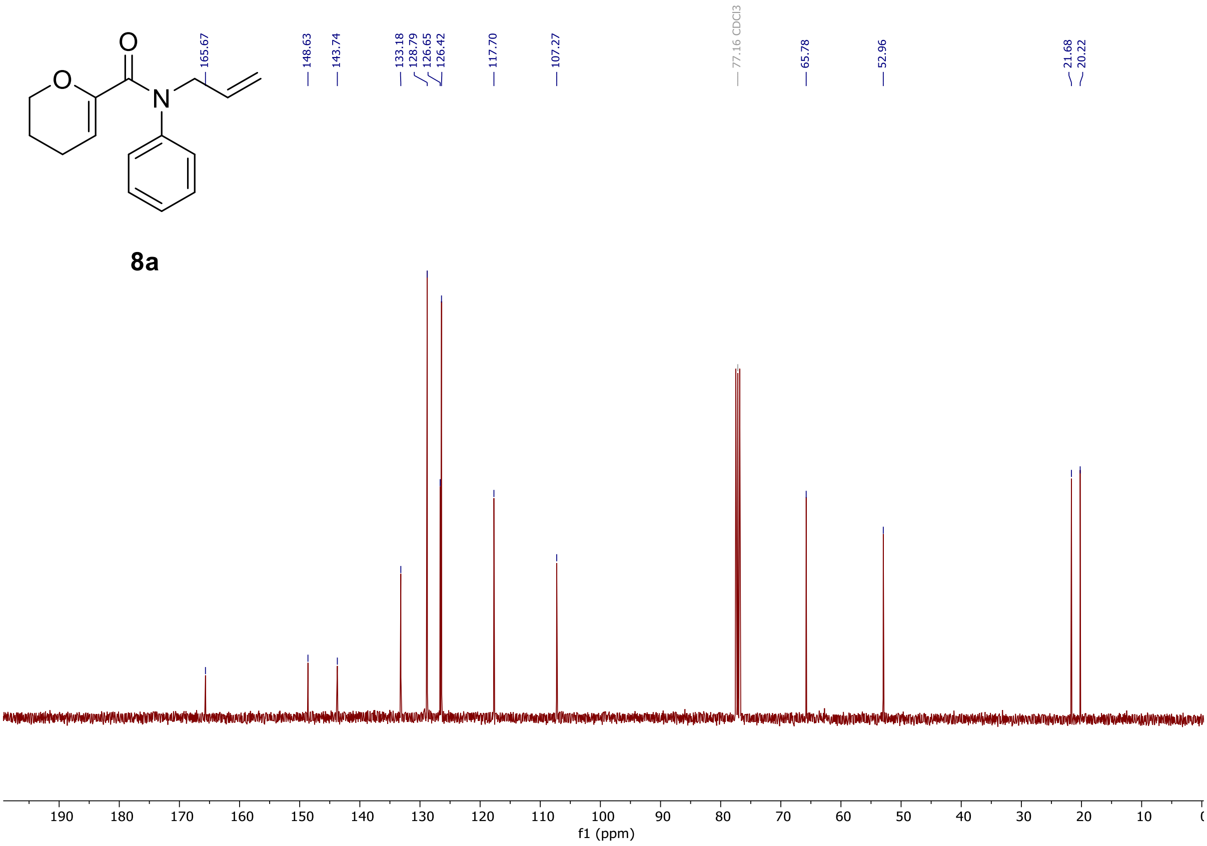

(3,4-Dihydro-2*H*-pyran-6-yl)(3,4-dihydroquinolin-1(2*H*)-yl)methanone (9a)

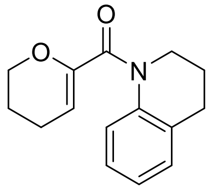

9a

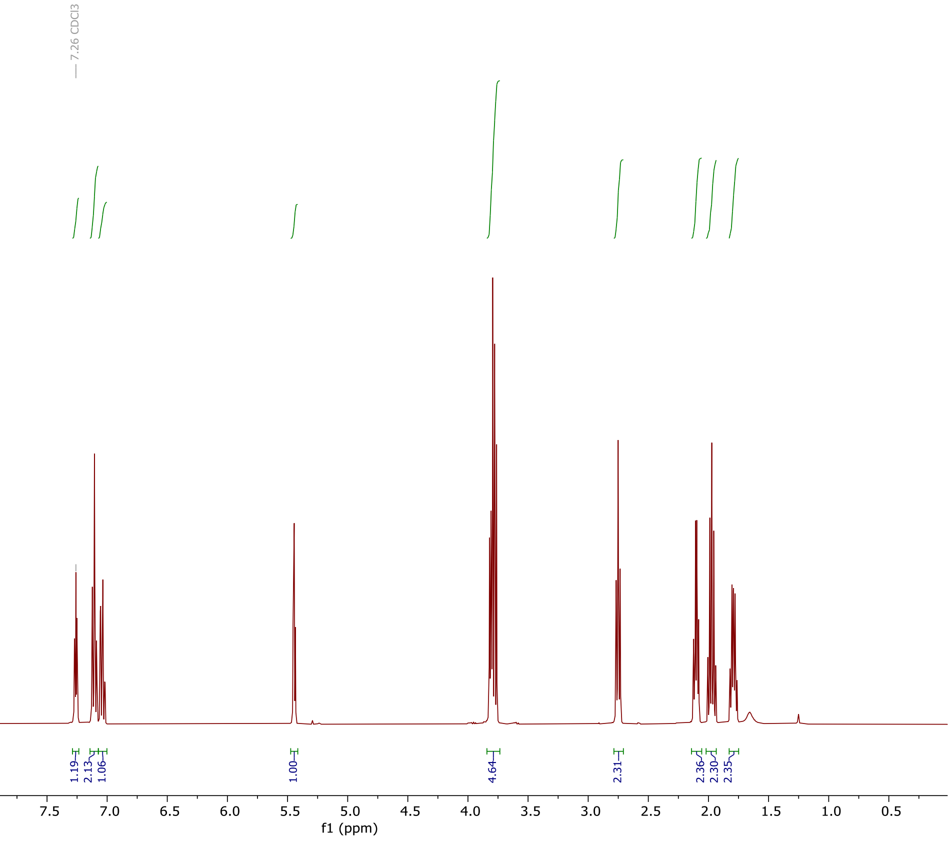

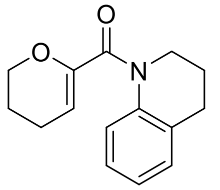

9a

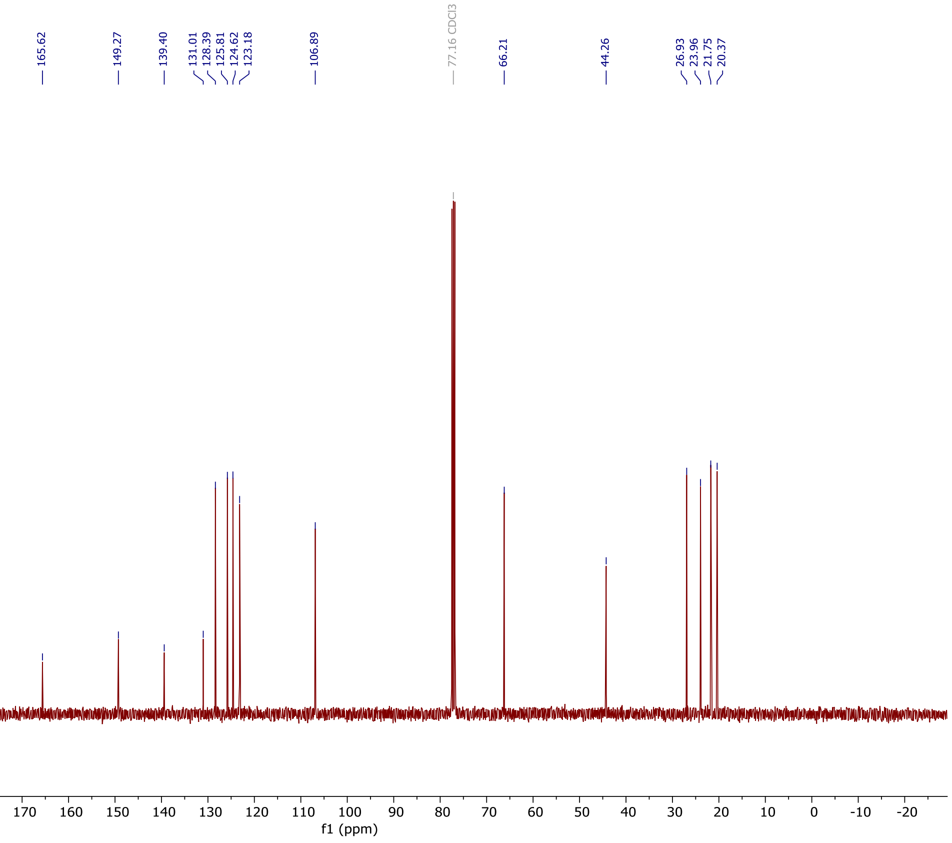

_N_-(4-Methoxybenzyl)-_N_-phenyl-3,4-dihydro-2_H_-pyran-6-carboxamide (10a)

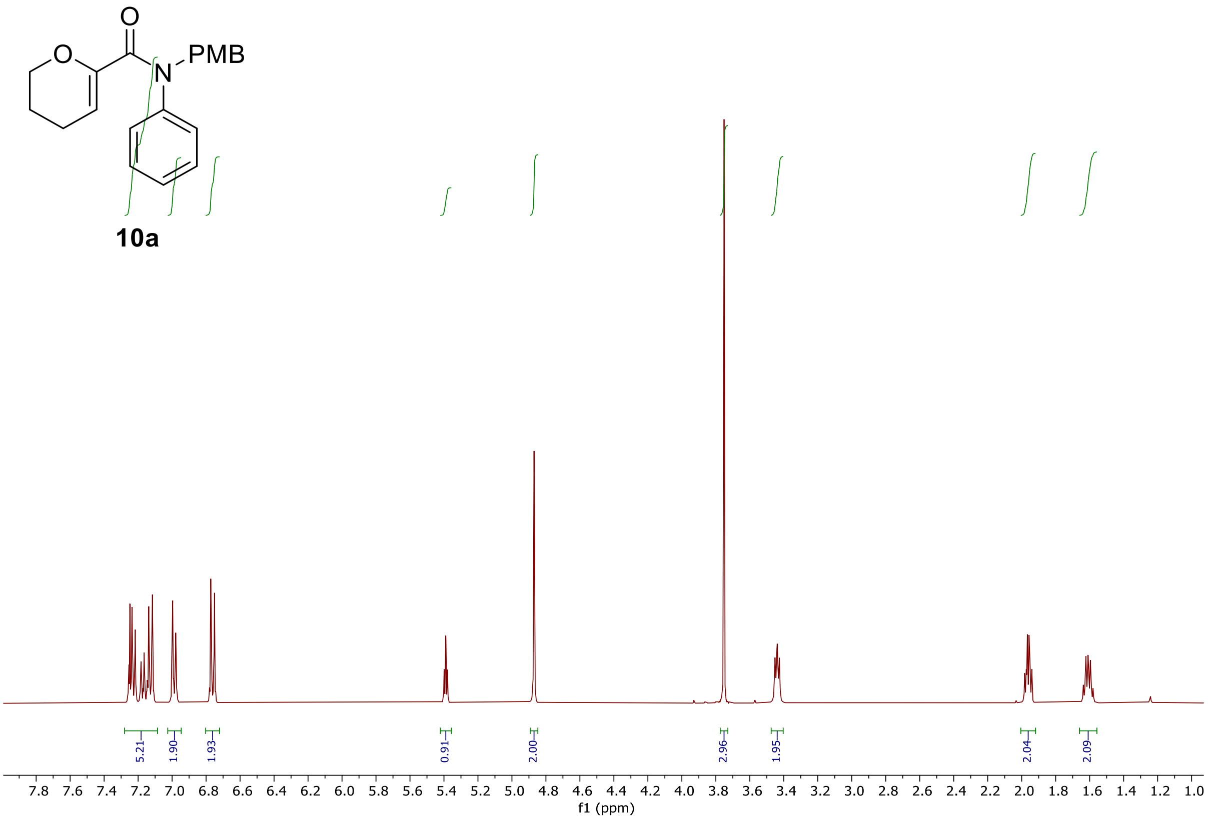

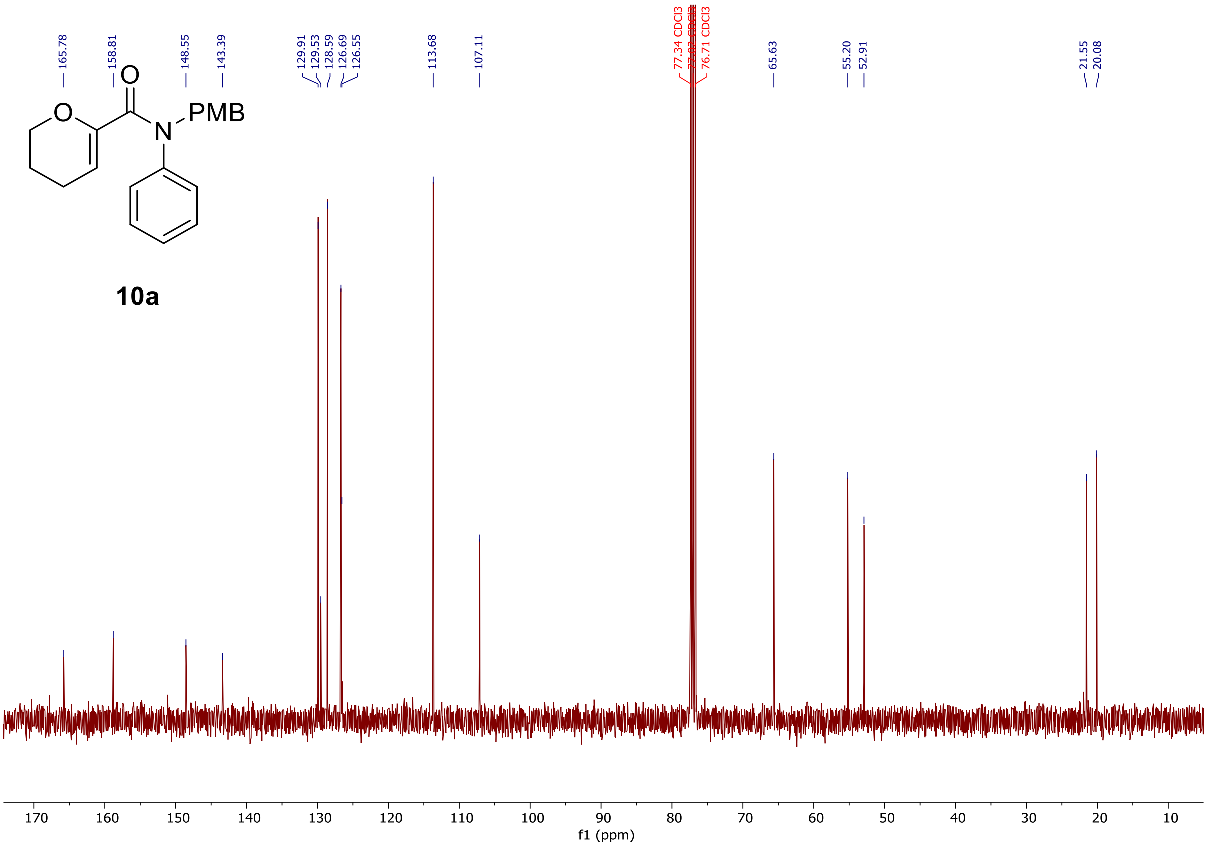

***N*-(3-Fluorophenyl)-*N*-methyl-3,4-dihydro-2*H*-pyran-6-carboxamide (11a)**

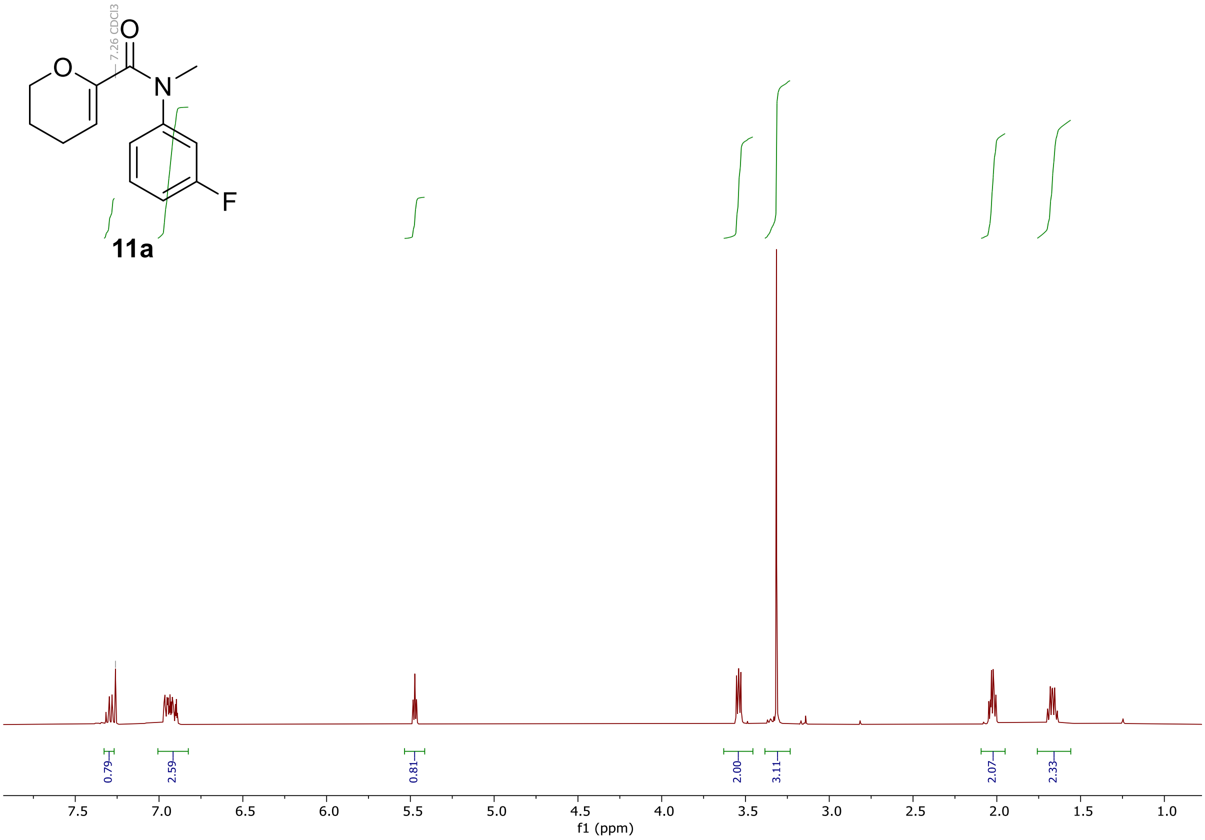

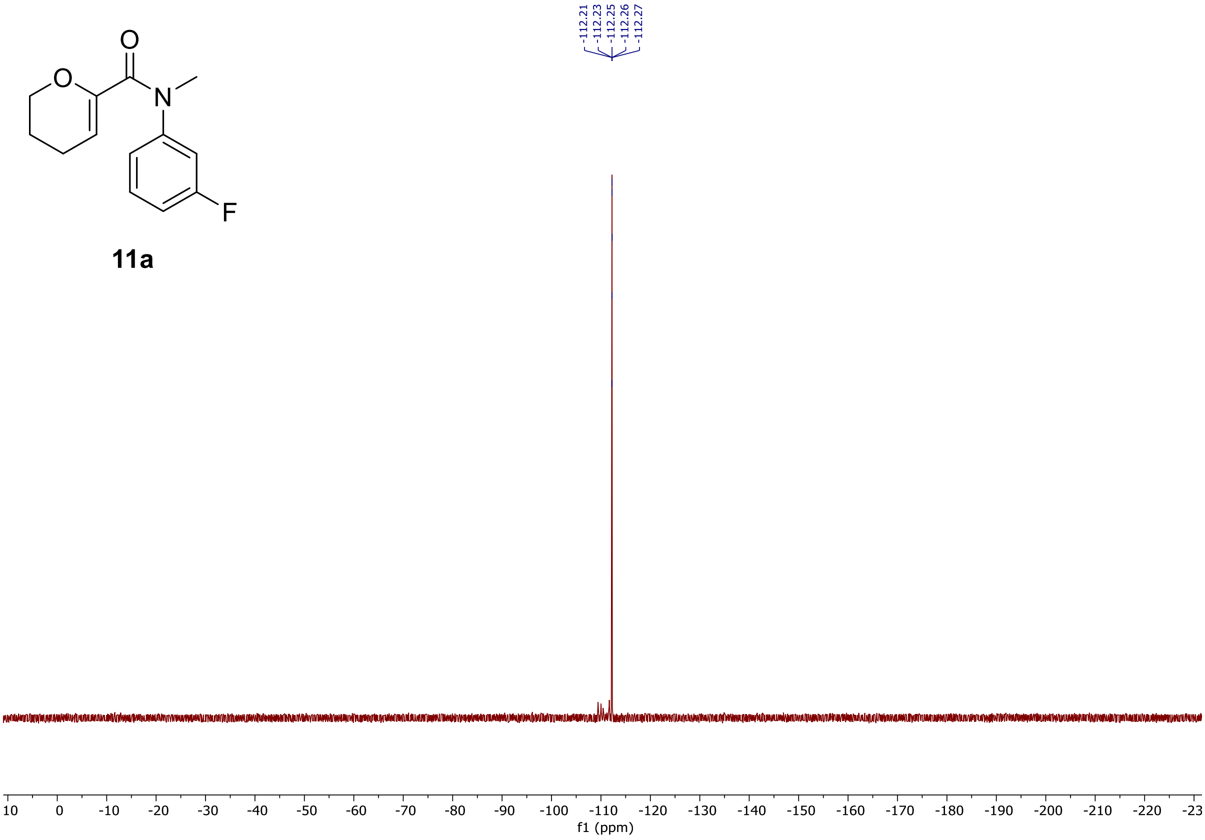

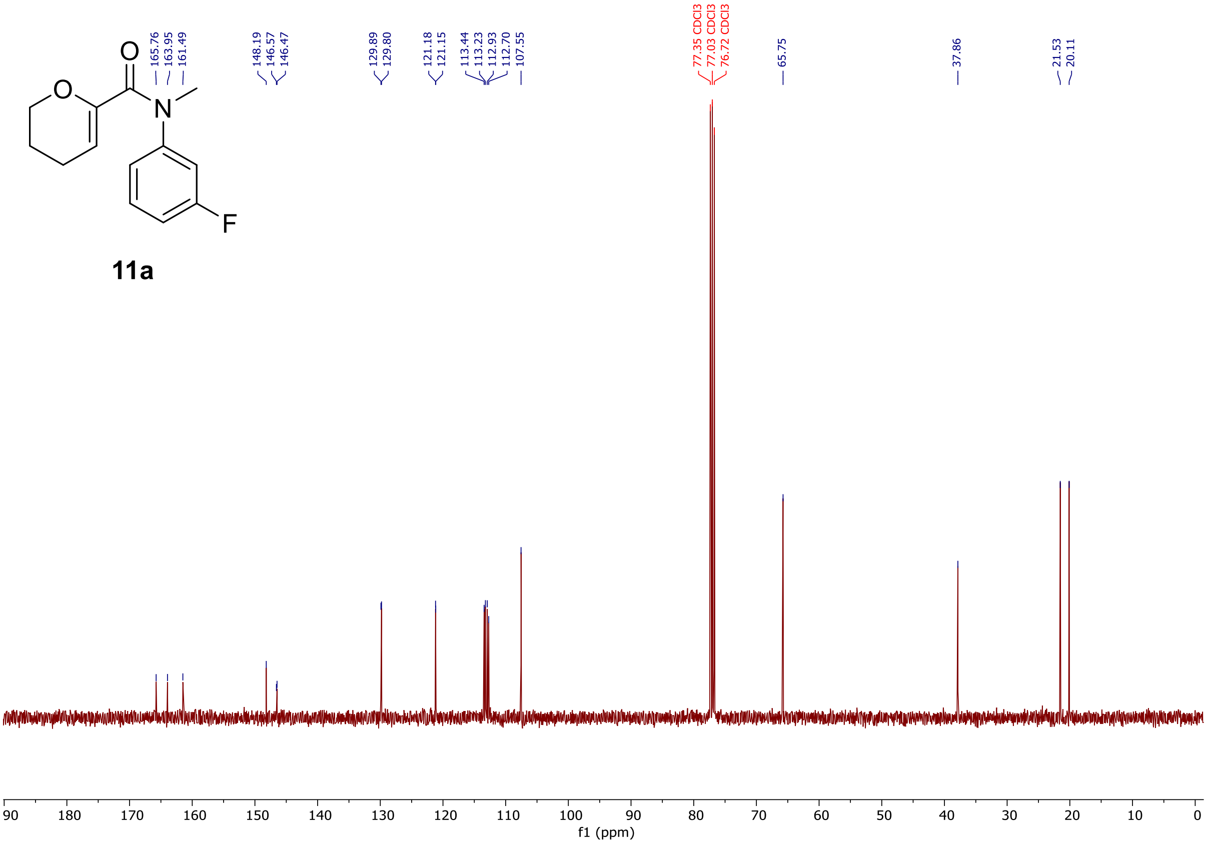

N-(3-Chlorophenyl)-*N*-methyl-3,4-dihydro-2*H*-pyran-6-carboxamide (12a)

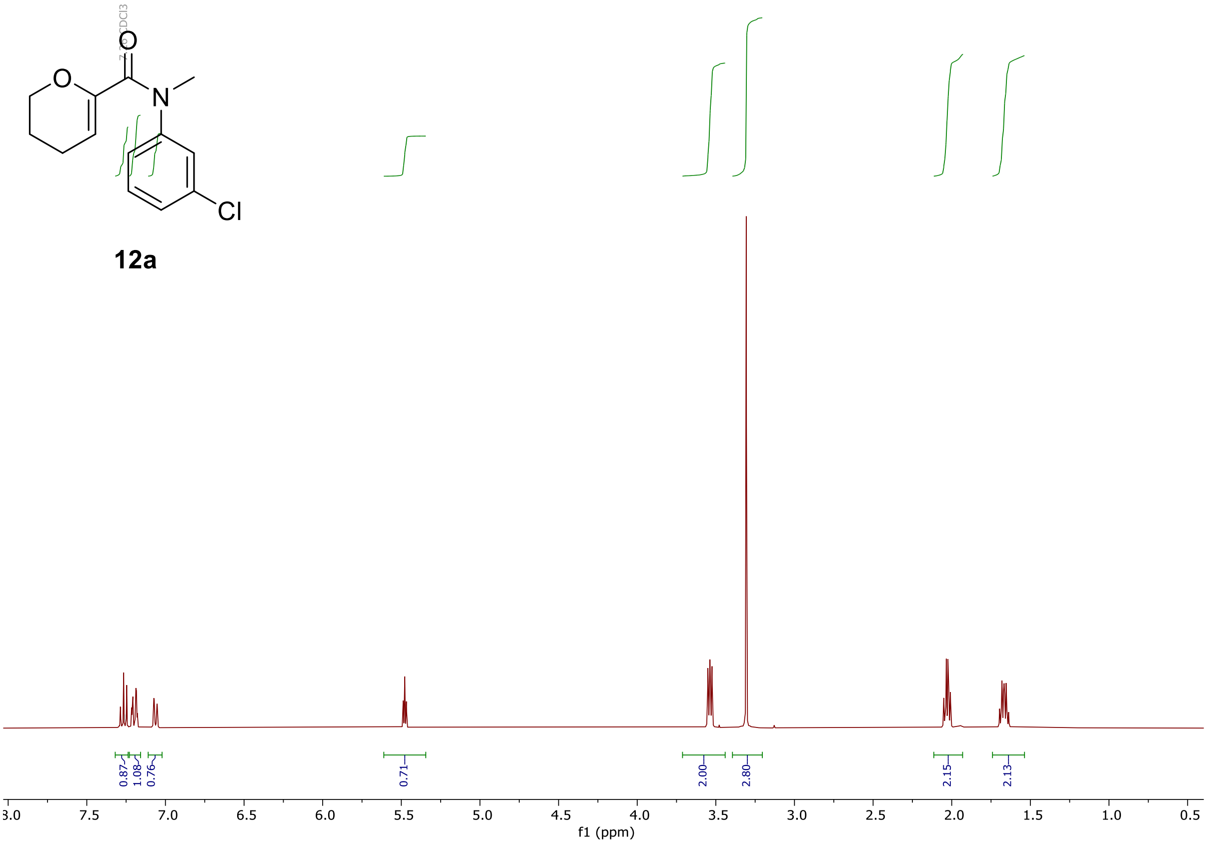

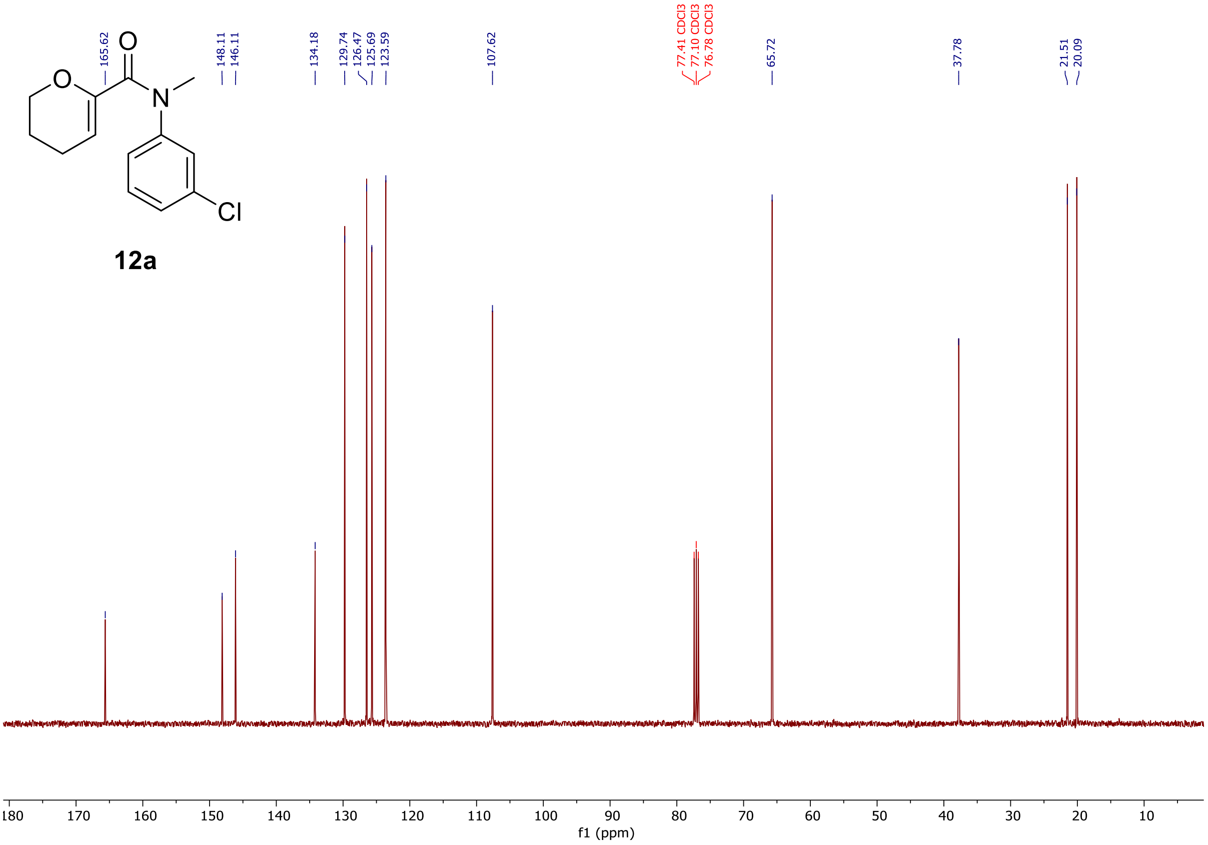

12a

***N*-(3-Methoxyphenyl)-*N*-methyl-3,4-dihydro-2*H*-pyran-6-carboxamide (13a)**

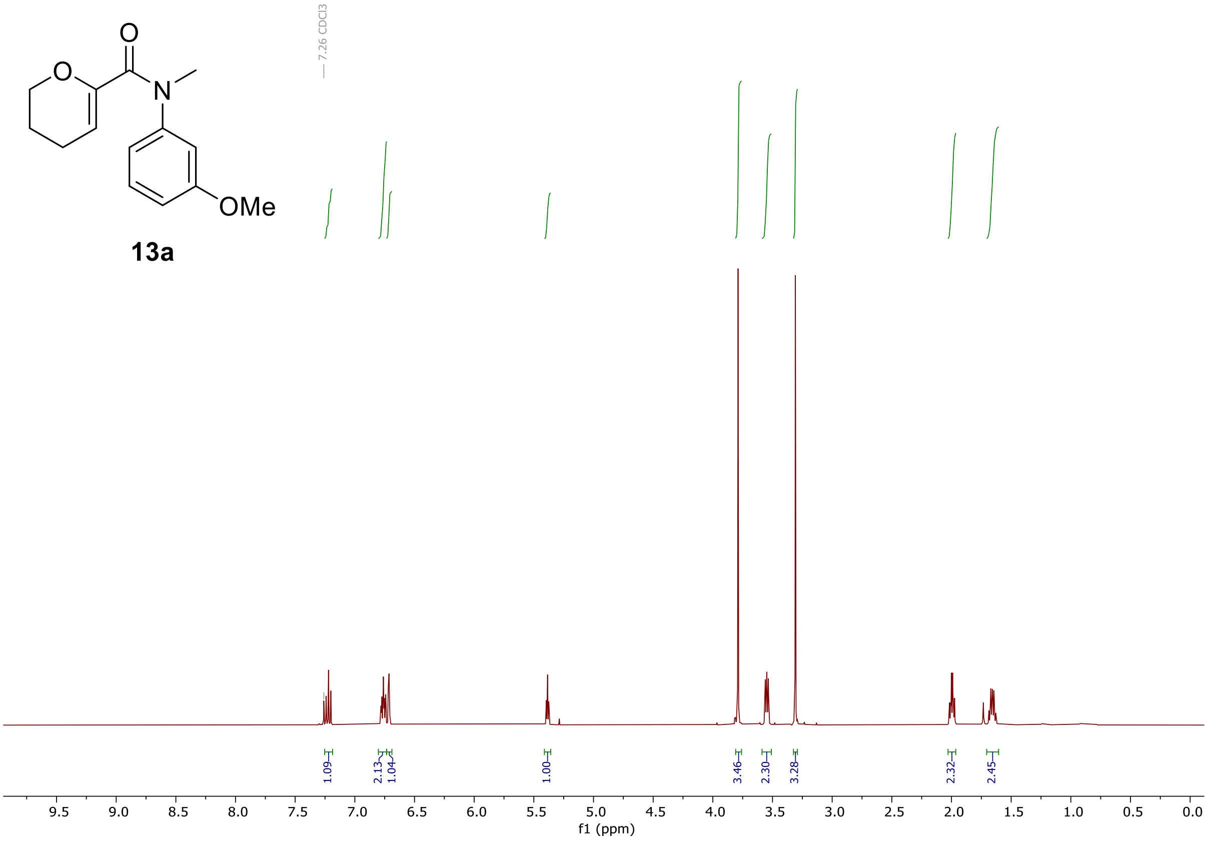

13a

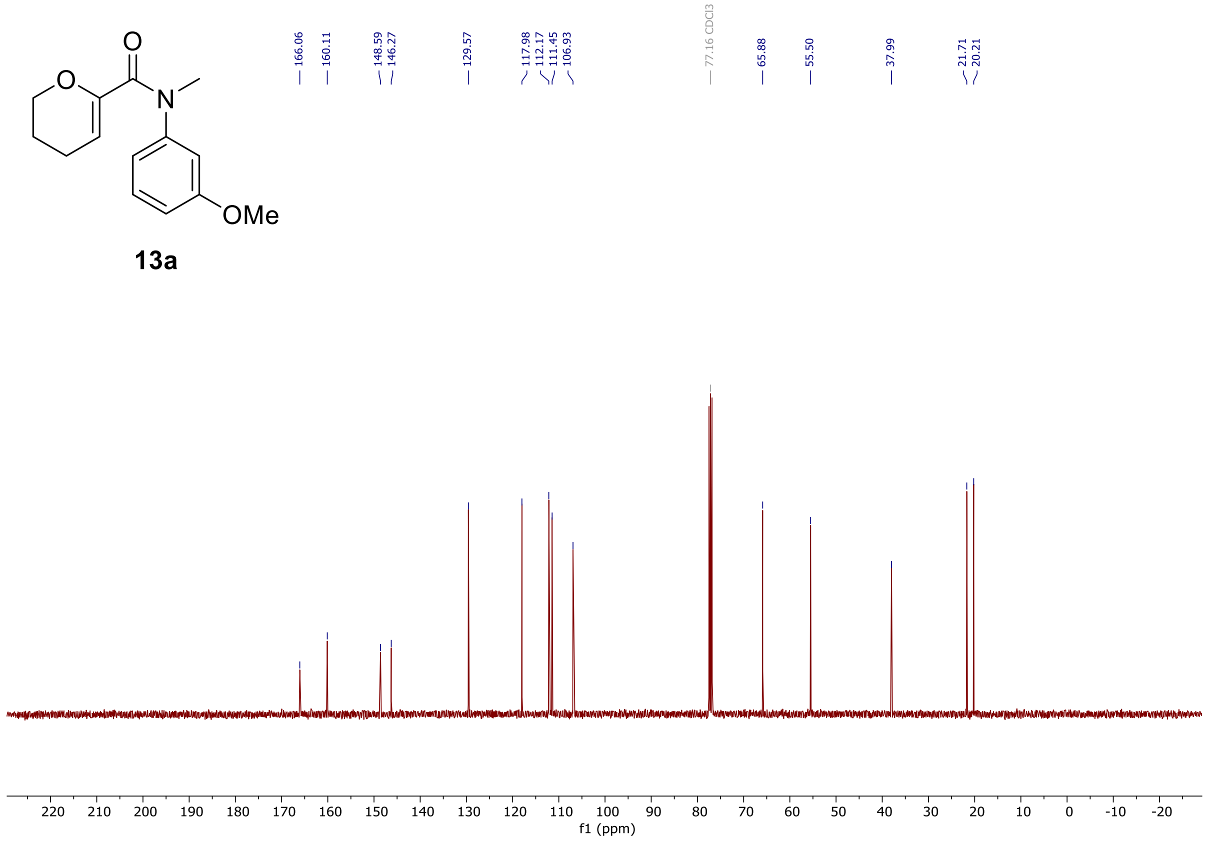

N-Methyl-*N*-(*m*-tolyl)-3,4-dihydro-2*H*-pyran-6-carboxamide (14a)

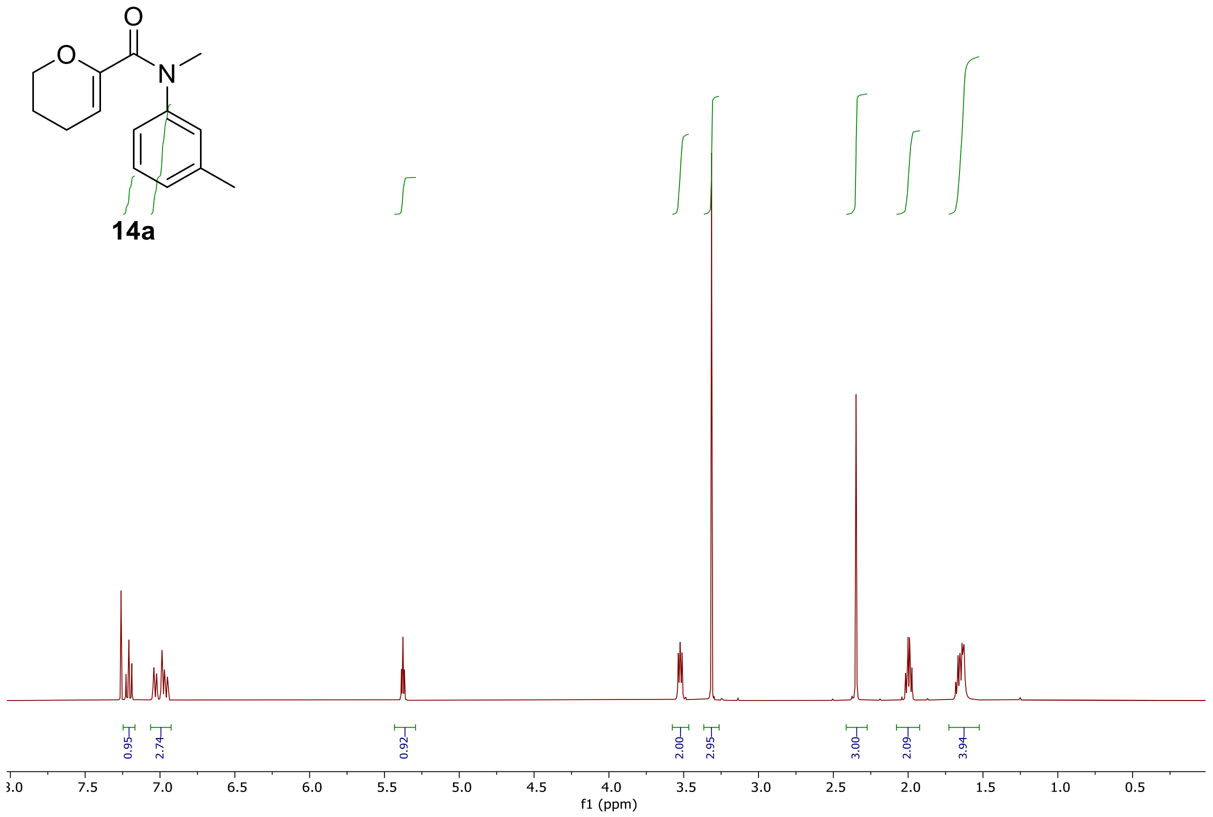

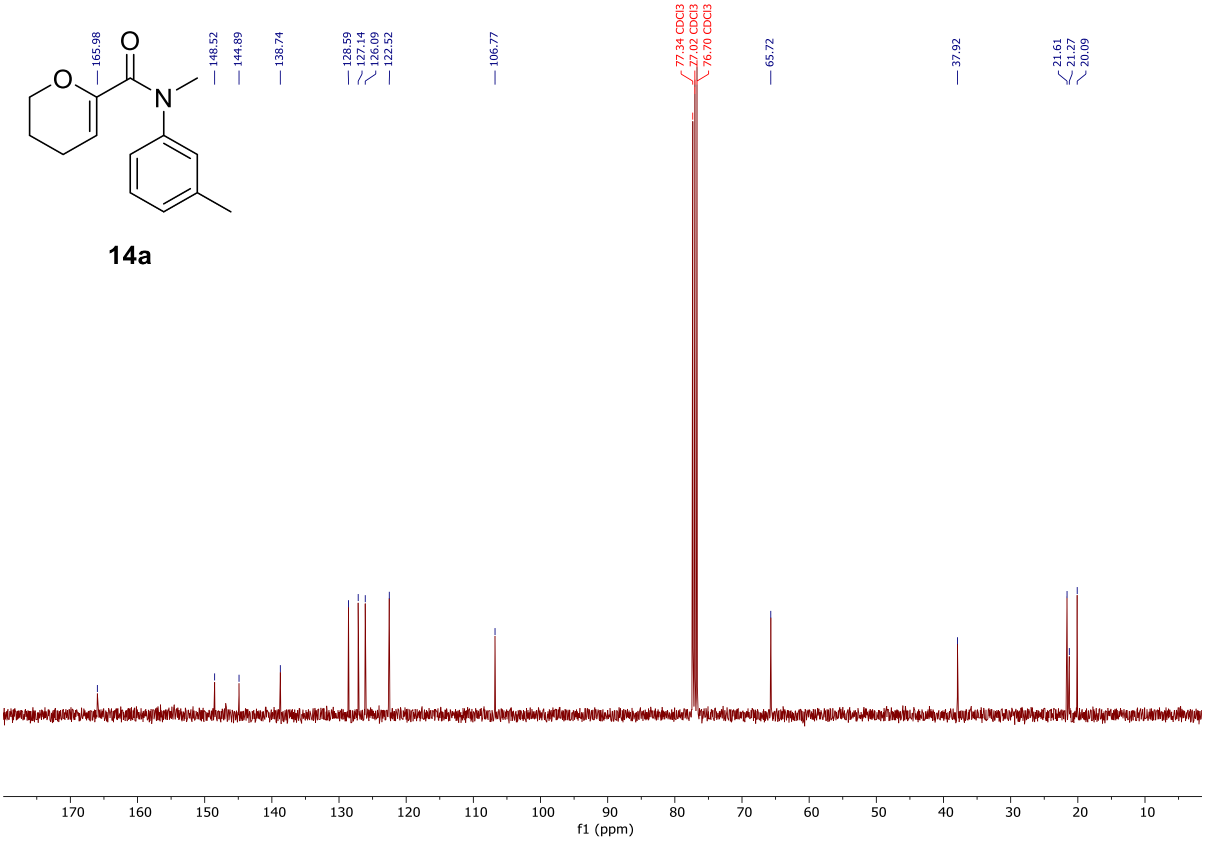

N-(3,5-Difluorophenyl)-*N*-methyl-3,4-dihydro-2*H*-pyran-6-carboxamide (15a)

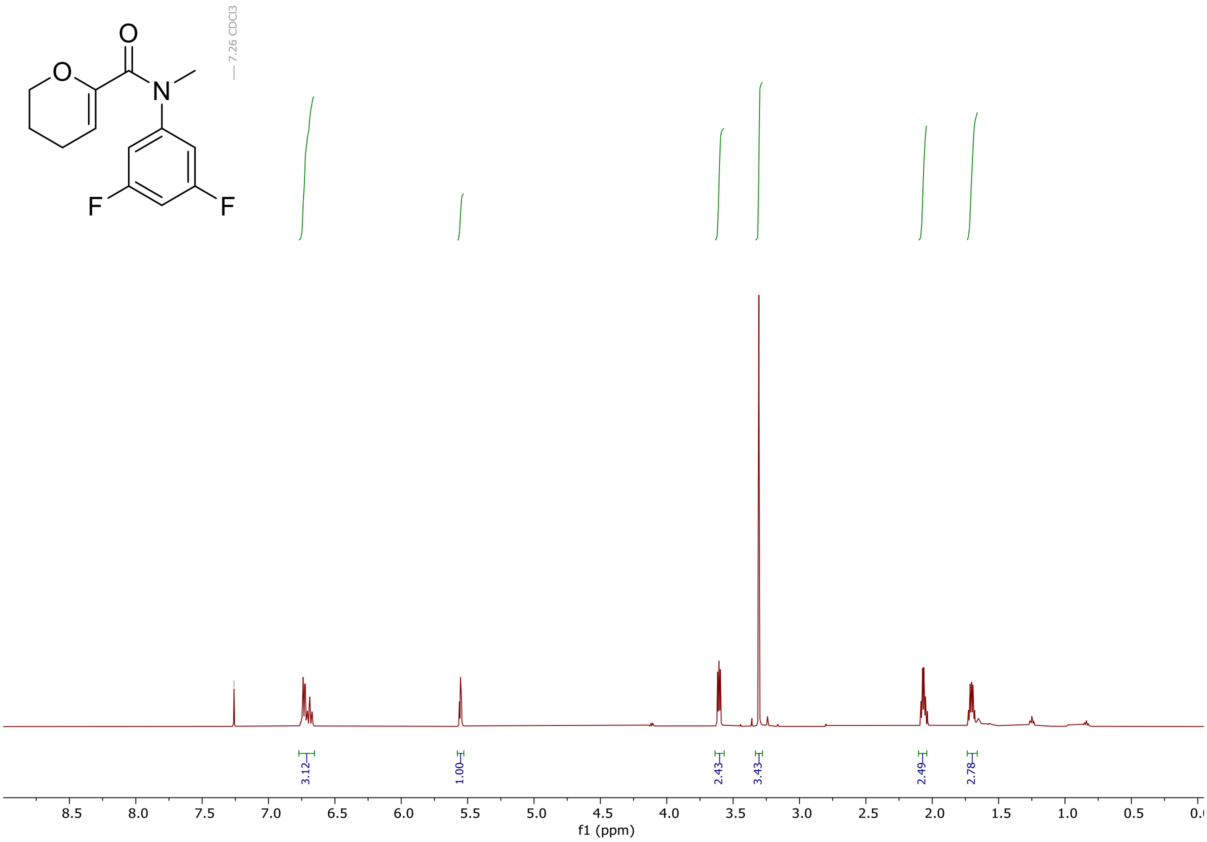

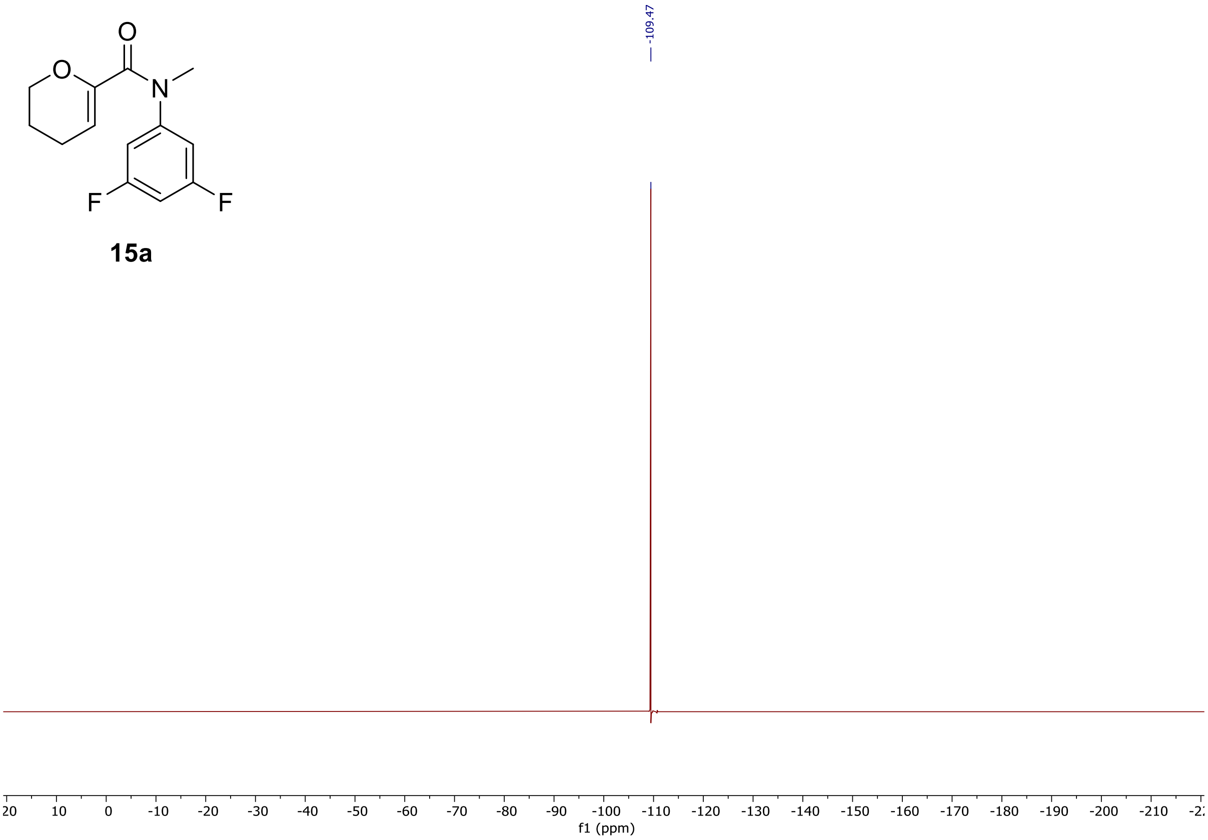
-109.47
f1 (ppm)

15a

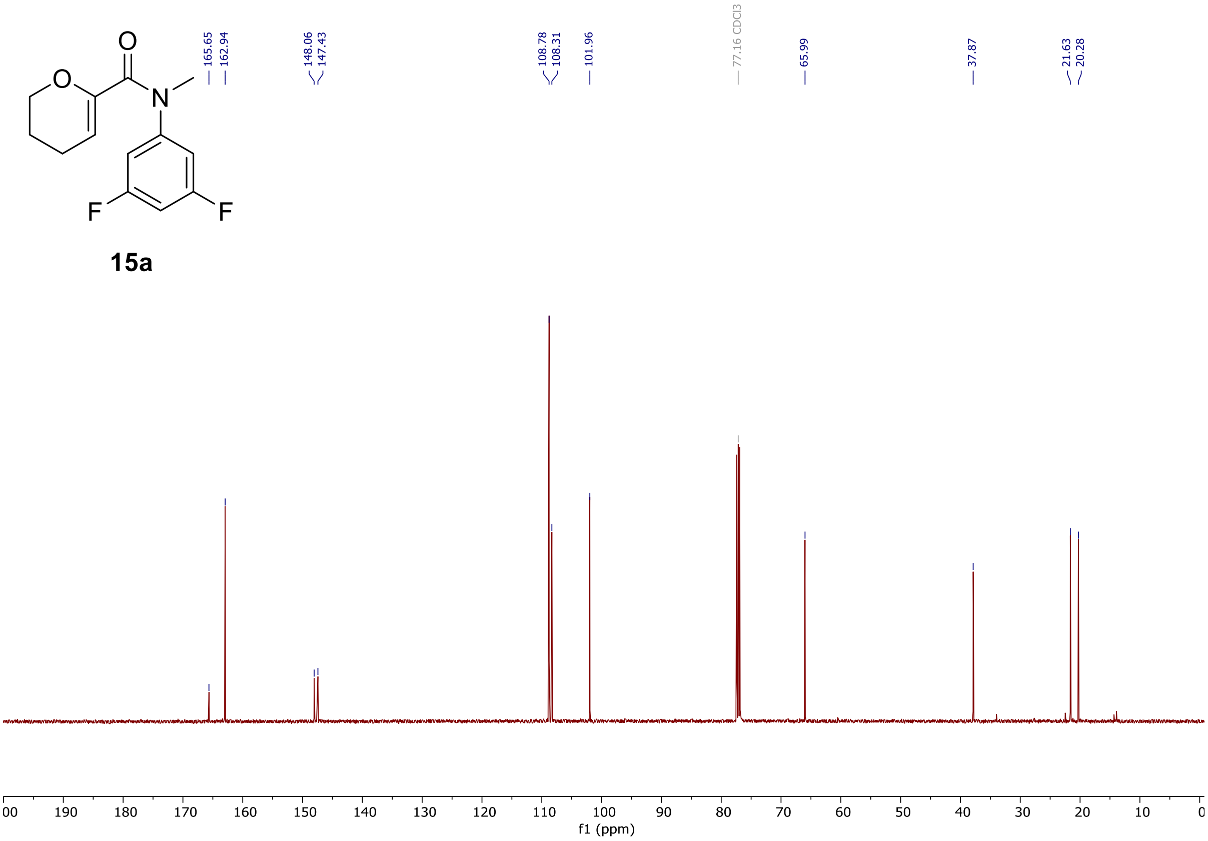
165.65
162.94
148.06
147.43
108.78
108.31
101.96
77.16 CDCl3
65.99
37.87
21.63
20.28
f1 (ppm)

15a

_N_-(4-Fluorophenyl)-_N_-methyl-3,4-dihydro-2_H_-pyran-6-carboxamide (16a)

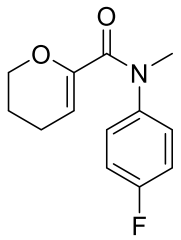

16a

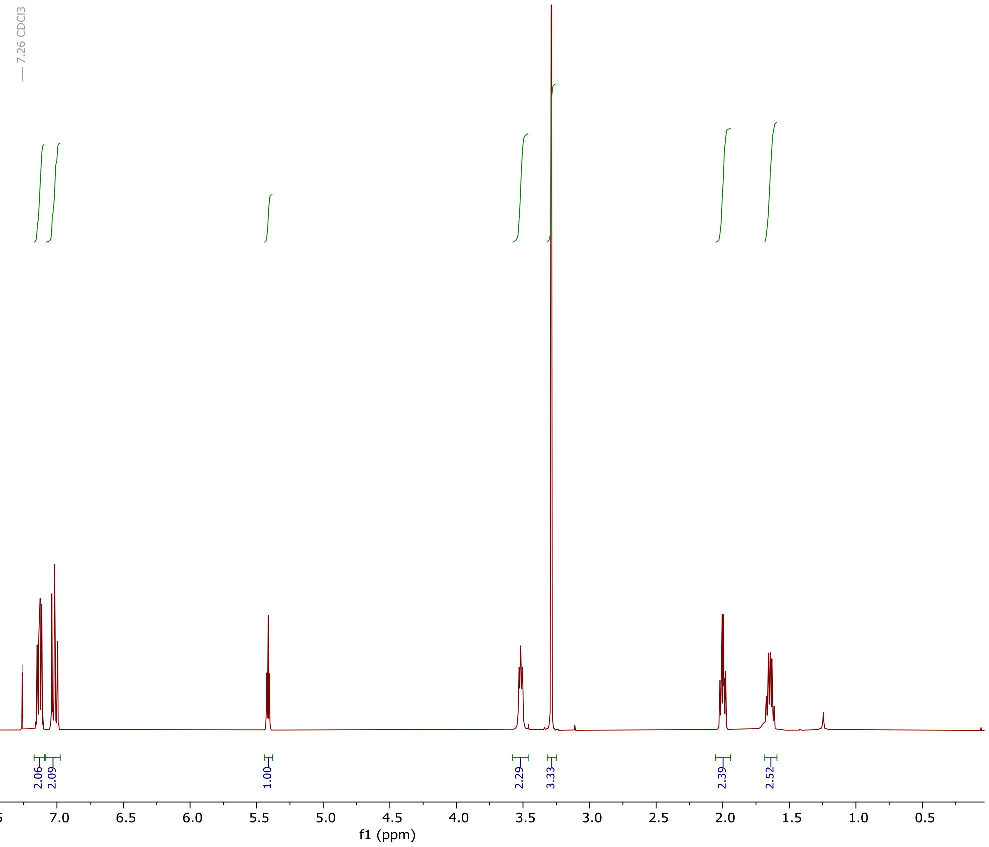

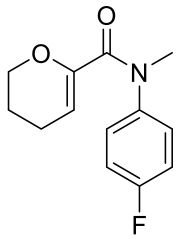

16a

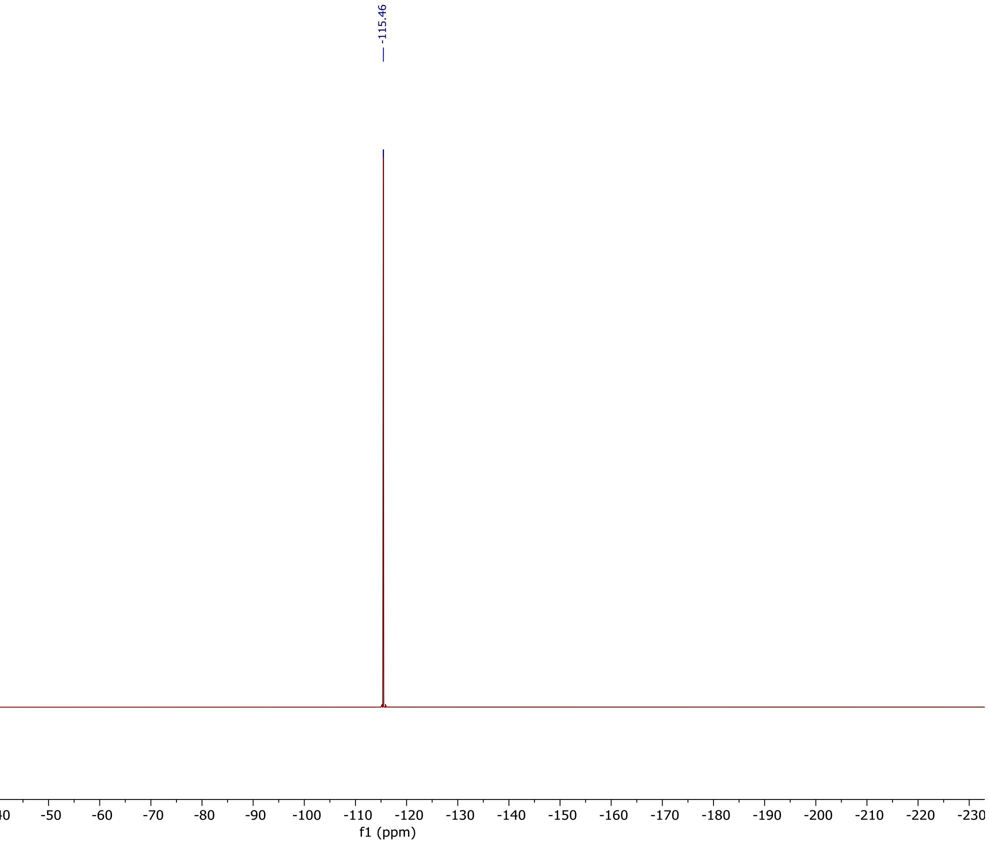

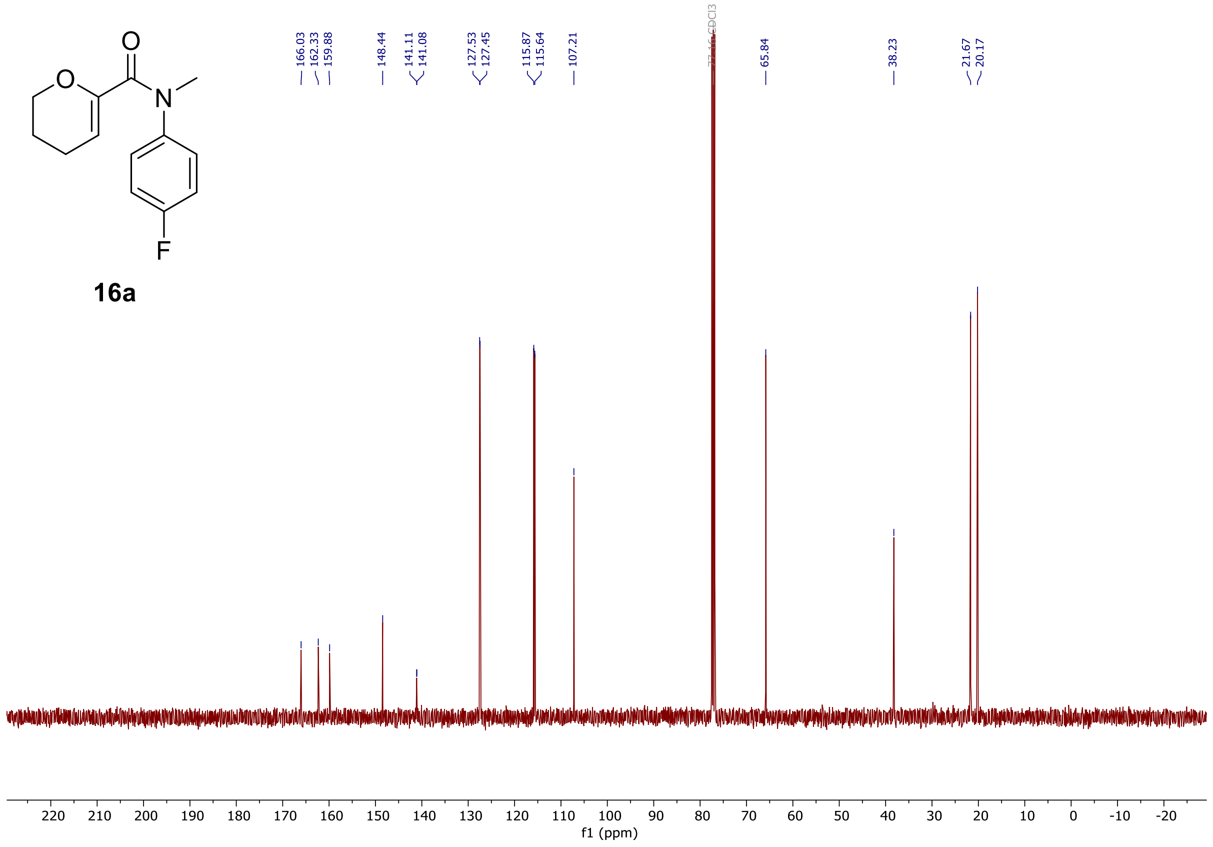

***N*-Benzyl-*N*-(4-chlorophenyl)-3,4-dihydro-2*H*-pyran-6-carboxamide (17a)**

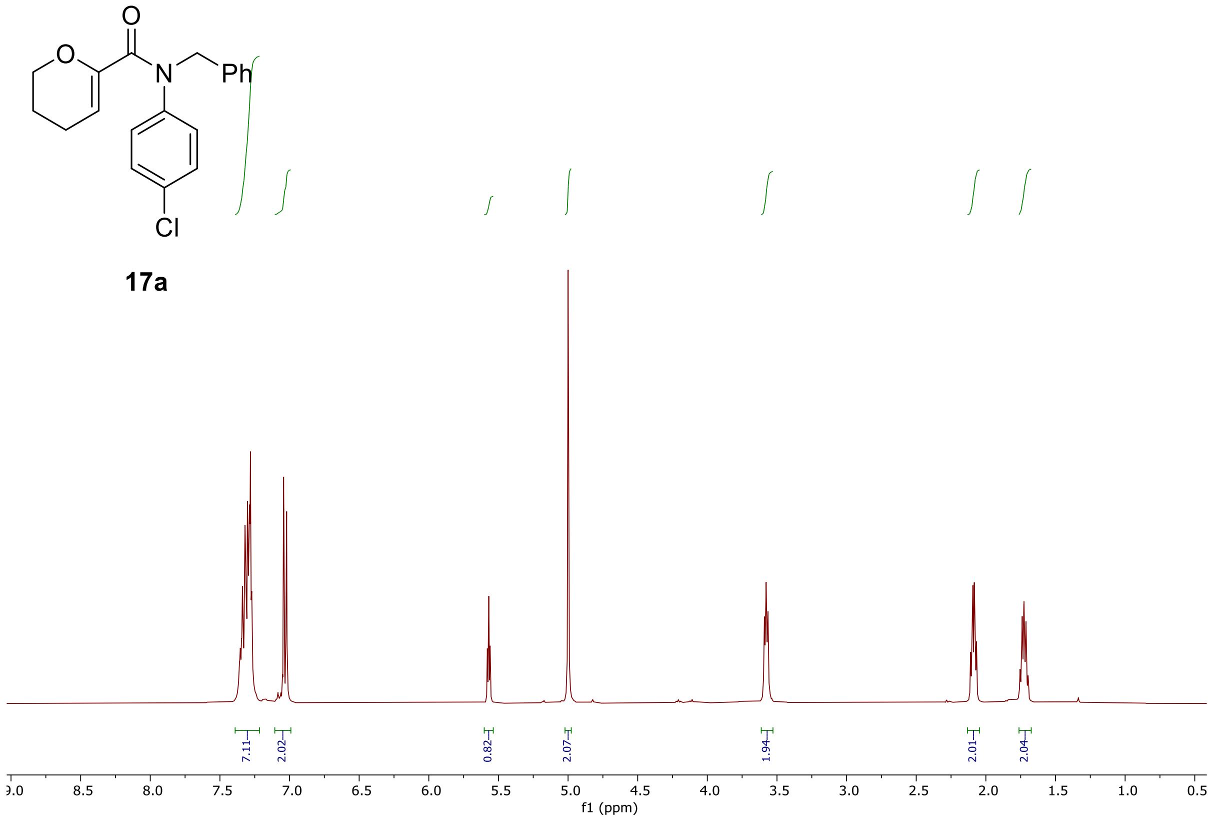

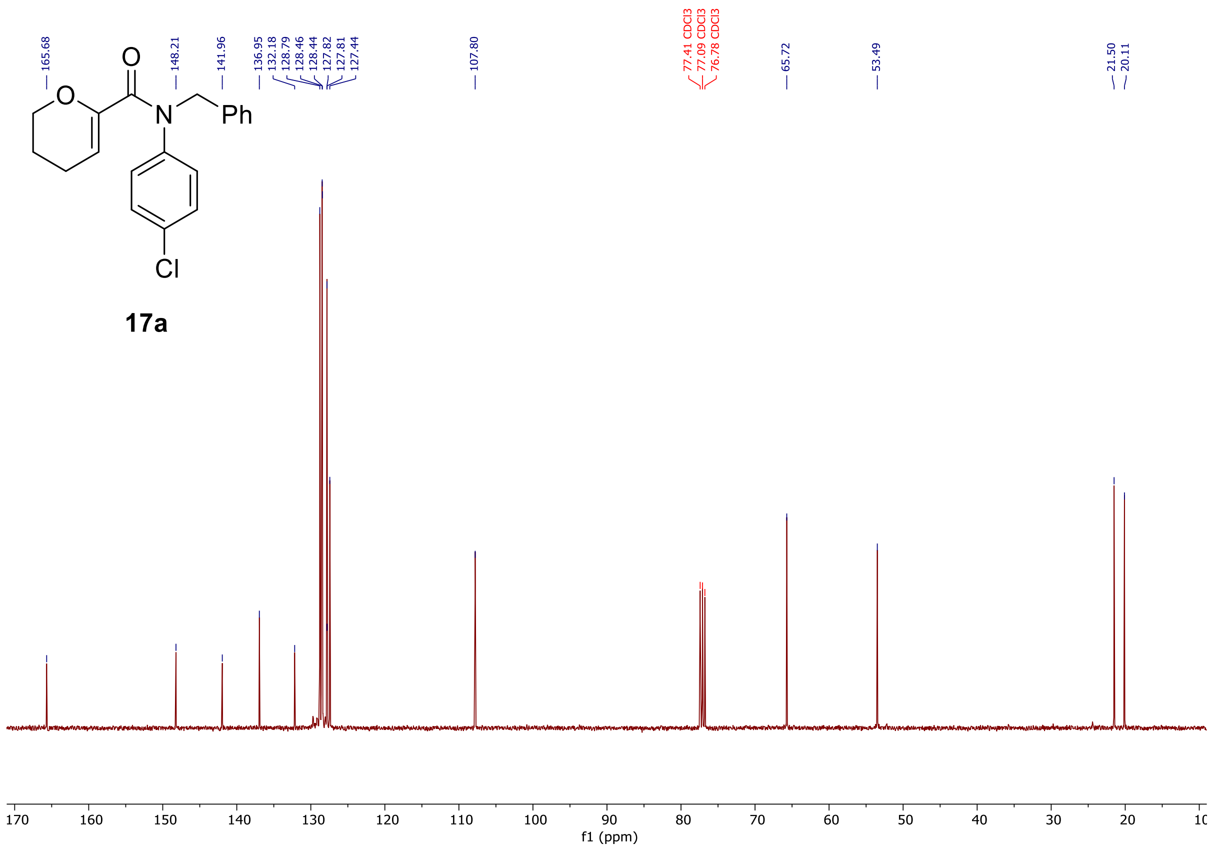

N-(4-Bromophenyl)-N-methyl-3,4-dihydro-2H-pyran-6-carboxamide (18a)

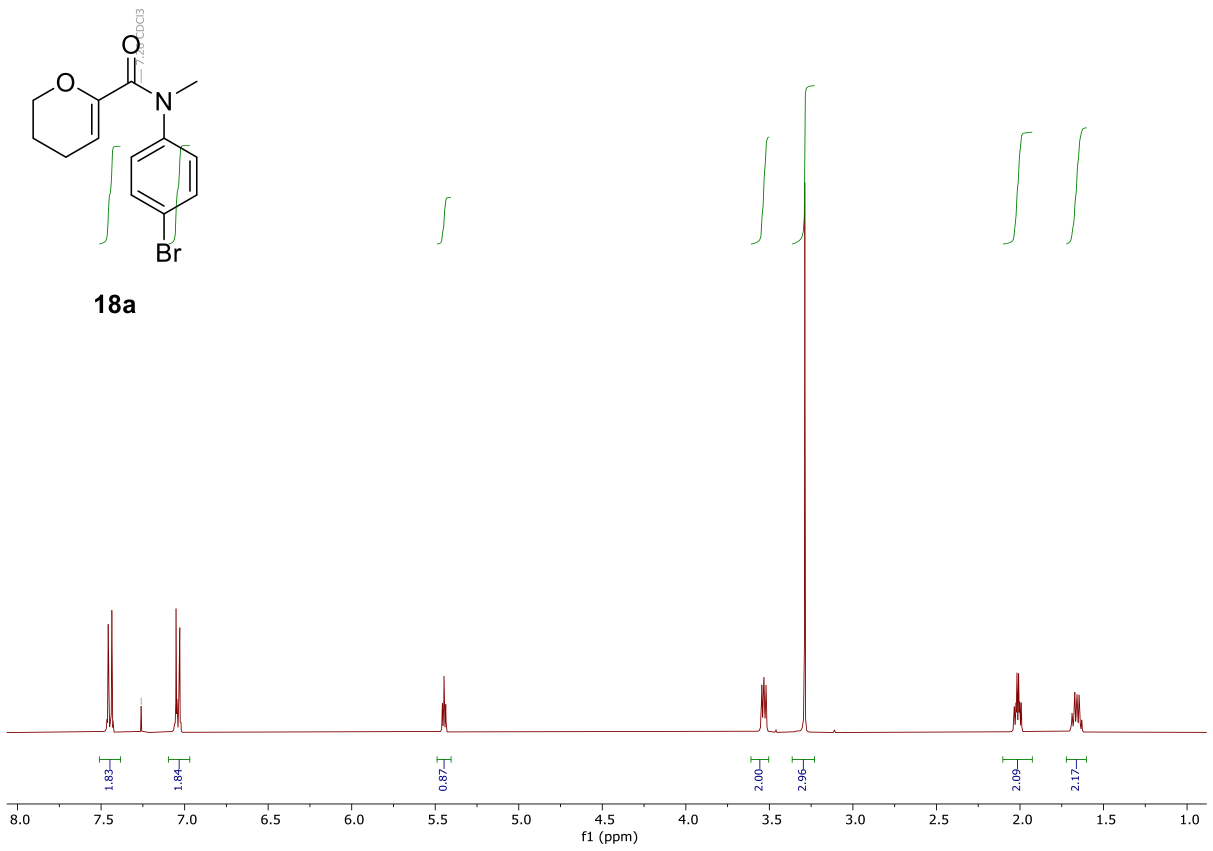

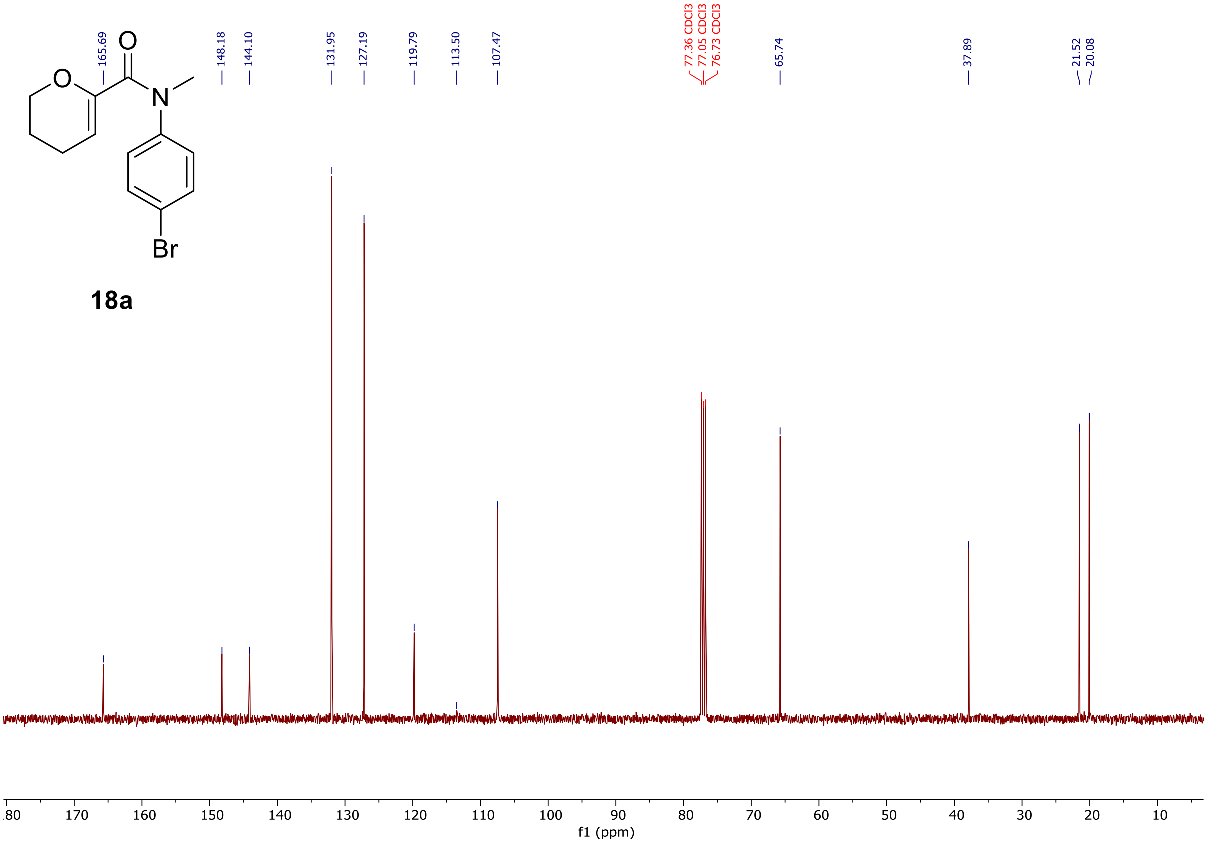

N-Methyl-*N*-(*p*-tolyl)-3,4-dihydro-2*H*-pyran-6-carboxamide (19a)

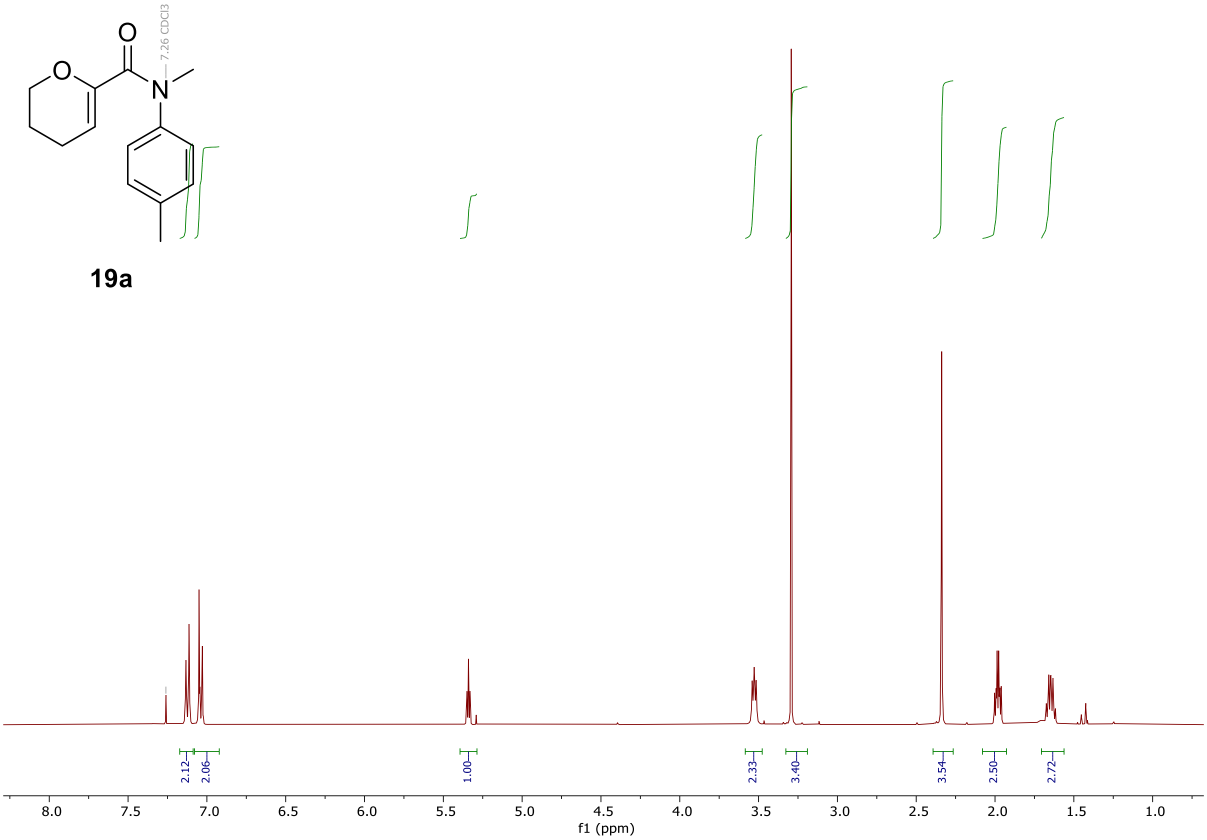

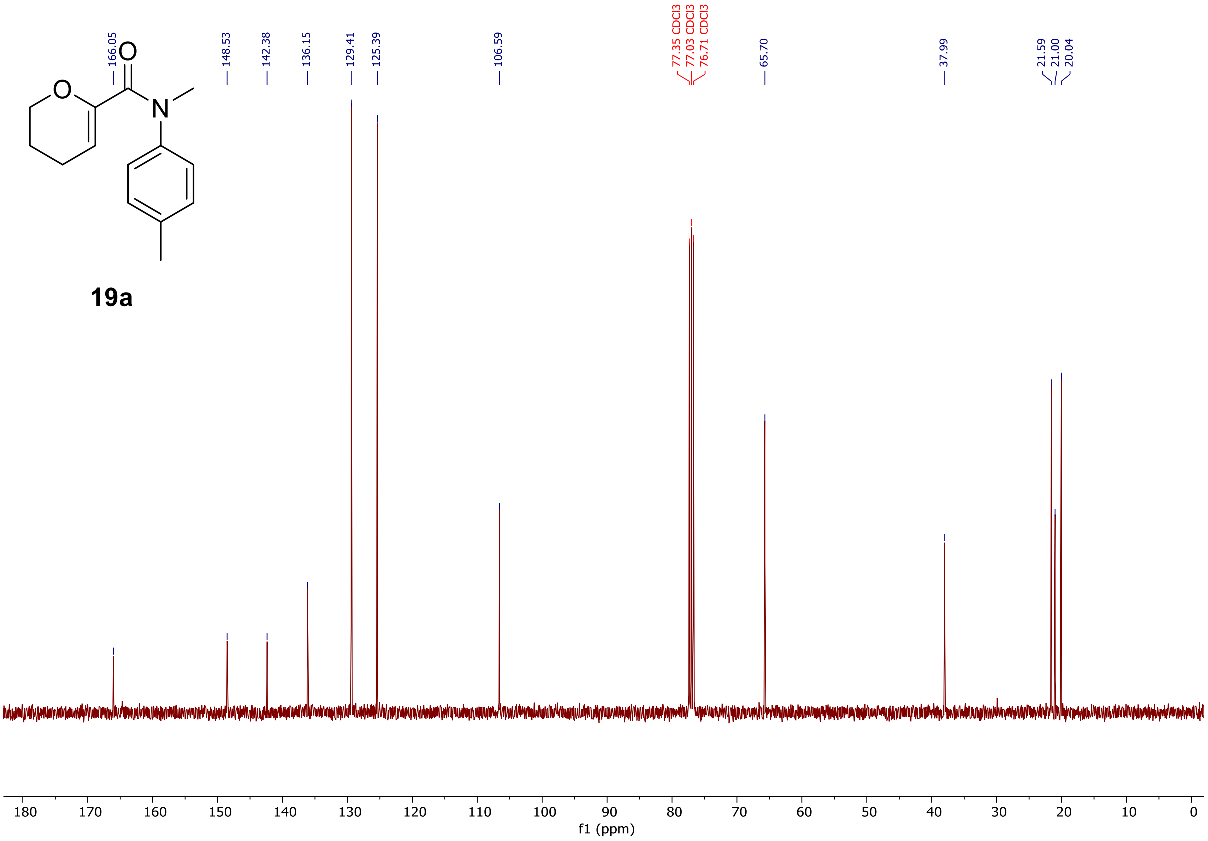

19a

N-Methyl-*N*-(4-(trifluoromethyl)phenyl)-3,4-dihydro-2*H*-pyran-6-carboxamide (20a)

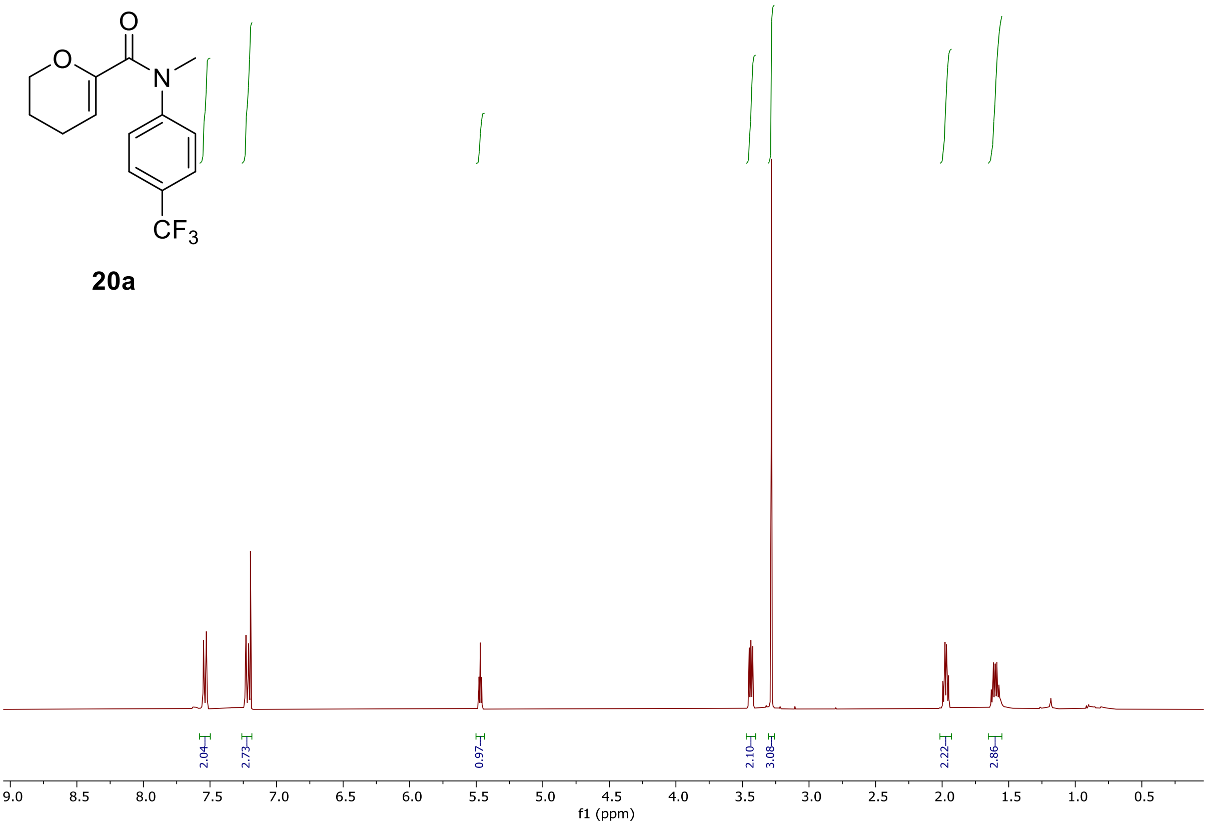

20a

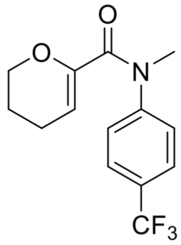

20a

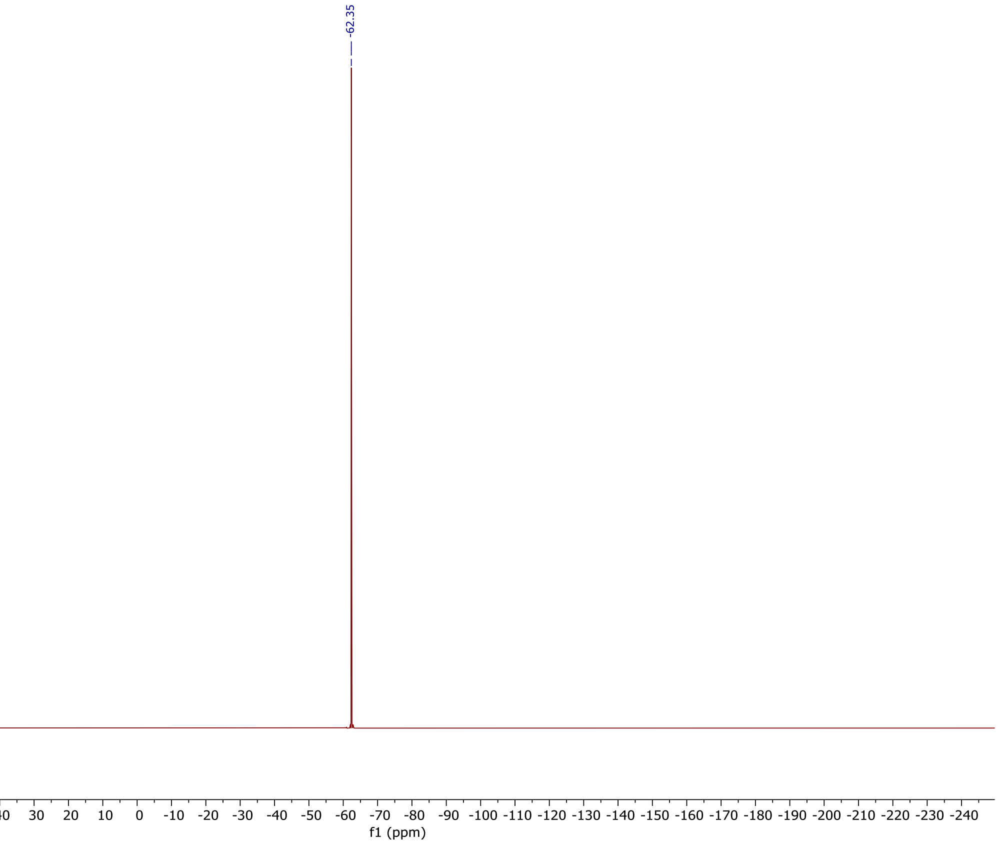

-62.35
90 80 70 60 50 40 30 20 10 0 -10 -20 -30 -40 -50 -60 -70 -80 -90 -100 -110 -120 -130 -140 -150 -160 -170 -180 -190 -200 -210 -220 -230 -240
f1 (ppm)

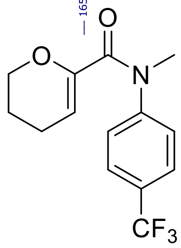

20a

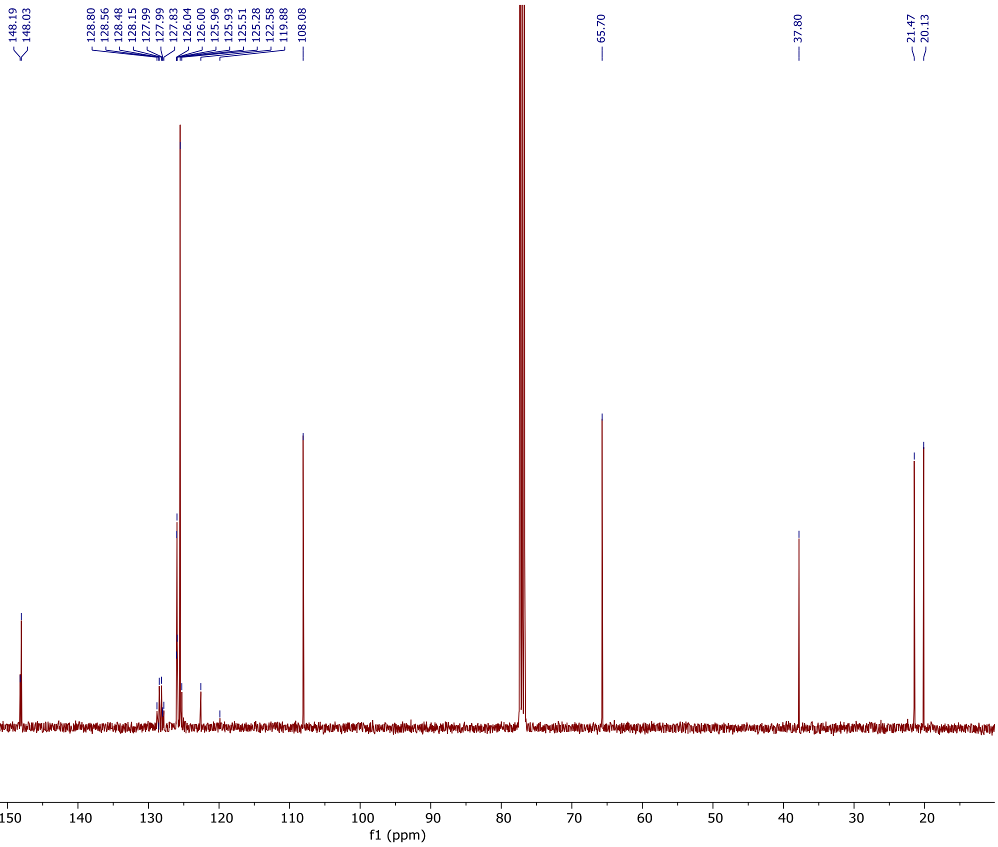

165.69
148.19
148.03
128.80
128.56
128.48
128.15
127.99
127.99
127.83
126.04
126.00
125.96
125.93
125.51
125.28
122.58
119.88
108.08
65.70
37.80
21.47
20.13
80 170 160 150 140 130 120 110 100 90 80 70 60 50 40 30 20
f1 (ppm)

_N_-(4-(_tert_-Butyl)phenyl)-_N_-methyl-3,4-dihydro-2_H_-pyran-6-carboxamide (21a)

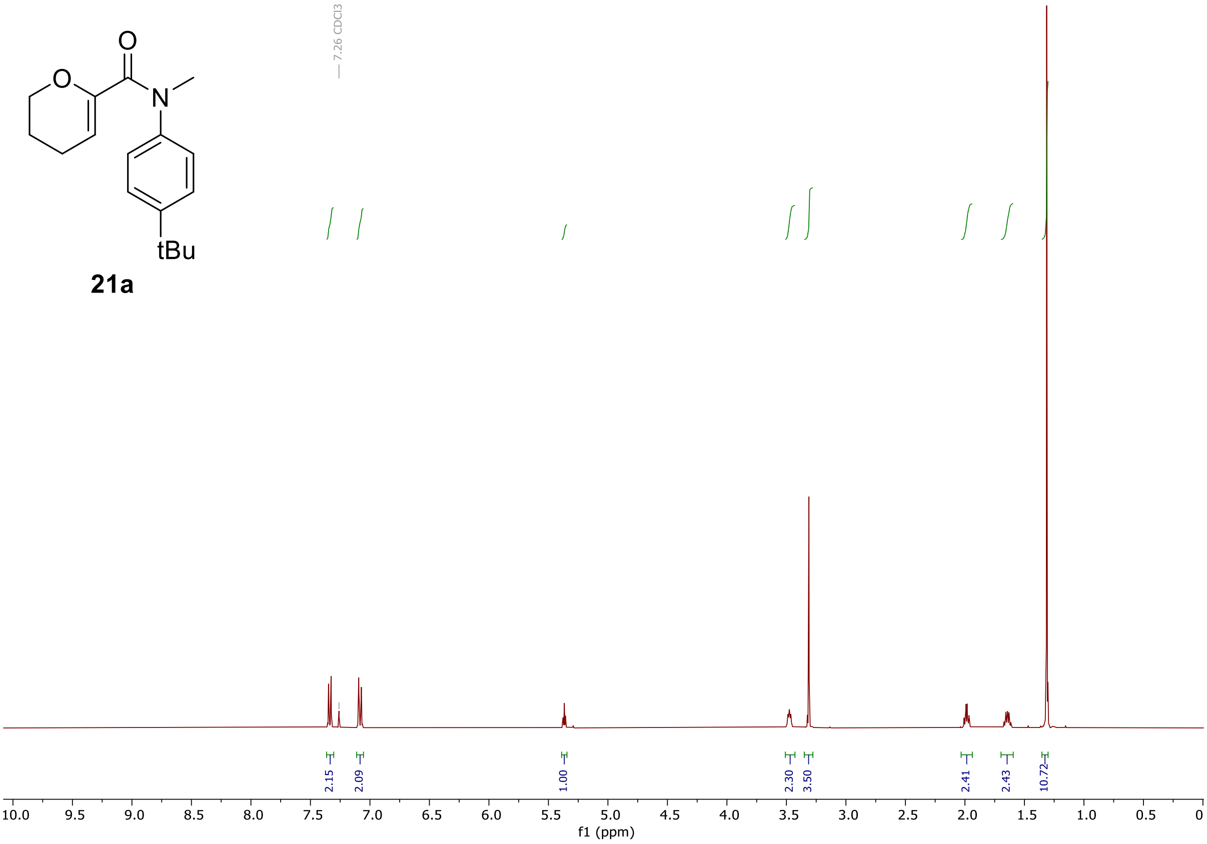

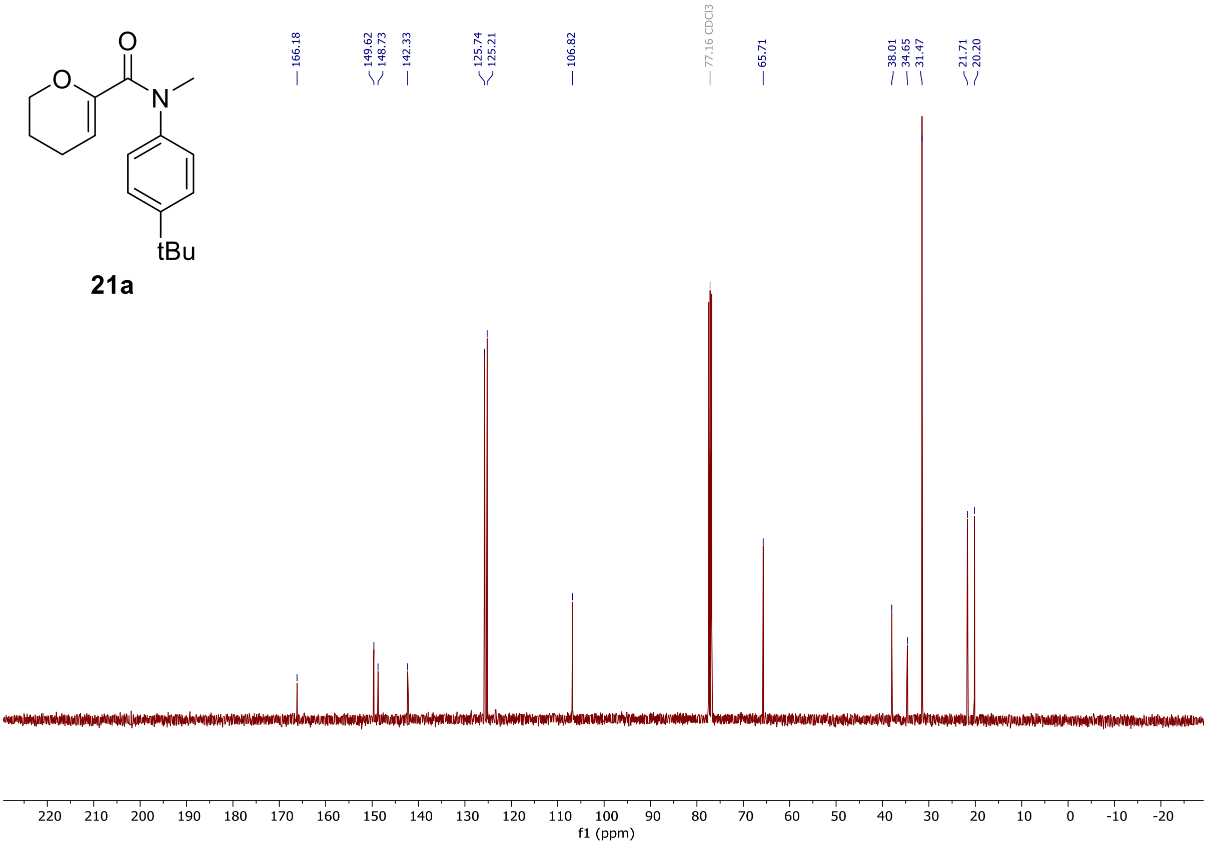

N-(4-methoxyphenyl)-N-methyl-3,4-dihydro-2H-pyran-6-carboxamide (22a)

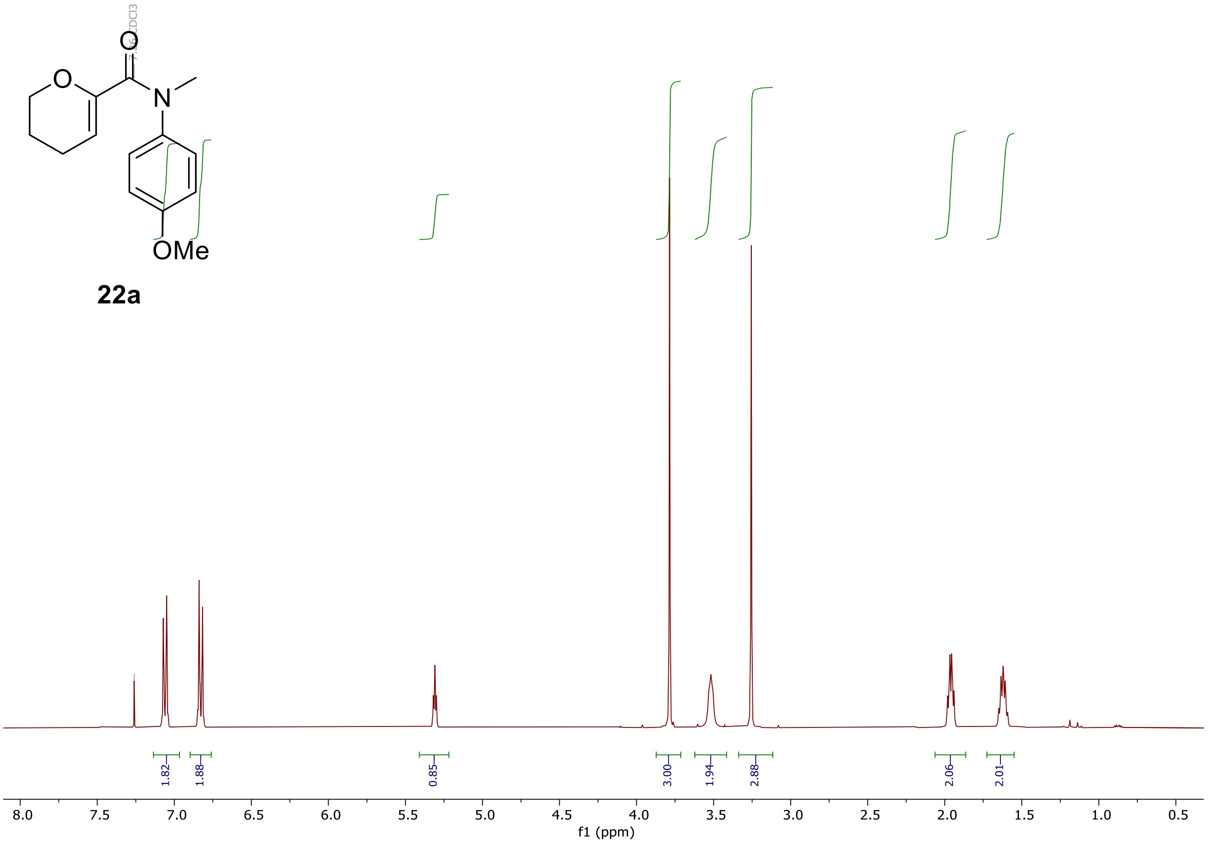

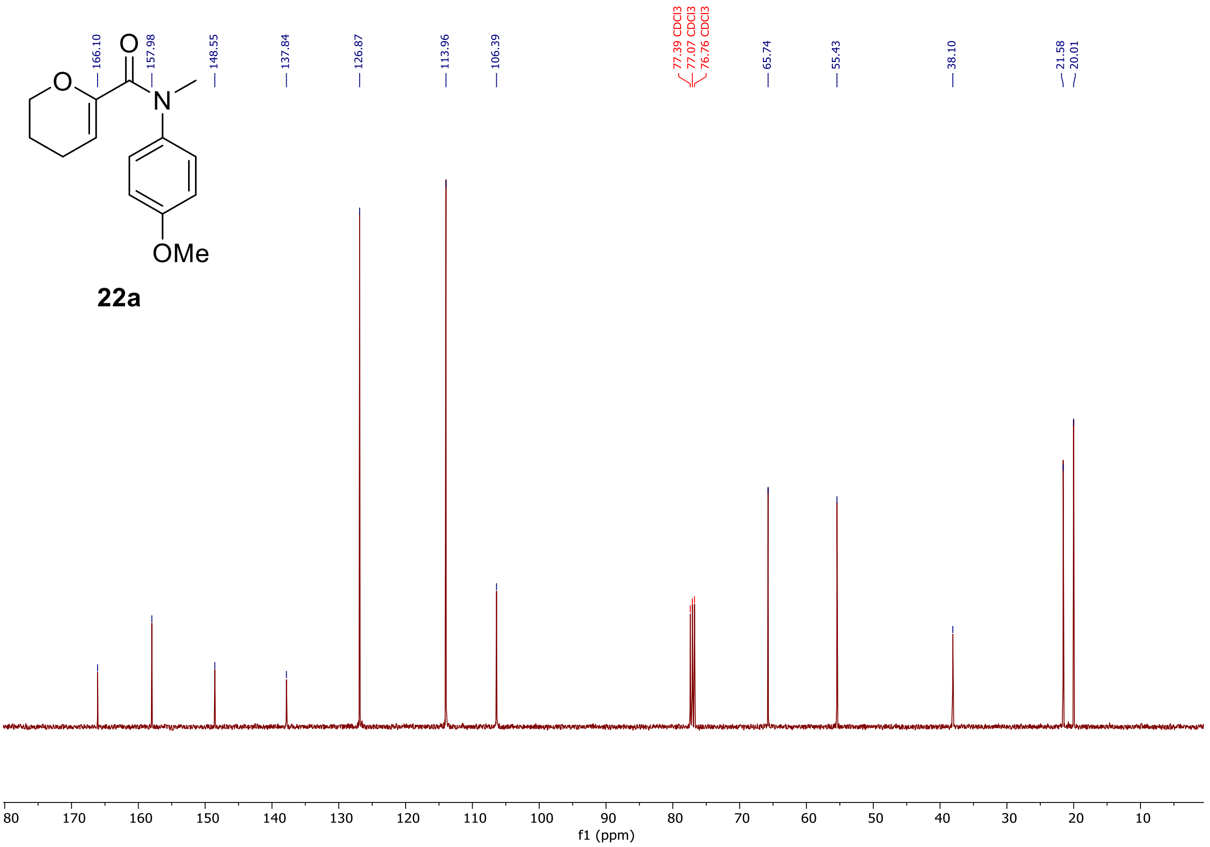

N-Methyl-N-(4-(methylthio)phenyl)-3,4-dihydro-2H-pyran-6-carboxamide (23a)

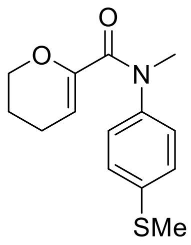

23a

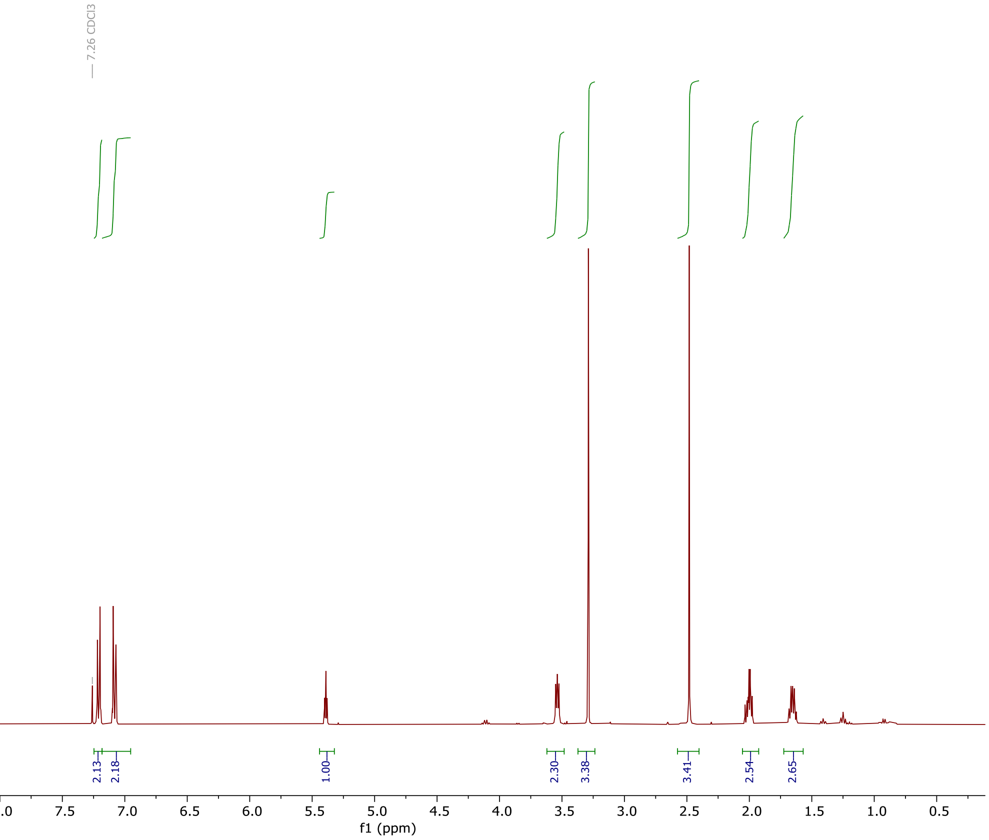

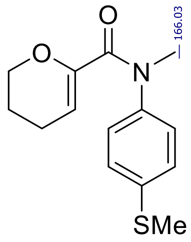

23a

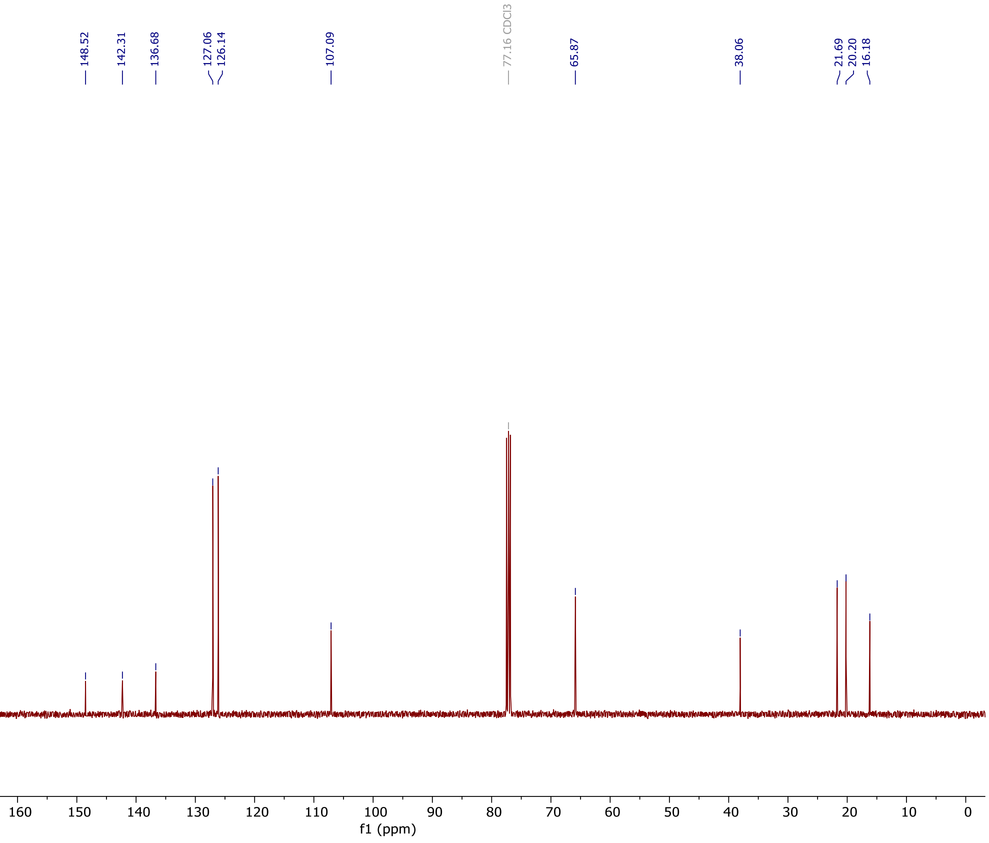

***N*-Methyl-*N*-phenyl-4,5-dihydrofuran-2-carboxamide (24a)**

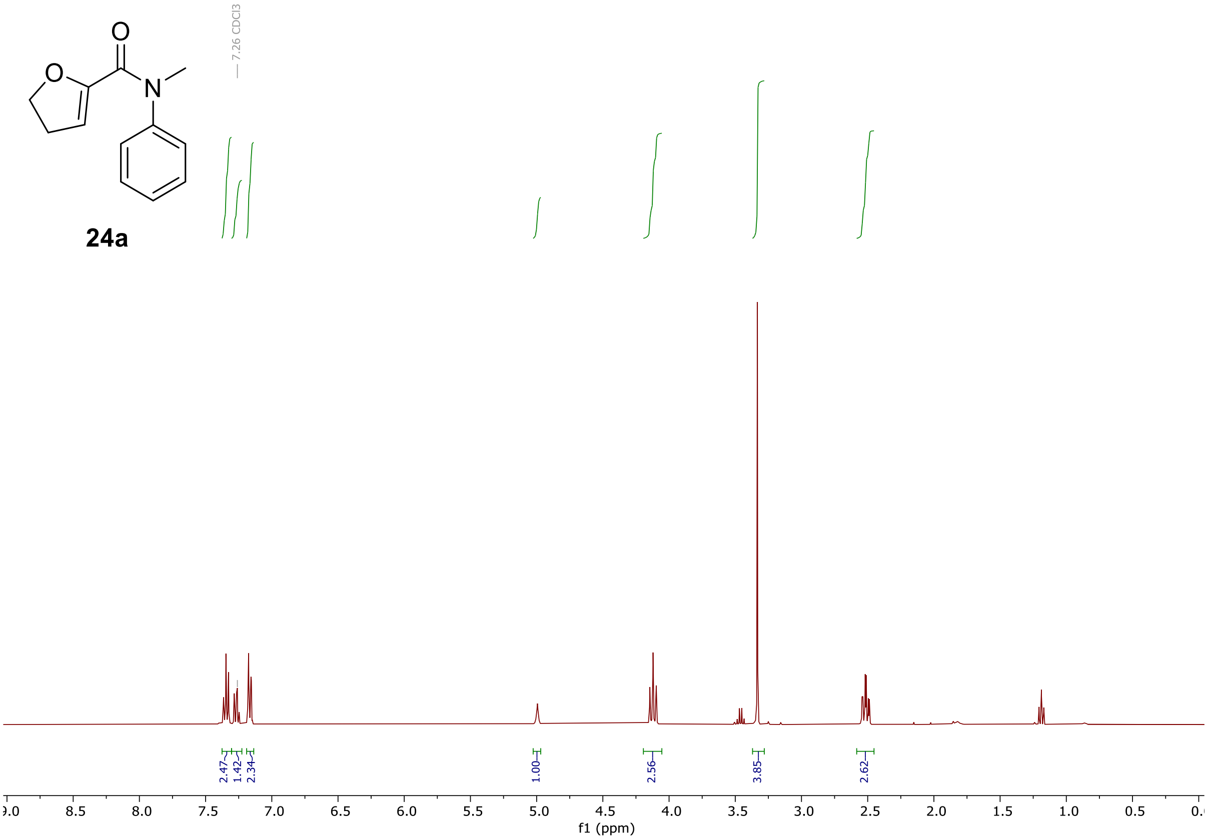

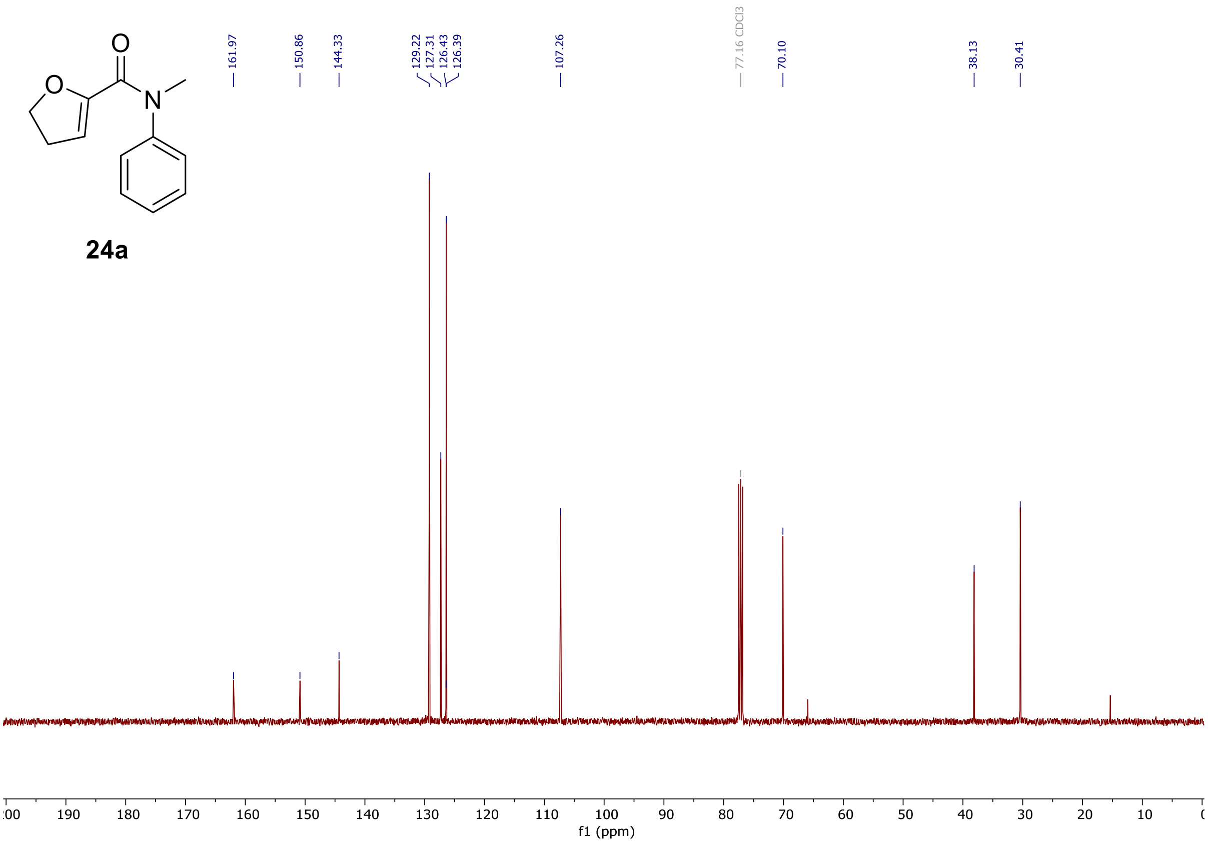

N-Methyl-*N*-(phenyl-d_5)-3,4-dihydro-2*H*-pyran-6-carboxamide (28)

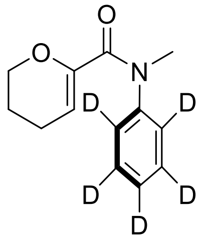

28

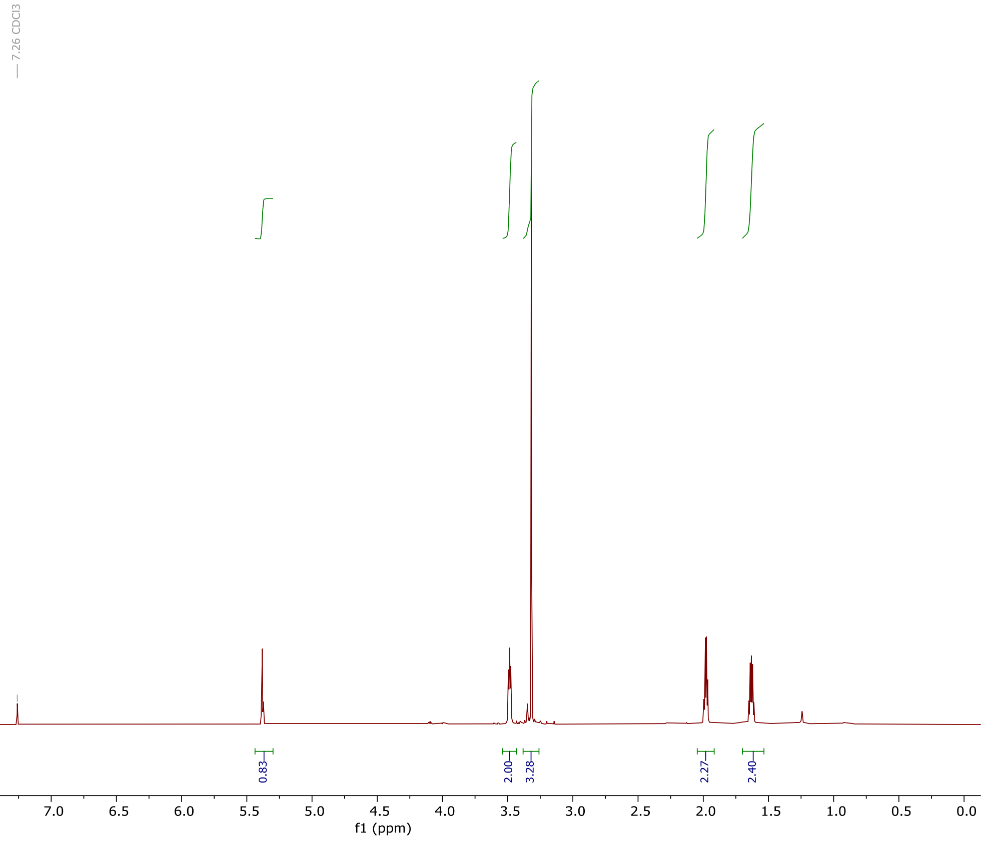

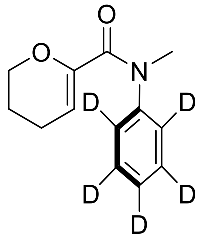

28

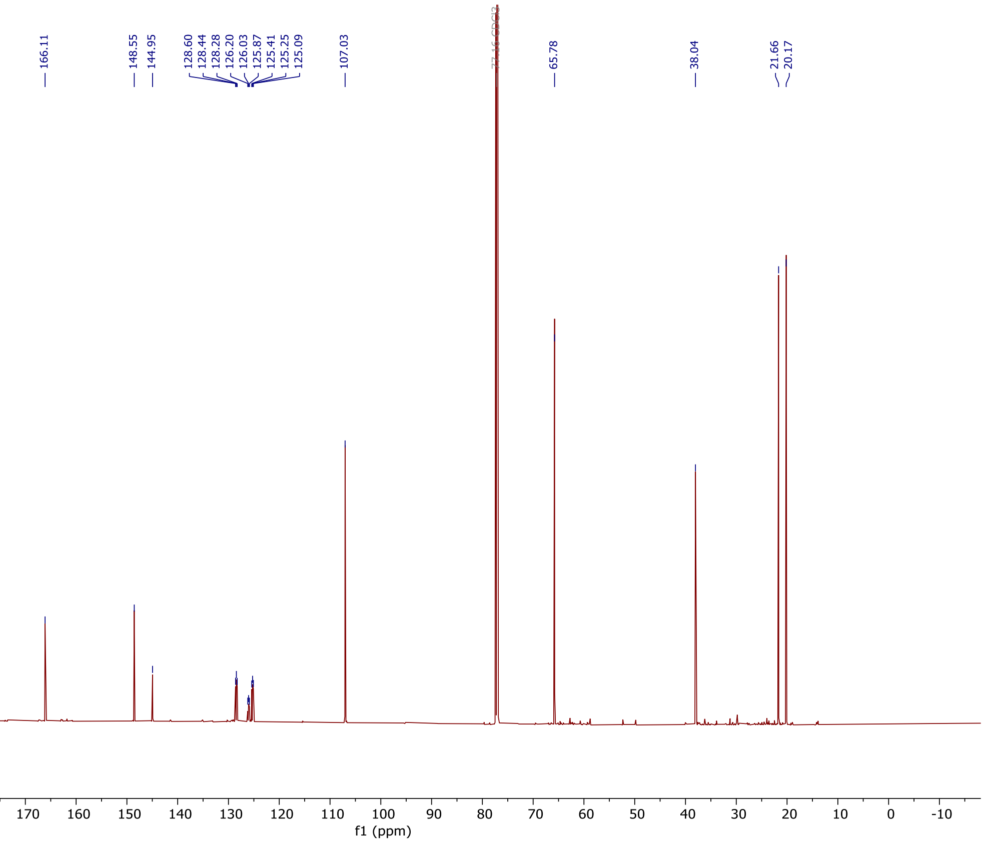

N-Ethyl-*N*-phenyl-3,4-dihydro-2*H*-pyran-6-carboxamide (S1a)

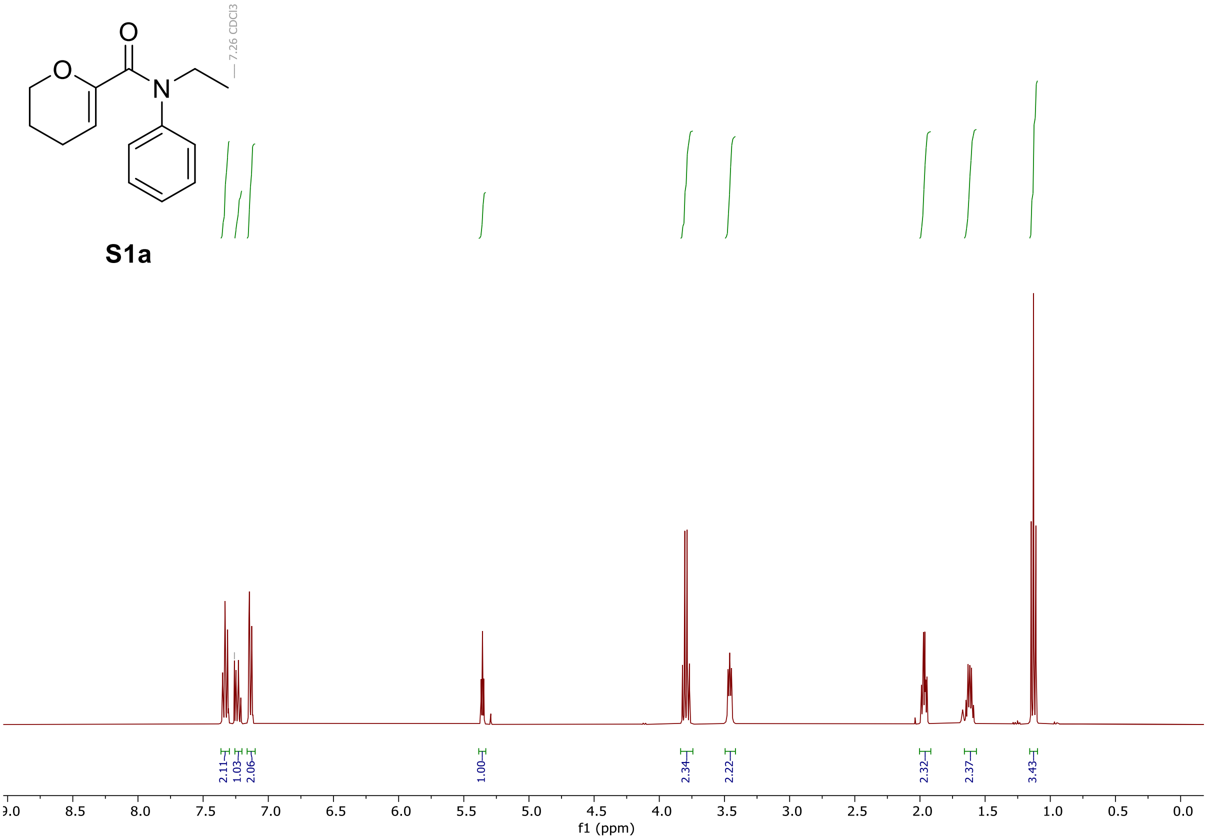

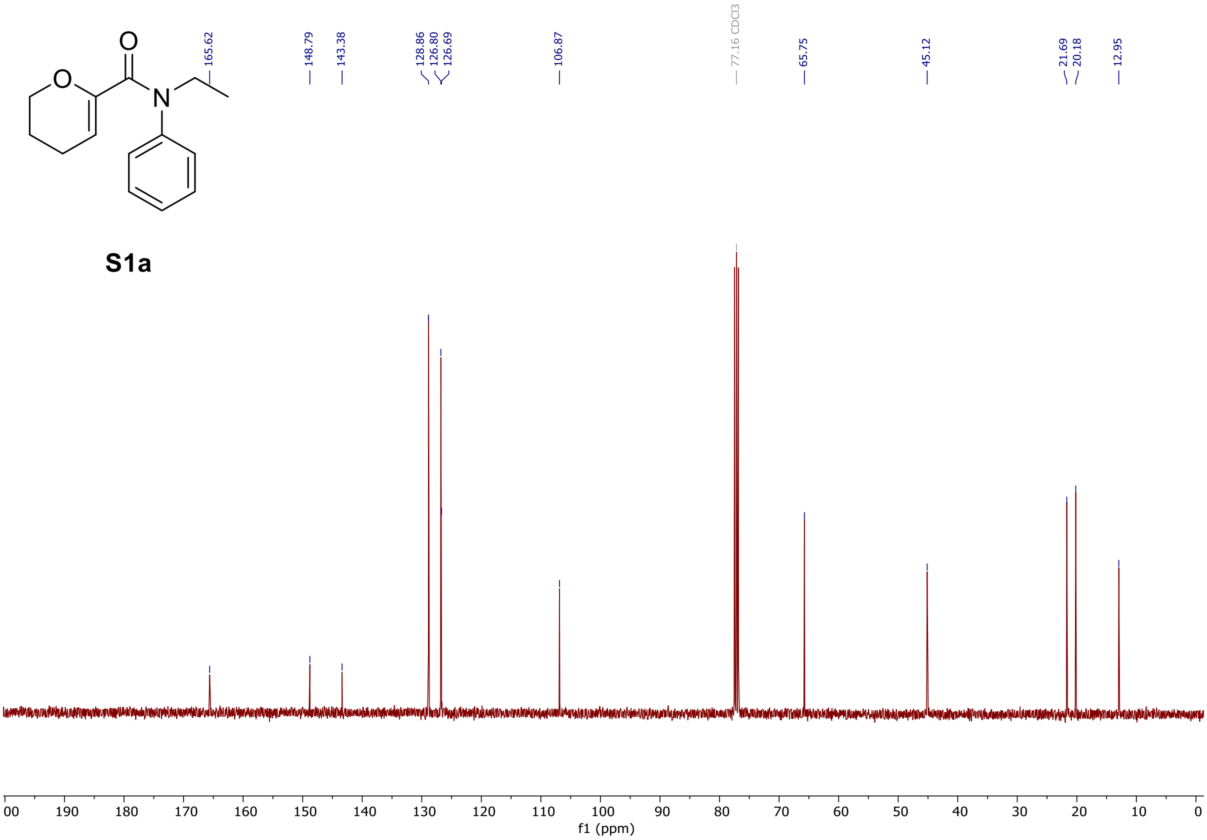

***N*-(4-Chlorophenyl)-*N*-methyl-3,4-dihydro-2*H*-pyran-6-carboxamide (S2a)**

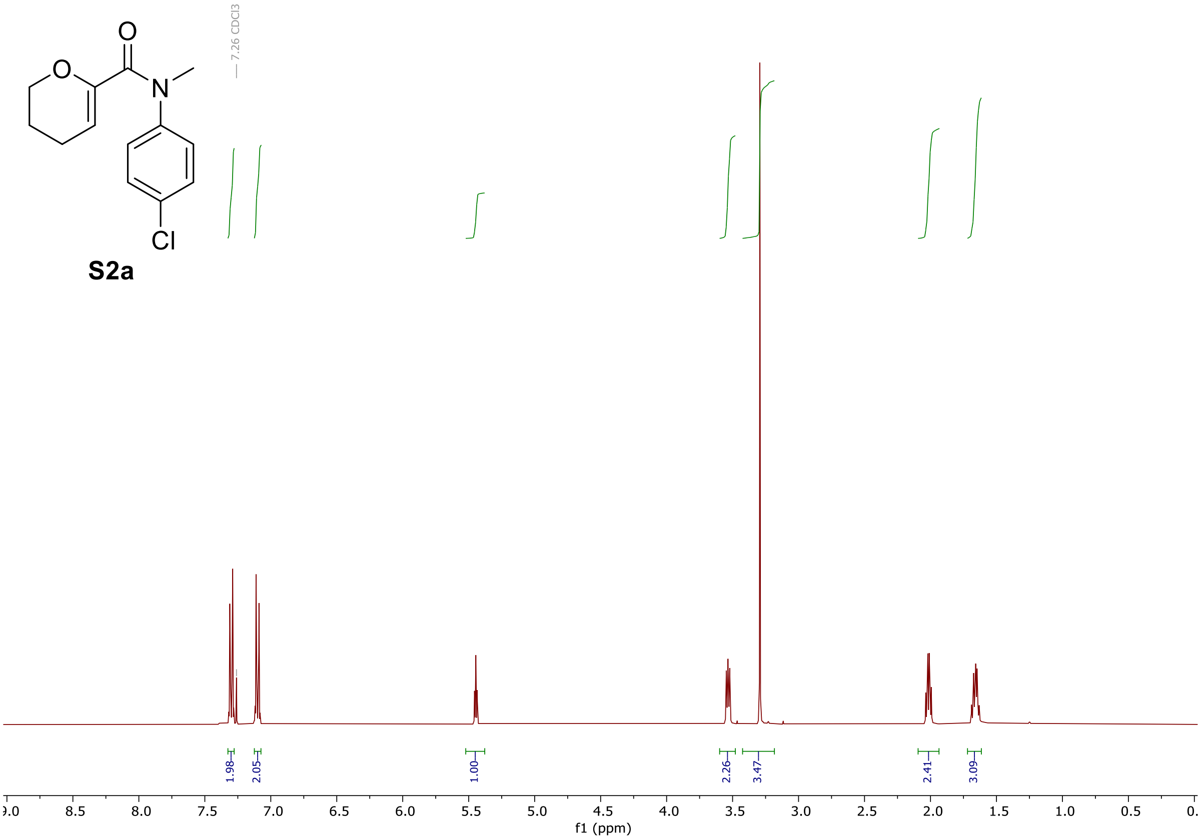

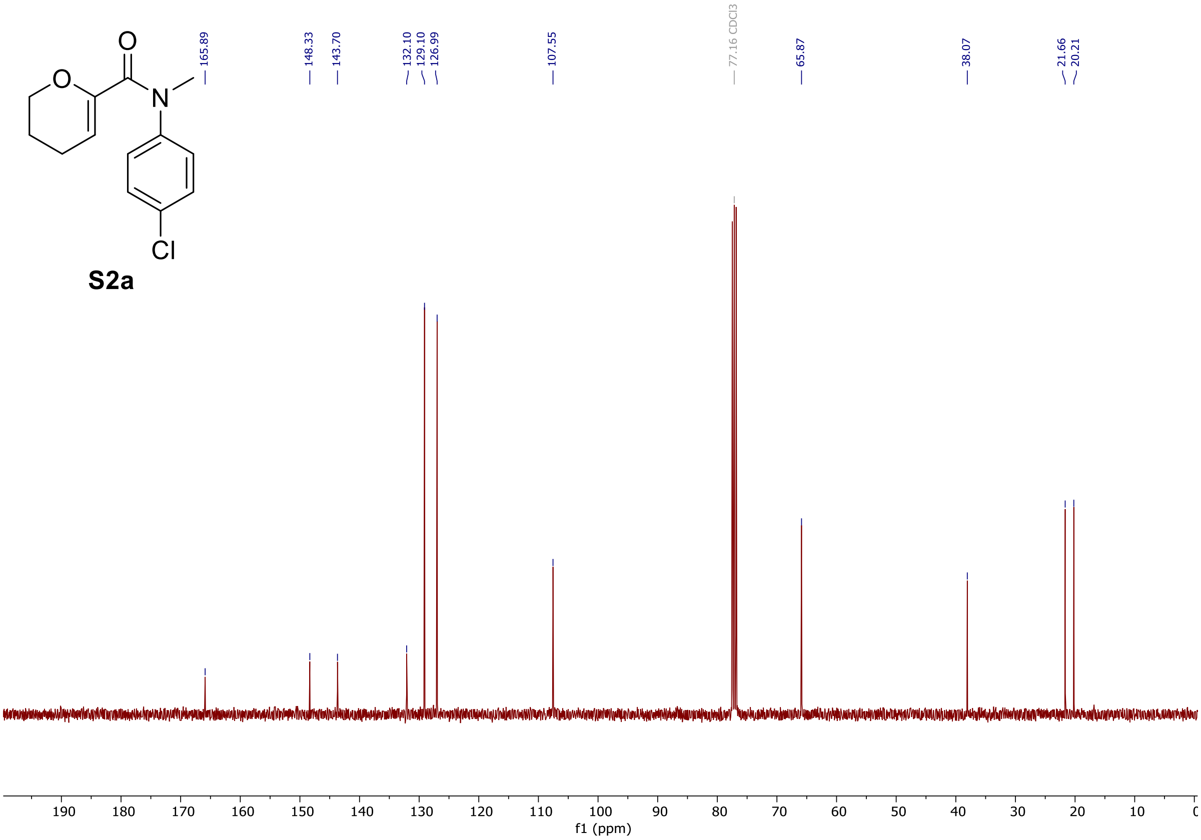

_N_-Methyl-_N_-phenyl-4,5,6,7-tetrahydrooxepine-2-carboxamide (S3a)

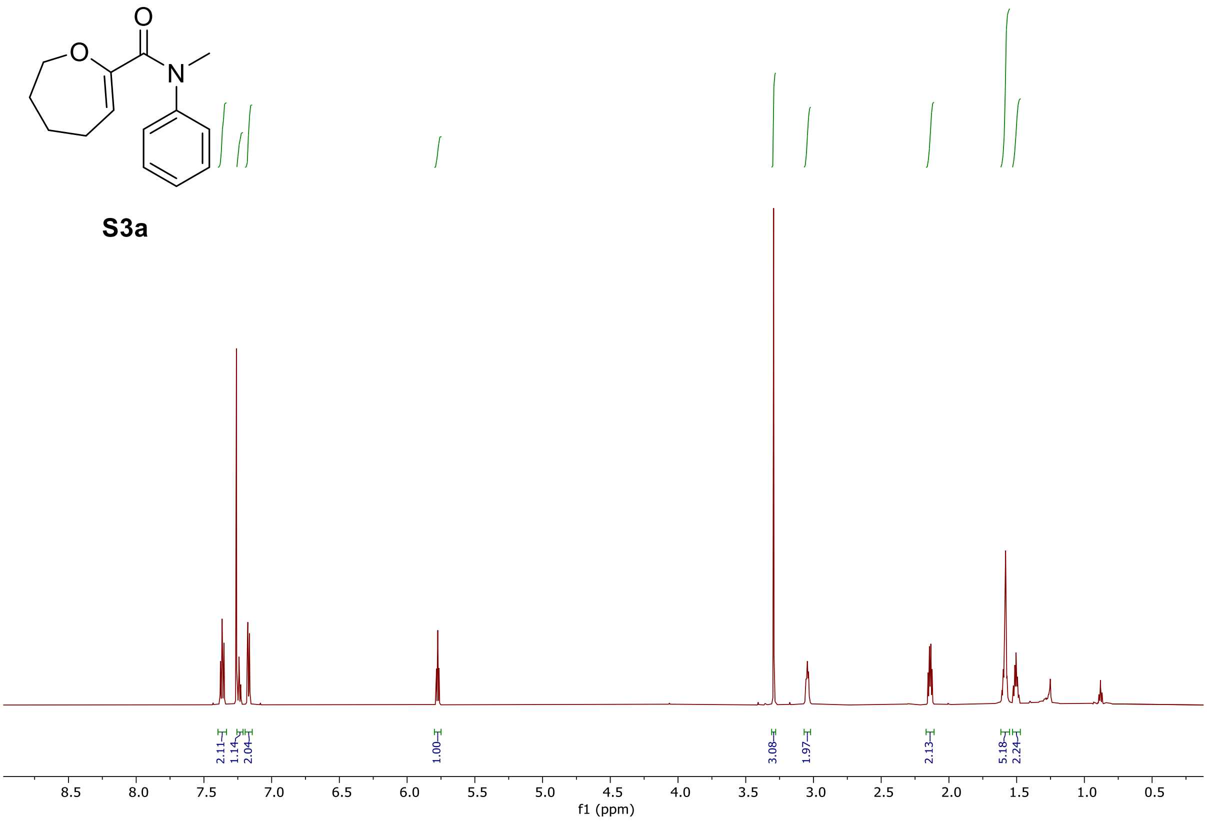

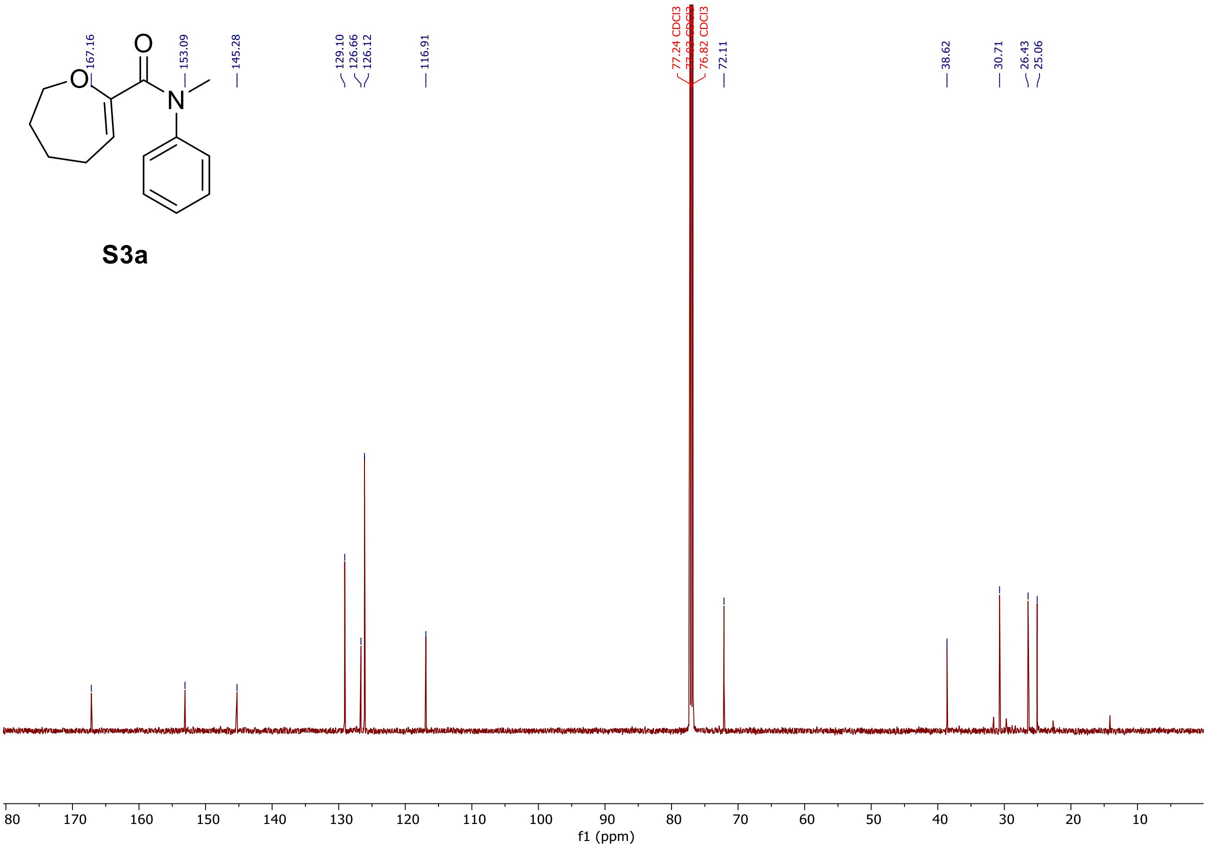

9.4. Photocyclization Products

(4a*R*,10b*S*)-6-Methyl-2,3,6,10b-tetrahydro-1*H*-pyrano[2,3-*c*]quinolin-5(4a*H*)-one (2)

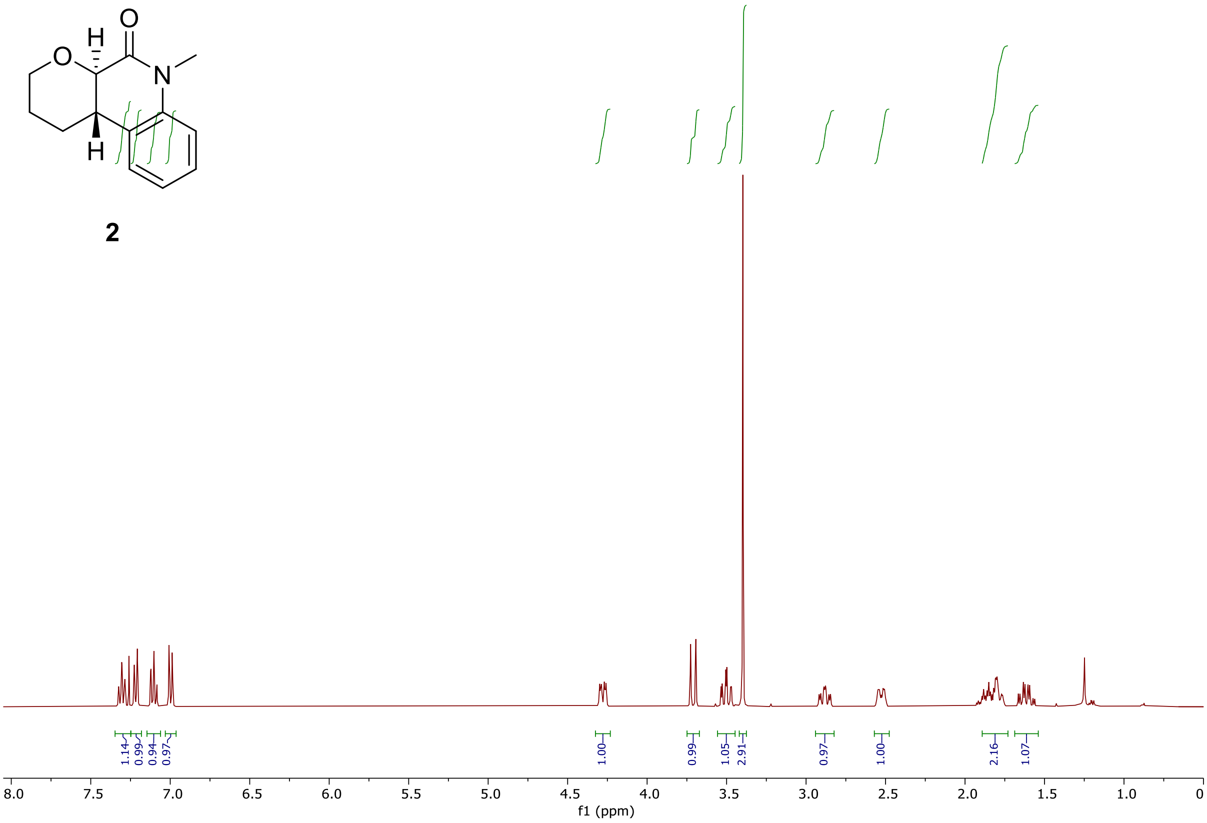

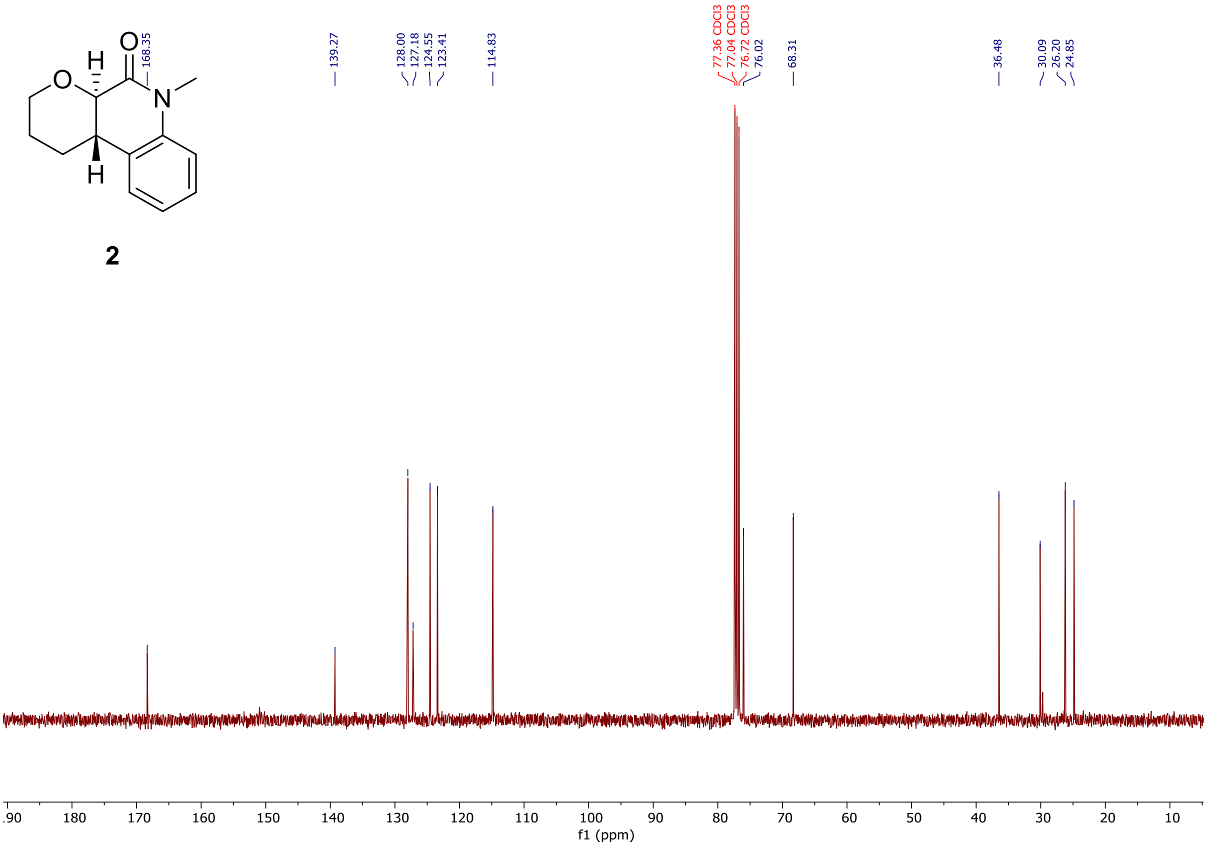

(4a*R*,10b*S*)-6-Benzyl-2,3,6,10b-tetrahydro-1*H*-pyrano[2,3-*c*]quinolin-5(4a*H*)-one (5)

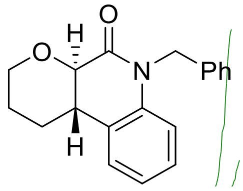

5

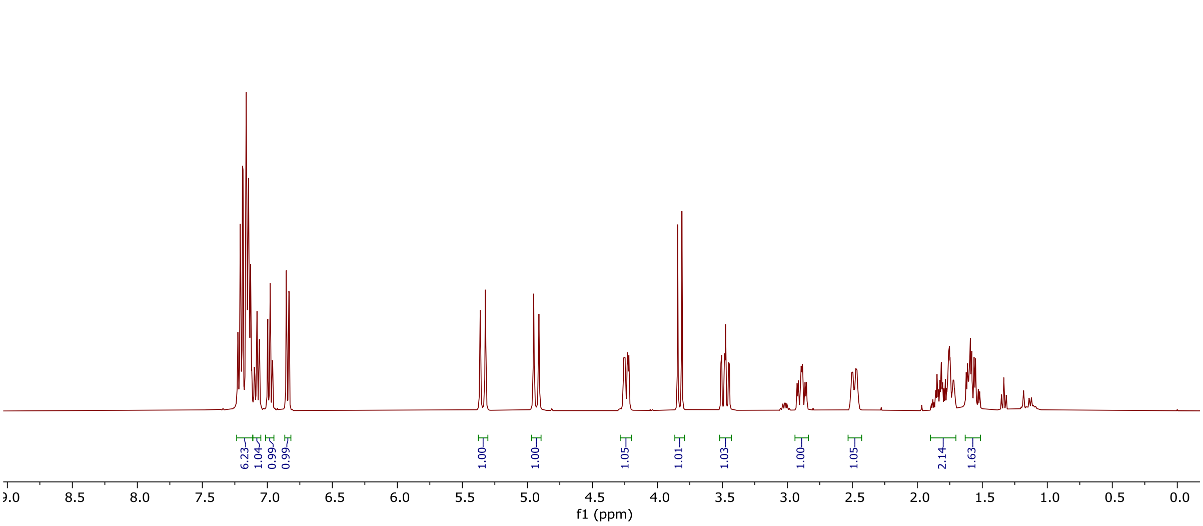

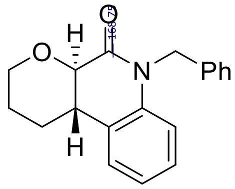

5

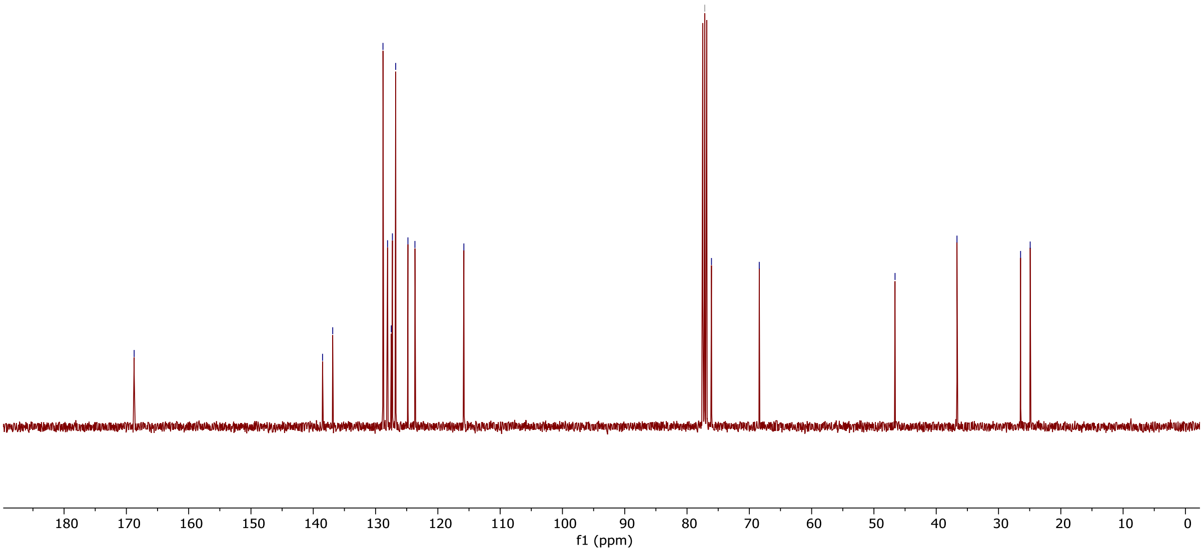

(4a*R*,10b*S*)-6-Isobutyl-2,3,6,10b-tetrahydro-1*H*-pyrano[2,3-*c*]quinolin-5(4aH)-one (6)

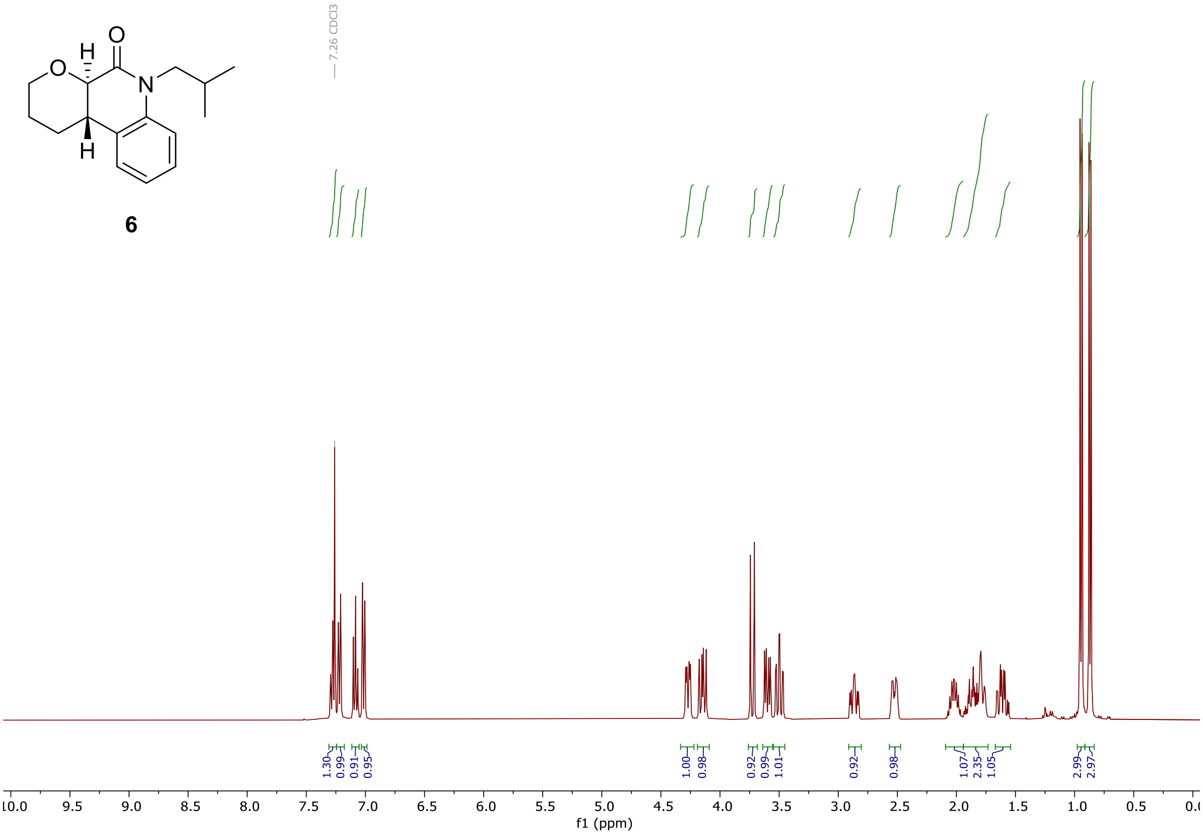

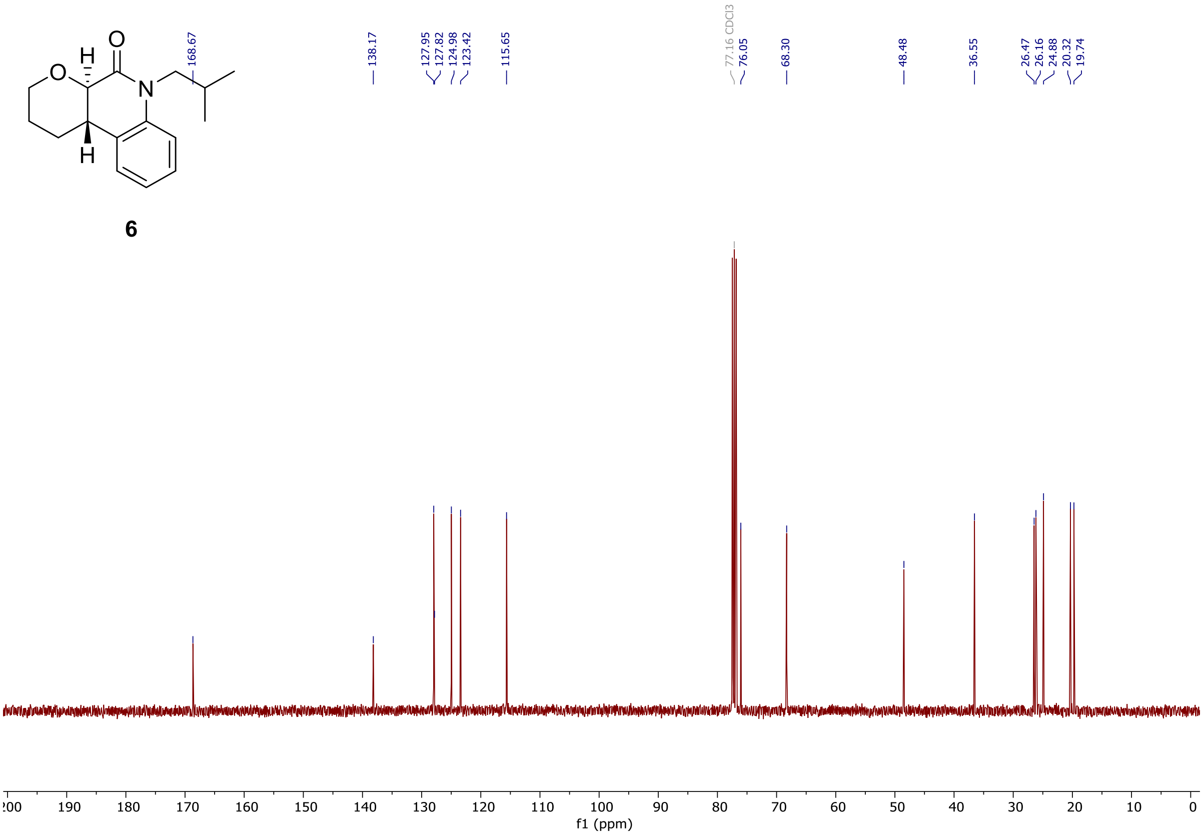

(4a*R*,10b*S*)-6-Propyl-2,3,6,10b-tetrahydro-1*H*-pyrano[2,3-*c*]quinolin-5(4a*H*)-one (7)

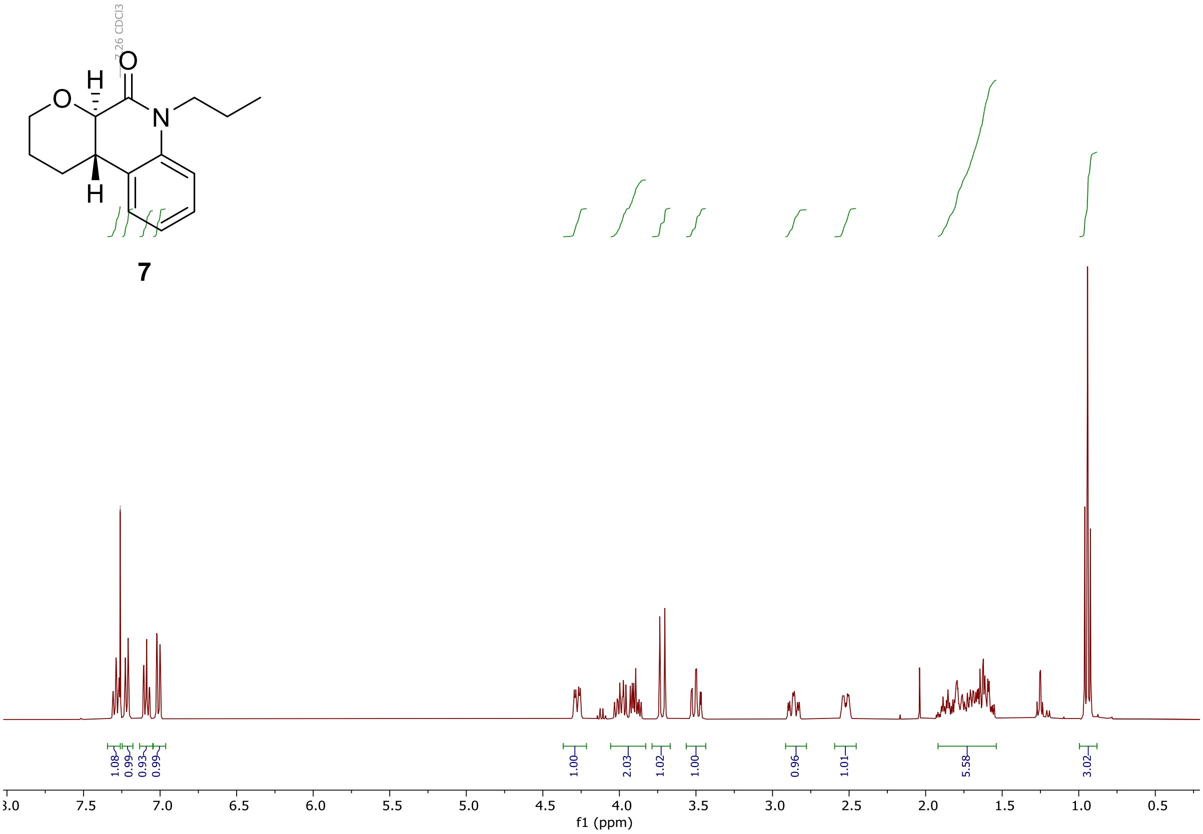

7

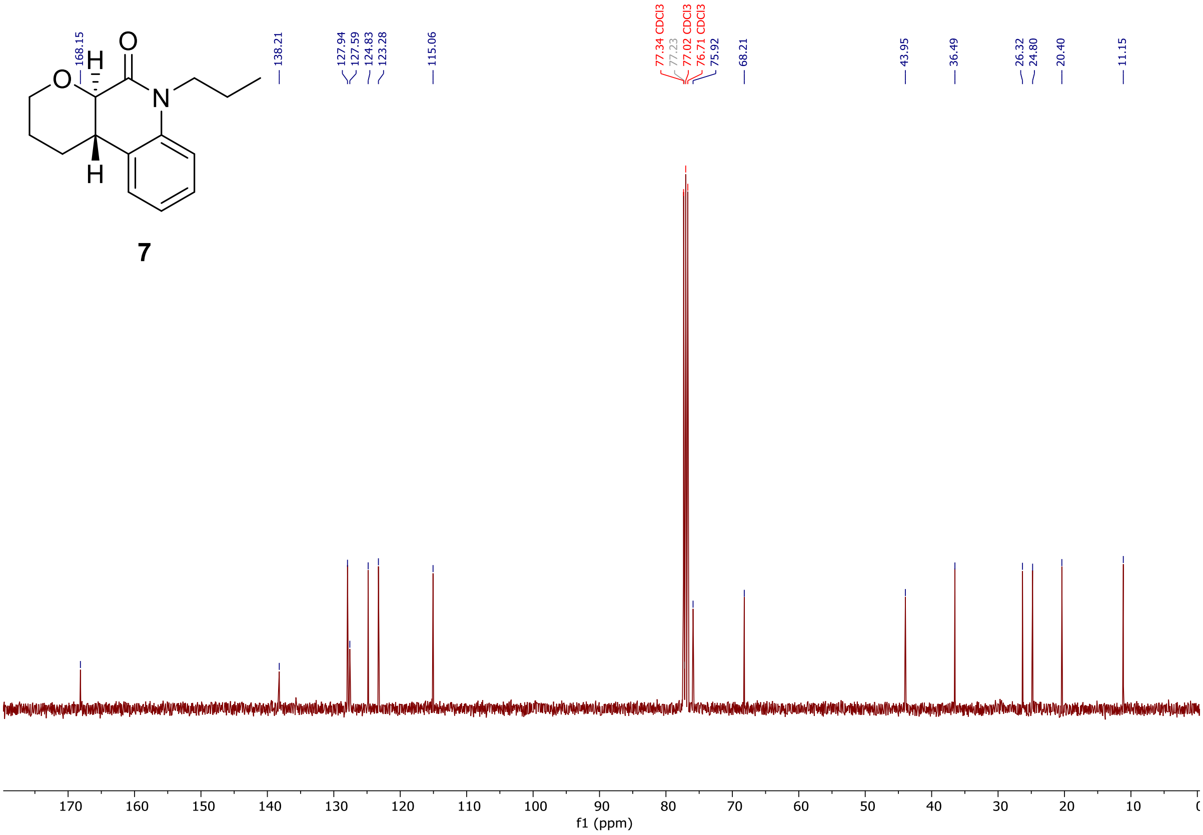

7

(4a*R*,10b*S*)-6-Allyl-2,3,6,10b-tetrahydro-1*H*-pyrano[2,3-*c*]quinolin-5(4a*H*)-one (8)

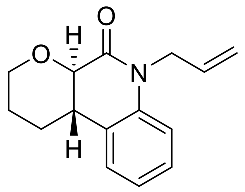

8

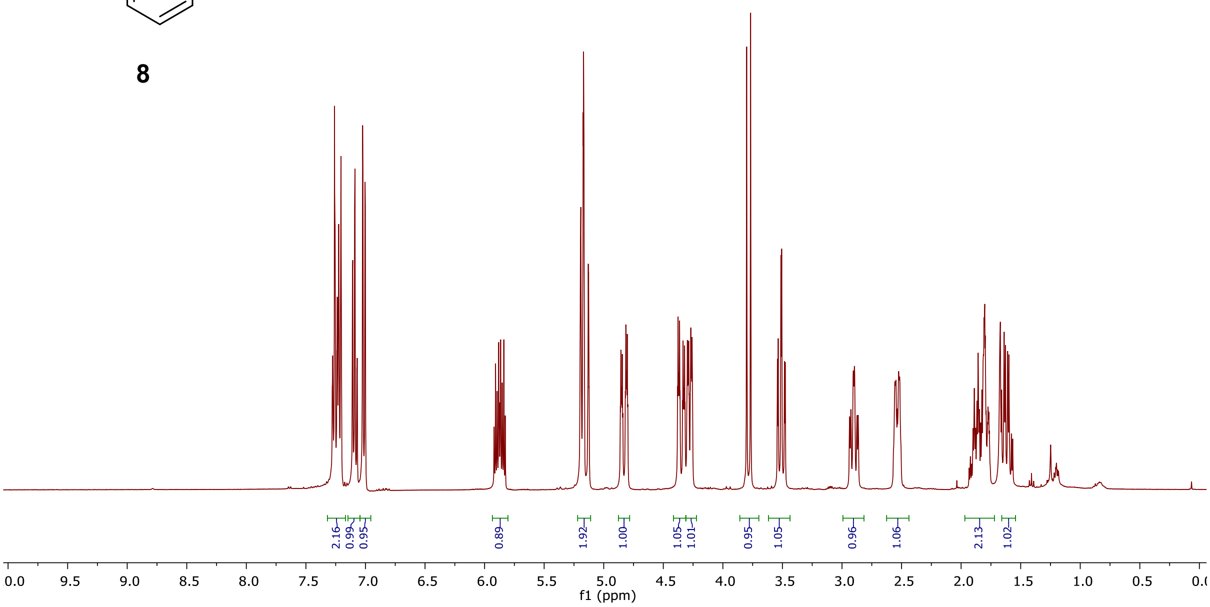

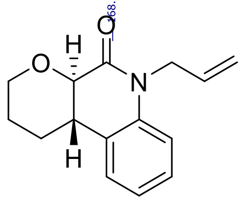

8

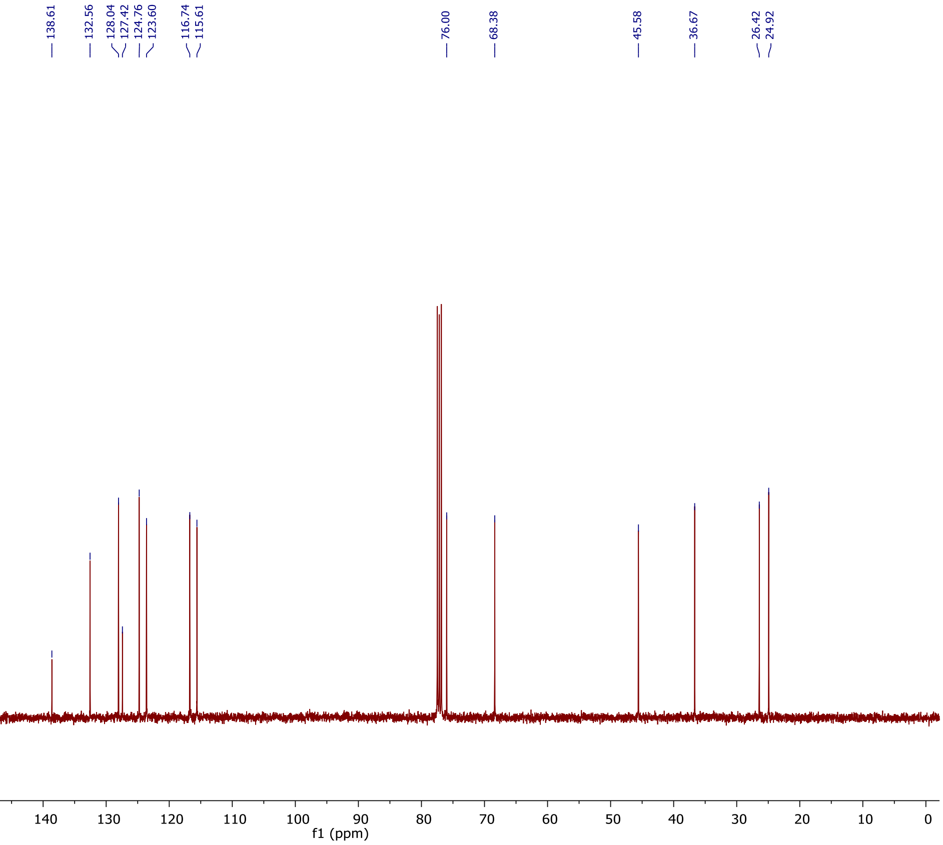

(8a*R*,12a*S*)-5,6,10,11,12,12a-Hexahydro-4*H*-pyrano[2,3-*c*]pyrido[3,2,1-*ij*]quinolin-8(8a*H*)-one (9)

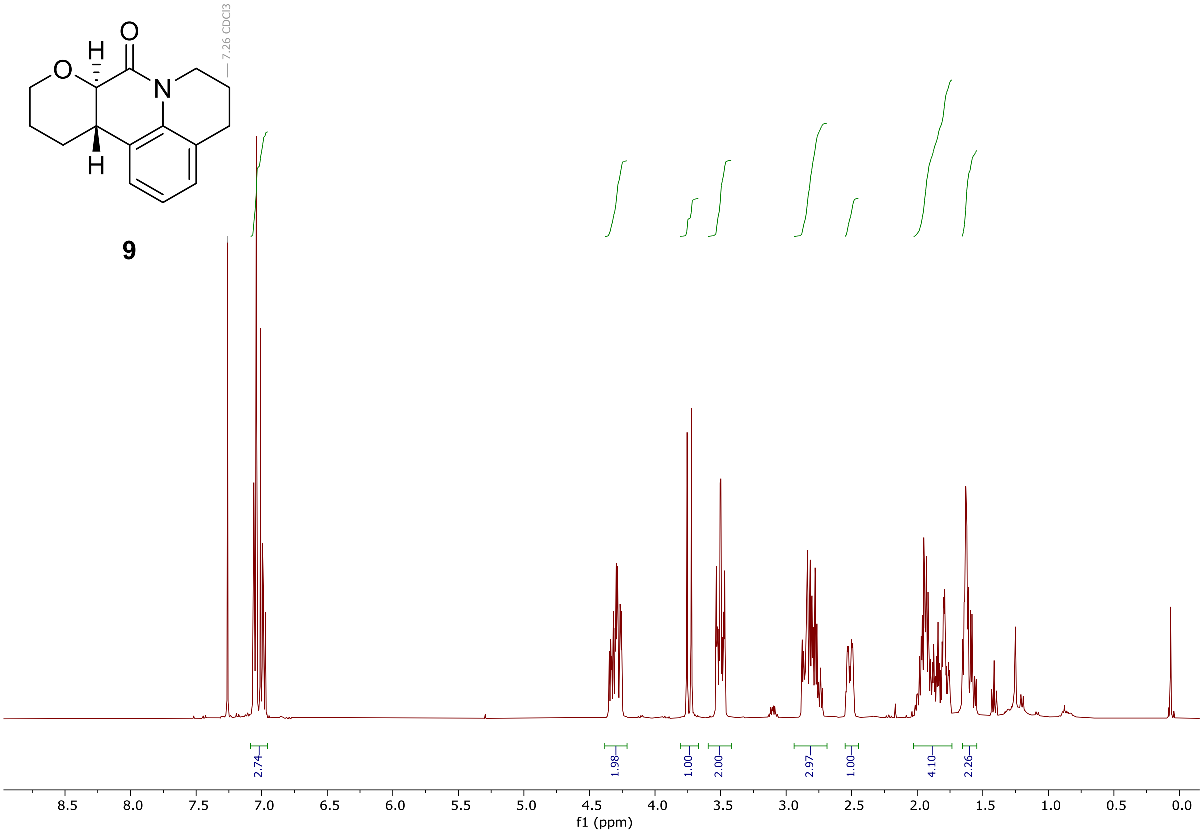

9

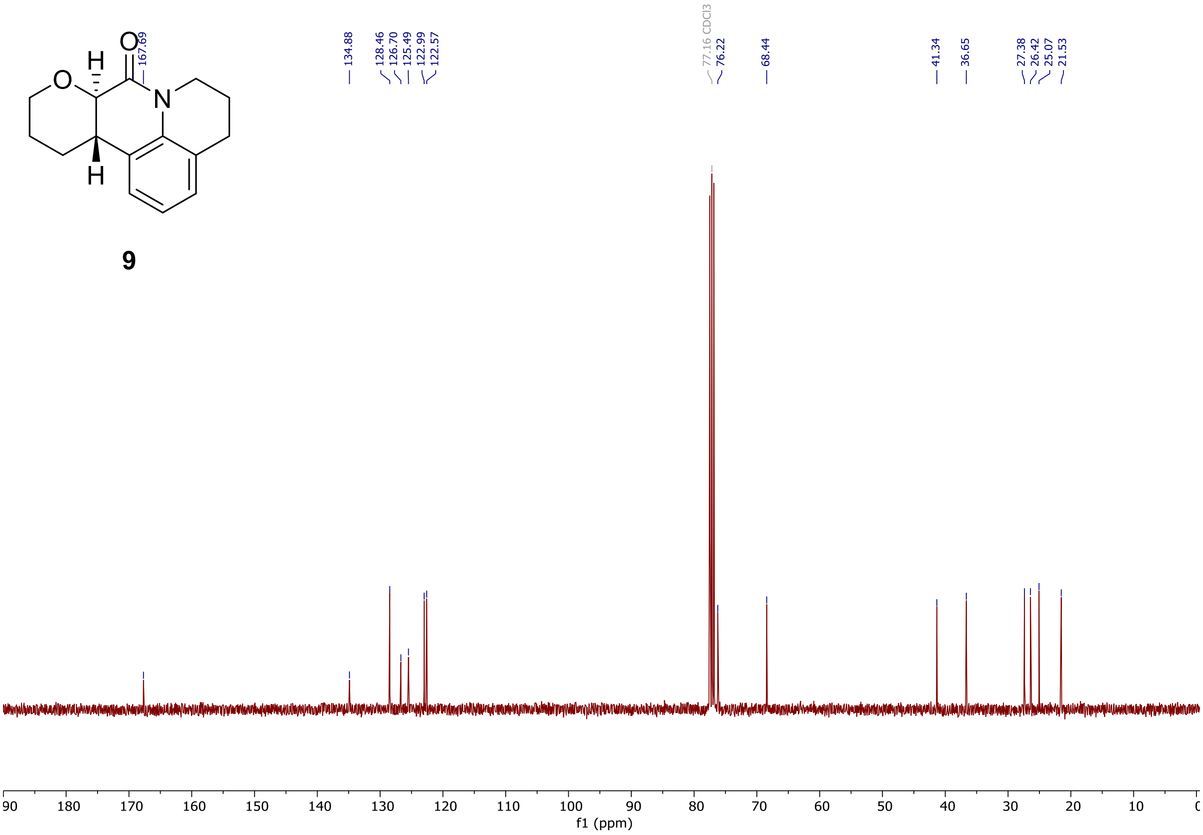

9

(4a*R*,10b*S*)-6-(4-Methoxybenzyl)-2,3,6,10b-tetrahydro-1*H*-pyrano[2,3-*c*]quinolin-5(4a*H*)-one (10)

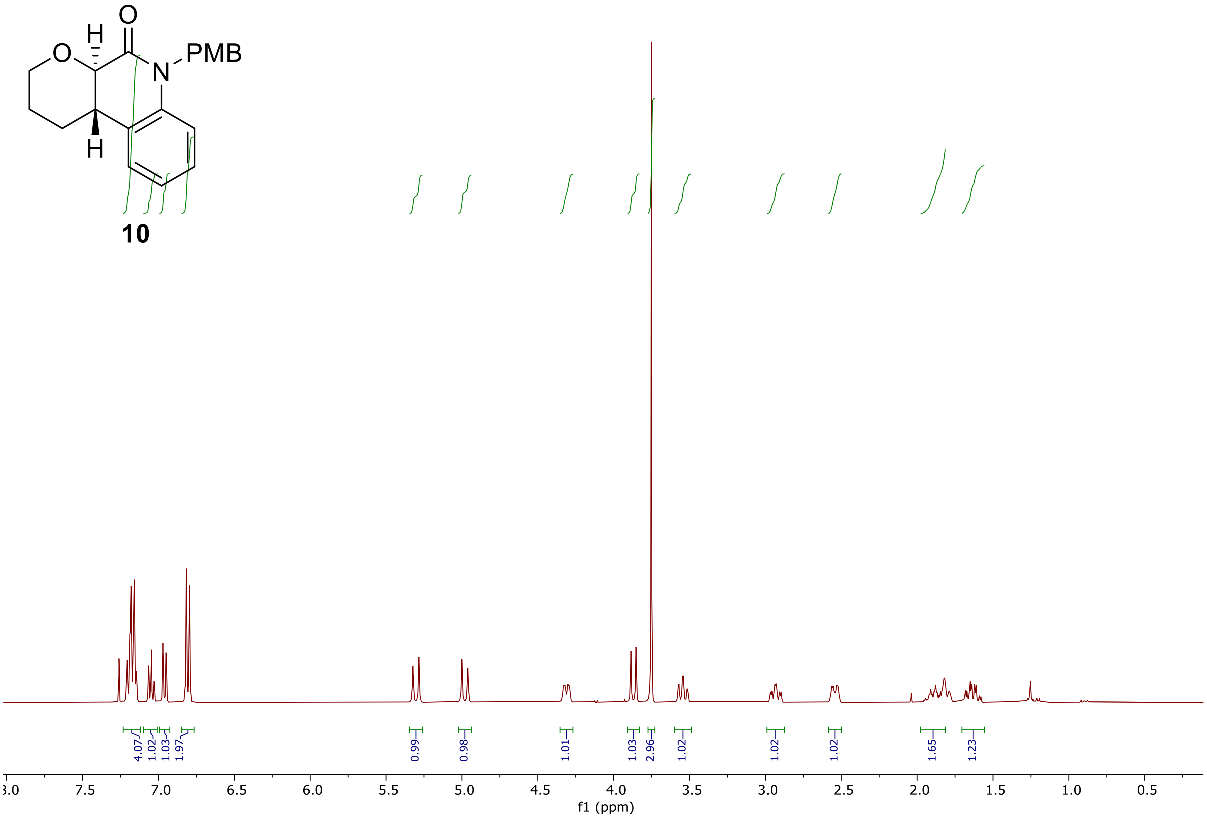

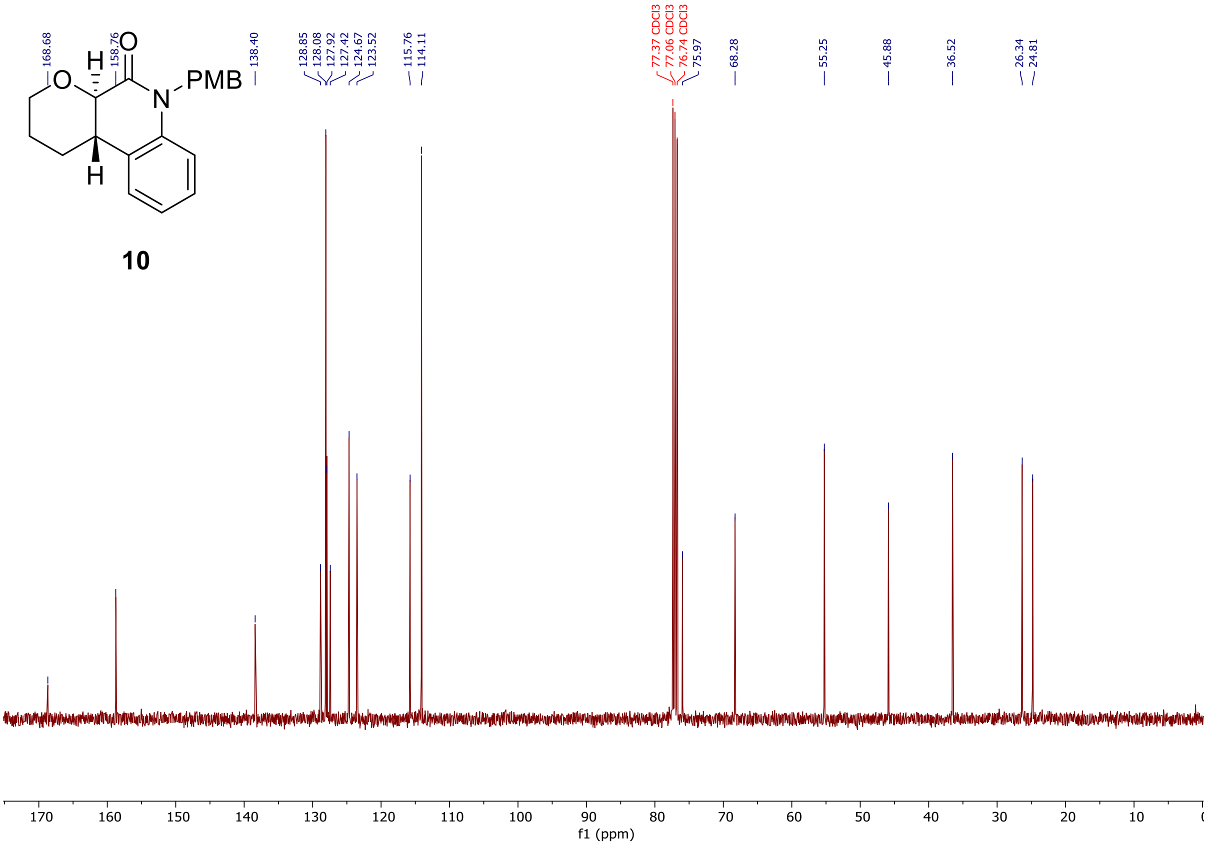

(4a*R*,10b*S*)-8-Fluoro-6-methyl-2,3,6,10b-tetrahydro-1*H*-pyrano[2,3-*c*]quinolin-5(4a*H*)-one (11), and (4a*R*,10b*S*)-10-Fluoro-6-methyl-2,3,6,10b-tetrahydro-1*H*-pyrano[2,3-*c*]quinolin-5(4a*H*)-one (11b)

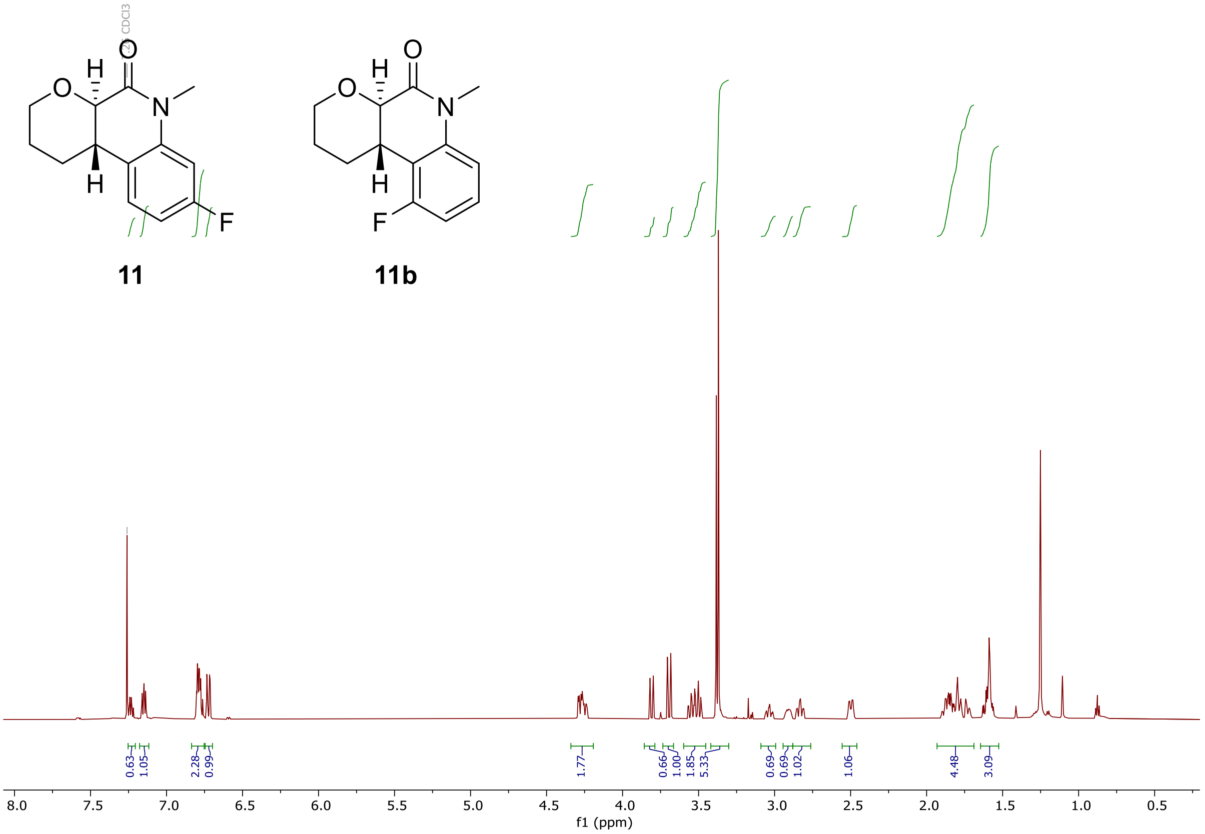

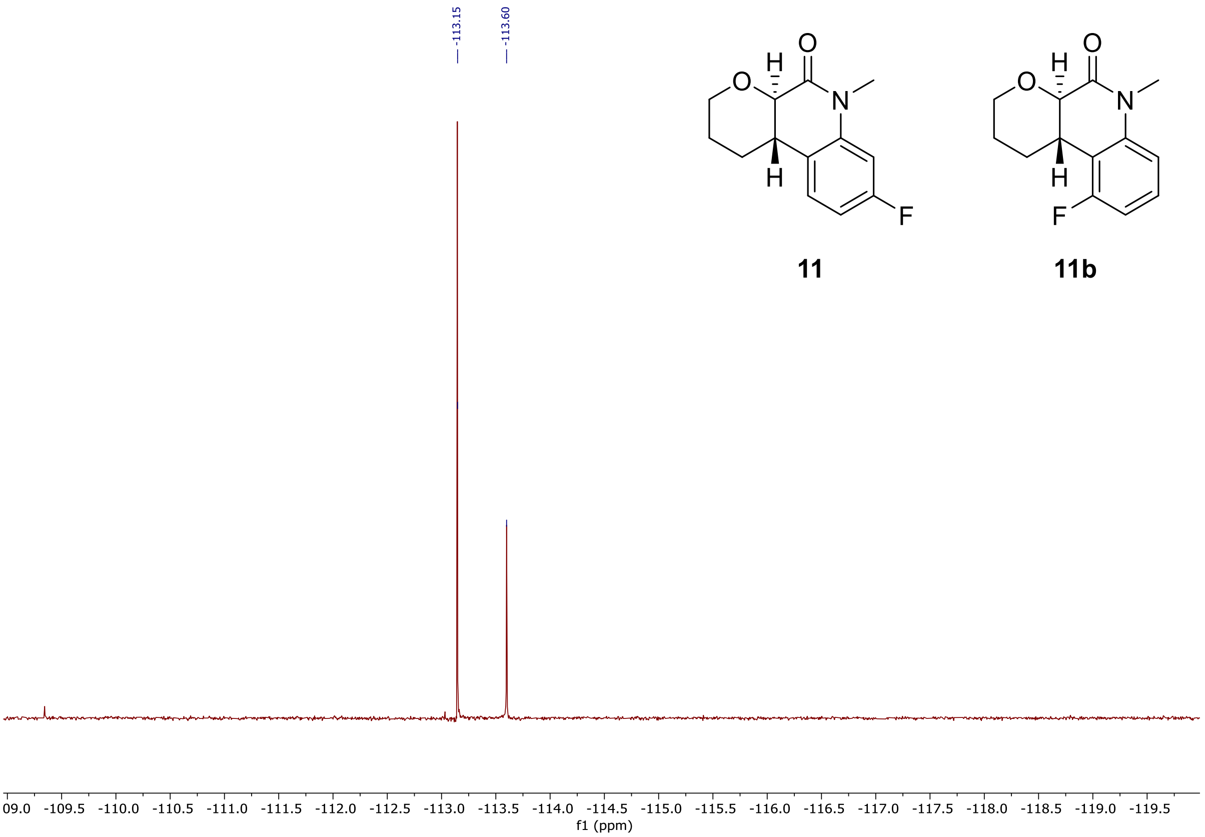

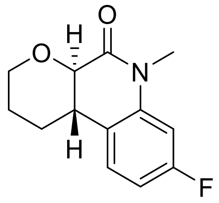

11

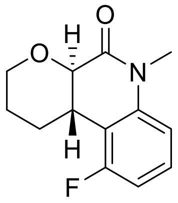

11b

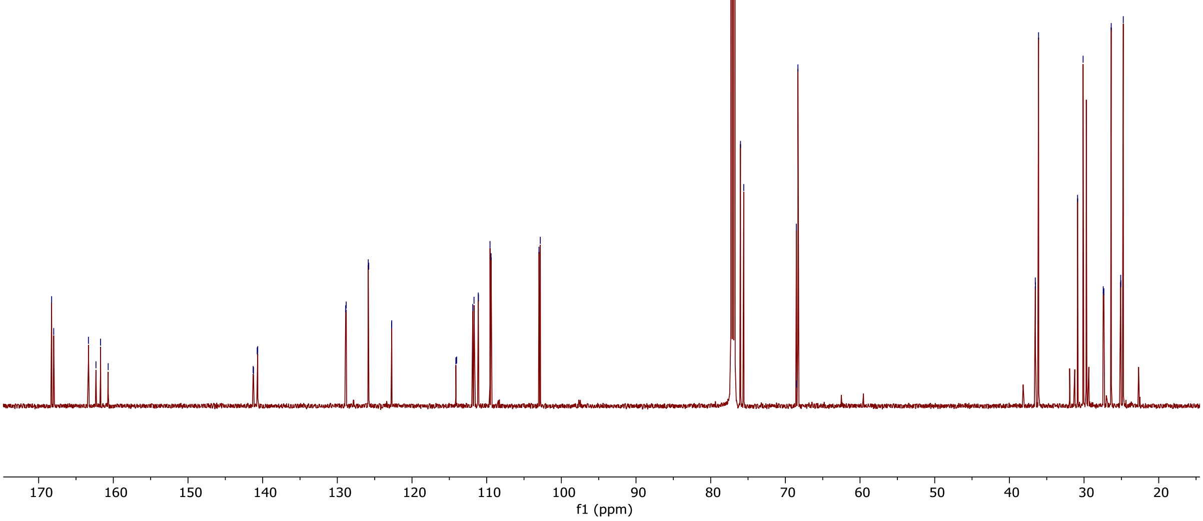
168.26
167.98
163.33
162.33
161.71
160.69
141.28
141.22
140.73
140.66
128.88
128.81
125.86
125.80
122.73
122.71
114.12
114.02
111.86
111.69
111.12
111.10
109.55
109.41
103.00
102.82
76.01
75.57
68.53
68.49
68.32
36.53
36.51
36.09
30.86
30.13
27.43
27.34
26.36
25.10
25.08
24.75
170 160 150 140 130 120 110 100 90 80 70 60 50 40 30 20
f1 (ppm)

(4a*R*,10b*S*)-8-Chloro-6-methyl-2,3,6,10b-tetrahydro-1*H*-pyrano[2,3-*c*]quinolin-5(4a*H*)-one (12) and (4aR,10bS)-10-chloro-6-methyl-2,3,6,10b-tetrahydro-1H-pyrano[2,3-c]quinolin-5(4aH)-one (12b)

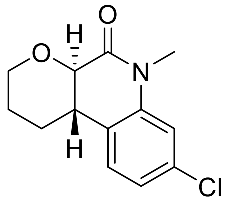

12

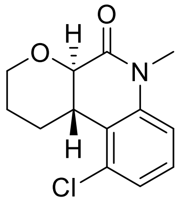

12b

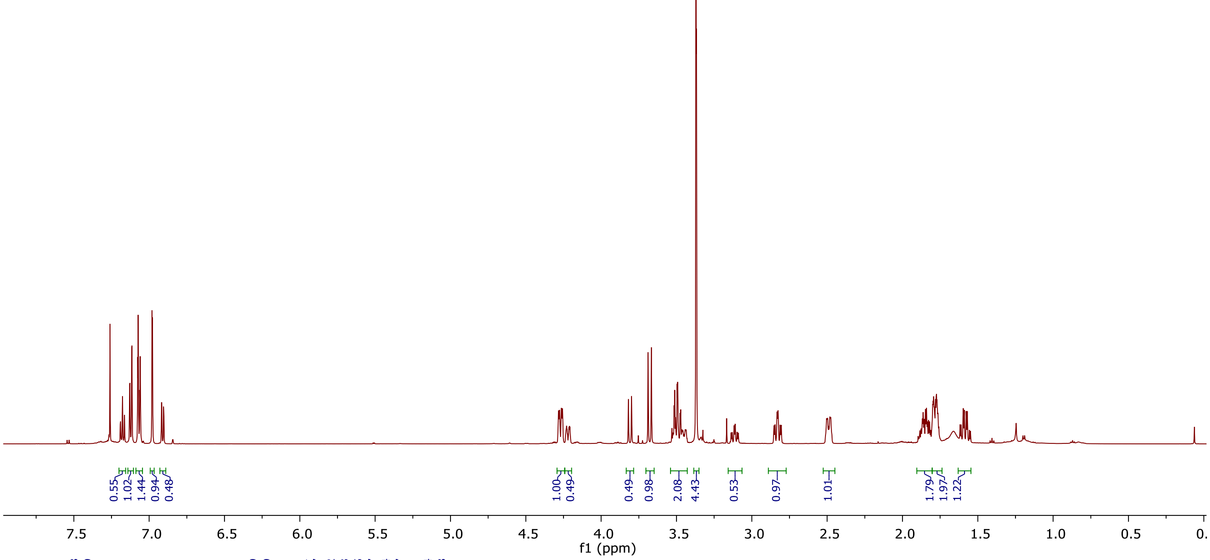

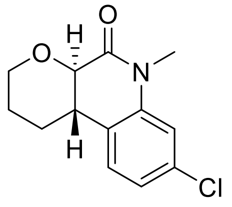

12

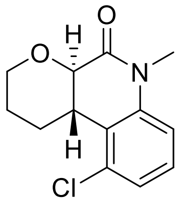

12b

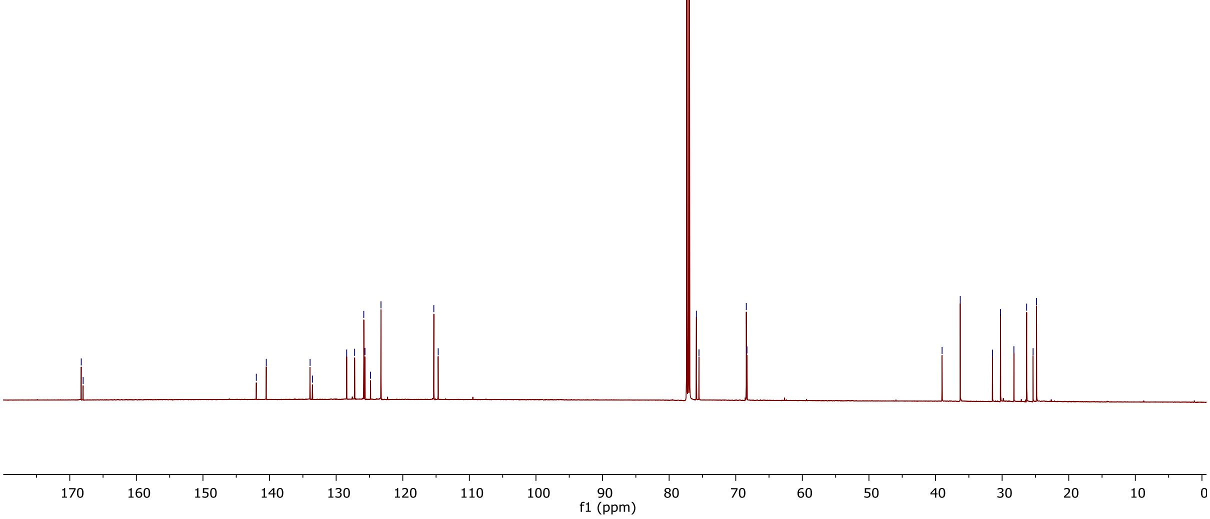

(4a*S*,10b*S*)-10-chloro-6-methyl-2,3,6,10b-tetrahydro-1*H*-pyrano[2,3-c]quinolin-5(4a*H*)-one (12b’)

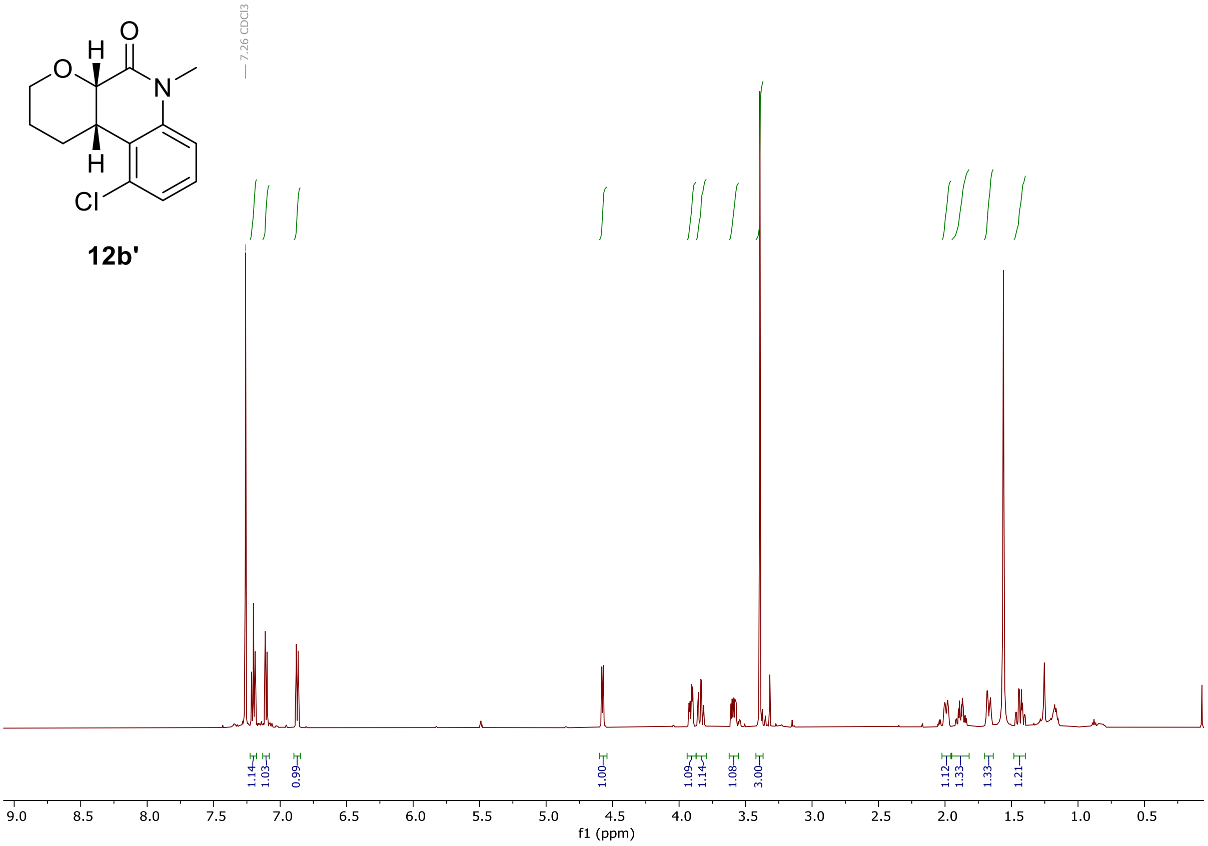

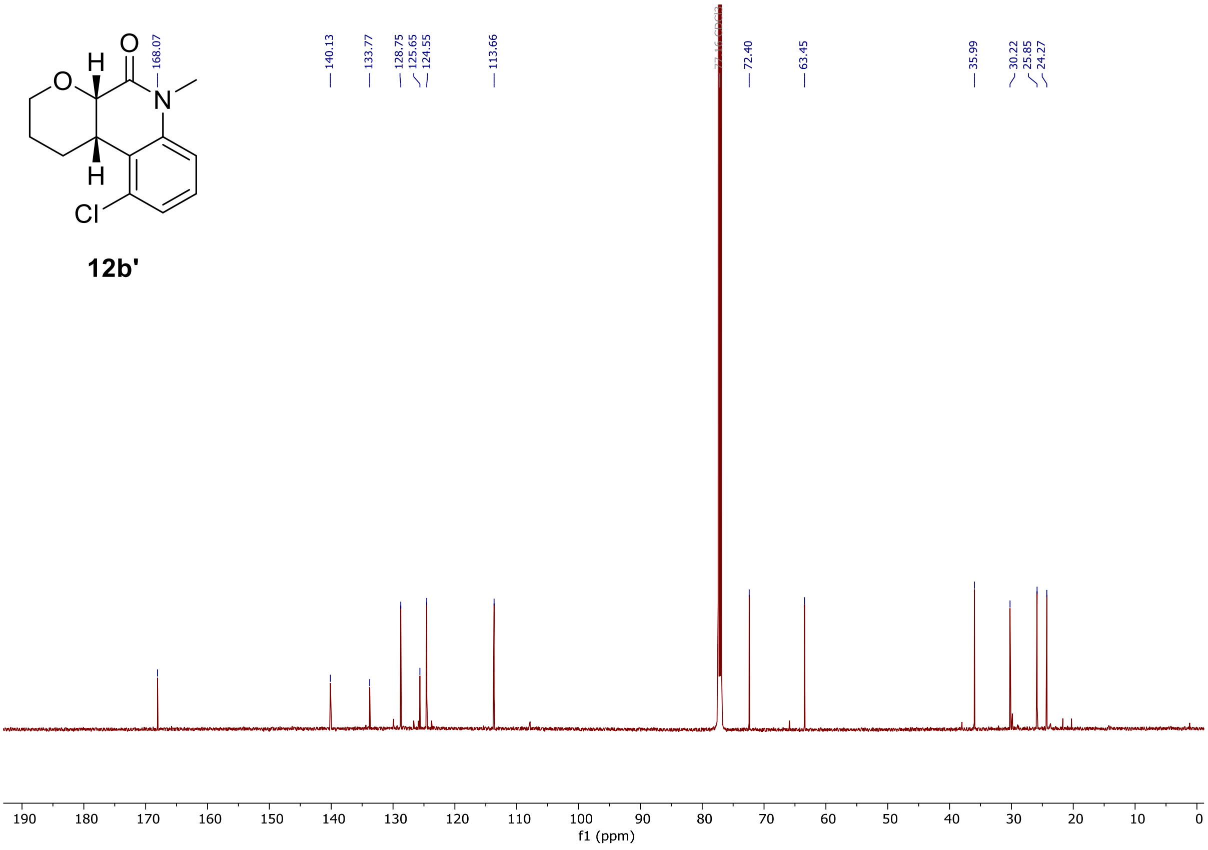

(4a*R*,10b*S*)-8-Methoxy-6-methyl-2,3,6,10b-tetrahydro-1*H*-pyrano[2,3-*c*]quinolin-5(4a*H*)-one (13), and (4a*R*,10b*S*)-10-methoxy-6-methyl-2,3,6,10b-tetrahydro-1*H*-pyrano[2,3-*c*]quinolin-5(4a*H*)-one (13b)

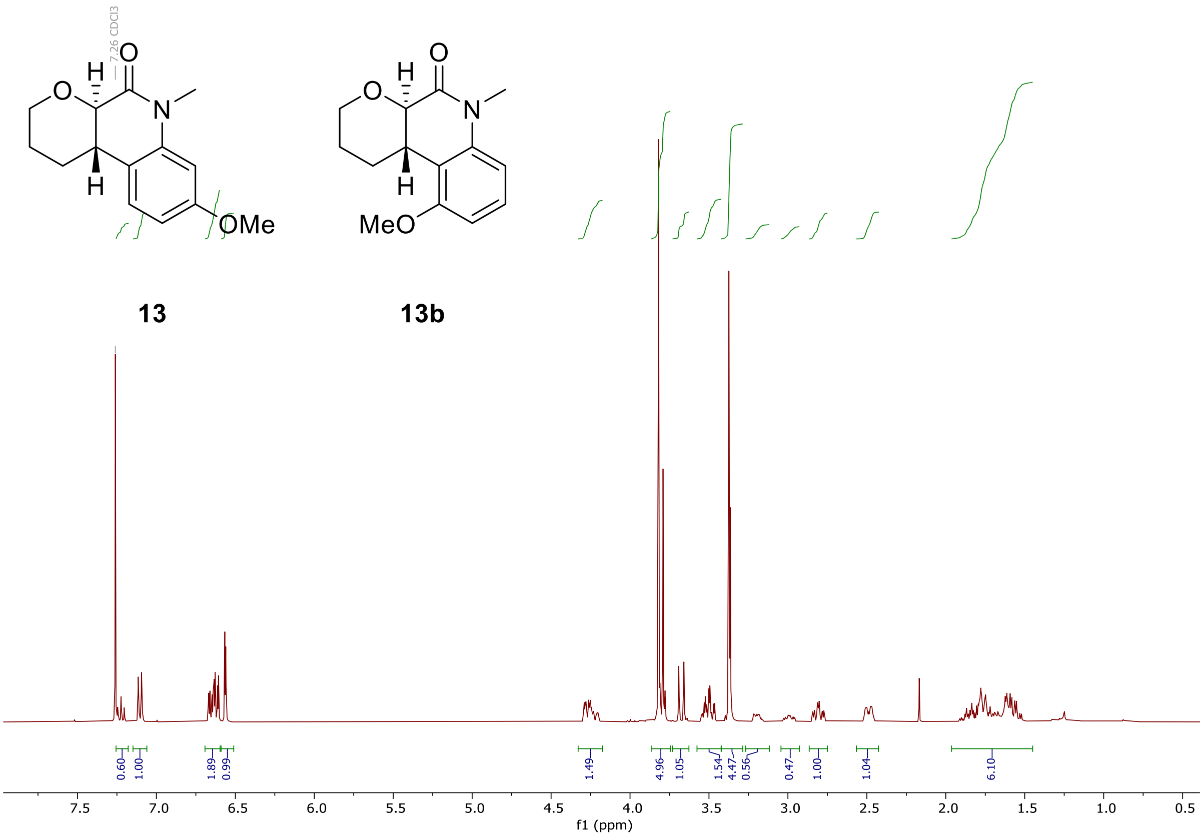

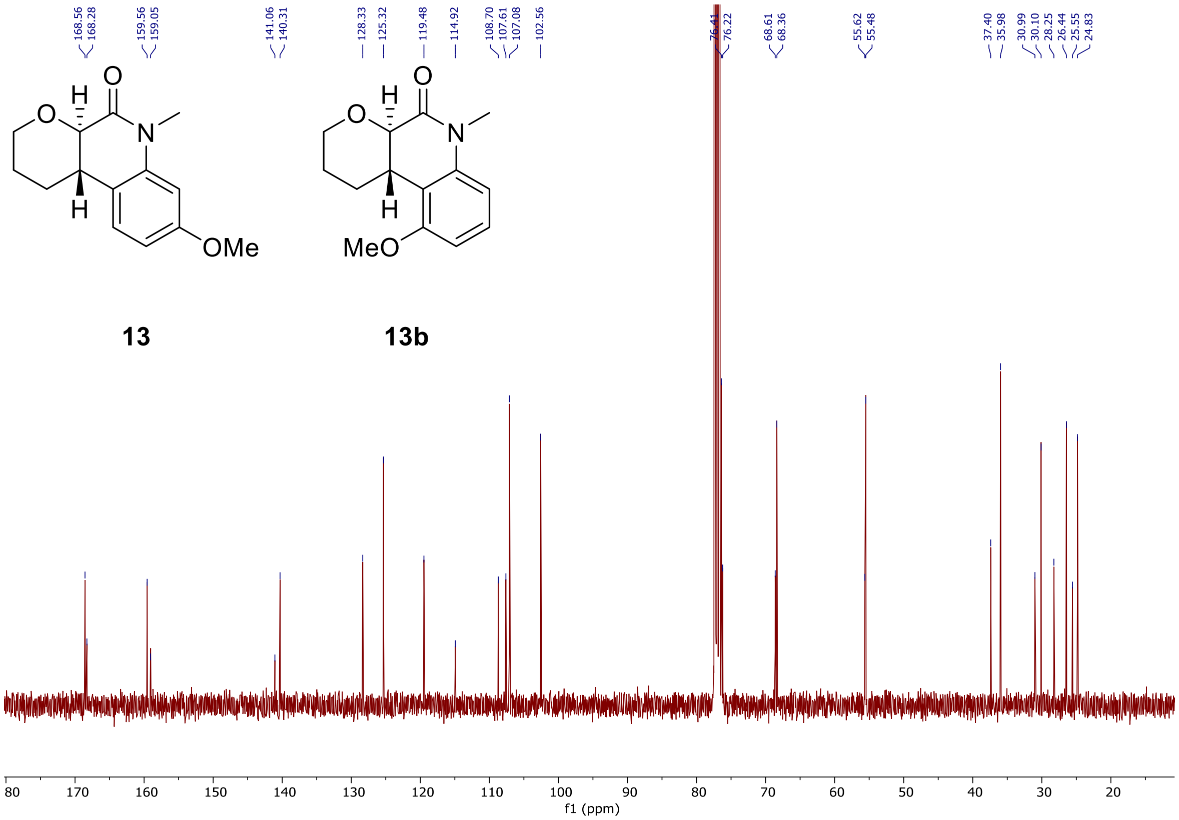

(4a*R*,10b*R*)-8-methoxy-6-methyl-2,3,6,10b-tetrahydro-1*H*-pyrano[2,3-*c*]quinolin-5(4a*H*)-one (13'')

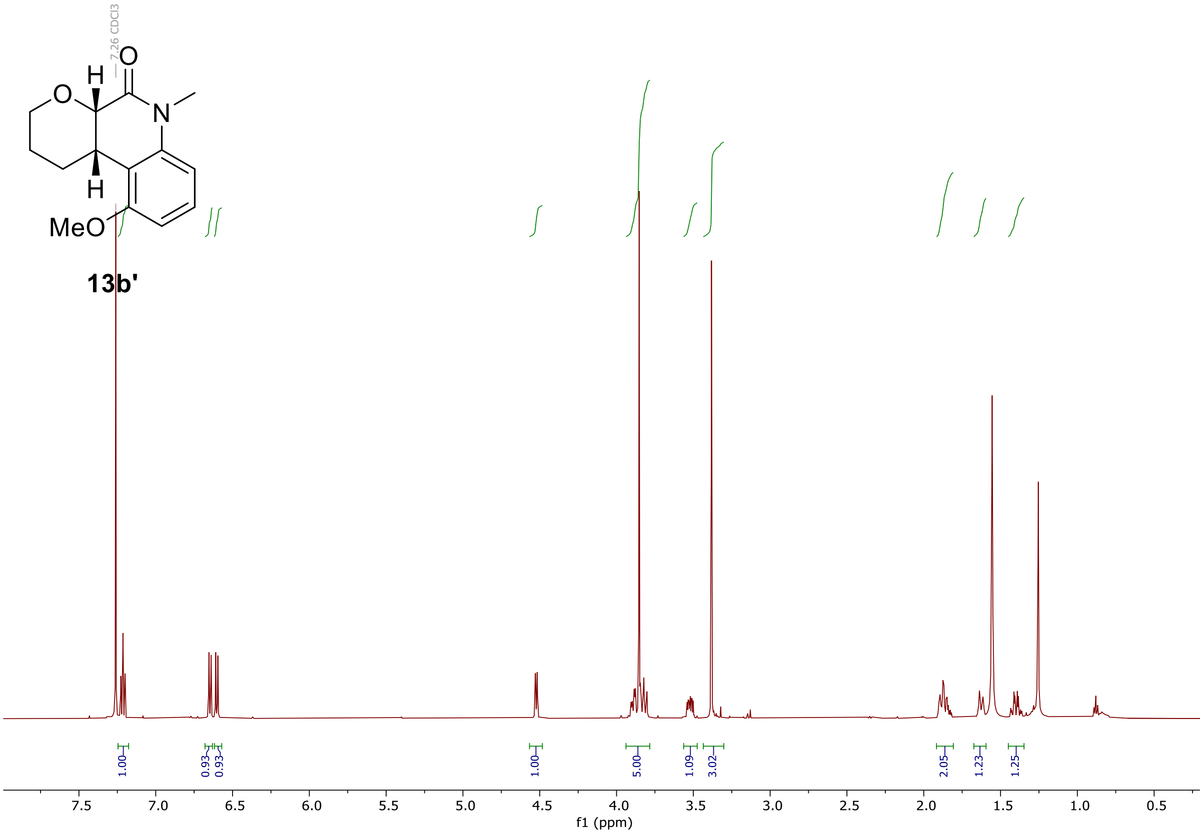

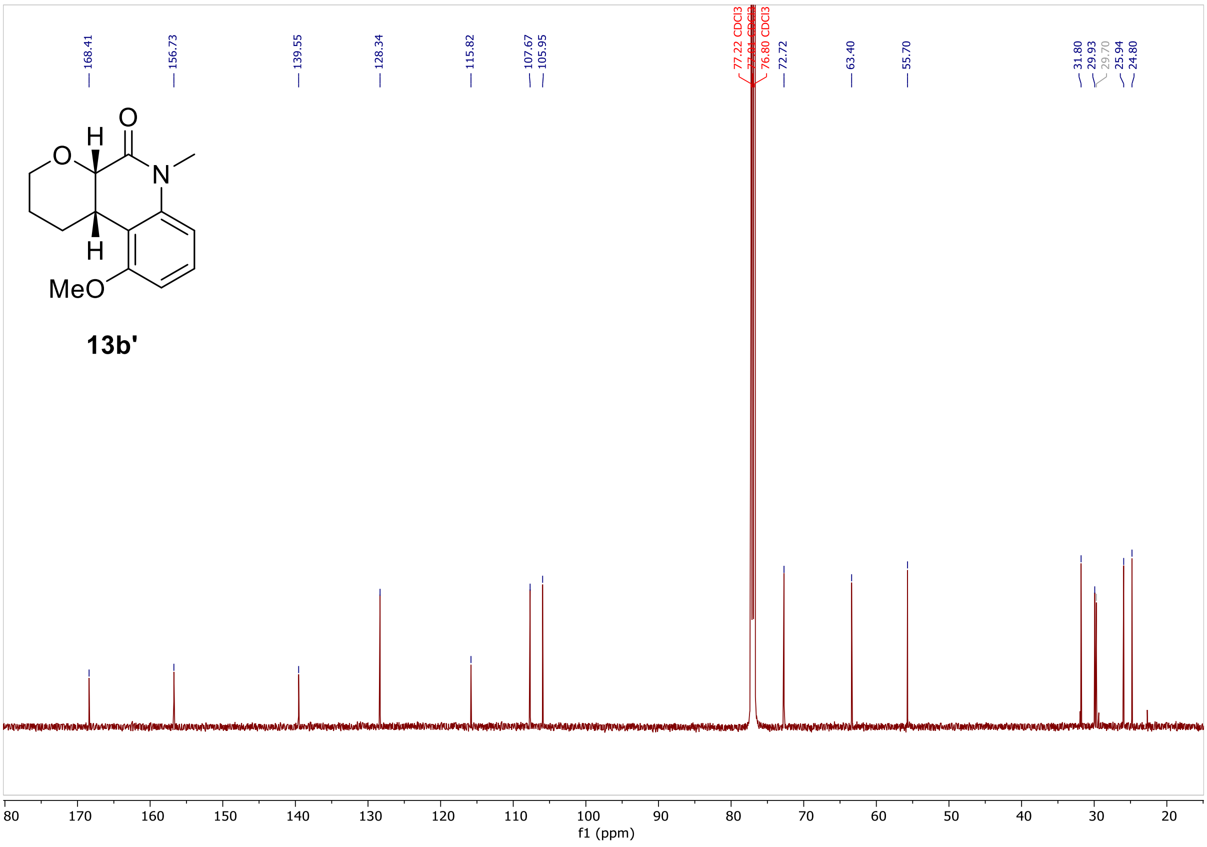

(4a*R*,10b*S*)-6,8-Dimethyl-2,3,6,10b-tetrahydro-1*H*-pyrano[2,3-*c*]quinolin-5(4a*H*)-one (14)

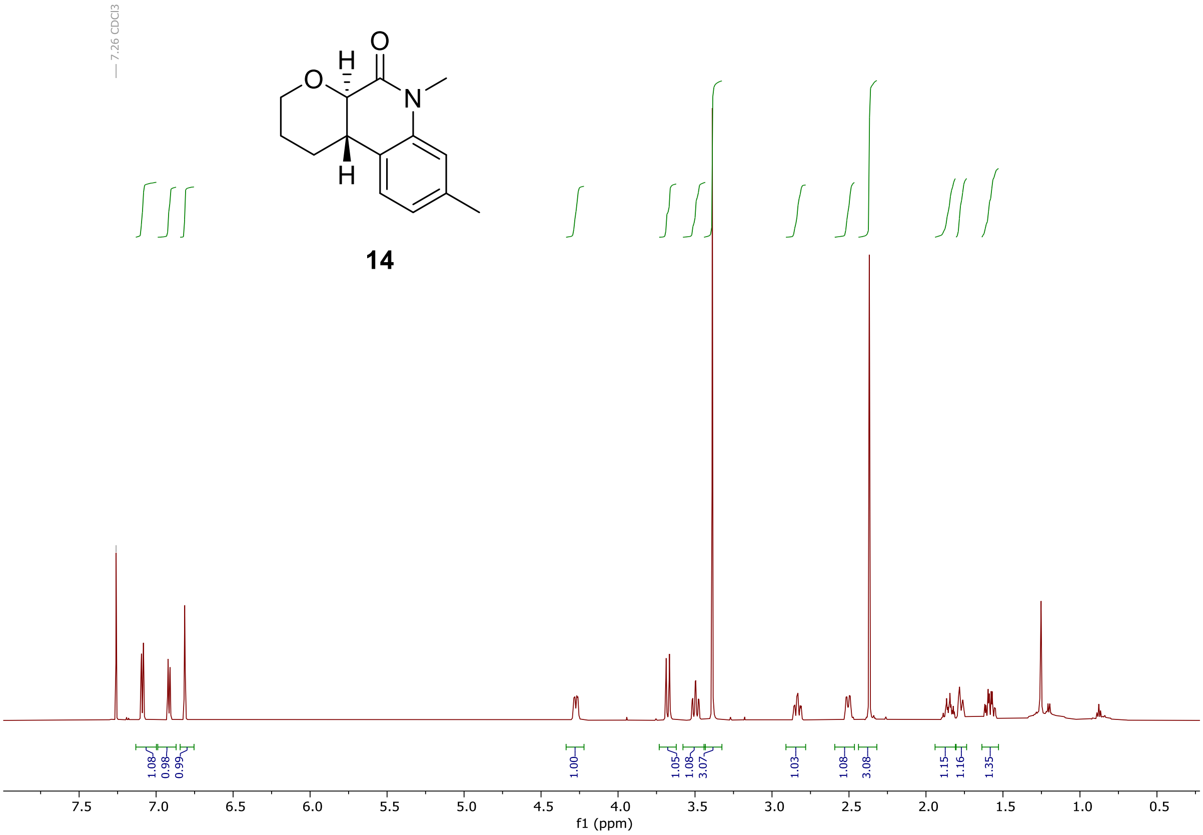

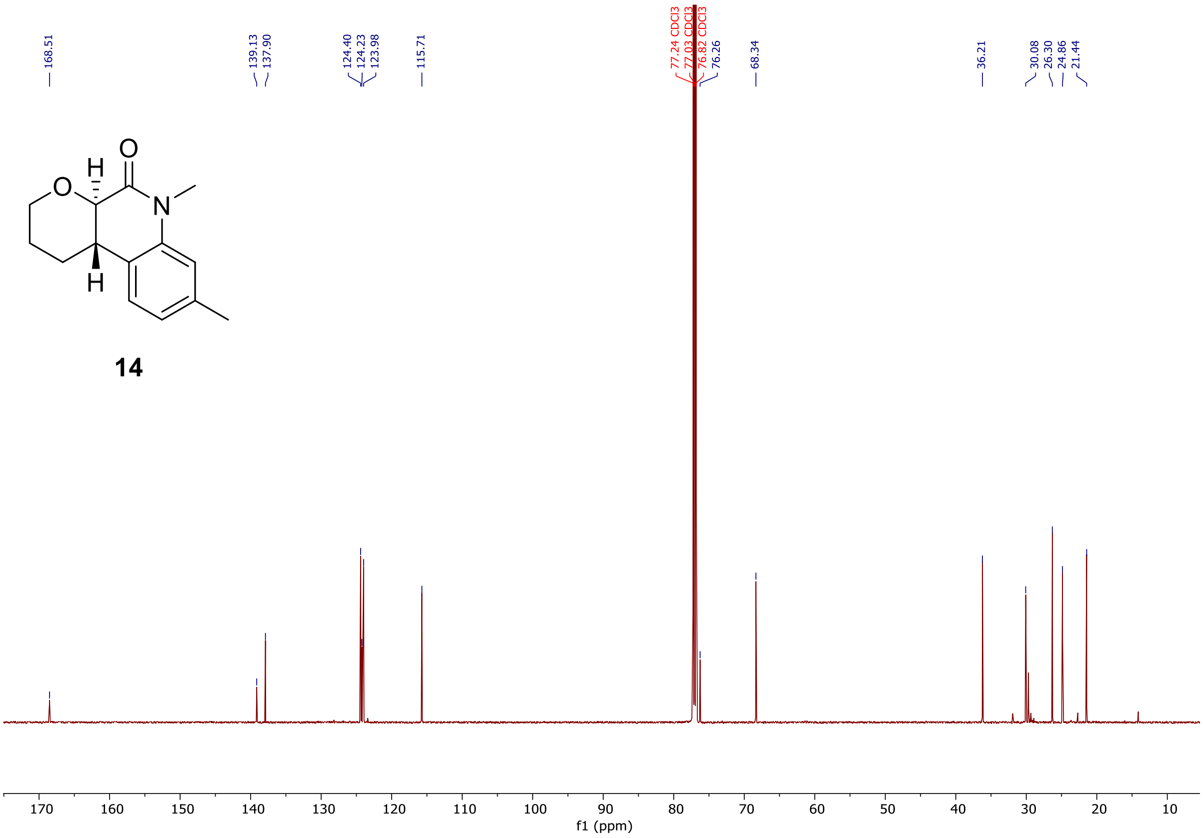

(4a*R*,10b*S*)-6,10-dimethyl-2,3,6,10b-tetrahydro-1*H*-pyrano[2,3-*c*]quinolin-5(4a*H*) (14b')

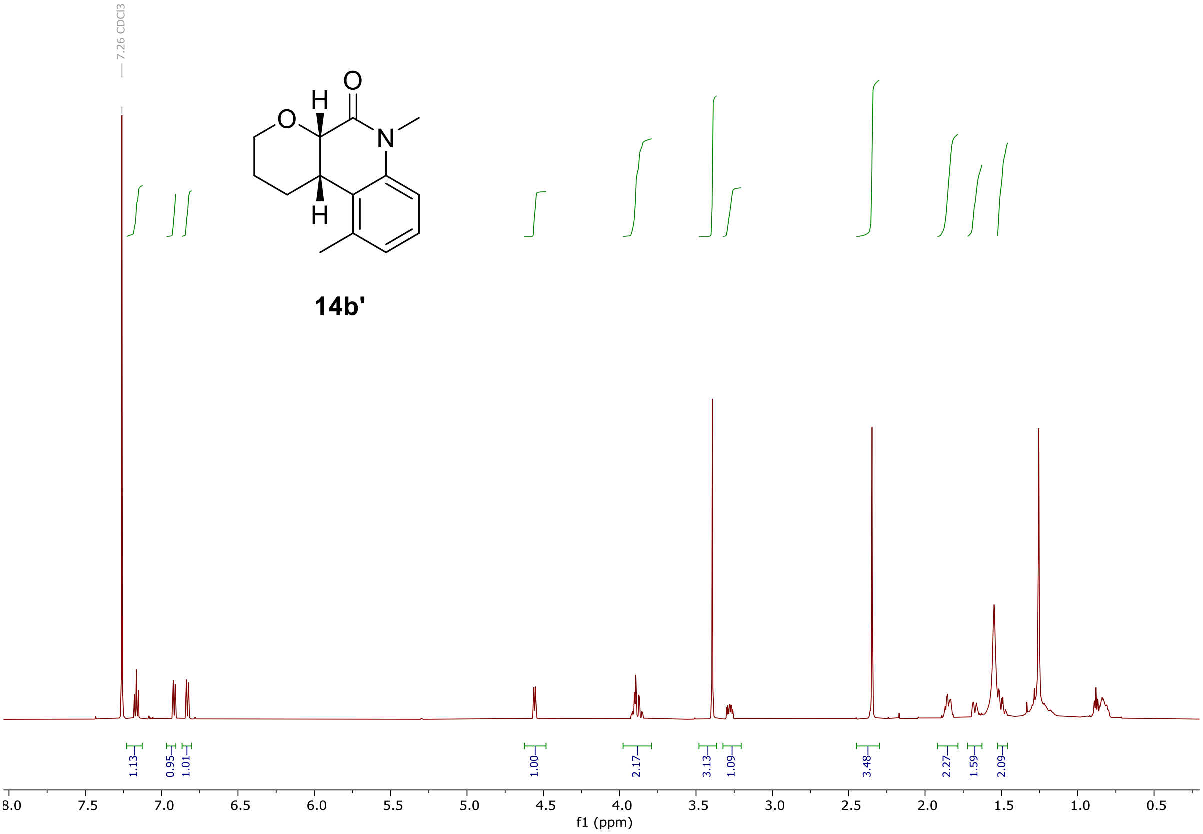

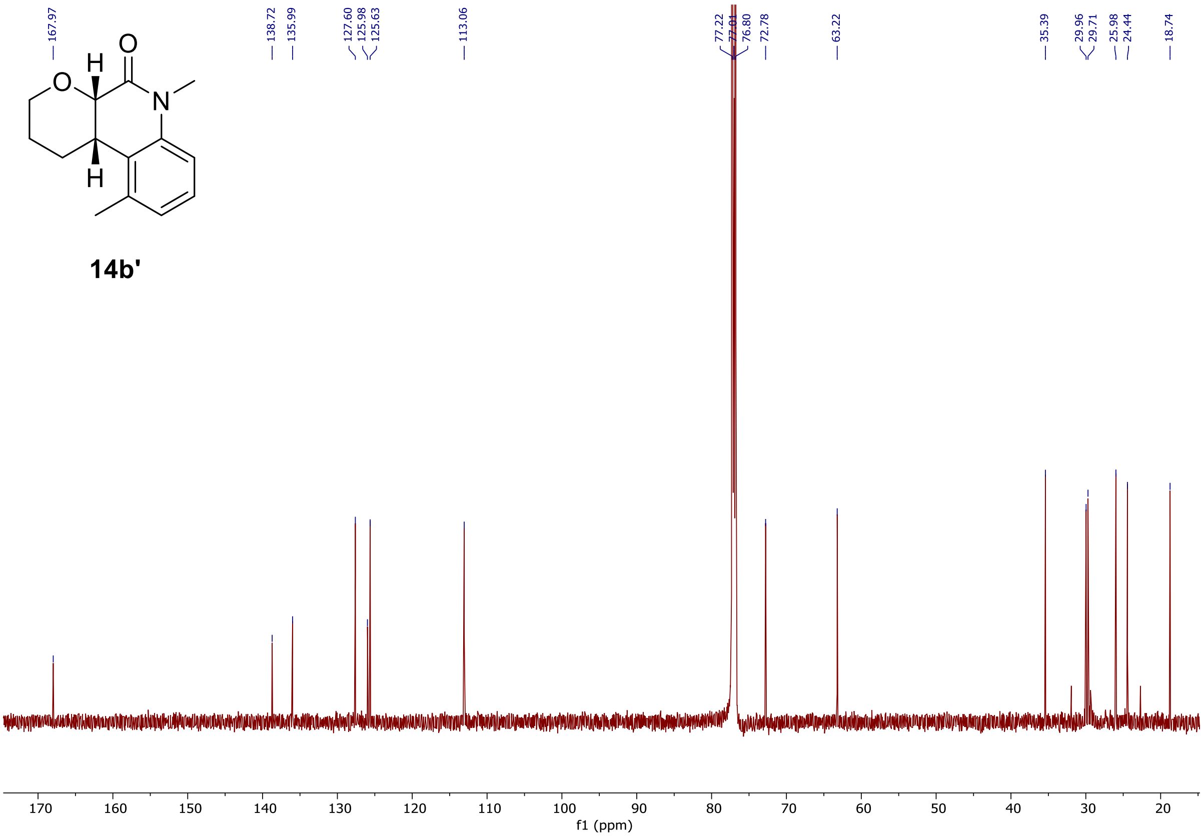

(4a*R*,10b*S*)-8,10-Difluoro-6-methyl-2,3,6,10b-tetrahydro-1*H*-pyrano[2,3-*c*]quinolin-5(4a*H*)-one (15)

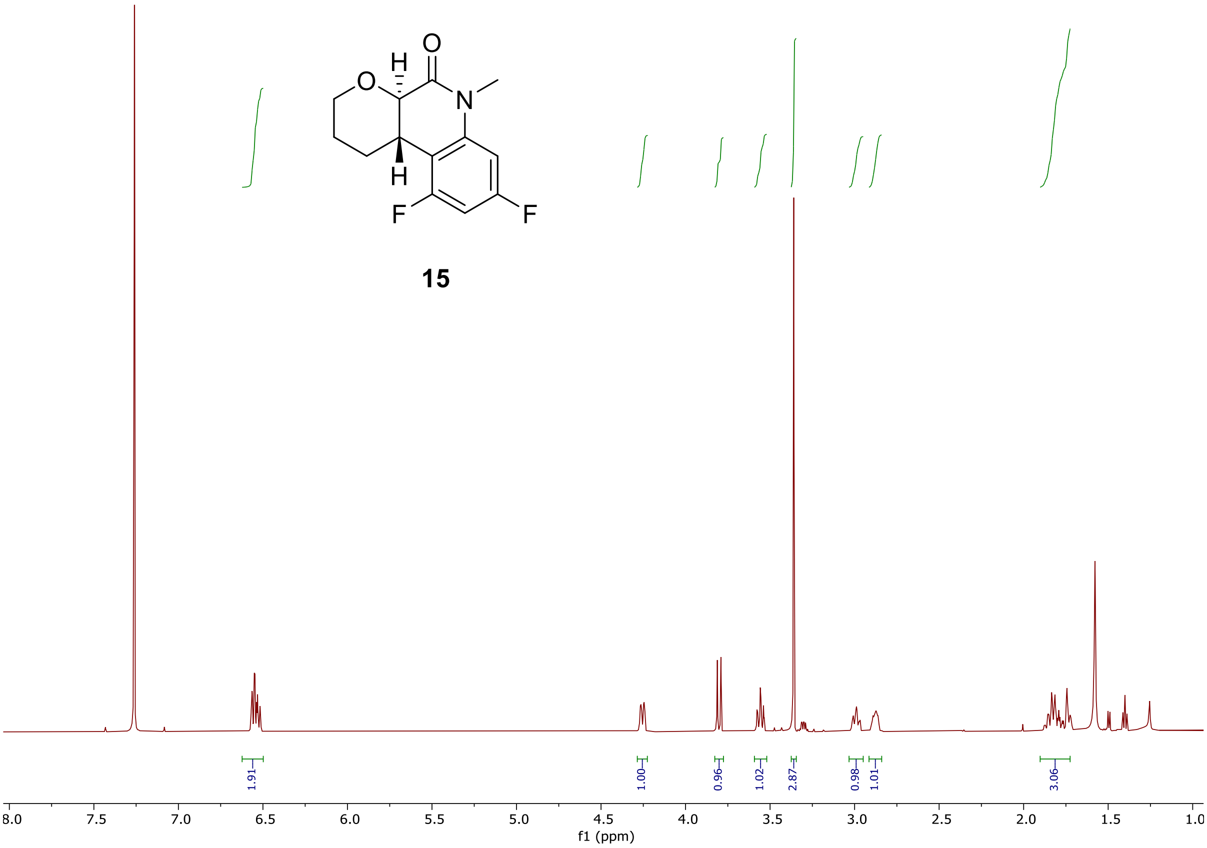

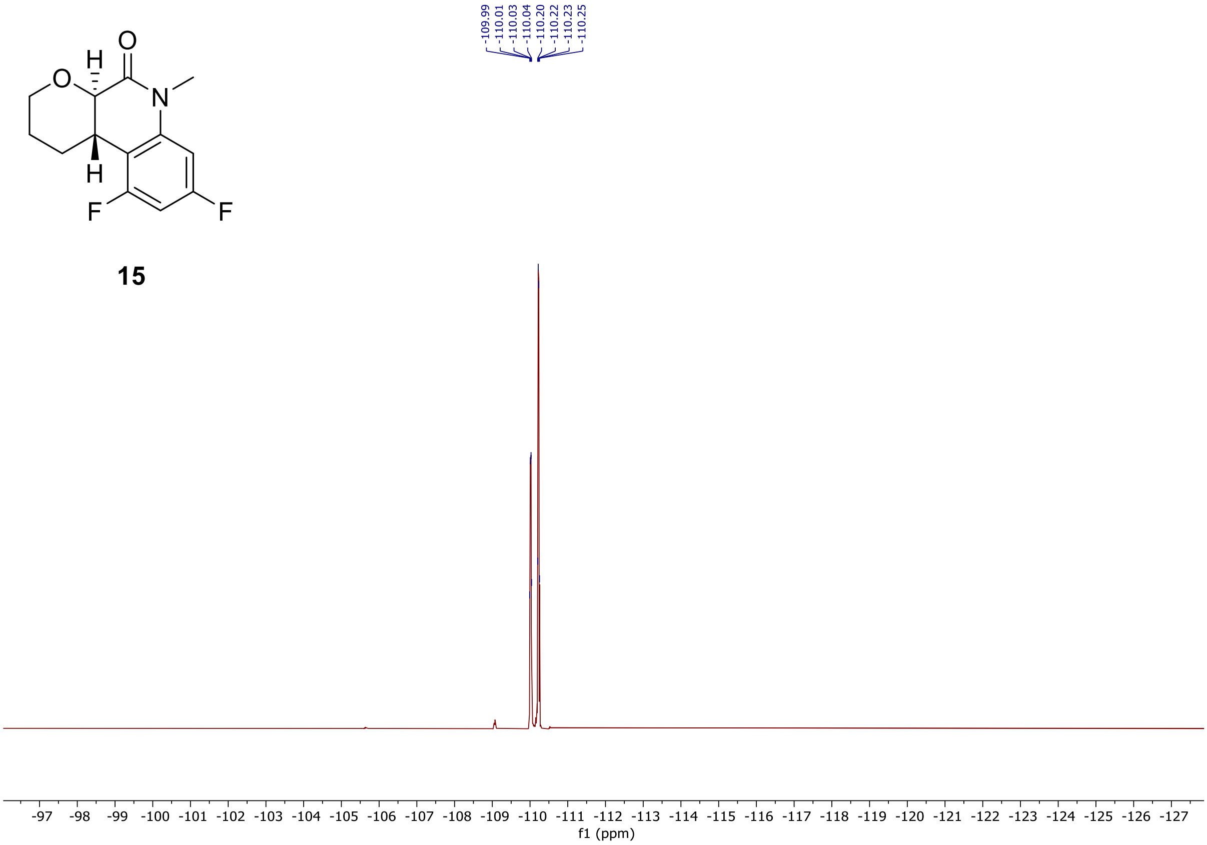

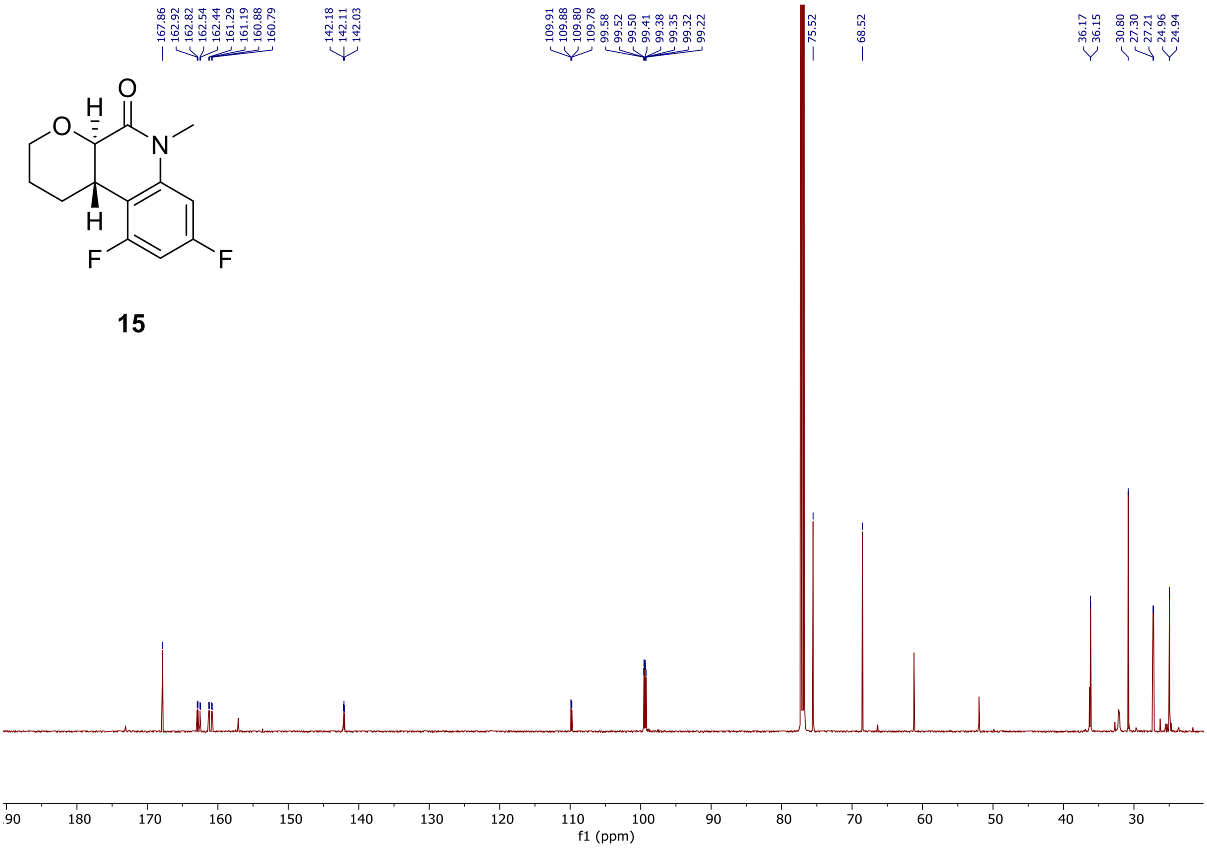

15

(4a*S*,10b*S*)-8,10-difluoro-6-methyl-2,3,6,10b-tetrahydro-1*H*-pyrano[2,3-*c*]quinolin-5(4a*H*)-one (15')

15'

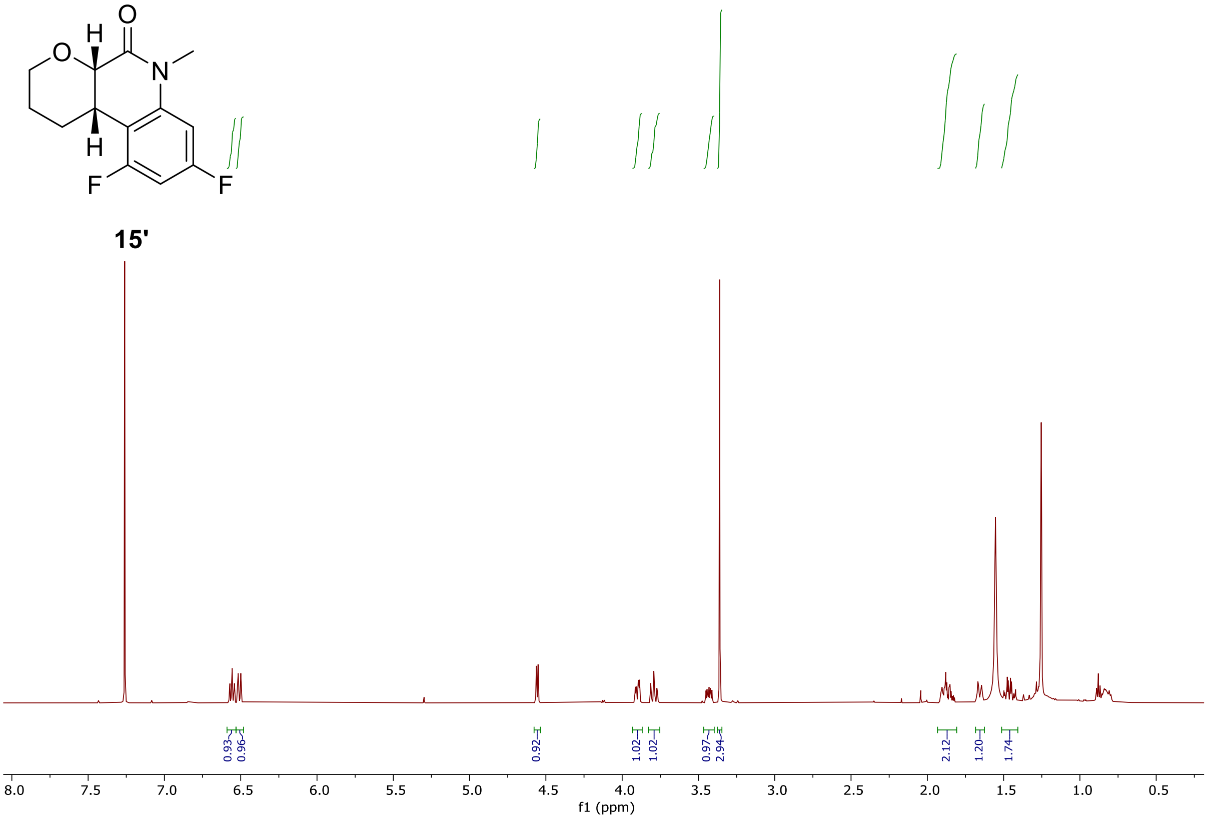

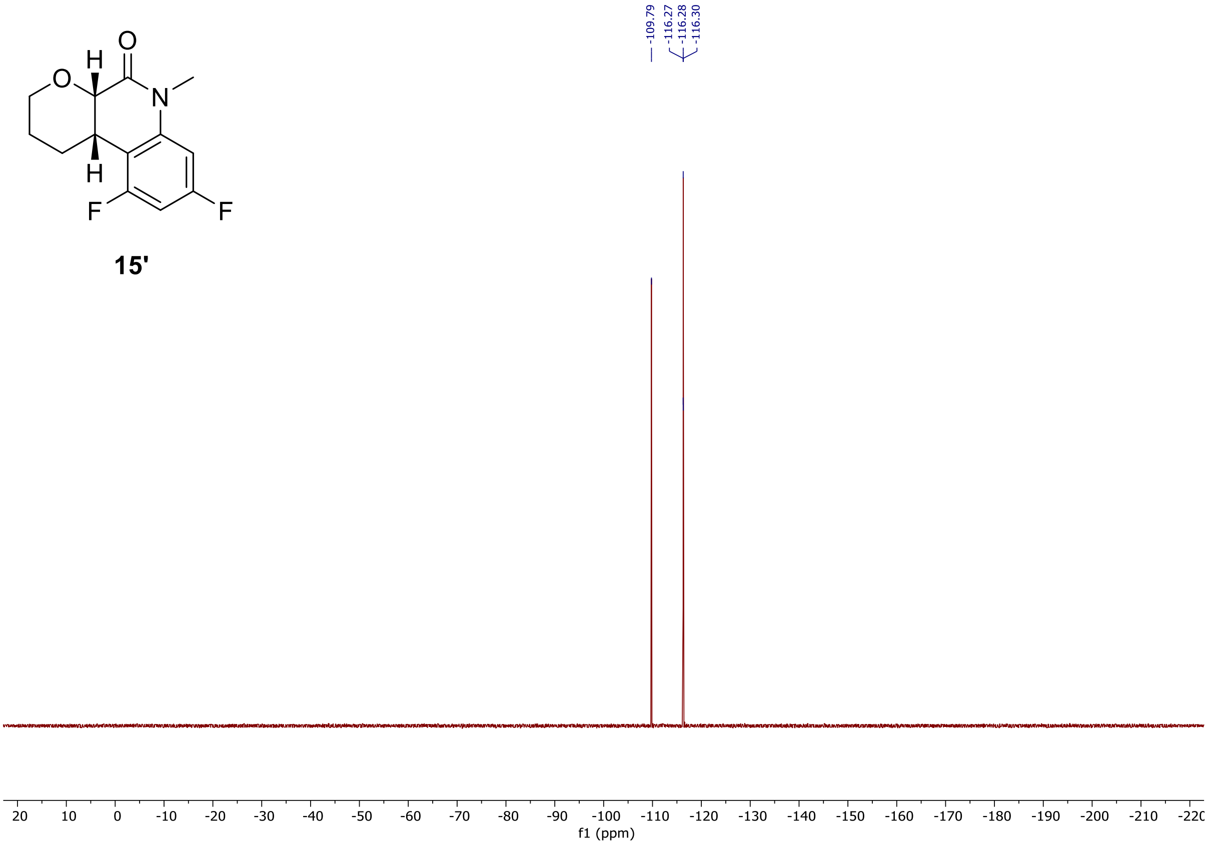

-109.79
-116.27
-116.28
-116.30
H
O
O
N
H
F
F
15'
20 10 0 -10 -20 -30 -40 -50 -60 -70 -80 -90 -100 -110 -120 -130 -140 -150 -160 -170 -180 -190 -200 -210 -220
f1 (ppm)

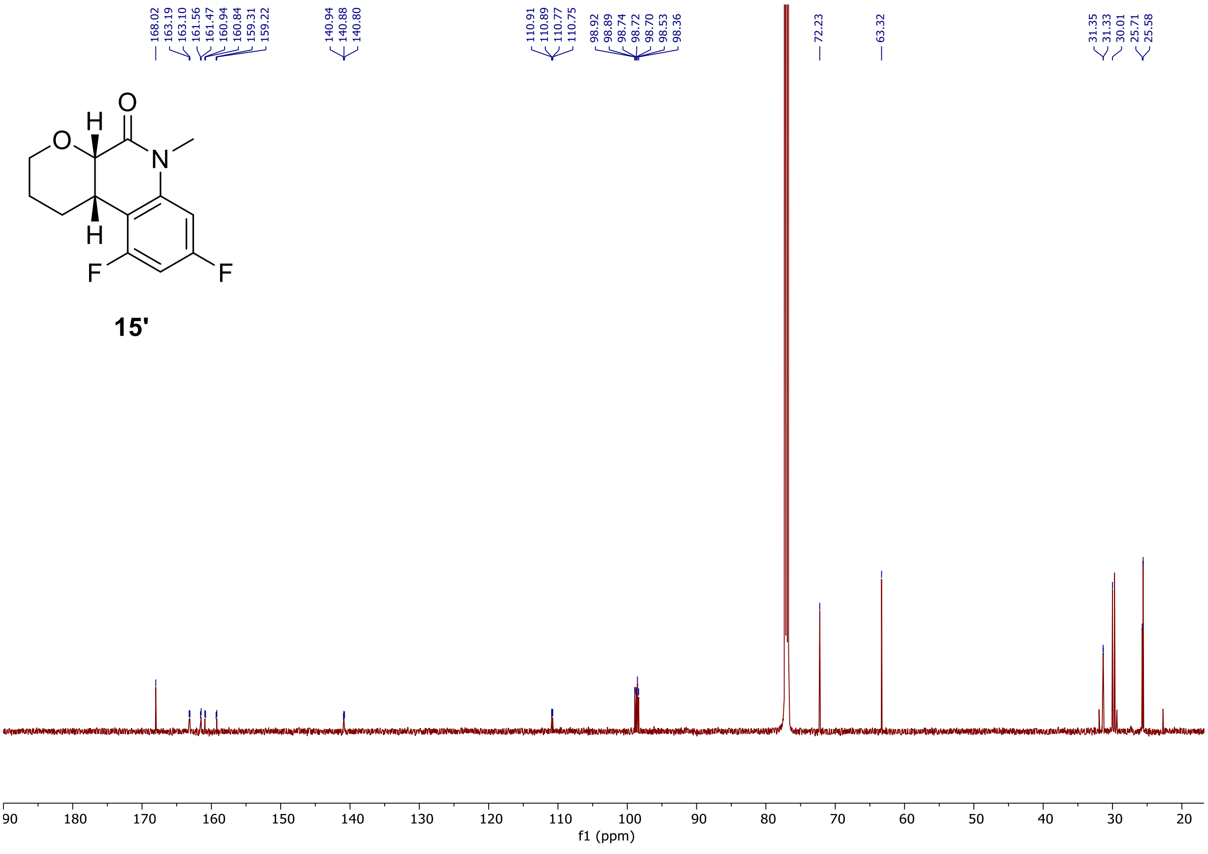

168.02
163.19
163.10
161.56
161.47
160.94
160.84
159.31
159.22
140.94
140.88
140.80
110.91
110.89
110.77
110.75
98.92
98.89
98.74
98.72
98.70
98.53
98.36
72.23
63.32
31.35
31.33
30.01
25.71
25.58
H
O
O
N
H
F
F
15'
190 180 170 160 150 140 130 120 110 100 90 80 70 60 50 40 30 20
f1 (ppm)

(4a*R*,10b*S*)-9-Fluoro-6-methyl-2,3,6,10b-tetrahydro-1*H*-pyrano[2,3-c]quinolin-5(4a*H*)-one (16)

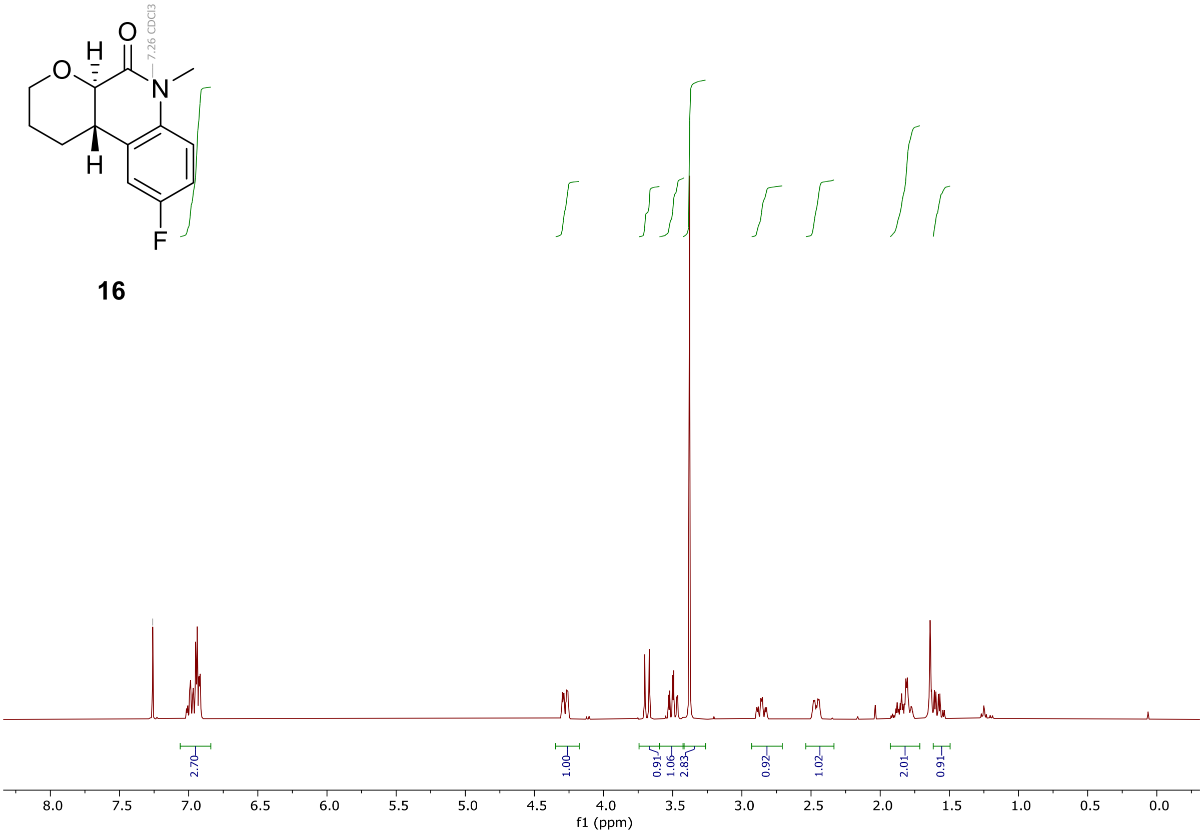

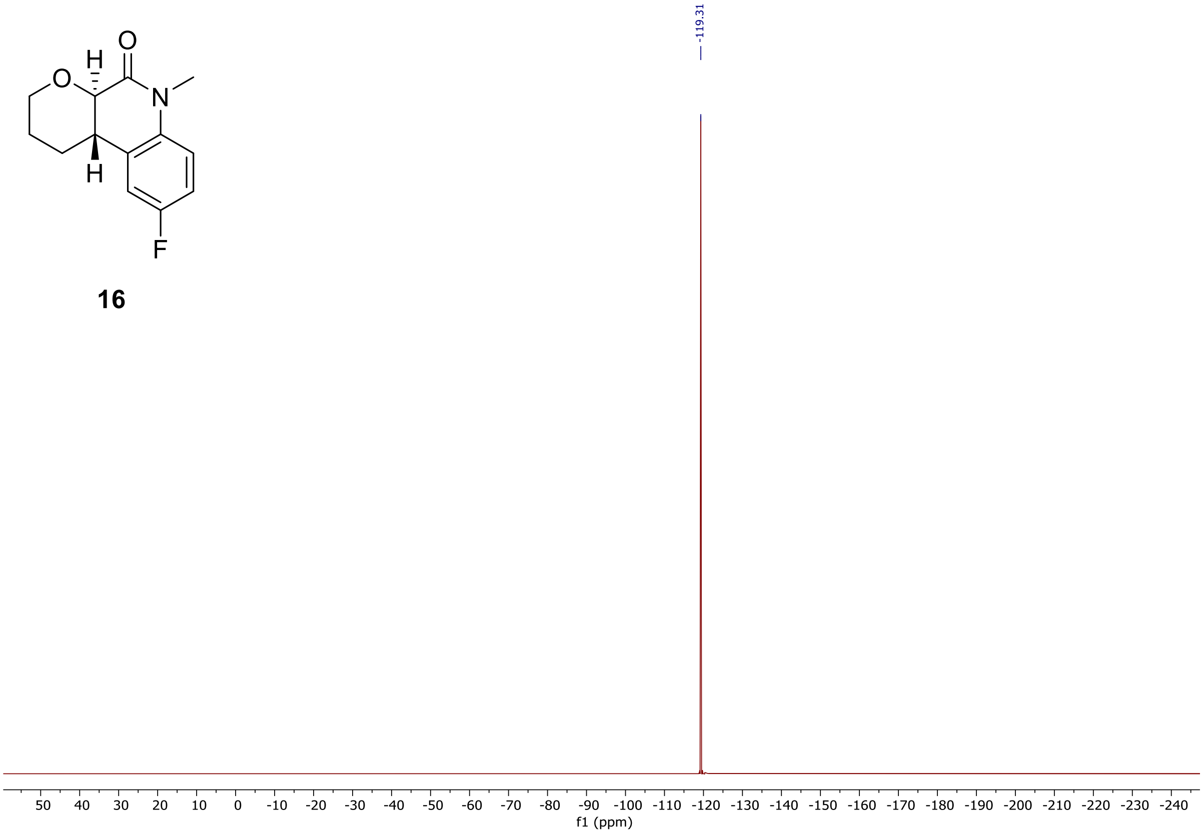

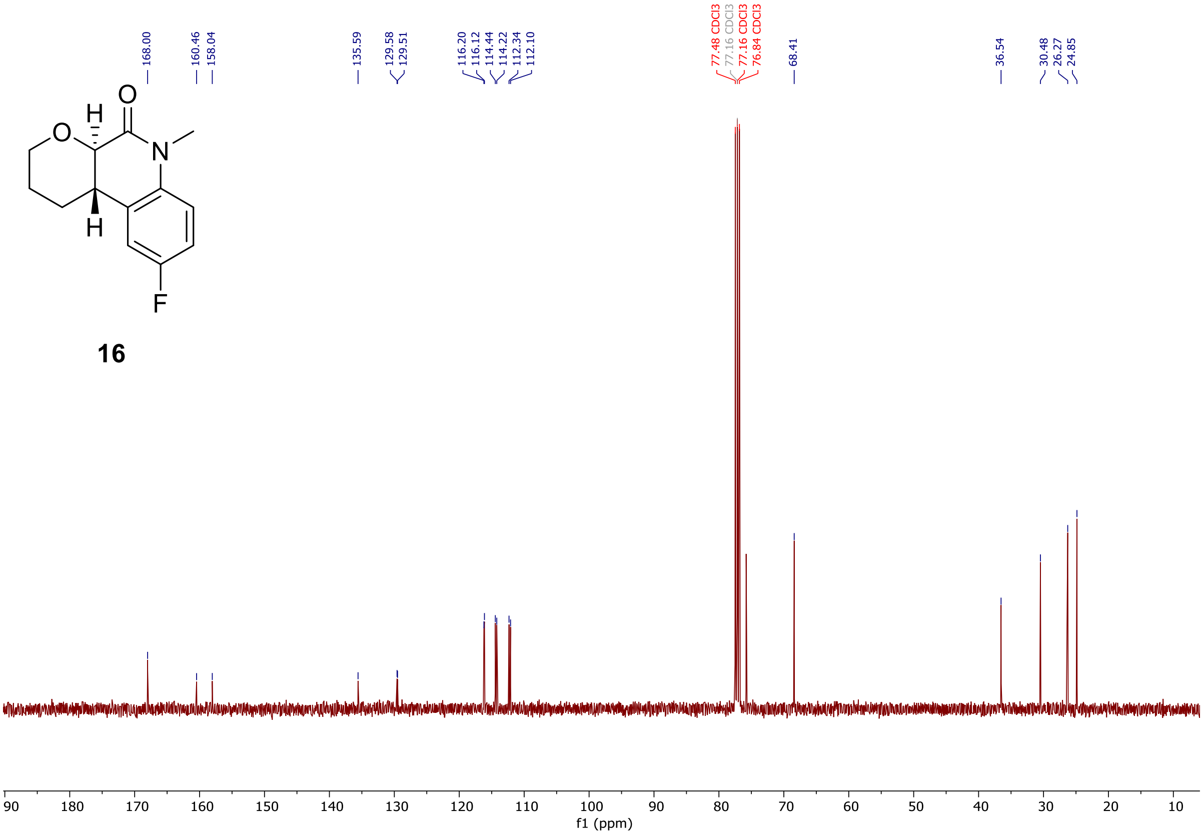
168.00
160.46
158.04
135.59
129.58
129.51
116.20
116.12
114.44
114.22
112.34
112.10
77.48 CDCl3
77.16 CDCl3
77.16 CDCl3
76.84 CDCl3
68.41
36.54
30.48
26.27
24.85
f1 (ppm)

16

(4a*R*,10b*S*)-6-Benzyl-9-chloro-2,3,6,10b-tetrahydro-1*H*-pyrano[2,3-*c*]quinolin-5(4a*H*)-one (17)

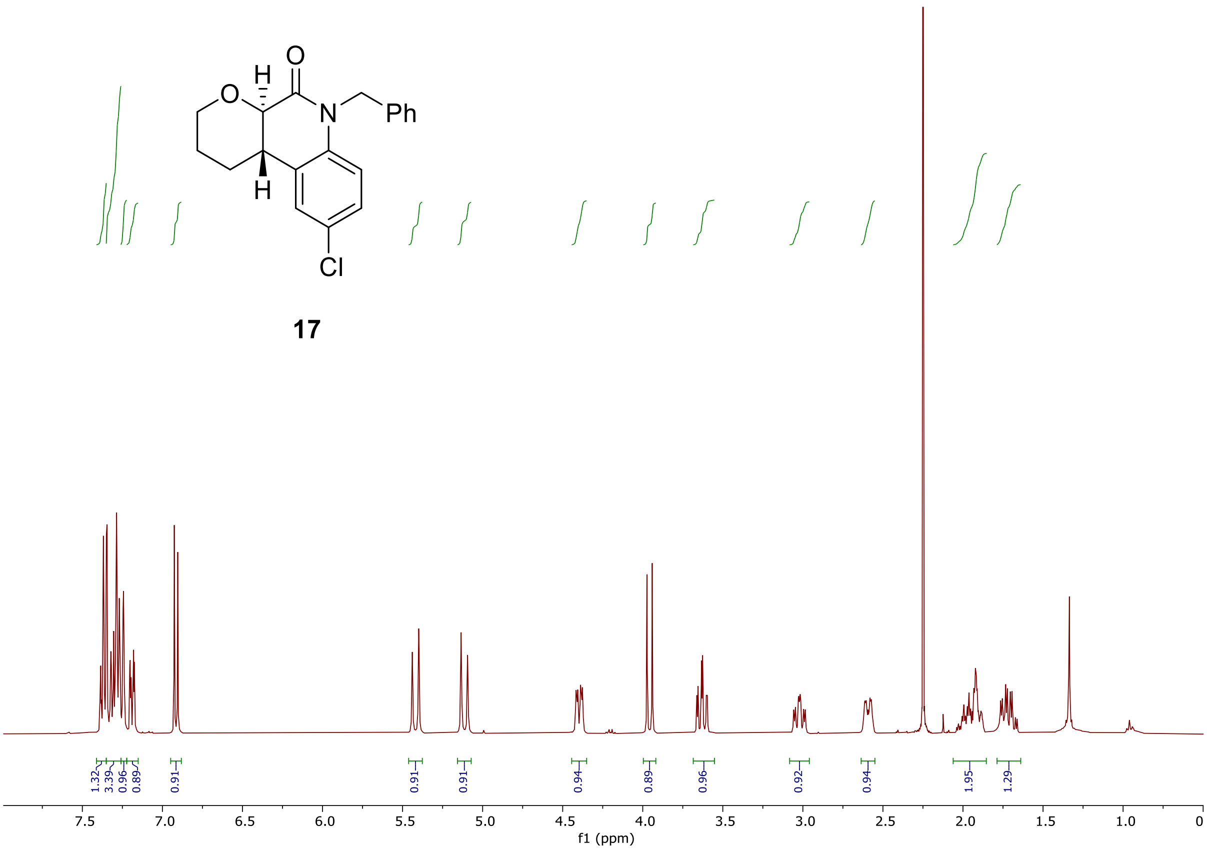

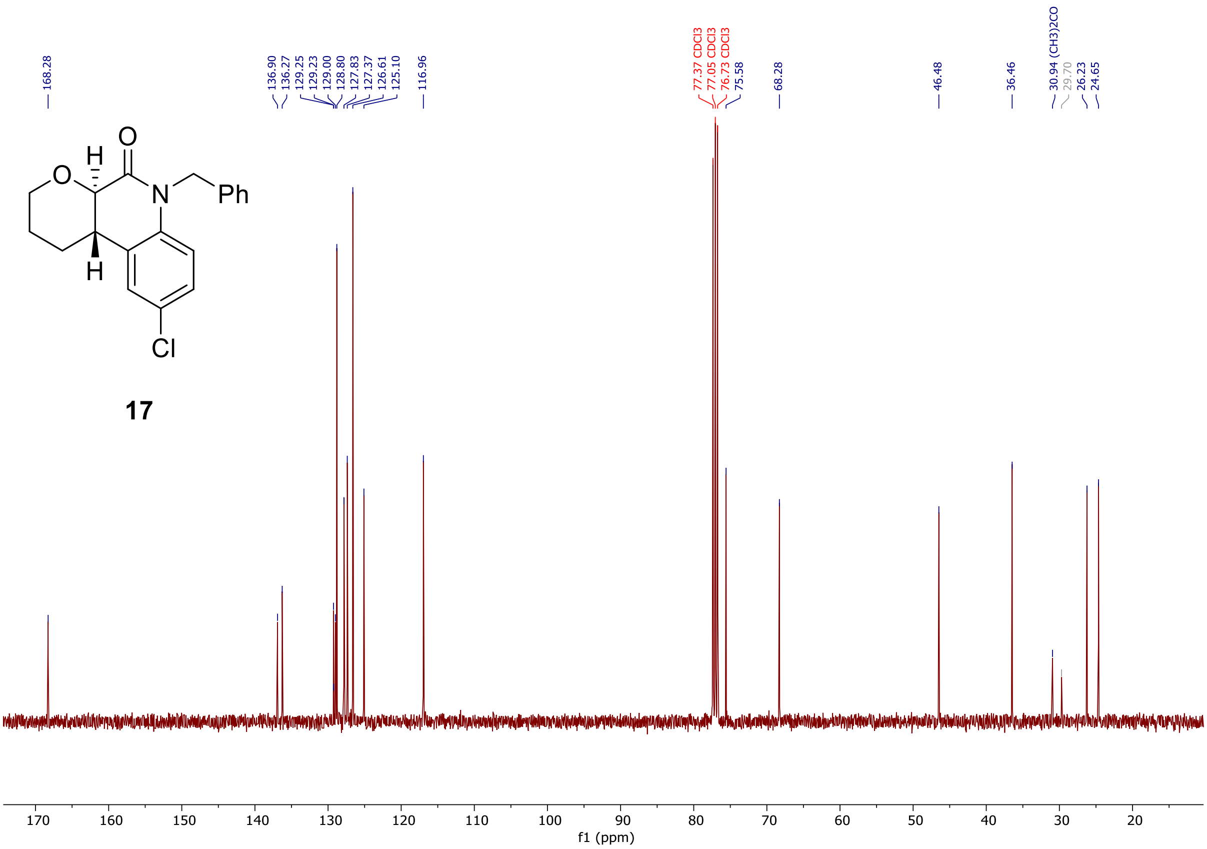

(4a*R*,10b*S*)-9-Bromo-6-methyl-2,3,6,10b-tetrahydro-1*H*-pyrano[2,3-*c*]quinolin-5(4a*H*)-one (18)

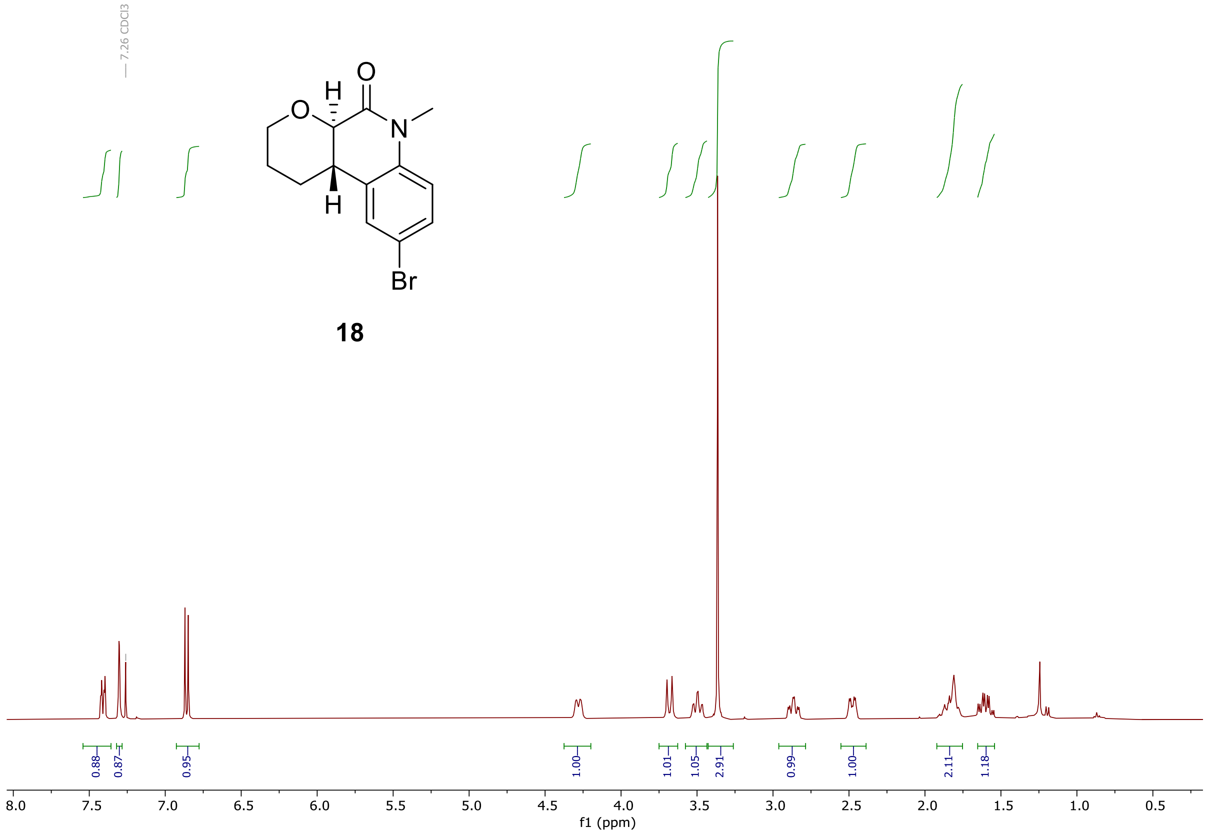

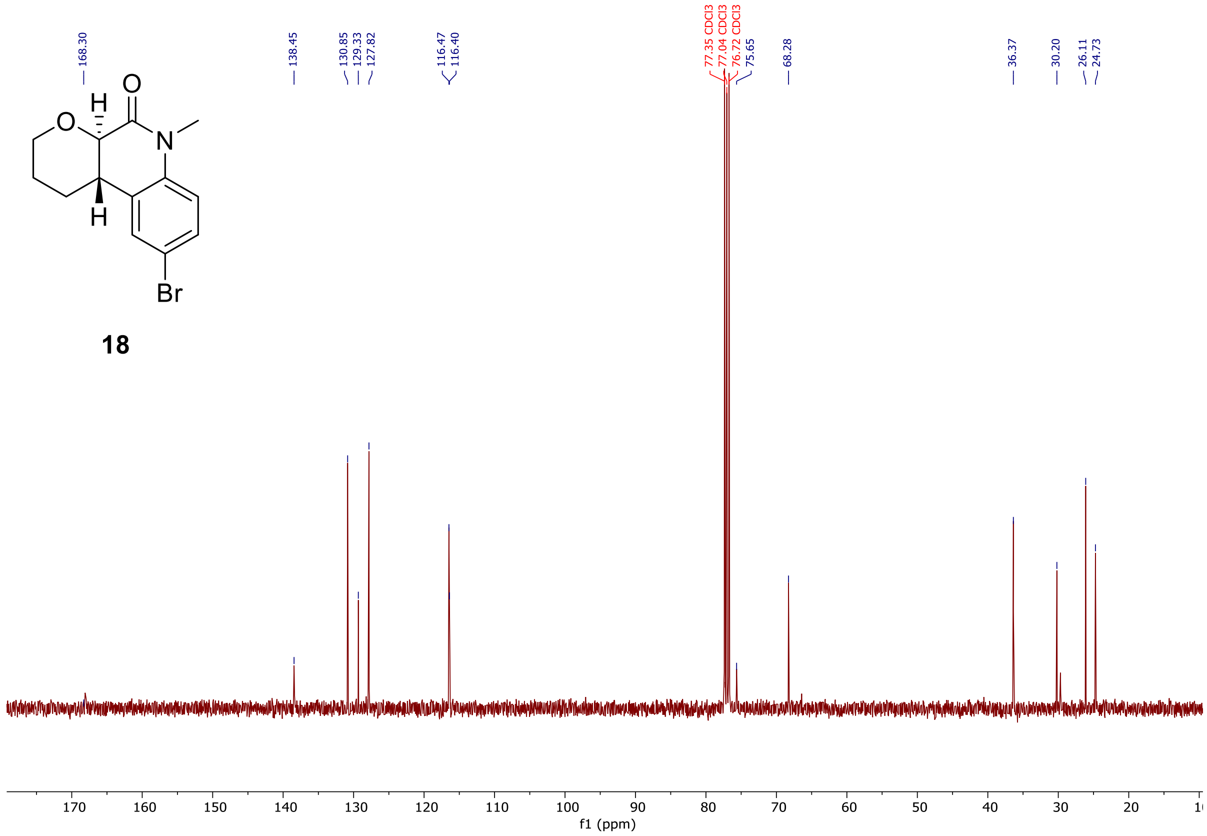

(4a*R*,10b*S*)-6,9-Dimethyl-2,3,6,10b-tetrahydro-1*H*-pyrano[2,3-c]quinolin-5(4a*H*)-one (19)

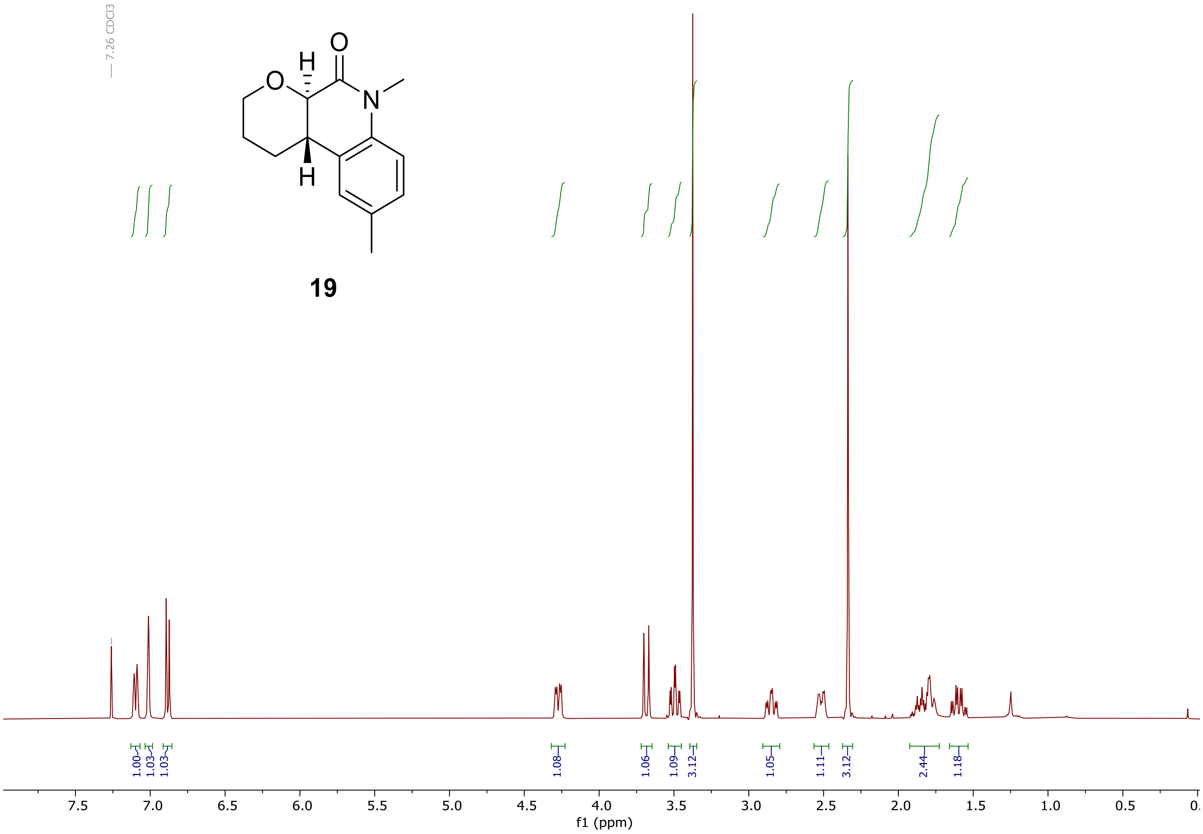

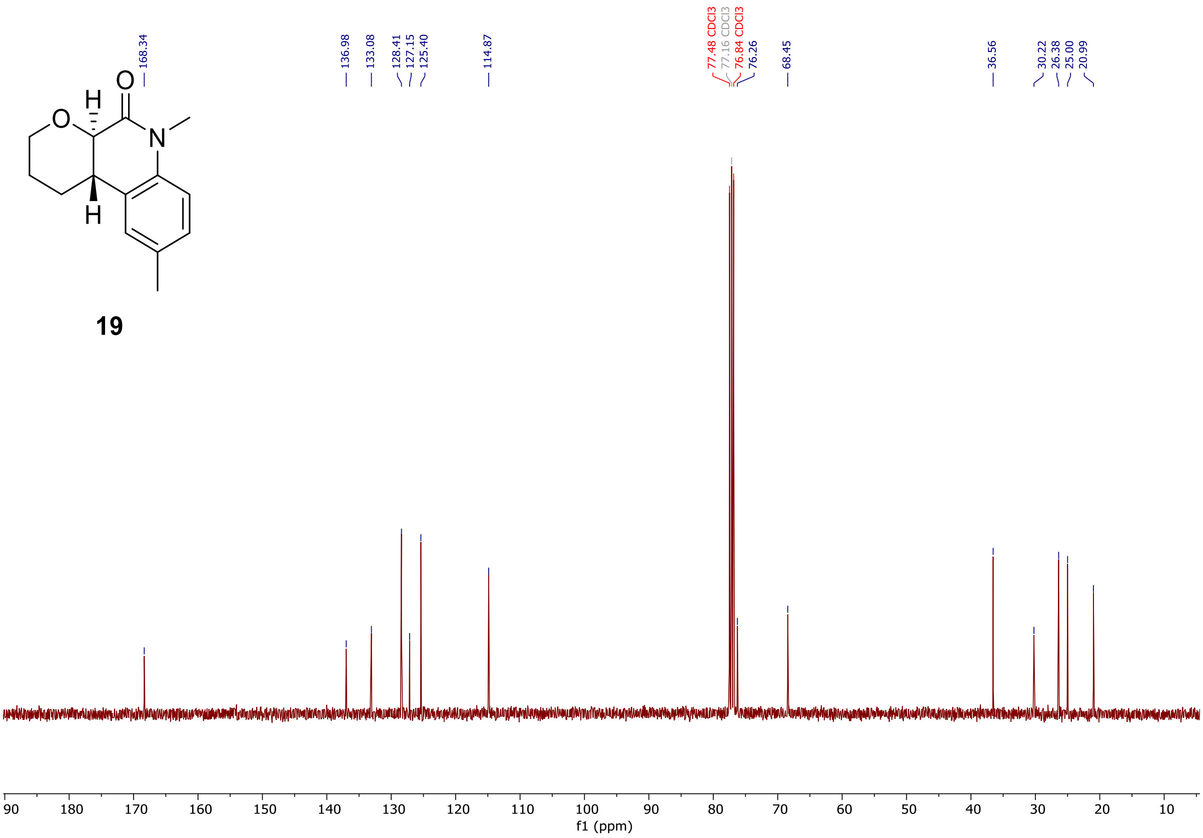

(4a*R*,10b*S*)-6-Methyl-9-(trifluoromethyl)-2,3,6,10b-tetrahydro-1*H*-pyrano[2,3-*c*]quinolin-5(4a*H*)-one (20)

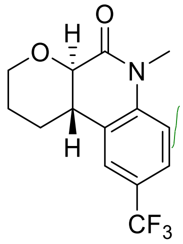

20

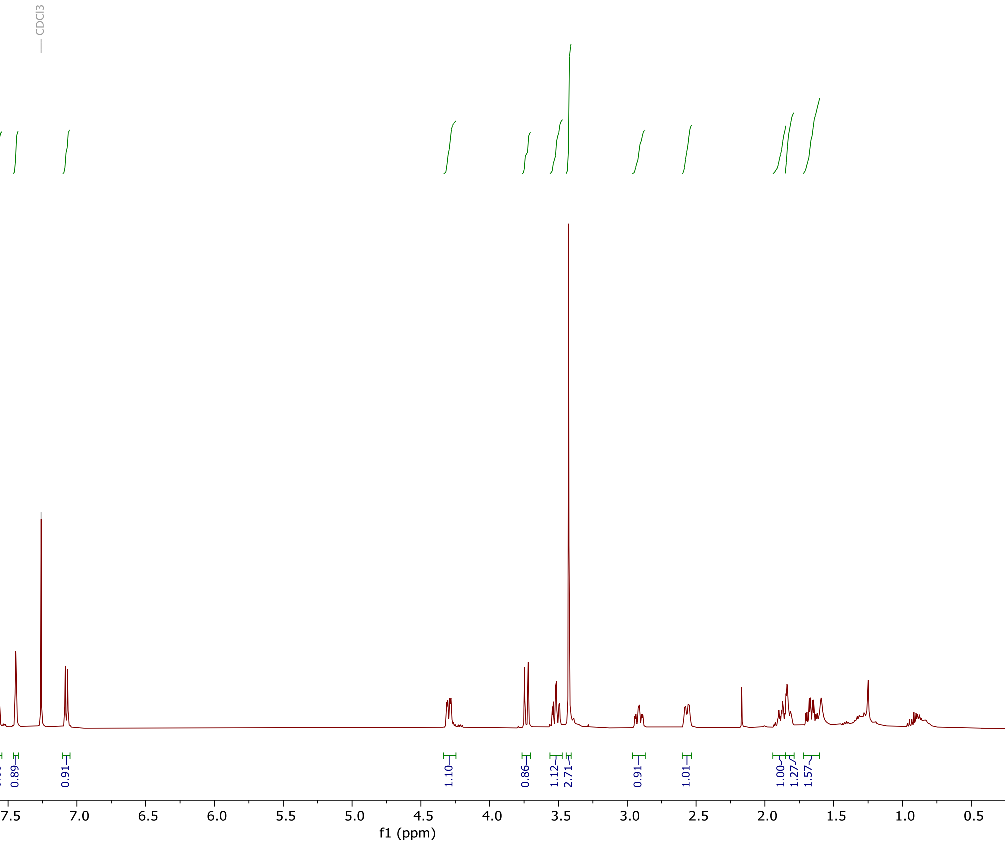

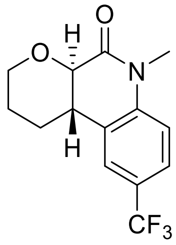

20

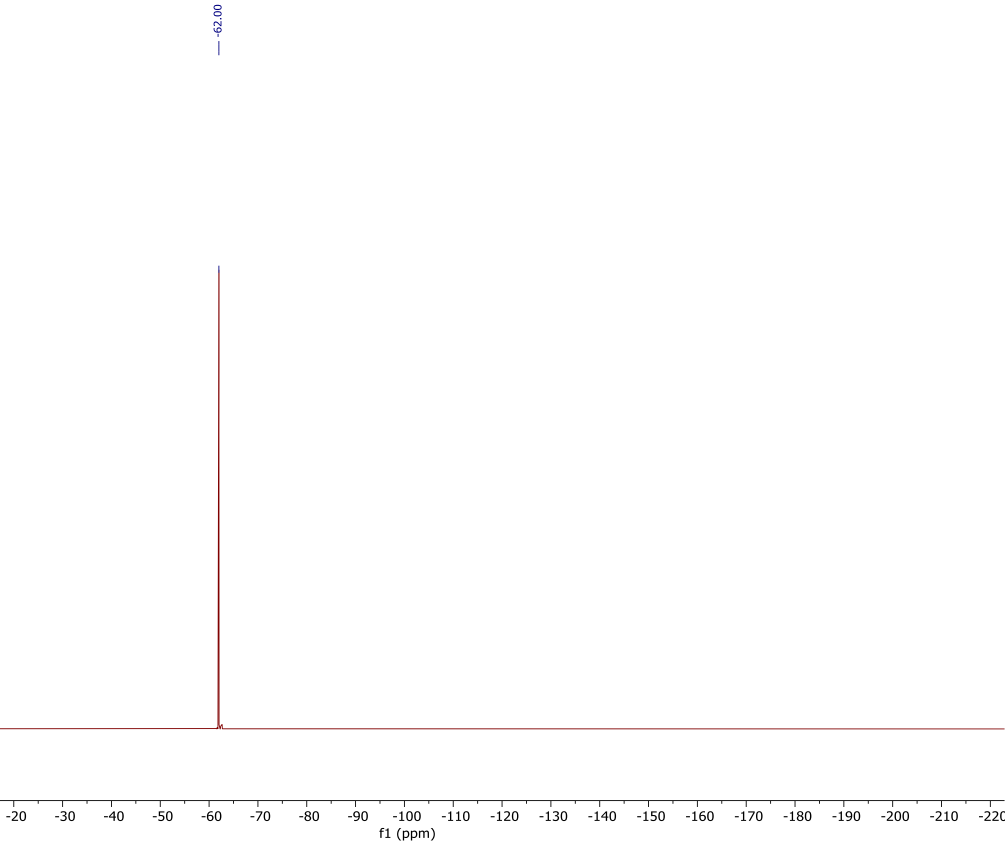

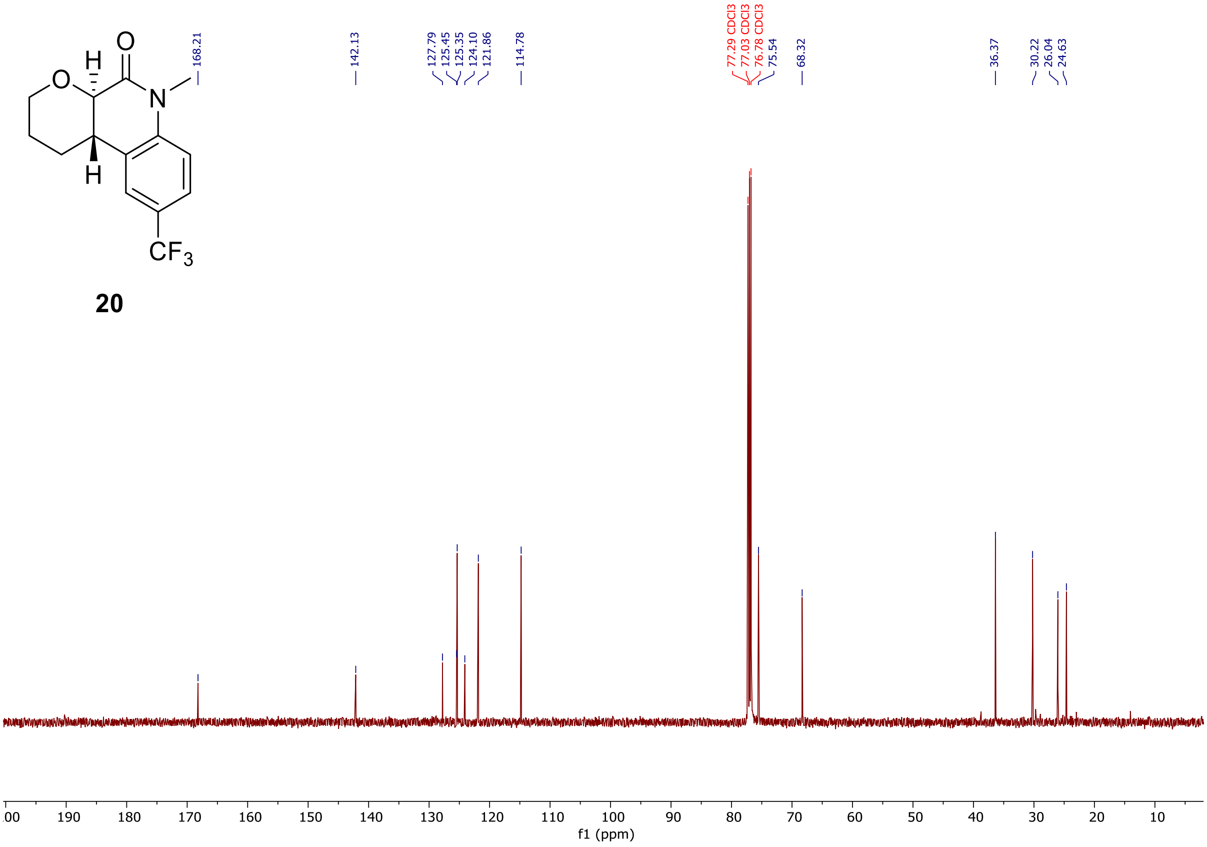

(4a*R*,10b*S*)-9-(*Tert*-butyl)-6-methyl-2,3,6,10b-tetrahydro-1*H*-pyrano[2,3-c]quinolin-5(4a*H*)-one (21)

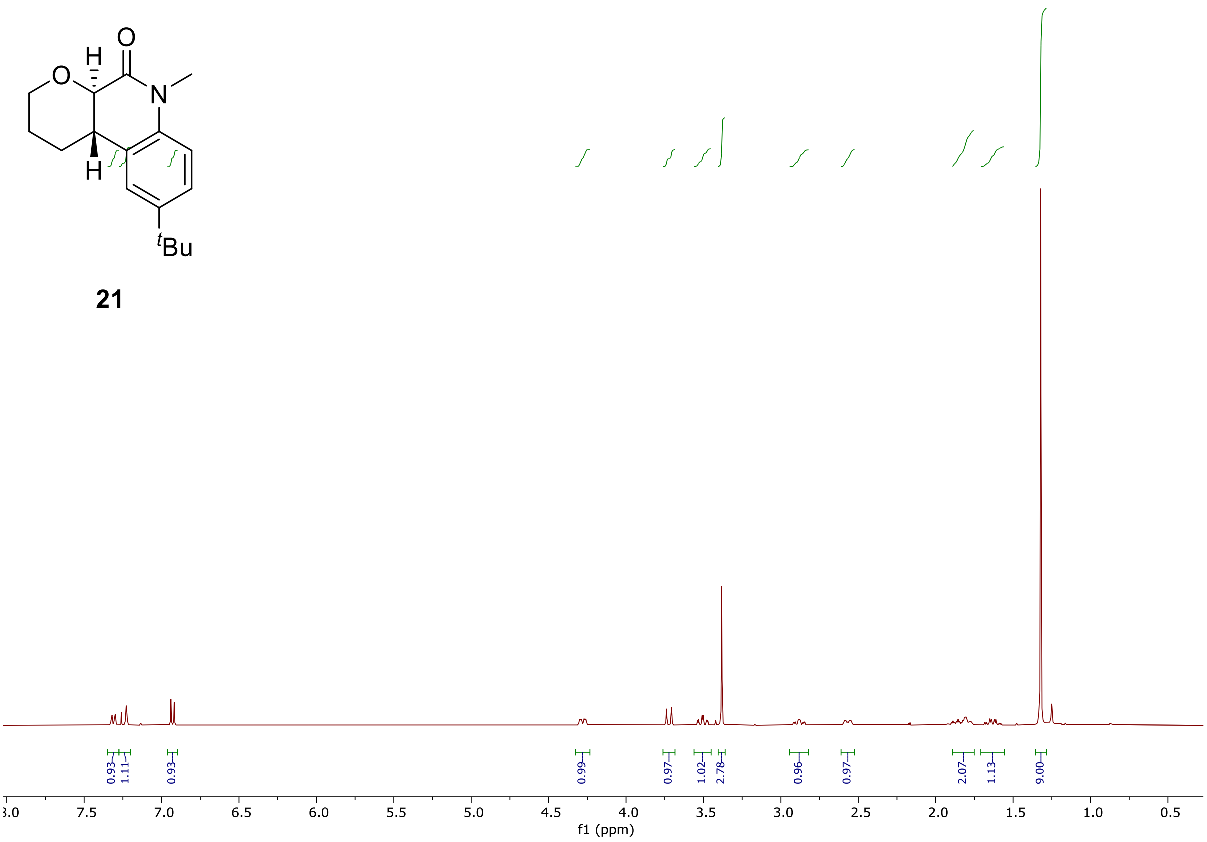

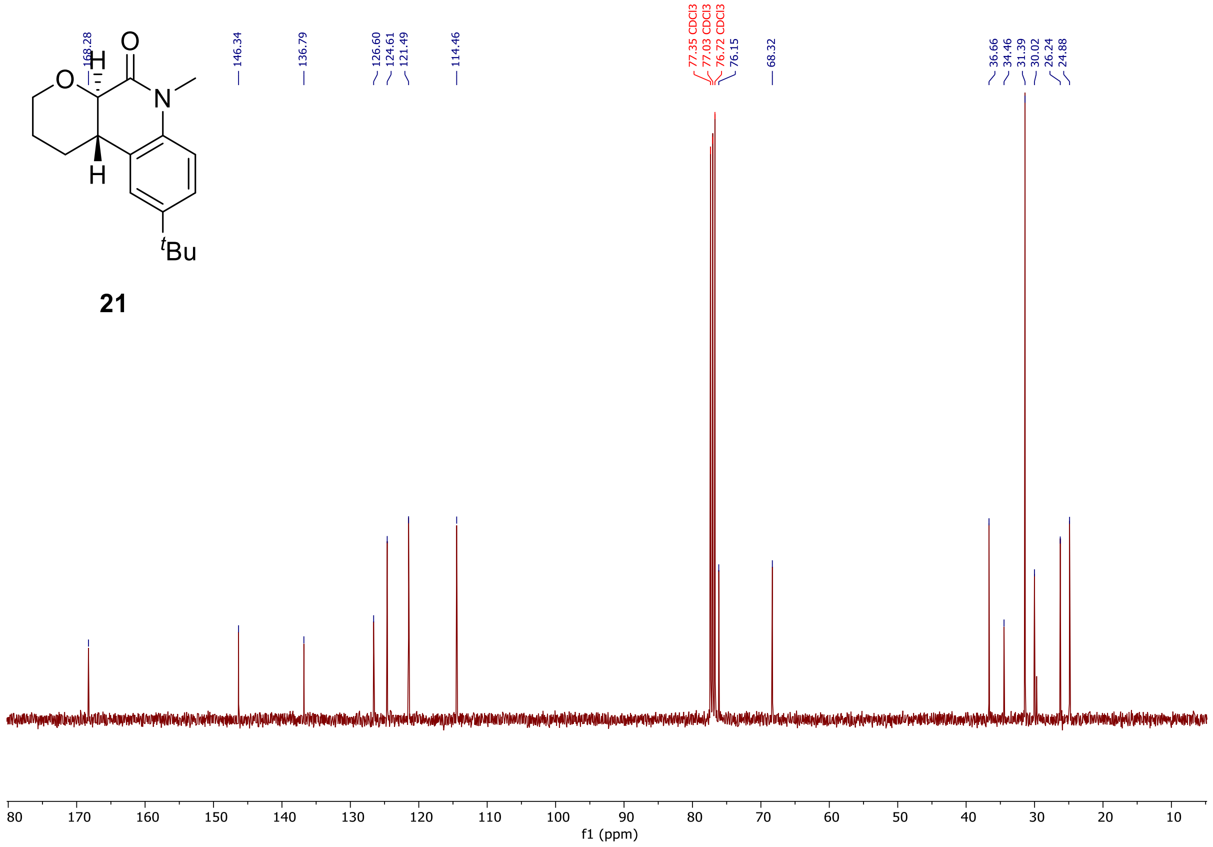

(4a*R*,10b*S*)-9-Methoxy-6-methyl-2,3,6,10b-tetrahydro-1*H*-pyrano[2,3-*c*]quinolin-5(4a*H*)-one (22)

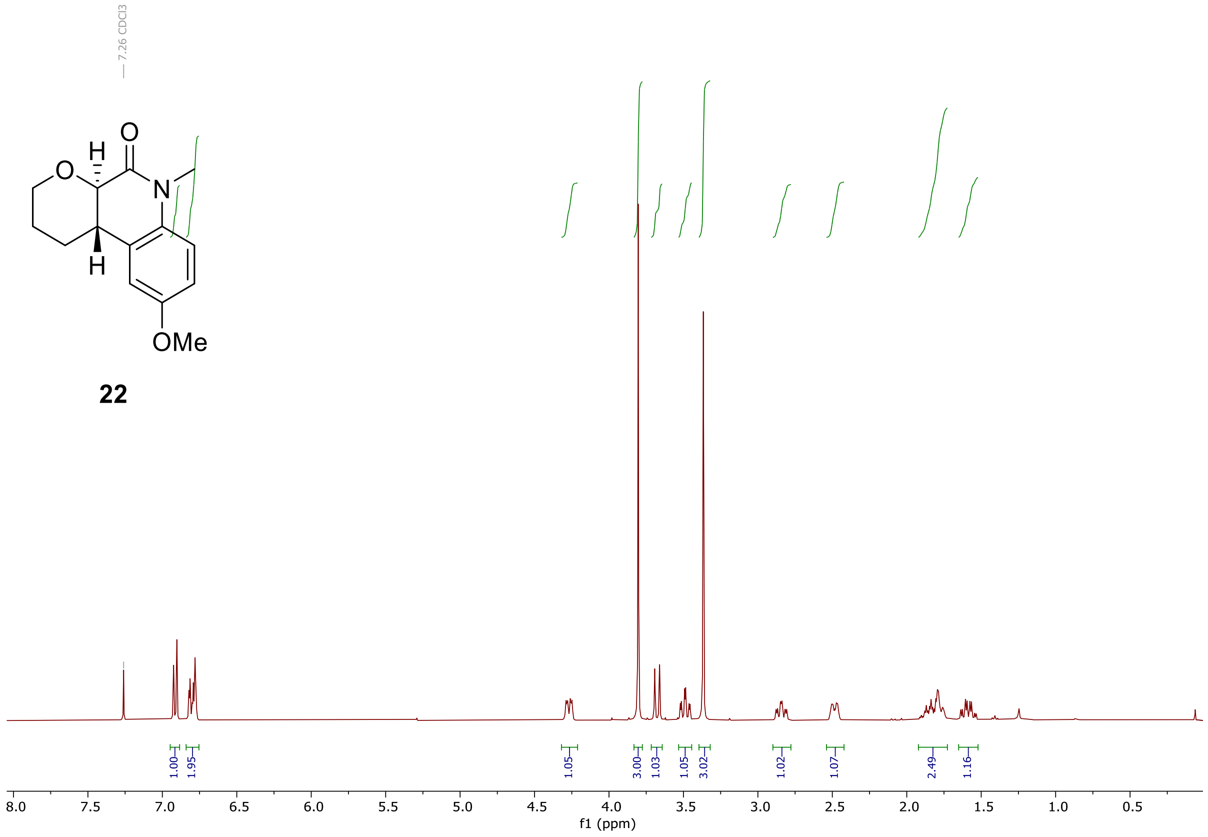

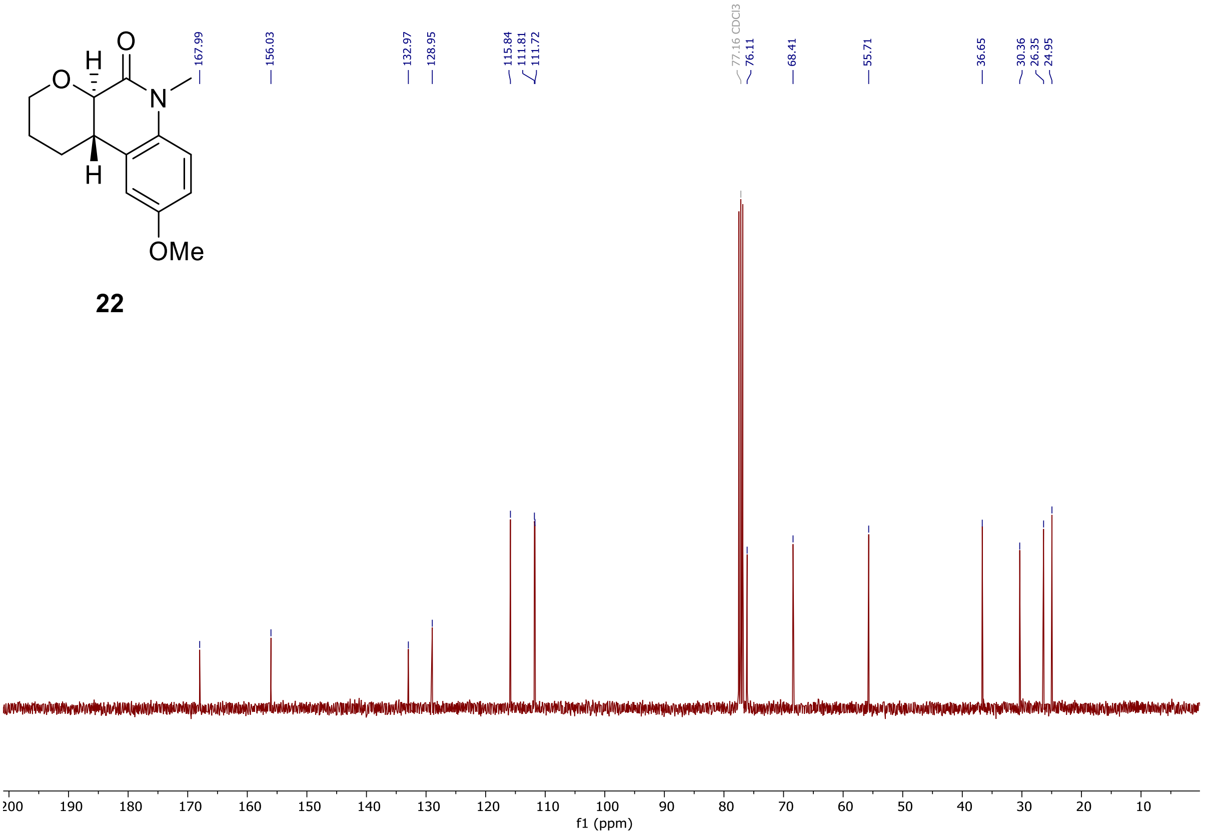

22

(4a*R*,10b*S*)-9-(Methylthio)-6-methyl-2,3,6,10b-tetrahydro-1*H*-pyrano[2,3-*c*]quinolin-5(4aH)-one (23)

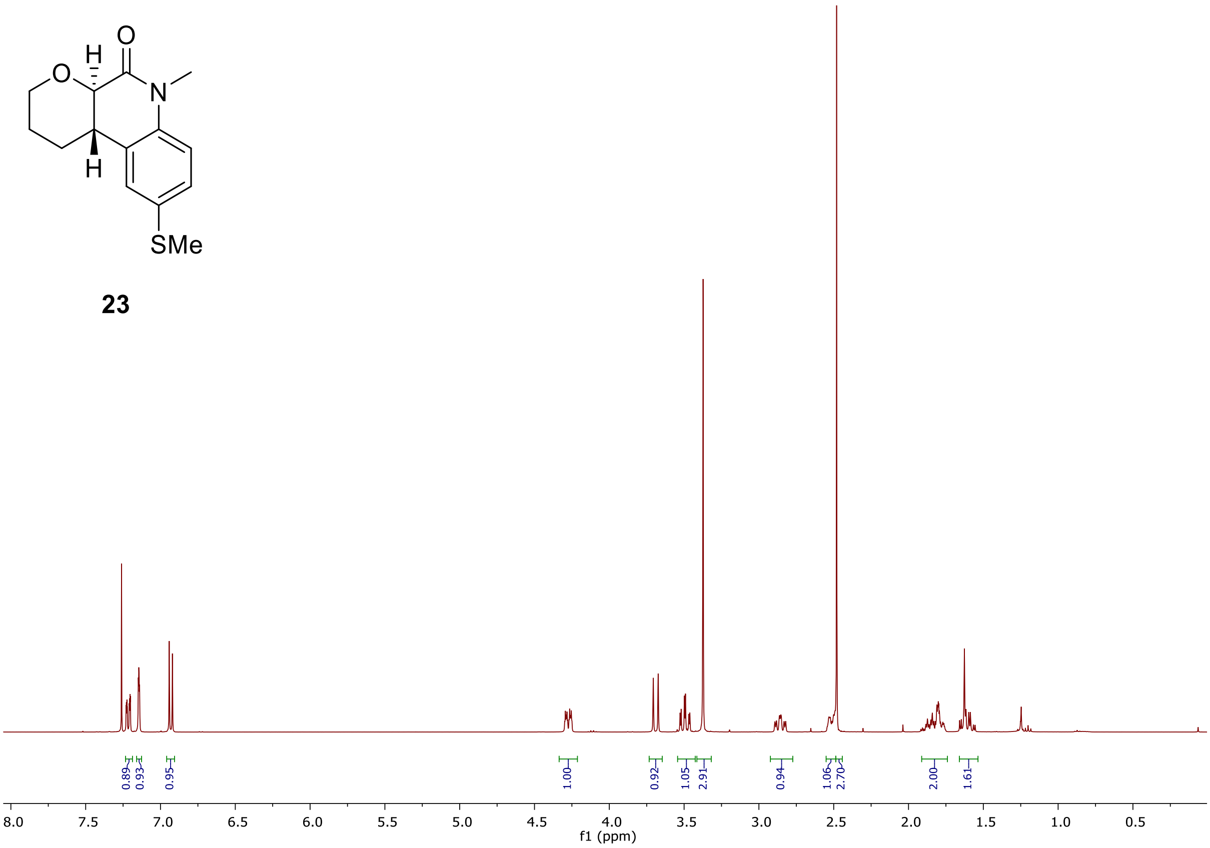

23

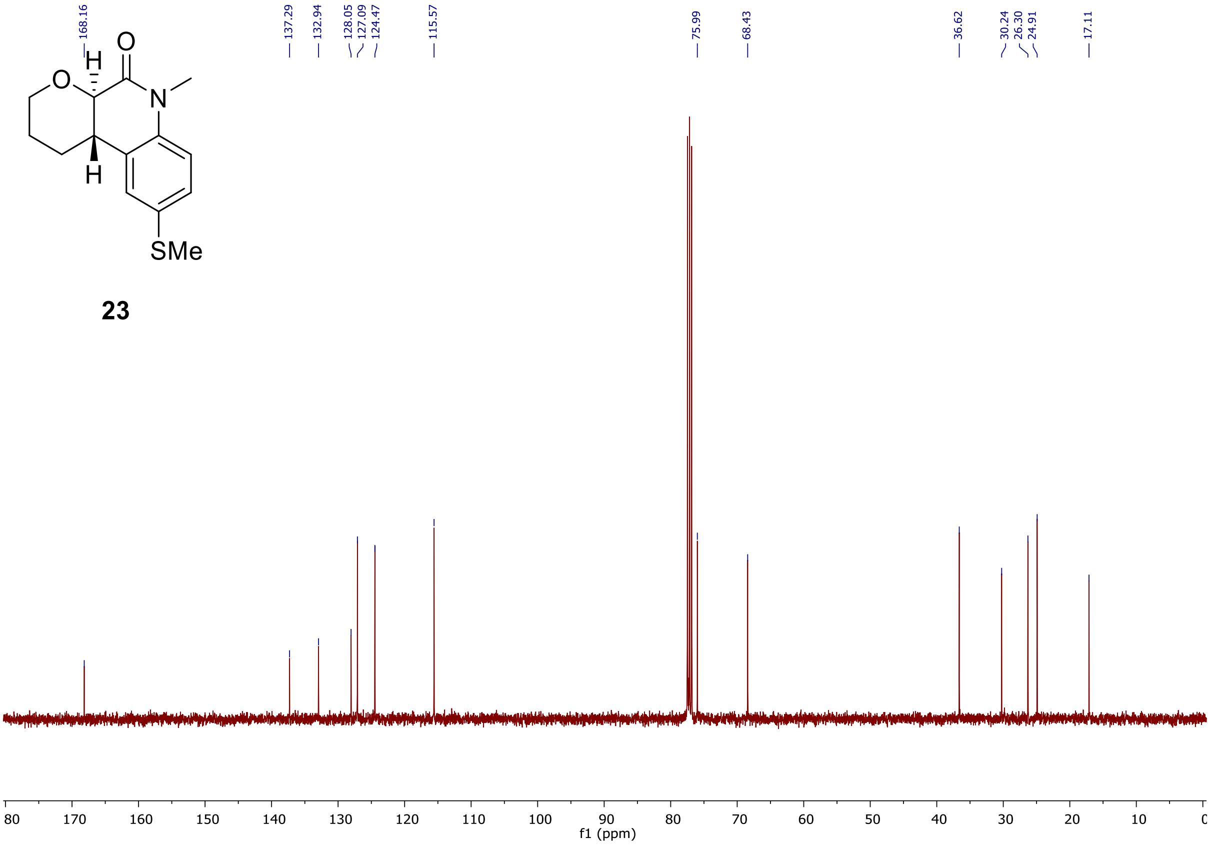

(3a*R*,9b*S*)-5-Methyl-1,2,5,9b-tetrahydrofuro[2,3-*c*]quinolin-4(3a*H*)-one (24)

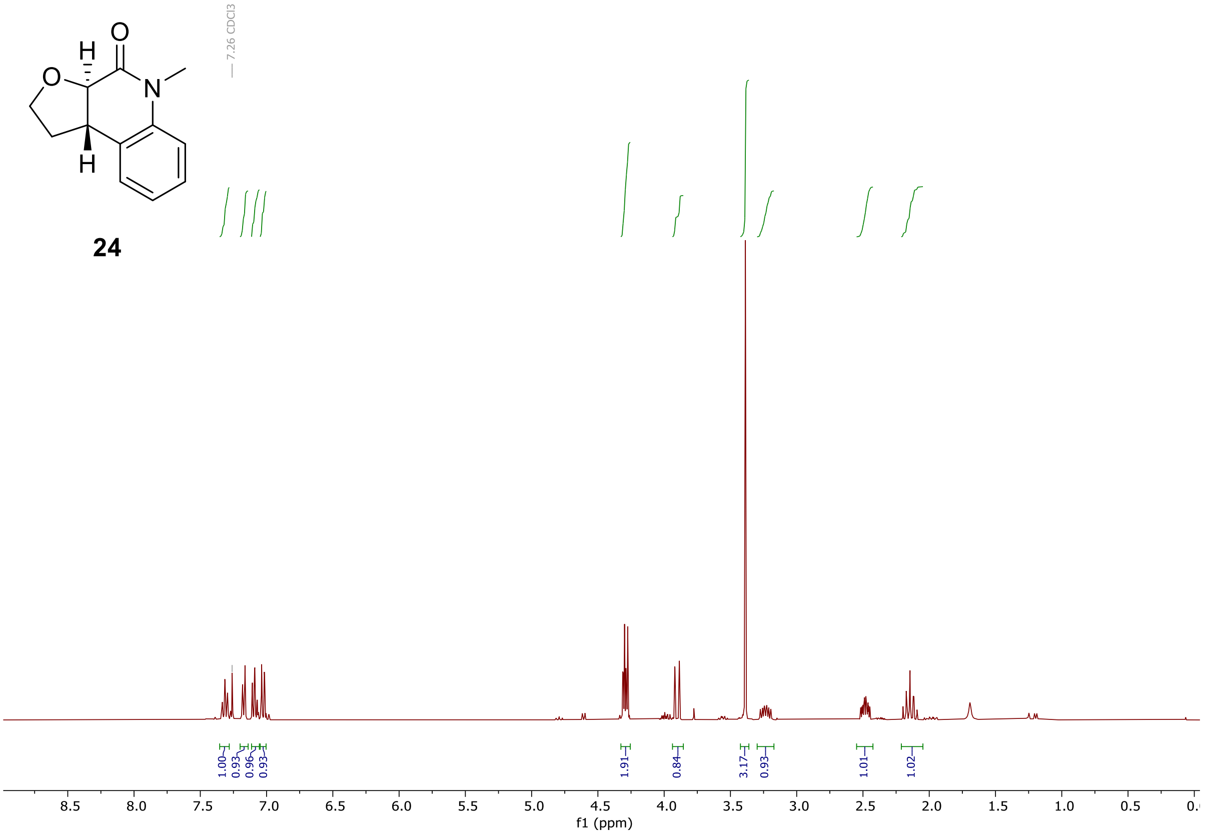

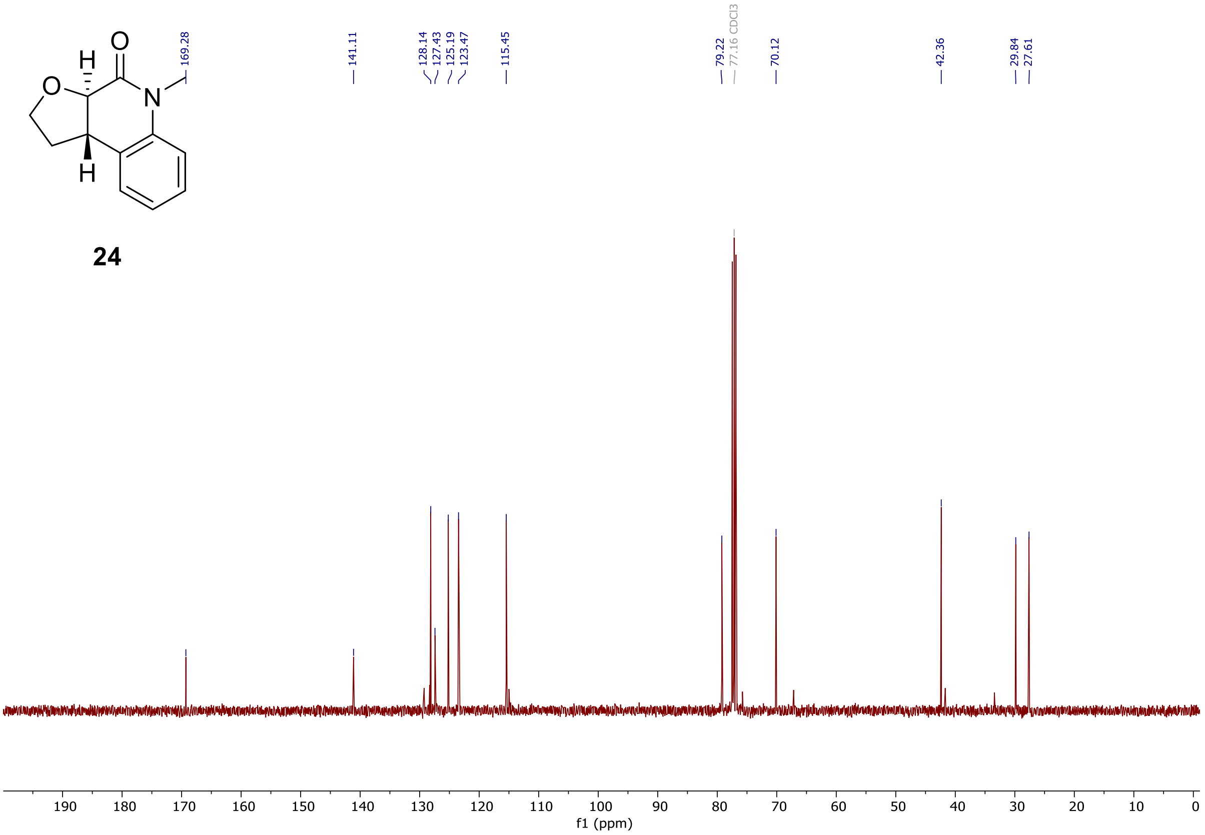

(4a*R*,10b*S*)-6-methyl-2,3,6,10b-tetrahydro-1*H*-pyrano[2,3-*c*]quinolin-5(4a*H*)-one-4a,7,8,9,10-d_5 (30)

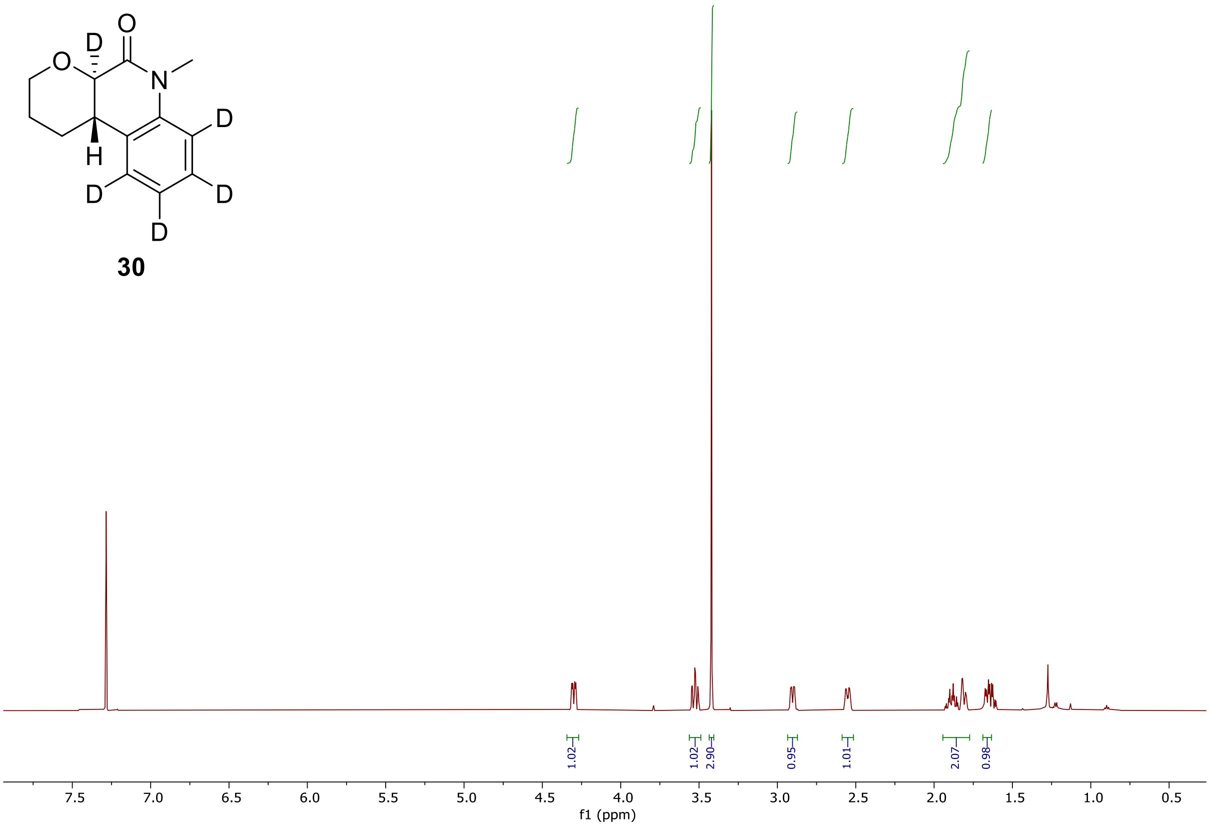

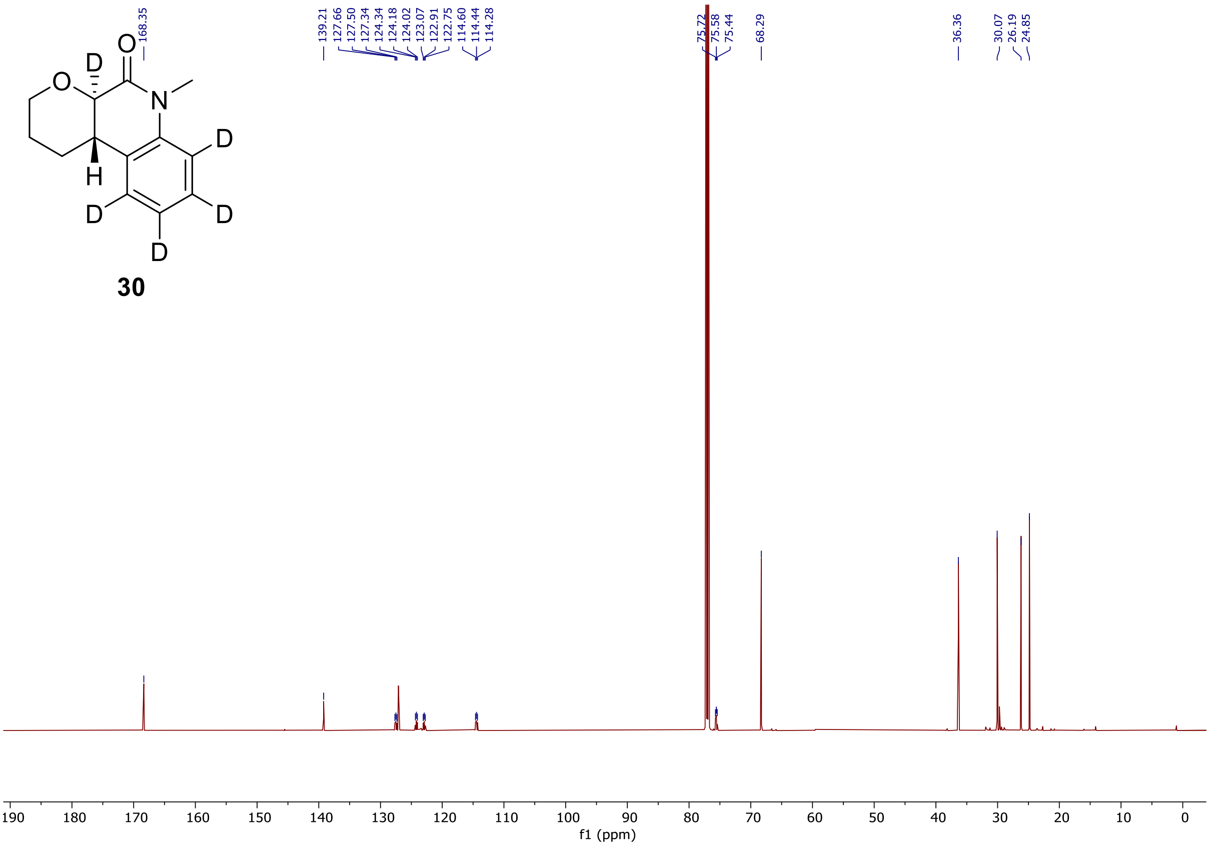

30

(4a*R*,10b*S*)-6-Ethyl-2,3,6,10b-tetrahydro-1*H*-pyrano[2,3-*c*]quinolin-5(4a*H*)-one (S1)

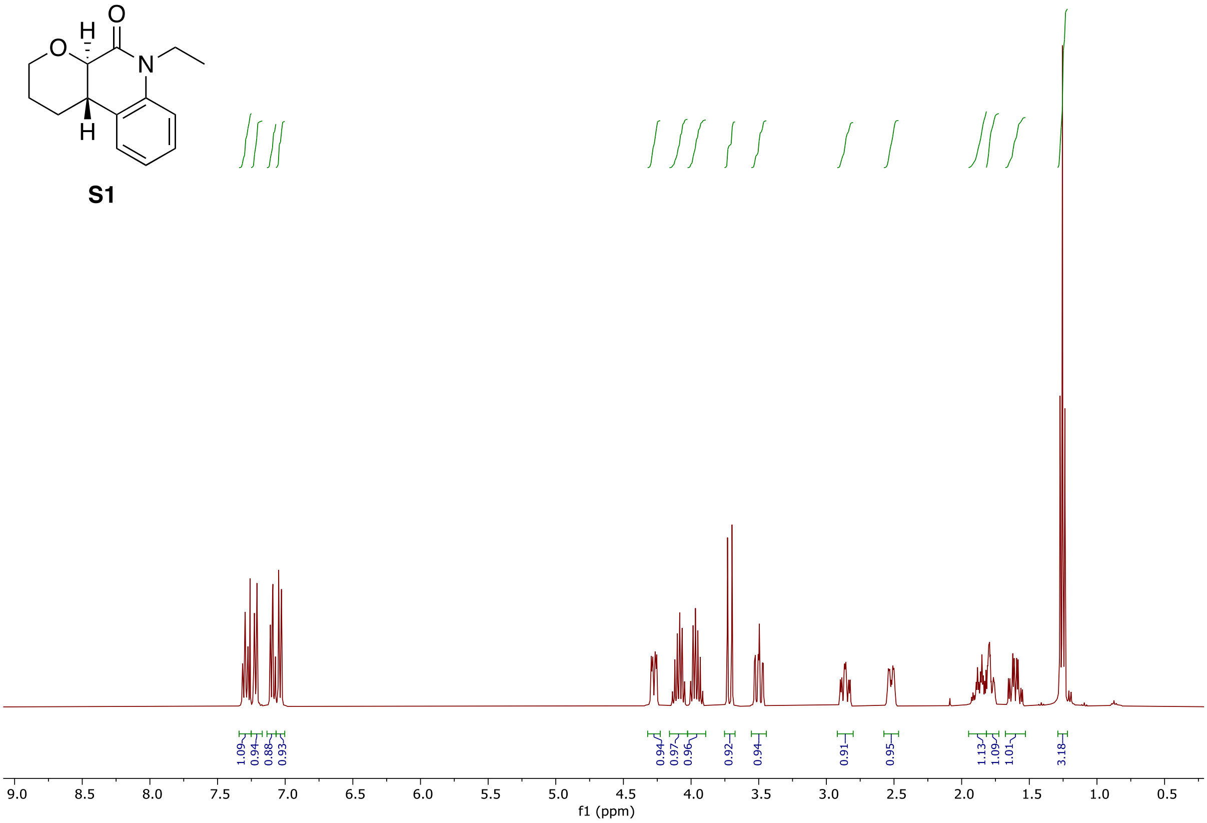

S1

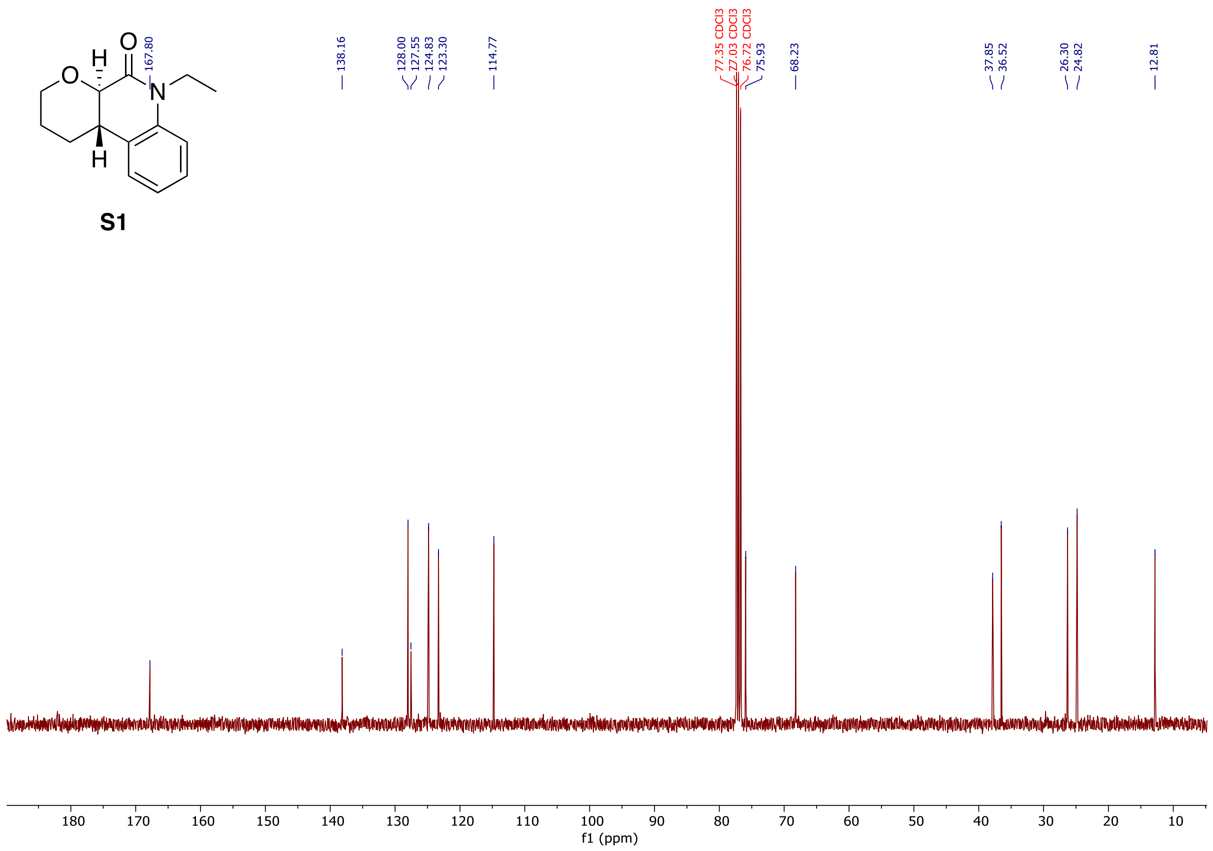

(4a*R*,10b*S*)-9-Chloro-6-methyl-2,3,6,10b-tetrahydro-1*H*-pyrano[2,3-c]quinolin-5(4a*H*)-one (S2)

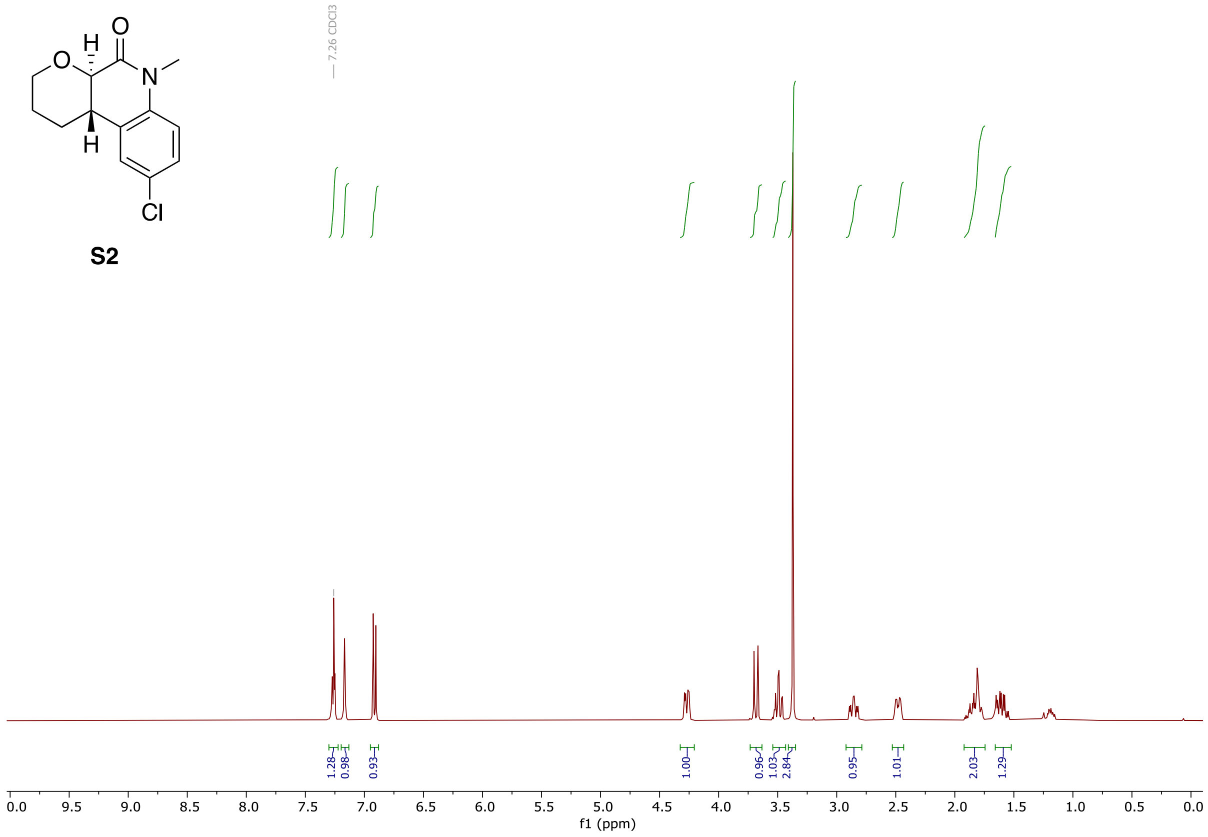

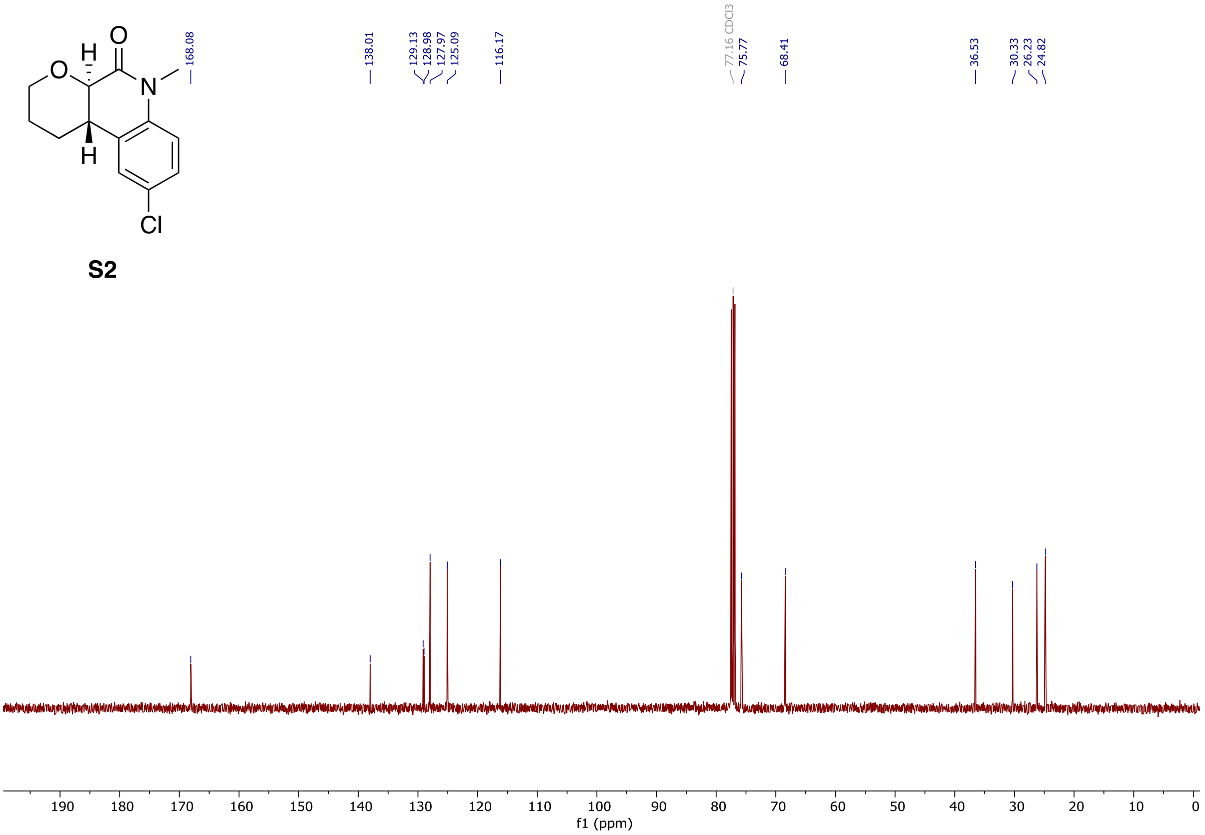

(5a*S*,11b*R*)-7-Methyl-1,2,3,4,7,11b-hexahydrooxepino[2,3-*c*]quinolin-6(5a*H*)-one (S3)

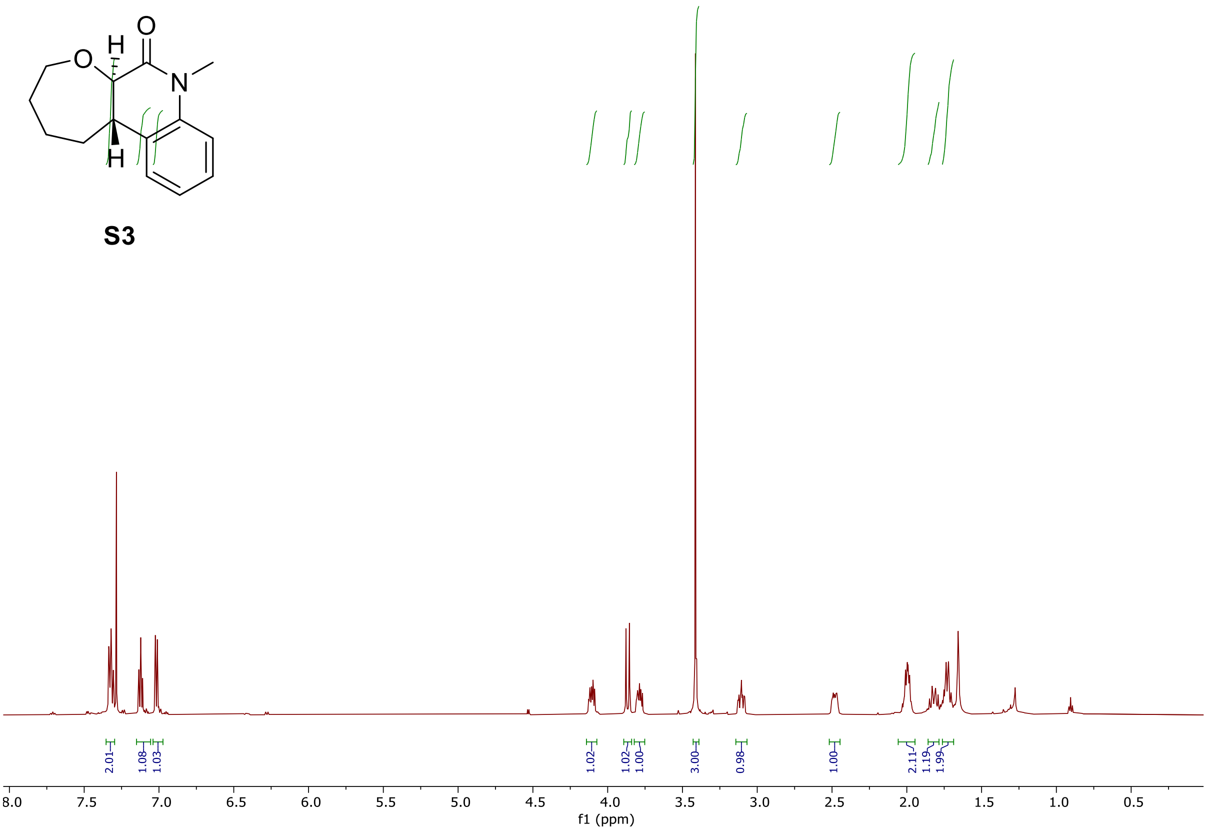

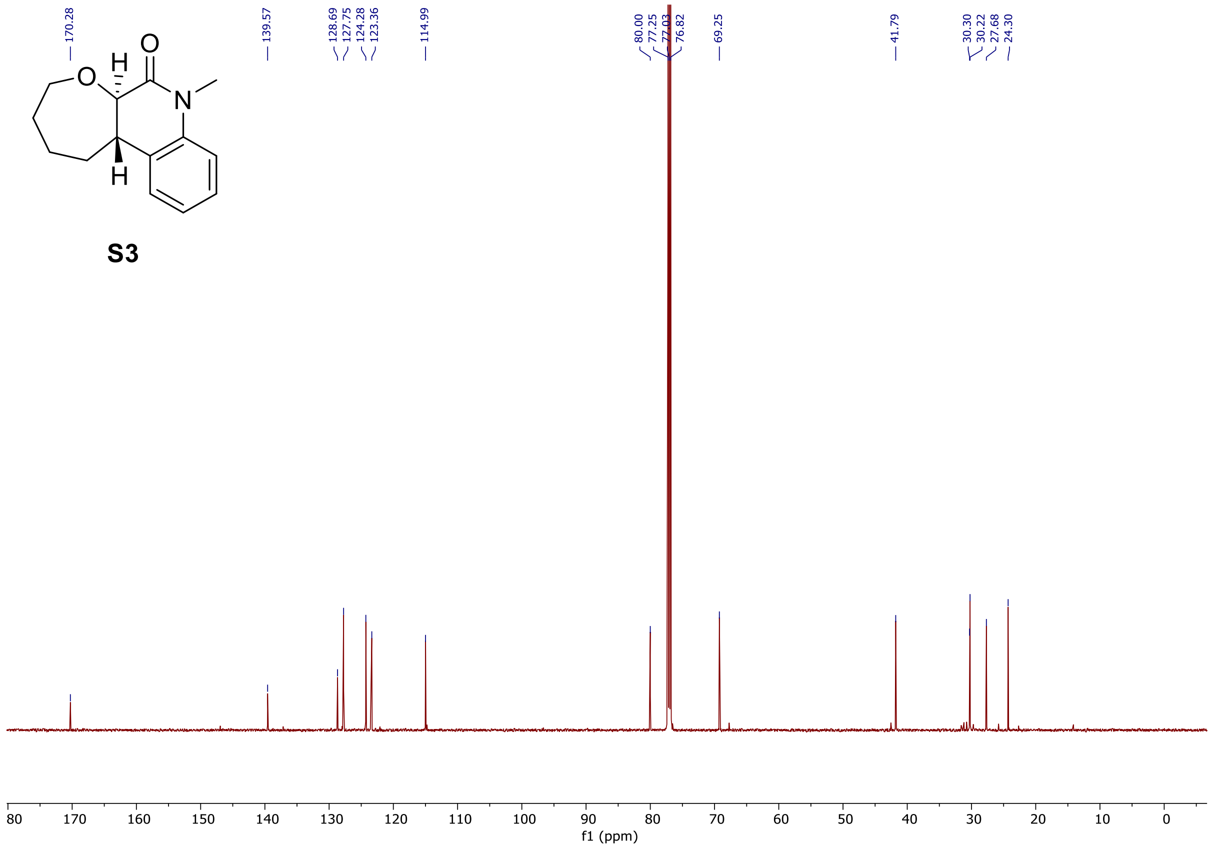

S3
170.28
139.57
128.69
127.75
124.28
123.36
114.99
80.00
77.25
77.03
76.82
69.25
41.79
30.30
30.22
27.68
24.30
180 170 160 150 140 130 120 110 100 90 80 70 60 50 40 30 20 10 0
f1 (ppm)

10. References

1. A. Singh, K. Teegardin, M. Kelly, K. S. Prasad, S. Krishnan, J. D. Weaver. *J. Organomet. Chem.* **2015**, *776*, 51–59.
2. E. D. Nacsa, D. W. C. MacMillan, *J. Am. Chem. Soc.* **2018**, *140*, 3322-3330.
3. W. Li, X. Liu, X. Hao, Y. Cai, L. Lin, Feng, X. *Angew. Chem. Int. Ed.* **2012**, *51*, 8644–8647.
4. (a) F. T. Oakes, F. A. Yang, J. F. Sebastian. *J. Org. Chem.* **1982**, *47*, 3094–3097. (b) J. J. Koening, T. Arndt, N. Gildermeister, J. -M. Neudörfl, M. Breugst. *J. Org. Chem.* **2019**, *84*, 7587–7605
5. J. L. Brennan, T. E. Keyes, R. J. Forster, *Langmuir*, **2006**, *22*, 10754-10761.
6. Y.-J. Yuan, Z.-T. Yu, H.-L. Gao, Z.-G. Zou, C. Zheng, W. Huang, *Chem. Eur. J.* **2013**, *19*, 6340-6349.
7. J. R. Ochola, M. O. Wolf, *Org. Biomol. Chem.* **2016**, ***14***, 9088-9092.
8. R. Schmidt, S. E. Braslavsky, *Pure Appl. Chem.* **1989**, *61*, 187–210.
9. (a) K. Tanaka, O. Kakinoki, F. Toda *Chem. Commun.* **1995**, 1053–1054.(b) M. J. Oddy, D. A. Kusza, W. F. Petersen. *Org. Lett.* **2021**, *23*, 8963–8967.
10. M. Montalti, A. Credi, L.; Prodi, M. T. Gandolfi, Handbook of Photochemistry. **2006**.
11. Xiong, Y. Photoswitchable Dyes for Super-Resolution Microscopy, DPhil Thesis, University Of Oxford, 2018.
12. C. A. Parker, *Trans. Faraday Soc.* **1954**, *50*, 1213–1221.
13. I. R. Laskar, T. M. Chen, *Chem. Mater.* **2004**, *16*, 111–117.
14. J. Cosier, A. M. Glazer, *J. Appl. Crystallogr.* **1986**, *19*, 105–107.
15. P. Parois, R. I. Cooper, A. L. Thompson, *Chem. Cent. J.* **2015**, *9*, 30.
16. R. I. Cooper, A. L. Thompson, D. J. Watkin, *J. Appl. Crystallogr.* **2010**, *43*, 1100–1107.
17. L. Palatinus, G. Chapuis, *J. Appl. Crystallogr.* **2007**, *40*, 786–790.
18. A. L. Spek, *Acta Crystallogr. Sect. C Struct. Chem.* **2015**, *71*, 9–18.
19. Y. Zhao, D. G. Truhlar, *Theor. Chem. Acc*. **2008**, *120*, 215 – 241.
20. a) F. Weigend, R. Ahlrichs. *Phys. Chem. Chem. Phys.* **2005**, *7*, 3297 – 3305; b) F. Weigend *Phys. Chem. Chem. Phys.* **2006**, *8*, 1057 – 1065.
21. a) W. J. Hehre, R. Ditchfield, J. A. Pople, *J. Chem. Phys.* **1972**, *56*, 2257 – 2261; b) P. C. Hariharan, J. A. Pople, *Theor. Chim. Acta.* **1973**, *28*, 213 – 222; c) R. Krishnan, J. S. Binkley, R. Seeger, J. A. Pople, *J. Chem. Phys.*, **1980**, *72*, 650 – 654; d) A. D. McLean, G. S. Chandler, *J. Chem. Phys.* **1980**, *72*, 5639 – 5648; e) M. M. Francl, W. J. Pietro, W. J. Hehre, J. S. Binkley, M. S. Gordon, D. J. DeFrees, J. A., Pople, *J. Chem. Phys.*, **1982**, *77*, 3654 – 3665.
22. S. Grimme, J. Antony, S. Ehrlich, H. Krieg, *J. Chem. Phys. 2010*, **132**, 154104 – 154119.
23. a) N. Munster, N. A. Parker, R. S. Paton, M. D. Smith, *Angew. Chem. Int. Ed.*, **2017**, *56*, 9468-9472; b) M. V. Popescu, A. Mekereeya, J. V. Alegre-Requena, R. S. Paton, M. D. Smith, *Angew. Chem. Int. Ed.*, **2020**, *59*, 23020-23024; c) P. C. St. John, Y. Guan, S. Kim, R. S. Paton, *Nat. Commun.*, **2020**, *11*, 2328; d) S. S. S. V., P. C. St. John, R. S. Paton, *Chem. Sci.*, **2021**, *12*, 13158-13166.
24. a) V. Barone, M. Cossi, *J. Phys. Chem. A*, **1998**, *102*, 1995-2001; b) M. Cossi, N. Rega, G. Scalmani, V. Barone, *J. Comp. Chem*, **2003**, *24*, 669-681.
25. A. V. Marenich, C. J. Cramer, D. G. Truhlar, *J. Phys. Chem. B*, **2009**, *113*, 6378-6396.
26. Gaussian 16, Revision C.01, M. J. Frisch, G. W. Trucks, H. B. Schlegel, G. E. Scuseria, M. A. Robb, J. R. Cheeseman, G. Scalmani, V. Barone, G. A. Petersson, H. Nakatsuji, X. Li, M. Caricato, A. V. Marenich, J. Bloino, B. G. Janesko, R. Gomperts, B. Mennucci, H. P. Hratchian, J. V. Ortiz, A. F. Izmaylov, J. L. Sonnenberg, D. Williams-Young, F. Ding, F. Lipparini, F. Egidi, J. Goings, B. Peng, A. Petrone, T. Henderson, D. Ranasinghe, V. G. Zakrzewski, J. Gao, N. Rega, G. Zheng, W. Liang, M. Hada, M. Ehara, K. Toyota, R. Fukuda, J. Hasegawa, M. Ishida, T. Nakajima, Y. Honda, O. Kitao, H. Nakai, T. Vreven, K. Throssell, J. A. Jr. Montgomery, J. E. Peralta, F. Ogliaro, M. J. Bearpark, J. J. Heyd, E. N. Brothers, K. N. Kudin, V. N. Staroverov, T. A. Keith, R. Kobayashi, J. Normand, K. Raghavachari, A. P. Rendell, J. C. Burant, S. S. Iyengar, J. Tomasi, M. Cossi, J. M. Millam, M. Klene, C. Adamo, R. Cammi, J. W. Ochterski, R. L. Martin, K. Morokuma, O. Farkas, J. B. Foresman, D. J. Fox, Gaussian, Inc., Wallingford CT, 2016.
27. R. Ketkaew, Y. Tantirungrotechai, P. Harding, G. Chastanet, P. Guionneau, M. Marchivie, D. J. Harding, *Dalton Trans.*, **2021**, *50*, 1086-1096.
28. The PyMOL Molecular Graphics System, version 2.0.7, Schrodinger, LLC.
29. E. D. Glendening, C. R. Landis, F. Weinhold, *J. Comput. Chem*. **2013**, *34*, 1429-1437.
30. a) EasyMECP version 0.3.2, J. Rodriguez-Guerra, **2020**, 10.5281/zenodo.42924322; b) N. J. Harvey, M.

Aschi, H. Schwarz, W. Koch, *Theor. Chem. Acc.* **1998**, *99*, 95-99.
31. K. Fukui, *Acc. Chem. Res.* **1981**, *14*, 363−368.
32. S. Grimme, *Chem. Eur. J.* **2012**, 18, 9955−9964.
33. V. S. Bryantsev, M. S. Diallo, W. A. Goddard III, *J. Phys. Chem. B* **2008**, *112,* 9709–9719.
34. R. E. Plata, D. A. Singleton, *J. Am. Chem. Soc,* **2015**, *137*, 3811–3826.
35. A. A. Isse, A. J. Gennaro, *J. Phys. Chem. B,* **2010**, *114*, 7894.
36. J. W. Diggle, A. J. Parker, *Aust. J. Chem.* **1974**, *27*, 1617-1621.
37. a) A. D. Becke, *J. Chem. Phys.* **1993**, *98*, 5648-5652; b) C. Lee, W. Yang, R. G. Parr, *Phys. Rev. B*, **1988**, *37*, 785-789; c) S. H. Vosko, L. Wilk, M. Nusair, *Can. J. Phys*., **1980**, *58*, 1200-1211; d) P. J. Stephens, F. J. Devlin, C. F. Chabalowshi, M. J. Frisch, *J. Phys. Chem.*, **1994**, *98*, 11623-11627.
38. a) J. -D. Chai, M. Head-Gordon, *Phys. Chem. Chem. Phys.* **2008**, *10*, 6615-6620; b) J. -D. Chai, M. Head-Gordon, *J. Chem. Phys.* **2008**, *128*, 084106.
